# Decreases in different Dnmt3b activities drive distinct development of hematologic malignancies in mice

**DOI:** 10.1016/j.jbc.2021.100285

**Published:** 2021-01-13

**Authors:** Katarina Lopusna, Pawel Nowialis, Jana Opavska, Ajay Abraham, Alberto Riva, Staci L. Haney, Rene Opavsky

**Affiliations:** 1 Department of Anatomy and Cell Biology, University of Florida College of Medicine, Gainesville, Florida, USA; 2 ICBR Bioinformatics, Cancer and Genetics Research Complex, University of Florida, Gainesville, Florida, USA; 3 Department of Internal Medicine, University of Nebraska Medical Center, 985950 Nebraska Medical Center, Omaha, Nebraska, USA

**Keywords:** DNA methylation, hematologic malignancies, Dnmt3b, lymphoma, leukemia, AF, accessory function, BCLs, B-cell lymphomas, CA, catalytic activity, CLL, chronic lymphocytic leukemia, COBRA, combined bisulfite restriction analysis, CTCLs, cutaneous T-cell lymphomas, DMCs, differentially methylated cytosines, DMRs, differentially methylated region, DNMTs, DNA methyltranferases, GSEA, gene set enrichment analysis, HDACs, histone deacetylases, ICF, immunodeficiency, centromeric instability, and facial anomalies syndrome, IPA, ingenuity pathway analysis, MBL, monoclonal B-cell lymphocytosis, MPD, myeloproliferative disease, MTCL, MYC-induced T-cell lymphomas, PTCL, peripheral T-cell lymphoma, PTCL-NOS, peripheral T-cell lymphoma–not otherwise specified, T-ALL, T-cell acute lymphoblastic leukemia, TCL, T-cell lymphoma, TS, tumor suppressor, TSS, transcription start site, WAT, white adipose tissue, WGBS, whole genome bisulfite sequencing

## Abstract

DNA methylation regulates gene transcription and is involved in various physiological processes in mammals, including development and hematopoiesis. It is catalyzed by DNA methyltransferases including Dnmt1, Dnmt3a, and Dnmt3b. For Dnmt3b, its effects on transcription can result from its own DNA methylase activity, the recruitment of other Dnmts to mediate methylation, or transcription repression in a methylation-independent manner. Low-frequency mutations in human DNMT3B are found in hematologic malignancies including cutaneous T-cell lymphomas, hairy cell leukemia, and diffuse large B-cell lymphomas. Moreover, Dnmt3b is a tumor suppressor in oncogene-driven lymphoid and myeloid malignancies in mice. However, it is poorly understood how the different Dnmt3b activities contribute to these outcomes. We modulated Dnmt3b activity *in vivo* by generating *Dnmt3b*^*+/−*^ mice expressing one *wild-type* allele as well as *Dnmt3b*^*+/CI*^ and *Dnmt3b*^*CI/CI*^ mice where one or both alleles express catalytically inactive Dnmt3b^CI^. We show that 43% of *Dnmt3b*^*+/−*^ mice developed T-cell lymphomas, chronic lymphocytic leukemia, and myeloproliferation over 18 months, thus resembling phenotypes previously observed in *Dnmt3a*^*+/−*^ mice, possibly through regulation of shared target genes. Interestingly, *Dnmt3b*^*+/CI*^ and *Dnmt3b*^*CI/CI*^ mice survived postnatal development and were affected by B-cell rather than T-cell malignancies with decreased penetrance. Genome-wide hypomethylation, increased expression of oncogenes such as Jdp2, STAT1, and Trip13, and p53 downregulation were major events contributing to *Dnmt3b*^*+/−*^ lymphoma development. We conclude that Dnmt3b catalytic activity is critical to prevent B-cell transformation *in vivo*, whereas accessory and methylation-independent repressive functions are important to prevent T-cell transformation.

DNA methylation is an epigenetic modification that contributes to a regulation of gene transcription in mammalian cells. Its association with H3K9me3 and H3K27me3 histone modifications in gene promoters results in gene repression (1, 2). Methylation can also enhance transcription by promoting more efficient binding of transcription factors to their recognition sites (3, 4) and by restricting the activation of alternative promoters within gene bodies (3). DNA methylation is involved in regulation of normal development, differentiation, X chromosome inactivation, and genomic imprinting. It also participates in hematopoiesis, and its deregulation contributes to the pathogenesis of immune disorders, hematologic malignancies, and cancer (5, 6, 7, 8).

Four catalytically active enzymes (Dnmt1, Dnmt3a, Dnmt3b, and Dnmt3c) and one catalytically inactive cofactor (Dnmt3L) belong to the family of DNA methyltransferases (Dnmts) in mice. All Dnmts contribute to genome-wide methylation with Dnmt1 considered to be the main maintenance enzyme while Dnmt3a and Dnmt3b primarily involved in *de novo* activities (9, 10, 11). Dnmt3L lacks catalytic activity (CA) but is critical for *de novo* methylation by linking Dnmt3a and Dnmt3b to chromatin through unmethylated H3 lysine 4 (12). Dnmt3c suppresses transposon activity specifically in male germ cells (13). Dnmts also repress transcription independently of methylation, *e.g.*, through association with histone deacetylases (14, 15, 16). Another activity associated with Dnmt3L and Dnmt3b is accessory function (AF) that consists of the ability to recruit other Dnmts to genomic loci to catalyze methylation (12, 17, 18).

Dnmt3b participates in *de novo* and maintenance methylation, repression of germ line genes, X chromosome inactivation, and its knockout in mice is embryonically lethal (9, 10). In addition to methylation of various genomic elements, Dnmt3b also binds to the bodies of actively transcribed genes through the interaction of its PWWP domain with histone H3 trimethylated at lysine 36 and plays a role in their preferential methylation in embryonic stem cells (19). Human DNMT3B is causatively linked to the immunodeficiency-centromeric instability-facial anomalies (ICF) syndrome—a rare recessive autosomal disorder characterized by mild facial anomalies, cognitive impairment, recurrent infections, and lack of memory B-cells in peripheral blood (20, 21, 22). DNMT3B likely plays a role in pathogenesis of various hematologic malignancies as genetic alterations were identified in cutaneous T-cell lymphomas (CTCLs) and B-cell lymphomas (BCLs) (23, 24). Various other modes can affect activity of DNMT3B in cells. For instance, along with DNMT3A, DNMT3B belongs to the top 1% of underexpressed genes in human chronic lymphocytic leukemia (CLL) (25, 26, 27). Dnmt3b activity is also modulated by complexing with other proteins. For instance, a protein TCL1 that is overexpressed in a number of human T-cell malignancies, including mature leukemias, T-cell prolymphocytic leukemia, and B-cell malignancies, such as Burkitt’s lymphoma and CLL (28, 29), binds to Dnmt3a and Dnmt3b and inhibits their activities (30) raising a possibility that Dnm3b activity is functionally decreased in a large number of hematologic malignancies. Studies in mice showed that Dnmt3b is a tumor suppressor (TS) in an oncogene-induced hematologic malignancies including T- and B-cell lymphomas induced by MYC and acute myeloid leukemia induced by MLL-AF9 (26, 31, 32, 33, 34). Others reported oncogenic functions for Dnmt3b in MYC-induced T-cell acute lymphoblastic leukemia (T-ALL) likely due to its role in tumor maintenance (35).

Here we utilized genetic approaches to understand whether modulation of Dnmt3b activities *in vivo* by either decreasing gene dose using germline inactivation of one allele (*Dnmt3b*^*+/−*^) or decreasing or elimination of its CA through the use of recently generated catalytically inactive Dnmt3b^CI^ allele (*Dnmt3b*^*+/CI*^ and *Dnmt3b*^*CI/CI*^). Similarly to our previous study of *Dnmt3a*^*+/−*^ mice, our data identify Dnmt3b as a haploinsufficient tumor suppressor in T-cell lymphomas (TCL) and CLL (36). We further found that several hypomethylated and overexpressed oncogenes including Jdp2, Trip13, and Stat1 may contribute to TCL along with downregulation of p53. A development of TCL was suppressed in *Dnmt3b*^*+/CI*^ and *Dnmt3b*^*CI/CI*^ suggesting that CA is less important for their development. Rather, a reduction in AF may be responsible for TCL development. This is further supported by methylation data from MYC-induced T-cell lymphomas with modulated Dnmt3b activities in which AF seemed to substantially suppress loss of methylation. In contrast, the observed development of CLL and BCLs in these mice suggest the importance in prevention of B-cell transformation. Furthermore, we found that CA is largely dispensable for postnatal development with mice surviving but developing ICF-like syndrome. In summary, our data show that Dnmt3b is a multifunctional protein involved in control of genes important to prevent ICF and tumorigenesis.

## Results

### *Dnmt3b*^*+/−*^ mice develop T-cell lymphomas

**Figure 1:**
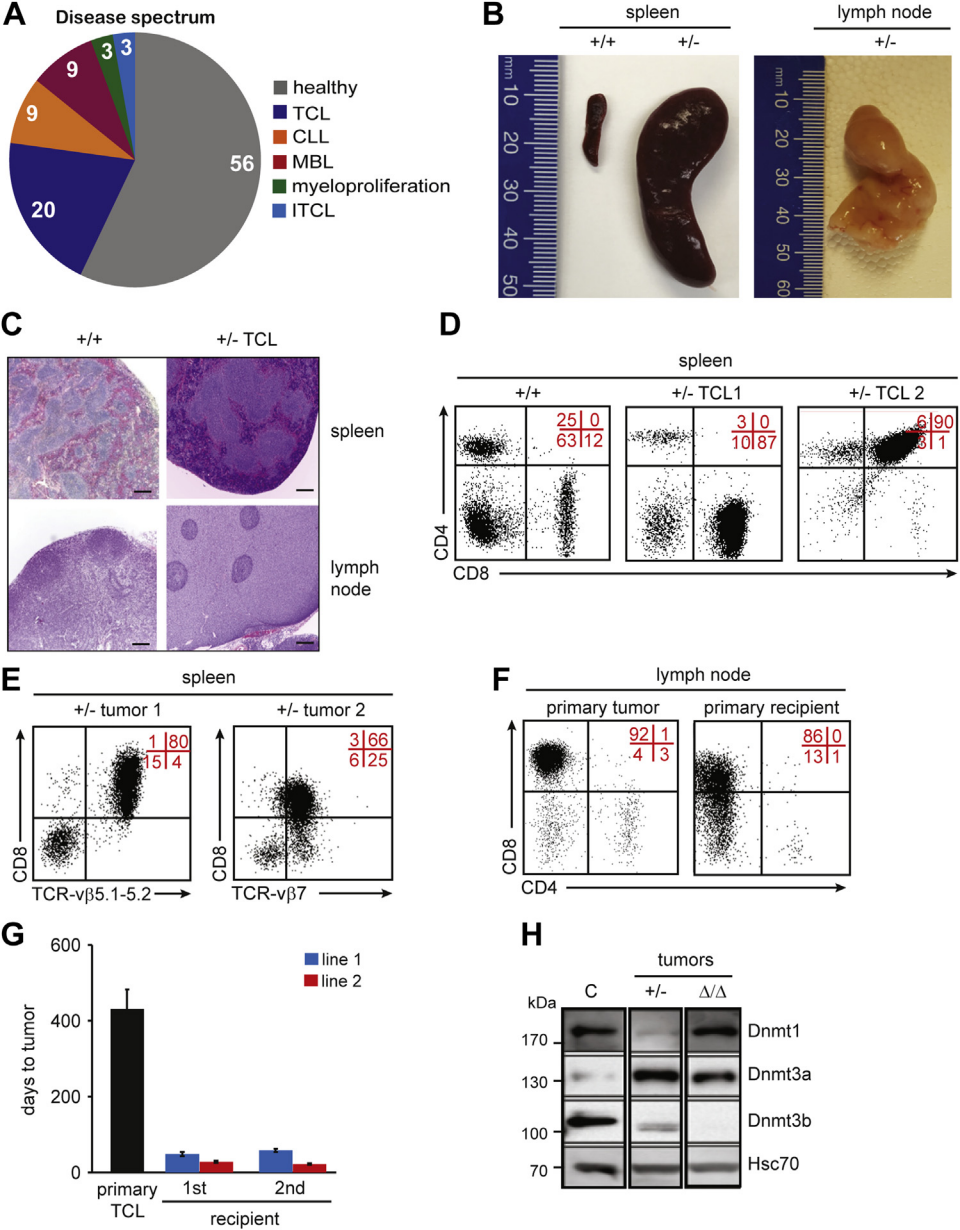
**Majority of *Dnmt3b***^***+/−***^**mice develop T-cell lymphomas.***A*, Disease spectrum observed in *Dnmt3b*^*+/−*^ mice (n = 35) determined by FACS. Values present percentage of mice diagnosed with indicated disease. *B*, Representative image of healthy spleen of *Dnmt3b*^*+/+*^ (+/+) mice and spleen and lymph node of terminally ill *Dnmt3b*^*+/−*^ (+/−) mice that developed TCL. *C*, Histological staining of spleen and lymph node of age-matched *Dnmt3b*^*+/+*^ control (*+/+*) and terminally ill *Dnmt3b*^*+/−*^ mouse (+/− TCL) (magnification 40x). *D*, Representative FACS diagrams of CD4 and CD8 expression in cells isolated from the spleen of healthy *Dnmt3b*^*+/+*^ mice (+/+) and terminally sick *Dnmt3b*^*+/−*^ mice that developed CD8+ (+/− TCL1) and CD4+CD8+ (+/− TCL2) lymphomas. Quadrant statistics are indicated in red here and in all figures. *E*, Representative FACS diagrams showing clonal TCR-vβ expression in *Dnmt3b*^*+/−*^ lymphomas (+/− tumor 1 and 2). *F*, Representative FACS diagrams of CD4 and CD8 expression in cells isolated from tumors that developed in terminally sick *Dnmt3b*^*+/−*^ mice (primary tumor) and sublethally irradiated FVB-recipient mice injected with primary lymphoma (primary recipient). *G*, Time to tumor development for primary mice (primary TCL), primary (first) and secondary (second) sublethally irradiated FVB-recipient mice serially transplanted with primary TCL isolated from the lymph nodes of terminally sick *Dnmt3b*^*+/−*^ mice. Data are presented as average time to tumor development. Two TCL lines are shown. *H*, Immunoblot analysis of Dnmt1, Dnmt3a, and Dnmt3b protein levels in healthy lymph node (C), *Dnmt3b*^*+/−*^ (+/−) and *Dnmt3b*^*Δ/Δ*^ (Δ/Δ) lymphomas. Hsc70 served as a loading control.

To evaluate the long-term consequences of Dnmt3b haploinsufficiency in mice, we generated and observed cohorts of *Dnmt3b*^*+/+*^ and *Dnmt3b*^*+/−*^ mice for 18 months. While control *Dnmt3b*^*+/+*^ and subset of *Dnmt3b*^*+/−*^ mice remained healthy, with no signs of deregulated hematopoiesis, 44% of *Dnmt3b*^*+/−*^ mice developed various hematologic malignancies including TCL, (20%) characterized by enlarged spleens and lymph nodes (Fig. 1, *A* and *B*, S1 and not shown). Histological analysis of spleens showed a near-complete effacement of the red pulp by massively expanded white pulp (Fig. 1*C*). Small-to medium-sized cells were present in both spleen and lymph nodes, and expressed markers of mature T-cells–CD3, CD5, TCRβ, and CD8 were negative for the expression of CD4, TCRγδ, NK-1.1, and CD16 (Fig. 1, *C* and *D* and not shown). In one case, we also observed a development of CD4+CD8+ immature TCL suggesting that decreased Dnmt3b levels may promote transformation of T-cells at earlier stages of the development (Figs. 1*D* and S1).

*Dnmt3b*^*+/−*^ lymphomas were likely monoclonal because most T-cells in the tumor uniformly expressed the same TCR receptor, *e.g.*, TCR-vβ 5.1 to 5.2 in tumor 1 or TCR-vβ 7 in tumor 2 (Fig. 1*E*). Cells showed full tumorigenic potential as transplantation of *Dnmt3a^+/-^* lymphoma cells induced peripheral T-cell lymphoma (PTCL) within 2 months in sublethally irradiated *wild-type* FVB recipient mice and while the same effect could be also observed upon subsequent transplantation of cells from tumors developed in recipient mice (Fig. 1, *F* and *G*). Tumors retained expression of Dnmt3b from *wild-type* allele suggesting that decreased levels of Dnmt3b, but not a complete inactivation, are sufficient to drive the disease development (Figs. 1*H* and S2). While Dnmt1 protein was downregulated, Dnmt3a level was increased in *Dnmt3b^+/-^* TCL possibly reflecting functional compensation for decreased Dnmt1/3b levels (Fig. 1*H*).

Altogether, these data demonstrate that the long-term Dnmt3b heterozygosity results in development of mostly mature CD8-positive TCLs similar to human PTCL–not otherwise specified (PTCL-NOS).

### A subset of *Dnmt3b*^*+/−*^ mice develop chronic lymphocytic leukemia

**Figure 2:**
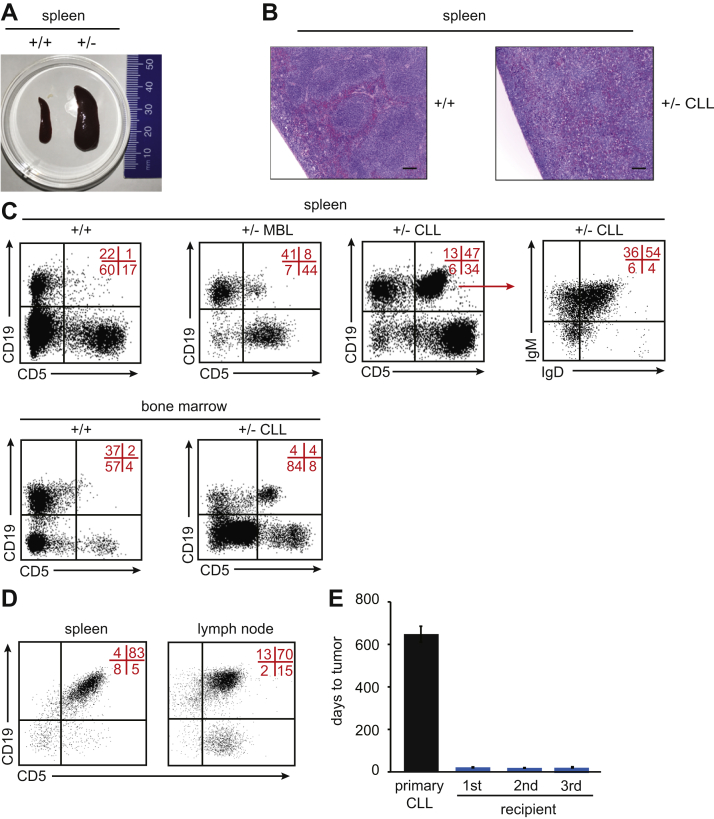
***Dnmt3b***^***+/−***^**mice also develop B-cell malignancies including MBL and CLL.***A*, Representative image of enlarged spleen of terminally ill *Dnmt3b*^*+/−*^ mice diagnosed with CLL (+/−) and healthy *wild-type* control (+/+). *B*, Histological staining of the spleen of terminally ill *Dnmt3b*^*+/−*^ mouse (+/− CLL) and age-matched *wild-type* control (*+/*+) (magnification 40x). *C*, Representative FACS diagrams of CD19 and CD5 expression in cells isolated from the spleen and bone marrow of healthy *Dnmt3b*^*+/+*^ mice (+/+) and terminally sick *Dnmt3b*^*+/−*^ mice that developed MBL (+/− MBL) and CLL (+/− CLL). Representative diagram of IgD and IgM expression on CD19+CD5+ cells isolated from +/- CLL spleen is also shown. *D*, Representative FACS diagrams of CD19 and CD5 expression in cells isolated from the spleen and lymph node of sublethally irradiated FVB-recipient mice injected with *Dnmt3b*^*+/−*^ CLL tumors as analyzed by FACS. *E*, Time to tumor development for primary mice (primary CLL), primary (first), secondary (second), and tertiary (third) sublethally irradiated FVB-recipient mice serially transplanted with primary CLL isolated from spleens of terminally sick *Dnmt3b*^*+/−*^ mice. Data are presented as mean ± SEM.

The second most common disease observed in *Dnmt3b*^*+/−*^ cohort was a CLL-like disease observed in three mice and characterized by splenomegaly and CD5+B220+CD19+ B-1a cells expansion of more than 20% in the blood, spleen, and bone marrow (Figs. 1*A*, 2, *A–C*, S1 and data not shown). Three mice showed signs of monoclonal B-cell lymphocytosis (MBL)—a less progressed form of CLL—in which the percentage of B-1a cells (CD5+B220+CD19+) in the blood is between 2% and 20%, with simultaneous expansion in the spleen and bone marrow (Fig. 2*C* and data not shown). Importantly, splenic cells from mice with either MBL or CLL were able to induce disease in recipient mice (Fig. 2, *D* and *E*), demonstrating that both populations contain true leukemic cells. Therefore, we refer to both conditions as CLL-like disease. In addition to PTCL and CLL, we also observed the development of a myeloproliferative disease (MPD) in one of the *Dnmt3b^+/−^* mice (Figs. 1*A*, S1 and data not shown). These mice showed expansion of Gr-1+CD11b+ myeloid cells in the blood, spleen, and bone marrow (data not shown). Thus, our data identify Dnmt3b as a haploinsufficient tumor suppressor gene in the prevention of TCLs and CLL that also may play a role in prevention of myeloid malignancies in mice.

### *Dnmt3b*^*CI/CI*^ mice survive postnatal development but develop ICF-like syndrome

Gene knockout of one allele in *Dnmt3b*^*+/−*^ mice decreases all functions of Dnmt3b protein including CA, AF, and methylation-independent repressive functions, thus precluding us to appreciate individual activities in *in vivo*. To determine the extent to which Dnmt3b CA plays a role in hematopoiesis and in its TS functions, we next utilized allele expressing catalytically inactive Dnmt3b (Dnmt3b^CI^) from endogenous locus. Dnmt3B^WT^ and Dnmt3b^CI^ protein levels are similar in mouse embryo and adult tissues demonstrating that an amino acid substitutions did not adversely affect regulation of Dnmt3b expression or protein stability as we reported previously (17).

**Figure 3:**
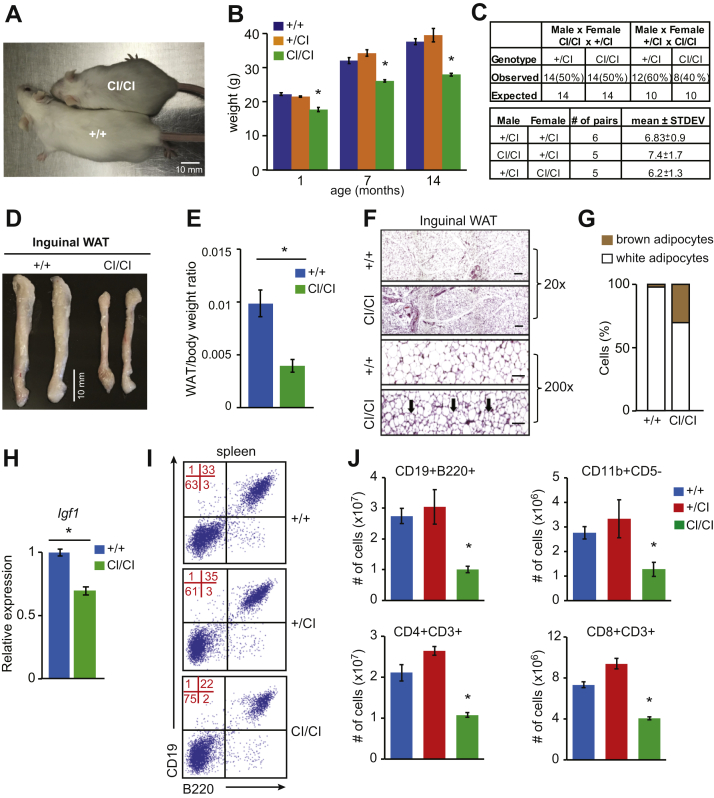
**Loss of Dnmt3b’s CA results in postnatal growth retardation and ICF-like phenotype.***A*, Representative image of 6-week-old *Dnmt3b*^*+/+*^ (+/+) and *Dnmt3b*^*CI/CI*^ (CI/CI) mice. *B*, The weights of *Dnmt3b*^*+/+*^ (+/+), *Dnmt3b*^*+/CI*^ (+/CI), and *Dnmt3b*^*CI/CI*^ (CI/CI) mice measured at 1, 7, and 14 months (n = 10, each). Data are presented as mean ± SEM, ∗*p* < 0.001 (by two-tailed Student’s *t*-test). *C*, Fertility rates of *Dnmt3b*^*CI/CI*^ (CI/CI) males and females showing observed and expected offspring genotypes, number of examined pairs of indicated genotypes, and average litter size are presented as mean ± STDEV. Differences are not statistically significant (χ^2^ test for genotype occurrence and two-tailed Student’s *t*-test for litter size). *D*, Representative image of inguinal white adipose tissue (WAT) isolated from *Dnmt3b*^*+/+*^ and *Dnmt3b*^*CI/CI*^ mice. *E*, Weights of inguinal WAT isolated from *Dnmt3b*^*+/+*^ (+/+) and *Dnmt3b*^*CI/CI*^ (CI/CI) mice normalized to body weight (n = 7, each). Data are presented as mean ± SEM. ∗*p* = 0.0094 by two-tailed Student’s *t*-test. *F*, Histological staining of inguinal WAT isolated from 8-week-old *Dnmt3b*^*+/+*^ and *Dnmt3b*^*CI/CI*^ mice at 20- and 200-fold magnifications. Arrows are pointing on brown-like adipocytes. *G*, Percentage of white and brown adipocytes in inguinal WAT of adult (8 weeks old) *Dnmt3b*^*+/+*^ (+/+) and *Dnmt3b*^*CI/CI*^ (CI/CI) mice. *H*, Real-time qRT-PCR analysis of *Igf1* levels in the liver of adult *Dnmt3b*^*+/+*^ (+/+) and *Dnmt3b*^*CI/CI*^ (CI/CI) mice (n = 3, each), normalized to *β-actin.* Data are presented as mean ± SEM, ∗*p* = 0.0041 by two-tailed Student’s *t*-test. *I*, Representative FACS diagrams showing CD19 and B220 expression in cells isolated from spleen of healthy (8 weeks old) *Dnmt3b*^*+/+*^ (+*/+*), *Dnmt3b*^*+/CI*^ (+/CI), and *Dnmt3b*^*CI/CI*^ (CI/CI) mice. Quadrant statistics are indicated in red. *J*, Absolute cell counts of B-cells (CD19 + B220+), myeloid cells (CD11b+CD5-), and mature T-cells (CD4+CD3+ and CD8+CD3+) in the spleen of *Dnmt3b*^*+/+*^ (+/+; n = 6), *Dnmt3b*^*+/CI*^ (+/CI; n = 2), and *Dnmt3b*^*CI/CI*^ (CI/CI; n = 6) mice as analyzed by FACS. Data are presented as mean ± SEM, ∗*p* < 0.05 by two-tailed Student’s *t*-test.

Unlike *Dnmt3b*^*-/-*^ mice, *Dnmt3b*^*CI/CI*^ mice survived embryogenesis, but the importance of Dnmt3b CA in adult mice remains unclear (17). As a prelude to assessing its role in hematopoiesis, we first analyzed postnatal development in cohorts of *Dnmt3b*^*+/CI*^ and *Dnmt3b*^*CI/CI*^ mice. *Dnmt3b*^*+/CI*^ mice were indistinguishable from their *wild-type* littermates and lived long lives. *Dnmt3b*^*CI/CI*^ mice were relatively normal, but their body weight was ∼20% lower than *wild-type* littermates at weaning, and this difference persisted throughout their postnatal lives (Fig. 3, *A* and *B*). Despite decreased size, both *Dnmt3b*^*CI/CI*^ males and females had normal life span and were fertile with litter size similar to controls (Fig. 3*C*). However, inguinal fat was significantly reduced with smaller white adipose tissue (WAT) deposits in *Dnmt3b*^*CI/CI*^ mice compared with controls (Fig. 3, *D* and *E*). Additionally, inguinal WAT showed presence of multilocular, brown-like adipocytes suggesting that Dnmt3b‘s CA plays a role in regulation of their development (Fig. 3, *F* and *G*). Previously, a development of brown adipocytes was linked to hypomethylation of genes responsible for mitochondrial respiratory chain and fatty acid oxidation (37). Because insulin-like growth factor 1 (*Igf1*) is implicated in a regulation of fat deposition and body size (38), we next analyzed its expression in liver—a major organ for its production - of *Dnmt3b*^*CI/CI*^ mice and found a small but significant reduction in transcript levels (Fig. 3*H*). Cerebral hyperplasia was also observed in *Dnmt3b*^*CI/CI*^ mice, but the molecular mechanism behind this remains unclear (Fig. S3*A*). *Dnmt3b*^*CI/CI*^ also had craniofacial defects including shortened nose, which is typical of ICF syndrome observed in humans and linked to DNMT3B mutations (Fig. S3, *B* and *C*). Consistently with the syndrome, hematopoiesis was less efficient with mildly decreased production of CD11b+ myeloid, CD3+ T-cells, and especially CD19+ B220+ B-cells observed in the spleens of *Dnmt3b^CI/CI^* mice (Figs. 3, *I* and *J* and S4).

Altogether, our data show that Dnmt3b’s CA is dispensable for postnatal development and fertility, but its absence results in a decreased body weight at least partially due to a fat deposition decrease likely associated with reduced Igf1 production in *Dnmt3b*^*CI/CI*^ mice. Loss of Dnmt3b’s CA induces phenotypes consistent with ICF syndrome in humans, in particular craniofacial defects and less efficient adult hematopoiesis.

### Dnmt3b’s CA is important for its tumor suppressor function in spontaneous lymphomagenesis

**Figure 4:**
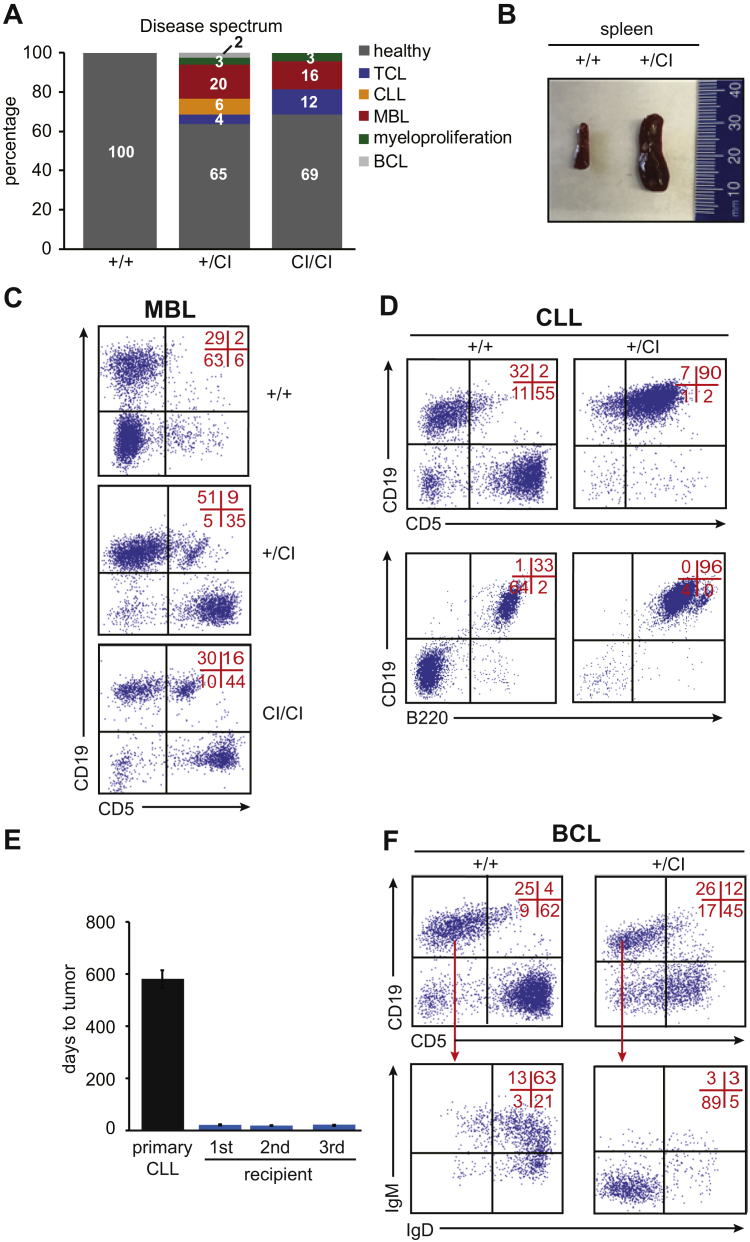
**Dnmt3b’s CA is important in the prevention of hematologic malignancies.***A*, Disease spectrum of *Dnmt3b*^*+/+*^ (+/+; n = 30) *Dnmt3b*^*+/CI*^ (+/CI; n = 67), and *Dnmt3b*^*CI/CI*^ (CI/CI; n = 32) mice as determined by FACS. *B*, Representative image of the enlarged spleen of terminally ill *Dnmt3b*^*+/CI*^ (+/CI) mouse that developed CLL and healthy spleen of age-matched *Dnmt3b*^*+/+*^ (+/+). *C*, Representative FACS diagram of CD5 and CD19 expression in cells isolated from the spleen of healthy *Dnmt3b*^*+/+*^ mice (*+/+*) and terminally sick *Dnmt3b*^*+/CI*^ (+/CI) and *Dnmt3b*^*CI/CI*^ (CI/CI) mice that developed MBL as analyzed by FACS. Quadrant statistics are indicated in red here and throughout whole figure. *D*, CD5, CD19, and B220 expression in cells isolated from the spleen of healthy *Dnmt3b*^*+/+*^ mice (+/+) and terminally sick *Dnmt3b*^*+/CI*^ (+/CI) mice that developed CLL as analyzed by FACS. *E*, Time to tumor development for primary mice (primary CLL), primary (first), secondary (second), and tertiary (third) sublethally irradiated FVB-recipient mice serially transplanted with cells isolated from the spleen of terminally sick *Dnmt3b*^*+/CI*^ mouse. Data are presented as mean ± SEM. *F*, CD5 and CD19 expression in cells isolated from the spleen of healthy *Dnmt3b*^*+/+*^ mice (+/+) and terminally sick *Dnmt3b*^*+/CI*^ (+/CI) mice that developed BCL as analyzed by FACS. Expression of IgM and IgD on CD19+CD5-cells is shown on the bottom panel.

To determine if Dnmt3b’s CA is important for its TS function in mouse spontaneous lymphomagenesis, we compared survival in cohorts of *Dnmt3b*^*+/+*^, *Dnmt3b*^*+/CI*^, and *Dnmt3b*^*CI/CI*^ mice. Similar to *Dnmt3b*^*+/−*^, *Dnmt3b*^*+/CI*^ mice developed a spectrum of hematologic malignancies including TCL, CLL, and myeloproliferation (Figs. 4*A* and S5). Compared with *Dnmt3b*^*+/−*^ mice with 44%, a combined disease penetrance in *Dnmt3b*^*+/CI*^ mice was reduced to 35% (23/67mice) with 65% of mice remaining healthy during the observational period of 18 months. Importantly, the disease spectrum was substantially changed with MBL and CLL being the most frequent diseases observed in these mice 20% (13/67 mice) and 6% (4/67 mice), respectively (Figs. 4*A* and S5). MBL and CLL were characterized by enlarged spleen and expansion of CD5+CD19 + B220+ cells in the spleen and blood (Fig. 4, *B–D* and not shown) similar to *Dnmt3b*^*+/−*^ mice. These cells induced disease in recipient mice upon serial transplantation into sublethally irradiated mice demonstrating their true leukemic nature (Fig. 4*E*). In addition, we also observed a development of immature BCL characterized by expansion of CD19+CD5-IgM-IgD-cells in the spleen (Fig. 4*F*).

TCL development was less frequent relative to *Dnmt3b*^*+/−*^ mice with only 4% (3/67) of mice developing CD8+ TCL (Figs. 4*A*, S5 and S6). As observed in *Dnmt3b*^*+/−*^ TCL, *Dnmt3b*^*+/CI*^ TCL tumors were serially transplantable into sublethally irradiated recipient mice demonstrating full tumorigenic transformation (not shown).

In total, 2/67 mice developed myeloproliferation characterized by expansion of CD11B-cells in the bone marrow (Fig. S7).

While the disease spectrum was similar in *Dnmt3b*^*CI/CI*^ mice, the penetrance of leukemia/lymphoma development was decreased with 31% of observed mice developing MBL/CLL and PTCL (Fig. 4*A*). In summary, these data suggest that CA contributes substantially to Dnmt3b’s TS function, in particular in B-cell malignancies.

### Methylomes of *Dnmt3b*^*+/−*^ and *Dnmt3a*^*Δ/Δ*^ lymphomas have both overlapping and unique features

**Figure 5:**
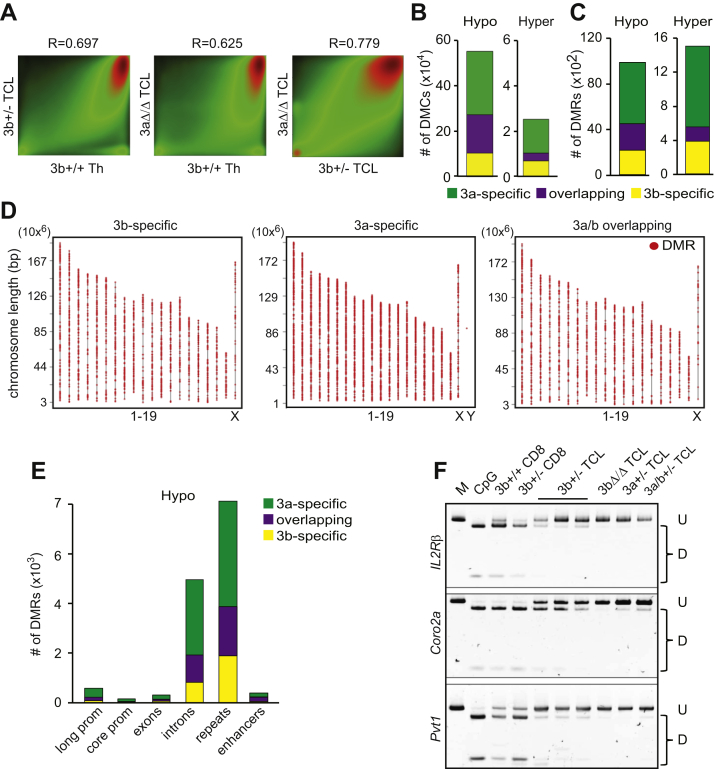
**Analysis of methylomes derived from mouse lymphomas by WGBS.***A*, Pairwise comparison of CpG methylation in *Dnmt3b*^*+/−*^ (*3b*+/- TCL), *Dnmt3a*^*Δ/Δ*^ (3aΔ/Δ TCL) lymphomas and healthy thymus (3b+/+ Th). The density of points increases from green to red. R-values represent Pearson’s correlation coefficients. *B*, Total numbers of hypomethylated and hypermethylated DMCs identified only in *Dnmt3b*^*+/−*^ lymphoma (3b-specific), only in *Dnmt3a*^*Δ/Δ*^ lymphoma (3a-specific), and in both (overlapping) relative to healthy thymus (meth. diff. ≥30% as analyzed by Metilene). *C*, Total numbers of hypomethylated and hypermethylated DMRs identified only in *Dnmt3b*^*+/−*^ lymphoma (3b-specific), only in *Dnmt3a*^*Δ/Δ*^ lymphoma (3a-specific), and in both (overlapping) relative to healthy thymus (DMR is defined as meth. diff. ≥30% in the same direction for three consecutive cytosines; p(MWU) <0.05 as analyzed by Metilene). *D*, Chromosomal distribution of DMRs identified only in *Dnmt3b*^*+/−*^ (3b-specific) lymphoma, only in *Dnmt3a*^*Δ/Δ*^ (3a-specific) lymphoma, and in both (3a/b overlapping) relative to healthy thymus. Only regions with sufficient coverage are shown. *E*, The numbers of DMRs associated with long promoters, core promoters, exons, introns, repeats, and enhancers identified only in *Dnmt3b*^*+/−*^ (3b-specific) lymphoma, only in *Dnmt3a*^*Δ/Δ*^ (3a-specific) lymphoma, and in both (overlapping) relative to healthy thymus. *F*, COBRA of putative Dnmt3b target gene promoters in *Dnmt3b*^*+/−*^ (3b +/- TCL), *Dnmt3b*^*Δ/Δ*^ (3bΔ/Δ TCL), *Dnmt3a*^*+/−*^ (3a +/- TCL), and *Dnmt3a*^*+/−*^*;Dnmt3b*^*+/−*^ (3a/b +/- TCL) lymphomas. *Dnmt3b*^*+/+*^ (3b+/+ CD8) and *Dnmt3b*^*+/−*^ (3b +/- CD8) healthy CD8 cells are shown. PCR products were digested with *BstU*I*, Taq*I *or Tai*I. Undigested fragments (U)—unmethylated DNA; digested fragments (D)—methylated DNA. PCR fragments generated from fully methylated genomic DNA that is undigested (M) or digested (CpG) are shown.

The development of TCL and CLL in *Dnmt3b*^*+/−*^ mice resembles phenotypes observed in *Dnmt3a*^*+/−*^ and *Dnmt3a*^*Δ/Δ*^ mice raising a possibility that similar molecular events drive lymphomagenesis in these settings (26, 36, 39). To assess this, we next analyzed methylomes of *Dnmt3b*^*+/−*^ and *Dnmt3a*^*Δ/Δ*^ TCL and publicly available normal thymus (ENCODE, Joe Ecker, Salk lab, ENCSR001MFH (40) by whole-genome bisulfite sequencing (WGBS). Initial analysis revealed that 6,300,000 CpG dinucleotides were covered at least 5x in all tested samples (Figs. S8 and S9). Out of these, 267,542 (4.2%) differentially methylated cytosines (DMCs; ≥30% change in methylation as analyzed by Metilene) showed ≥30% methylation reduction and 10,379 (0.16%) DMCs had ≥30% methylation increase in *Dnmt3b*^*+/−*^ PTCL cells when compared with normal thymus (Figs. 5, *A* and *B* and S9). In *Dnmt3a*^*Δ/Δ*^ PTCL, we identified 441,135 (7%) DMCs hypomethylated and 18,572 (0.29%) DMCs hypermethylated when compared with a normal control (Figs. 5*B* and S9). Interestingly, 166,047 (∼62%) hypomethylated DMCs were overlapping between *Dnmt3b*^*+/−*^ PTCL and *Dnmt3a*^*Δ/Δ*^ PTCL (Fig. 5*B*). Hypomethylation in both tumor groups was apparent for many genomic features including long and core promoters, exons, introns, CpG islands, and repeat elements (Figs. S10 and S11). Further analysis revealed 4433 hypomethylated and 548 hypermethylated differentially methylated regions (DMRs; ≥3 consecutive DMCs in the distance ≤50 bp with methylation change in the same direction ≥30%, p (MWU) <0.05 as analyzed by Metilene) in *Dnmt3b*^*+/−*^ PTCL (Fig. 5*C*). Of these, 2314 hypomethylated and 168 hypermethylated DMRs were identified also in *Dnmt3a*^*Δ/Δ*^ PTCL, and the overlap was observed across the genome (Fig. 5, *C–E*). Based on methylation patterns, *Dnmt3b*^*+/−*^ and *Dnmt3a*^*Δ/Δ*^ PTCL clustered closer and apart from normal thymus control (Fig. S12). This similarity is likely driven by decreased Dnmt3b and Dnmt3a levels. It does not seem to be just a consequence of T-cell transformation due to overlaps in hypomethylated DMRs being detected between *Dnmt3b*^*+/−*^ and *Dnmt3a*^*Δ/Δ*^ PTCL, especially in introns and repeats, even after hypomethylated DMRs present in *wild-type* MYC-induced TCLs expressing Dnmt3a and Dnmt3b were filtered out (Figs. S13 and S14). Such results suggest that Dnmt3b and Dnmt3a may cooperate in regulating target loci methylation. Data obtained from WGBS were further validated by combined bisulfite restriction analysis (COBRA), which showed DMRs substantially hypomethylated in promoters of *Il2Rβ*, *Coro2a*, and *Pvt1* genes in *Dnmt3a*^*+/−*^ TCL (Fig. 5*F*). Interestingly, these loci were even more hypomethylated in *Dnmt3b*^*Δ/Δ*^; *Dnmt3a*^*+/−*^, and *Dnmt3a*^*Δ/Δ*^ lymphomas we derived in our previous studies (26, 31, 39) further suggesting interplay between Dnmt3b and Dnmt3a in their methylation functions (Fig. 5*F*). Thus, our analysis of methylomes identifies both specific and overlapping events associated with decreased Dnmt3b and Dnmt3a levels in mouse lymphomas.

### Putative overlapping targets of Dnmt3b and Dnmt3a are associated with H3K4me1 and H3K27ac marks

**Figure 6:**
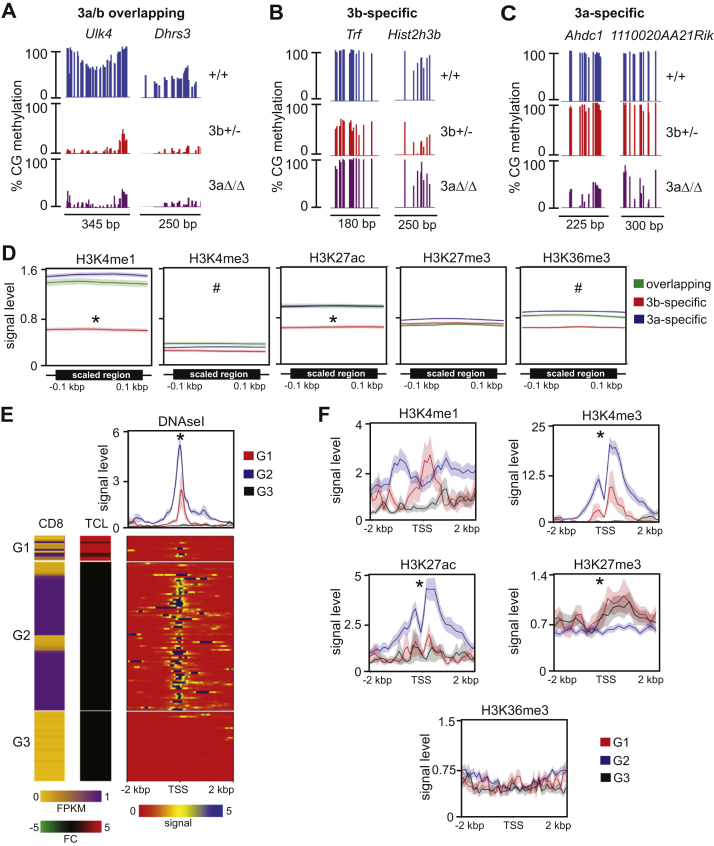
**Regions commonly hypomethylated in *Dnmt3b^+/-^* and *Dnmt3a*^*Δ*^^*/*^^*Δ*^ lymphomas are associated with H3K4me1 and H3K27ac.***A*, Visualization of single-CG methylation of representative DMRs commonly hypomethylated in *Dnmt3b*^*+/−*^ (3b+/−) and *Dnmt3a*^*Δ/Δ*^ (3aΔ/Δ) lymphomas in comparison with healthy thymus (+/+). *B*, Visualization of single-CG methylation of representative DMRs hypomethylated exclusively in *Dnmt3b*^*+/−*^ (3b+/−) and not *Dnmt3a*^*Δ/Δ*^ (3aΔ/Δ) lymphomas in comparison with healthy thymus (+/+). *C*, Visualization of single-CG methylation of representative DMRs hypomethylated exclusively in *Dnmt3a*^*Δ/Δ*^ (3aΔ/Δ) and not *Dnmt3b*^*+/−*^ (3b+/−) lymphomas in comparison with healthy thymus (+/+). *D*, Enrichment of DMRs hypomethylated exclusively in *Dnmt3b*^*+/−*^ (3b-specific), exclusively in *Dnmt3a*^*Δ/Δ*^ (3a-specific) or common for both lymphomas (overlapping) for chromatin marks in *wild-type* thymus as detected by analysis of ChIP-seq (ENCODE). Plots show profiles of mean histone signal levels ±SEM for DMRs scaled to same length and surrounding 100 bp unscaled regions. ∗ denotes statistically significant difference (*p* < 0.05 by two-tailed Student’s *t*-test) between 3b-specific and other groups of DMRs. # denotes all DMR groups significantly different from each other (*p* < 0.05 by two-tailed Student’s *t*-test). SEM values are presented as shading around mean value line. *E*, Chromatin accessibility and expression of genes with hypomethylated promoters in *Dnmt3b*^*+/−*^ lymphomas. Genes were assigned to three groups. G1 group with increased expression in lymphoma; G2 group without change in lymphoma samples and high FPKM values (>0.2) in *wild-type* CD8 T-cells; G3 group without change in lymphoma samples and low FPKM values (<0.2) in *wild-type* CD8 T-cells. *First panel* presents FPKM values in healthy *Dnmt3b*^*+/+*^ CD8+ T-cells (CD8), *second panel*—fold change expression in *Dnmt3b*^*+/−*^ (TCL) lymphomas relative to healthy *Dnmt3b*^*+/+*^ CD8+ T-cells, *third panel* presents results of DNAseI sensitivity assay (ENCODE) in the region from -2 to +2 kbp around TSS; plot above shows mean signal ±SEM. ∗*p* < 0.05 by two-tailed Student’s *t*-test. *F,* Histone marks occupancy signals ±SEM in the region from -2 to +2 kbp around TSS of hypomethylated and overexpressed genes in groups defined on figure (*E*); ∗*p* < 0.05 by two-tailed Student’s *t*-test. SEM values are presented as shading around mean value line.

Our analysis revealed 2314 DMRs hypomethylated in both *Dnmt3b*^*+/−*^ PTCL and *Dnmt3a*^*Δ/Δ*^ PTCL raising a possibility that methylation of these regions depends on activity of both Dnmt3b and Dnmt3a (Fig. 5*C*). We term these DMRs as *3a/b-overlapping*, such as *Ulk4* and *Dhrs3* loci (Fig. 6*A*). In total, 2119 DMRs were specifically hypomethylated only in *Dnmt3b*^*+/−*^ PTCL (*3b-specific*) (Fig. 5*C*), for instance, *Trf* locus and *Hist2h3b* promoter (Fig. 6*B*). In total, 5287 hypomethylated DMRs were detected specifically only in *Dnmt3a*^*Δ/Δ*^ PTCL (*3a-specific*), *e.g.*, *Ahdc1* and *1110020A21Rik* loci (Figs. 5*C* and 6C). To analyze association between histone modifications and these three types of DMRs, we next utilized available ChIP-seq data of normal *Dnmt3b*^*+/+*^ thymocytes (ENCODE-ENCSR325LOF) (40). We found that *3a-specific* and *3a/3b-overlapping* DMRs are marked with H3K4me1, H3K27ac, and H3K36me3 on a genome-wide level in normal thymus (Fig. 6*D*). This signature was present in various genomic regions including enhancers, promoters, introns, exons, and repetitive elements (Fig. S15). In contrast, we did not observe any specific chromatin signature genome-wide or in individual genomic elements in *3b-specific* DMRs in normal thymus (Figs. 6*D* and S15). To determine the effects of DNA methylation, we next analyzed association between promoter hypomethylation and gene expression in *Dnmt3b*^*+/−*^ PTCL and found that 13/126 *3b-specific* hypomethylated promoters (10%) had increased expression relative to thymic control (termed *G1 group*; FC ≥ 2, *p* < 0.05; Fig. 6*E* and Supporting Information 1). Genes unchanged in expression upon promoter demethylation belonged to two distinct groups characterized by either high (*G2 group*; FPKM>0.2; n = 77) or low (*G3 group*; FPKM<0.2; n = 36) FPKM values in normal thymus (Fig. 6*E* and Supporting Information 1). *G1* genes were expressed in thymus, had open chromatin around transcription start site (TSS) and enrichment in both activating H3K4me3 and H3K27me3 repressive histone marks (Fig. 6, *E* and *F* and Supporting Information 1). *G2* genes were already expressed in control thymus, had open chromatin and only activating histone marks around TSS (Fig. 6, *E* and *F* and Supporting Information 1). *G3* genes were not expressed in thymus, had closed chromatin and enrichment in repressive H3K27me3 mark around TSS (Fig. 6, *E* and *F* and Supporting Information 1). Thus, DNA methylation appears to provide additional layer of regulation for genes with histone mark signature H3K4me3+H3K27me3+ that is characteristic for bivalent promoters, whereas it seems to be less important for genes with repressive H3K27me3+ mark only or for the actively transcribed genes.

### Tumor suppressor p53 and putative oncogenes are deregulated in *Dnmt3b*^*+/−*^ lymphomas

To understand further molecular effects of monoallelic Dnmt3b loss on TCL development and the extent to which it resembles Dnmt3a-deficient lymphomas, we next analyzed gene expression in *Dnmt3b*^*+/−*^ and *Dnmt3a*^*Δ/Δ*^ TCLs by RNA-seq.

**Figure 7:**
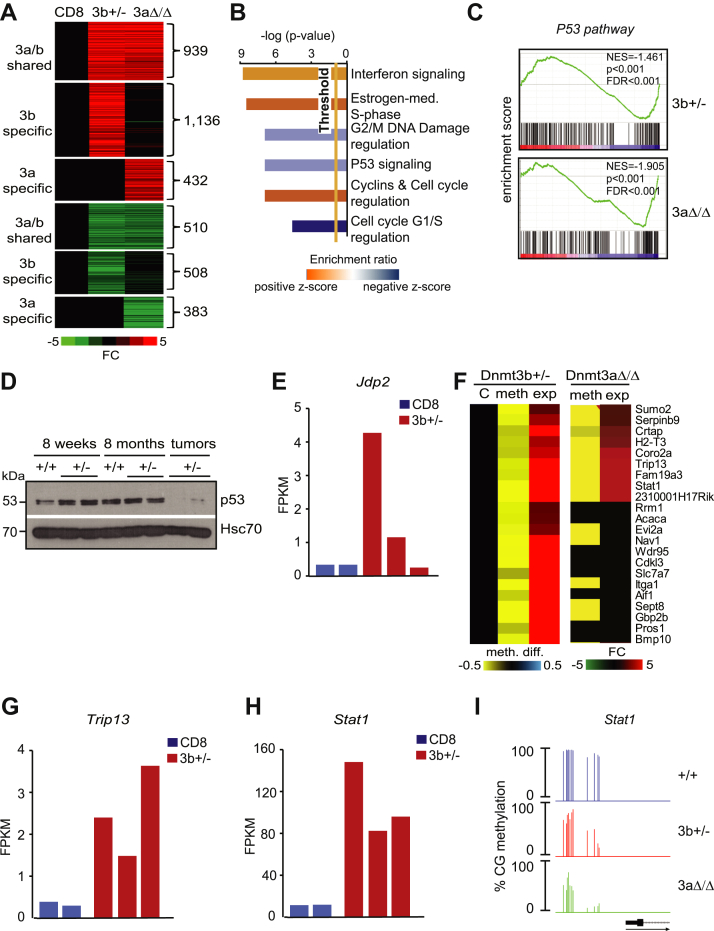
**P53 pathway is commonly deregulated in *Dnmt3b* *^+/-^* and *Dnmt3a***^***Δ/Δ***^**TCL.***A*, Heat map showing fold change expression values of subset of differentially expressed genes (FC ≥ 3, *p* < 0.05 by DESeq) in *Dnmt3b*^*+/−*^ (3b+/−; n = 3) and *Dnmt3a*^*Δ/Δ*^ (3aΔ/Δ; n = 3) lymphomas when compared with control CD8+ T-cells (CD8; n = 2). *B*, Ingenuity pathway analysis of genes commonly deregulated (FC ≥ 3, *p* < 0.05 by DESeq) in *Dnmt3b^+/-^* and *Dnmt3a*^*Δ/Δ*^ lymphomas when compared with control CD8+ T-cells. The top canonical pathways are displayed (*p* < 0.05 by right-tailed Fisher’s exact test). *C*, GSEA using RNA-seq data shows negative enrichment in *P53 pathway* in Dnmt3b^*+/−*^ (3b+/−) and *Dnmt3a*^*Δ/Δ*^ (3aΔ/Δ) lymphomas when compared with control CD8+ T-cells. Normalized enrichment scores (NES), false discovery rate (FDR), and *p*-values are shown. *D*, Immunoblot analysis of p53 protein levels in healthy 8 weeks and 8-month-old *wild-type* (+/+) and *Dnmt3b*^*+/−*^ (+/−) mice and *Dnmt3b*^*+/−*^ lymphomas (tumors). Hsc70 served as a loading control. *E*, *Jdp2* expression by RNA-seq in control CD8+ T-cells (CD8; n = 2) and *Dnmt3b*^*+/−*^ (3b*+/−*; n = 3) lymphomas. *F*, Heat maps showing overlap between promoter hypomethylation (meth. diff. ≥30%; p(MWU) <0.05) and gene overexpression (FC ≥ 2, *p* < 0.05 by DESeq) in *Dnmt3b*^*+/−*^ (n = 3) and *Dnmt3a*^*Δ/Δ*^ (n = 3) lymphomas when compared with control CD8+ T-cells (C; n = 2). *G*, *Trip13* expression by RNA-seq in control CD8+ T-cells (CD8; n = 2) and *Dnmt3b*^*+/−*^ (3b+/−; n = 3) lymphomas. *H*, *Stat1* expression by RNA-seq in control CD8+ T-cells (CD8; n = 2) and *Dnmt3b*^*+/−*^ (3b+/−; n = 3) lymphomas. *I*, Percentage of methylation at individual CpGs of *Stat1* promoter locus in *Dnmt3b*^*+/−*^ (3b+/−) and *Dnmt3a*^*Δ/Δ*^ (3aΔ/Δ) lymphomas and in healthy thymus (*+/+*).

In total, 2076 upregulated and 1018 downregulated genes (FC ≥ 2, *p* < 0.05) were identified in *Dnmt3b*^*+/−*^ TCL relative to normal control (Fig. 7*A* and Supporting Information 2). Out of these, 939 upregulated and 510 downregulated events were shared between both types of lymphomas with remaining changes specific to Dnmt3b and additional specific ones found in *Dnmt3a*^*Δ/Δ*^ TCL (Fig. 7*A* and Supporting Information 2). Thus, ∼40% deregulated genes are shared between both groups suggesting that these lymphomas may have common drivers of disease development (Fig. 7*A* and Supporting Information 2). Based on their expression profiles, *Dnmt3b*^*+/−*^ and *Dnmt3a*^*Δ/Δ*^ TCL clustered together and apart from normal CD8+ T-cells (Fig. S16).

Ingenuity pathway analysis (IPA) using *3a/b shared* gene expression changes (Fig. 7*A* and Supporting Information 2) revealed upregulated *interferon signaling, estrogen-mediated S-phase entry, cyclins and cell cycle regulation* and suppression of G2/M DNA damage regulation, *p53 signaling*, and *cell cycle G1/S regulation* (Fig. 7*B*). P53 pathway downregulation was also identified by gene set enrichment analysis (GSEA) (Fig. 7*C*). The importance of p53 in prevention of T-cell transformation in mice (41) prompted us to examine the p53 protein during lymphomagenesis in *Dnmt3b*^*+/−*^ mice. P53 levels in thymi of symptomless *Dnmt3b*^*+/−*^ mice at different ages were unaffected relative to control *Dnmt3b*^*+/+*^ (Fig. 7*D*). In contrast, p53 was downregulated in *Dnmt3b*^*+/−*^ TCL consistently with downregulation detected by IPA and GSEA highlighting its likely involvement in tumorigenesis (Fig. 7*D*). Trp53 transcript levels were not downregulated in lymphomas (data not shown) suggesting rather a proteolytic degradation. A major negative regulator of p53—Mdm2—was not upregulated indicating no involvement in its proteolytic degradation (Fig. S17).

We have recently linked a negative regulator of *Trp53*—Jun dimerization protein 2 (Jdp2) as a contributor to p53 downregulation in *Dnmt3a*^*Δ/Δ*^ PTCLs (39, 42). Interestingly, analysis of RNAseq data identified upregulation of Jdp2 suggesting that its increased expression may contribute to p53 downregulation also in *Dnmt3b*^*+/−*^ PTCL (Fig. 7*E*). Because Dnmt3b haploinsufficiency resulted in TCL with substantially deregulated methylomes, we next hypothesized that gene hypomethylation accompanied by increased expression may also contribute to lymphomagenesis (Fig. 7*F* and Supporting Information 3). We identified 22 genes that become overexpressed upon promoter hypomethylation in *Dnmt3b*^*+/−*^ PTCL. Out of these, nine genes were also hypomethylated and overexpressed in *Dnmt3a*^*Δ/Δ*^ PTCLs suggesting that these are shared targets of Dnmt3b and Dnmt3a. This signature contained several genes with possible oncogenic activities in TCL including *Trip13* and *Stat1* (43, 44). Both were strongly upregulated and hypomethylated in all *Dnmt3b*^*+/−*^ TCL (Fig. 7, *G–I* and S18).

Stat1 activation was also detected by Panther and Reactome pathway analysis using hypomethylated and overexpressed genes through deregulation of *IL9* and *IL21* signaling (Fig. S19). Given the well-established role of Stat1 as an oncogene in T-cells, our data strongly suggest that this upregulation contributes to lymphomagenesis (44).

Altogether, these data suggest that downregulation of p53 and upregulation of Jdp2 and Stat1 are likely relevant in initiation/progression of lymphomagenesis.

### Dnmt3b’s CA and AF contribute to generation of TCLs methylomes

**Figure 8:**
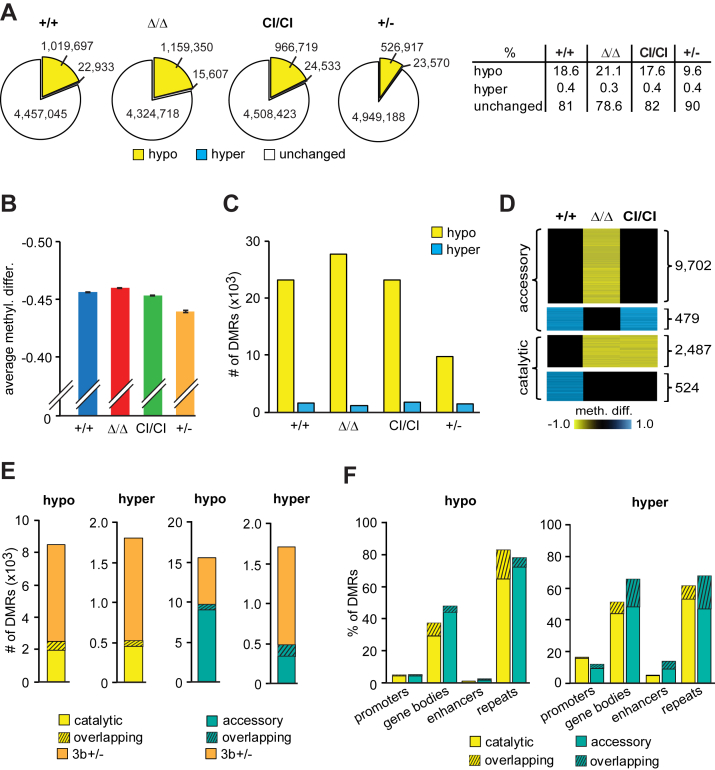
**A comparison of methylomes derived from *Dnmt3b***^***Δ/Δ***^***, Dnmt3b***^***CI/CI***^**, and *Dnmt3b***^***+/−***^**T-cell lymphomas.***A*, *Left.* Total numbers of DMCs (meth. diff. ≥30% as based on average of two samples; covered ≥5x) in *MYC*;*Dnmt3b*^*+/+*^ (+/+), *MYC;Dnmt3b*^*Δ/Δ*^ (Δ/Δ), *MYC;Dnmt3b*^*CI/CI*^ (CI/CI), and *Dnmt3b*^*+/−*^ (+/−) TCLs when compared with healthy thymus. Data were obtained by WGBS and analysed using Metilene. *Right.* The percentage of hypo- and hypermethylated CpGs identified in lymphomas of indicated genotypes (n = 2 each). *B*, Comparison of average differences between methylation levels of corresponding CpGs of healthy thymus and *MYC*;*Dnmt3b*^*+/+*^, *MYC;Dnmt3b*^*Δ/Δ*^, *MYC;Dnmt3b*^*CI/CI*^, and *Dnmt3b*^*+/−*^ lymphomas (n = 2 each). Data are presented as mean ±SEM. *C*, Total number of hypo- and hypermethylated DMRs (≥3 consecutive DMCs in the distance ≤50 bp with methylation change in the same direction ≥30%, p (MWU) <0.05 as analyzed by Metilene based on average of two samples) identified in *MYC;Dnmt3b*^*+/+*^*, MYC;Dnmt3b*^*Δ/Δ*^*, MYC;Dnmt3b*^*CI/CI*^, and *Dnmt3b*^*+/−*^ lymphomas relative to normal thymocytes (n = 2 each). *D*, Heat map showing DMRs identified in *MYC;Dnmt3b*^*+/+*^*, MYC;Dnmt3b*^*Δ/Δ*^, *and MYC;Dnmt3b*^*CI/CI*^ lymphomas relative to healthy thymus (n = 2 each) as analyzed by Metilene. Putative targets of Dnmt3b’s catalytic and accessory functions are indicated. *E*, Number of hypomethylated and hypermethylated DMRs identified in *Dnmt3b*^*+/−*^ lymphoma and their overlap with putative targets of Dnmt3b’s catalytic and accessory activity identified in (*D*). Number of DMRs identified in *Dnmt3b*^*+/−*^ lymphomas and MYC-induced lymphomas analyzed in (*D*) part is indicated by striped bars and labeled *overlapping*. *F*, The relative distribution of Dnmt3b’s accessory and catalytic targets described in (*D*) among indicated genomic elements presented as percentage. Percentage of DMRs identified also in *Dnmt3b*^*+/−*^ lymphomas is indicated by striped bars and labeled *overlapping*.

Because TCL development observed in *Dnmt3b*^*+/−*^ mice was suppressed in *Dnmt3b*^*CI/CI*^, we were unable to analyze molecular effects of Dnmt3b’s CA on the tumor methylome. We, therefore, sought to use a model allowing us to compare TCLs methylomes obtained from full Dnmt3b inactivation to those without Dnmt3b’s CA. Therefore, we used *EμSRa-tTA;Teto-MYC* model in which MYC transgene overexpression results in a development of immature TCLs (45). We have previously shown that a conditional loss of Dnmt3b in *EμSRa-tTA;Teto-MYC;Teto-Cre;Rosa26LOXP*^*EGFP*^*;Dnmt3b*^*fl/fl*^ mice (termed *MYC;Dnmt3b*^*Δ/Δ*^) resulted in accelerated TCL (31). Here we generated *EμSRa-tTA;Teto-MYC;Dnmt3b*^*+/+*^ (termed *MYC;Dnmt3b*^*+/+*^) and *EμSRa-tTA;Teto-MYC;Dnmt3b*^*CI/CI*^ (termed *MYC;Dnmt3b*^*CI/CI*^) mice and harvested CD4+CD8+ TCLs that developed in these mice (Fig. S20). Next, we used WGBS to determine methylation patterns in *MYC;Dnmt3b*^*+/+*^, *MYC;Dnmt3b*^*CI/CI*^, and *MYC;Dnmt3b*^*Δ/Δ*^ lymphomas (31) (two samples per genetic group). WGBS data were next compared with methylation data derived from *Dnmt3b*^*+/−*^ PTCL (Fig. 5) and normal thymus (ENCSR001MFH) (40) (Figs. S8 and S21). This comparison yielded methylation readouts of 5,499,675 CpGs covered ≥5x in all samples. All lymphomas showed reduction in methylation relative to thymocytes with the highest percentage of hypomethylated DMCs (≥30% change in methylation as analyzed by Metilene based on average of two samples) present in *MYC;Dnmt3b*^*Δ/Δ*^ lymphomas (21.1%) and the lowest in *Dnmt3b*^*+/−*^ PTCL (9.6%) (Fig. 8, *A* and *B*). Gains in methylation were also seen affecting approximately 0.4% of CpGs in all lymphomas (Fig. 8*A*). DMR analysis (DMR defined as ≥3 consecutive DMCs in the distance ≤50 bp with methylation change in the same direction ≥30%, p (MWU) <0.05 as analyzed by Metilene based on average of two samples) revealed that *MYC;Dnmt3b*^*Δ/Δ*^ TCLs had the highest number of hypomethylated and the lowest number of hypermethylated DMRs relative to normal thymocytes (∼27,000, and ∼1,100, respectively; Fig. 8*C*). Total number of hypomethylated DMRs was similar between *MYC;Dnmt3b*^*+/+*^ and *MYC;Dnmt3b*^*CI/CI*^ lymphomas (∼23,000) suggesting that Dnmt3b^CI^ protein restored at least some Dnmt3b functions lost in *MYC;Dnmt3b*^*Δ/Δ*^ lymphomas (Fig. 8*C*). The lowest number of hypomethylated DMRs was observed in *Dnmt3b*^*+/−*^ PTCL (Fig. 8*C*). A number of hypermethylated DMRs was similar—∼2000—across all tumor groups.

To begin to uncover putative targets of Dnmt3b’s CA or AF, we first filtered out ∼3500 mostly hypomethylated DMRs that were observed in all MTCLs presumably as a result of transformation and thus not linked to Dnmt3b (data not shown). As many as 9702 hypomethylated DMRs were present only in *MYC;Dnmt3b*^*Δ/Δ*^ but not in *MYC;Dnmt3b*^*CI/CI*^ or *MYC;Dnmt3b*^*+/+*^ suggesting that these DMRs may represent putative targets of Dnmt3b’s AF (Fig. 8*D*). Thus, Dnmt3b^CI^ may have contributed to retaining of methylation of ∼80% of regions (9702/12,189) that are hypomethylated in *MYC;Dnmt3b*^*Δ/Δ*^ TCL. In contrast, 2487 DMRs may represent putative targets of Dnmt3b’s CA because they were detected in both *MYC;Dnmt3b*^*Δ/Δ*^ and *MYC;Dnmt3b*^*CI/CI*^ MTCLs (Fig. 8*D*). Further analysis revealed 1335 hypermethylated DMRs out of which 332 were present in *MYC;Dnmt3b*^*+/+*^, *MYC;Dnmt3b*^*Δ/Δ*^, and *MYC;Dnmt3b*^*CI/CI*^ TCL and therefore likely not dependent on Dnmt3b activities (Fig. 8*D* and data not shown), while 479 might be putative targets of Dnmt3b’s AF because they were detected in *MYC;Dnmt3b*^*CI/CI*^ but not in *MYC;Dnmt3b*^*Δ/Δ*^ TCL. In addition, 524 DMRs might be putative targets of Dnmt3b’s CA because they are hypermethylated in *MYC;Dnmt3b*^*+/+*^ lymphomas but not in *MYC;Dnmt3b*^*Δ/Δ*^ or *MYC;Dnmt3b*^*CI/CI*^ MTCLs that lack CA (Fig. 8*D*).

A small subset of a putative targets of Dnmt3b’s AF and CA in MTCL lymphomas (both hypo- and hypermethylated) was also observed in *Dnmt3b*^*+/−*^ PTCL suggesting that these loci might be in particular sensitive to decreased levels of Dnmt3b (Fig. 8*E*).

Dnmt3b’s targets in TCLs were distributed relatively equally across various genomic elements including promoters, gene bodies, enhancers, and repeats, but AF appears to be more involved in preventing hypomethylation in gene bodies (Figs. 8*F* and S22). In contrast, a relative contribution of CA to hypermethylated DMRs seems to be increased in promoters possibly indicating a direct role in *de novo* methylation (Figs. 8*F* and S22).

Altogether, our data indicate that Dnmt3b’s AF plays a major role in lymphomagenesis substantially suppressing loss of methylation observed in tumors without Dnmt3b perhaps because it may be involved in maintenance methylation. Furthermore, both CA and AF may be important in a generation of hypermethylated DMRs possibly indicating a role in *de novo* methylation. However, such conclusions have to be further confirmed in more functional studies in the future.

## Discussion

Here we show that decreasing Dnmt3b’s activities *in vivo* in *Dnmt3b*^*+/−*^, *Dnmt3b*^*+/CI*^, and *Dnmt3b*^*CI/CI*^ mice results in development of various hematologic malignancies, mostly TCL and CLL, highlighting its tumor suppressor function in spontaneous lymphomagenesis.

Several interesting results were obtained from phenotypic observations in mice. For instance, mice expressing Dnmt3b^CI^ developed hematologic malignancies with decreased incidence relative to *Dnmt3b*^*+/−*^ mice. This could be due to a different magnitude of DNA methylomes deregulation in *Dnmt3b*^*+/CI*^ and *Dnmt3b*^*CI/CI*^ lymphomas, which is likely less pronounced than in *Dnmt3b*^*+/−*^ lymphomas. This hypothesis stems from the fact that Dnmt3b^CI^ protein retains AF consisting of the ability to recruit other DNMTs, which provide their CA for cytosine methylation thereby stabilizing methylome. Such AF rescued 95% of DNA methylation during mouse embryogenesis in *Dnmt3b*^*CI/CI*^ relative to *Dnmt3b*^*-/-*^ embryos at E11.5 (17). Alternatively, accumulation of other pathogenic events such as deregulated gene expression or genetic alterations is favored in *Dnmt3b*^*+/−*^ mice. Regardless of the reason for a decreased disease penetrance in *Dnmt3b*^*+/CI*^ and *Dnmt3b*^*CI/CI*^ mice, our data clearly demonstrate that even monoallelic inactivation of Dnmt3b’s CA activity promotes malignant hematopoiesis highlighting its crucial role—and in a broader sense the role of reduced cytosine methylation—in the prevention of hematopoietic cell transformation.

Another interesting finding is that *Dnmt3b*^*CI/CI*^ mice had decreased tumor incidence relative to *Dnmt3b*^*+/CI*^ (from 35% to 31%) and changed disease spectrum, which is surprising as full CA inactivation would be predicted to promote, rather than suppress, tumorigenesis. However, this could be caused by hematopoietic cell reduction resulting in a smaller cellular pool available for transformation or changes in microenvironment due to the presence of homozygous germline Dnmt3b inactivation of CA in *Dnmt3b*^*CI/CI*^ mice. Interestingly, data obtained here on mice with different Dnmt3b activities are similar to those observed in mice with varying degrees of Dnmt3a inactivation. A conditional inactivation of Dnmt3a in hematopoietic cells results in 100% disease penetrance predominantly CLL with few cases of PTCL (26, 39). Similarly, *Dnmt3a^+/-^* mice harboring a conventional knockout allele of Dnmt3a in FVB mouse strain develop either MBL/CLL or PTCL or MPD with 67% penetrance over 16 months (39). *Dnmt3a*^*+/−*^ mice on BL6 background developed various myeloid conditions including myeloproliferative disease and myeloid leukemia with 56% penetrance over the course of 2 years (46). Taken all these data together, a decrease in Dnmt3b favors development of T-cell malignancies, while the CA seems less important due to the disease being less frequently observed in *Dnmt3b*^*+/CI*^ and *Dnmt3b*^*CI/CI*^ compared with *Dnmt3b*^*+/−*^mice. In contrast, loss of only Dnmt3b’s CA but not AF in *Dnmt3b*^*+/CI*^ mice favors development of B-cell malignancies in particular CLL. Similarities in phenotypic consequences of long-term heterozygosity in mice suggest that Dnmt3a and Dnmt3b may coordinately control genes contributing to transformation. We identified several molecular events that are similar between *Dnmt3b*^*+/−*^ and *Dnmt3a^Δ^^/^^Δ^* TCL. One important molecular event observed in *Dnmt3b*^*+/−*^ TCL is downregulation of tumor suppressor p53 on protein level, which we also detected in *Dnmt3a^Δ^^/^^Δ^* TCL previously (39). Decreased p53 contributes to T-cell transformation as *Trp53^-/-^* mice are highly susceptible to spontaneous development of thymic lymphomas (41). Like in *Dnmt3a^Δ^^/^^Δ^* TCL, we also found that Jdp2–a component of the AP-1 transcription factor complex that represses transactivation-mediated by the Jun family of proteins—is upregulated in a majority of *Dnmt3b*^*+/−*^ TCL. Jdp2 is an oncogene that collaborates with the loss of p27^kip1^ cyclin-dependent inhibitor to induce lymphomas (47) and also negatively regulates Trp53 promoting T-cell leukemia development in mice (42). Jdp2 upregulation in *Dnmt3a^Δ^^/^^Δ^* PTCLs was associated with decreased p53 and causatively contributed to disease progression (39). Similarly, Jdp2 is upregulated in majority of *Dnmt3b*^*+/−*^ lymphomas while p53 is downregulated suggesting that similar to *Dnmt3a^Δ^^/^^Δ^* PTCLs, both events may be linked or contribute independently to lymphomagenesis. Additional putative oncogenic event shared between *Dnmt3b^+/-^* and *Dnmt3a^Δ^^/^^Δ^* PTCL was promoter hypomethylation accompanied by gene upregulation. Genes with oncogenic functions, such as Stat1 and Trip13, were upregulated possibly contributing to disease initiation/progression (43, 44, 48). Thus, downregulation of p53 along with activation of oncogenes likely represents important events promoting lymphomagenesis in both *Dnmt3a^+/-^* and *Dnmt3a^Δ^^/^^Δ^* mice.

Analysis of *Dnmt3b*^*+/−*^ and *Dnmt3a*^*Δ/Δ*^ TCL methylation and its association with histone marks also revealed that both *Dnmt3a-specific* and *Dnmt3a/b-overlapping* hypomethylated DMRs are associated with H3K4me1, H3K27ac, and H3K36me3 in normal thymus on a genome-wide level. In contrast, no specific chromatin modification signature on genome-wide level in *Dnmt3b specific* DMRs in normal thymus was found.

These data are in line with our previous study suggesting that in embryos, the accessory activity of Dnmt3b is promoted by the presence of activating marks such as H3K36me3 (17). The fact that we observe such association in thymus for *Dnmt3a/b-overlapping* but not *Dnmt3b-specific* DMRs suggests that Dnmt3a may be the enzyme providing CA, while Dnmt3b supplies AF function for the methylation of these loci. At the same time, CA of Dnmt3b was associated with the presence of repressive H3K27me3 in embryos, but not on genome-wide level in thymus. However, a group of 13 genes (*G1 group*), whose promoters were marked by both activating H3K4me3 and H3K27me3 repressive histone marks in normal thymocytes, was the only group of genes responding to promoter methylation loss by gene upregulation specifically in *Dnmt3b*^*+/−*^ tumors. By the presence of H3K4me3 and H3K27me3 marks, G1 group resembles genes with bivalent promoters. Given previous reports that promoters that are marked with H3K27me3 in embryonic stem cells are more likely to gain DNA methylation during differentiation, and that DNA methylation promotes acquisition of H3K27me3 on bivalent promoters (2, 49), our data suggest that Dnmt3b-dependent DNA methylation contributes to regulation of expression at least in a subset of genes containing bivalent promoters.

Additional interesting findings came from a generation and methylome analysis of MYC-induced TCLs that either had full Dnmt3b inactivation *MYC;Dnmt3b*^*Δ/Δ*^ or just inactivation of its CA while retaining other functions including accessory (*MYC;Dnmt3b*^*CI/CI*^). This analysis revealed that contribution of Dnmt3b’s AF to maintenance methylation may be higher than the CA itself because 80% of hypomethylated DMRs seen in *MYC;Dnmt3b*^*Δ/Δ*^ were not seen in *MYC;Dnmt3b*^*CI/CI*^. In contrast, both CA and AF appear to be relatively equally contributing to the generation of hypermethylated DMRs in particular in promoters possibly highlighting their equivalent role in *de novo* methylation. However, these conclusions were derived from differential analysis of mouse tumors in which numerous activities are deregulated providing a lot of variables that may affect data interpretation. Therefore, a caution has to be exercised in our data interpretation before more rigorous functional approaches confirm such conclusions. Nonetheless, our data point to involvement of Dnmt3b’s CA and AF in basic methylation functions in mouse lymphomagenesis.

Another interesting aspect of this study is finding that Dnmt3b’s CA is largely dispensable for postnatal development yet playing a role in fat metabolism and preventing development of ICF-like syndrome. Several features of *Dnmt3b*^*CI/CI*^ mice are consistent with human ICF syndrome including facial anomaly, reduced body weight, and hematopoietic defects, especially impaired lymphocyte development. We identified additional feature associated with ICF syndrome—cerebral hyperplasia. This is not associated with higher sensitivity to malignant development as we have not detected brain tumors in any of the analyzed mice. Rather, this result points to a typical clinical observation of psychological and cognitive developmental delay in observed in ICF patients (50).

Attempts to establish ICF model were previously done by generation of mice expressing analogues of human DNMT3B single-point mutations derived from ICF patients such as A609T mutant, which disrupts the interactions with Dnmt3a and Dnmt3b1, and D823G, altering protein localization (51). While homozygous mutant mice were born and had ICF-like features, they were not viable with most mice dying within 24 h.

*Dnmt3b*^*CI/CI*^ mice, like humans, survive postnatal development and resemble individuals with ICF, thus may serve as good models for understanding the etiology of ICF syndrome and identification of target genes regulated by DNA methylation during development.

## Methods

### Mouse studies

To generate conventional Dnmt3b knockout allele, we used the approach described previously (31, 36, 39) utilizing Dnmt3b^2loxP^ mice obtained from E. Li (Novartis Institutes for Biomedical Research, Cambridge, Massachusetts, USA). Mice were kept in FVB/N genetic background and were generated using standard genetic crosses. Mice harboring conventional knock-in mutations (P656 V and C657D) in Dnmt3b coding sequence (Dnmt3b^CI^) were generated as described before (17). *EμSRα-tTA;Teto-MYC* mice were obtained from D.W. Felsher (Stanford University). Mice harboring conditional knockout of Dnmt3b (*EμSRα-tTA;Teto-MYC;TetoCre; Rosa26LOXP*^*EGFP*^*;Dnmt3b*^*fl/fl*^ - termed *MYC;Dnmt3b*^*Δ/Δ*^) were generated as described before (31). For MYC-induced T-cell lymphomagenesis studies, *E*μ*SRα-tTA;Teto-MYC;Dnmt3b*^*+/+*^ (termed *MYC;Dnmt3b*^*+/+*^) and *E*μ*SRα-tTA;Teto-MYC;Dnmt3b*^*CI/CI*^ (termed *MYC;Dnmt3b*^*CI/CI*^) mice were harvested when terminally sick. Cells from lymphomas were analyzed by FACS, used for DNA isolation and WGBS. All experimental animal procedures were approved by the Institutional Animal Care and Use Committee (IACUC) at the University of Florida under protocol number 201609589 and complied with all relevant ethical regulations for animal testing and research. All mice were housed in a pathogen-free barrier facility at the UF.

### Histology

Formalin-fixed paraffin-embeded sections (4 μm) of the spleen, lymph node, and inguinal WAT were stained with hematoxylin (Sigma, H9627) for 40 s and with eosin (Sigma-Aldrich, HT110116) for 30 s. The tissue sections were mounted with Permount mounting medium (Fisher Scientific, SP15–100). All procedures were conducted by Molecular Pathology Core, University of Florida. Images were generated with a Zeiss Axio Imager 2 microscope (Carl Zeiss, Inc, Thornwood, NY). For adipose tissue analysis, adipocytes were counted for four nonoverlapping fields of view for three *Dnmt3b*^*+/+*^ and *Dnmt3b*^*CI/CI*^ mice. Cells with single large lipid droplet were considered white adipocytes and cells with multiple small droplets—brown.

### Skeletal staining

For cranium staining, mice heads were skinned and macerated in 2% KOH for 3 days. Eviscerated skulls were stained with 0.005% Alizarin Red (Sigma-Aldrich, A5533) solution for 4 days and washed with 1% KOH for 1 day to remove excess staining. Skeletons were transferred to glycerol and photographed using Zeiss Stemi 305 CAM Digital Stereo Zoom Microscope (Carl Zeiss, Inc, Thornwood, NY).

### Flow cytometry

Freshly isolated single-cell suspensions were prepared from the spleens, lymph nodes, bone marrow, and tumor masses. Red blood cells were lysed using ammonium–chloride–potassium (ACK) lysis buffer. Cells were stained with BioLegend antibodies for 30 min in 100 μl volume. Total B-cells (CD19+ B220+), myeloid cells (CD11b+CD5-), mature CD4+ T-cells (CD4+CD3+), and mature CD8+ T-cells (CD8+CD3+) were analyzed in spleen. Tumor burden in *Dnmt3b*^*+/−*^, *Dnmt3b*^*+/CI*^, *MYC;Dnmt3b*^*+/+*^*, MYC;Dnmt3b*^*Δ/Δ*^, *and MYC;Dnmt3b*^*CI/CI*^ mice was evaluated using CD4+ and CD8+ antibodies for PTCL and MTCL or CD19+, CD5+, IgM, and IgD antibodies for CLL and CD11b+ for myeloproliferation. Data were acquired on the LSRFortessa flow cytometers (BD Biosciences, San Diego, CA, USA) and analyzed with FACSDiva software (BD Biosciences, San Diego, CA, USA).

The analysis of clonality in tumors was performed by using mouse Vβ TCR screening kit (BD PharMingen). Aliquots of cell suspensions isolated from the spleens of terminally sick mice were stained with a panel of mAbs recognizing TCR vβ chain, including vβ 2, 3, 4, 5.1 and 5.2, 6, 7, 8.1 and 8.2, 8.3, 9, 10b, 11, 12, 13, 14, and 17a TCR per manufacturer's instruction. Tumors were considered monoclonal if they stained for one TCR.

### Combined bisulfite restriction analysis

COBRA was carried out as described previously (52). Briefly, bisulfite conversion of genomic DNA was carried out using the Epitect Bisulfite Kit (Qiagen). PCR products were digested with restriction enzymes *BstUI, TaqI*, *or TaiI* (NEB). Digested products were then loaded on an 8% PAGE gel, separated by electrophoresis, and stained by SYBR Gold (Invitrogen). Mouse bisulfite specific primers are shown in Supporting Information 5.

### WGBS and bioinformatic analysis

The WGBS libraries were prepared and sequenced on an Illumina NovaSeq6000 sequencer using 150 bp long paired-end reads (Novogen, USA). Publicly available WGBS data for mouse thymus control were obtained from ENCODE (Joe Ecker, Salk lab, ENCSR001MFH) (40). Quality check, trimming, filtering, and alignment of reads to the *Mus musculus* UCSC mm10 reference genome were performed at the ICBR Bioinformatics Core (UF, Florida). The aligned BAM files were uploaded to the Galaxy web platform (53). Methylation calling was performed with Methyldackel (v 0.3.0.1) using the mm10-CG index (https://github.com/dpryan79/MethylDackel.git). Only CpG sites with a minimum sequencing depth 5x were included in analysis. Methylation scores were visualized with the Integrated Genome Browser (IGB) (54). Scatter plots of methylation score were generated in Rstudio v1.1.4.6 using package gplots. Genome-wide Pearson correlation analysis of CpG sites was performed using deepTools package multiBigWigsummary and plotCorrelation (55). Differentialy methylated cytosines (DMCs) and differentially methylated regions (DMRs) were determined by Metilene (56). DMCs are defined as CpGs with methylation change of ≥30%. DMRs were defined based on average of minimum three consecutive DMCs with methylation change in the same direction ≥30% (p(MWU) < 0.05). Maximal base pair cutoff for a distance between consecutive DMCs in DMR was set to 50 bp. Annotation of methylated CpGs and DMRs to long promoters, core promoters, exons, introns, CGIs, enhancers, and repeats was performed using bedtools intersect. The DMR was retained if the overlap between these elements and DMR was at least 50% of the length of the DMR. Chromosomal coordinates of TSS, gene bodies, exons, introns, CGIs, and repeats were acquired from the USCS Table browser. Coordinates of enhancers identified in CD4+CD8+ cells and thymus cells were obtained from Enhancer atlas (57). Long promoter was defined as 1500 bp upstream to 500 bp downstream of the TSS. Core promoter was defined as 300 bp upstream to 150 bp downstream of the TSS.

### ChIP-seq data analysis

Publicly available data on chromatin modifications in mouse thymus from 8-week-old mice were obtained from ENCODE (Bing Ren, UCSD lab and John Stamatoyannopoulos, UW; ENCSR325LOF) (40). Heat map of DNAse I sensitivity assay and profiles of peak signals were generated using the computeMatrix, plotProfile, and plotHeatmap scripts from the deepTools3 package (55). Plots showing histone enrichment profiles across DMRs scaled to 200 bp were computed using 10 bp long nonoverlaping bins. Flanking unscaled −100 and +100 bp regions are shown. Plots presenting histone modification profiles and DNAseI sensitivity assay for genes with hypomethylated promoters that become upregulated in *Dnmt3b*^*+/−*^ and/or *Dnmt3a*^*Δ/Δ*^ lymphomas show unscaled region from −2000 bp to +2000 bp around TSS.

### RNA-seq

Library generation and sequencing were performed on NovaSeq 6000 platform using paired-end 150 bp runs (Novogene, USA). Previously published RNA-seq data for mice lymphomas driven by conditional loss of Dnmt3a (*EμSRα-tTA;Teto-Cre;Dnmt3a*^*fl/fl*^*;Rosa26LOXP*^*EGFP/EGFP*^) (36, 39) or expression of human MYC oncogene (16) (*EμSRα-tTA;Teto-MYC*) and control CD8+ T-cells (36) were added to analysis. Trimmed sequencing data were first aligned to *Mus musculus* UCSC mm10 reference genome using STAR aligner. RNA-seq data with minimum mapped quality 50 were quantified using the RNA-seq quantitation pipeline in SeqMonk software (http://www.bioinformatics.babraham.ac.uk/projects/seqmonk/). DeSeq2 was used to calculate differential expression. For differentially expressed genes, only genes with a fold change ≥2 and a *p* value <0.05 were considered to be significant. Reactome and Panther pathway analysis was conducted using WebGestalt (58). Ingenuity pathway analysis (Qiagen) (59) was used to analyze activated and decreased signaling pathways. Hierarchical clustering was performed in Cluster 3.0 (60), and heat maps were visualized in Java TreeView 3.0.

### Gene set enrichment analysis

All FPKM values for *Dnmt3b*^*+/−*^;*Dnmt3a*^*Δ/Δ*^ lymphomas and control CD8+ T-cells were converted to GCT expression data set. CLS files were generated using CLSFileCreator (v4) (http://software.broadinstitute.org/cancer/software/genepattern/modules/docs/ClsFileCreator/4). All *hallmarks* gene set was downloaded from Broad Institute’s Molecular Signatures Database. GSEA (v3.0) (61, 62) was used to test the relationship between RNA-seq expression data and the *All hallmarks* gene set. Gene sets enriched in less than 15 genes and more than 500 genes were excluded from the analysis. Gene sets with a false discovery rate (FDR) value <0.25 and *p* <0.05 after performing 1000 permutations were considered to be significantly enriched.

### Western blotting

Western blots were performed as previously described (31), using the following antibodies: Dnmt1 (sc271729, Santa Cruz; dilution 1:1000), Dnmt3a (sc-20703, Santa Cruz; dilution 1:1000), Dnmt3b (PA1-884, Thermo Fisher; dilution 1:1000), p53 (sc6243, Santa Cruz; dilution 1:1000), Hsc70 (sc-7298, Santa Cruz; dilution 1:10,000). Uncropped and unprocessed scans of blots are available in Source Data file.

### Real-time qRT-PCR

Total mRNA was isolated as described previously (31) from *Dnmt3b*^*+/+*^ and *Dnmt3b*^*CI/CI*^ liver. RNA was reverse transcribed with the SuperScript III Reverese transcriptase (Thermo Fisher) using oligo(dT) primers. Real-time qRT-PCR was performed with the iQ SYBR Green Supermix (Bio-Rad) on a CFX96 Touch Real-Time PCR Detection System (Bio-Rad). Fast PCR cycling conditions were used (95 °C for 3 min, 40 cycles (95 °C for 10 s, 58–63.5 °C for 30 s)), followed by a dissociation curve analysis. All qPCR measurements were performed in duplicate reactions and normalized to the expression of housekeeping gene (β-actin). In parallel, no-RT controls were amplified to rule out the presence of contaminating genomic DNA. Primer sequences for qPCR are provided in Supporting Information 5.

### Statistical analysis

Statistical significance of means ± SEM was evaluated using the two-tailed Student’s *t*-test. For all statistical analyses, *p* values <0.05 were considered significant. The significance between observed and expected genotype representation of *Dnmt3b*^*+/CI*^ and *Dnmt3b*^*CI/CI*^ mice was calculated using Chi-squared test. Differential histone enrichment was analyzed by Student’s *t*-test or Welch’s unequal variances *t*-test.

## Data Availability

All relevant data are available from the corresponding author upon reasonable request. The WGBS and RNA-seq data were deposited at the NCBI Gene Expression Omnibus database [GSE154270, GSE154451, GSE78146] (63).

## Conflict of interest

The authors declare that they have no conflicts of interest with the contents of this article.

## Supporting information

Figures S1–S22

Supporting informations 1–5
