# Supplementary material for: Decreases in different Dnmt3b activities drive distinct development of hematologic malignancies in mice
Source: J Biol Chem. 2021 Jan 13;296:100285. doi: 10.1016/j.jbc.2021.100285 (PMC7949038; doi:10.1016/j.jbc.2021.100285)
Supplement: Figures S1–S22 [file mmc1.pdf]

Supporting information for:

Decreases in different Dnmt3b activities drive distinct development of hematologic malignancies in mice

Katarina Lopusna*, Pawel Nowialis*, Jana Opavska, Ajay Abraham, Alberto Riva, Staci L. Haney, Rene Opavsky

*Equal contribution

Content:
Supporting figures S1-S22

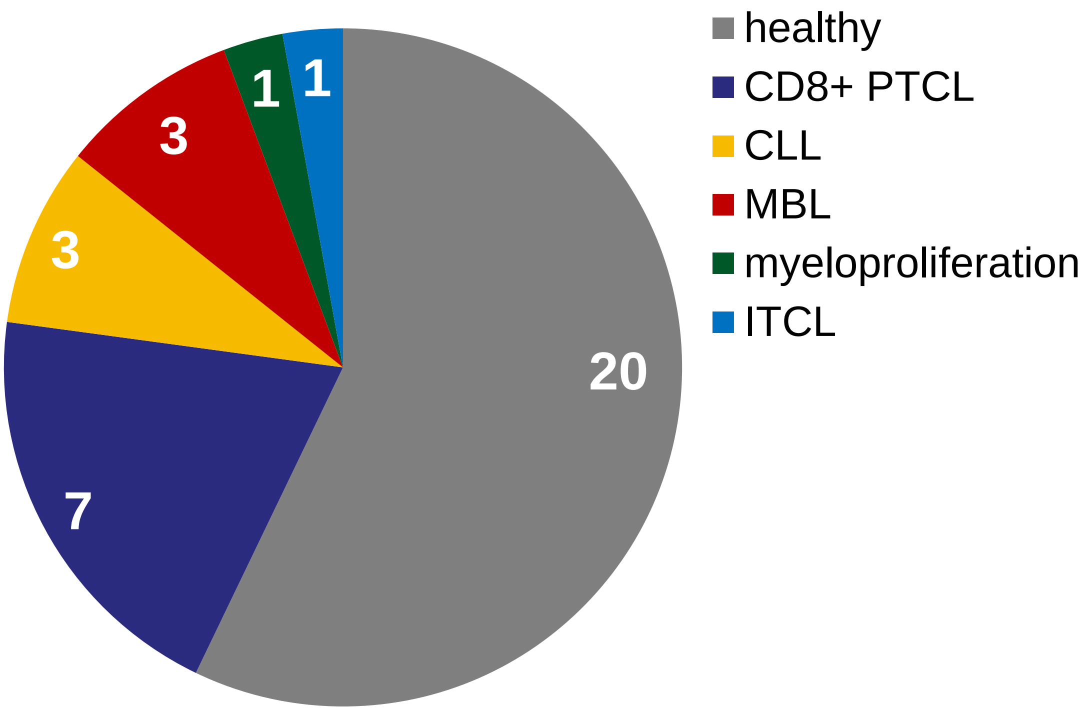

Figure S1. Disease spectrum observed in *Dnmt3b*$^{+/-}$ mice.
Dnmt3b$^{+/-}$ mice (n=35) were harvested as terminally sick. Single cell suspension of spleen and lymph node was prepared and analyzed by FACS. Data are presented as number of mice with detected phenotype.

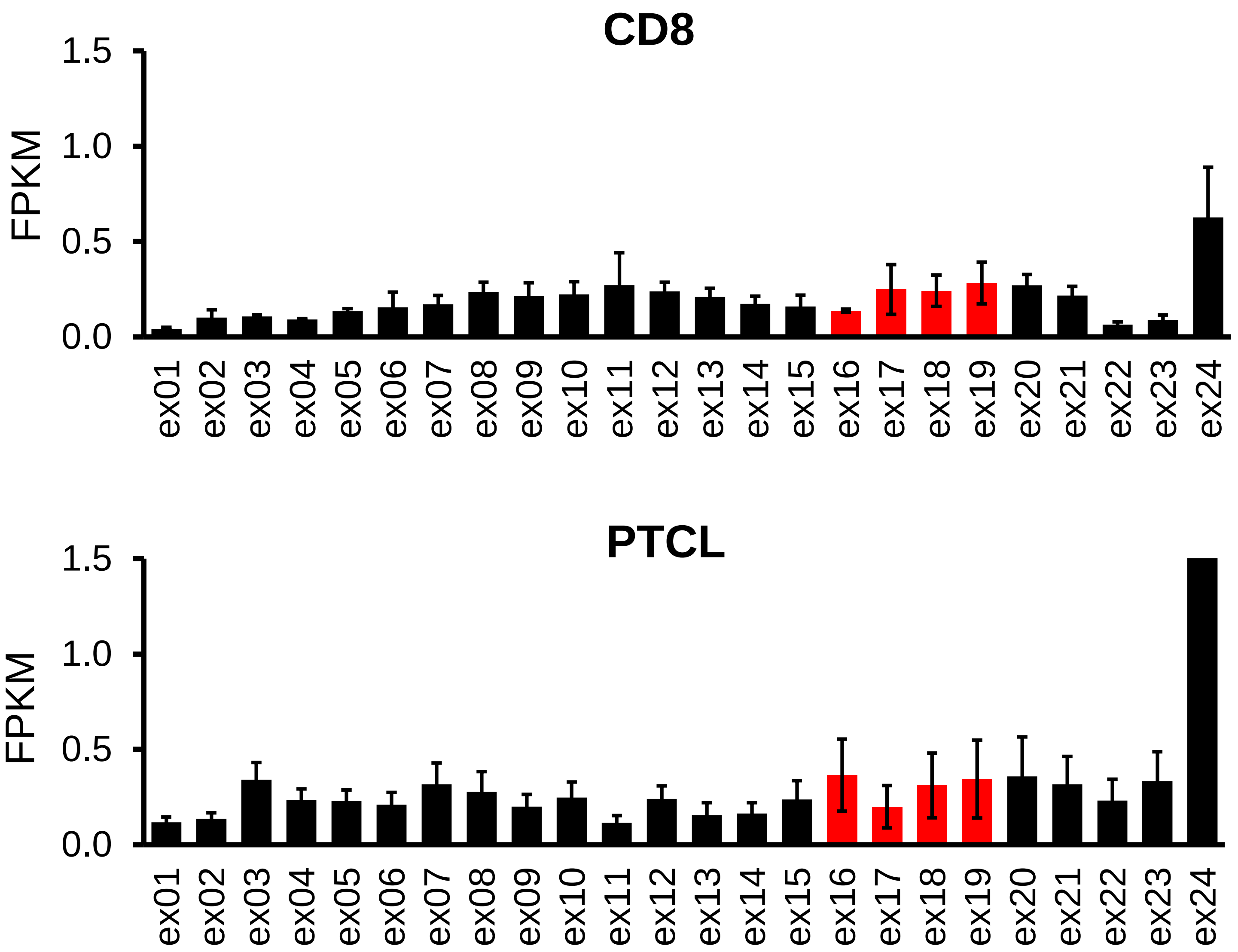

Figure S2. Normalized read counts across exons of Dnmt3b in control CD8+ T-cells and *Dnmt3b*$^{+/-}$ PTCL.

Reads acquired by RNA-seq of normal CD8+ T-cells (n=2) and *Dnmt3b*$^{+/-}$ PTCL (n=3) were normalized to the length of the exon in Dnmt3b gene and to the total number of reads per each sample. Data are presented as means ± SEM. Red indicates exons flanked by LoxP sites (exon 16-19) in Dnmt3b gene.

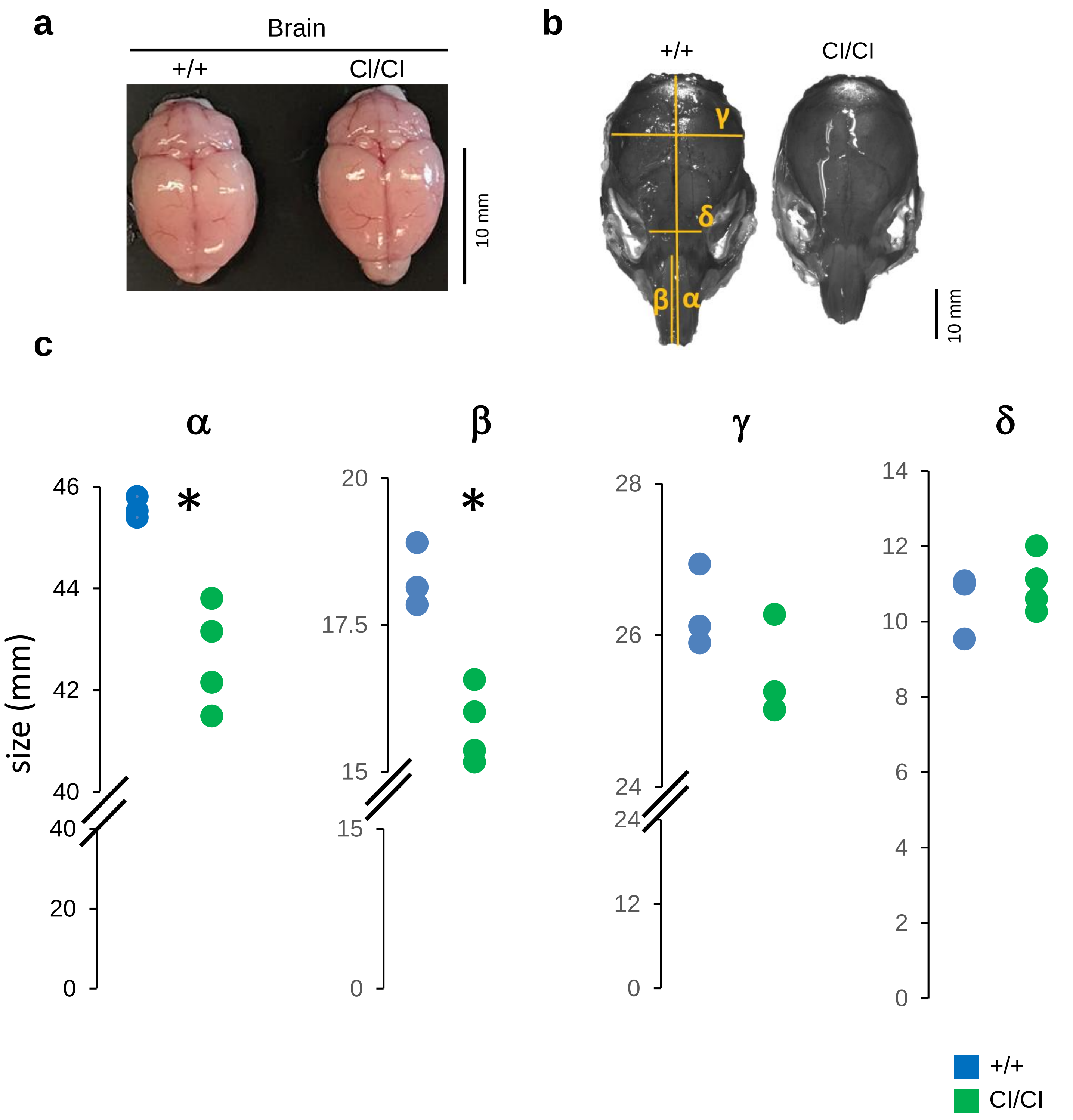

Figure S3. *Dnmt3b$^{CI/CI}$* mice have shortened nose and brain hyperplasia, characteristic for ICF-like syndrome.

a. Representative image of brains of *Dnmt3b$^{+/+}$* and *Dnmt3b$^{CI/CI}$* mice.

b. Gross morphology of skulls of *Dnmt3b$^{+/+}$* and *Dnmt3b$^{CI/CI}$* mice.

c. Dimensions of skulls isolated from *Dnmt3b$^{+/+}$* (n=3) and *Dnmt3b$^{CI/CI}$* (n=4) mice. Orientation of α, β, γ and δ dimensions is depicted by orange lines on the image in (**b**). *p=0.005 for α; p=0.003 for β by two-tailed Student's t-test.

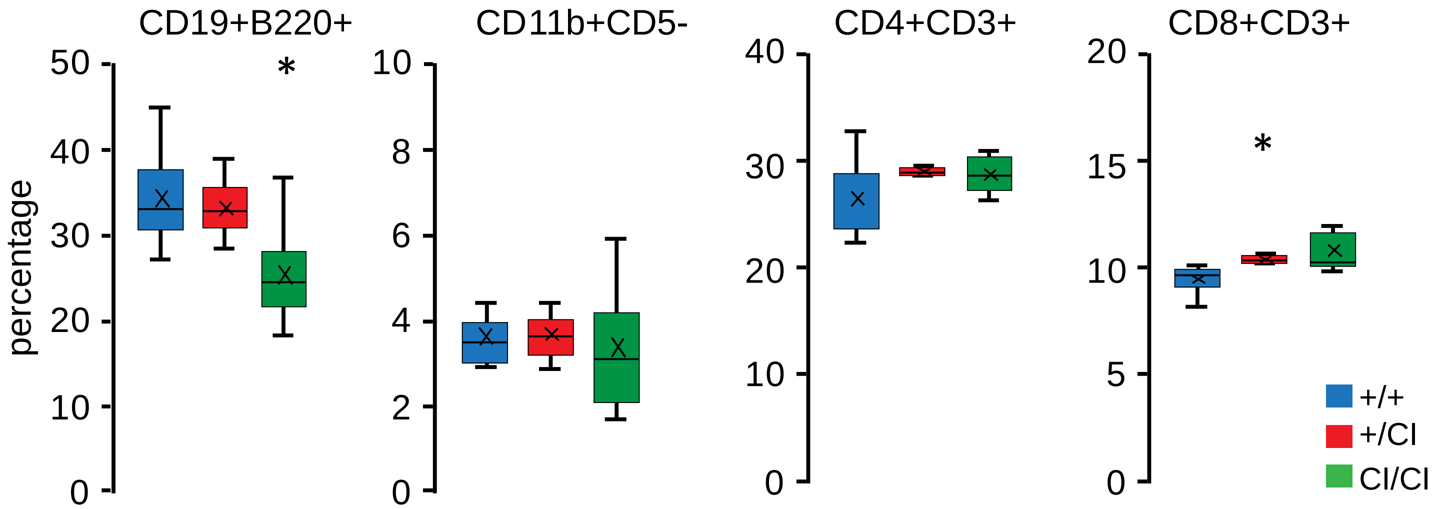

Figure S4. Percentage of B-, T- and myeloid cells in *Dnmt3b$^{+/CI}$* spleen is similar to *Dnmt3b$^{+/+}$* mice.
Percentage of total B-cells (CD19+B220+), myeloid cells (CD11b+CD5-) and mature T-cells (CD4+CD3+ and CD8+CD3+) in spleen of *Dnmt3b$^{+/+}$* (n=6) *Dnmt3b$^{+/CI}$* (n=2) and *Dnmt3b$^{CI/CI}$* mice (n=6). Horizontal line represents median, bounds of box—likely range of variation and whiskers—min and max values. *p < 0.05 by two-tailed Student’s t-test.

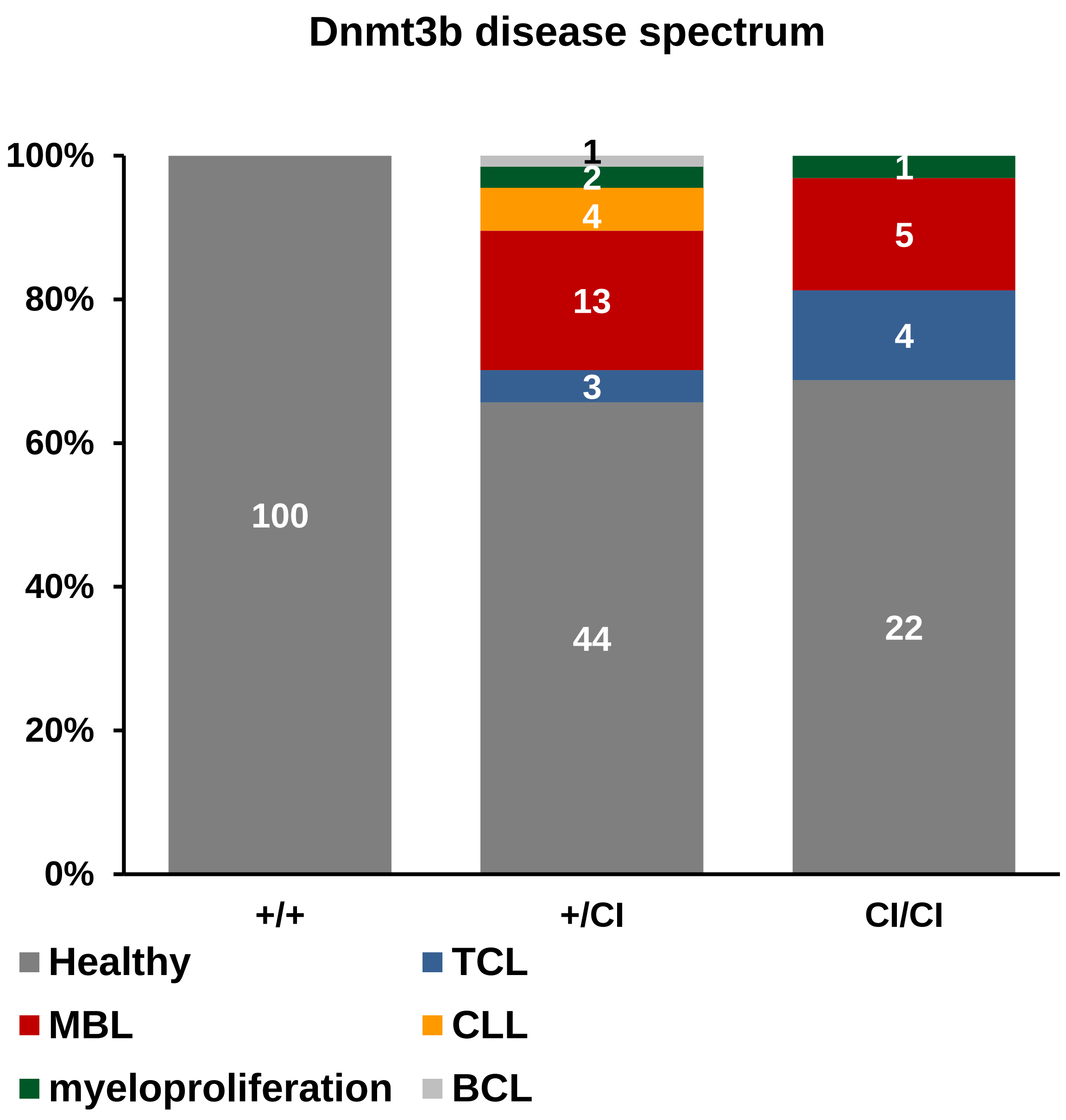

Figure S5 Disease spectrum observed in *Dnmt3b*$^{+/CI}$ *and Dnmt3b*$^{CI/CI}$ mice. *Dnmt3b*$^{+/CI}$ (+/CI; n=67) and *Dnmt3b*$^{CI/CI}$ (CI/CI; n=32) mice were harvested as terminally sick. Single cell suspension of spleen and lymph node was prepared and analyzed by FACS. Data are presented as number of mice with detected phenotype.

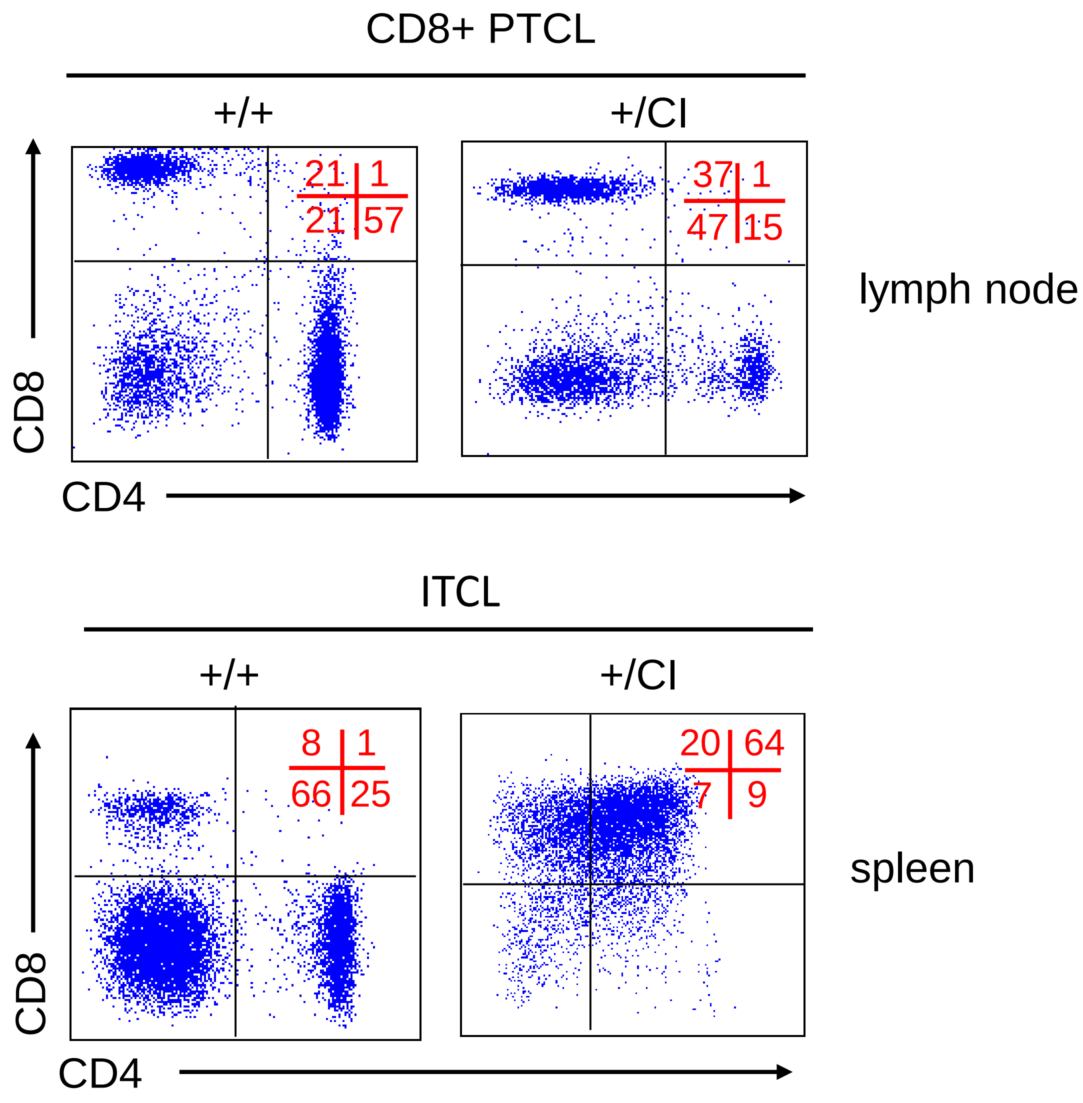

Figure S6. *Dnmt3b*$^{CI/CI}$ mice develop T-cell lymphoma.
Representative FACS diagrams showing expression of CD4 and CD8 in lymphocytes isolated from *Dnmt3b*$^{+/+}$ healthy control and *Dnmt3b*$^{CI/CI}$ mice that developed CD8+ PTCL (top) and immature CD4+CD8+ T-cell lymphoma (ITCL; bottom). Quadrant statistics is presented on top right.

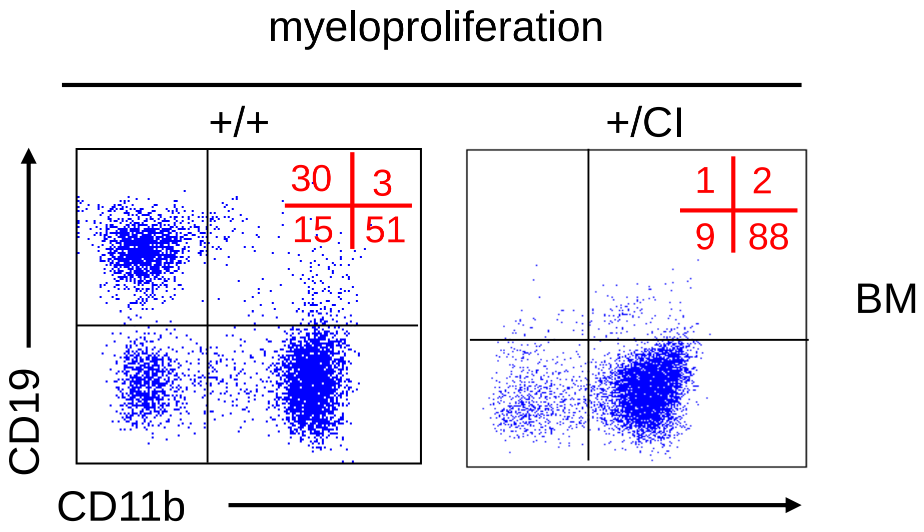

Figure S7. *Dnmt3b*$^{+/CI}$ mice develop myeloproliferative disease.
Representative FACS diagrams showing expression of CD19 and CD11b in lymphocytes isolated from bone marrow of *Dnmt3b*$^{+/+}$ healthy control and *Dnmt3b*$^{+/CI}$ mice that myeloproliferative disease. Quadrant statistics is presented on top right.

Sample	Paired end reads before QC	Paired end reads after QC and filtering	Aligned reads	Mapping efficiency	CpG 5x
Th1	1,064,276,391	1,005,183,826	421,293,754	41.91%	20,028,501
Th2	1,139,704,978	1,077,910,865	449,979,971	41.75%	19,183,719
Dnmt3b$^{+/-}$ 1	531,263,266	475,327,587	316,817,632	66.65%	16,166,103
Dnmt3b$^{+/-}$ 2	265,442,207	255,451,250	148,130,997	57.99%	8,371,129
Dnmt3a$^{\Delta/\Delta}$ 1	342,481,149	329,232,689	188,186,272	57.16%	9,856,843
Dnmt3a$^{\Delta/\Delta}$ 2	363,548,192	299,427,583	194,707,773	65.03%	14,879,373

Figure S8. Summary of data obtained from the sequenced Whole genome bisulfite sequencing (WGBS) libraries.

Post sequencing quality check of WGBS data obtained from DNA of normal *Dnmt3b*$^{+/+}$ thymus control (n=2) and *Dnmt3b*$^{+/-}$ and *Dnmt3a*$^{\Delta/\Delta}$ lymphomas (n=2 each). The last column indicate the number of CpGs covered at least 5x.

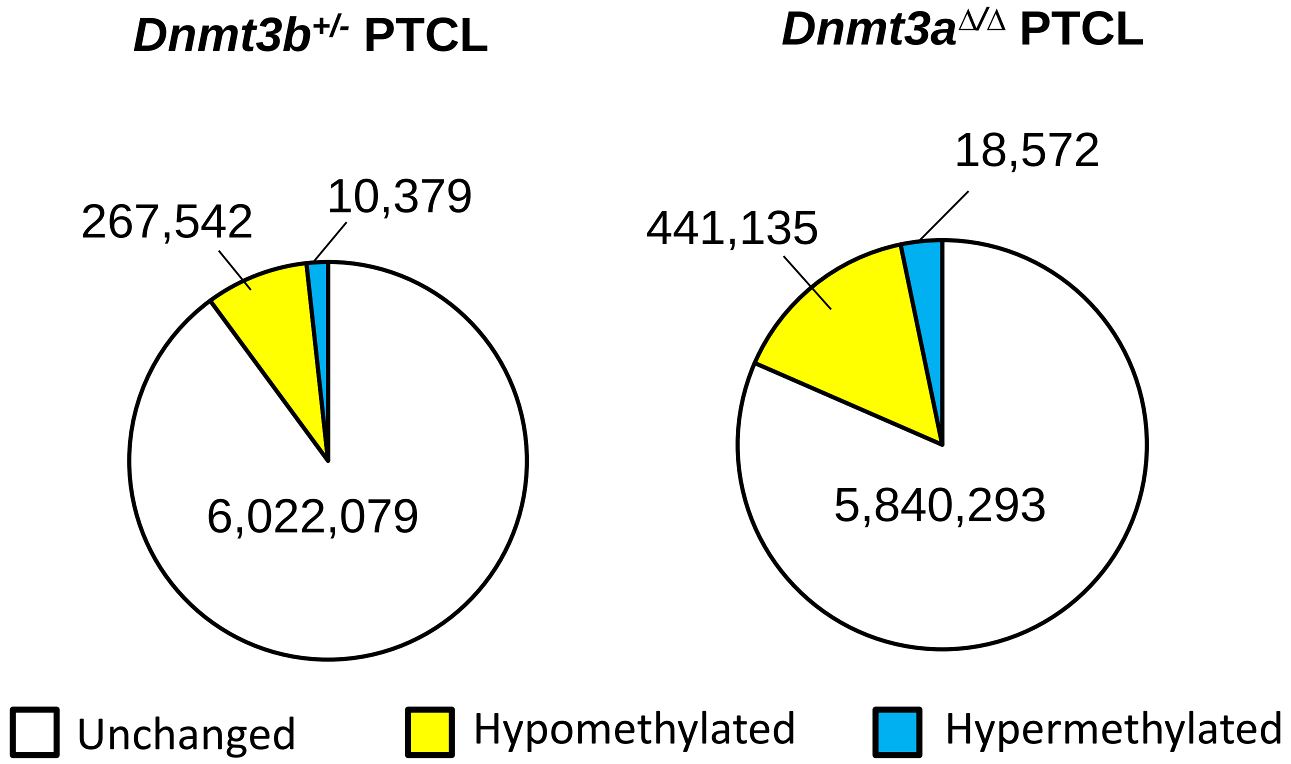

Figure S9. Total number of differentially methylated CpGs in *Dnmt3b*$^{+/-}$ and *Dnmt3a*$^{\Delta/\Delta}$ PTCL.

Differentially methylated CpGs identified in *Dnmt3b*$^{+/-}$ (n=2) and *Dnmt3a*$^{\Delta/\Delta}$ (n=2) PTCL when compared to normal thymus control (n=2). Differentially methylated CpGs are defined as either hypo- (yellow) or hypermethylated (blue) by ≥ 30% change in percent CpG methylation in tumor samples compared to thymus control samples. CpGs not meeting these criteria are shown in white (unchanged).

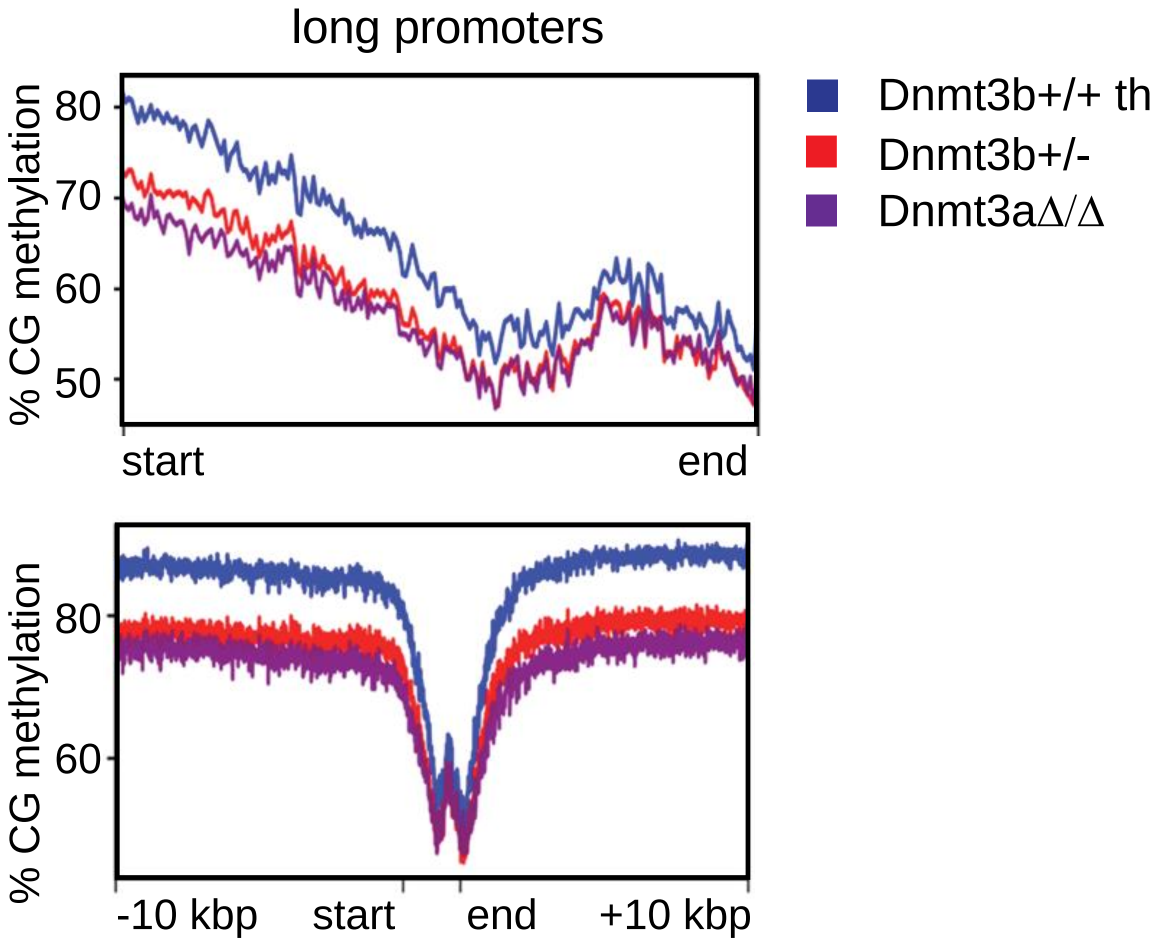

Figure S10. Distribution of CpG methylation in the long promoters and region from −10 to +10 kbp around TSS in *Dnmt3b*$^{+/+}$ Thymus, *Dnmt3b*$^{+/-}$ PTCL and *Dnmt3a*$^{\Delta/\Delta}$ PTCL.

Percentage of CG methylation was obtained by WGBS from genomic DNA isolated from *Dnmt3b*$^{+/+}$ thymus (n=2), *Dnmt3b*$^{+/-}$ PTCL (n=2) and *Dnmt3a*$^{\Delta/\Delta}$ PTCL (n=2). Methylation levels were visualized by IGB software and are presented as percentage of methylation for indicated regions.

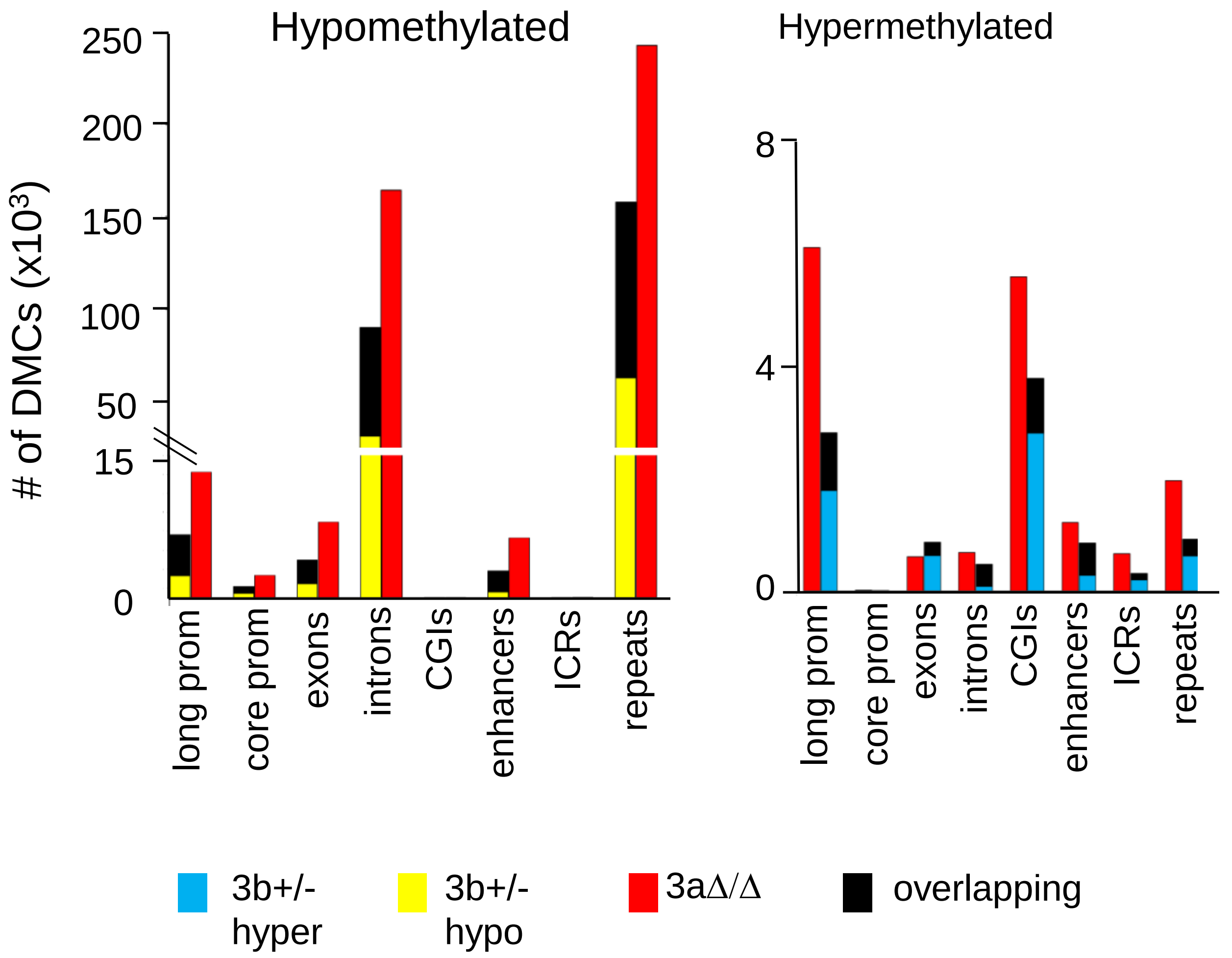

Figure S11. Distribution of hypomethylated and hypermethylated DMCs across genomic features.

The number of DMCs associated with long promoters (−1,500 to +500 bp relative to TSS), core promoters (−300 to +150 bp relative to TSS), exons, introns, CGIs, enhancers, ICRs and repeats identified in *Dnmt3b*$^{+/-}$ PTCL (n=2) and *Dnmt3a*$^{\Delta/\Delta}$ PTCL (n=2) when compared to normal thymus control (n=2).

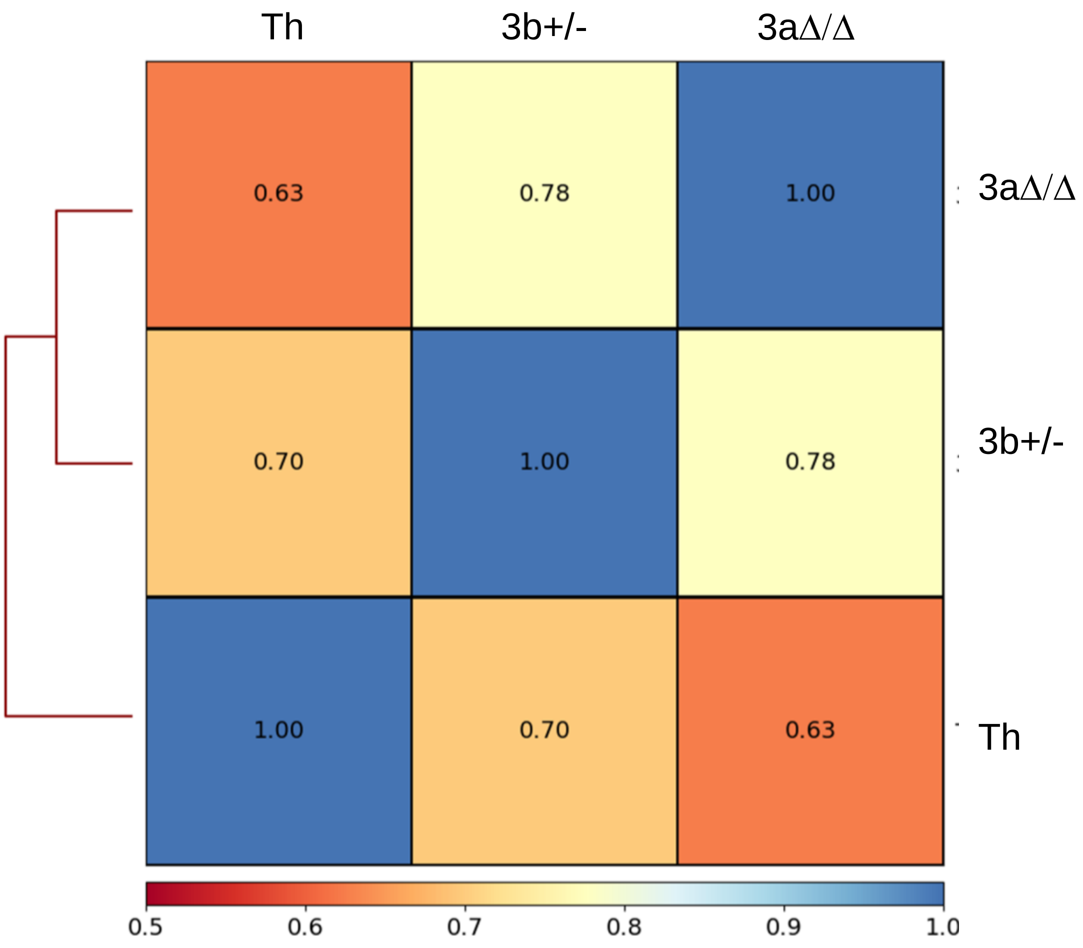

Figure S12. Correlation analysis of methylomes of *Dnmt3b*$^{+/-}$ and *Dnmt3a*$^{Δ/Δ}$ lymphomas.
Pearson correlation coefficients and hierarchical clustering of genome-wide methylation data. Clustering of the samples and correlation analysis was based on methylation values of all CGs present in all analyzed samples (*Dnmt3a*$^{Δ/Δ}$ (3aΔ/Δ; n=2), *Dnmt3b*$^{+/-}$ (3b+/-; n=2) and control thymus (Th; n=2) covered at least 5x. Pearson correlation analysis was performed using deepTools packages multiBigWigsummary and plotCorrelation.

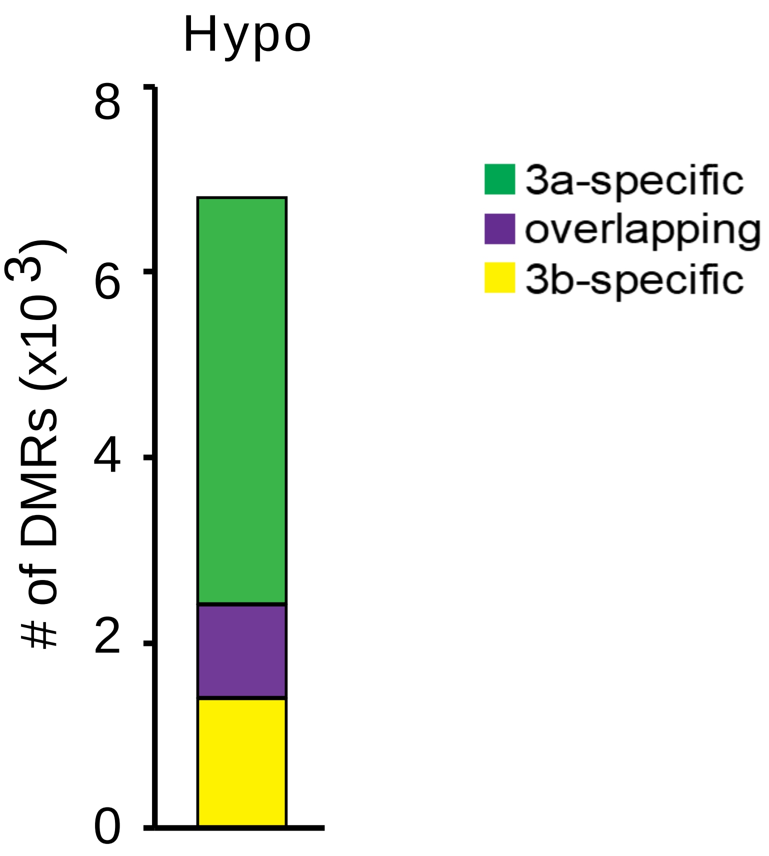

Figure S13. Overlap between hypomethylated DMRs identified in *Dnmt3b*$^{+/-}$ and *Dnmt3a*$^{\Delta/\Delta}$ PTCL.
Total number of hypomethylated DMRs (≥3 consecutive DMCs in the same direction in the distance ≤50 bp, average DMC methylation change ≥30%) identified in *Dnmt3b*$^{+/-}$ (n=2) and *Dnmt3a*$^{\Delta/\Delta}$ (n=2) PTCL when compared to normal thymus control (n=2). Presented are only those DMRs which were not identified in *Dnmt3b*$^{+/+}$ MYC-induced T-cell lymphoma (n=2) when compared to control thymus.

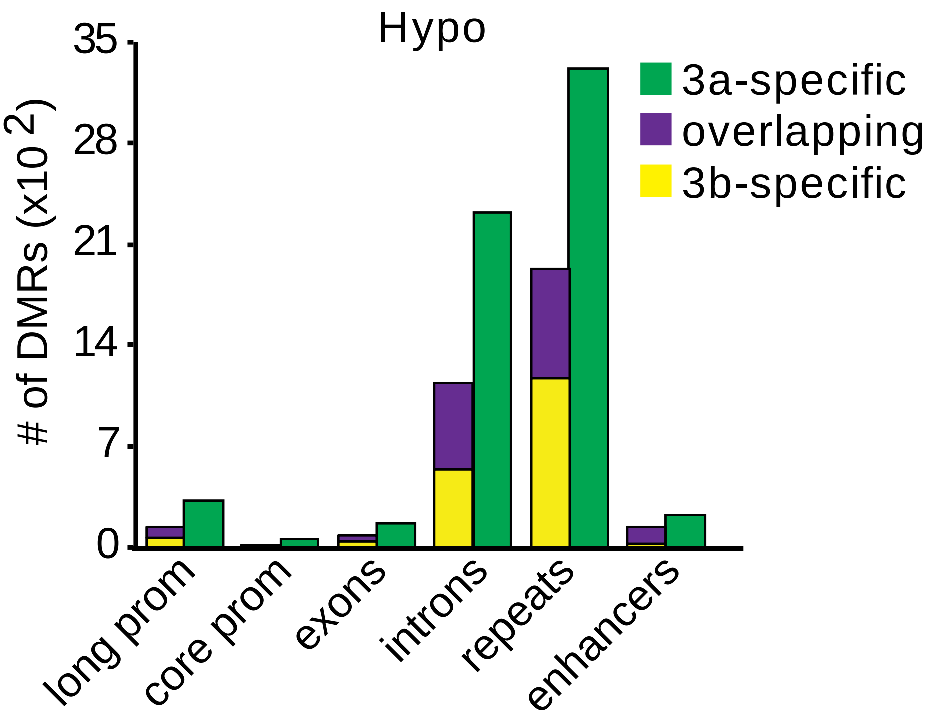

Figure S14. Distribution of hypomethylated DMRs among indicated genomic elements.

The number of DMRs (≥3 consecutive DMCs in the same direction in the distance ≤ 50 bp, average DMC methylation change ≥30%) associated with long promoters (−1,500 to +500 bp relative to TSS), core promoters (−300 to +150 bp relative to TSS), exons, introns, enhancers and repeats identified in *Dnmt3b*$^{+/-}$ PTCL (n=2) and *Dnmt3a*$^{\Delta/\Delta}$ PTCL (n=2) when compared to normal thymus control (n=2). Presented are only those DMRs which were not identified in *Dnmt3b*$^{+/+}$ MYC-induced T-cell lymphoma (n=2) when compared to control thymus.

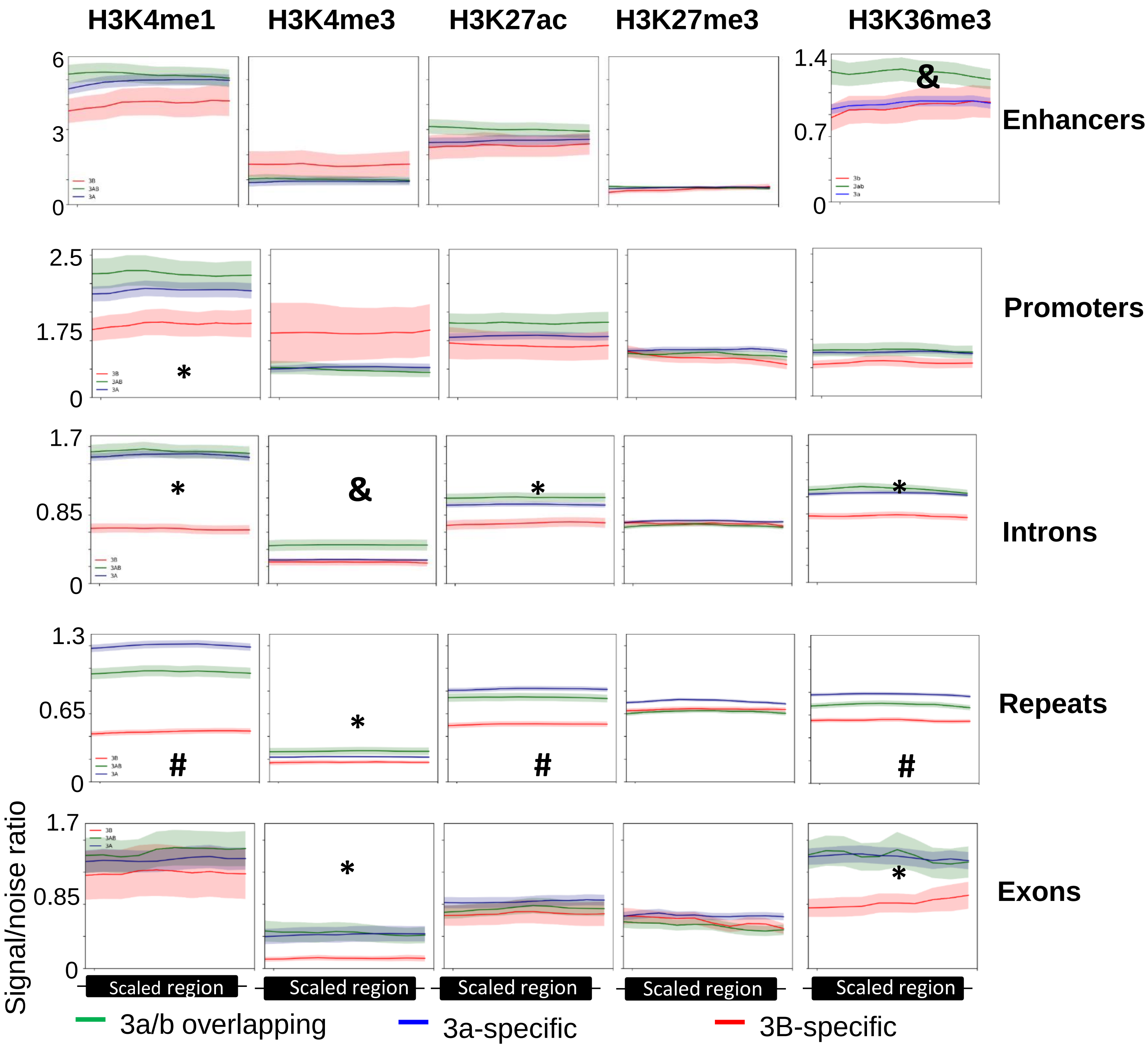

Figure S15. Enrichment of *3b-specific*, *3a-specific* and *3a/b overlapping* DMRs for chromatin marks in control *Dnmt3b*$^{+/+}$ thymus.
Enrichment of Dnmt3b-specific, Dnmt3a-specific and Dnmt3a/b overlapping DMRs for indicated chromatin marks within indicated genomic elements in control Dnmt3b$^{+/+}$ thymus as detected by analysis of ChIP-seq. Plots shows mean levels ± SEM for DMRs scaled to the same length and surrounding 100 bp unscaled regions. Statistical significance was calculated by Student's t-test and Welch's t-test; *denotes statistically significant difference ($p<0.05$) between 3b-specific and other groups of DMRs. & denotes statistically significant difference ($p<0.05$) between 3a/b-overlapping and other groups of DMRs. # denotes all DMR groups significantly different from each other ($p<0.05$).SEM values are presented as shading around mean value line.

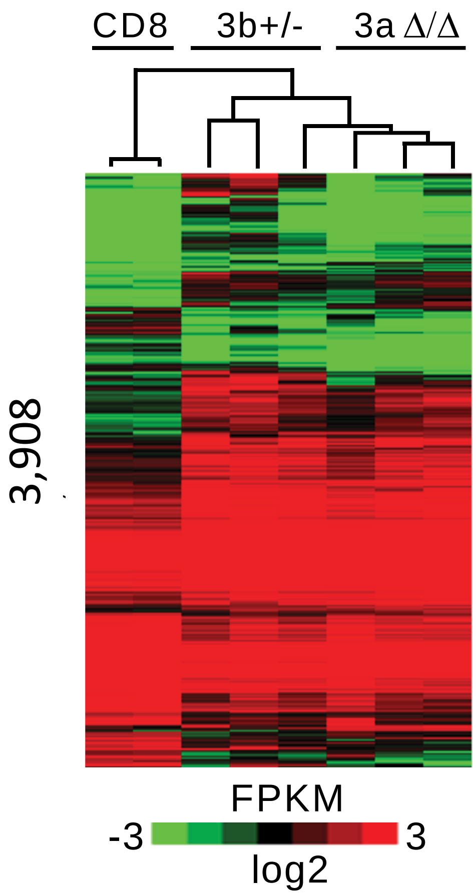

Figure S16. *Dnmt3b*$^{+/-}$ and *Dnmt3a*$^{\Delta/\Delta}$ lymphomas cluster together separate from normal CD8+ T-cells.

Heat map showing $\log_2$(FPKM) expression values of differentially expressed genes (FC≥3, p<0.05 by DESeq) in *Dnmt3b*$^{+/-}$ (3b+/-; n=3) and *Dnmt3a*$^{\Delta/\Delta}$ (3aΔ/Δ; n=3) lymphomas when compared to control CD8+ T-cells (CD8; n=2). Dendrogram above shows hierarchical clustering of lymphoma samples based on presented subset of genes.

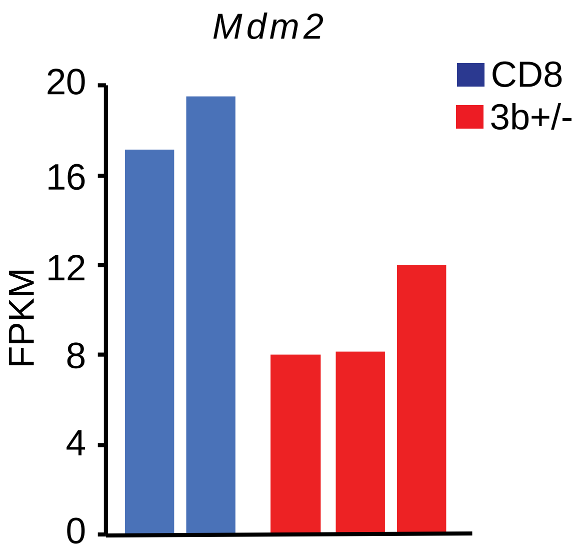

Figure S17. Negative regulator of p53 – Mdm2 is not upregulated in *Dnmt3b*$^{+/-}$ lymphomas.
Mdm2 expression by RNA-seq in control CD8+ T-cells (CD8; n=2) and *Dnmt3b*$^{+/-}$ (3b+/-; n=3) lymphomas.

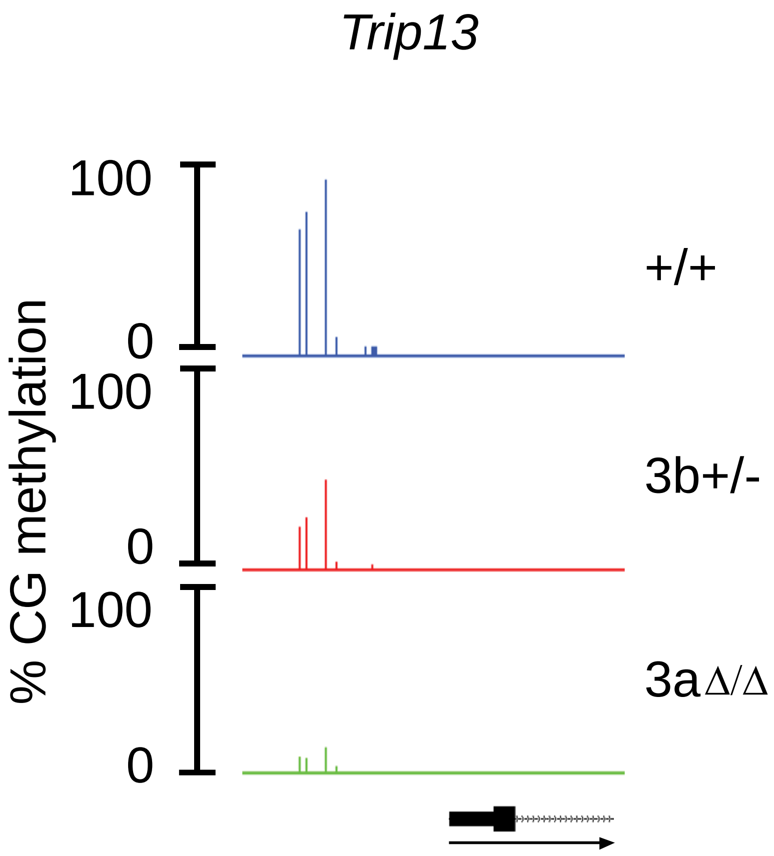

Figure S18. DNA methylation analysis of promoter of putative target of cooperative activity of Dnmt3b and Dnmt3a.
Visualization of single-CG profiles of *Trip13* promoter locus in *Dnmt3b*$^{+/-}$ (3b+/-) and *Dnmt3a*$^{\Delta/\Delta}$ (3aΔ/Δ) lymphomas and in healthy thymus (+/+).

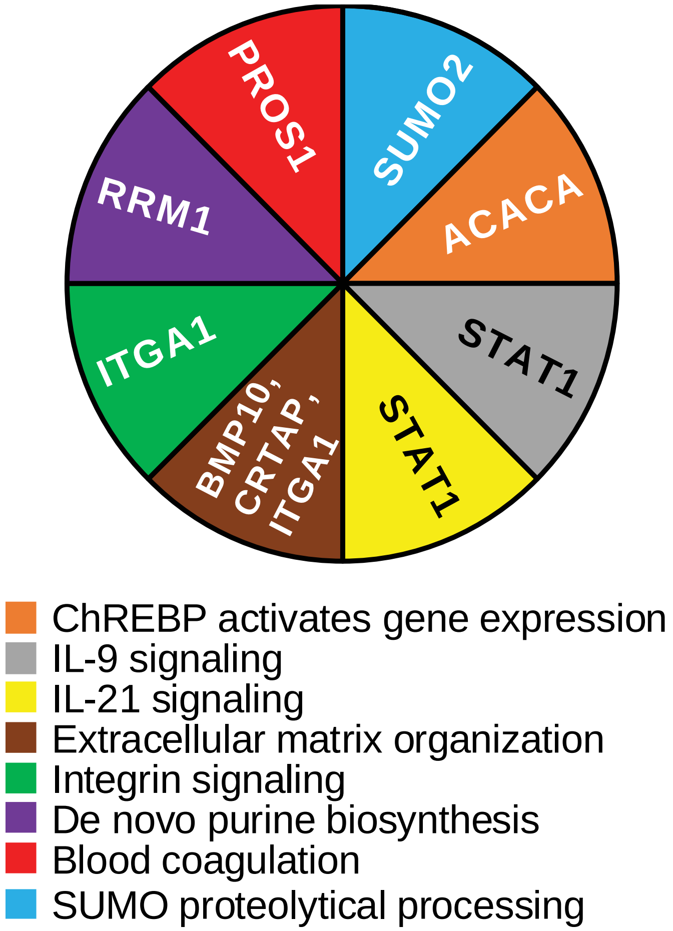

Figure S19. Panther and Reactome pathway analysis of genes hypomethylated in long promoter and overexpressed in *Dnmt3b*$^{+/-}$ PTCL.

Genes with hypomethylated DMR identified within their promoter and upregulated (FC≥2, p<0.05 by DESeq) in Dnmt3b$^{+/-}$ PTCL when compared to control thymus were included in analysis. Main enriched categories with gene identities is shown.

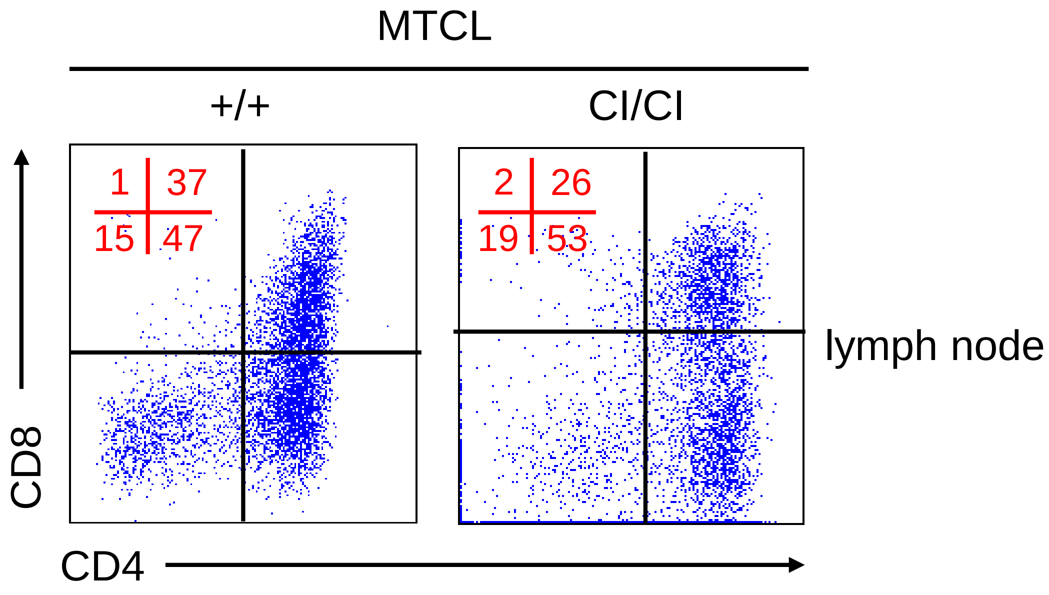

Figure S20. ***MYC;Dnmt3b***$^{+/+}$ **and** ***MYC;Dnmt3b***$^{CI/CI}$ **mice develop CD4+CD8+ double positive T-cell lymphomas**.
Representative FACS diagrams showing expression of CD4 and CD8 in lymphocytes isolated from lymph node of terminally ill *MYC;Dnmt3b*$^{+/+}$ (+/+) and *MYC;Dnmt3b*$^{CI/CI}$ (CI/CI) mice. Quadrant statistics is presented in red.

Sample	Paired end reads before QC	Paired end reads after QC and filtering	Aligned reads	Mapping efficiency	CpG 5x
MYC; Dnmt3b$^{+/+}$ 1	270,728,679	262,602,237	173,664,712	66.13%	15,193,499
MYC; Dnmt3b$^{+/+}$ 2	308,553,861	299,685,724	196,259,430	65.49%	15,679,969
MYC; Dnmt3b$^{\Delta/\Delta}$ 1	314,495,771	305,704,187	202,783,058	66.32%	15,866,511
MYC; Dnmt3b$^{\Delta/\Delta}$ 2	309,912,864	300,507,157	190,333,766	63.34%	14,389,764
MYC; Dnmt3b$^{CI/CI}$ 1	329,954,545	319,527,942	201,955,043	63.20%	16,816,756
MYC; Dnmt3b$^{CI/CI}$ 2	285,079,066	276,128,188	176,215,172	63.82%	15,762,095

Figure S21. Summary of data obtained from WGBS of MTCL lymphomas. Post sequencing quality check of WGBS data obtained from DNA of *MYC;Dnmt3b*$^{+/+}$*, MYC;Dnmt3b*$^{\Delta/\Delta}$ *and MYC;Dnmt3b*$^{CI/CI}$ lymphomas (n=2 each). The last column indicate the number of CpGs sequenced at least 5x.

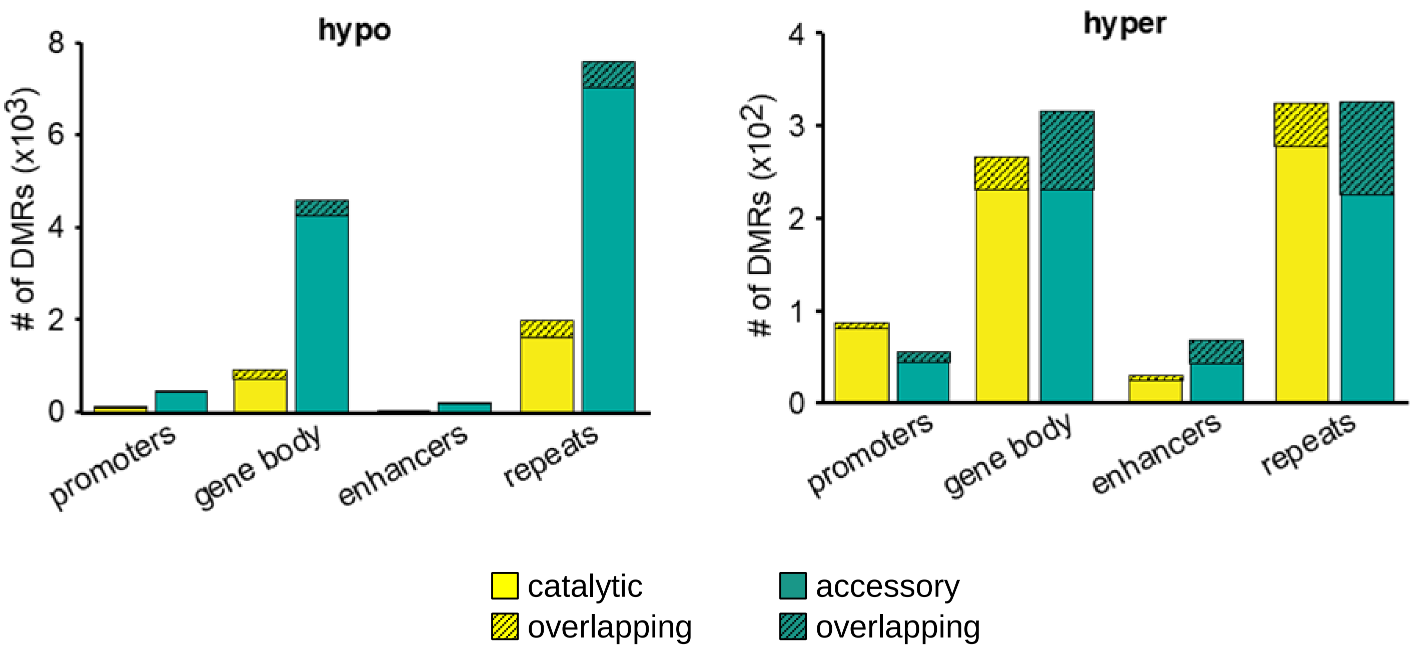

Figure S22. Number of hypomethylated and hypermethylated DMRs distributed among indicated genomic elements.

Percentage of catalytic and accessory targets (defined in **Fig. 8d**) associated with promoters, gene bodies, enhancers and repeats. Fraction of DMRs identified also in *Dnmt3b*$^{+/-}$ lymphomas is marked with striped pattern (overlapping).
