# Supplementary material for: Decreases in different Dnmt3b activities drive distinct development of hematologic malignancies in mice
Source: J Biol Chem. 2021 Jan 13;296:100285. doi: 10.1016/j.jbc.2021.100285 (PMC7949038; doi:10.1016/j.jbc.2021.100285)
Supplement: Supporting informations 1–5 [file mmc2.pdf]

Supporting Information for Fig. 6e - Fold change and FPKM expression values, and region coordinates underlying presented heat maps.

Expression values			Visualized region				
CD8 (FPKM)	TCL (FC)	Chr#	Start	End	Gene name	strand	Group
0.07383821	5.60892919	chr1	135605295	135609295	Nav1	-	G1
0.0108051	11.2452159	chr13	115099964	115103964	Itga1	-	G1
0.27764506	16.230708	chr17	35174068	35178068	Aif1	-	G1
4.15508315	2.63756622	chr11	79528609	79532609	Evi2a	-	G1
0.18361418	8.15398593	chr14	54415780	54419780	Slc7a7	-	G1
0.25690133	23.3305023	chr3	142592846	142596846	Gbp2b	+	G1
0.21544033	7.53374215	chr11	52002220	52006220	Cdkl3	+	G1
1.0255411	5.65897493	chr5	149526679	149530679	Wdr95	+	G1
0.01513203	37.3949204	chr6	87426993	87430993	Bmp10	+	G1
3.59790412	2.11975433	chr7	102439694	102443694	Rrm1	+	G1
1.45212274	2.23740882	chr11	84127676	84131676	Acaca	+	G1
0.45340274	14.6150765	chr11	53517257	53521257	Sept8	+	G1
0.36143411	33.8183958	chr16	62852306	62856306	Pros1	+	G1
0.10733459	#N/A	chr9	80263450	80267450	Gm27227	-	G2
0.11814084	#N/A	chr11	96462547	96466547	Gm11529	-	G2
0.11969269	#N/A	chr3	90241512	90245512	Creb3l4	-	G2
0.12444038	#N/A	chr2	153527971	153531971	Nol4l	-	G2
0.14965372	#N/A	chr3	29891177	29895177	Gm33206	-	G2
0.17466015	#N/A	chr8	18976256	18980256	Defb40	-	G2
0.40903178	#N/A	chr4	40720398	40724398	Gm6297	-	G2
0.83245257	#N/A	chr9	111055519	111059519	Ccrl2	-	G2
0.91003867	#N/A	chr7	127510869	127514869	1700008J07R	-	G2
1.33449495	#N/A	chr6	118560226	118564226	Ankrd26	-	G2
1.51928909	#N/A	chr15	99472730	99476730	Bcdin3d	-	G2
1.77229886	#N/A	chr4	134285895	134289895	Pdik1l	-	G2
1.78582172	#N/A	chr17	51177352	51181352	Tbc1d5	-	G2
1.81117383	#N/A	chr4	34776423	34780423	Smim8	-	G2
2.25758287	#N/A	chr2	127790488	127794488	1500011K16F	-	G2
2.25830332	#N/A	chr2	121138653	121142653	Lcmt2	-	G2
3.04108743	#N/A	chr4	119420420	119424420	Ppcs	-	G2
3.49107943	#N/A	chr5	113991894	113995894	Ssh1	-	G2
4.22191297	#N/A	chr12	102741661	102745661	Moap1	-	G2
5.03735523	#N/A	chr8	14088301	14092301	Erich1	-	G2
5.76285373	#N/A	chr17	15525301	15529301	Pdcd2	-	G2

5.81872038	#N/A	chr11	86542805	86546805	Rps6kb1	-	G2
6.55023013	#N/A	chr2	150902741	150906741	Abhd12	-	G2
6.70957426	#N/A	chr10	91179315	91183315	Tmpo	-	G2
7.1999465	#N/A	chr17	36256069	36260069	Rpp21	-	G2
10.3603769	#N/A	chr5	21783251	21787251	Dnajc2	-	G2
11.8737316	#N/A	chr10	54074609	54078609	Man1a	-	G2
13.6565585	#N/A	chr12	108001602	108005602	Bcl11b	-	G2
17.8672817	#N/A	chr14	40964807	40968807	Tspan14	-	G2
21.4274048	#N/A	chr15	81398077	81402077	St13	-	G2
24.1309794	#N/A	chr3	88459182	88463182	Sema4a	-	G2
43.559771	#N/A	chr16	87493873	87497873	Cct8	-	G2
48.8121171	#N/A	chr7	127135823	127139823	Spn	-	G2
49.5155247	#N/A	chr11	115512387	115516387	jpt1	-	G2
57.1048667	#N/A	chr9	22133711	22137711	Acp5	-	G2
108.487366	#N/A	chr15	102229935	102233935	Itgb7	-	G2
129.708269	#N/A	chr15	78493066	78497066	Il2rb	-	G2
157.610305	#N/A	chr4	129571641	129575641	Lck	-	G2
0.10186326	#N/A	chr1	185089991	185093991	Gm37662	+	G2
0.1063767	#N/A	chr9	107545298	107549298	Zmynd10	+	G2
0.12004027	#N/A	chr2	169387081	169391081	Gm14250	+	G2
0.13922704	#N/A	chr3	96266654	96270654	Hist2h3b	+	G2
0.15031591	#N/A	chr8	21944762	21948762	Defb13	+	G2
0.21298679	#N/A	chr3	152941313	152945313	Gm16231	+	G2
0.29376868	#N/A	chr11	17951970	17955970	Etaa1os	+	G2
0.34401608	#N/A	chr19	29044245	29048245	1700018L02F	+	G2
0.8362784	#N/A	chr11	86542991	86546991	Tubd1	+	G2
0.94454122	#N/A	chr1	58971522	58975522	Stradb	+	G2
1.07966951	#N/A	chr7	25150457	25154457	D930028M14	+	G2
1.2329883	#N/A	chr2	121138428	121142428	Adal	+	G2
2.10941335	#N/A	chr11	67453437	67457437	Gas7	+	G2
2.21581478	#N/A	chr7	45015961	45019961	Rras	+	G2
3.30713027	#N/A	chr9	48493442	48497442	Gm5617	+	G2
4.86552128	#N/A	chr17	74336987	74340987	Spast	+	G2
4.94443962	#N/A	chr11	97683826	97687826	Cisd3	+	G2
6.73015137	#N/A	chr1	171915515	171919515	Slamf6	+	G2
7.06448576	#N/A	chrX	101530734	101534734	Taf1	+	G2
8.30345132	#N/A	chr14	55601571	55605571	Irf9	+	G2
9.82598649	#N/A	chr2	160643888	160647888	Top1	+	G2
9.88511358	#N/A	chr13	73935811	73939811	Brd9	+	G2
10.0208329	#N/A	chr4	128652702	128656702	Phc2	+	G2
12.1855638	#N/A	chr17	31205873	31209873	Ubash3a	+	G2

14.1272959	#N/A	chr7	28980050	28984050	Map4k1	+	G2
14.7314626	#N/A	chr10	122676762	122680762	Ppm1h	+	G2
15.8560327	#N/A	chr6	124710336	124714336	Phb2	+	G2
17.2692317	#N/A	chr9	106168928	106172928	Wdr82	+	G2
19.8391254	#N/A	chr2	71743616	71747616	Itga6	+	G2
21.1260669	#N/A	chr2	29122181	29126181	Setx	+	G2
21.2332921	#N/A	chr1	60975927	60979927	Icos	+	G2
25.361323	#N/A	chr1	36759798	36763798	Zap70	+	G2
27.3088805	#N/A	chr16	33249456	33253456	Snx4	+	G2
35.6994698	#N/A	chr8	88270403	88274403	Adcy7	+	G2
36.9088587	#N/A	chr9	21422908	21426908	Dnm2	+	G2
41.9364413	#N/A	chr10	127319964	127323964	Arhgap9	+	G2
56.6300413	#N/A	chr3	60470830	60474830	Mbnl1	+	G2
57.3680805	#N/A	chr9	44602892	44606892	Ddx6	+	G2
201.534632	#N/A	chr6	71320788	71324788	Cd8b1	+	G2
0.04814662	#N/A	chr1	106610945	106614945	Gm37053	-	G3
0.04201781	#N/A	chr1	136692210	136696210	Platr22	+	G3
0.06868768	#N/A	chr1	171915583	171919583	Gm37065	-	G3
0.03062871	#N/A	chr10	93827554	93831554	Usp44	+	G3
0.03559078	#N/A	chr11	88226937	88230937	Gm38534	+	G3
0.03900275	#N/A	chr11	117422162	117426162	Gm11732	+	G3
0.03645	#N/A	chr12	104108723	104112723	Serpina3a	+	G3
0.00706927	#N/A	chr13	43118838	43122838	Phactr1	+	G3
0.0265609	#N/A	chr13	84218109	84222109	A230107N01	-	G3
0.05308031	#N/A	chr13	90896581	90900581	Atp6ap1l	-	G3
0.09582904	#N/A	chr14	51769590	51773590	Ang4	-	G3
0.02394881	#N/A	chr16	45694553	45698553	Tmprss7	-	G3
0.05792822	#N/A	chr17	23641269	23645269	Mmp25	-	G3
0.07072469	#N/A	chr17	23782077	23786077	Flywch2	-	G3
0.01514889	#N/A	chr17	28860196	28864196	Pnpla1	+	G3
0.01922003	#N/A	chr18	37316380	37320380	Pcdhb5	+	G3
0.056619	#N/A	chr2	37327748	37331748	Olfr368	+	G3
0.04053867	#N/A	chr2	87146659	87150659	Olfr1111	-	G3
0.01035556	#N/A	chr2	88387248	88391248	Olfr1178	+	G3
0.08265546	#N/A	chr2	163085601	163089601	Gtsf1l	-	G3
0.04461116	#N/A	chr3	11840359	11844359	Gm10745	-	G3
0.03204849	#N/A	chr3	28888616	28892616	Gm1527	+	G3
0.03089713	#N/A	chr3	36502809	36506809	Gm11548	-	G3
0.00675913	#N/A	chr3	79682867	79686867	Rxfp1	-	G3
0.023308	#N/A	chr5	73402047	73406047	Cwh43	+	G3
0.06735848	#N/A	chr5	76818465	76822465	C530008M17	+	G3

0.04246424	#N/A	chr6	55170990	55174990	Inmt	-	G3
0.00474173	#N/A	chr6	119193492	119197492	Cacna1c	-	G3
0.05312181	#N/A	chr6	135306384	135310384	Pbp2	-	G3
0.02086695	#N/A	chr7	66729104	66733104	Cers3	+	G3
0.00902654	#N/A	chr7	103608351	103612351	Olfr620	-	G3
0.01532314	#N/A	chr8	41213978	41217978	Fgl1	-	G3
0.01261704	#N/A	chr9	14736733	14740733	Piwil4	-	G3
0.00747528	#N/A	chrX	7615387	7619387	Cacna1f	+	G3
0.02451039	#N/A	chrX	57039073	57043073	Brs3	+	G3
0.04150446	#N/A	chrX	102796367	102800367	Dmrtc1c1	+	G3

#N/A - not differentially expressed

Supporting Information for Fig. 7a - Heatmap representing overlap of differentially expressed genes between Dnmt3b$^{+/-}$ and Dnmt3a$^{\Delta/\Delta}$ PTCL when compared to control thymus

Gene ID	CD8	Dnmt3b$^{+/-}$	Dnmt3a$^{\Delta/\Delta}$
Sept10		24.7560096	22.8567991
1110002L01Rik		3.70136824	3.61612328
1190007I07Rik		6.19240616	3.82366551
1600014C10Rik		6.77679572	5.38146291
1700025G04Rik		3.53439597	7.3020288
1810009A15Rik		2.72186089	2.25124872
1810037I17Rik		2.38628812	2.00585631
2010008C14Rik		12.788302	10.0349303
2010016I18Rik		8.69760572	5.79122293
2310001H17Rik		9.20521758	8.6200189
2310009A05Rik		3.93916538	3.08920031
2310061I04Rik		2.62957202	2.45879511
2610035D17Rik		2.99698263	2.98781179
2610037D02Rik		11.0672691	6.61060027
2610306M01Rik		4.93025241	4.13751249
2810006K23Rik		5.16087941	3.79290117
3110062M04Rik		5.24229802	2.71228203
4632404H12Rik		8.59279017	4.92974748
4930427A07Rik		3.35760588	4.10249075
4931431C16Rik		4.6294588	4.00664095
4933404O12Rik		8.1003897	7.47604597
5730409E04Rik		35.2241632	20.9764925
5830468F06Rik		33.6220818	20.2802987
6030458C11Rik		4.2244451	4.43859178
6330409D20Rik		4.64964762	4.02950544
6530402F18Rik		3.74510286	2.70230393
9930111J21Rik1		2.86207718	2.62261281
9930111J21Rik2		4.62119551	4.5919534
A330040F15Rik		39.5241884	29.3791019
A430018G15Rik		9.83489329	13.6746773
A730017L22Rik		2.54970598	2.68490549
A930037H05Rik		7.70185384	5.39205116
Abcb10		6.40339961	3.72899261
Abi3		4.46323837	3.90306368
AC121965.1		7.20213212	3.49618467
AC130217.2		16.6004281	41.612354
AC131739.1		17.9685366	14.1963662

AC151730.3		95.5118811	130.869964
AC153495.1		9.80869615	12.6313838
AC165080.4		6.19341176	6.96105035
Acaca		2.23740882	3.06454515
Acot13		3.24829478	3.19277302
Acot7		5.19420921	3.37214453
Acsl5		2.50515508	2.03917854
Actb		5.45879983	3.28072262
Ada		4.29415265	4.06148967
Adamts13		128.43409	70.9592516
Adap1		9.6359469	5.79423458
Adarb2		27.3393841	35.9589565
Adcy1		107.561851	10.0552305
AI504432		3.94003237	3.77004412
AI506816		68.1895021	55.4245718
Aifm2		23.0851971	25.3859425
Akip1		4.88537387	3.00503908
Aldoc		17.1012895	12.7535788
Alg6		3.20783909	3.35092439
Alms1		3.59966012	3.67690835
Ampd2		2.78668378	2.37348221
Anapc13		3.6387677	2.82356459
Angptl2		16.9758711	22.8605894
Angptl4		5.13083428	4.05783075
Ankle1		32.2023907	25.0557603
Anln		26.2491726	28.2630605
Anxa9		9.39563863	7.92150317
Ap1s1		3.33264141	2.38511453
Apitd1		6.43986395	7.05129301
Apobec3		3.645037	4.01710036
Apobr		10.1724323	13.1764461
Apol10b		25.6905128	30.5508535
Aqp9		12.9042947	6.76348305
Arhgap11a		3.96990484	3.31508807
Arhgap19		6.2311208	5.28719025
Arhgap27os3		4.9077211	4.31594491
Arhgef10		5.79354292	4.84549088
Arhgef39		5.80461007	5.19229678
Armc7		3.8768882	2.63149608
Armt1		2.65803449	2.73050326
Arsb		15.2518871	15.3086721
Asf1b		4.640322	4.33873757
Aspm		13.5986902	13.8217297

Asrgl1		3.57574718	4.04060931
Atg4a		5.64040834	4.59895866
Atg9b		70.5158917	52.6522379
Atp11a		2.25273538	2.14681257
Atp1a2		29.6553097	26.7659155
Atp8b4		3.7438693	4.40685885
AU020206		8.62478613	8.31782341
Aunip		33.226376	30.3491313
Aurkb		24.4854601	20.5283952
AW112010		6.17481669	4.76791505
AW146154		3.3332576	3.52548896
AY074887		9.31034811	9.17038748
B230307C23Rik		4.63714755	4.53186776
B230354K17Rik		3.84182901	4.44199726
B4galt5		4.01614444	2.62734927
Bak1		3.32017555	2.57976856
Bard1		4.21973411	4.20187953
Bbs12		12.4029522	6.47811352
Bbs9		2.53123895	2.82161207
BC030336		5.73114336	4.13200822
BC030867		10.7704983	11.0027385
BC037034		4.78851353	3.01079719
BC051226		13.2832702	3.3550899
BC147527		9.98772317	6.06198097
Bcl2		3.38158373	4.31908102
Bcl2l1		2.6086143	2.74420789
Bcl2l12		2.85512422	2.42357644
Bfar		2.2447472	2.01585629
Birc5		17.6250951	14.3486077
Brca1		3.70841024	4.20732117
Brca2		3.50243169	4.37596702
Brd3		2.8686659	2.33807656
Bri3bp		3.41934291	2.08249443
Brip1		5.92963654	8.1422488
Brip1os		2.55727187	2.69099011
Bsn		21.4217267	17.3043079
Bspry		7.67792735	9.32025779
Bst2		14.3435969	3.1349705
Bub1		10.4995325	11.1644459
Bub1b		15.1733478	16.1402475
C030010L15Rik		4.76986008	4.7462586
C1qtnf6		17.4555569	14.3591608
C2		21.5027455	8.01958952

Cacna1a		19.2916863	9.13771375
Cad		2.4640217	2.46172523
Cap1		2.07623816	2.14355098
Car12		5.80606719	6.57306617
Casp7		4.47588815	3.22964164
Cbfa2t3		12.2148244	12.6369833
Cbx1		3.11155774	3.33855173
Cbx5		5.59957624	6.95586415
Ccdc136		10.0022962	14.535411
Ccdc18		11.6289442	12.111917
Cchcr1		3.21780257	3.29026345
Ccna2		8.13281369	8.04366201
Ccnb1		16.4749441	10.8207875
Ccnb1ip1		9.35293577	7.85810176
Ccnb2		17.2978157	16.3766405
Ccnd2		2.87217204	2.80453169
Ccne1		7.48990158	4.67285332
Ccng1		2.70055289	3.9063164
Ccr2		27.4371233	31.0337695
Ccr5		14.7198249	19.6341604
Cd22		10.2986239	11.4241779
Cd38		8.02546112	6.09808449
Cd48		2.88849746	2.00871361
Cd68		13.9483739	4.51330492
Cd79b		7.62974095	2.89915292
Cdc23		2.21224567	2.31180602
Cdc25c		20.7673894	17.9138279
Cdc45		5.44290277	4.84340909
Cdc6		14.7986156	17.2502331
Cdc7		2.57845054	2.17734697
Cdca2		9.26418017	10.5738261
Cdca3		7.60307132	5.88033259
Cdca5		5.23938493	4.80600396
Cdca7		10.6672682	11.2486087
Cdca7l		2.6120577	2.88769347
Cdca8		9.8202941	7.40060649
Cdk1		16.2589244	12.3941694
Cdk2		2.28572299	2.48106959
Cdk4		2.30798525	2.20907297
Cdkn2c		10.2519627	3.92208697
Cenpe		11.4607793	11.1854776
Cenpf		21.1150915	21.2367768
Cenph		7.82554851	6.97076305

Cenpi	10.3883081	8.78345927
Cenpm	15.484633	8.32579117
Cenpn	5.39788818	4.85054564
Cep55	6.63678711	6.65496662
Cep83os	2.46970364	2.56484351
Chaf1a	6.34120524	6.75467673
Chek1	3.74000183	6.27034999
Chek2	10.6565328	8.74212464
Chst11	3.04952186	2.67648722
Chtf18	3.23117281	3.60850134
Cipc	2.08924447	2.78318123
Cisd1	5.4309623	3.27445857
Cit	11.4699036	8.5359963
Ckap2l	11.1259068	9.3545599
Cklf	2.5519399	2.1205454
Cks1b	8.24787155	5.78805511
Cldnd2	11.7701129	8.15007451
Clec2d	12.161425	4.15517882
Cln3	4.01944417	5.26241752
Clspn	10.7195957	9.83079508
Clu	536.598486	32.0441168
Cmc4	3.63809802	2.96971668
Cmpk2	29.8044133	11.5450963
Cnp	3.3385212	2.37383251
Coa3	3.96531956	3.53648885
Coasy	3.13452604	2.54267605
Col15a1	85.6987612	68.5445967
Col4a2	353.881968	20.8258307
Coro2a	3.8397816	4.53782878
Cox7b	5.81068314	2.76453664
Cpne3	2.94487821	2.96453053
Cpox	2.64212791	2.47244372
Cpsf2	2.54564379	2.47119326
Crk	2.29529884	2.3112839
Crtap	5.14264343	2.91833632
Crybg3	14.8692338	4.45592526
Csf2	32.7348441	65.5625058
Cstf2	2.26142933	2.38071374
Ctrl	107.265457	17.0780847
Cxcr3	8.08902529	5.08543805
Cyb5b	2.65299898	2.34637193
Cyb5d2	3.04492813	3.21251248
D030028A08Rik	2.92597056	3.74850954

D11Wsu47e		4.30443717	3.72195238
D630008O14Rik		3.49774708	4.38890287
Dars2		2.55389327	2.78851958
Dbi		5.09620824	3.65937358
Dcaf17		2.08252196	2.29162712
Dcp2		2.61794821	2.46094936
Dctd		6.7346433	6.97478619
Dctpp1		6.85728138	5.13155354
Dcun1d2		2.08862044	2.05246982
Dcun1d3		2.91538369	2.07833579
Ddit4		23.5942498	8.7154736
Ddx58		2.61098322	2.95172037
Depdc1a		10.7209336	11.927612
Depdc1b		5.12227351	4.57075585
Dhfr		7.38196859	6.17155559
Dhrs1		2.56021961	2.14665343
Dhrs7		2.66951007	2.20570242
Dhx58		14.285132	9.53950721
Dhx58os		17.8948287	9.56719029
Diaph3		8.90829537	9.22777699
Dkc1		2.86774167	2.73612583
Dleu7		69.8104896	81.7548186
Dlg4		3.32486937	2.29467708
Dlgap5		6.44132912	5.51087844
Dna2		8.75230038	12.9091045
Dnaja3		2.80255519	2.48214343
Dnmt1		2.86567002	3.31499223
Dpagt1		3.25918305	3.34887133
Drc1		5.86839406	4.33270235
Dscc1		3.63313145	4.40668592
Dscr3		2.69875736	2.37027068
Dtx3l		5.84995588	5.63658035
Dtymk		2.67299513	2.31110389
Dynll2		4.26477383	3.00808787
Dzip3		3.06358864	3.22105893
E230001N04Rik		6.98555326	4.99076663
E2f1		5.36671122	4.13478986
E2f2		5.8881663	5.04537231
E2f3		3.77817672	3.7167786
E2f7		8.69150192	7.61263076
E2f8		14.9432706	12.8816593
Ect2		6.23410205	7.59925005
Edaradd		11.2398551	12.6016327

Eef1akmt1	3.87012848	3.07970145
Eif2ak2	12.5422012	11.8797183
Eldr	42.7560184	66.0909346
Elovl6	6.33849357	8.77793166
Eno1	2.51434647	2.59481665
Entpd1	9.27704733	8.02194361
Eomes	3.72183195	6.64347083
Epsti1	7.12392697	3.4110255
Ercc6l	4.49172712	3.79095215
Erg28	3.38808272	2.7395153
Ermp1	2.75435218	2.33325501
Esco2	14.690614	14.2900067
Espl1	38.791851	35.9074518
Etfb	3.00224411	2.76284102
Evi2	2.86696186	2.68292598
Evi2b	2.87081968	2.75066397
Exo1	3.46835878	3.79171318
Exoc8	5.09525738	3.31656053
Ezh2	4.08754206	3.75827074
F2rl2	19.0446583	13.016476
F730043M19Rik	16.8211336	5.10862398
Fadd	4.39246157	2.49715892
Fads6	60.2667175	64.876903
Fah	6.27530828	4.70957554
Fam111a	7.54432523	5.53652373
Fam122b	2.37532351	2.54211153
Fam136a	3.0932809	2.8759221
Fam173b	3.45722395	3.05180501
Fam185a	3.567605	4.65813252
Fam19a3	18.3036795	7.12298108
Fam212b	6.23602122	6.05233853
Fam3b	25.9722291	22.789305
Fam57a	4.83809466	5.32426912
Fam57b	13.9024955	3.25772114
Fam72a	7.06869792	4.47534071
Fanca	3.23618114	2.4198418
Fancd2	9.28961006	10.3917267
Fancg	2.13962569	2.05496469
Fasn	2.5109287	2.87309104
Fbxl18	4.04445774	2.64190789
Fbxl8	3.8607755	2.26503953
Fbxo48	4.76066942	5.48760704
Fcgrt	4.31108479	3.50279487

Ffar1		23.5117397	11.3374771
Fgl2		38.3558527	32.0654068
Fhl2		12.7677992	8.6505593
Fignl1		6.97100294	8.75455064
Fkbp10		8.10003011	24.941818
Fn1		367.721227	61.1619711
Fn3krp		4.23782354	4.96018769
Foxk2		2.12648405	2.20367606
Fsbp		9.69913493	9.22794446
Fut7		24.8001916	7.82299543
G0s2		14.5005083	13.3356144
Galk1		3.61606489	3.22529554
Gapdh		2.80152387	2.43189058
Gart		2.80832836	2.30772623
Gbp10		6.88712904	6.33925162
Gbp4		2.5573103	2.1041997
Gbp5		7.90489229	4.16781885
Gbp6		5.67815299	4.69466694
Gbp7		9.29966702	6.56489408
Gbp8		39.3405964	27.9502844
Gdf11		3.84882269	5.15135582
Gemin4		6.6877309	7.82478592
Gen1		4.8098947	5.89875147
Gimap4		2.43284418	3.36422477
Gimap7		2.66479338	3.18014288
Gins1		8.84159054	7.795791
Glod4		2.70611223	3.34235834
Gm10260		61.289553	57.614943
Gm10459		5.27255564	2.80211312
Gm10602		4.781672	3.16693964
Gm10684		4.50395466	5.58382309
Gm11508		9.28066862	4.96528396
Gm11626		4.194769	2.87307194
Gm11695		13.3462772	6.94778876
Gm12185		10.2009841	9.6696233
Gm12216		5.47470975	3.18387134
Gm12223		11.8928863	18.8029594
Gm12253		49.958242	23.8834655
Gm12500		3.54171954	3.85682472
Gm13091		8.45624615	6.02829589
Gm14029		3.26552956	2.52166323
Gm15232		4.43553875	5.96418937
Gm15545		2.58078183	2.40742115

Gm15629		3.45218593	2.87985113
Gm16085		15.144371	20.0536576
Gm16163		5.86756121	2.58452234
Gm16754		5.94740884	3.82896548
Gm17233		10.2711001	6.44368843
Gm17655		18.9859462	15.6994104
Gm17767		204.331487	213.652994
Gm19705		8.26421885	8.3615801
Gm20496		4.38128824	2.71614692
Gm20531		4.33345535	4.29250532
Gm20559		4.91905749	4.41096115
Gm20663		55.9324864	106.471708
Gm20667		15.9970428	30.6462741
Gm20708		2.77451246	3.09013408
Gm21970		3.16353041	3.08125595
Gm26517		3.83295123	3.57768699
Gm26527		13.5389582	15.5621446
Gm26619		61.2908882	57.614958
Gm26637		31.5395861	28.9572337
Gm26762		2.64986339	2.35563583
Gm26771		9.22133733	4.43493172
Gm26825		13.777528	13.0044285
Gm2788		18.7771362	8.79412913
Gm28044		4.76798498	4.08943925
Gm28053		2.13951275	2.04861692
Gm28177		5.43941756	5.18984903
Gm28778		2.95555785	2.55848569
Gm29439		8.39417876	11.0460174
Gm3055		9.85270163	7.2988966
Gm32856		10.3312186	9.88511899
Gm33460		3.63545479	5.73569654
Gm35037		36.207368	24.2813679
Gm35315		5.28707347	5.24655483
Gm36936		27.394641	77.4547436
Gm37233		13.9511328	9.89994128
Gm37422		2.40384016	2.1282906
Gm37787		7.40179745	6.89084893
Gm37844		6.52620305	5.27691632
Gm38244		5.12971145	3.99316025
Gm38357		8.60763108	7.48722857
Gm38979		65.7666511	64.6291574
Gm4070		26.158879	35.728629
Gm42547		3.28715592	3.23623561

Gm42742	3.16500081	2.18260522
Gm43059	5.97266994	18.9201619
Gm43197	8.01031664	8.21137909
Gm43198	4.24891118	4.1490423
Gm43302	5.04372792	4.44374248
Gm43791	16.1481628	17.0283078
Gm44148	26.3052122	14.3681479
Gm44166	13.9466336	23.8315247
Gm44175	5.83121083	10.5185765
Gm44198	11.3495124	12.8648683
Gm44985	18.6941213	12.6939906
Gm45193	3.39383829	2.66881917
Gm45233	2.94445645	2.82047406
Gm45606	2.36276811	2.98360155
Gm45631	4.80389875	3.60823702
Gm45867	2.39164467	3.28678839
Gm45884	5.94663077	5.66175519
Gm45890	5.82424396	4.05707848
Gm4737	18.8030113	17.9522125
Gm6594	45.4944907	43.3872653
Gm6685	21.9360646	21.3594884
Gng2	2.82232868	3.10476727
Gngt2	5.05588953	3.69085276
Gpr155	4.2289014	5.4526187
Gpsm2	5.1884442	3.67836647
Gstp2	73.7317028	30.6228583
Gstp3	4.80469986	2.87212785
Gtse1	7.74620272	6.68901857
Gusb	3.6136987	2.44882424
Gvin1	7.15151507	9.18104561
Gyg	3.70275857	4.29826803
Gzmb	101.292458	21.6723732
H2-T24	16.830426	18.1295561
H2-T3	3.33084032	3.14714927
Hba-a2	160.456884	112.297929
Hdac11	5.91509047	5.42197328
Hells	3.249013	4.2021195
Herc6	3.40017067	3.23931645
Higd1a	2.47054392	2.83234663
Hikeshi	4.00681199	3.35798782
Hip1	8.30013995	3.67693985
Hmga1-rs1	38.3333963	25.9237948
Hmmr	21.4353867	26.007028

Hn1l		3.03501635	4.12427405
Hnrnpll		2.4788099	2.86543728
Hopxos		8.61965389	4.47577223
Hsd11b1		2.45564859	2.43932136
Hsh2d		3.63803587	2.87173811
Ifi206		6.45347526	7.49860894
Ifi214		3.68115992	3.48949539
Ifi27l2a		10.0859986	4.89550491
Ifi35		2.8016426	2.30862242
Ifi47		4.44695137	4.71041873
Ifih1		4.63814249	3.79341019
Ifit1		21.0952781	10.7059073
Ifit1bl1		14.1235352	8.28932178
Ifit1bl2		24.3209223	18.5098968
Ifit2		8.73009471	5.97144251
Ifit3		19.4501502	15.5301291
Ifit3b		68.9082126	52.2800081
Ifnar2		3.29387638	2.59162541
Ifrd2		2.36413733	2.13612304
Ift52		2.35545077	2.19862992
Igf2bp2		32.033443	6.93585922
Igsf23		63.7522806	66.3204825
Igtp		9.75226854	7.2906941
Il10ra		9.17656464	3.77853049
Il10rb		3.61349358	3.40366395
Il12rb1		12.5948647	8.36541259
Il15ra		5.10867221	3.74525114
Il18r1		3.78900313	3.74019386
Il1rl1		141.659209	11.8262899
Impa1		2.57400881	2.93126039
Impa2		6.44969079	5.31224428
Incenp		3.69183412	2.93782717
Inppl1		2.57718488	2.04904598
Insig2		3.56523445	2.42084529
Ipp		3.20509689	2.39692492
Iqcc		2.68729744	2.79497245
Iqgap3		17.9609223	10.4921969
Irf7		9.01440834	3.74190605
Irgm1		5.05739683	6.01908102
Irgm2		16.368464	12.7881027
Isg15		55.0391574	11.3505097
Islr		22.6669486	304.3433
Isoc2b		5.80901563	4.98074782

Itgb3		11.8689944	15.8047331
Itih5		15.7104036	6.15543449
Itpripl2		12.903973	10.7391166
Jaml		5.36780874	6.53121992
Jup		6.78217544	8.20015653
Kbtbd11		6.7667253	6.22675782
Kcna3		4.80494933	4.40393349
Kcnf1		14.1526659	15.2951212
Kcnip3		8.58839394	9.19190111
Kcnk5		8.61985096	5.35254303
Kcnk6		6.97640484	5.72339961
Keap1		4.75238735	3.19034945
Kif11		8.60653287	9.7887907
Kif14		14.8974755	15.8251002
Kif15		10.5563533	14.5554584
Kif18b		23.9141067	23.9636911
Kif20a		4.77335512	4.20337743
Kif22		5.86530195	4.82846657
Kif2c		28.9255889	21.5040423
Kif4		12.2583541	9.93368641
Kifc1		16.3616875	14.799634
Klhl20		2.73199683	3.25421605
Klhl42		3.77839328	3.16431599
Knl1		15.174741	16.9522168
Knstrn		3.48490134	2.70499177
Kntc1		20.1986123	15.8909582
Kpna2		3.37663543	4.10791696
Lage3		4.1651383	3.05796843
Lair1		4.40835238	5.6867643
Lamp2		5.36496469	3.45948967
Lamtor2		4.9158329	2.22584686
Lamtor4		6.08365386	3.02641789
Lcp1		2.12279418	2.00613003
Ldha		2.60732768	2.2305686
Lgals3bp		8.23738025	5.911526
Lgalsl		3.6418112	6.49389488
Lhx2		31.4057856	24.7128662
Lig1		4.43393569	4.04068434
Lmnb1		3.58766074	3.30288648
Lockd		26.4301654	18.039211
Lpar5		12.1531603	9.75980975
Lpgat1		3.00417103	2.92436539
Lrr1		33.4859215	42.5713536

Lrrc75a		15.241051	49.5670055
Lrrk1		5.22016267	9.09756366
Lsm10		4.64393391	2.51776622
Lta4h		2.64153633	2.48462257
Ly6a		131.717456	51.9067988
Ly6c1		67.3840863	13.6957737
Ly6e		4.68386732	4.40310607
Lyrm9		4.94315743	4.24446702
Lyst		4.70294403	7.97943955
Mad2l1		2.55015804	2.42090185
Mansc1		8.94990735	9.42408048
Map7d1		2.29234345	2.18991528
Mastl		4.48873498	3.59367968
Mavs		3.63403168	3.07349092
Mb21d1		2.69328518	3.40176104
Mblac2		2.48347686	2.14345689
Mcee		3.85333696	2.98850139
Mcm10		20.4235776	18.5502261
Mcm2		3.96422127	3.6595361
Mcm6		2.80362793	2.90319944
Melk		10.1325955	8.05870524
Mettl21a		3.50832295	2.68531856
Mettl7a1		14.8159277	4.67635042
Mfsd10		2.82218527	2.1469539
Mgat1		5.33175862	4.37216032
Mgme1		2.45692237	2.51154898
Mif		4.47133808	2.62183916
Mis12		3.10454279	3.6596493
Mis18bp1		4.85761382	5.13451334
Mitd1		3.9697471	4.18302643
Mki67		16.2615408	16.3854896
Mlkl		33.6388212	45.0497372
Mmachc		7.49567015	6.79639138
Mmgt2		4.00129448	4.00386002
Mov10		4.32583382	3.38623411
Mplkip		3.23735201	2.96989308
Mpp1		3.2747679	2.65505475
Mrpl14		4.70930049	3.27495891
Mrpl34		4.17412533	2.73674453
Mrpl36		4.12820403	2.34063754
Mrpl52		5.23257065	2.12612241
Mrps21		4.93109624	2.77788898
Ms4a4a		37.5258744	39.052735

Ms4a4b		8.575902	12.8002573
Ms4a4c		8.21066226	8.50577667
Ms4a6b		2.23784044	2.08885899
Ms4a6d		55.4150847	40.3196042
Mtch1		3.44000402	3.06419275
Mtfr2		10.274892	7.49747513
Mthfd1		2.0942546	2.23848629
Mthfsd		2.48499853	2.0340214
Mut		3.99871219	4.27325207
Mx1		24.9242497	11.1386468
Mxd3		6.44169366	4.05030617
Mybl1		5.89811118	6.31205858
Mybl2		4.51655514	3.64823487
Myc		3.25287903	3.32566511
Myo5a		3.02160735	2.77446322
Myo6		63.9452248	8.09596366
Naa10		4.13295513	2.68654722
Naa38		6.43473965	3.75247024
Nabp1		2.65713134	2.53261318
Naglu		4.31402463	2.80524448
Naip2		4.31067353	5.20692198
Ncapd2		4.30999885	4.17882921
Ncapg		10.0995514	11.1284418
Ncapg2		6.09743719	6.67624079
Ncaph		7.8911532	6.9715164
Ncbp2		2.36407065	2.17861596
Nccrp1		9.66944498	5.48520964
Ncoa7		2.30790971	2.92421396
Ndufb3		3.1614735	2.34746434
Ndufs5		6.18281715	3.40949834
Nedd1		2.18390438	2.36303907
Nefh		3.52201527	5.3107908
Neil3		36.7418732	31.6249068
Nek2		12.1903688	10.1940432
Neurl1b		21.6577387	11.9958492
Nfic		2.56630188	2.16110791
Nid1		118.030486	14.4059088
Nkg7		2.61168005	2.41321353
Nlgn2		11.8756426	5.86070026
Nln		2.13522066	2.22105012
Nlrp10		34.9148803	32.3235129
Nlrx1		5.8948931	5.31990726
Nmral1		12.9445616	5.30544826

Nop10		4.08158281	4.03621424
Npcd		10.3733593	7.37613736
Nr1h5		7.54372473	13.6616066
Nrm		3.05124726	2.39628985
Nrp2		23.4880275	14.0569583
Nsd2		4.47674288	4.6225958
Nsl1		4.72442969	3.41103503
Nudcd1		2.95755151	3.50631898
Nudt16		4.02588108	2.59111586
Nudt19		2.40512599	2.18334433
Nudt3		2.3459135	2.20567128
Nudt7		3.98650721	4.44183637
Nudt8		5.51598217	2.72375322
Nuf2		21.9621274	19.9649867
Nusap1		13.3181202	14.3033197
Nutf2		2.84715428	2.17533663
Oas1a		29.0573355	17.1484209
Oas1g		453.687516	392.999824
Oas2		44.5221797	24.5630475
Oas3		21.7429099	11.975787
Oasl2		27.9899959	12.4877088
Ociad2		10.9329978	20.3089237
Oip5		9.43814423	6.09729646
Olfr524		11.9501629	30.7863919
Olfr56		5.20915354	6.0850299
Oma1		2.40872349	2.59974619
Orai3		5.05145976	2.88615708
Osbpl3		9.63541374	16.2350809
Oxsm		4.5196292	3.24132425
P2rx7		17.9901194	6.38590616
Pagr1a		3.12851754	2.32634384
Pald1		7.25207086	4.8107181
Pam16		3.80872846	2.4079295
Papola		2.15114344	2.36513815
Parp11		6.60863536	4.55843172
Parp12		8.42489535	4.69494819
Parp14		3.46134788	2.80482296
Parp9		5.07326381	4.34298928
Parpbp		13.1734204	11.7653578
Pask		6.18771026	5.34104208
Pbk		121.176218	69.6136082
Pclaf		23.6715068	23.4091436
Pcsk4		7.85336454	6.8590961

Pctp		6.52925689	9.54227043
Pcyox1		4.26272537	2.55032881
Pdf		3.05854017	2.12681757
Pdia4		2.99699555	2.01254436
Pfas		2.37980098	2.97743851
Pfdn1		2.54980904	2.26379313
Pfdn6		3.38954844	2.73057568
Pfn1		2.96596814	2.15390359
Pgam1		2.73511287	2.42789784
Pgk1		2.93321503	2.30742078
Pglyrp2		3.65717107	3.12946051
Pgpep1l		7.60835502	11.0476742
Phf11a		4.34970723	2.98376374
Phf11b		3.71275754	2.60751659
Phf11c		2.83878606	2.58406954
Phf19		5.97963505	6.39751747
Phgdh		2.68697932	2.05324394
Phlda3		18.4256316	14.8700155
Pidd1		3.18087305	2.99982907
Pif1		35.5476121	34.3617597
Pigc		2.84163619	2.00148039
Pigf		3.16182534	3.51576983
Pigg		5.95876832	4.94645231
Pigw		4.89856938	3.46797838
Pilrb1		111.561532	39.543475
Pim2		3.52663428	4.385508
Pimreg		117.910226	93.0779779
Pkig		5.47697043	3.33597817
Pkmyt1		3.70420763	3.25095319
Pla2g15		7.60799066	3.87629567
Pla2g16		2.748683	2.09986603
Pla2g4c		21.3080066	26.4549703
Plac8		48.519287	22.1063728
Plekhg3		3.48047486	4.82886922
Plin3		6.15705269	4.78762137
Plk1		11.5635598	8.08590478
Plxnc1		3.18000602	2.17791561
Pml		4.06223882	4.0486358
Pola1		3.85163167	3.40441425
Pole		4.15449572	4.04327867
Polh		2.80150183	3.49837051
Polq		2.49892269	2.9553834
Pop1		3.31077382	3.16119635

Ppa1		5.05115111	3.43112797
Ppil1		7.21512754	5.62529483
Ppp1r18os		5.91921102	3.33091316
Pqlc3		2.73452128	2.06968372
Prc1		8.748787	7.74306217
Prelid2		4.00473633	4.30481849
Pros1		33.8183958	4.27887925
Prr11		13.7235365	12.4587355
Prrc1		2.29499842	2.48625656
Psat1		2.84002447	2.89154934
Psmb10		3.52673734	2.68066073
Psmb8		3.70892485	2.71687357
Psmb9		5.37426587	4.05149954
Psme1		2.20342976	2.15351037
Psme2b		7.04964995	6.64798589
Psph		2.26339757	2.03376568
Psrc1		11.6232829	6.51613807
Ptger3		131.874239	65.9576312
Ptp4a3		2.50310332	3.03014197
Ptpn6		3.22114771	3.71554784
Pvrig		17.0508002	26.5525665
Pxmp4		4.39484206	4.2215254
Pycrl		3.32158778	2.93974276
Rab27a		3.44264065	4.2999328
Rabepk		3.91961065	2.42303506
Rad1		3.17425179	2.88131126
Rad51		8.16727011	6.70462282
Rad51ap1		6.4470278	4.87808542
Rad54b		12.4495821	13.1090087
Rai14		39.3896664	21.8583594
Rapgefl1		9.60078838	21.0601015
Rassf4		6.47534244	9.68031399
Rbm10		2.95659266	2.24605675
Rbm43		2.90210664	2.44553252
Rcbtb2		8.00276178	6.56080412
Rcc1		2.67406051	2.31853898
Rcn1		3.91833795	2.23685766
Reep5		3.38112589	3.34418981
Rgs12		9.3372866	7.50832169
Rhoq		3.30106401	4.36596995
Rinl		2.83711098	2.3836393
Riox2		2.62092011	3.21283517
Rmi2		17.9665193	12.3825303

Rnasel	5.4078331	3.17803566
Rnf135	3.73630715	4.262818
Rnf157	5.70466621	6.3619481
Rnf213	10.4056129	6.64985249
Rnf41	3.36217579	2.15418914
Rnf5	3.16655274	2.53346732
Rpl38	6.20146982	2.75806255
Rps2-ps6	360.263741	246.990165
Rrm2	18.3600767	13.8426394
Rrp1b	2.07827404	2.18227515
Rsu1	2.13007494	2.0616767
Rtp4	21.2907981	21.1525603
Ruvbl1	2.38267256	2.00973334
Ruvbl2	2.71581757	2.45300685
Ryr1	16.0480601	13.4475809
S100a10	3.03711791	2.22761503
Samd3	5.4597839	8.5578069
Samd9l	4.4896387	4.08304287
Sash3	2.90981599	2.06013533
Scd2	4.21155853	6.54078997
Scrn3	4.37200896	5.4921814
Sdc3	12.5020104	4.1365353
Sdf2l1	6.25955799	3.57665479
Sdhaf2	2.04877682	2.14674004
Sec61g	3.70002966	2.21038469
Sema6b	40.5167458	14.6930899
Sema7a	21.8163115	7.27828824
Senp8	6.92357134	7.08707015
Serpinb9	3.17679914	2.10320906
Setbp1	9.76464548	8.02077135
Sfrp2	23.6934994	7.92507461
Sfxn1	2.10134457	2.53650763
Sgo1	8.68409876	8.72453118
Sgo2a	3.58944174	3.67059299
Sh3bgrl	3.06193811	2.54334148
Sh3bp2	7.40733876	4.05555402
Sh3pxd2b	4.76934303	7.89784416
Shcbp1	10.4690126	10.1604827
Shmt1	2.06650367	2.65880505
Ska1	7.04466852	5.96934737
Ska2	5.49214982	6.59351144
Sla	3.13374627	3.52631308
Slamf7	5.4498932	5.5187749

Slc16a13		3.71434032	4.34506421
Slc16a5		5.5882451	5.62806182
Slc1a5		2.6735017	2.05063616
Slc29a1		2.51440541	2.03027159
Slc33a1		2.33047971	2.11283366
Slc35a2		3.38703467	2.02854365
Slc35b4		2.17220074	2.2931689
Slc35c1		2.99675412	2.1234149
Slc39a6		2.78251198	2.34156989
Slc43a1		7.42249547	4.64748505
Slc9a5		4.07602687	5.94771514
Slfn1		3.38165296	2.70870973
Slfn3		7.73743643	6.00757747
Slfn8		4.48239401	5.04543617
Slfn9		5.16043636	3.95482168
Smc2		3.34423559	3.71706218
Smim11		3.17630134	2.13547753
Smim24		10.6522524	5.16068896
Smim4		3.94531722	2.17156859
Smpdl3b		6.65078906	6.28710098
Smyd2		2.60679095	2.77566686
Snx1		2.21759709	2.43058181
Socs2		72.5695666	9.50022017
Sord		3.32240274	3.097907
Spag5		8.4282753	9.32471981
Sparc		384.770888	29.1552644
Spc24		9.96977538	10.3718592
Spc25		12.1908844	12.9871816
Spin2c		12.171659	14.3832336
Srm		2.31161333	2.46324715
Srp54a		11.9868959	17.0285896
Srsf1		2.05490885	2.30694697
Stat1		9.28706094	8.09461935
Stat2		4.17435987	2.50604287
Stc2		6.03610708	5.86427737
Stil		4.98552185	6.11927696
Stmn1		4.930157	7.23334971
Suclg1		2.26072532	2.19074124
Sumo2		2.02261301	2.08574753
Susd1		3.99231826	6.6130261
Swi5		3.10227217	2.10192805
Syce2		4.07713405	3.95349563
Syne2		2.00035871	2.01392178

Syngr2	4.23017306	2.09777552
Synj2bp	2.34690515	2.44467721
Tacc3	3.9196404	3.38087191
Tap1	2.74342782	2.39655864
Tapbpl	3.59960038	2.91776671
Tbc1d10c	2.59591038	2.01750959
Tbc1d31	3.32383323	3.91955399
Tbkbp1	6.72079975	9.71836555
Tbl2	2.51173332	2.42921256
Tbxa2r	7.89137311	3.96549601
Tcf19	9.08553493	8.29178713
Tec	2.10778759	2.2250749
Tefm	4.86863207	3.49560325
Tex13c2	31.3318578	31.243125
Tfap4	2.93566307	2.52529077
Tfdp1	4.68094175	5.05538721
Tgtp1	19.243178	19.9343495
Tgtp2	10.2378277	10.7966387
Thap2	4.89895984	5.03471613
Themis2	9.48493515	6.59753003
Thyn1	2.10855815	2.41383269
Ticrr	4.85485191	5.49112966
Timm8a1	3.34814949	2.47320852
Timmdc1	2.72300297	2.09665043
Tipin	2.31500286	2.80227383
Tk1	9.79760815	9.49903495
Tkt	2.9177213	2.08394929
Tlr7	18.8948989	24.7439462
Tm6sf1	2.14488951	2.57540944
Tmco1	3.40817551	2.39132371
Tmed3	2.69617294	2.1619879
Tmem106a	9.63800275	7.28911647
Tmem126b	2.77034873	2.43851865
Tmem140	5.59910943	3.03049289
Tmem156	2.65339731	2.69062773
Tmem18	2.77844973	2.74590332
Tmem181a	4.76334566	5.38875969
Tmem229b	2.796567	2.17426076
Tmem256	4.3099238	3.68196112
Tmem261	4.76263397	2.36970083
Tmem37	12.6501055	8.19498131
Tmsb4x	2.68984023	2.74064799
Tnfaip8	2.01619308	2.26576255

Tnfsf10	5.90662929	7.00168967
Tnfsf14	6.88178921	6.88102886
Tnfsf8	4.22692953	4.76268637
Top2a	11.4460429	15.0608299
Tor4a	4.99527822	2.79532751
Tpi1	8.97503368	6.61061519
Tpx2	14.0073851	13.5024625
Trim12c	2.85995536	2.9033681
Trim14	3.48750751	2.3958153
Trim30a	3.42911676	3.55482487
Trim34a	3.71786135	4.2578059
Trim34b	4.09865471	4.26595063
Trim37	2.33566057	3.51805076
Trim46	13.4629773	9.70614031
Trim5	20.8740644	37.2024416
Trim56	3.12903416	2.48921065
Trim65	3.22112141	2.83996931
Trip13	7.6600759	6.62912536
Trmt1l	2.16720057	2.12112772
Troap	29.9561005	24.329885
Tsfm	3.75273947	2.92705589
Ttc16	6.4683439	6.21783752
Ttc39c	6.94655931	4.06578417
Ttc9c	3.58791773	3.2558964
Ttf2	2.86474547	2.75878113
Ttk	19.5701907	22.6012288
Tuba1b	2.86338221	2.65709738
Tufm	2.41664404	2.03243252
Twf2	3.14772581	2.10242488
Txn1	3.14350351	2.6558097
Txndc5	2.59997723	3.22588805
Uba1	3.50033245	2.50156901
Ube2c	20.1599464	12.6871677
Ufsp2	2.53255699	2.62727767
Uhrf1	11.0375752	9.78027075
Ulbp1	10.06815	7.21532211
Unc93b1	12.0330362	3.09923697
Uprt	3.11196522	3.74136601
Uqcc2	5.78796009	4.21915622
Uqcr11	6.03509343	2.34452268
Usp18	53.6829927	31.818178
Vdac1	2.22163118	2.10280867
Vkorc1l1	2.55821585	2.36155068

Vwa5a		4.19014851	5.5154281
Wdcp		2.59435579	2.77833187
Wdr1		2.24488527	2.00165186
Wdr31		66.9812957	99.9671205
Wdr76		3.14568851	3.57454476
Wdr86		9.64336903	16.5324083
Wdr95		5.65897493	3.32279315
Wee1		4.65839373	5.17763888
Wnt8a		60.5481087	82.7173193
Xaf1		10.9922077	11.835986
Xkr5		27.1472123	18.8064494
Xkr8		13.1257822	9.50876283
Xrcc6		2.58216403	3.49235143
Ywhag		4.45793555	3.38584771
Zbp1		12.4150546	6.85234614
Zbtb26		6.49700695	5.56859746
Zcchc18		7.25664985	8.26696506
Zdhhc24		4.15283944	3.06728848
Zfp113		2.5110754	2.43290421
Zfp217		2.77671866	2.54829085
Zfp3		14.3333239	9.14371206
Zfp324		6.61503676	4.78890212
Zfp329		5.06940471	4.65911602
Zfp365		5.72580644	5.88740638
Zfp397		2.71616175	2.29815741
Zfp41		5.15475576	5.00583223
Zfp458		3.49566394	3.41279178
Zfp459		3.28754262	2.88823316
Zfp626		3.11345528	2.72918721
Zfp7		2.34473236	2.92209723
Zfp709		2.76801989	2.86953686
Zfp738		4.2845265	3.70796906
Zfp759		3.49769341	3.53788836
Zfp790		3.01783222	3.38459324
Zfp933		2.35071366	2.47360958
Zfp934		3.62348751	3.12474369
Zfp953		2.48405078	2.42149661
Zfp958		2.97676464	3.32354729
Zkscan3		2.8609235	2.91738454
Zkscan7		2.734448	3.25928614
Zkscan8		3.1081479	3.27234234
Zmat3		4.14138007	4.50206452
1-Mar		6.17432658	

8-Mar		18.5913666	
3-Sep		14.7751103	
8-Sep		14.6150765	
9-Sep		2.68939287	1.87453272
0610009B22Rik		5.06752542	
0610038B21Rik		10.4839922	
0610039K10Rik		3.71607761	
1110004E09Rik		2.80565378	
1500009L16Rik		13.2825558	
1700030C12Rik		8.29999716	
1700066B19Rik		21.6189051	
1700071M16Rik		21.1421997	
1700123M08Rik		11.9471968	
1810030O07Rik		2.27727734	
2010107G23Rik		14.364305	
2010315B03Rik		2.80395732	
2310010J17Rik		5.52837513	
2500002B13Rik		10.4028936	
2600006K01Rik		8.9670057	
2810001G20Rik		3.7463498	
2810414N06Rik		8.66812677	
2900041M22Rik		8.782897	
4931406C07Rik		2.59334956	
5830432E09Rik		3.36929683	
5830454E08Rik		11.5046184	
6330408A02Rik		2.62629133	
6330537M06Rik		5.29092159	
6430550D23Rik		2.60679702	
9330175E14Rik		5.00566459	
9430064I24Rik		5.28137888	
9630013D21Rik		25.0098033	
A430046D13Rik		5.83889318	
A430057M04Rik		2.96821192	
A430110L20Rik		13.6625655	
A930029G22Rik		4.15225259	
Aaas		2.17744117	
Aacs		2.61509289	
Aarsd1		2.197345	
Abca9		19.1071847	
Abcd1		3.90390572	
AC122317.1		2.4192198	
AC124577.4		5.44728416	
AC126459.1		27.1101082	

AC126459.3		25.832748	
AC133650.1		3.61591926	
AC142191.1		11.6923277	
AC152827.2		6.66334027	
AC153912.2		2.89803505	
AC158605.3		3.9512116	
AC159264.1		16.9628201	
AC238811.2		19.3158661	
Acaa1a		2.13008628	
Acox3		2.58851799	1.89466217
Adam15		3.29370674	
Adamdec1		23.0166119	
Adat3		3.36338496	1.86737974
Adcy9		24.2776512	
Adgre1		60.4053639	
Adgrf5		21.8977358	
Adora3		11.7148547	
Adprh		2.21517831	
Aebp1		19.4385205	
Aen		2.22093004	
Agpat3		2.015905	1.80490481
Ahsa1		2.17751517	
AI467606		3.41414382	
Aif1		16.230708	
Aifm1		2.32372807	
AK157302		11.1963495	
AL513022.2		34.614562	
AL591582.1		6.18218787	
Alad		3.99940136	
Aldh18a1		2.25241796	1.80977932
Aldh1a2		44.4983786	
Aldh2		10.283441	
Aldh3b1		12.1966666	
Alg2		2.54372468	
Alg3		2.45080802	
Alox15		32.6377834	
Alpk2		20.4153781	
Amdhd2		2.72376449	
Amer1		2.50995694	
Amigo1		3.81781961	
Amotl1		26.0829555	
Amotl2		24.7605536	
Anapc11		3.29136308	

Anxa3		21.6085266	
Anxa4		14.8369377	
Ap1s2		2.40093345	1.98776764
Ap2s1		3.48003515	1.86497009
Ap5b1		6.33735842	
Apaf1		2.06732784	
Aplnr		30.0045046	
Apobec2		24.9861214	
Apoe		19.3016519	
Apol7c		24.543774	
Aqp1		81.2563921	
Arhgap22		8.73006457	
Arhgap24		10.6923694	
Arhgap27		2.1418413	1.94895437
Arhgap27os2		6.878385	
Arhgap30		2.11607334	1.79964775
Arhgap31		3.61649332	
Arhgef40		6.33696244	
Arid3a		2.878584	
Arl6ip1		2.40574398	1.94849382
Arl6ip4		2.23987271	
Armc6		2.12372892	
Arnt2		21.8895476	
Arpc4		2.06272118	
Arpc5l		2.19781176	1.75259422
Art2b		6.88281025	
Art3		11.6067447	
Asb13		2.40575166	1.85153798
Asb2		27.5032904	
Asb4		31.6363175	
Asph		22.3078655	
Atcay		16.7749754	
Atf6b		2.61871193	
Atox1		3.34816529	
Atp5j2		2.14166856	
Atp5k		6.18003397	
Atp6v0a1		6.6997454	
Atp6v1g2		9.79533858	
Atpaf1		2.15615339	
Atpif1		2.44408892	
AW011738		8.87537597	
AW209491		3.46661433	
Axl		13.8464986	

B130055M24Rik		2.96260982	
B4galt4		38.793247	
Bace2		15.3317835	
Bad		4.0497218	
Banf1		2.44298764	1.81814784
Batf3		10.9622373	
BC003965		2.43536311	
BC017643		2.53776336	
BC025920		3.40248209	
BC029722		2.24200874	
Bgn		510.447008	
Bloc1s1		2.23624608	
Blvrb		5.36680887	
Bmp1		28.4503004	
Bmp10		37.3949204	
Bnip2		2.06538758	
Bola1		6.53831316	
Bola2		4.05391366	
Brms1l		2.9497799	
Bscl2		2.39400426	
Bsg		2.73563716	
Bst1		16.2806316	
Btk		6.31064967	
C030034I22Rik		3.01089842	
C130074G19Rik		16.6668924	
C1qa		92.1880061	
C1qb		96.4935678	
C1qc		293.397024	
C1ra		92.0398298	
C1s1		179.411509	
C230062I16Rik		8.30599162	
C3		580.455091	
C4b		428.243976	
C5ar1		51.9392179	
C6		89.7485323	
C920021L13Rik		9.8506563	
Cacna1b		18.9042393	
Cacna1h		26.1919261	
Cacna1i		22.0001548	
Cacnb3		9.78592472	
Cacng8		27.5934625	
Calhm2		3.62774876	
Calm3		2.82151263	1.94581265

Camkk2		2.5912601	
Capg		7.75286536	
Capn2		2.12238856	
Card14		2.13319872	
Cav1		36.7090572	
Cavin1		20.5817202	
Cbx6		4.2406679	
Ccdc38		4.10945104	
Ccdc40		17.2065102	
Ccdc51		3.34306584	
Ccl2		48.9676933	
Ccl21a		151.936999	
Ccl22		16.4422305	
Ccl6		29.3263005	
Ccl7		23.5076458	
Ccl8		190.673599	
Ccl9		15.692312	
Ccnd1		3.15063435	
Ccnd3		2.49968582	1.60444909
Ccnf		3.90378808	
Ccr1		21.659944	
Ccr4		88.6818369	
Ccr6		18.8116158	
Cd151		4.79917028	
Cd19		7.6600078	
Cd200r1		15.5672589	
Cd209b		357.698132	
Cd248		24.1352963	
Cd300a		12.3349658	
Cd300c2		13.7662525	
Cd300lb		15.8108925	
Cd300ld		18.5338213	
Cd300lf		18.2335194	
Cd34		28.6878108	
Cd4		13.7529369	
Cd40		11.5366412	
Cd5l		133.863455	
Cd63		43.0706933	
Cd79a		9.68307596	
Cd81		3.59016373	
Cd93		25.5109332	
Cdc20		3.07627569	
Cdh5		44.5961911	

Cdkl3		7.53374215	
Cdkn2a		67.0881574	
Cela1		241.726282	
Cela2a		219.049428	
Cenpx		2.54466961	
Cep250		2.16266678	
Cercam		4.74604142	
Cfb		124.171042	
Cfh		20.7728848	
Cfp		5.50478647	
Chaf1b		2.57065059	
Chil1		36.9574148	
Chmp5		2.03862777	
Chst1		27.551077	
Chst14		3.6291818	
Chst3		7.69613525	
Ciita		5.87772052	
Cish		4.00984247	
Ckap4		3.78245723	
Clca3a1		25.2684272	
Clec10a		25.8148236	
Clec14a		26.0201025	
Clec3b		40.7573583	
Clec4a1		27.2821702	
Clec4a3		42.3964097	
Clec4e		41.5111746	
Clec4n		49.4136516	
Clec5a		19.2684207	
Clic1		2.40966775	
Clip3		15.2673485	
Clmp		11.5393679	
Cma1		43.8547158	
Cmc2		11.237843	
Cmklr1		18.4398866	
Cmtm3		3.6269372	
Cmtm7		15.4286235	
Cndp2		2.84402483	
Cnpy2		4.22535694	
Col1a1		169.281811	
Col23a1		5.32927283	
Col3a1		780.176411	
Col4a1		559.975867	
Col5a1		114.300392	

Commd5		2.99165114	
Comt		3.01596316	
Comtd1		2.66400174	
Cops7a		2.21688864	1.82550488
Coro1b		2.47868038	1.65873075
Coro1c		2.54366309	1.99208962
Cox17		2.52132477	
Cox5a		2.95927726	
Cox5b		2.22510858	
Cox6a1		3.29917197	
Cox8a		4.13888064	
Cpa3		60.0684447	
Cpb1		175.733053	
Cpe		17.3323294	
Cpq		4.65453358	
Cpt2		4.55226926	
Crebl2		2.21958108	
Creg1		2.39575233	
Crispld2		12.7720292	
Csf1		10.1960173	
Csf2ra		5.01880883	
Csf2rb		8.5097019	
Csf3r		9.84058388	
Csrp1		2.91037128	
Cst7		3.04743112	
Ctgf		25.3577362	
Ctnnb1		2.16611777	
Ctnnd1		23.2656721	
Ctrb1		997.539453	
Ctrc		99.7999509	
Ctsb		3.83619286	
Ctsh		8.13396753	
Ctss		2.79892484	
Ctsz		5.29965266	
Cttn		10.892854	
Cuta		2.21086013	
Cx3cl1		26.3640039	
Cxcl10		49.1880111	
Cxcl12		264.716505	
Cxcr6		7.47924595	
Cxx1a		8.05164006	
Cyb561		27.0617718	
Cyb561a3		2.25343136	

Cyba		3.64098269	
Cyp3a13		29.5880734	
Cyp4f16		5.25397674	
Cysltr1		18.6700263	
D10Jhu81e		3.12807697	
D130017N08Rik		6.24089828	
D17H6S53E		2.86168891	
D430001F17Rik		2.46089692	
D530033B14Rik		4.40812157	
D630039A03Rik		45.7147086	
Dab2		41.3034554	
Dbnl		2.02651524	1.89063698
Dclk1		16.8492427	
Dcn		238.199038	
Dctn5		2.0707824	1.78307051
Ddc		24.6180347	
Derl3		14.669075	
Des		48.3962299	
Dip2a		3.56734721	
Dleu2		3.03846941	
Dmpk		23.3547923	
Dnm3		13.2272614	
Dnph1		21.6513792	
Doc2g		6.53840723	
Dok3		18.7632351	
Dpm3		3.96367558	
Dpt		53.8163736	
Dpysl3		34.043905	
Dpysl5		12.8420493	
Dst		11.2063465	
Dtd2		3.08356341	
Dusp3		12.1321904	
Dut		2.79870878	
Dyrk4		22.4758019	
Ebi3		10.945897	
Ebp		5.15956155	
Ech1		3.00094731	
Ecscr		26.384089	
Efemp2		7.31341257	
Egfl7		33.2035088	
Ehbp1l1		2.2775335	
Eme1		3.33059573	
Emilin2		31.7748534	

Emp2		14.7958104	
Enah		10.0035484	
Enkd1		6.52639029	
Enpp5		2.91628697	
Epb41l3		26.5007828	
Epha3		21.4829919	
Etl4		10.2846415	
Evi2a		2.63756622	
Evpl		12.1505311	
Exo5		2.82266892	
Exoc3l		5.59072401	
Exoc3l4		13.8626678	
F13a1		50.0301034	
F2rl3		8.75880241	
F730311O21Rik		19.8439389	
Fabp4		62.1125901	
Fahd1		6.25648287	
Fam102a		2.15731126	
Fam110a		3.39527289	
Fam120c		2.88526521	
Fam129a		3.59750013	
Fam162a		2.39610746	
Fam26f		5.67595587	
Fam3a		3.03316046	
Fblim1		17.8810368	
Fbln2		44.3603843	
Fbn1		126.385914	
Fbxl19		3.17059998	
Fbxo17		5.16535316	
Fbxo27		2.80137626	
Fbxo41		47.2130513	
Fcer1g		40.2205342	
Fcgr1		32.4030155	
Fcgr2b		25.5369621	
Fcgr3		22.8945513	
Fcgr4		31.5904675	
Fcmr		10.5206045	
Fcrl1		8.91298313	
Fdxr		2.42848884	
Fen1		2.03224736	
Fermt3		2.55068683	1.78143322
Ffar2		19.4948035	
Fgd2		4.37299056	

Fgd5		16.8202081	
Fgf23		13.7522603	
Fgr		7.1398181	
Fkbp2		3.06805196	
Fkbp5		2.60297589	
Fkbp9		41.7035955	
Flad1		2.52623661	1.91077765
Flrt3		16.3044191	
Flt4		50.1435412	
Foxj1		9.97963534	
Foxm1		3.43425453	
Fscn1		16.2137815	
Fstl1		104.036772	
Ftsj1		2.91114776	
Fubp3		2.014339	
G6pdx		2.47337645	
Gab2		9.62237137	
Gadd45g		10.2209975	
Galr3		31.7434885	
Gar1		3.19386771	1.99896897
Gas2l3		5.73455154	
Gata1		29.8697909	
Gatc		2.28102603	
Gatm		10.6846649	
Gbp2		7.7149622	
Gbp2b		23.3305023	
Gbp3		4.31677343	
Gcnt1		17.170481	
Gda		22.789139	
Gdpd5		4.18760599	
Gfra2		41.3373847	
Ggact		3.17556238	
Gins3		2.2612383	
Glipr2		2.7138306	1.88909056
Glmp		2.08629324	
Glycam1		77.5208831	
Gm11725		6.81682517	
Gm13387		12.6873236	
Gm14548		17.7757154	
Gm15506		5.37503761	
Gm15821		2.15374159	
Gm15987		16.6377062	
Gm16567		49.0503669	

Gm16675		7.35617114	
Gm17092		8.04211004	
Gm17122		2.7249464	
Gm17189		21.847942	
Gm19412		2.81484281	
Gm20163		10.5184895	
Gm20412		45.8543551	
Gm20425		2.36249112	
Gm20499		3.53642528	
Gm20547		122.423491	
Gm21188		70.4319369	
Gm21987		4.21158689	
Gm21988		2.36650426	
Gm26564		2.31941534	
Gm26625		6.80926479	
Gm26716		11.3947439	
Gm26850		3.5348985	
Gm26857		8.26145355	
Gm26888		19.9769228	
Gm26896		4.12256022	
Gm27021		4.34673901	
Gm27029		2.1822463	
Gm28038		2.53463318	
Gm28041		5.45481949	
Gm28221		10.4177772	
Gm28635		3.46842608	
Gm28731		3.96639927	
Gm2a		2.02692669	
Gm31748		5.63493219	
Gm3336		18.2752888	
Gm36551		39.7754153	
Gm37795		15.5021012	
Gm37881		13.8488218	
Gm37893		5.10683052	
Gm42141		2.84579628	
Gm42372		11.2934721	
Gm42674		7.55689107	
Gm42889		99.0014314	
Gm43068		20.7931533	
Gm43584		10.678576	
Gm43597		4.68913013	
Gm43755		31.4100461	
Gm44250		7.59315666	

Gm44878		3.60493011	
Gm44986		2.9873202	
Gm45133		3.95574409	
Gm45205		2.41544642	
Gm45235		5.42215691	
Gm45418		13.1570258	
Gm45705		7.17193732	
Gm45706		7.1719378	
Gm45717		44.4910036	
Gm45799		2.93690985	
Gm5431		45.5034223	
Gm6904		5.27805599	
Gm7160		4.5319093	
Gmnn		2.73958645	
Gnpda1		3.14764473	
Golga1		2.19711417	
Gpm6b		6.66837089	
Gpnmb		440.921596	
Gpr107		2.15992646	
Gpr35		13.5318332	
Gpr65		2.41674446	
Gpx1		2.25275069	
Grem1		46.1719328	
Grina		5.1635023	
Grk3		3.69820077	
Grn		6.30992108	
Gskip		2.09466381	
Gstm2		18.0856048	
Gstp1		3.18149561	
Gtf2h3		2.1654112	1.87488248
Gucy1a3		16.7053857	
Gzma		11.7655157	
Gzmk		9.23470904	
H2afj		4.98838395	
H2afx		3.91008248	
H2-Eb2		17.1349712	
H2-Ob		5.19590294	
H2-Q10		5.593645	
H2-T23		7.51006945	
Hck		8.50590842	
Hdac6		2.15499961	1.9861545
Hebp1		54.358025	
Hectd4		2.27357645	

Hexa		2.26605057	
Hfe		9.9348572	
Hic1		8.0083069	
Hint1		2.13862903	
Hirip3		2.13085637	
Hk3		15.0131569	
Hmcn2		62.8166445	
Hmga1		2.59266478	
Hmgn3		9.13446124	
Hopx		5.23394641	
Hotairm1		19.0353394	
Hoxa1		17.0272739	
Hoxb3		13.2217492	
Hoxb4		5.03526512	
Hp		77.8172374	
Hpgds		2.66912911	
Hpse		12.9533924	
Hsd3b7		4.33667468	
Hspd1		2.09379589	1.91195229
Hspg2		166.08246	
Htatip2		2.37193249	
Htra1		41.8653373	
Htra3		20.4696109	
Hvcn1		3.07804434	1.98396923
Hyal2		6.54948345	
Icosl		3.85344518	
Idh1		4.73720472	
Idnk		2.09513337	
Ifi202b		120.279156	
Ifi207		11.4009465	
Ifi211		16.383399	
Ifi30		8.82446768	
Ifi44		56.7358037	
Ifitm2		47.8173753	
Ifitm3		68.8890922	
Igf1		70.2280555	
Igfbp3		38.2134577	
Igfbp7		58.0662986	
Igip		3.03202985	
Igkj2		32.2318702	
Igkj5		51.9776091	
Iglc2		11.68079	
Iigp1		14.9238773	

Ikbip		5.67015135	
Il1a		16.5473325	
Il1r1		34.7417752	
Il1rn		37.1931033	
Il21		16.7651033	
Il2ra		31.1527619	
Il4i1		3.62041204	
Il7		9.28177852	
Il9r		10.036577	
Inafm2		4.36736883	
Inha		17.2074889	
Inhbb		11.1592655	
Insl3		2.8905341	
Iqsec2		5.82777271	
Isg20		12.3292581	
Itga1		11.2452159	
Itgam		7.86742598	
Itgb5		31.9712071	
Itgb8		19.512522	
Itm2b		2.01543028	
Itpripl1		2.66569606	
Jagn1		2.76824604	
Jchain		393.13629	
Jpx		3.7448022	
Jrkl		2.81793981	
Kank2		25.6398419	
Kank3		6.79106806	
Kantr		3.0643998	
Kbtbd6		11.731098	
Kcnk15		58.8052368	
Kctd17		11.9823157	
Kctd21		8.87326308	
Kdelc2		19.1010475	
Kdelr2		2.26940961	
Kdr		42.1991958	
Kifc5b		3.40395206	
Klra2		23.6296373	
Klrg1		5.53008959	-11.283633
Krtcap2		2.45595468	
Lag3		4.09187739	
Lama5		109.108815	
Lamb1		51.8572933	
Lamb2		34.0119345	

Lamc1		6.2347718	
Lat2		4.06453041	
Lbhd1		2.34511735	1.97428449
Lenep		3.69164966	
Lfng		2.16460173	
Lgals1		4.56359881	
Lgals4		3.06153682	
Lgmn		10.5338978	
Lhfpl2		26.0777313	
Lif		34.0920123	
Lima1		21.1324466	
Lmnb2		2.99966534	1.91565127
Lpar1		26.8370782	
Lpl		59.9670909	
Lrg1		55.2770258	
Lrp1		28.657697	
Lrp11		10.8635549	
Lrrc25		16.5979743	
Lrrk2		6.18700012	
Lrrn4		40.6337642	
Lsm11		2.31466777	
Lsm2		3.15698005	
Lsm6		2.55746721	1.98695151
Lsm7		2.42068541	
Lst1		19.3885972	
Ltbp2		35.1446505	
Ltbp4		84.6514878	
Ltbr		9.41158403	
Lurap1		10.2896276	
Ly6i		72.2638379	
Ly6k		18.631182	
Lyl1		24.4327634	
Lyn		6.12278502	
Lyz1		65.6511934	
Lyz2		27.3547117	
Lzts2		3.28049381	
Madcam1		26.1121971	
Maf		7.89630972	
Mafb		49.0192318	
Magt1		2.0377569	
Man2b2		3.35983766	
Map6		470.628709	
Mapkapk3		2.66249637	

Mark1		38.9684053	
Mbd4		2.19359054	
Mcemp1		23.0516261	
Mcm5		2.31209843	
Mcm7		2.11288372	
Mdfic		9.14085713	
Mecr		2.78773786	
Med20		2.02393974	
Med9		2.67518231	
Megf8		9.49067493	
Meox1		20.4024966	
Mertk		73.2596354	
Mfsd4b4		2.55641782	
Mgat4b		3.52221506	
Mgat5b		26.2186516	
Mgl2		26.7472701	
Mgst1		41.0963478	
Mical2		8.12571843	
Mlec		2.80676249	
Mmp11		3.28246703	
Mmp14		17.31866	
Mmp23		22.4152633	
Mmp9		10.1013903	
Mmrn2		31.6992063	
Mob3a		2.31290746	
Mocos		5.27267947	
Mogs		2.53993161	1.85969496
Mpzl2		17.3092792	
Mrc1		72.1947507	
Mroh8		4.42065777	
Mrpl12		2.31991941	
Mrpl18		2.06820709	
Mrpl22		2.17279389	
Mrpl38		2.01135397	
Mrpl51		2.46205633	
Mrpl57		2.43753632	
Mrps12		3.24178788	
Mrps15		2.20946762	
Ms4a1		10.7871432	
Ms4a4d		10.1406059	
Ms4a7		50.9259075	
Msl3		2.30694718	
Msr1		69.0600774	

Msrb1		2.60370197	
Mtcp1		2.88609552	
Mtfp1		9.29467707	
Mtg2		2.19376687	
Mvp		2.12352123	
Mxra7		39.6302405	
Myadm		7.38430976	
Myl10		94.8848107	
Myl6b		8.01327396	
Mylk		46.5029883	
Myo10		10.7049301	
Myo1c		4.01057936	
Myo1e		8.0662022	
Myt1l		28.2277928	
Mzb1		27.8593064	
Naa40		2.03989688	1.85378561
Nacc2		6.4109025	
Nagk		3.12742199	
Napsa		4.64957525	
Nav1		5.60892919	
Ncald		8.31881012	
Ncf4		3.40122381	
Ndufa1		2.73639099	
Ndufa12		3.86066908	
Ndufa13		2.16643826	
Ndufa8		2.0684483	
Ndufab1		2.43952787	
Ndufb10		2.05950585	
Ndufs6		2.3166151	
Neat1		2.77177228	
Neb		45.842594	
Necab3		13.4226556	
Nectin2		28.8860142	
Nemp1		2.65826673	
Nfam1		3.65339591	
Nfib		16.1657145	
Nhp2		2.67351448	
Nme1		2.31160661	
Noa1		2.16310048	
Nop2		2.13298046	1.80447665
Nov		36.2181851	
Nrep		37.4689294	
Nrros		2.25475548	1.8665434

Nsdhl		2.75506564	
Nt5e		2.24614713	
Ntpcr		2.75414602	
Nudt22		3.15022541	
Nupr1		22.4532219	
Oasl1		49.3345537	
Obsl1		20.9642849	
Ogfrl1		4.6526228	
Ogt		2.52304548	
Olig3		11.9151338	
Osbpl1a		24.9759993	
Osmr		25.5160233	
Ostm1		2.14130071	
Ovca2		2.03715729	
P2ry1		50.979805	
P2ry13		82.7746283	
P2ry6		21.1336547	
P4ha2		34.4573934	
Pa2g4		2.01179542	1.93050869
Pacsin2		2.65624496	
Pagr1b		2.44060594	
Pak6		53.4739782	
Paqr4		5.25879879	
Pard3		24.4095214	
Pard3b		21.3098165	
Parm1		206.371221	
Parp1		2.0210659	1.79989244
Parp3		2.21361313	
Pcdh1		12.1867312	
Pcna-ps2		88.8181343	
Pcnx2		10.299959	
Pcolce		10.7555159	
Pde6d		2.24065776	
Pdk3		2.0595371	
Pdlim2		2.95114041	1.88640099
Pdzd11		2.64676511	
Pfkfb3		2.32011971	
Pgs1		2.01457897	
Phf23		2.01689506	1.8483576
Pi16		16.0477115	
Piga		2.91992129	
Pigh		3.72737656	
Pigv		2.82811235	

Pigx		2.97049218	
Pigz		68.0597695	
Pik3r6		16.9066024	
Pilrb2		46.0882592	
Pin4		3.36600669	
Pirb		34.4489401	
Pkdcc		21.4732937	
Pkp3		2.9645125	
Pla2g2d		71.0404883	
Pla2g4a		31.2876278	
Pla2g7		78.2135224	
Plau		68.3171018	
Plcb3		3.03259296	
Plcg2		4.28887337	
Plekhf1		5.57982195	
Plekhg1		16.109168	
Plekhj1		2.16000961	
Plekho2		2.2216263	
Plin2		4.14119895	
Plin4		12.4232992	
Plod3		2.81643781	1.95009619
Plvap		127.05059	
Plxnb2		12.8888596	
Plxnd1		4.76300516	
Pmp22		24.8954043	
Pnkd		2.61433587	
Pnliprp2		21.2430853	
Pola2		2.0983994	
Pold1		2.30141623	
Polr2f		2.96970617	
Polr2j		2.40798896	
Polr2l		3.76333019	
Pomgnt2		18.2270447	
Pon3		12.3779792	
Postn		76.2511406	
Ppp2r2c		13.5295343	
Prdm1		3.91148326	
Prdx4		4.24729996	
Prf1		3.12203681	
Prim1		2.20817443	
Prn		54.9415532	
Prol1		87.5124716	
Prr33		2.66786057	

Prr5		14.5989669	
Prrt2		3.45415194	
Prrx1		57.7880071	
Prss23		22.3158202	
Psap		2.20889081	
Psma1		2.06303787	1.98933263
Psmb2		2.07665162	1.79152514
Psmb3		2.39220087	
Psmb5		2.19385474	
Psme2		2.5430166	1.94034034
Ptafr		15.6838068	
Ptgr1		5.40711282	
Ptpn7		2.32805861	
Ptprb		16.7593914	
Ptprcap		3.59611013	
Pura		2.85854078	
Pus1		2.27430583	1.84089194
Pxdc1		32.1530874	
Pycard		2.14529792	
Pygl		8.54935042	
Pyurf		2.92717429	
Rab27b		24.5150324	
Rab29		2.68694923	
Rab3a		4.10743287	
Rab3il1		50.3281274	
Rab5b		2.4250149	1.99371228
Rab5c		2.29645713	
Rad54l		2.9158045	
Ran		2.26992043	1.92060505
Rangap1		2.04359393	
Rap1gap		11.1303349	
Rasa4		3.01239312	
Rbbp7		2.22978645	1.86868305
Rccd1		2.44609806	
Reln		33.9456284	
Relt		3.09524627	
Rexo2		3.38707009	
Rgag4		8.24267508	
Rgp1		2.04972747	
Rhbdd1		2.35045567	
Rhbdd3		3.07238959	
Rnase4		9.85046819	
Rnaseh2a		2.22104188	1.80969713

Rnasek		2.82975819	
Rnaset2a		3.10916914	
Robo4		18.531517	
Romo1		2.43781003	
Rp2		2.41007928	
Rpn1		2.04319651	
Rprd1a		3.21661139	
Rps21		4.54970964	
Rps27l		3.94078794	
Rpusd1		2.86150025	
Rragb		3.04451183	
Rrm1		2.11975433	
Rsad2		50.9090193	
Rtkn		24.8586046	
Rtn3		2.01166298	1.89919775
Rubcn		2.13998586	
Rufy3		3.64550657	
Rundc3a		6.53752223	
Rwdd4a		2.01897051	1.93215789
S100a1		11.1302127	
S100a13		3.12947466	
S100a4		7.29873832	
S100a6		25.3197015	
S1pr3		14.1508997	
Sac3d1		3.89721802	
Samhd1		4.03232704	
Sarm1		4.19080947	
Sash1		49.8549484	
Saysd1		3.25876544	
Scamp4		3.03755935	
Scara3		24.1711404	
Scarb1		2.87514902	
Scd1		5.48578318	
Scgb2b26		61.9087281	
Scgb2b27		97.0815368	
Scimp		14.7551862	
Scin		16.4194503	
Scn1b		20.6313086	
Sdc1		5.33656185	
Sec61b		2.58030456	
Selenoh		2.63892489	1.88025832
Selplg		2.79521576	1.82482603
Sema4d		2.31523407	1.93223123

Sephs2		2.3833528	
Serpina1b		33.5423579	
Serpina3f		27.9818954	
Serpina3g		14.7822704	
Serpina3n		146.726294	
Serpinb1a		7.4469051	
Serpine1		34.3951168	
Serpine2		28.3468893	
Serping1		304.678784	
Serpinh1		212.466691	
Sf3b5		2.68573702	
Sfi1		2.62852333	1.99063659
Sfxn2		3.041315	
Sgsh		2.48487478	1.99763505
Sh3bgrl2		12.5209966	
Sh3d19		7.56661842	
Shank3		26.5282995	
Shmt2		2.01820782	1.71302599
Siglec1		261.559979	
Sigmar1		2.40763623	1.92843069
Sirpa		26.3030126	
Sirpb1a		10.5234928	
Sirt6		3.00762875	
Siva1		2.69496573	
Six5		14.2589954	
Skap2		3.35330728	
Skida1		23.9443047	
Skp2		3.6940683	
Slc11a1		13.3602009	
Slc15a3		9.73375109	
Slc16a3		8.36669236	
Slc1a3		32.2713971	
Slc25a10		2.84743355	
Slc25a39		2.25772094	
Slc25a45		2.46867797	1.76027705
Slc25a5		2.69374708	
Slc27a3		11.8776257	
Slc27a4		2.49395048	
Slc29a3		2.03723869	
Slc31a2		3.14503991	
Slc35c2		2.21358017	1.69483771
Slc39a2		13.3298048	
Slc39a8		3.43860408	

Slc46a3		2.67281632	1.96266316
Slc7a7		8.15398593	
Slc9a3r2		17.9061098	
Slco2a1		75.2117667	
Slco2b1		23.7213677	
Slco5a1		16.7990113	
Slfn4		29.7688093	
Slfn5		16.0231117	
Slfn5os		23.2113503	
Slpi		62.7515116	
Smoc2		15.8173998	
Smpd1		3.72387499	
Smtn		27.8813933	
Smug1		3.18302269	
Snapc5		3.62734096	
Snn		4.23266212	
Snrnp35		3.56535329	
Snrpa		2.01595411	
Snrpd3		2.33390508	1.77981427
Snrpf		3.4564005	
Snx8		4.54227154	
Soat2		25.321912	
Socs1		5.6014796	
Sod3		119.911487	
Sorcs2		15.7780066	
Sparcl1		31.8466607	
Spats2		140.086189	
Specc1		12.0749121	
Spi1		6.79381317	
Spic		47.7354298	
Spns2		20.8048097	
Sptssa		2.51650123	1.80510081
Srek1ip1		2.23998703	
Srsf9		2.04192111	
Srxn1		11.1437044	
Ssbp4		2.11908637	
Ssna1		2.06043611	
St14		5.39855372	
St3gal4		2.35452872	1.95666113
St6galnac1		64.0320542	
St6galnac6		6.15704845	
Stab1		34.4761934	
Stag3		40.7788896	

Steap4		19.0301089	
Stom		4.51802274	
Ston1		30.1653512	
Stx11		4.66923711	
Stx12		2.50418686	
Sugct		5.47391275	
Sulf1		53.6563652	
Sult1a1		8.99689816	
Surf1		2.1316151	
Swap70		3.84808243	
Synpo2		12.2163351	
Tagap1		2.11755587	
Tagln2		2.05480903	
Taldo1		2.46677993	
Tap2		2.34913596	
Tapbp		2.26515919	
Tbc1d16		21.9077244	
Tbxas1		12.6580448	
Tcrg-C4		55.0021675	
Tcrg-V3		28.0290168	
Tctn1		2.79765189	
Tecpr2		2.33709931	
Tenm4		11.5684983	
Tfec		18.8975789	
Tgfbi		17.7608915	
Tgm2		43.3237949	
Tha1		3.58618544	
Thbd		14.7371887	
Thbs1		44.7842175	
Thbs4		75.0976758	
Thg1l		2.81053764	
Thnsl2		13.8854267	
Thop1		2.19851798	
Thrsp		33.9773603	
Thsd7a		10.8849792	
Tie1		18.4523876	
Tifab		12.8258521	
Timd4		32.4543188	
Timeless		2.48091836	
Timm10		2.90026282	
Timm10b		2.75960387	
Timp3		50.4694193	
Tjp1		15.46612	

Tldc1	4.44421012
Tlr11	11.5506254
Tlr12	4.70226687
Tlr13	19.0586101
Tlr4	39.2528933
Tlr6	3.13348072
Tlr8	24.9965874
Tlr9	6.00692266
Tm4sf1	19.3587404
Tm4sf5	8.96238908
Tmbim1	6.79933964
Tmem101	2.54697489
Tmem109	2.27255136
Tmem127	2.02326458
Tmem129	3.42608698
Tmem132a	9.53832062
Tmem143	3.07168168
Tmem147	2.9168757
Tmem159	8.88006889
Tmem173	2.40342037
Tmem176a	18.3261864
Tmem176b	18.4240938
Tmem202	4.88941029
Tmem258	2.41922344
Tmem67	7.33763645
Tmem70	2.10214696
Tmppe	3.94701426
Tnc	160.101095
Tnfaip2	7.98015985
Tnfrsf13b	2.26559891
Tnfrsf13c	3.18366084
Tnfsf4	80.7810437
Tnfsf9	8.92547549
Tnfsfm13	23.7150866
Tnp2	52.4548687
Tns3	7.84833087
Tnxb	34.2092931
Tomm5	2.49560529
Tor3a	5.85729079
Tox2	10.9463691
Tpgs1	2.43687366
Tpsab1	46.6721044
Tpsb2	33.526527

Trafd1		3.90512863	
Trf		6.38895936	
Trgv2		25.4453491	
Trim16		4.24680095	
Trim30b		9.64344896	
Trim30c		12.0334065	
Trim30d		15.2802991	
Trim7		12.3286844	
Trim8		2.22736938	
Trmt12		3.8848502	
Tsen54		2.1485421	
Tshr		136.240987	
Tspan10		19.2505832	
Tspan4		6.50452821	
Tspan7		46.7787695	
Tspo		5.12540331	
Tspyl3		2.4753446	
Tspyl4		2.64148607	
Tssc4		2.608216	
Tssk6		4.64482481	
Ttc32		2.8435119	
Ttll12		2.5086281	
Ttn		12.5705346	
Tub		16.2737737	
Tubg1		2.38973106	
Tyms		2.28999391	
Tyrobp		6.85341652	
Ubd		33.2347682	
Ube2l6		13.705953	
Ugt1a7c		9.18013864	
Unc13a		3.13930372	
Unc5cl		4.06029091	
Upp1		19.4305886	
Uqcr10		2.89532032	
Uqcrq		3.02813878	
Uxs1		2.44944919	
Vcam1		31.2794283	
Vcan		11.470209	
Vim		2.94539249	
Vmp1		2.49338794	
Vsig10		24.0106776	
Vwa1		16.3475035	
Wars		2.98523483	

Wdr54		20.0884716	
Wfdc17		67.8250537	
Xpnpep3		2.07205975	
Ywhah		2.2407184	1.87095925
Zbtb3		13.8826083	
Zbtb7b		11.2064845	
Zbtb8a		2.80147678	
Zbtb8os		2.07505627	
Zdhhc12		2.41225085	
Zdhhc6		2.24340035	1.99719091
Zfp467		9.65253373	
Zfp473		4.20901381	
Zfp536		35.2360128	
Zfp787		4.07509202	
Zmynd15		5.12813009	
Znhit1		2.75220732	
Zswim3		5.42004607	
C1galt1			2
Zfp60			2
Daxx			2
Sept11			2.51822886
1110028F11Rik			14.1922464
1110038B12Rik			2.38712679
1700016P03Rik			3.48712229
1700020I14Rik			2.33917847
2210010C04Rik			31.4103562
2310058D17Rik			4.25371218
2610002M06Rik			2.40848779
2610509F24Rik			82.9385295
2900026A02Rik			11.0129858
4930453N24Rik			2.02986898
4930458D05Rik			5.82537561
4930480K23Rik			4.49555715
4930513N10Rik			2.38031518
4933421A08Rik			15.1674524
5033428I22Rik			11.2697267
5430427O19Rik			6.28412254
6030443J06Rik			5.33587629
Abcg3			2.19960593
Abr			2.03980326
AC122305.4			5.31477151
AC130217.1			9.80537226
AC138284.3			13.0556338

AC164092.2	7.73859001
AC171003.1	9.71853334
Acsf2	2.64128995
Acsl6	31.9302664
Adal	2.22664462
Adamts14	4.07939477
Adssl1	7.32742958
Afmid	3.2718562
Ago3	2.18834841
Airn	4.42273118
Akr1c13	2.52675162
Aldh7a1	6.26843177
Alox12	7.04449286
Antxr2	2.41439591
Arl4d	10.4282087
Arl5a	2.45150107
Armc2	241.647091
As3mt	8.13370064
Asap2	2.82221515
Atm	2.2038318
Atp6v1g3	15.1824049
AU022252	2.18035952
AW046200	6.9487104
B130034C11Rik	11.5311182
B230398E01Rik	2.35720298
B3glct	2.15823797
B930095G15Rik	7.19651549
Bet1	2.58228766
Bmpr1a	4.57813049
Borcs7	3.11729929
C030029H02Rik	45.9572335
C330024D21Rik	8.9302825
C79130	11.629832
Cacnb4	5.37165968
Cacng6	56.6776596
Camk2a	8.41737234
Car9	18.8148132
Ccdc102a	3.02136364
Ccdc116	3.39798403
Ccdc50	2.0008966
Ccdc58	2.26971854
Ccl3	3.79603141
Cdc25a	2.58814846

Cdca4	2.10320105
Cdk2ap1	2.0625997
Cdk5rap3	2.7179831
Cdk6	2.24854757
Cdkn2aipnl	2.38584357
Cdpf1	2.14861993
Celsr1	2.29613974
Cenpv	3.20025171
Cep41	2.1590475
Cfap77	17.1713351
Chad	13.1096548
Chsy1	3.28425431
Ckap2	4.30333947
Cldn4	20.4195671
Clic4	3.25403265
Cmya5	11.124172
Cnksr1	52.202113
Cnrip1	3.03664092
Copg2	2.03941229
Cpa1	33.2300473
Crot	2.18799357
Crtam	3.43540676
Csnk2b	2.06512892
Csrnp3	77.2679272
CT009713.5	34.6236174
CT009718.4	2.04186878
CT010575.2	11.1653191
Cxcr5	6.25002715
Cym	16.41593
Cyp17a1	20.1550328
Cysltr2	4.63693832
Dap	2.6246935
Dapk2	3.67365244
Dars	2.25218869
Decr1	2.30239453
Dgkh	6.7614302
Dhdh	8.61551375
Dlg5	3.49454564
Dnmt3b	3.21320911
Dock5	2.15765676
Dsg2	25.982749
Dsp	77.1722737
Dtl	2.40247685

Dtnbp1	2.67859707
Dus4l	2.45222639
Dusp19	2.47933953
E330009J07Rik	2.44511316
Eci1	2.40229563
Eea1	2.35443546
Efcab6	51.143811
Enc1	2.56883579
Eno3	2.42028755
Entpd4	21.4672264
Erap1	2.01994445
F2r	2.57424938
Fam109b	17.4718338
Fam184a	14.9963792
Fam206a	2.00308841
Fam208a	2.10183047
Fam214a	2.38328959
Fam83h	5.81178373
Farp1	6.60822589
Fasl	4.25015653
Fes	3.84689532
Frem2	91.3570437
Galm	2.69980827
Galnt7	2.10233354
Gemin5	2.64530721
Gemin6	4.10922924
Gimap8	2.3569104
Gk	2.16347109
Gm10612	19.6045225
Gm11739	3.13728776
Gm12319	4.72621253
Gm12454	13.5439468
Gm12503	5.46086497
Gm13212	3.7668915
Gm13856	3.50703287
Gm14230	2.87317398
Gm14326	4.25404319
Gm15228	100.011524
Gm16086	15.1501519
Gm16090	14.6403605
Gm16158	2.69868975
Gm16364	2.76308615
Gm16731	4.10410867

Gm17035		6.47173305
Gm19261		3.0579984
Gm20707		2.66350327
Gm20752		30.1388848
Gm20939		7.32276355
Gm26588		10.2663713
Gm26773		2.62537355
Gm26789		3.51866002
Gm28379		2.50981632
Gm28610		7.45364299
Gm28935		10.8619162
Gm28942		24.8853531
Gm2895		8.85461929
Gm30414		17.4232744
Gm3558		12.3368369
Gm36401		8.83679146
Gm37010		17.6161361
Gm37249		25.0219581
Gm37261		8.72221457
Gm37589		3.25477738
Gm37697		26.3335051
Gm38037		14.1375985
Gm38303		2.89320894
Gm38340		11.0184981
Gm38372		3.52214824
Gm4208		15.6057394
Gm42987		7.39898236
Gm43196		2.94922037
Gm43339		3.05318337
Gm43961		4.96954025
Gm44002		7.09545138
Gm44238		20.5076383
Gm44667		3.03724974
Gm44672		3.70781128
Gm44697		12.3145804
Gm44752		14.9667652
Gm44888		2.82847647
Gm45426		19.3752196
Gm45527		4.95650079
Gm45746		14.6278283
Gm4924		2.22988914
Gm5141		2.93807481
Gm7334		8.50168356

Gm8817		16.5137934
Gne		2.18001986
Gnptab		2.1883537
Golim4		2.54468734
Gpat3		5.66717863
Gpr45		9.57227229
Gpx7		4.80005189
Grhl3		6.38646709
Gsap		3.35323031
Gtf2a1l		14.4196177
Haus1		2.78551999
Hdgfl2		2.07206191
Heatr5b		2.2232642
Helz2		2.04996808
Hgsnat		2.32906496
Hmbox1		2.26065026
Hook3		2.13119416
Hprt		2.02313898
Hrh2		2.38719422
Hs1bp3		3.26023914
Hsf4		4.30301396
Ice2		2.47767057
Ide		2.45637079
Ifi209		2.53894249
Ifi213		3.05231153
Ifi27		2.29452916
Ifitm10		2.53403454
Igf2r		2.62580443
Ikzf3		2.35043678
Il18rap		2.65596941
Il2rb		2.75765475
Inpp5b		2.18654569
Ipo5		2.12887425
Iqcf5		18.7755395
Irf9		2.51730271
Itga2		5.37414942
Itm2a		8.69145829
Kbtbd12		49.3991616
Kbtbd13		19.2797996
Kcnq5		2.77264585
Klc1		2.0095755
Klhl30		96.7538455
Klhl32		13.0674216

Klhl5		2.71581917
Klrc1		5.99209024
Klrc3		18.4212859
Klrd1		2.62712028
Klre1		30.4265813
Klrk1		4.87501931
Krtcap3		2.83182468
Lactb2		3.29566613
Lax1		2.82531972
Lekr1		6.02633347
Lgals9		2.32867062
Lmbr1		3.28853561
Lpp		2.20169636
Luzp1		6.00745616
Ly6c2		5.68302621
Man2a1		2.4960334
Marveld2		12.4215441
Mbnl3		2.74272306
Mcts1		2.10384203
Mdn1		2.24454403
Mef2a		2.78454808
Mettl4		4.13026042
Mex3a		2.73742357
Mgll		4.83820694
Mmab		2.16571865
Mmp16		30.3124585
Mms22l		2.33625163
Mns1		2.73563752
Msh2		2.64596595
Msi1		3.86433233
Mst1		2.60338285
Myo1f		2.24556508
Myo7a		21.2712685
Myom2		15.9718736
Mypopos		2.79324876
N4bp1		4.30052134
Nap1l5		6.74660149
Napepld		3.23868634
Nat6		2.63569262
Nbeal2		3.03145757
Nck1		2.12640798
Ndrg1		5.76241154
Ndufa4		2.01974228

Nedd4	4.58991906
Nek4	4.76437176
Nmnat2	5.07156868
Nod1	2.57316897
Nradd	3.86761564
Nt5dc2	9.12432233
Nudt14	2.52662692
Nudt2	3.13002238
Nudt5	2.21623635
P3h4	19.2114764
Pafah2	2.69221915
Palld	2.72150167
Park7	2.07666575
Parp10	2.01642126
Pcgf2	3.50481066
Pdgfb	5.62799494
Pdk1	2.19415526
Per3	5.59157951
Pgd	2.36752781
Pik3ap1	11.7890488
Pik3cg	2.02855139
Pik3r3	2.43310792
Pkn3	5.72798824
Platr11	26.4049112
Platr17	2.79754088
Platr25	2.65069439
Plch1	10.7632377
Plekha8	2.81841986
Pls1	15.1343267
Pmch	15.4098202
Pnliprp1	36.089562
Pnpo	2.33221208
Pnpt1	2.25917968
Polk	2.66145716
Pot1a	2.07464018
Ppp1r26	28.4099224
Ppp3cc	2.59774583
Prag1	4.5716418
Prdx2	2.26517754
Prkdc	2.10168084
Prps2	2.60732914
Prpsap1	2.11798335
Prr13	2.28998194

Ptgr2		2.65379959
Ptpn12		2.07265829
Ptpn14		4.53223241
Ptpn4		2.68029514
Ptpn5		58.7505902
Pvt1		3.26375848
Racgap1		2.01355654
Rad50		2.74579719
Rad51d		2.06955268
Raph1		4.07331411
Rars2		2.17898833
Rasl12		117.93567
Raver2		2.06229882
Rbbp9		3.1368343
Rbl1		2.00156554
Rfx5		2.34397576
Rif1		2.14751109
Rims2		73.7831585
Rln3		12.8146506
Rnf122		2.40700548
Rnf130		2.77511295
Rnf169		2.43734082
Rnf43		5.72131485
Rsad1		2.5582717
Rtkn2		21.3744053
Ryk		6.48940285
Sass6		2.92019849
Scamp5		34.288047
Scn2b		7.55903821
Scrn2		3.22934335
Scube1		62.0851562
Sele		10.0690695
Serpinb5		31.735432
Sh2d1a		3.23427082
Sh2d3c		2.08099707
Sike1		2.13669426
Slc16a2		6.2369631
Slc35a5		2.58940768
Slc35f2		2.02045942
Slc37a2		4.40212674
Slc39a10		2.93040892
Slc39a5		17.9691727
Slc43a2		2.46787292

Slc43a3	4.35635343
Slc9a7	3.20653844
Snx10	2.60388164
Sp100	2.0546882
Spcs3	2.10821289
Spopl	2.12647346
Srbd1	2.3330228
St3gal6	4.77636965
St6galnac4	2.36676364
Stap1	2.30494306
Stard10	2.66614536
Stn1	2.33002197
Styk1	15.9496911
Sult5a1	84.9253437
Tbx21	3.44582725
Tcam1	35.3976678
Tex9	2.89925463
Tgds	2.38828729
Tia1	2.08047018
Tmem163	4.84040839
Tmem200a	25.900846
Tmem59l	110.720483
Tmpo	2.20265491
Tmtc1	9.03429944
Tmtc4	2.14354573
Tnip3	10.1955898
Trim2	2.88097876
Trim42	16.9617591
Trio	2.70012206
Trip4	2.51882543
Tshz3	3.99565503
Tspan9	17.242181
Tstd3	2.44248763
Tubd1	4.1550075
Tyro3	2.49543056
Utp11	2.09203055
Wdr6	2.41764366
Wipf1	2.18738823
Ybx3	3.28163888
Zbtb18	2.89709369
Zbtb37	2.36492675
Zc3h7b	2.18261196
Zfp160	2.39805665

Zfp322a		2.27122489
Zfp367		2.58257557
Zfp493		6.63920703
Zfp512		5.87783707
Zfp652		2.20981927
Zfp683		10.5697964
Zfp712		5.08068419
Zfp758		2.10910044
Zfp760		5.90486762
Zfp780b		2.45992044
Zfp808		2.23682268
Zfp935		2.13195125
Zfp950		2.70501873
Zfp984		2.2501391
Zfp985		5.41759388
Zfp987		3.00397017
Zfp991		7.68614128
Zfp992		2.77368537
Zfp993		22.9555661
7-Mar	-5.4155751	-3.0827733
1700113A16Rik	-4.4883932	-3.6969506
1810026B05Rik	-4.9572192	-3.4313508
2310039L15Rik	-30.141631	-37.59791
2810455O05Rik	-3.448099	-2.9963696
3110043O21Rik	-3.0513023	-5.0275365
4930511M06Rik	-31.790619	-18.717381
4930523C07Rik	-3.5459451	-3.313465
4930562F07Rik	-3.7130454	-3.4367243
5430405H02Rik	-3.4563392	-3.3789328
5730508B09Rik	-3.3797388	-3.9778518
6720427I07Rik	-2.5680085	-3.400029
9330162G02Rik	-3.4675899	-2.5100453
9430098F02Rik	-7.6691732	-3.7843125
Abca1	-2.2293698	-6.1524741
Abt1	-2.3525702	-2.3716841
AC113441.3	-16.935159	-47.099412
AC124179.2	-2.7988455	-2.5253159
AC124414.1	-5.1508807	-3.2630716
AC124556.1	-4.9746131	-2.6615093
AC125071.1	-33.543788	-7.3862786
AC129597.1	-7.1662361	-3.7634775
AC132332.1	-17.705224	-17.089664
AC151971.5	-21.905313	-4.4959226

AC153912.4		-3.337927	-2.0432615
AC163032.1		-2.0014532	-2.4961301
Acot2		-3.1778279	-2.9329237
Actn1		-5.3970608	-20.955768
Adh1		-6.1198476	-10.980883
Aebp2		-2.525925	-2.4023988
Afap1		-8.3303122	-41.353365
Aff1		-3.6600475	-3.1448848
Aff4		-3.5991914	-2.2216542
Akap2		-6.8939904	-9.5601744
Alpl		-12.158517	-49.526589
Amd1		-7.6453601	-5.2776283
Amigo2		-4.7470037	-8.2313538
Ankrd13c		-3.0041783	-2.2810124
Ankrd50		-7.7603076	-5.3439191
Arih2		-2.5521373	-2.1925832
Arl5b		-5.3865854	-2.8504565
Armcx5		-2.7244955	-2.2721986
Arntl		-3.1953709	-3.2409264
Arrdc3		-3.5014287	-4.0265581
Atg2a		-2.7689062	-2.9624942
Atg7		-2.1309911	-2.2824495
Atp6v0d1		-2.1159312	-2.3880892
Atp6v0e2		-16.767931	-47.519565
Atxn7l2		-2.6318727	-2.2311692
Avl9		-3.8505864	-2.7301312
Bach2		-4.1528348	-4.4883631
Bach2os		-4.1816183	-4.6690156
Baiap2		-2.4046225	-2.6697821
Baz2b		-2.5025804	-9.2816354
Bbs2		-2.4547747	-2.0352297
Bcl10		-4.1990655	-3.9409689
Bcl6		-2.0218965	-3.5988933
Bcl9		-2.3167049	-4.7336819
Bend3		-2.9576753	-2.4153245
Bex2		-14.734271	-21.282888
Birc2		-2.8450453	-2.194765
Birc3		-3.2375592	-2.5968562
Blzf1		-2.8972437	-2.1007508
Bod1		-2.9425698	-2.9513996
Bri3		-2.2626589	-2.730528
Bsdc1		-3.2659244	-2.6807048
Btg1		-6.125637	-3.7751483

Btg3	[redacted]	-13.69878	-7.6560604
C1qtnf12	[redacted]	-2.587578	-4.3289442
C230038L03Rik	[redacted]	-3.8083762	-3.1047709
C230096K16Rik	[redacted]	-10.289278	-7.5636082
Cables2	[redacted]	-2.67388	-2.5896518
Cacng4	[redacted]	-22.011148	-28.243167
Cacul1	[redacted]	-3.2074074	-2.4215367
Calcrl	[redacted]	-3.6302079	-3.5851086
Car2	[redacted]	-3.5274128	-6.4811051
Ccdc174	[redacted]	-2.5811317	-2.0073146
Ccdc28a	[redacted]	-3.2129962	-2.6513785
Ccdc59	[redacted]	-2.273994	-2.5077833
Ccnyl1	[redacted]	-3.7833062	-2.8547715
Ccr9	[redacted]	-7.4284009	-14.224616
Ccsap	[redacted]	-3.1004667	-2.8237216
Cd226	[redacted]	-5.6671558	-44.681458
Cd28	[redacted]	-3.8915699	-2.3628653
Cdk11b	[redacted]	-2.7155847	-2.0770967
Cdk17	[redacted]	-2.7273783	-2.4432151
Cdk19	[redacted]	-2.5072504	-2.272045
Cdkn2aip	[redacted]	-3.2355784	-2.3123589
Cenpa	[redacted]	-2.6644463	-2.9935202
Chd2	[redacted]	-3.4074377	-2.8147488
Chic2	[redacted]	-2.9327827	-2.0188299
Chka	[redacted]	-2.9257378	-2.4213281
Cirbp	[redacted]	-2.8237278	-3.3117351
Cited2	[redacted]	-3.7383612	-3.7297415
Clint1	[redacted]	-2.9772065	-2.599551
Clk1	[redacted]	-4.9647924	-2.7589481
Cnga1	[redacted]	-3.3421713	-4.3857988
Cnksr3	[redacted]	-11.665275	-59.854669
Cnnm4	[redacted]	-2.2647229	-2.858415
Cops8	[redacted]	-2.8945827	-2.4167028
Coq8a	[redacted]	-2.2909962	-2.7690196
Cpeb4	[redacted]	-2.6785623	-2.212538
Cpne4	[redacted]	-4.4282672	-22.096637
Cpne8	[redacted]	-4.7289045	-23.581993
Crebrf	[redacted]	-4.2703513	-2.1723416
Cry2	[redacted]	-3.3758601	-2.4569336
Csrnp2	[redacted]	-3.1674437	-2.5935888
CT030161.3	[redacted]	-4.7214985	-16.953466
Ctdsp2	[redacted]	-2.0196171	-2.5196663
Cxxc5	[redacted]	-5.3939953	-19.996498

Cyth2		-2.2954577	-2.3689351
Cytip		-3.3238114	-2.5832814
D230025D16Rik		-2.1227716	-2.1529367
Dbf4		-3.5986862	-2.2327611
Dcdc2c		-7.4770784	-8.4950528
Ddx3x		-2.982558	-2.2348778
Dgat1		-5.1013729	-6.2389959
Dhx38		-2.3023727	-2.1278009
Dhx40		-3.5018619	-2.9235765
Dip2c		-5.3366917	-32.179523
Dnajb4		-6.9891377	-3.3237033
Dsel		-3.9037131	-40.397804
Dusp16		-3.0867011	-3.6980645
Dusp4		-5.8165063	-7.523129
E230016M11Rik		-5.7951195	-12.692607
E430014B02Rik		-12.796373	-3.811302
Efnb2		-5.0242483	-6.005199
Ehbp1		-5.3285384	-8.7244199
Ehd1		-2.2014535	-2.6061693
Eif1		-4.503527	-3.4163299
Eif2ak3		-3.1847166	-3.9080892
Eif5		-3.2842997	-2.0707858
Elmsan1		-5.8528418	-3.2486613
Emb		-2.9564885	-3.0093204
Epc2		-4.831416	-2.891501
Ephx1		-6.2920518	-3.9603065
Ets2		-2.3268681	-2.2574429
Ext1		-4.4661308	-7.7739641
Faah		-2.1070667	-2.6872461
Fam103a1		-2.9636733	-2.3523614
Fam107b		-4.4438902	-3.0548432
Fam117b		-3.0692963	-2.7747207
Fam209		-20.485689	-18.265504
Fam222a		-6.4729864	-6.58878
Fbrsl1		-2.9119233	-3.1173667
Fbxl3		-3.4537222	-2.3919808
Fbxo32		-5.9846754	-3.4448899
Fbxo33		-3.5038722	-2.8116247
Fdxacb1		-4.0438538	-2.8763794
Fnip1		-3.7340412	-2.3868156
Foxo1		-2.6079024	-2.6627317
Foxq1		-17.535887	-22.660839
Frat1		-12.325731	-6.7969344

Gabpb1		-2.9488582	-2.0606325
Gadd45a		-8.9081651	-16.564055
Gch1		-6.3679429	-4.4964212
Ginm1		-2.3661051	-2.5614915
Gla		-2.1641797	-2.7565065
Glce		-2.7628101	-2.4305219
Glt8d2		-4.9599495	-7.3159287
Gm14168		-2.7258359	-6.3239045
Gm15491		-3.7004075	-2.848701
Gm15503		-15.577316	-34.531297
Gm15638		-3.4619554	-3.3575512
Gm16229		-2.3775271	-3.5300267
Gm17024		-11.273756	-5.1656815
Gm17644		-11.073206	-8.7852408
Gm26561		-6.5415681	-3.4475428
Gm26635		-4.1227229	-2.7313495
Gm26649		-3.6948928	-2.2578374
Gm26690		-6.2645077	-3.9606888
Gm26735		-12.486286	-35.344911
Gm26767		-8.643038	-2.9848959
Gm26982		-12.478464	-6.5670428
Gm28035		-2.3331516	-3.3416317
Gm28042		-2.0833981	-3.4757847
Gm28306		-3.0142303	-2.4900696
Gm29542		-2.9988824	-2.5141698
Gm36445		-5.1345819	-3.2549728
Gm37370		-21.560358	-7.072938
Gm37637		-7.7196116	-3.3651798
Gm38077		-3.2004441	-2.0561709
Gm38220		-6.6078407	-3.4113101
Gm42595		-3.9296644	-3.5794424
Gm42636		-13.085708	-9.8745154
Gm43203		-6.5827755	-3.4929359
Gm43292		-3.1173636	-3.5820157
Gm43352		-12.092316	-10.07271
Gm43412		-7.9582228	-5.575683
Gm43652		-20.260641	-8.3847263
Gm44053		-11.047028	-4.5943757
Gm44103		-2.4417956	-2.0419776
Gm44553		-27.097569	-11.225181
Gm45244		-21.15002	-27.439881
Gm45718		-7.7635365	-3.4949303
Gm527		-9.355663	-3.6295196

Gm5608		-15.482363	-25.857711
Gm7008		-2.99759	-4.0750868
Gm7102		-32.90357	-57.473848
Gm9889		-21.756245	-21.633798
Gpatch3		-4.022759	-3.2335757
Gpr132		-6.5659884	-4.6275071
Gprc5a		-21.949307	-28.121164
Grk1		-3.7591833	-3.9683473
Gtdc1		-2.9142863	-2.0497264
Gypc		-3.0988153	-5.0889947
H3f3b		-3.2486287	-2.7054812
H60b		-7.95708	-6.6067836
Haus2		-2.6607343	-2.1421413
Hbp1		-3.8850884	-2.8057591
Hbs1l		-3.6155809	-2.4995318
Heca		-3.0159744	-2.2279603
Hes5		-9.9949864	-37.805006
Hexim1		-6.5452993	-4.1792255
Hic2		-3.5433091	-2.6372814
Hip1r		-8.7012425	-5.4820993
Hist1h1c		-7.375195	-5.2279208
Hivep1		-2.4483487	-3.4159927
Hnrnph1		-3.5081889	-2.0426194
Hspa5		-2.3866802	-2.4654259
Iba57		-4.9041249	-3.6496788
Ica1l		-3.1371618	-2.6931166
Ifngr2		-4.8876188	-43.664477
Ifrd1		-8.2265555	-6.7534581
Ikzf5		-2.8731333	-2.1197535
Il4ra		-3.2842166	-2.4419393
Il6st		-4.0793056	-5.6621234
Ing3		-5.2572805	-3.0117221
Ing5		-2.5770297	-2.0558461
Inpp5a		-2.8819848	-3.1870629
Insig1		-2.9430033	-2.4293327
Intu		-7.6992434	-7.4463161
Iqcb1		-6.0092976	-3.3251859
Irf6		-5.5512623	-3.6781664
Isca1		-2.8455741	-2.352482
Itk		-3.2104485	-2.3882692
Itpkb		-3.0397149	-2.318981
Jarid2		-3.0428386	-2.1188677
Jmjd7		-2.7480904	-4.0093864

Jmy		-9.0117379	-4.0122201
Kbtbd2		-3.6346002	-2.3173309
Kdm2b		-2.8724497	-3.997834
Kdm3a		-2.9624035	-2.2304209
Kdm5b		-5.484696	-3.2370155
Kdm6b		-9.1387289	-3.9933428
Kdm6bos		-9.3441775	-4.2483215
Kdm7a		-3.3254285	-2.0992153
Klf11		-4.5593986	-3.5480305
Klf4		-10.999031	-32.903895
Klf7		-3.603374	-2.0559818
Klhl24		-3.5818588	-2.1846257
Klhl6		-2.1612397	-2.0633801
Kpna1		-5.0207828	-2.8191705
Ldlrad4		-3.0311128	-2.6943431
Lef1		-5.8050058	-4.5559106
Lemd3		-4.2410869	-2.9130994
Lman1l		-3.7713199	-26.796222
Lmbr1l		-2.5737682	-2.9837765
Lrig1		-2.5778307	-4.0000078
Lrp12		-7.4298898	-26.527043
Lrrc14b		-2.6297167	-2.5726622
Lrrc8a		-2.2763125	-3.1976359
Maf1		-2.0309456	-2.210593
Mafk		-3.0507467	-2.6855385
Maml1		-2.9424076	-2.4471984
Maml3		-17.397516	-36.525322
Man1c1		-4.0873125	-10.913242
Map1lc3b		-3.4433976	-3.4562279
Map3k14		-3.136882	-3.3476443
Mapk8		-2.5649993	-2.0397872
Mbip		-2.4844301	-2.1270887
Med13l		-3.2740352	-2.6732067
Med16		-2.5127907	-2.8096071
Med26		-2.1465057	-2.1183121
Med4		-2.1635549	-2.0480762
Med6		-3.3414203	-2.3759043
Mef2d		-3.3829053	-3.0199952
Mex3b		-3.1577576	-4.8815883
Mex3c		-2.582825	-2.0965335
Mfhas1		-3.6565903	-6.5130878
Mgea5		-3.455074	-2.6355952
Mir17hg		-2.2379283	-2.1664803

Mob2		-3.0486377	-3.1730016
Morn3		-6.9154251	-6.2920738
Mtmr10		-4.0983351	-4.0604068
Mtmr14		-2.3794358	-2.5687563
Mtss1		-4.8147833	-12.898172
Mturn		-2.863336	-2.8523805
Mxi1		-2.2826716	-3.0521905
Nab2		-2.7837391	-3.6193501
Neil1		-3.9877375	-2.5764931
Nfil3		-6.7876642	-2.7671358
Nfkb2		-3.0151729	-3.8421156
Nhsl1		-11.937185	-74.336586
Ninj1		-2.0929484	-5.3788286
Nmb		-4.1705098	-4.6944018
Nr1d2		-5.6503852	-3.1223048
Nrip1		-4.4318778	-3.0821852
Ntrk3		-4.0050147	-7.5505919
Nufip1		-3.0517879	-2.0490707
Odc1		-3.4897214	-4.4097585
Olfr613		-6.9659458	-6.522877
Orai1		-2.4032436	-4.0234252
Oser1		-5.1017214	-3.9079503
Pakap		-6.8902521	-9.56358
Pank4		-3.3941793	-3.339197
Pard6g		-3.3058674	-6.0573769
Patj		-3.9847837	-3.795263
Pde3b		-2.129154	-2.4740287
Pde4b		-7.7546422	-7.8037149
Pde4d		-9.9857548	-15.019322
Pfn2		-3.694626	-2.4774673
Phc1		-2.2721478	-2.0391674
Pi4k2b		-21.354978	-20.016016
Pik3ip1		-2.1313478	-2.6312351
Pim3		-4.4476971	-4.7023955
Pip4k2a		-2.3938949	-2.7752145
Plag1		-5.9185339	-2.5134627
Plaur		-11.010143	-80.967671
Plcxd2		-4.1325264	-3.0247499
Plk2		-4.58945	-15.490046
Plk3		-3.1880327	-4.1864927
Pmel		-3.2560998	-4.6645757
Pnpla8		-5.3947919	-4.2344678
Pnrc1		-7.0908442	-3.469533

Polg2		-2.5163743	-2.1087012
Pop4		-2.9627616	-2.2714535
Pp2d1		-4.6831749	-3.1859409
Ppic		-12.564061	-160.02794
Ppm1a		-3.1250078	-2.3462095
Ppp2r2d		-3.5203304	-2.3079984
Ppp2r3a		-5.1317	-8.2053655
Ppp2r3d		-2.9468529	-3.1240457
Pramef8		-3.3661696	-2.5951335
Prdx6		-3.1317436	-3.0227949
Psd		-7.068723	-5.1489543
Ptp4a1		-5.7909469	-3.6043502
Pygm		-5.2025464	-3.9794927
Rab33b		-3.0413176	-2.2657257
Rab3ip		-3.9132091	-4.4743003
Rab5a		-3.1114677	-2.1555289
Rabl3		-2.8015465	-2.021174
Ramp1		-4.6024654	-77.444238
Ramp3		-55.14896	-145.84986
Rap1b		-3.019444	-2.4099465
Rapgef4		-3.5174611	-2.1483549
Rarg		-3.4001002	-19.965349
Rassf3		-3.552652	-3.6038736
Rb1cc1		-4.40494	-2.9883737
Rbm18		-2.9636942	-2.3287896
Rbm39		-4.2014658	-2.6187913
Rbm48		-3.7826444	-2.8360529
Rbsn		-2.1921912	-2.1411074
Rcn3		-4.8639813	-11.071896
Rel		-6.7009023	-3.369579
Rela		-2.1699898	-2.1744126
Relb		-3.9848667	-5.2808348
Reps1		-3.8591947	-2.7753081
Retreg1		-2.273113	-2.4421978
Rexo1		-2.0783678	-2.0168095
Rflnb		-4.7192099	-3.9880742
Rgcc		-4.1336682	-14.265194
Rgs10		-3.9298575	-3.7784424
Rhebl1		-3.825678	-3.4261342
Rictor		-3.3079461	-2.0360224
Rnf103		-4.3135844	-4.3059507
Rnf185		-2.9982869	-2.3302958
Rnf19a		-7.6056071	-8.182167

Rora		-4.4695225	-7.2617409
Rps2		-2.8359101	-3.1296892
Rrad		-2.744923	-3.0265305
Rragd		-4.136326	-2.1936715
Rybp		-3.2534236	-2.4013964
Samd8		-3.5542399	-2.508924
Scg5		-6.5225334	-20.062539
Sde2		-3.528276	-2.5110907
Sdhaf1		-2.1692406	-2.5999401
Sec61a2		-2.4700692	-2.0558084
Selenok		-2.8547782	-2.9648472
Sema6c		-5.2858172	-4.932902
Serinc1		-3.6526614	-3.4649415
Sf1		-2.4461574	-2.1198631
Sf3b1		-2.8921091	-2.0233035
Sfmbt2		-10.974672	-38.274044
Sgms1		-4.1533944	-4.0676519
Siah1a		-3.0105497	-2.3126312
Siah2		-4.3049011	-3.3952049
Sirt1		-3.6216622	-2.2021685
Ski		-3.1748011	-4.039068
Skil		-4.1548451	-2.3582693
Slc12a7		-2.4611238	-2.8398297
Slc27a6		-9.9907581	-37.030873
Slc35b3		-2.4460273	-2.3300194
Slc35d1		-3.3799438	-2.0410861
Slc35f6		-2.5547873	-3.3680209
Slc3a2		-2.3039691	-2.7875478
Slc7a14		-16.873622	-7.8675598
Slu7		-2.8398678	-2.0447879
Smad7		-6.7911241	-4.9076751
Smg9		-2.1430401	-2.5796555
Smim3		-5.4305621	-48.229632
Smndc1		-2.9698186	-2.0747697
Sox4		-10.487851	-42.743819
Sp9		-3.4004794	-2.6764647
Spata2		-2.6735755	-2.8146926
Spty2d1		-4.4237544	-2.7236412
Sqstm1		-2.4136595	-2.5374478
Srgn		-2.0364139	-2.7796213
Srp9		-2.546132	-2.8161606
Srsf5		-2.35386	-2.0205665
Ssbp3		-2.9441289	-4.3657613

Stat5a		-3.7984344	-3.2839336
Stat5b		-2.3971916	-2.7453571
Stk17b		-4.1280935	-2.3286117
Stx5a		-2.4354122	-2.1910531
Syf2		-2.8095458	-2.3144883
Taf4b		-3.5766442	-2.1072338
Taf7		-3.1043447	-2.0571419
Tatdn2		-3.7337819	-3.0370579
Tax1bp1		-2.9324439	-2.5332049
Tbc1d15		-4.492751	-3.8478333
Tbc1d30		-6.143954	-11.650943
Tbc1d32		-3.1053559	-23.664438
Tcp11l2		-6.7458142	-4.7357877
Tdg		-4.4756389	-4.2342768
Tdp2		-3.0107936	-2.1230974
Tex30		-2.6883679	-2.5342763
Tfb2m		-3.2773014	-2.1345772
Tgfbr2		-2.2287158	-2.499168
Tgif2		-4.1235244	-3.6091588
Thumpd1		-2.0878424	-2.077319
Tiparp		-4.8845637	-4.1511843
Tiprl		-2.9634518	-2.6641503
Tmem2		-3.9927785	-7.6585118
Tmem87b		-3.0759089	-2.421875
Tnfrsf10b		-3.1784842	-4.1743405
Tnfrsf12a		-6.4265031	-7.2021189
Tnfrsf26		-3.6016497	-2.9940503
Tnip1		-4.232836	-3.6328398
Tnn		-7.7371707	-6.0291049
Tob2		-2.7688528	-2.7630287
Tollip		-2.3705082	-2.280999
Traf6		-3.2925678	-2.4067218
Trav16n		-11.135543	-30.507329
Trav2		-9.3130049	-72.88355
Trav3-3		-11.87558	-18.800233
Trav6-1		-15.480442	-45.247283
Trav7-3		-13.659375	-71.751252
Trav7n-4		-14.559267	-21.077215
Trbv1		-22.123265	-59.886264
Trbv2		-6.7375871	-31.180723
Trbv21		-38.454706	-14.878355
Trbv30		-12.437324	-44.710522
Trbv4		-8.3755558	-57.371469

Trdc		-5.9374649	-29.086968
Trp53bp2		-2.7828224	-2.0118771
Trp53inp2		-8.3536432	-8.8440041
Trpm1		-13.885014	-87.196003
Tsc22d1		-3.7756015	-29.354836
Tsc22d3		-3.3872504	-3.7172416
Tshz1		-2.1297976	-2.4280263
Tspyl1		-2.4627223	-2.4388994
Tubb2a		-4.024028	-12.321034
Tubb3		-7.9793182	-48.591747
Ubc		-2.7640249	-3.3354885
Ube2e3		-4.7100169	-2.5827483
Ubn1		-2.4007264	-2.1925629
Ulk1		-2.5553125	-2.7501569
Usp12		-2.3625018	-2.1513776
Usp16		-3.3105187	-2.5513807
Usp2		-29.787448	-26.554069
Usp22		-2.6791892	-2.0006469
Usp28		-3.6487468	-3.8641794
Vamp2		-2.1310644	-2.1075524
Vamp3		-2.2571085	-2.0255176
Vgll4		-3.5664286	-3.5095003
Vipr1		-2.6627787	-2.2966862
Vmn1r58		-65.499741	-49.279279
Wdr45b		-2.4356668	-2.077284
Wfikkn1		-6.3863549	-6.1642053
Wipi2		-2.3085427	-2.2077299
Wls		-3.5941976	-36.322803
Wsb1		-3.4974117	-2.4519326
Xkrx		-4.5731431	-9.5247096
Ypel5		-3.1957128	-2.9901531
Ythdc1		-3.684207	-2.2799717
Zbtb10		-30.262027	-40.44091
Zbtb21		-5.3432619	-3.0760556
Zc2hc1c		-23.11305	-7.0629678
Zc3hc1		-3.4223177	-2.4218214
Zcchc12		-23.30486	-30.644885
Zdbf2		-19.396092	-24.98122
Zfand2a		-8.1149479	-5.0588689
Zfp212		-3.9069953	-3.6315397
Zfp335os		-2.8440037	-2.4482882
Zfp472		-4.0865406	-2.763998
Zfp488		-14.355271	-17.248179

Zfp628		-2.7369061	-2.3655922
Zfp703		-2.0404662	-3.3282848
Zfp868		-3.0041198	-2.3815311
Zfp869		-4.2273538	-2.6688548
Zfp954		-2.6100759	-2.189826
Zfyve28		-5.8640827	-13.312882
Zkscan14		-2.6578402	-2.494786
Zkscan5		-2.8073766	-2.5513574
Zranb1		-3.5232662	-2.3754639
Zrsr1		-3.6266074	-3.2038016
Zscan18		-17.221708	-3.9407976
Zswim4		-4.2292989	-4.3156188
Zyg11b		-2.6539098	-2.3961923
1110032A03Rik		-2.2311519	
1110059G10Rik		-2.3205318	-1.9918218
1700056N10Rik		-5.0805795	
1700109H08Rik		-4.2063961	
2410089E03Rik		-2.1425536	-1.9413597
2700016F22Rik		-2.1443855	-1.8908524
4930506C21Rik		-2.5255584	
4930590J08Rik		-5.7578344	
4930599N23Rik		-2.5832346	
4931413K12Rik		-7.0309234	
9430034N14Rik		-8.899243	
9530052E02Rik		-63.606628	
9830144P21Rik		-2.0893396	-1.6443366
A930004J17Rik		-2.3717122	
Aar2		-2.0843801	-1.9311252
Abhd10		-2.1125799	
Abhd17b		-2.8585929	-1.9963054
Abi1		-2.0326862	
AC113059.1		-4.4126161	
AC122197.1		-6.893824	
AC122539.3		-7.3777834	
AC153969.1		-4.2819221	
AC154510.1		-2.747822	
AC156952.1		-6.4319718	
AC158802.1		-2.1460856	
AC159193.3		-22.416988	
AC159193.5		-2.390924	
Acbd3		-2.1053127	-1.7218605
Acer2		-3.0723684	
Acpp		-3.1643881	

Aff3		-2.0898869	
Aim2		-2.5851658	
Akap8l		-2.0512852	-1.4491585
Akirin2		-2.2097834	-1.8334775
Apol8		-126.54102	
Appbp2		-2.0595933	
Appl2		-2.2687885	
Arap2		-2.1760862	
Arf2		-2.1662336	-1.8724331
Arid4b		-2.1783185	
Arid5a		-2.3256189	
Arnt		-2.0314498	-1.9334031
Atf2		-2.79547	-1.9208498
Atf4		-3.1795134	
Atg12		-2.3742171	
Atg14		-3.5561643	
Atl2		-2.2407426	-1.5104718
AU019823		-2.2778642	
B230303O12Rik		-2.718661	
Bach1		-2.3510151	-1.5402814
Bag3		-3.9908651	
Bap1		-2.0301467	-1.9083164
Bcas2		-2.2846756	-1.850066
Bclaf1		-2.0339749	
Bend4		-27.641062	
Bicdl1		-2.4900349	
Bicra		-2.0842011	
Bnip3l		-2.4261325	-1.8657296
Brix1		-2.2018846	-1.7895635
Bud31		-2.2257276	-1.8859192
Bzw2		-2.0019358	-1.6734777
C230085N15Rik		-9.4544037	
Calm2		-2.0353073	
Camsap2		-2.3586199	
Capn7		-2.2165604	-1.8916633
Casz1		-2.6437779	
Cbll1		-2.2439944	-1.9141489
Ccdc186		-2.131637	-1.9680999
Ccdc43		-2.1480161	
Ccer2		-52.966916	
Ccng2		-2.5050814	-1.969271
Ccnl1		-2.6791787	
Ccnl2		-2.2766039	

Ccr7		-8.2633298	
Cd55		-5.0007742	
Cd69		-6.3306978	
Cdadc1		-2.198229	
Cdc14b		-4.6021502	
Cdc73		-2.3190179	-1.656309
Cebpz		-2.422309	
Cenpl		-2.3113521	-1.8544274
Cenpq		-2.8177957	
Cep120		-2.3434331	-1.7782451
Cep290		-6.0908835	
Cep57		-2.3725558	
Cep63		-2.5162671	
Chd1		-2.4407756	-1.46297
Chd7		-2.5116294	
Chkb		-2.1315068	-1.9587201
Chrna2		-16.893018	
Chst15		-3.098254	
Cir1		-2.0112411	
Cnbp		-2.2782512	-1.9957654
Cnot7		-2.0639388	
Cnst		-2.5939219	-1.6057649
Coq10b		-6.9574078	
Cox11		-2.404052	
Cpeb2		-4.4953151	
Crebbp		-2.3158985	
Crnkl1		-2.0242654	
Csde1		-2.3534795	
Csrnp1		-3.2073193	
Cul3		-2.6491959	-1.8298161
Cux2		-12.884957	
Cwc25		-2.7998473	-1.990257
D430013B06Rik		-6.0570855	
Dcaf10		-2.0259887	-1.6972904
Ddx3y		-2765.3387	
Ddx5		-2.0828723	-1.7628579
Dgke		-2.0048056	
Dhx15		-2.0070827	-1.5769805
Dnajb13		-2.3827551	
Dnajb9		-10.068372	
Dnajc27		-2.0448329	
Dnttip2		-2.2769617	-1.9444053
Dusp1		-20.073872	

Dusp10		-16.408843	
Dusp2		-8.9076246	
Dusp5		-4.9833167	
Dusp6		-2.2919048	
Dusp8		-31.882712	
Dyrk1a		-2.4636679	-1.816135
E2f5		-2.3996903	
E330011M16Rik		-4.1957833	
E430024P14Rik		-7.7574521	
Egr1		-3.9294281	
Eif2a		-2.2220244	
Eif2s3y		-1134.9912	
Eif4a2		-2.2391342	
Eif4g2		-2.3442791	-1.6327947
Eml5		-3.0812794	
Epc1		-2.2936915	
Erbin		-2.1443459	
Ercc6		-2.0521645	
Etaa1		-2.5964208	-1.9164926
Fam126b		-2.0394009	
Fam91a1		-2.5685772	-1.8968089
Far2		-2.7105163	-1.8137387
Fbxo11		-2.0360696	
Fos		-12.968282	
Fosb		-58.183302	
Foxj3		-2.2558994	-1.6398447
Foxn3		-2.1199911	
Foxp1		-2.6616913	-1.9011849
Frat2		-19.280698	
Frmd6		-2.5368397	
Frs2		-2.5386395	-1.9099264
Fyttd1		-2.4586355	-1.668755
Gabrr2		-5.8114866	
Galnt6		-2.0198957	
Galt		-2.1834723	-1.8614492
Gatad2b		-2.0047821	
Gem		-3.4367132	
Ghrl		-5.3484093	
Gimap6		-3.6450712	
Gk5		-3.7552707	
Gm11175		-4.354125	
Gm11455		-5.4365596	
Gm11831		-17.575013	

Gm12167		-2.4637986	-1.9798252
Gm12248		-5.3374865	
Gm12940		-2.2801749	
Gm13008		-2.8305304	
Gm13807		-3.2415195	
Gm14966		-3.1269748	
Gm15337		-2.8469519	
Gm15340		-30.865565	
Gm15898		-16.421899	
Gm15943		-3.9398018	
Gm16185		-3.5350794	
Gm16618		-11.402922	
Gm16853		-6.313928	
Gm19757		-3.5104188	
Gm20417		-2.9925684	
Gm2245		-2.0736046	-1.6847028
Gm26648		-2.5282698	-1.6681694
Gm26699		-3.257651	
Gm26799		-3.8638022	
Gm26809		-3.3693606	
Gm26823		-3.8272867	
Gm26824		-7.5454983	
Gm26882		-2.0867791	
Gm26885		-2.8717158	
Gm28289		-2.008886	
Gm28529		-2.8820332	
Gm28941		-8.6402869	
Gm29361		-2.3650411	
Gm35585		-15.782748	
Gm36931		-3.9050546	-1.8707053
Gm37123		-2.6811312	
Gm37198		-6.3561209	
Gm37248		-11.828785	
Gm37289		-6.9124666	
Gm37352		-9.9539586	
Gm37376		-2.0006715	
Gm37660		-17.385671	
Gm37678		-2.7037818	
Gm37738		-14.952377	
Gm37760		-3.6657988	
Gm37790		-7.6075829	
Gm37939		-13.666902	
Gm37968		-5.1460064	

Gm38033		-6.3800406	
Gm38043		-20.977143	
Gm38062		-16.234344	
Gm38067		-14.338559	
Gm38190		-3.0984472	
Gm38257		-15.052216	
Gm38393		-53.95895	
Gm42462		-9.0446931	
Gm42585		-4.2485321	
Gm42611		-5.9087493	
Gm42632		-14.490227	
Gm42658		-4.8430443	
Gm42659		-4.1972187	
Gm42664		-2.5596004	
Gm42748		-5.1874504	
Gm42829		-17.592619	
Gm42869		-12.447682	
Gm42941		-24.757378	
Gm43024		-16.334715	
Gm43055		-2.9025576	
Gm43300		-6.4451038	
Gm43462		-9.0973147	
Gm43484		-16.589632	
Gm43604		-3.3214739	
Gm43661		-22.834546	
Gm43684		-35.514435	
Gm44164		-6.4194995	
Gm44424		-5.2745947	
Gm44686		-7.7599677	
Gm44775		-7.0637939	
Gm44876		-3.2074008	-1.9125206
Gm45053		-5.5884147	
Gm45081		-24.836775	
Gm45221		-3.6924945	
Gm45222		-7.1026082	
Gm45728		-18.223546	
Gm45738		-6.4901063	
Gm5148		-3.2220397	
Gm614		-4.259228	
Gm9752		-2.2346689	
Gorab		-2.2530228	-1.795615
Got1		-2.7526781	-1.8540624
Gpbp1		-2.8622271	-1.9101767

Gpc1		-2.3069074	
Gramd1c		-3.4451495	
Gramd3		-3.0826561	-1.7275106
H2-Q2		-7.2347281	
Haus3		-2.6057753	-1.8720861
Herpud2		-2.5270154	-1.7943697
Hey1		-7.0607941	
Hps3		-2.0346953	
Ick		-2.1066369	
Ier2		-3.6345685	
Ier5		-3.2630576	
Imp4		-2.1123934	-1.9658579
Impact		-3.5333872	
Inpp5f		-2.4836386	-1.9937774
Insr		-2.0629635	
Ipmk		-2.0037637	-1.6890166
Irs2		-79.576334	
Jmjd1c		-3.4558561	
Jun		-3.1306973	
Junb		-7.9356495	
Jund		-4.4220995	
Junos		-4.1901824	
Kansl1		-2.3025909	-1.6157327
Kat6b		-2.4956099	
Kdm5a		-2.5284509	-1.7404569
Kdm5d		-437.17224	
Kif5b		-2.0321781	
Klf2		-4.8905148	
Klf6		-5.3901908	
Klf9		-4.9936331	
Klhdc2		-4.843596	
Klhl11		-2.0484245	
Klhl12		-2.273305	-1.6077341
Larp7		-2.3981762	-1.7968669
Ldlrap1		-5.6380657	
Lin7c		-2.9380171	-1.8940641
Lncpint		-5.2735321	
Lpin1		-2.1554759	-1.9305093
Lrig3		-21.526863	
Lrrc1		-3.2386165	
Lrrc23		-5.7810558	
Maff		-55.968187	
Map3k1		-2.0955142	

Map3k3		-2.3056006	-1.9227555
Map4k2		-2.0130042	-1.9962682
Map4k4		-2.6028023	-1.831341
Map4k5		-3.1551323	
Mapk6		-2.2035291	
Masp2		-2.4804151	-1.5734059
Mat2b		-2.2446915	-1.7347412
Matr3		-2.5466956	-1.7557812
Mboat7		-2.0583866	-1.9836626
Mbtd1		-2.3981343	
Meioc		-15.472318	
Mepce		-2.0588538	-1.8928773
Mllt3		-2.3602742	-1.549069
Mob1b		-2.5997065	-1.7259741
Mphosph6		-2.1131066	-1.8243959
Mpp5		-2.5209753	-1.8995396
Mrpl9		-2.0106566	-1.9335411
Mrps30		-2.4984107	
Mtmr12		-2.0795678	-1.5869957
Mtpap		-2.087871	
Mynn		-2.3092228	
N4bp2l2		-2.121871	-1.6279334
Nemf		-2.2738105	
Nfatc3		-2.0189445	
Nfkbia		-5.3112858	
Nfkbiz		-4.6536969	
Nim1k		-10.572104	
Nmd3		-2.006391	-1.6542246
Nr4a1		-10.312445	
Nr4a2		-20.50575	
Nr4a3		-25.778463	
Nrde2		-2.0378055	-1.8353412
Nup153		-2.1965844	-1.8522882
Nup98		-2.2843428	-1.9198702
Osbpl9		-2.1620952	-1.8025591
Otud1		-10.893304	
P2ry10		-3.8269116	
Paip2b		-2.1426799	
Parp6		-2.4536776	-1.7497186
Patl1		-2.0134214	
Pcf11		-2.4390113	
Pde12		-2.1519333	-1.8999797
Peli1		-2.2049817	-1.7591885

Per1		-15.214756	
Per2		-3.2465876	
Pex11g		-8.8417331	
Pik3c2a		-2.0541574	-1.8371365
Pithd1		-2.3968625	-1.992514
Plce1		-7.3586461	
Plekha5		-2.3021498	
Plekhg2		-2.0183082	-1.800877
Pogz		-2.150787	-1.5469487
Polr2d		-2.1900298	-1.848675
Polr3f		-2.3194192	-1.6834125
Ppm1l		-2.4944349	
Ppp1r15a		-13.709199	
Ppp2r2a		-2.562769	-1.8279082
Ppp4r2		-2.1261669	
Prkch		-2.2680742	-1.4269811
Prosc		-2.0885438	-1.6207313
Prpf38a		-2.1660522	-1.7617025
Prr7		-3.1174152	
Pstk		-2.2127776	-1.8568452
Ptbp2		-3.3040696	
Rab11fip1		-2.0898598	
Rab4a		-3.4233596	
Ralgps1		-2.7238188	
Ranbp9		-2.1749286	
Rapgef2		-2.0350638	
Rapgef4os2		-2.938281	
Rbak		-2.1990805	
Rbm22		-2.1109051	-1.728656
Rbm27		-2.1698629	-1.6277018
Rbm33		-2.5831977	-1.7605348
Rbpj		-2.0603497	-1.8579675
Rc3h1		-2.1041635	
Rec114		-5.0775287	
Rfx3		-5.6549482	
Rgs1		-3.9158654	
Rgs2		-21.995348	
Rims3		-4.7467282	
Riok3		-2.3947258	-1.9271449
Ripk2		-2.1882147	-1.8832764
Rlf		-2.8160787	-1.9562622
Rnf125		-4.3941336	
Rnf138		-3.1383042	

Rnf145		-2.4538833	-1.8739234
Rnf146		-2.0003523	-1.7326833
Rnf26		-2.1739809	
Rpf1		-2.5160285	-1.9535521
Rps24		-2.0325262	
Rps27		-2.5912594	
Rsbn1		-2.043603	-1.5828522
Rsrc2		-2.0812156	
S1pr1		-5.3808454	
Satb1		-3.383569	-1.9013135
Scaf8		-2.2291546	-1.4961181
Scmh1		-3.6564559	
Sec62		-2.0710452	-1.7426453
Senp6		-2.250711	
Sertad1		-2.036679	
Sesn1		-2.805945	-1.7898432
Setd2		-2.4828064	
Sgk1		-2.2330086	
Sh3pxd2a		-2.057821	
Sidt1		-2.8688145	
Sik1		-23.828124	
Slc15a2		-2.5640726	
Slc25a16		-2.2328297	-1.7680353
Slc25a23		-3.1037836	
Slc25a3		-2.0998326	
Slc25a36		-3.4088469	-1.9384574
Slc35e2		-2.3678998	-1.6408543
Slc44a1		-3.8380204	
Smad4		-2.531781	-1.9117654
Smarca5		-2.0971163	-1.4523201
Smurf1		-2.4035615	
Snhg1		-2.9865251	
Snhg14		-36.140201	
Snrpn		-80.712602	
Snurf		-18.935005	
Spata13		-2.1336464	-1.7725732
Spice1		-2.215666	
Spred1		-3.4663072	
Spred2		-2.794754	-1.6408184
Spry2		-17.315264	
Srfbp1		-3.0326009	-1.7443349
Srrm1		-2.1334194	
Srsf11		-2.1323893	

Srsf12		-4.1808813	
Ssh2		-3.2829877	
Strn3		-2.2402197	-1.6529959
Suco		-2.8034041	-1.7428201
Synj2		-3.9448394	
Tacc1		-2.1961853	-1.3945461
Tacc2		-15.204233	
Taf1d		-2.0085951	
Taf5		-2.3566423	
Taf8		-2.4478616	-1.915231
Tagap		-10.764051	
Tanc1		-2.0833884	-1.8648164
Tdrp		-3.8451407	
Tet1		-5.7916656	
Tex45		-101.11827	
Tfdp2		-2.4660984	
Tgif1		-17.53484	
Timm29		-2.3643196	-1.8838746
Tle4		-2.8114059	-1.6782963
Tlk1		-2.1783241	-1.6756398
Tlk2		-2.6647581	-1.681011
Tmem167b		-2.2061512	
Tmem184a		-3.632734	
Tmem216		-2.013241	
Tmem55b		-2.0678694	-1.9322813
Tmem57		-2.0969331	-1.8420721
Tnfaip3		-53.194252	
Tnks		-2.0718618	-1.6092103
Tom1l1		-2.2266849	
Topors		-2.3703103	
Tra2b		-2.6148377	-1.8595618
Traj30		-16.477852	
Traj49		-15.367809	
Trav19		-17.721664	
Trbj2-1		-9.7388266	
Trbv19		-23.472009	
Trim13		-6.3985408	
Trmo		-2.2642058	-1.8852088
Tsc1		-2.0066836	-1.7265812
Tsc22d2		-2.1475484	
Ttc28		-4.2569006	
Ttc3		-2.1300001	
Twistnb		-2.1790533	-1.8821011

Txnrd3		-3.5258077	
Ubald2		-3.635223	
Ube2b		-2.6469547	-1.7775669
Ube2d3		-2.2330435	
Ube2g1		-2.0080226	
Ube2j1		-2.4295535	-1.80342
Ubxn7		-2.1494498	
Unkl		-2.3733163	-1.9256257
Usp38		-2.1066683	-1.9091909
Vapa		-2.1621583	
Vps37b		-12.913419	
Wac		-3.1291408	-1.9655788
Wasl		-2.4265242	
Wbp11		-2.0569656	-1.7441345
Wipf3		-19.049786	
Yipf4		-2.0851365	
Ypel2		-4.3890513	
Yrdc		-2.2106417	
Ythdf1		-2.1788928	-1.7110357
Zbed5		-6.9580226	
Zbtb11		-2.1595812	
Zbtb17		-2.2373616	-1.9341245
Zc3h12a		-8.9371904	
Zc3hav1		-2.2300294	-1.854591
Zcchc10		-2.9233602	
Zcchc11		-2.208423	
Zfp112		-3.0909012	
Zfp281		-2.2359815	
Zfp354c		-2.1016438	
Zfp36l2		-3.6132936	-1.8654087
Zfp410		-2.2948424	-1.980698
Zfp51		-2.8662126	-1.8242337
Zfp518a		-2.1827781	
Zfp53		-2.586733	-1.9569779
Zfp592		-2.7007299	-1.9281276
Zfp619		-4.0924326	
Zfp827		-12.917209	
Zfp874b		-2.4706228	-1.9498559
Zfp949		-2.1471108	-1.6919505
Zfp951		-2.194434	
Zfp955b		-2.3350232	
Zfp959		-2.0293299	
Zhx2		-2.457424	

Zmym1		-2.35513	-1.8110865
Znrf3		-2.2925217	
Zscan10		-7.1998177	
Zswim6		-2.2055308	
Zzz3		-2.3748142	-1.7496931
1110051M20Rik			-2.0045119
1810043G02Rik			-2.9107276
2310022B05Rik			-2.0095457
2810429I04Rik			-17.380274
4732440D04Rik			-2.9314782
4930426D05Rik			-26.941755
4930426L09Rik			-12.321516
5430400D12Rik			-22.659487
5830405F06Rik			-57.182158
A230005M16Rik			-11.625972
A630014C17Rik			-2.5353188
Aaed1			-2.6962082
Abca3			-2.1661795
Abca5			-6.153348
Abca7			-2.702255
AC122273.2			-2.416668
AC126262.1			-20.109788
Acot11			-8.4544728
Acsl3			-17.571799
Actn2			-35.246858
Adgra3			-19.561566
Ahr			-26.099121
Akr1e1			-7.2904772
Akt3			-7.7354425
Aldh1b1			-3.9509561
Alkbh6			-2.1249132
Als2cl			-4.7580945
Ankrd55			-17.626122
Anxa5			-4.7811939
Apbb2			-35.290888
Arf4			-2.0247319
Arhgap29			-10.987864
Armcx3			-13.762549
Armcx4			-61.166897
Armcx6			-35.545327
Asb14			-5.6428641
Atp6v0c			-2.183925
Atrnl1			-7.7096618

Auts2		-19.621599
B3gnt9		-2.4034083
B4galnt4		-9.8302639
Bcl2a1b		-4.7247409
Begain		-26.295563
Bend5		-6.288278
Bmpr2		-3.1695958
Bmyc		-11.620198
C77080		-4.5645848
Camp		-18.99331
Capn10		-2.0828085
Carns1		-2.4727989
Cask		-13.717147
Casp1		-5.6468639
Ccdc157		-3.1788148
Ccdc88a		-3.7597299
Cd1d1		-2.1134785
Cd53		-2.0784879
Cd83		-5.1389256
Cd86		-4.7238592
Cd9		-12.556297
Cdc42bpa		-11.358809
Cdc42bpb		-4.3984997
Cdc42ep4		-6.9891742
Cdkn1c		-25.833309
Cdr2		-2.1914523
Celsr2		-3.1299674
Cep164		-2.1073167
Cerkl		-20.645381
Cers6		-12.76299
Cfl2		-2.054093
Chn2		-15.706638
Chrm3		-18.020066
Chst2		-13.493384
Clip2		-2.7311455
Cnn3		-14.398933
Col6a3		-45.493122
Cpd		-4.1532959
Cpm		-5.3332945
Cry1		-2.2038139
Csnk1e		-6.5590106
CT030159.3		-3.3304342
CT030161.2		-19.336867

Ctla4		-27.654884
Ctsl		-20.76664
Cyp2s1		-13.251459
Cystm1		-35.991337
Cyth3		-2.1314592
Dapk1		-11.476404
Dcun1d4		-2.7826215
Ddr1		-4.6557516
Dedd2		-2.4928586
Dgka		-2.0434592
Dnajc12		-6.0730397
Dnajc6		-41.850153
Dpp4		-4.9234734
Dtx4		-3.8999844
Dusp12		-2.0554625
E130208F15Rik		-2.4304241
Echdc2		-5.7770416
Egr2		-3.9864054
Eid2		-4.0972377
Elk3		-2.0480785
Emp1		-10.545051
Eng		-22.307751
Epas1		-6.1461778
Epha2		-17.963127
Ephb6		-4.2564571
Erdr1		-4.1565309
Esr1		-21.166233
Etv3		-2.1101318
Fam118a		-2.7877523
Fam129b		-6.3454718
Fam133b		-2.0057757
Fam151a		-9.8168403
Fbrs		-2.0387525
Fgfr1		-34.390653
Fgfrl1		-3.918928
Firre		-4.1990692
Fnbp1l		-4.825613
Foxo4		-2.2319642
Fth1		-2.0744382
Fzd7		-11.841283
Galnt10		-35.181489
Galnt9		-50.420334
Gcsh		-2.2654803

Ggt1		-4.5134315
Ggt5		-5.16423
Glis3		-35.261238
Glul		-2.1294797
Gm10827		-9.3962994
Gm11775		-3.2037826
Gm13561		-13.339683
Gm14085		-2.0591274
Gm16001		-2.8993356
Gm16059		-6.6463127
Gm16161		-4.0820129
Gm17200		-3.0191525
Gm20457		-2.9050653
Gm2238		-4.1457846
Gm26728		-8.9665261
Gm28513		-25.378568
Gm32633		-13.745182
Gm35082		-19.651043
Gm43127		-4.2024424
Gm43267		-19.979861
Gm45059		-4.7776372
Gm45669		-11.564357
Gm7457		-33.528728
Gm7967		-33.802249
Gm9967		-2.1726637
Gna12		-2.0572999
Gnb4		-5.4934081
Gng12		-5.9806808
Gpr3		-18.171487
Gpr68		-18.238816
Gpr83		-14.76198
Gprc5b		-18.060783
Gramd1a		-2.4398708
Grhl1		-4.6909715
Gria3		-26.701098
Gspt2		-25.669773
Gtf2ird1		-8.3029762
Havcr2		-8.7170417
Herpud1		-2.3718728
Hist4h4		-9.1110734
Hoxa7		-21.561987
Hs6st2		-20.316048
Icos		-4.2479399

Id2	[redacted]	-10.277601
Ier5l	[redacted]	-24.98935
Igfbp4	[redacted]	-38.376705
Igsf3	[redacted]	-18.10971
Il21r	[redacted]	-2.4414244
Ildr1	[redacted]	-5.1060305
Inf2	[redacted]	-4.5376985
Iqck	[redacted]	-10.867645
Irf4	[redacted]	-7.400947
Irgq	[redacted]	-2.1726471
Itga4	[redacted]	-13.684881
Itga7	[redacted]	-6.9802778
Itgae	[redacted]	-7.5322333
Itgb7	[redacted]	-4.4530547
Jakmip1	[redacted]	-2.1187499
Kcna2	[redacted]	-11.026222
Kctd12b	[redacted]	-24.519128
Kif3a	[redacted]	-4.8209065
Kifap3	[redacted]	-8.936892
Klf13	[redacted]	-2.417875
Klhl14	[redacted]	-16.570715
Lancl3	[redacted]	-22.053731
Ldhb	[redacted]	-30.446097
Lhfpl3	[redacted]	-20.327884
Lmo4	[redacted]	-3.9734203
Lrrc75b	[redacted]	-23.262668
Lrrn4cl	[redacted]	-8.6526116
Lrrtm2	[redacted]	-31.31803
Ltf	[redacted]	-9.5899644
Lypd6b	[redacted]	-7.1756513
Lztfl1	[redacted]	-2.8541682
Maged1	[redacted]	-5.70542
Magee1	[redacted]	-2.3981332
Maoa	[redacted]	-24.089656
Map1lc3a	[redacted]	-8.3655025
Map7d3	[redacted]	-39.237012
Map9	[redacted]	-24.133499
Marcksl1	[redacted]	-5.3859074
Mast4	[redacted]	-11.54012
Mgst3	[redacted]	-7.0381424
Mical3	[redacted]	-10.145672
Mir155hg	[redacted]	-5.4590471
Mir22hg	[redacted]	-6.7753567

Mitf		-9.1561685
Ms4a6c		-5.2983401
Msl3l2		-40.864306
Msrb2		-8.7482993
Muc3		-21.054264
Nedd4l		-2.6209306
Neurl3		-6.8080639
Nfix		-5.0954692
Ngp		-10.271931
Nipal1		-2.6390116
Nlrc4		-10.81063
Nme4		-3.8469914
Nrbp1		-2.0344475
Nrp1		-4.1783596
Ntn4		-29.281276
Oat		-2.7392047
Ogfod2		-2.4483523
P2rx4		-3.2517896
Pak1		-6.0288742
Palmd		-15.03408
Pcbp3		-25.99261
Pde8a		-2.5673003
Pecam1		-2.5618672
Peg13		-4.174866
Pex16		-2.3099024
Pgap1		-4.0875553
Phf13		-2.1965423
Phlda1		-6.4795254
Phyhd1		-3.073133
Pid1		-4.7087221
Pink1		-2.048921
Pla2g4b		-2.2725738
Plcb4		-6.3592967
Plcl1		-20.325947
Plekho1		-3.3077546
Plxnb1		-3.8933761
Pou2f2		-2.4992223
Ppp1r37		-2.1517784
Pqlc1		-2.1217279
Prex1		-2.0925231
Ptgdr2		-3.3087678
Ptov1		-2.7252332
Pttg1ip		-2.4554692

Qrfp		-13.536482
Rab6b		-23.433609
Rab7b		-5.7506889
Ralb		-4.4318576
Ranbp10		-2.6403845
Ret		-10.588168
Rfx2		-2.6496602
Rgs16		-16.135949
Rnf144a		-6.6563816
Rnf216		-4.2129117
Rpl29		-3.8914626
Rpl39l		-18.790821
Rps6ka2		-2.9159545
Runx1		-2.12995
S100a9		-6.9474726
Scamp1		-31.135665
Sdcbp2		-5.4478559
Sema4f		-2.6924996
Serinc5		-3.965645
Serp2		-28.226899
Serpini1		-2.1071006
Sesn2		-2.084655
Sfn		-2.6260059
Sfr1		-2.0325754
Shb		-27.702864
Slc12a2		-8.1085243
Slc16a10		-22.649464
Slc19a2		-2.1218765
Slc25a13		-11.162325
Slc25a25		-2.2346851
Slc26a10		-2.1500947
Slc2a1		-2.370158
Slc4a7		-4.0443971
Slc6a19		-29.66494
Spata6		-23.799518
Spats2l		-22.709638
Spry1		-18.286379
Sqor		-5.2482708
St3gal3		-2.216928
St6galnac3		-3.0135623
St8sia1		-31.30147
Stk11ip		-2.2740854
Stk38l		-2.1826564

Stx1a		-4.5289197
Sult6b2		-15.667148
Susd4		-36.208103
Syde2		-114.71376
Syt11		-2.8298074
Sytl3		-9.9380868
Tagln		-2.4777624
Tbc1d10a		-2.0384463
Tbc1d17		-2.3602168
Tbc1d19		-33.68696
Tdrkh		-15.332042
Tgfbr3l		-2.0852738
Them4		-3.5784874
Thy1		-20.590874
Timp2		-5.427826
Tle1		-62.818395
Tm2d2		-2.1023167
Tmem248		-2.0184652
Tmem50b		-2.602189
Tmtc2		-23.393194
Tnfrsf25		-14.602086
Tnfrsf9		-5.9072959
Tns1		-8.149374
Tns2		-7.039583
Trat1		-10.064147
Trav12-1		-21.737183
Trav12-3		-25.83331
Trav16		-15.667151
Trav16d-dv11		-58.475264
Trav21-dv12		-30.933797
Trav5-1		-36.284603
Trav6-2		-43.07115
Trav6-4		-28.931384
Trav6-6		-18.036339
Trav6d-5		-43.715101
Trav6n-6		-21.779522
Trav7-1		-71.333928
Trav7-4		-27.044167
Trav7d-3		-21.903345
Trav7d-4		-27.186682
Trav8d-1		-38.461653
Trav8n-2		-47.03399
Trav9-1		-15.675283

Trbj1-2		-27.342276
Trbv12-1		-165.91679
Trbv20		-34.813116
Trbv26		-66.355588
Trbv29		-81.493649
Trbv3		-50.641015
Trbv31		-17.890766
Trbv5		-62.943878
Trdv2-2		-15.66715
Trerf1		-8.8580949
Trib1		-9.6260612
Trib2		-18.370219
Trim62		-2.2222966
Trp53i11		-3.3139989
Tspan13		-4.9992251
Tspan5		-2.2220989
Tspan6		-19.511788
Tspyl2		-2.0185399
Tubb2b		-17.954137
Tusc1		-17.233048
Twsg1		-2.043867
Txnip		-2.5443934
Uchl1		-15.667148
Unc45a		-2.5951408
Use1		-2.0689326
Usf2		-2.2694856
Usp6nl		-4.4098681
Utf1		-17.82747
Utp14b		-36.196397
Vangl2		-12.956625
Vps18		-2.2175845
Vsir		-2.3404666
Wnt5b		-26.221004
Ypel3		-2.068775
Zan		-19.363841
Zbed3		-5.5693377
Zc3h12c		-7.8726125
Zcchc14		-18.441965
Zfp503		-17.241188
Zfp516		-6.9390441
Zfp69		-2.7725647
Zfp821		-2.0130179
Zfp947		-5.7179721

Gdpd3		-2

Supporting Information for Fig. 7f - Heat maps showing overlap between promoter hypomethylation (meth. diff. ≥ 30%; p(MWU) < 0.05) and gene expression (FC ≥ 3, p < 0.05 by DESeq) in Dnmt3b+/- (3b+/-; n=3) and Dnmt3a$^{\Delta/\Delta}$ (3a$^{\Delta/\Delta}$; n=3) lymphomas when compared to control CD8+ T- cells (C; n = 2).

		Dnmt3b+/-		Dnmt3aΔ/Δ	
	C	meth	expr	meth	expr
Sumo2		-0.70	2.02	-0.85	2.09
Serpinb9		-0.47	3.18	-0.73	2.10
Crtap		-0.38	5.14	-0.40	2.92
H2-T3		-0.40	3.33	-0.60	3.15
Coro2a		-0.39	3.84	-0.81	4.54
Trip13		-0.44	7.66	-0.65	6.63
Fam19a3		-0.43	18.30	-0.67	7.12
Stat1		-0.58	9.29	-0.75	8.09
2310001H17Rik		-0.70	9.21	-0.72	8.62
Rrm1		-0.46	2.12		
Acaca		-0.45	2.24		
Evi2a		-0.47	2.64	-0.65	
Nav1		-0.82	5.61	-0.84	
Wdr95		-0.49	5.66		
Cdkl3		-0.50	7.53		
Slc7a7		-0.34	8.15		
Itga1		-0.76	11.25	-0.79	
Sept8		-0.40	14.62		
Aif1		-0.76	16.23	-0.81	
Gbp2b		-0.66	23.33	-0.63	
Pros1		-0.37	33.82		
Bmp10		-0.47	37.39		

Supporting Information for Fig. 8d - Change in methylation and region coordinates underlying presented heat maps

DMR coordinates			methylation difference		
chrom	start	end	+/+	Δ/Δ	CI/CI
chr9	77961342	77961384		-0.340872	
chr6	119580010	119580064		-0.975645	
chr6	121097860	121097896		-0.955229	
chr6	133958421	133958435		-0.911358	
chr2	161638286	161638343		-0.796116	
chr2	163090240	163090258		-0.763698	
chr9	80097053	80097074		-0.727661	
chr3	103789042	103789093		-0.726589	
chr6	82382406	82382490		-0.715852	
chr6	105827197	105827216		-0.710202	
chr10	45020047	45020097		-0.697763	
chrX	166566775	166566804		-0.697727	
chr16	5193943	5193993		-0.689135	
chr2	156095031	156095059		-0.68863	
chr12	54353162	54353201		-0.688475	
chr6	136935655	136935735		-0.687382	
chr17	29041247	29041290		-0.686533	
chr12	70074925	70074934		-0.680931	
chr2	161454386	161454410		-0.679039	
chr16	7463092	7463124		-0.677568	
chr6	71354915	71355005		-0.677102	
chr6	100467107	100467142		-0.676209	
chr9	59200109	59200183		-0.675484	
chr3	133359733	133359772		-0.67472	
chr6	79507734	79507779		-0.672377	
chr15	25987872	25987931		-0.671121	
chr7	28177586	28177664		-0.670278	
chr8	41562181	41562201		-0.669909	
chr1	67404237	67404294		-0.667765	
chr1	164429964	164430007		-0.667275	
chr6	78232146	78232220		-0.663604	
chr15	82034167	82034209		-0.663186	
chr2	160198802	160198834		-0.660064	
chr5	96197538	96197693		-0.65921	
chr1	166124015	166124054		-0.659074	
chr9	104921455	104921490		-0.656558	
chrX	13286105	13286179		-0.65117	

chrX	7972748	7972816	[redacted]	-0.649542	[redacted]
chr4	46463614	46463645	[redacted]	-0.648083	[redacted]
chr7	74531304	74531330	[redacted]	-0.645659	[redacted]
chr13	100493775	100493801	[redacted]	-0.645098	[redacted]
chr9	31208295	31208410	[redacted]	-0.644955	[redacted]
chr5	104013370	104013434	[redacted]	-0.640945	[redacted]
chr10	21897086	21897163	[redacted]	-0.640268	[redacted]
chr12	101053505	101053563	[redacted]	-0.639389	[redacted]
chr4	42979249	42979304	[redacted]	-0.638953	[redacted]
chr6	94395002	94395062	[redacted]	-0.636311	[redacted]
chr11	11750592	11750599	[redacted]	-0.63154	[redacted]
chr11	98528474	98528482	[redacted]	-0.629968	[redacted]
chr1	36910624	36910636	[redacted]	-0.629482	[redacted]
chr11	115478714	115478737	[redacted]	-0.629145	[redacted]
chr9	57830406	57830461	[redacted]	-0.627374	[redacted]
chr15	55543116	55543140	[redacted]	-0.627109	[redacted]
chr15	80578048	80578087	[redacted]	-0.626823	[redacted]
chr6	83568940	83568981	[redacted]	-0.626818	[redacted]
chr3	94917033	94917050	[redacted]	-0.626402	[redacted]
chr8	57579275	57579287	[redacted]	-0.626078	[redacted]
chr10	92934323	92934399	[redacted]	-0.625907	[redacted]
chr9	82964301	82964352	[redacted]	-0.625379	[redacted]
chr19	44312317	44312379	[redacted]	-0.625351	[redacted]
chr1	60760749	60760792	[redacted]	-0.624499	[redacted]
chr10	91161541	91161586	[redacted]	-0.623784	[redacted]
chr15	55018544	55018565	[redacted]	-0.622015	[redacted]
chr18	74998591	74998650	[redacted]	-0.621359	[redacted]
chr10	21154683	21154723	[redacted]	-0.621096	[redacted]
chr1	184955712	184955767	[redacted]	-0.618935	[redacted]
chr2	154647928	154647981	[redacted]	-0.618145	[redacted]
chr1	185401420	185401462	[redacted]	-0.61731	[redacted]
chr16	8728820	8728849	[redacted]	-0.617001	[redacted]
chr6	119115175	119115206	[redacted]	-0.614865	[redacted]
chr6	15823971	15824004	[redacted]	-0.612447	[redacted]
chr13	38213736	38213780	[redacted]	-0.610399	[redacted]
chr15	7997038	7997064	[redacted]	-0.608406	[redacted]
chr1	97952631	97952694	[redacted]	-0.606344	[redacted]
chr9	71885204	71885262	[redacted]	-0.605423	[redacted]
chr10	112634773	112634835	[redacted]	-0.604644	[redacted]
chr6	71356651	71356717	[redacted]	-0.603536	[redacted]
chr7	66680087	66680144	[redacted]	-0.603323	[redacted]
chr5	21708336	21708379	[redacted]	-0.602551	[redacted]
chr12	73659752	73659802	[redacted]	-0.600463	[redacted]

chr9	46179423	46179475	[redacted]	-0.6004	[redacted]
chr9	50483296	50483308	[redacted]	-0.599437	[redacted]
chr13	45874798	45874858	[redacted]	-0.598017	[redacted]
chr14	13990691	13990732	[redacted]	-0.597364	[redacted]
chr7	81202368	81202433	[redacted]	-0.597196	[redacted]
chr10	116463897	116463976	[redacted]	-0.596441	[redacted]
chr18	84864031	84864072	[redacted]	-0.594259	[redacted]
chr2	156631098	156631132	[redacted]	-0.594086	[redacted]
chr11	98491186	98491205	[redacted]	-0.593973	[redacted]
chr12	12417315	12417381	[redacted]	-0.593543	[redacted]
chr3	103781763	103781812	[redacted]	-0.593486	[redacted]
chr19	55543435	55543511	[redacted]	-0.593348	[redacted]
chr19	11571857	11571952	[redacted]	-0.592488	[redacted]
chr1	107568819	107568840	[redacted]	-0.592487	[redacted]
chr10	117817782	117817826	[redacted]	-0.591713	[redacted]
chr7	68076391	68076488	[redacted]	-0.59143	[redacted]
chr18	34918342	34918413	[redacted]	-0.5905	[redacted]
chr6	71356032	71356099	[redacted]	-0.590405	[redacted]
chr4	116960370	116960439	[redacted]	-0.590357	[redacted]
chr2	58580274	58580344	[redacted]	-0.590072	[redacted]
chr5	122468845	122468888	[redacted]	-0.589854	[redacted]
chr16	38357850	38357903	[redacted]	-0.589815	[redacted]
chr6	54587158	54587239	[redacted]	-0.589567	[redacted]
chr9	69523086	69523156	[redacted]	-0.589121	[redacted]
chr16	18575789	18575849	[redacted]	-0.586896	[redacted]
chr18	70603159	70603181	[redacted]	-0.586833	[redacted]
chr6	121048595	121048668	[redacted]	-0.586581	[redacted]
chr11	87452207	87452262	[redacted]	-0.585844	[redacted]
chr15	96696172	96696211	[redacted]	-0.585741	[redacted]
chr5	113843983	113844059	[redacted]	-0.585647	[redacted]
chr17	65924365	65924381	[redacted]	-0.585553	[redacted]
chr4	41295096	41295135	[redacted]	-0.58501	[redacted]
chr7	100705672	100705703	[redacted]	-0.58394	[redacted]
chr4	59328169	59328191	[redacted]	-0.583929	[redacted]
chr3	102076036	102076081	[redacted]	-0.58375	[redacted]
chr19	36329461	36329499	[redacted]	-0.583312	[redacted]
chr13	93245704	93245730	[redacted]	-0.582974	[redacted]
chr16	95742857	95742890	[redacted]	-0.582693	[redacted]
chr6	134318368	134318409	[redacted]	-0.581681	[redacted]
chr12	112565861	112565901	[redacted]	-0.581083	[redacted]
chr17	63460145	63460188	[redacted]	-0.580699	[redacted]
chr1	178401153	178401172	[redacted]	-0.580132	[redacted]
chr13	12409240	12409302	[redacted]	-0.579912	[redacted]

chr11	70879820	70879846	[redacted]	-0.579808	[redacted]
chr11	51633354	51633367	[redacted]	-0.57905	[redacted]
chr2	153907700	153907736	[redacted]	-0.577021	[redacted]
chr5	149252998	149253074	[redacted]	-0.576254	[redacted]
chr4	8867213	8867237	[redacted]	-0.576127	[redacted]
chr11	100392363	100392398	[redacted]	-0.575896	[redacted]
chr1	69155566	69155630	[redacted]	-0.575409	[redacted]
chr7	66731357	66731399	[redacted]	-0.575203	[redacted]
chr10	92384399	92384452	[redacted]	-0.57484	[redacted]
chr6	128766650	128766721	[redacted]	-0.574396	[redacted]
chr15	94422653	94422709	[redacted]	-0.574116	[redacted]
chr8	60910557	60910629	[redacted]	-0.573989	[redacted]
chr5	65876911	65876966	[redacted]	-0.573188	[redacted]
chrX	130498898	130498963	[redacted]	-0.573129	[redacted]
chr12	59152405	59152452	[redacted]	-0.571934	[redacted]
chr19	56441312	56441368	[redacted]	-0.571627	[redacted]
chr2	70329456	70329540	[redacted]	-0.571006	[redacted]
chr8	30569570	30569620	[redacted]	-0.570502	[redacted]
chr5	60598544	60598606	[redacted]	-0.570345	[redacted]
chr19	26730688	26730750	[redacted]	-0.570266	[redacted]
chrX	85268333	85268395	[redacted]	-0.570229	[redacted]
chr2	137373751	137373782	[redacted]	-0.569973	[redacted]
chr4	141696968	141697008	[redacted]	-0.56981	[redacted]
chr3	108123652	108123703	[redacted]	-0.569435	[redacted]
chr6	78580385	78580409	[redacted]	-0.569031	[redacted]
chr17	86034274	86034319	[redacted]	-0.56876	[redacted]
chr1	43433095	43433165	[redacted]	-0.568383	[redacted]
chr1	66826626	66826717	[redacted]	-0.568206	[redacted]
chr9	112272668	112272710	[redacted]	-0.567854	[redacted]
chr17	5120657	5120804	[redacted]	-0.567319	[redacted]
chr16	22188303	22188320	[redacted]	-0.566857	[redacted]
chr15	57521055	57521135	[redacted]	-0.566769	[redacted]
chr2	68842127	68842167	[redacted]	-0.566239	[redacted]
chr5	124414724	124414742	[redacted]	-0.566228	[redacted]
chr18	6201715	6201813	[redacted]	-0.565488	[redacted]
chr3	52280517	52280564	[redacted]	-0.564946	[redacted]
chr12	69204325	69204388	[redacted]	-0.564734	[redacted]
chr3	138224712	138224775	[redacted]	-0.564153	[redacted]
chr18	5249397	5249423	[redacted]	-0.563478	[redacted]
chr15	51798483	51798576	[redacted]	-0.56301	[redacted]
chr3	143944707	143944776	[redacted]	-0.562794	[redacted]
chr2	31451221	31451294	[redacted]	-0.562714	[redacted]
chr15	85469788	85469859	[redacted]	-0.561709	[redacted]

chr18	46915009	46915057	[redacted]	-0.561075	[redacted]
chr8	107528645	107528691	[redacted]	-0.560453	[redacted]
chr2	72046282	72046305	[redacted]	-0.559755	[redacted]
chr17	29039520	29039561	[redacted]	-0.559565	[redacted]
chr1	37687096	37687136	[redacted]	-0.559268	[redacted]
chr17	5343712	5343821	[redacted]	-0.558738	[redacted]
chr10	61232092	61232137	[redacted]	-0.558063	[redacted]
chr11	105444074	105444089	[redacted]	-0.557692	[redacted]
chr2	71124246	71124300	[redacted]	-0.557638	[redacted]
chr12	107991930	107991998	[redacted]	-0.55736	[redacted]
chr17	83807949	83807975	[redacted]	-0.555894	[redacted]
chr15	36998960	36999042	[redacted]	-0.555768	[redacted]
chr5	35262874	35262898	[redacted]	-0.555719	[redacted]
chr6	76222952	76222985	[redacted]	-0.555686	[redacted]
chr9	109943054	109943082	[redacted]	-0.555376	[redacted]
chr14	105068642	105068715	[redacted]	-0.555347	[redacted]
chr9	46127108	46127168	[redacted]	-0.554976	[redacted]
chr2	131252094	131252133	[redacted]	-0.55488	[redacted]
chr11	115478476	115478534	[redacted]	-0.554326	[redacted]
chr5	124111006	124111023	[redacted]	-0.554043	[redacted]
chr2	44987742	44987815	[redacted]	-0.553935	[redacted]
chr6	128699351	128699412	[redacted]	-0.553817	[redacted]
chr7	133634908	133634964	[redacted]	-0.553793	[redacted]
chr2	180688561	180688681	[redacted]	-0.553074	[redacted]
chr10	89820124	89820168	[redacted]	-0.553036	[redacted]
chr6	38345523	38345539	[redacted]	-0.552907	[redacted]
chr7	114154215	114154248	[redacted]	-0.552309	[redacted]
chr14	105260703	105260742	[redacted]	-0.552183	[redacted]
chr9	41885116	41885194	[redacted]	-0.551382	[redacted]
chr4	62679024	62679095	[redacted]	-0.550703	[redacted]
chr16	24340667	24340681	[redacted]	-0.55055	[redacted]
chr2	142714322	142714388	[redacted]	-0.550277	[redacted]
chr17	63472160	63472223	[redacted]	-0.549703	[redacted]
chr19	35082500	35082550	[redacted]	-0.548288	[redacted]
chr5	65884585	65884610	[redacted]	-0.548182	[redacted]
chr17	47947371	47947436	[redacted]	-0.547987	[redacted]
chr2	75684509	75684551	[redacted]	-0.547885	[redacted]
chr13	46909259	46909315	[redacted]	-0.547736	[redacted]
chr4	131889551	131889617	[redacted]	-0.547431	[redacted]
chr14	66142854	66142887	[redacted]	-0.546779	[redacted]
chr1	13383582	13383645	[redacted]	-0.545619	[redacted]
chr7	49411973	49412044	[redacted]	-0.545501	[redacted]
chr9	77960973	77960992	[redacted]	-0.5455	[redacted]

chr14	107385924	107385929	[redacted]	-0.545273	[redacted]
chr5	92362564	92362620	[redacted]	-0.544878	[redacted]
chr6	54499044	54499137	[redacted]	-0.544632	[redacted]
chr10	26517791	26517830	[redacted]	-0.544582	[redacted]
chr3	130323067	130323116	[redacted]	-0.544404	[redacted]
chr3	144558946	144558996	[redacted]	-0.544375	[redacted]
chr1	155943582	155943674	[redacted]	-0.544081	[redacted]
chr13	48927411	48927452	[redacted]	-0.54328	[redacted]
chr8	56828681	56828733	[redacted]	-0.54327	[redacted]
chr18	42004050	42004103	[redacted]	-0.542619	[redacted]
chr9	49243565	49243584	[redacted]	-0.542503	[redacted]
chr9	84249061	84249114	[redacted]	-0.542442	[redacted]
chr8	60374658	60374724	[redacted]	-0.542254	[redacted]
chr17	44637693	44637770	[redacted]	-0.541586	[redacted]
chr15	20746536	20746613	[redacted]	-0.541445	[redacted]
chr11	52342953	52342995	[redacted]	-0.541279	[redacted]
chr17	29038732	29038777	[redacted]	-0.540642	[redacted]
chr9	59199814	59199921	[redacted]	-0.540147	[redacted]
chr3	51557178	51557217	[redacted]	-0.539779	[redacted]
chr3	152272660	152272687	[redacted]	-0.539527	[redacted]
chr9	70581427	70581502	[redacted]	-0.539195	[redacted]
chr2	94143084	94143183	[redacted]	-0.538619	[redacted]
chr6	92298759	92298791	[redacted]	-0.538376	[redacted]
chr7	29131730	29131756	[redacted]	-0.538366	[redacted]
chr2	121972783	121972850	[redacted]	-0.537309	[redacted]
chr6	121449199	121449247	[redacted]	-0.537281	[redacted]
chr12	91149871	91149948	[redacted]	-0.537147	[redacted]
chr10	91566534	91566609	[redacted]	-0.537015	[redacted]
chr17	56118186	56118220	[redacted]	-0.536515	[redacted]
chr13	43865422	43865433	[redacted]	-0.536441	[redacted]
chr6	108224067	108224086	[redacted]	-0.535651	[redacted]
chr3	122009970	122010135	[redacted]	-0.535566	[redacted]
chr19	29378823	29378853	[redacted]	-0.535493	[redacted]
chr5	97091007	97091116	[redacted]	-0.53547	[redacted]
chr17	10354390	10354413	[redacted]	-0.534813	[redacted]
chr16	45999524	45999582	[redacted]	-0.53346	[redacted]
chr17	27781274	27781316	[redacted]	-0.533454	[redacted]
chr3	142901356	142901406	[redacted]	-0.533277	[redacted]
chr5	55082453	55082503	[redacted]	-0.533083	[redacted]
chr7	73585789	73585802	[redacted]	-0.533015	[redacted]
chr15	59046929	59047012	[redacted]	-0.533003	[redacted]
chr10	91420288	91420307	[redacted]	-0.532662	[redacted]
chr9	111033612	111033666	[redacted]	-0.532503	[redacted]

chr10	68153714	68153735	[redacted]	-0.532126	[redacted]
chr9	77957844	77957903	[redacted]	-0.531735	[redacted]
chr15	60433392	60433441	[redacted]	-0.531671	[redacted]
chr13	43865486	43865556	[redacted]	-0.531335	[redacted]
chr18	5270056	5270111	[redacted]	-0.531197	[redacted]
chr18	5745696	5745717	[redacted]	-0.531188	[redacted]
chr3	158012315	158012390	[redacted]	-0.530503	[redacted]
chr14	31403168	31403222	[redacted]	-0.530421	[redacted]
chr19	44817190	44817207	[redacted]	-0.530379	[redacted]
chr19	58214776	58214822	[redacted]	-0.530001	[redacted]
chr3	49840495	49840549	[redacted]	-0.529967	[redacted]
chr10	117557384	117557425	[redacted]	-0.529328	[redacted]
chr8	126816325	126816343	[redacted]	-0.528976	[redacted]
chr18	5596727	5596780	[redacted]	-0.528545	[redacted]
chr12	99359900	99359936	[redacted]	-0.528506	[redacted]
chr17	87115510	87115612	[redacted]	-0.527916	[redacted]
chr14	86956359	86956404	[redacted]	-0.52786	[redacted]
chrX	12909579	12909628	[redacted]	-0.527775	[redacted]
chr17	6747578	6747610	[redacted]	-0.527709	[redacted]
chr6	4666615	4666678	[redacted]	-0.52753	[redacted]
chr16	33169369	33169447	[redacted]	-0.527524	[redacted]
chrX	153233339	153233359	[redacted]	-0.527207	[redacted]
chr2	139967078	139967139	[redacted]	-0.527176	[redacted]
chr11	68445074	68445127	[redacted]	-0.527154	[redacted]
chr4	106382004	106382037	[redacted]	-0.527004	[redacted]
chr3	30102325	30102388	[redacted]	-0.526292	[redacted]
chr1	38499156	38499168	[redacted]	-0.526271	[redacted]
chr1	86594762	86594822	[redacted]	-0.525912	[redacted]
chr5	124633075	124633125	[redacted]	-0.525641	[redacted]
chr2	118448476	118448533	[redacted]	-0.525577	[redacted]
chr2	58467178	58467236	[redacted]	-0.525376	[redacted]
chr11	78684041	78684107	[redacted]	-0.525342	[redacted]
chr2	94392878	94392965	[redacted]	-0.525159	[redacted]
chr9	82984566	82984610	[redacted]	-0.525069	[redacted]
chr2	68980893	68980947	[redacted]	-0.524344	[redacted]
chr5	115109542	115109575	[redacted]	-0.524266	[redacted]
chr3	135604466	135604519	[redacted]	-0.524097	[redacted]
chr18	53800899	53800940	[redacted]	-0.524002	[redacted]
chr14	55656583	55656629	[redacted]	-0.523922	[redacted]
chr5	37964308	37964366	[redacted]	-0.523729	[redacted]
chr6	85938442	85938493	[redacted]	-0.523511	[redacted]
chr5	148541363	148541424	[redacted]	-0.523301	[redacted]
chr6	140313597	140313619	[redacted]	-0.523046	[redacted]

chr3	7163298	7163342	[redacted]	-0.522943	[redacted]
chr1	106318648	106318691	[redacted]	-0.522897	[redacted]
chr11	23195182	23195264	[redacted]	-0.522864	[redacted]
chr7	79393663	79393689	[redacted]	-0.522747	[redacted]
chrX	169987492	169987551	[redacted]	-0.522594	[redacted]
chr11	68437941	68437962	[redacted]	-0.522455	[redacted]
chr16	92648845	92648904	[redacted]	-0.522408	[redacted]
chr8	45670880	45670962	[redacted]	-0.521925	[redacted]
chr2	72618772	72618823	[redacted]	-0.521847	[redacted]
chr15	59537551	59537607	[redacted]	-0.521674	[redacted]
chr11	101359530	101359663	[redacted]	-0.521669	[redacted]
chr9	107375425	107375439	[redacted]	-0.521546	[redacted]
chr3	108831687	108831734	[redacted]	-0.521421	[redacted]
chr7	90472436	90472492	[redacted]	-0.521418	[redacted]
chr13	114600599	114600619	[redacted]	-0.521319	[redacted]
chr12	54789085	54789145	[redacted]	-0.521161	[redacted]
chr12	107974830	107974863	[redacted]	-0.521154	[redacted]
chr11	100806585	100806606	[redacted]	-0.52114	[redacted]
chr12	107975172	107975258	[redacted]	-0.520937	[redacted]
chrX	13293426	13293450	[redacted]	-0.520913	[redacted]
chr11	74740170	74740200	[redacted]	-0.520764	[redacted]
chr11	86541234	86541273	[redacted]	-0.520704	[redacted]
chr14	29980327	29980354	[redacted]	-0.520695	[redacted]
chr3	105741396	105741470	[redacted]	-0.520687	[redacted]
chr4	116405782	116405830	[redacted]	-0.520393	[redacted]
chr3	100160757	100160810	[redacted]	-0.520137	[redacted]
chr1	13576276	13576361	[redacted]	-0.519979	[redacted]
chr17	12975875	12975927	[redacted]	-0.519971	[redacted]
chr15	38289513	38289546	[redacted]	-0.519654	[redacted]
chr3	135524723	135524765	[redacted]	-0.519594	[redacted]
chr16	43868536	43868553	[redacted]	-0.519032	[redacted]
chr12	73617052	73617071	[redacted]	-0.518922	[redacted]
chr2	12175693	12175754	[redacted]	-0.518735	[redacted]
chr13	53391873	53391918	[redacted]	-0.518137	[redacted]
chr7	123001892	123001935	[redacted]	-0.518113	[redacted]
chr12	77560249	77560316	[redacted]	-0.51784	[redacted]
chr11	106526932	106527020	[redacted]	-0.517778	[redacted]
chr10	128046737	128046784	[redacted]	-0.517741	[redacted]
chr9	56244749	56244812	[redacted]	-0.517702	[redacted]
chr19	10694662	10694734	[redacted]	-0.517635	[redacted]
chr11	96956491	96956578	[redacted]	-0.51753	[redacted]
chr15	72715254	72715289	[redacted]	-0.517423	[redacted]
chr5	148360562	148360606	[redacted]	-0.517396	[redacted]

chr9	31872241	31872301	[redacted]	-0.517336	[redacted]
chr9	94839239	94839338	[redacted]	-0.5173	[redacted]
chr2	160495785	160495831	[redacted]	-0.516967	[redacted]
chr18	34541833	34541876	[redacted]	-0.516932	[redacted]
chr14	60712269	60712333	[redacted]	-0.516926	[redacted]
chr13	45896333	45896496	[redacted]	-0.516837	[redacted]
chrX	49393440	49393512	[redacted]	-0.516502	[redacted]
chr6	49803934	49804005	[redacted]	-0.516322	[redacted]
chr9	108629604	108629701	[redacted]	-0.516066	[redacted]
chr2	158969482	158969600	[redacted]	-0.515667	[redacted]
chr11	87241090	87241137	[redacted]	-0.515357	[redacted]
chr1	128749469	128749482	[redacted]	-0.515356	[redacted]
chrX	100911145	100911159	[redacted]	-0.515102	[redacted]
chr19	6376387	6376441	[redacted]	-0.514814	[redacted]
chr10	4988931	4989001	[redacted]	-0.514761	[redacted]
chr8	53655801	53655873	[redacted]	-0.51459	[redacted]
chr11	113664118	113664147	[redacted]	-0.514508	[redacted]
chr9	58250376	58250436	[redacted]	-0.514479	[redacted]
chr7	126367001	126367166	[redacted]	-0.514423	[redacted]
chr10	43131165	43131215	[redacted]	-0.514329	[redacted]
chr11	6843459	6843491	[redacted]	-0.51428	[redacted]
chr1	168686736	168686804	[redacted]	-0.514115	[redacted]
chr6	67278108	67278160	[redacted]	-0.513939	[redacted]
chr11	63170159	63170207	[redacted]	-0.513894	[redacted]
chr5	20515421	20515483	[redacted]	-0.513741	[redacted]
chr7	79886834	79886898	[redacted]	-0.513725	[redacted]
chr9	22690629	22690687	[redacted]	-0.513563	[redacted]
chr18	49523173	49523204	[redacted]	-0.513445	[redacted]
chr10	12485271	12485307	[redacted]	-0.513398	[redacted]
chr6	58196806	58196832	[redacted]	-0.513088	[redacted]
chr10	115689075	115689150	[redacted]	-0.512937	[redacted]
chr8	13988870	13988904	[redacted]	-0.512909	[redacted]
chr2	79137624	79137665	[redacted]	-0.512826	[redacted]
chr2	68924712	68924761	[redacted]	-0.512171	[redacted]
chr13	51675670	51675777	[redacted]	-0.512088	[redacted]
chr1	134566196	134566217	[redacted]	-0.512007	[redacted]
chr3	53833781	53833850	[redacted]	-0.512006	[redacted]
chr18	42564214	42564256	[redacted]	-0.51158	[redacted]
chrX	101644369	101644410	[redacted]	-0.51148	[redacted]
chr15	96545237	96545287	[redacted]	-0.51139	[redacted]
chr2	5635625	5635655	[redacted]	-0.511242	[redacted]
chr12	92232369	92232448	[redacted]	-0.511079	[redacted]
chr8	116990745	116990758	[redacted]	-0.510911	[redacted]

chr6	32970857	32970908	[redacted]	-0.51085	[redacted]
chr8	36905393	36905448	[redacted]	-0.510765	[redacted]
chr7	99875450	99875507	[redacted]	-0.510763	[redacted]
chr16	50590647	50590678	[redacted]	-0.510662	[redacted]
chr19	57590566	57590653	[redacted]	-0.510583	[redacted]
chr3	28111438	28111471	[redacted]	-0.510561	[redacted]
chr6	146831553	146831591	[redacted]	-0.510263	[redacted]
chr11	103943719	103943810	[redacted]	-0.509946	[redacted]
chr10	42826194	42826211	[redacted]	-0.509856	[redacted]
chr3	103789290	103789430	[redacted]	-0.509414	[redacted]
chr5	52571725	52571775	[redacted]	-0.509364	[redacted]
chr2	84836865	84836964	[redacted]	-0.50932	[redacted]
chr6	51472777	51472819	[redacted]	-0.509306	[redacted]
chr4	58043065	58043103	[redacted]	-0.509276	[redacted]
chr10	97059631	97059674	[redacted]	-0.509217	[redacted]
chr7	71368706	71368727	[redacted]	-0.509209	[redacted]
chr1	35676389	35676449	[redacted]	-0.509187	[redacted]
chr6	28790344	28790457	[redacted]	-0.509168	[redacted]
chr11	18076312	18076350	[redacted]	-0.508915	[redacted]
chr12	34613796	34613847	[redacted]	-0.508873	[redacted]
chr6	133191830	133191871	[redacted]	-0.50855	[redacted]
chr6	17926162	17926219	[redacted]	-0.508529	[redacted]
chr10	28524943	28525008	[redacted]	-0.508207	[redacted]
chr10	122728866	122728908	[redacted]	-0.508175	[redacted]
chr9	7363832	7363903	[redacted]	-0.508109	[redacted]
chr11	58715360	58715401	[redacted]	-0.507681	[redacted]
chr18	84865750	84865863	[redacted]	-0.507582	[redacted]
chr10	115434997	115435014	[redacted]	-0.507524	[redacted]
chr17	50202201	50202222	[redacted]	-0.507316	[redacted]
chr10	62660463	62660530	[redacted]	-0.507056	[redacted]
chr17	3194039	3194130	[redacted]	-0.50704	[redacted]
chr13	24300581	24300652	[redacted]	-0.506988	[redacted]
chr4	118008226	118008288	[redacted]	-0.506864	[redacted]
chr10	110952486	110952510	[redacted]	-0.506843	[redacted]
chr5	136097705	136097755	[redacted]	-0.506704	[redacted]
chr1	106096912	106096943	[redacted]	-0.506699	[redacted]
chr8	78562612	78562666	[redacted]	-0.506431	[redacted]
chr2	3525813	3525843	[redacted]	-0.50642	[redacted]
chr12	59265173	59265209	[redacted]	-0.506307	[redacted]
chr11	80139350	80139419	[redacted]	-0.506025	[redacted]
chr6	137150357	137150446	[redacted]	-0.505725	[redacted]
chr5	57977678	57977763	[redacted]	-0.505672	[redacted]
chr10	58811862	58811916	[redacted]	-0.505646	[redacted]

chr12	110492255	110492323	[redacted]	-0.505427	[redacted]
chr17	15804198	15804239	[redacted]	-0.505223	[redacted]
chr12	70829868	70829924	[redacted]	-0.504994	[redacted]
chr4	141934435	141934461	[redacted]	-0.504899	[redacted]
chr10	120909602	120909657	[redacted]	-0.50476	[redacted]
chr1	144167191	144167270	[redacted]	-0.504718	[redacted]
chr13	45886070	45886084	[redacted]	-0.504626	[redacted]
chr7	127825538	127825565	[redacted]	-0.504355	[redacted]
chr11	5900135	5900179	[redacted]	-0.504294	[redacted]
chr9	40348977	40349037	[redacted]	-0.504156	[redacted]
chr14	26891498	26891507	[redacted]	-0.503792	[redacted]
chr10	43534850	43534874	[redacted]	-0.503758	[redacted]
chr15	12160571	12160631	[redacted]	-0.503732	[redacted]
chr14	119252486	119252550	[redacted]	-0.503717	[redacted]
chr4	35217376	35217432	[redacted]	-0.503612	[redacted]
chr1	179991981	179992002	[redacted]	-0.503274	[redacted]
chr14	107660219	107660290	[redacted]	-0.503154	[redacted]
chr3	19458754	19458790	[redacted]	-0.50294	[redacted]
chr15	27993730	27993792	[redacted]	-0.502869	[redacted]
chr6	51213864	51213947	[redacted]	-0.502719	[redacted]
chr6	48518301	48518317	[redacted]	-0.502569	[redacted]
chr5	107265754	107265796	[redacted]	-0.502385	[redacted]
chrX	142732727	142732749	[redacted]	-0.502236	[redacted]
chrX	100627800	100627865	[redacted]	-0.502214	[redacted]
chr7	36837637	36837701	[redacted]	-0.502206	[redacted]
chr13	54082035	54082057	[redacted]	-0.502124	[redacted]
chr6	50618210	50618286	[redacted]	-0.502092	[redacted]
chr6	99281218	99281257	[redacted]	-0.502059	[redacted]
chr18	84868464	84868577	[redacted]	-0.50203	[redacted]
chr8	71442568	71442686	[redacted]	-0.501983	[redacted]
chr1	168717517	168717549	[redacted]	-0.501983	[redacted]
chr9	57822906	57823011	[redacted]	-0.501858	[redacted]
chr11	66547965	66548021	[redacted]	-0.501801	[redacted]
chr3	59263167	59263236	[redacted]	-0.501586	[redacted]
chr10	93241450	93241469	[redacted]	-0.501514	[redacted]
chr10	118400158	118400212	[redacted]	-0.501476	[redacted]
chr6	88163158	88163288	[redacted]	-0.501473	[redacted]
chr6	115546346	115546398	[redacted]	-0.501427	[redacted]
chr19	16174048	16174110	[redacted]	-0.501369	[redacted]
chr7	130435899	130435988	[redacted]	-0.501349	[redacted]
chr12	113207911	113207957	[redacted]	-0.501183	[redacted]
chr5	36774748	36774814	[redacted]	-0.50093	[redacted]
chr4	151980272	151980361	[redacted]	-0.500882	[redacted]

chr10	111233562	111233610	[redacted]	-0.500772	[redacted]
chr5	73205806	73205881	[redacted]	-0.500445	[redacted]
chr2	145716635	145716688	[redacted]	-0.500361	[redacted]
chr15	96308916	96308946	[redacted]	-0.500334	[redacted]
chr2	145561171	145561210	[redacted]	-0.500201	[redacted]
chr5	74325578	74325616	[redacted]	-0.499998	[redacted]
chr7	80555917	80555970	[redacted]	-0.499628	[redacted]
chr7	6945053	6945089	[redacted]	-0.4996	[redacted]
chr3	126703533	126703586	[redacted]	-0.499401	[redacted]
chr6	51462461	51462516	[redacted]	-0.499306	[redacted]
chr5	66248960	66249006	[redacted]	-0.49926	[redacted]
chr2	113989201	113989253	[redacted]	-0.499232	[redacted]
chr16	52867847	52867892	[redacted]	-0.498938	[redacted]
chr15	96696428	96696476	[redacted]	-0.498819	[redacted]
chr19	30703407	30703480	[redacted]	-0.498771	[redacted]
chr9	58587187	58587195	[redacted]	-0.498752	[redacted]
chr17	7190299	7190376	[redacted]	-0.498434	[redacted]
chr11	98516148	98516155	[redacted]	-0.498378	[redacted]
chr11	118246054	118246155	[redacted]	-0.498345	[redacted]
chr12	107932367	107932408	[redacted]	-0.498241	[redacted]
chr18	23911388	23911444	[redacted]	-0.498088	[redacted]
chr8	113418647	113418718	[redacted]	-0.497926	[redacted]
chr5	21290048	21290111	[redacted]	-0.497899	[redacted]
chr2	71507023	71507070	[redacted]	-0.497504	[redacted]
chr14	24597339	24597406	[redacted]	-0.49744	[redacted]
chr6	37929402	37929468	[redacted]	-0.496965	[redacted]
chr1	150449161	150449212	[redacted]	-0.49681	[redacted]
chr8	46935928	46935963	[redacted]	-0.496589	[redacted]
chr14	87039885	87039957	[redacted]	-0.496486	[redacted]
chr16	6688265	6688328	[redacted]	-0.496289	[redacted]
chr12	83727195	83727234	[redacted]	-0.495897	[redacted]
chr9	32746894	32746931	[redacted]	-0.495672	[redacted]
chr3	53256629	53256655	[redacted]	-0.495667	[redacted]
chr8	25934914	25934952	[redacted]	-0.495397	[redacted]
chr6	7843563	7843618	[redacted]	-0.495181	[redacted]
chr2	11166908	11166992	[redacted]	-0.495033	[redacted]
chr2	4869562	4869607	[redacted]	-0.494699	[redacted]
chr2	93736746	93736770	[redacted]	-0.494693	[redacted]
chr1	169519328	169519370	[redacted]	-0.494607	[redacted]
chr4	124814563	124814636	[redacted]	-0.494551	[redacted]
chr15	96356799	96356946	[redacted]	-0.49453	[redacted]
chr4	108663735	108663828	[redacted]	-0.494304	[redacted]
chr6	89109534	89109599	[redacted]	-0.494163	[redacted]

chr1	58724656	58724722	[redacted]	-0.494156	[redacted]
chr11	100817524	100817584	[redacted]	-0.494117	[redacted]
chr7	39555071	39555114	[redacted]	-0.494014	[redacted]
chr1	54647822	54647885	[redacted]	-0.493982	[redacted]
chr7	122954766	122954802	[redacted]	-0.493979	[redacted]
chr6	139758811	139758831	[redacted]	-0.493899	[redacted]
chr6	49269043	49269120	[redacted]	-0.493809	[redacted]
chr17	4765390	4765452	[redacted]	-0.493797	[redacted]
chr2	6545602	6545662	[redacted]	-0.493644	[redacted]
chr7	55914404	55914414	[redacted]	-0.493472	[redacted]
chr6	143171737	143171810	[redacted]	-0.493461	[redacted]
chr1	38491003	38491033	[redacted]	-0.493441	[redacted]
chr1	173074411	173074492	[redacted]	-0.493356	[redacted]
chr13	41090259	41090351	[redacted]	-0.493226	[redacted]
chr17	4992466	4992506	[redacted]	-0.493212	[redacted]
chr10	122760211	122760267	[redacted]	-0.492781	[redacted]
chr4	108944090	108944117	[redacted]	-0.49267	[redacted]
chr6	51541267	51541332	[redacted]	-0.492662	[redacted]
chrX	20562261	20562331	[redacted]	-0.492466	[redacted]
chr15	96265998	96266117	[redacted]	-0.492443	[redacted]
chr8	35833500	35833547	[redacted]	-0.492357	[redacted]
chr13	102875432	102875634	[redacted]	-0.4923	[redacted]
chr12	59016950	59017029	[redacted]	-0.492257	[redacted]
chr2	118567189	118567224	[redacted]	-0.492091	[redacted]
chr19	32498921	32498991	[redacted]	-0.492037	[redacted]
chr10	4600318	4600390	[redacted]	-0.491831	[redacted]
chr9	63715428	63715582	[redacted]	-0.491466	[redacted]
chr7	127894428	127894439	[redacted]	-0.491249	[redacted]
chr10	117815800	117815830	[redacted]	-0.491038	[redacted]
chr10	93407812	93407872	[redacted]	-0.490925	[redacted]
chr9	62637247	62637277	[redacted]	-0.490731	[redacted]
chr6	92511236	92511287	[redacted]	-0.490668	[redacted]
chr13	14186829	14186894	[redacted]	-0.49059	[redacted]
chr3	127478450	127478503	[redacted]	-0.490466	[redacted]
chr8	26032857	26032927	[redacted]	-0.490381	[redacted]
chr17	59159458	59159511	[redacted]	-0.490369	[redacted]
chr6	128654853	128654896	[redacted]	-0.490344	[redacted]
chr2	107229535	107229614	[redacted]	-0.490241	[redacted]
chr16	92762339	92762423	[redacted]	-0.490163	[redacted]
chr18	70571388	70571449	[redacted]	-0.489843	[redacted]
chr5	20496396	20496465	[redacted]	-0.489809	[redacted]
chr13	36926494	36926569	[redacted]	-0.48973	[redacted]
chr10	116542370	116542420	[redacted]	-0.489549	[redacted]

chr11	58202747	58202785	[redacted]	-0.489467	[redacted]
chr15	93216179	93216187	[redacted]	-0.489256	[redacted]
chr1	137931753	137931794	[redacted]	-0.489235	[redacted]
chr7	73630490	73630582	[redacted]	-0.489212	[redacted]
chr2	154515487	154515539	[redacted]	-0.489072	[redacted]
chr10	42832070	42832127	[redacted]	-0.488954	[redacted]
chr12	118411406	118411450	[redacted]	-0.488944	[redacted]
chr2	69085384	69085491	[redacted]	-0.48888	[redacted]
chr18	50034517	50034526	[redacted]	-0.488701	[redacted]
chr1	43461823	43461882	[redacted]	-0.488692	[redacted]
chr15	59530052	59530125	[redacted]	-0.488674	[redacted]
chr15	94422963	94422993	[redacted]	-0.488631	[redacted]
chr12	82946368	82946411	[redacted]	-0.488489	[redacted]
chrX	17525018	17525063	[redacted]	-0.488435	[redacted]
chr10	91014314	91014358	[redacted]	-0.48838	[redacted]
chrX	8053891	8053932	[redacted]	-0.488366	[redacted]
chr19	9234980	9235042	[redacted]	-0.488322	[redacted]
chr2	121112401	121112444	[redacted]	-0.488079	[redacted]
chr12	71159345	71159381	[redacted]	-0.487956	[redacted]
chr7	89986148	89986238	[redacted]	-0.487792	[redacted]
chr16	38496052	38496101	[redacted]	-0.487729	[redacted]
chr5	65620679	65620690	[redacted]	-0.487601	[redacted]
chr11	6197571	6197596	[redacted]	-0.487574	[redacted]
chr18	65447790	65447828	[redacted]	-0.487472	[redacted]
chr7	43759374	43759389	[redacted]	-0.487316	[redacted]
chr6	104051276	104051334	[redacted]	-0.487141	[redacted]
chrX	99882995	99883071	[redacted]	-0.48714	[redacted]
chr1	102192099	102192166	[redacted]	-0.487116	[redacted]
chr2	61263838	61263870	[redacted]	-0.487114	[redacted]
chr1	191372491	191372552	[redacted]	-0.487099	[redacted]
chr14	59737917	59737951	[redacted]	-0.487065	[redacted]
chr17	86880347	86880409	[redacted]	-0.486908	[redacted]
chr19	56896209	56896284	[redacted]	-0.486769	[redacted]
chr6	133261108	133261131	[redacted]	-0.486576	[redacted]
chr17	31845945	31845999	[redacted]	-0.486452	[redacted]
chr13	60346290	60346357	[redacted]	-0.486052	[redacted]
chr9	56919614	56919654	[redacted]	-0.486022	[redacted]
chr13	17616285	17616370	[redacted]	-0.486005	[redacted]
chr1	44057791	44057838	[redacted]	-0.485978	[redacted]
chr15	98738301	98738363	[redacted]	-0.485918	[redacted]
chr4	40253384	40253451	[redacted]	-0.485714	[redacted]
chr2	104006871	104006905	[redacted]	-0.485623	[redacted]
chr4	127292812	127292859	[redacted]	-0.485542	[redacted]

chr15	97706452	97706544	[redacted]	-0.485465	[redacted]
chr10	84007819	84007877	[redacted]	-0.485369	[redacted]
chr4	44721866	44721946	[redacted]	-0.485328	[redacted]
chr7	30663817	30663849	[redacted]	-0.484917	[redacted]
chr2	104406420	104406515	[redacted]	-0.484746	[redacted]
chr5	66138738	66138758	[redacted]	-0.484586	[redacted]
chr14	63177108	63177142	[redacted]	-0.484549	[redacted]
chr14	86800147	86800182	[redacted]	-0.484083	[redacted]
chr7	90139620	90139687	[redacted]	-0.483927	[redacted]
chr6	101433869	101433907	[redacted]	-0.483911	[redacted]
chr3	133096205	133096223	[redacted]	-0.483866	[redacted]
chr5	72823418	72823464	[redacted]	-0.483454	[redacted]
chr14	87266970	87267011	[redacted]	-0.483298	[redacted]
chr3	94801885	94801927	[redacted]	-0.483293	[redacted]
chr19	41835678	41835714	[redacted]	-0.482954	[redacted]
chr2	5629586	5629652	[redacted]	-0.482824	[redacted]
chr16	94373474	94373533	[redacted]	-0.48282	[redacted]
chr1	40217211	40217261	[redacted]	-0.482734	[redacted]
chr19	32648921	32648941	[redacted]	-0.482696	[redacted]
chr12	37776357	37776399	[redacted]	-0.482648	[redacted]
chr10	117057419	117057492	[redacted]	-0.482573	[redacted]
chr14	60745573	60745604	[redacted]	-0.482418	[redacted]
chr5	147437696	147437772	[redacted]	-0.482341	[redacted]
chr6	50762439	50762465	[redacted]	-0.482325	[redacted]
chr7	123001418	123001477	[redacted]	-0.482282	[redacted]
chr15	87530529	87530609	[redacted]	-0.482248	[redacted]
chr5	23524394	23524501	[redacted]	-0.482243	[redacted]
chr13	99310648	99310694	[redacted]	-0.482208	[redacted]
chr13	97407262	97407317	[redacted]	-0.482109	[redacted]
chrX	105870009	105870081	[redacted]	-0.482081	[redacted]
chr16	96285373	96285459	[redacted]	-0.482067	[redacted]
chr15	100004371	100004446	[redacted]	-0.481997	[redacted]
chrX	130088534	130088562	[redacted]	-0.481626	[redacted]
chr11	117772485	117772525	[redacted]	-0.481557	[redacted]
chr6	37726870	37726919	[redacted]	-0.481294	[redacted]
chr19	26608804	26608871	[redacted]	-0.481029	[redacted]
chr13	109408505	109408568	[redacted]	-0.480779	[redacted]
chr13	99246457	99246475	[redacted]	-0.480722	[redacted]
chr15	12629217	12629291	[redacted]	-0.480578	[redacted]
chr3	39313007	39313089	[redacted]	-0.480478	[redacted]
chr10	81127195	81127227	[redacted]	-0.480395	[redacted]
chr14	122225402	122225451	[redacted]	-0.480388	[redacted]
chr5	35336295	35336333	[redacted]	-0.480023	[redacted]

chr14	120314598	120314765	[redacted]	-0.480009	[redacted]
chr4	107975020	107975061	[redacted]	-0.480001	[redacted]
chr4	45028166	45028213	[redacted]	-0.48	[redacted]
chr2	91705664	91705676	[redacted]	-0.479961	[redacted]
chr2	168959597	168959630	[redacted]	-0.479913	[redacted]
chr2	91729452	91729476	[redacted]	-0.479843	[redacted]
chr5	114826926	114826954	[redacted]	-0.479586	[redacted]
chr18	65618000	65618035	[redacted]	-0.479527	[redacted]
chr3	97969636	97969717	[redacted]	-0.4795	[redacted]
chr14	79591324	79591398	[redacted]	-0.479488	[redacted]
chr11	71970191	71970229	[redacted]	-0.47936	[redacted]
chr1	190834312	190834345	[redacted]	-0.479315	[redacted]
chr14	66251513	66251564	[redacted]	-0.479314	[redacted]
chr9	40771785	40771838	[redacted]	-0.47931	[redacted]
chr11	46429674	46429690	[redacted]	-0.479224	[redacted]
chr12	75161998	75162045	[redacted]	-0.478955	[redacted]
chr9	32925236	32925316	[redacted]	-0.478915	[redacted]
chr5	96934596	96934682	[redacted]	-0.478864	[redacted]
chr13	4327476	4327526	[redacted]	-0.478799	[redacted]
chr1	179183346	179183401	[redacted]	-0.478791	[redacted]
chr5	90942792	90942830	[redacted]	-0.478787	[redacted]
chr9	86418802	86418833	[redacted]	-0.478607	[redacted]
chr12	75851496	75851551	[redacted]	-0.478479	[redacted]
chr10	13997570	13997652	[redacted]	-0.478425	[redacted]
chr12	110416906	110416928	[redacted]	-0.478407	[redacted]
chr16	54584009	54584071	[redacted]	-0.478192	[redacted]
chr2	132132565	132132597	[redacted]	-0.478091	[redacted]
chr5	86794676	86794731	[redacted]	-0.478065	[redacted]
chr5	138417480	138417531	[redacted]	-0.477846	[redacted]
chr18	63839133	63839197	[redacted]	-0.477693	[redacted]
chr10	111526234	111526313	[redacted]	-0.477626	[redacted]
chr16	43806914	43806981	[redacted]	-0.47752	[redacted]
chr8	111143558	111143590	[redacted]	-0.477488	[redacted]
chrX	48162859	48162879	[redacted]	-0.477436	[redacted]
chr9	86633768	86633783	[redacted]	-0.477297	[redacted]
chr17	46883533	46883651	[redacted]	-0.477188	[redacted]
chr11	80549160	80549360	[redacted]	-0.477168	[redacted]
chr18	54140700	54140783	[redacted]	-0.476947	[redacted]
chr3	78865022	78865100	[redacted]	-0.476922	[redacted]
chr6	42379171	42379215	[redacted]	-0.476748	[redacted]
chrX	52547522	52547592	[redacted]	-0.476665	[redacted]
chr5	8983745	8983766	[redacted]	-0.476509	[redacted]
chr13	92056665	92056742	[redacted]	-0.476447	[redacted]

chr11	32186437	32186485	[redacted]	-0.476417	[redacted]
chr7	72518137	72518208	[redacted]	-0.476274	[redacted]
chr14	57111323	57111395	[redacted]	-0.476192	[redacted]
chr19	53599618	53599650	[redacted]	-0.476023	[redacted]
chrX	57792144	57792182	[redacted]	-0.475982	[redacted]
chr7	125473131	125473152	[redacted]	-0.475946	[redacted]
chr14	73195151	73195251	[redacted]	-0.475764	[redacted]
chr4	99667186	99667240	[redacted]	-0.475733	[redacted]
chr4	59875003	59875039	[redacted]	-0.475722	[redacted]
chr18	41442520	41442578	[redacted]	-0.475569	[redacted]
chr3	112985529	112985593	[redacted]	-0.475556	[redacted]
chr19	36358494	36358529	[redacted]	-0.475439	[redacted]
chr15	35938590	35938666	[redacted]	-0.475339	[redacted]
chr4	68915459	68915529	[redacted]	-0.475088	[redacted]
chr17	47825868	47825912	[redacted]	-0.475042	[redacted]
chr7	65984499	65984558	[redacted]	-0.474982	[redacted]
chr6	125220157	125220310	[redacted]	-0.474977	[redacted]
chr6	133427138	133427196	[redacted]	-0.474836	[redacted]
chr5	52851278	52851323	[redacted]	-0.474796	[redacted]
chr13	77211000	77211052	[redacted]	-0.474791	[redacted]
chr7	122999615	122999641	[redacted]	-0.47475	[redacted]
chr3	95271651	95271680	[redacted]	-0.474699	[redacted]
chr8	69037934	69037983	[redacted]	-0.474688	[redacted]
chr15	34909713	34909751	[redacted]	-0.474666	[redacted]
chr10	110742910	110743000	[redacted]	-0.474644	[redacted]
chr6	120760269	120760327	[redacted]	-0.474493	[redacted]
chr2	105597285	105597340	[redacted]	-0.474462	[redacted]
chr1	62151728	62151792	[redacted]	-0.474426	[redacted]
chr11	44762697	44762749	[redacted]	-0.474418	[redacted]
chr7	126751927	126751953	[redacted]	-0.474301	[redacted]
chr9	47817743	47817803	[redacted]	-0.474163	[redacted]
chr19	47881504	47881582	[redacted]	-0.474149	[redacted]
chr14	47350925	47351015	[redacted]	-0.474115	[redacted]
chr10	109839087	109839112	[redacted]	-0.474021	[redacted]
chr2	59903550	59903623	[redacted]	-0.474003	[redacted]
chr15	36206882	36206957	[redacted]	-0.473938	[redacted]
chr15	99462966	99462988	[redacted]	-0.473905	[redacted]
chr2	53019403	53019461	[redacted]	-0.473799	[redacted]
chr11	5655638	5655723	[redacted]	-0.473745	[redacted]
chr17	63016752	63016822	[redacted]	-0.473723	[redacted]
chr19	46390798	46390812	[redacted]	-0.473684	[redacted]
chr5	29743431	29743468	[redacted]	-0.473639	[redacted]
chr1	132630665	132630712	[redacted]	-0.473591	[redacted]

chr17	78933837	78933880	[redacted]	-0.47352	[redacted]
chr19	5834591	5834705	[redacted]	-0.473338	[redacted]
chr10	6906163	6906235	[redacted]	-0.473324	[redacted]
chr1	178520101	178520170	[redacted]	-0.473035	[redacted]
chr16	4541191	4541245	[redacted]	-0.472887	[redacted]
chr6	48826341	48826372	[redacted]	-0.47273	[redacted]
chr4	59276800	59276817	[redacted]	-0.472654	[redacted]
chr6	17095275	17095327	[redacted]	-0.472621	[redacted]
chr2	152374732	152374764	[redacted]	-0.472576	[redacted]
chr15	39796136	39796215	[redacted]	-0.47252	[redacted]
chr6	125264583	125264601	[redacted]	-0.472324	[redacted]
chr9	72711913	72711981	[redacted]	-0.472266	[redacted]
chr9	22123468	22123529	[redacted]	-0.47217	[redacted]
chr2	59314663	59314708	[redacted]	-0.472037	[redacted]
chr2	172616315	172616366	[redacted]	-0.471716	[redacted]
chrX	93429561	93429583	[redacted]	-0.471704	[redacted]
chr6	82526216	82526313	[redacted]	-0.471656	[redacted]
chr6	136626753	136626816	[redacted]	-0.471566	[redacted]
chr9	122635658	122635710	[redacted]	-0.471505	[redacted]
chr10	45048175	45048229	[redacted]	-0.471469	[redacted]
chr2	151809310	151809331	[redacted]	-0.471413	[redacted]
chr17	8857711	8857743	[redacted]	-0.471353	[redacted]
chr13	33776021	33776065	[redacted]	-0.471145	[redacted]
chr15	36802975	36803021	[redacted]	-0.471077	[redacted]
chr7	111045332	111045386	[redacted]	-0.471	[redacted]
chr1	181198873	181198923	[redacted]	-0.470962	[redacted]
chr6	136012956	136013013	[redacted]	-0.470837	[redacted]
chr2	65173388	65173495	[redacted]	-0.47076	[redacted]
chr2	72676949	72677001	[redacted]	-0.470705	[redacted]
chr13	43607275	43607341	[redacted]	-0.470657	[redacted]
chrX	93760406	93760472	[redacted]	-0.470654	[redacted]
chrX	39421749	39421811	[redacted]	-0.470567	[redacted]
chr2	104885617	104885653	[redacted]	-0.470503	[redacted]
chr1	128594888	128594925	[redacted]	-0.470419	[redacted]
chr11	35223978	35224002	[redacted]	-0.470391	[redacted]
chr5	105569528	105569602	[redacted]	-0.47019	[redacted]
chr6	142506058	142506089	[redacted]	-0.470152	[redacted]
chr11	76390946	76391006	[redacted]	-0.470144	[redacted]
chr14	55089737	55089783	[redacted]	-0.46995	[redacted]
chr16	92694175	92694248	[redacted]	-0.469869	[redacted]
chr2	139949841	139949931	[redacted]	-0.469791	[redacted]
chr6	50432591	50432648	[redacted]	-0.469717	[redacted]
chr16	31392854	31392903	[redacted]	-0.469659	[redacted]

chr6	125267445	125267484	[redacted]	-0.469654	[redacted]
chr19	14435553	14435659	[redacted]	-0.469635	[redacted]
chr14	52293731	52293784	[redacted]	-0.469506	[redacted]
chr1	156442487	156442550	[redacted]	-0.469364	[redacted]
chr17	15718867	15718903	[redacted]	-0.469131	[redacted]
chr11	88708532	88708565	[redacted]	-0.469113	[redacted]
chr12	8962595	8962608	[redacted]	-0.468964	[redacted]
chr8	11095385	11095418	[redacted]	-0.46889	[redacted]
chr4	123667212	123667237	[redacted]	-0.468637	[redacted]
chr10	20204711	20204736	[redacted]	-0.468633	[redacted]
chr10	96991806	96991878	[redacted]	-0.468387	[redacted]
chr18	45624323	45624408	[redacted]	-0.46834	[redacted]
chr6	125569311	125569375	[redacted]	-0.46831	[redacted]
chr19	53341768	53341801	[redacted]	-0.468306	[redacted]
chr17	65252707	65252765	[redacted]	-0.468282	[redacted]
chr9	64896788	64896823	[redacted]	-0.468264	[redacted]
chr10	21804194	21804251	[redacted]	-0.468175	[redacted]
chr12	111103320	111103388	[redacted]	-0.467998	[redacted]
chr13	43809582	43809615	[redacted]	-0.467981	[redacted]
chr8	91645415	91645468	[redacted]	-0.467931	[redacted]
chr7	30761091	30761138	[redacted]	-0.467893	[redacted]
chr8	122958990	122959025	[redacted]	-0.467835	[redacted]
chr2	172823108	172823197	[redacted]	-0.467636	[redacted]
chr13	101331180	101331204	[redacted]	-0.467501	[redacted]
chr14	52428524	52428590	[redacted]	-0.467435	[redacted]
chr15	38197638	38197744	[redacted]	-0.467328	[redacted]
chr7	123167255	123167328	[redacted]	-0.467307	[redacted]
chr7	68302273	68302378	[redacted]	-0.467202	[redacted]
chr10	121058916	121058942	[redacted]	-0.467194	[redacted]
chr8	118251725	118251791	[redacted]	-0.467114	[redacted]
chr18	14968958	14968968	[redacted]	-0.466945	[redacted]
chr19	12771016	12771034	[redacted]	-0.466919	[redacted]
chr1	86577527	86577551	[redacted]	-0.466857	[redacted]
chr19	28064189	28064267	[redacted]	-0.46681	[redacted]
chr2	141090083	141090156	[redacted]	-0.466771	[redacted]
chr6	121310057	121310206	[redacted]	-0.466527	[redacted]
chr14	21724596	21724648	[redacted]	-0.466321	[redacted]
chr11	34081321	34081355	[redacted]	-0.466178	[redacted]
chr13	95389962	95390022	[redacted]	-0.465934	[redacted]
chr5	147025164	147025224	[redacted]	-0.465858	[redacted]
chr3	135513391	135513445	[redacted]	-0.465832	[redacted]
chr5	66264156	66264227	[redacted]	-0.465827	[redacted]
chr16	11825005	11825054	[redacted]	-0.4657	[redacted]

chr11	107584781	107584802	[redacted]	-0.465695	[redacted]
chr18	68846819	68846871	[redacted]	-0.465653	[redacted]
chr6	94746681	94746754	[redacted]	-0.465641	[redacted]
chr13	33001939	33001998	[redacted]	-0.465615	[redacted]
chr15	97605627	97605673	[redacted]	-0.465577	[redacted]
chr15	58125737	58125784	[redacted]	-0.465575	[redacted]
chr12	110395903	110395931	[redacted]	-0.465504	[redacted]
chr11	90665815	90665853	[redacted]	-0.465488	[redacted]
chr1	37002105	37002180	[redacted]	-0.465468	[redacted]
chr4	94591620	94591682	[redacted]	-0.465437	[redacted]
chr18	84865576	84865671	[redacted]	-0.465423	[redacted]
chr14	120078271	120078312	[redacted]	-0.465306	[redacted]
chr2	144465576	144465699	[redacted]	-0.465298	[redacted]
chr12	54980104	54980148	[redacted]	-0.465189	[redacted]
chr11	11041671	11041752	[redacted]	-0.465123	[redacted]
chr9	71880305	71880347	[redacted]	-0.464987	[redacted]
chr17	44429955	44429992	[redacted]	-0.464961	[redacted]
chr13	111396223	111396251	[redacted]	-0.464938	[redacted]
chr5	27771278	27771353	[redacted]	-0.464924	[redacted]
chr16	91993103	91993180	[redacted]	-0.464877	[redacted]
chr18	45984489	45984533	[redacted]	-0.464702	[redacted]
chr8	112212101	112212138	[redacted]	-0.464594	[redacted]
chr6	137540523	137540601	[redacted]	-0.464567	[redacted]
chr11	59452272	59452375	[redacted]	-0.464484	[redacted]
chrX	140765763	140765881	[redacted]	-0.464377	[redacted]
chr1	36234318	36234350	[redacted]	-0.464075	[redacted]
chr2	152457672	152457721	[redacted]	-0.464046	[redacted]
chr3	143161323	143161358	[redacted]	-0.464018	[redacted]
chr14	51001910	51001949	[redacted]	-0.46395	[redacted]
chr5	115554982	115555017	[redacted]	-0.463887	[redacted]
chr17	65902462	65902488	[redacted]	-0.463805	[redacted]
chr17	80779598	80779656	[redacted]	-0.463766	[redacted]
chr10	116515834	116515863	[redacted]	-0.463724	[redacted]
chr17	44645502	44645519	[redacted]	-0.463702	[redacted]
chr13	109842381	109842435	[redacted]	-0.463683	[redacted]
chr9	81677593	81677609	[redacted]	-0.463636	[redacted]
chr9	51184394	51184457	[redacted]	-0.463601	[redacted]
chr6	83098129	83098151	[redacted]	-0.463577	[redacted]
chr18	34864497	34864587	[redacted]	-0.463556	[redacted]
chr5	135705130	135705194	[redacted]	-0.463509	[redacted]
chr3	86100727	86100782	[redacted]	-0.463396	[redacted]
chr3	30336626	30336687	[redacted]	-0.463341	[redacted]
chr10	41529481	41529526	[redacted]	-0.463338	[redacted]

chr3	32357837	32357906	[redacted]	-0.463298	[redacted]
chr13	49294196	49294262	[redacted]	-0.463118	[redacted]
chr10	119227479	119227538	[redacted]	-0.463021	[redacted]
chr7	127386292	127386334	[redacted]	-0.46293	[redacted]
chr8	81540297	81540318	[redacted]	-0.462876	[redacted]
chr18	6167356	6167373	[redacted]	-0.462664	[redacted]
chr16	21568548	21568599	[redacted]	-0.462643	[redacted]
chrX	36403405	36403477	[redacted]	-0.462422	[redacted]
chr11	118968148	118968209	[redacted]	-0.462055	[redacted]
chr18	33869092	33869136	[redacted]	-0.462048	[redacted]
chr5	121292978	121293031	[redacted]	-0.461999	[redacted]
chr6	71045640	71045707	[redacted]	-0.461973	[redacted]
chr16	11315587	11315610	[redacted]	-0.461948	[redacted]
chr17	50636119	50636219	[redacted]	-0.461914	[redacted]
chr4	136044541	136044602	[redacted]	-0.461792	[redacted]
chr12	107991594	107991677	[redacted]	-0.461743	[redacted]
chr11	73130825	73130872	[redacted]	-0.461727	[redacted]
chr2	102609678	102609736	[redacted]	-0.461588	[redacted]
chr4	137734244	137734307	[redacted]	-0.461534	[redacted]
chr4	46851539	46851565	[redacted]	-0.461434	[redacted]
chr2	59557236	59557278	[redacted]	-0.461402	[redacted]
chr4	109325538	109325577	[redacted]	-0.461232	[redacted]
chr11	84511710	84511742	[redacted]	-0.461204	[redacted]
chr1	169516597	169516650	[redacted]	-0.461098	[redacted]
chr12	110397049	110397079	[redacted]	-0.460971	[redacted]
chr10	26989029	26989097	[redacted]	-0.460953	[redacted]
chr2	27284573	27284604	[redacted]	-0.460875	[redacted]
chr17	87665211	87665274	[redacted]	-0.460778	[redacted]
chr7	5079848	5079901	[redacted]	-0.460499	[redacted]
chr13	45006730	45006782	[redacted]	-0.460475	[redacted]
chr6	116285032	116285101	[redacted]	-0.460332	[redacted]
chrX	92071688	92071726	[redacted]	-0.460256	[redacted]
chr13	6104628	6104672	[redacted]	-0.460152	[redacted]
chr5	73852446	73852509	[redacted]	-0.460096	[redacted]
chr11	99913604	99913616	[redacted]	-0.460021	[redacted]
chr4	107974610	107974668	[redacted]	-0.459997	[redacted]
chr17	28737100	28737185	[redacted]	-0.459888	[redacted]
chr11	68440258	68440380	[redacted]	-0.459872	[redacted]
chr13	107954071	107954118	[redacted]	-0.459828	[redacted]
chr12	9040291	9040330	[redacted]	-0.459712	[redacted]
chr13	94425335	94425364	[redacted]	-0.45971	[redacted]
chr12	54291663	54291748	[redacted]	-0.459676	[redacted]
chr2	150802694	150802766	[redacted]	-0.459664	[redacted]

chr13	32300357	32300408	[redacted]	-0.45955	[redacted]
chr7	140275242	140275306	[redacted]	-0.459293	[redacted]
chr6	122507154	122507191	[redacted]	-0.45929	[redacted]
chr11	17325571	17325604	[redacted]	-0.459172	[redacted]
chr12	84758691	84758791	[redacted]	-0.459117	[redacted]
chr1	98052662	98052715	[redacted]	-0.459112	[redacted]
chr6	143054273	143054316	[redacted]	-0.45911	[redacted]
chr8	40970338	40970417	[redacted]	-0.459084	[redacted]
chr1	172155899	172155994	[redacted]	-0.458863	[redacted]
chr4	116551362	116551403	[redacted]	-0.458807	[redacted]
chr17	64626829	64626879	[redacted]	-0.45877	[redacted]
chr13	8842090	8842183	[redacted]	-0.458732	[redacted]
chr19	46878444	46878480	[redacted]	-0.458715	[redacted]
chr16	4672133	4672173	[redacted]	-0.458653	[redacted]
chr3	52283309	52283373	[redacted]	-0.458467	[redacted]
chr8	33042887	33042937	[redacted]	-0.458452	[redacted]
chr11	109818681	109818740	[redacted]	-0.458401	[redacted]
chr11	107190048	107190088	[redacted]	-0.458301	[redacted]
chr15	59391118	59391181	[redacted]	-0.458182	[redacted]
chr5	99371283	99371344	[redacted]	-0.458148	[redacted]
chr2	18829403	18829441	[redacted]	-0.45813	[redacted]
chr10	127675252	127675331	[redacted]	-0.458115	[redacted]
chr12	39870047	39870096	[redacted]	-0.458072	[redacted]
chr4	59942833	59942857	[redacted]	-0.458022	[redacted]
chr11	97161560	97161615	[redacted]	-0.457993	[redacted]
chr8	35156405	35156511	[redacted]	-0.457968	[redacted]
chr15	67186359	67186426	[redacted]	-0.457949	[redacted]
chr9	37364730	37364799	[redacted]	-0.457899	[redacted]
chr11	114914410	114914482	[redacted]	-0.457856	[redacted]
chr2	29124134	29124164	[redacted]	-0.457762	[redacted]
chr6	99433369	99433410	[redacted]	-0.45766	[redacted]
chr16	78343958	78343996	[redacted]	-0.457605	[redacted]
chr4	108081443	108081493	[redacted]	-0.457571	[redacted]
chr11	103945011	103945026	[redacted]	-0.457551	[redacted]
chr2	157463030	157463099	[redacted]	-0.457543	[redacted]
chr9	46984119	46984145	[redacted]	-0.457522	[redacted]
chr19	12343214	12343278	[redacted]	-0.457513	[redacted]
chr17	11419994	11420065	[redacted]	-0.45748	[redacted]
chr8	109982909	109982985	[redacted]	-0.457357	[redacted]
chrX	53705255	53705287	[redacted]	-0.457341	[redacted]
chr17	21669665	21669741	[redacted]	-0.457233	[redacted]
chr11	102932885	102932921	[redacted]	-0.457216	[redacted]
chr10	75274646	75274718	[redacted]	-0.457208	[redacted]

chr7	73423112	73423167	[redacted]	-0.457156	[redacted]
chr14	63793443	63793457	[redacted]	-0.457143	[redacted]
chr3	81499024	81499075	[redacted]	-0.456986	[redacted]
chrX	42520698	42520724	[redacted]	-0.456946	[redacted]
chrX	50788137	50788196	[redacted]	-0.456945	[redacted]
chr14	116283938	116283944	[redacted]	-0.456908	[redacted]
chr16	73621148	73621223	[redacted]	-0.456889	[redacted]
chr9	98395955	98396000	[redacted]	-0.456876	[redacted]
chr4	107317163	107317193	[redacted]	-0.456864	[redacted]
chr9	57320513	57320545	[redacted]	-0.456813	[redacted]
chr14	48714156	48714232	[redacted]	-0.456782	[redacted]
chr2	115701402	115701465	[redacted]	-0.456714	[redacted]
chr15	20061891	20061980	[redacted]	-0.456701	[redacted]
chr17	19319794	19319855	[redacted]	-0.456676	[redacted]
chr14	49183787	49183847	[redacted]	-0.456553	[redacted]
chr9	31871633	31871713	[redacted]	-0.456508	[redacted]
chr16	94626228	94626308	[redacted]	-0.456461	[redacted]
chr10	39927426	39927471	[redacted]	-0.456419	[redacted]
chr2	157303170	157303224	[redacted]	-0.456393	[redacted]
chr2	59765650	59765829	[redacted]	-0.456384	[redacted]
chr15	50667515	50667564	[redacted]	-0.456346	[redacted]
chr7	45638479	45638612	[redacted]	-0.45613	[redacted]
chr5	98068927	98068945	[redacted]	-0.456098	[redacted]
chr10	59590579	59590631	[redacted]	-0.456063	[redacted]
chr10	119294066	119294132	[redacted]	-0.456056	[redacted]
chr10	117822544	117822678	[redacted]	-0.456006	[redacted]
chr14	117163519	117163602	[redacted]	-0.455929	[redacted]
chr3	51022573	51022615	[redacted]	-0.455853	[redacted]
chr8	127670990	127671075	[redacted]	-0.455792	[redacted]
chr12	116129997	116130085	[redacted]	-0.455728	[redacted]
chr4	145232982	145232995	[redacted]	-0.455634	[redacted]
chr9	53525843	53525893	[redacted]	-0.455612	[redacted]
chr16	57486956	57486993	[redacted]	-0.455471	[redacted]
chr16	92740543	92740639	[redacted]	-0.455438	[redacted]
chr7	91118520	91118599	[redacted]	-0.455371	[redacted]
chr5	121364092	121364131	[redacted]	-0.455333	[redacted]
chr14	78630095	78630117	[redacted]	-0.455272	[redacted]
chr4	32389747	32389830	[redacted]	-0.455232	[redacted]
chr16	33456978	33457028	[redacted]	-0.455188	[redacted]
chr2	6649971	6650079	[redacted]	-0.455154	[redacted]
chr9	121545864	121545934	[redacted]	-0.45513	[redacted]
chr19	46669841	46669942	[redacted]	-0.455026	[redacted]
chr2	128057030	128057084	[redacted]	-0.454992	[redacted]

chrX	53466163	53466240	[redacted]	-0.454988	[redacted]
chr19	29260576	29260631	[redacted]	-0.45497	[redacted]
chr10	12594976	12595065	[redacted]	-0.454946	[redacted]
chr6	137330481	137330526	[redacted]	-0.454941	[redacted]
chr14	79558278	79558329	[redacted]	-0.454795	[redacted]
chr14	47624305	47624376	[redacted]	-0.454643	[redacted]
chr2	124889680	124889749	[redacted]	-0.454568	[redacted]
chr14	56187708	56187763	[redacted]	-0.454409	[redacted]
chr11	107023461	107023510	[redacted]	-0.4544	[redacted]
chr18	53841212	53841297	[redacted]	-0.454239	[redacted]
chr15	64184183	64184252	[redacted]	-0.454236	[redacted]
chr15	28941003	28941063	[redacted]	-0.454207	[redacted]
chr19	57499097	57499152	[redacted]	-0.45414	[redacted]
chr8	83173424	83173448	[redacted]	-0.454032	[redacted]
chrX	8927194	8927224	[redacted]	-0.45397	[redacted]
chr7	12353271	12353301	[redacted]	-0.45394	[redacted]
chr5	26971891	26971994	[redacted]	-0.453751	[redacted]
chr10	14052874	14052923	[redacted]	-0.453738	[redacted]
chr3	115608167	115608212	[redacted]	-0.453724	[redacted]
chr12	71687600	71687644	[redacted]	-0.453659	[redacted]
chr1	92204789	92204915	[redacted]	-0.453494	[redacted]
chr4	116688287	116688350	[redacted]	-0.453454	[redacted]
chr8	46951914	46951974	[redacted]	-0.45342	[redacted]
chr10	115381113	115381145	[redacted]	-0.453371	[redacted]
chr11	31386455	31386522	[redacted]	-0.453243	[redacted]
chrX	13690903	13690944	[redacted]	-0.453184	[redacted]
chr10	79861916	79861956	[redacted]	-0.453158	[redacted]
chr10	116945793	116945819	[redacted]	-0.453113	[redacted]
chr8	107431389	107431419	[redacted]	-0.452959	[redacted]
chr1	98031883	98031918	[redacted]	-0.452752	[redacted]
chr18	28138111	28138143	[redacted]	-0.4526	[redacted]
chr13	56749101	56749144	[redacted]	-0.452596	[redacted]
chr1	67186289	67186332	[redacted]	-0.452593	[redacted]
chr15	94334840	94334863	[redacted]	-0.452522	[redacted]
chr2	139186968	139187014	[redacted]	-0.452473	[redacted]
chr2	104918034	104918087	[redacted]	-0.45215	[redacted]
chr15	27483836	27483883	[redacted]	-0.45212	[redacted]
chr18	50052011	50052066	[redacted]	-0.452097	[redacted]
chr1	60911332	60911369	[redacted]	-0.452071	[redacted]
chr18	68051350	68051375	[redacted]	-0.452052	[redacted]
chr8	79690158	79690208	[redacted]	-0.452006	[redacted]
chr19	34959647	34959688	[redacted]	-0.452003	[redacted]
chr1	80199109	80199204	[redacted]	-0.451958	[redacted]

chr11	46125283	46125340	[redacted]	-0.451869	[redacted]
chr17	12738941	12739000	[redacted]	-0.45182	[redacted]
chr12	82253744	82253793	[redacted]	-0.451702	[redacted]
chr6	104747795	104747829	[redacted]	-0.451634	[redacted]
chr17	35279420	35279466	[redacted]	-0.451583	[redacted]
chr15	93489645	93489686	[redacted]	-0.451566	[redacted]
chr1	16705499	16705581	[redacted]	-0.451498	[redacted]
chr6	128123000	128123038	[redacted]	-0.451367	[redacted]
chr2	119023283	119023321	[redacted]	-0.45131	[redacted]
chr1	80523980	80524024	[redacted]	-0.451204	[redacted]
chr9	14741115	14741191	[redacted]	-0.451119	[redacted]
chr10	117603774	117603861	[redacted]	-0.451046	[redacted]
chr12	75161833	75161937	[redacted]	-0.450848	[redacted]
chr9	59518858	59518979	[redacted]	-0.450708	[redacted]
chr14	36893495	36893547	[redacted]	-0.45055	[redacted]
chr11	98343583	98343637	[redacted]	-0.450441	[redacted]
chr11	116840100	116840266	[redacted]	-0.450396	[redacted]
chr11	82822221	82822260	[redacted]	-0.450313	[redacted]
chr13	34897543	34897592	[redacted]	-0.450296	[redacted]
chr11	59211632	59211651	[redacted]	-0.450228	[redacted]
chr19	26414983	26415010	[redacted]	-0.450192	[redacted]
chr8	22051574	22051625	[redacted]	-0.450148	[redacted]
chr2	45513882	45513965	[redacted]	-0.450124	[redacted]
chr2	9536521	9536575	[redacted]	-0.450103	[redacted]
chr13	114102211	114102277	[redacted]	-0.450041	[redacted]
chr13	80591987	80592044	[redacted]	-0.449981	[redacted]
chr12	110389458	110389479	[redacted]	-0.449892	[redacted]
chr10	42701563	42701665	[redacted]	-0.449747	[redacted]
chr10	24315781	24315845	[redacted]	-0.449729	[redacted]
chr13	11665923	11666007	[redacted]	-0.44972	[redacted]
chr9	69507070	69507107	[redacted]	-0.449714	[redacted]
chr2	119106149	119106185	[redacted]	-0.449642	[redacted]
chr8	86382009	86382042	[redacted]	-0.449627	[redacted]
chr8	79781455	79781503	[redacted]	-0.449596	[redacted]
chr2	140644788	140644840	[redacted]	-0.449573	[redacted]
chr2	34250462	34250499	[redacted]	-0.449444	[redacted]
chrX	70962541	70962584	[redacted]	-0.449429	[redacted]
chr2	59715704	59715726	[redacted]	-0.449412	[redacted]
chr9	89824744	89824766	[redacted]	-0.449381	[redacted]
chr19	32269309	32269347	[redacted]	-0.449341	[redacted]
chr13	117104497	117104548	[redacted]	-0.449229	[redacted]
chr13	64842943	64843002	[redacted]	-0.449129	[redacted]
chr9	112444941	112445009	[redacted]	-0.448986	[redacted]

chr9	27012770	27012796	[redacted]	-0.448951	[redacted]
chr11	103888688	103888750	[redacted]	-0.448903	[redacted]
chr7	51108016	51108024	[redacted]	-0.448897	[redacted]
chr13	97494550	97494590	[redacted]	-0.448852	[redacted]
chr13	55221869	55221940	[redacted]	-0.448723	[redacted]
chr15	55331688	55331739	[redacted]	-0.448645	[redacted]
chrUn_	9600	9664	[redacted]	-0.448573	[redacted]
chr2	139952900	139952961	[redacted]	-0.448569	[redacted]
chr7	45484488	45484563	[redacted]	-0.448547	[redacted]
chr3	143384859	143384912	[redacted]	-0.448543	[redacted]
chr13	24474222	24474258	[redacted]	-0.448417	[redacted]
chr5	43676843	43676916	[redacted]	-0.448401	[redacted]
chr11	83228068	83228124	[redacted]	-0.448305	[redacted]
chr11	78693310	78693458	[redacted]	-0.448112	[redacted]
chr10	127119791	127119833	[redacted]	-0.447979	[redacted]
chr7	19196802	19196831	[redacted]	-0.447976	[redacted]
chr10	12596101	12596140	[redacted]	-0.447965	[redacted]
chr11	30506332	30506388	[redacted]	-0.447913	[redacted]
chr3	97213893	97213949	[redacted]	-0.447908	[redacted]
chr18	39027770	39027840	[redacted]	-0.44785	[redacted]
chr9	96089032	96089095	[redacted]	-0.447755	[redacted]
chr16	77099617	77099689	[redacted]	-0.447737	[redacted]
chr5	24803151	24803188	[redacted]	-0.447663	[redacted]
chr6	143590326	143590392	[redacted]	-0.447651	[redacted]
chr2	135985695	135985733	[redacted]	-0.447639	[redacted]
chr2	9859750	9859852	[redacted]	-0.44763	[redacted]
chr14	62254360	62254397	[redacted]	-0.447528	[redacted]
chr3	52272130	52272199	[redacted]	-0.447486	[redacted]
chr14	21159630	21159697	[redacted]	-0.447459	[redacted]
chr3	59162557	59162606	[redacted]	-0.447403	[redacted]
chr15	81667954	81668003	[redacted]	-0.447383	[redacted]
chr6	123030820	123030893	[redacted]	-0.447306	[redacted]
chr11	24161022	24161083	[redacted]	-0.447269	[redacted]
chr6	116183800	116183864	[redacted]	-0.447196	[redacted]
chr3	65876268	65876322	[redacted]	-0.447165	[redacted]
chr17	29488650	29488685	[redacted]	-0.447149	[redacted]
chr10	92559865	92559913	[redacted]	-0.447138	[redacted]
chr13	49093091	49093152	[redacted]	-0.447122	[redacted]
chr14	26908467	26908522	[redacted]	-0.447099	[redacted]
chr2	80355349	80355412	[redacted]	-0.446935	[redacted]
chr10	42027736	42027787	[redacted]	-0.446877	[redacted]
chr3	89862512	89862581	[redacted]	-0.446859	[redacted]
chr10	75327733	75327820	[redacted]	-0.446787	[redacted]

chr3	42128232	42128315	[redacted]	-0.446723	[redacted]
chr8	87193825	87193870	[redacted]	-0.446701	[redacted]
chr12	71067097	71067241	[redacted]	-0.44667	[redacted]
chr2	47685520	47685579	[redacted]	-0.446591	[redacted]
chr18	53887677	53887730	[redacted]	-0.446543	[redacted]
chr12	107646127	107646161	[redacted]	-0.446518	[redacted]
chr15	74818692	74818749	[redacted]	-0.446388	[redacted]
chr4	98118543	98118554	[redacted]	-0.44615	[redacted]
chr2	139970109	139970174	[redacted]	-0.446044	[redacted]
chr11	69718952	69718977	[redacted]	-0.445978	[redacted]
chr9	32719621	32719684	[redacted]	-0.445812	[redacted]
chr8	116891657	116891715	[redacted]	-0.445806	[redacted]
chr17	27087770	27087787	[redacted]	-0.445791	[redacted]
chr9	84318733	84318783	[redacted]	-0.445656	[redacted]
chr3	6480900	6480918	[redacted]	-0.445655	[redacted]
chr7	64388865	64388964	[redacted]	-0.445629	[redacted]
chr7	19910242	19910327	[redacted]	-0.445559	[redacted]
chr3	104947093	104947105	[redacted]	-0.445527	[redacted]
chr1	38577857	38577896	[redacted]	-0.445408	[redacted]
chr11	34091151	34091280	[redacted]	-0.44521	[redacted]
chr15	98426489	98426569	[redacted]	-0.445201	[redacted]
chr17	79789281	79789329	[redacted]	-0.445149	[redacted]
chr18	39823995	39824005	[redacted]	-0.445128	[redacted]
chr15	35916560	35916577	[redacted]	-0.445106	[redacted]
chr18	37931585	37931655	[redacted]	-0.445083	[redacted]
chr17	35925745	35925762	[redacted]	-0.445024	[redacted]
chr2	115276521	115276576	[redacted]	-0.444938	[redacted]
chr10	119564412	119564437	[redacted]	-0.444933	[redacted]
chr11	109293690	109293768	[redacted]	-0.444895	[redacted]
chr5	31772634	31772669	[redacted]	-0.444873	[redacted]
chr13	90959614	90959649	[redacted]	-0.444864	[redacted]
chr15	51846019	51846074	[redacted]	-0.444843	[redacted]
chr5	73154796	73154837	[redacted]	-0.444793	[redacted]
chrX	13551262	13551337	[redacted]	-0.444784	[redacted]
chr10	44380149	44380171	[redacted]	-0.444696	[redacted]
chr10	21348913	21348966	[redacted]	-0.444643	[redacted]
chr1	177315994	177316036	[redacted]	-0.44464	[redacted]
chr11	110469666	110469713	[redacted]	-0.444476	[redacted]
chr11	35539148	35539190	[redacted]	-0.444474	[redacted]
chr5	30848799	30848853	[redacted]	-0.444349	[redacted]
chr10	121036985	121036995	[redacted]	-0.444316	[redacted]
chr9	71718366	71718441	[redacted]	-0.444283	[redacted]
chr17	86885449	86885478	[redacted]	-0.444276	[redacted]

chr2	158057782	158057869	[redacted]	-0.444218	[redacted]
chr5	136475949	136476007	[redacted]	-0.444159	[redacted]
chr10	41881368	41881409	[redacted]	-0.444101	[redacted]
chr2	118190203	118190276	[redacted]	-0.444063	[redacted]
chr5	20611584	20611625	[redacted]	-0.444026	[redacted]
chr10	116971150	116971226	[redacted]	-0.443995	[redacted]
chr4	148634206	148634299	[redacted]	-0.443986	[redacted]
chr6	83842379	83842464	[redacted]	-0.443971	[redacted]
chr18	84499996	84500025	[redacted]	-0.443954	[redacted]
chr17	8034402	8034442	[redacted]	-0.44382	[redacted]
chr6	61107462	61107515	[redacted]	-0.443808	[redacted]
chr13	107143796	107143852	[redacted]	-0.443681	[redacted]
chr17	16006357	16006389	[redacted]	-0.443681	[redacted]
chr6	8484432	8484483	[redacted]	-0.443671	[redacted]
chr17	12342808	12342865	[redacted]	-0.443662	[redacted]
chr11	49991784	49991855	[redacted]	-0.443548	[redacted]
chr13	56743456	56743556	[redacted]	-0.443523	[redacted]
chr2	23830999	23831064	[redacted]	-0.443476	[redacted]
chr17	83510315	83510391	[redacted]	-0.443406	[redacted]
chr13	99473299	99473353	[redacted]	-0.443391	[redacted]
chr3	53634615	53634660	[redacted]	-0.443359	[redacted]
chr5	97775995	97776059	[redacted]	-0.44311	[redacted]
chr12	102588765	102588872	[redacted]	-0.443062	[redacted]
chr2	38834741	38834782	[redacted]	-0.443061	[redacted]
chr12	67084980	67085034	[redacted]	-0.443024	[redacted]
chr7	67588488	67588551	[redacted]	-0.442967	[redacted]
chr4	52260364	52260409	[redacted]	-0.442909	[redacted]
chr1	54990852	54990894	[redacted]	-0.442903	[redacted]
chr13	51982701	51982765	[redacted]	-0.442902	[redacted]
chr15	86727739	86727792	[redacted]	-0.442862	[redacted]
chr5	78374353	78374392	[redacted]	-0.44285	[redacted]
chr2	90849717	90849755	[redacted]	-0.442826	[redacted]
chr13	97217778	97217838	[redacted]	-0.442809	[redacted]
chr18	62863874	62863941	[redacted]	-0.442746	[redacted]
chr7	109422974	109423036	[redacted]	-0.442696	[redacted]
chr6	124450135	124450212	[redacted]	-0.44267	[redacted]
chr3	152133597	152133634	[redacted]	-0.442602	[redacted]
chr8	89009554	89009610	[redacted]	-0.442569	[redacted]
chrX	13288591	13288632	[redacted]	-0.442545	[redacted]
chr2	75720808	75720861	[redacted]	-0.442519	[redacted]
chr10	76121840	76121851	[redacted]	-0.442396	[redacted]
chr10	84935368	84935383	[redacted]	-0.442366	[redacted]
chr3	103214157	103214183	[redacted]	-0.442336	[redacted]

chr8	81983935	81984020	[redacted]	-0.442287	[redacted]
chr2	170993817	170993854	[redacted]	-0.442212	[redacted]
chr4	3500175	3500222	[redacted]	-0.442111	[redacted]
chr7	19761671	19761734	[redacted]	-0.44204	[redacted]
chr8	81260495	81260570	[redacted]	-0.442004	[redacted]
chr11	77732135	77732176	[redacted]	-0.441998	[redacted]
chr17	15743087	15743214	[redacted]	-0.441894	[redacted]
chr18	20632875	20632981	[redacted]	-0.441887	[redacted]
chr2	49816962	49817020	[redacted]	-0.441837	[redacted]
chr18	52431742	52431786	[redacted]	-0.441834	[redacted]
chr3	135559074	135559093	[redacted]	-0.441828	[redacted]
chr12	91211947	91211992	[redacted]	-0.441747	[redacted]
chr15	96690012	96690087	[redacted]	-0.441713	[redacted]
chr1	64859886	64859941	[redacted]	-0.441709	[redacted]
chr15	100238192	100238256	[redacted]	-0.441709	[redacted]
chr9	72106754	72106829	[redacted]	-0.441693	[redacted]
chr2	132265517	132265560	[redacted]	-0.441678	[redacted]
chr10	12809019	12809091	[redacted]	-0.441649	[redacted]
chr4	93125252	93125320	[redacted]	-0.441616	[redacted]
chr19	29469305	29469322	[redacted]	-0.441596	[redacted]
chr10	63166218	63166265	[redacted]	-0.441563	[redacted]
chr2	24747649	24747658	[redacted]	-0.441556	[redacted]
chr7	113783458	113783525	[redacted]	-0.441533	[redacted]
chr6	50347216	50347265	[redacted]	-0.441514	[redacted]
chr2	9868708	9868790	[redacted]	-0.441503	[redacted]
chr17	27789764	27789812	[redacted]	-0.441494	[redacted]
chr18	77990595	77990632	[redacted]	-0.441387	[redacted]
chr2	13291243	13291300	[redacted]	-0.441368	[redacted]
chr5	57786385	57786444	[redacted]	-0.441259	[redacted]
chr2	114119877	114119931	[redacted]	-0.441232	[redacted]
chr14	63135292	63135305	[redacted]	-0.441181	[redacted]
chr2	165873515	165873580	[redacted]	-0.441145	[redacted]
chr19	42173462	42173498	[redacted]	-0.441086	[redacted]
chrX	14608644	14608686	[redacted]	-0.441078	[redacted]
chr1	20540896	20540939	[redacted]	-0.441022	[redacted]
chr9	115183049	115183111	[redacted]	-0.440997	[redacted]
chr11	70143397	70143472	[redacted]	-0.440912	[redacted]
chr6	49549901	49549974	[redacted]	-0.440857	[redacted]
chr17	64053309	64053366	[redacted]	-0.440799	[redacted]
chr11	84125681	84125711	[redacted]	-0.440682	[redacted]
chr6	125263916	125264006	[redacted]	-0.440677	[redacted]
chr8	22680735	22680779	[redacted]	-0.44066	[redacted]
chr12	81318293	81318365	[redacted]	-0.440612	[redacted]

chr11	51224252	51224296	[redacted]	-0.440535	[redacted]
chr10	12822940	12823017	[redacted]	-0.440516	[redacted]
chr11	118963802	118963848	[redacted]	-0.440509	[redacted]
chr19	21641526	21641578	[redacted]	-0.4405	[redacted]
chr11	80392410	80392471	[redacted]	-0.440467	[redacted]
chr1	20788621	20788686	[redacted]	-0.44046	[redacted]
chr7	127085965	127086040	[redacted]	-0.440358	[redacted]
chr4	116406003	116406084	[redacted]	-0.440355	[redacted]
chr2	76714743	76714800	[redacted]	-0.44033	[redacted]
chr14	122747582	122747631	[redacted]	-0.440308	[redacted]
chr18	23839960	23840010	[redacted]	-0.440307	[redacted]
chr10	67498296	67498343	[redacted]	-0.440304	[redacted]
chr10	28673549	28673581	[redacted]	-0.440262	[redacted]
chr6	98854050	98854076	[redacted]	-0.440241	[redacted]
chr1	34114101	34114202	[redacted]	-0.440174	[redacted]
chr3	152947210	152947343	[redacted]	-0.440152	[redacted]
chr17	88032876	88032920	[redacted]	-0.44008	[redacted]
chr12	84780336	84780420	[redacted]	-0.440001	[redacted]
chr1	63496069	63496114	[redacted]	-0.439974	[redacted]
chr8	121751806	121751892	[redacted]	-0.439862	[redacted]
chr19	56687999	56688073	[redacted]	-0.439799	[redacted]
chr11	118260878	118260905	[redacted]	-0.439788	[redacted]
chr2	18394827	18394918	[redacted]	-0.439675	[redacted]
chr1	60978196	60978213	[redacted]	-0.439644	[redacted]
chr17	86989424	86989492	[redacted]	-0.439632	[redacted]
chr4	34912161	34912211	[redacted]	-0.439616	[redacted]
chr2	8331165	8331247	[redacted]	-0.439613	[redacted]
chr15	96695442	96695498	[redacted]	-0.439538	[redacted]
chr16	17341805	17341891	[redacted]	-0.439338	[redacted]
chr2	70969780	70969826	[redacted]	-0.439283	[redacted]
chr7	101539337	101539354	[redacted]	-0.439208	[redacted]
chr1	8639642	8639684	[redacted]	-0.439106	[redacted]
chr13	56121717	56121784	[redacted]	-0.439098	[redacted]
chr6	147141963	147142042	[redacted]	-0.439033	[redacted]
chr2	96240537	96240568	[redacted]	-0.438969	[redacted]
chr7	116260304	116260356	[redacted]	-0.438867	[redacted]
chr2	59905597	59905679	[redacted]	-0.438824	[redacted]
chr17	3356010	3356028	[redacted]	-0.438736	[redacted]
chr15	49247707	49247774	[redacted]	-0.438705	[redacted]
chr13	102353820	102353887	[redacted]	-0.438704	[redacted]
chr6	84405213	84405247	[redacted]	-0.438615	[redacted]
chr1	46722332	46722383	[redacted]	-0.438571	[redacted]
chr9	71571937	71572018	[redacted]	-0.438472	[redacted]

chr11	53201618	53201704	[redacted]	-0.438461	[redacted]
chr11	116210936	116210957	[redacted]	-0.438438	[redacted]
chr10	25366002	25366076	[redacted]	-0.438333	[redacted]
chr17	35249105	35249130	[redacted]	-0.438328	[redacted]
chr1	79307212	79307271	[redacted]	-0.438313	[redacted]
chr5	122391293	122391396	[redacted]	-0.438248	[redacted]
chrX	7442770	7442815	[redacted]	-0.438234	[redacted]
chr7	46526387	46526434	[redacted]	-0.438227	[redacted]
chr15	50674212	50674279	[redacted]	-0.438196	[redacted]
chr1	43216225	43216301	[redacted]	-0.438191	[redacted]
chr10	117852788	117852841	[redacted]	-0.438151	[redacted]
chr11	80689132	80689198	[redacted]	-0.438147	[redacted]
chr9	49624513	49624565	[redacted]	-0.43805	[redacted]
chr8	34854876	34854903	[redacted]	-0.438045	[redacted]
chr11	62090553	62090653	[redacted]	-0.437961	[redacted]
chr8	120514636	120514695	[redacted]	-0.437895	[redacted]
chr6	140642322	140642359	[redacted]	-0.43789	[redacted]
chr17	67938877	67938933	[redacted]	-0.437856	[redacted]
chr16	30511866	30511919	[redacted]	-0.437822	[redacted]
chr9	84857008	84857052	[redacted]	-0.437775	[redacted]
chr4	136010608	136010664	[redacted]	-0.437665	[redacted]
chr19	4660838	4660902	[redacted]	-0.437569	[redacted]
chr8	37088868	37088922	[redacted]	-0.437551	[redacted]
chr4	101215761	101215814	[redacted]	-0.437528	[redacted]
chr2	103991989	103992020	[redacted]	-0.437526	[redacted]
chr3	60561617	60561702	[redacted]	-0.43748	[redacted]
chr8	104449010	104449058	[redacted]	-0.437436	[redacted]
chr1	41712785	41712836	[redacted]	-0.437426	[redacted]
chr9	84039041	84039072	[redacted]	-0.437414	[redacted]
chr19	38596656	38596689	[redacted]	-0.437383	[redacted]
chr16	10145370	10145461	[redacted]	-0.437317	[redacted]
chr1	175627893	175627982	[redacted]	-0.437295	[redacted]
chr15	11989699	11989711	[redacted]	-0.437229	[redacted]
chr16	54004851	54004910	[redacted]	-0.437157	[redacted]
chr13	45932412	45932453	[redacted]	-0.436993	[redacted]
chr7	122999906	122999953	[redacted]	-0.436961	[redacted]
chrX	49204785	49204843	[redacted]	-0.436951	[redacted]
chr3	40525259	40525299	[redacted]	-0.436873	[redacted]
chr17	29039755	29039865	[redacted]	-0.436868	[redacted]
chr8	35596469	35596539	[redacted]	-0.436728	[redacted]
chr19	30168602	30168675	[redacted]	-0.436615	[redacted]
chr6	146996942	146996993	[redacted]	-0.436424	[redacted]
chr14	45048064	45048138	[redacted]	-0.436403	[redacted]

chrX	51404790	51404843	[redacted]	-0.436363	[redacted]
chr19	56757603	56757686	[redacted]	-0.436359	[redacted]
chr11	89836122	89836164	[redacted]	-0.436308	[redacted]
chr5	28400151	28400191	[redacted]	-0.436306	[redacted]
chr4	31920937	31921018	[redacted]	-0.43627	[redacted]
chr1	144022994	144023072	[redacted]	-0.436262	[redacted]
chr3	29214613	29214655	[redacted]	-0.436255	[redacted]
chr4	140711070	140711135	[redacted]	-0.4362	[redacted]
chr1	101483754	101483850	[redacted]	-0.436156	[redacted]
chr9	30426322	30426404	[redacted]	-0.436151	[redacted]
chr10	21136222	21136283	[redacted]	-0.436078	[redacted]
chr15	100016497	100016575	[redacted]	-0.436044	[redacted]
chr10	111758069	111758104	[redacted]	-0.436032	[redacted]
chr12	11112484	11112581	[redacted]	-0.436016	[redacted]
chr17	29853731	29853820	[redacted]	-0.43595	[redacted]
chr12	58270598	58270682	[redacted]	-0.435933	[redacted]
chr7	66731459	66731470	[redacted]	-0.435887	[redacted]
chr18	15636620	15636669	[redacted]	-0.435854	[redacted]
chr6	40476752	40476770	[redacted]	-0.435798	[redacted]
chr13	108860734	108860797	[redacted]	-0.435795	[redacted]
chr19	32774956	32775005	[redacted]	-0.435687	[redacted]
chr2	156104574	156104609	[redacted]	-0.435682	[redacted]
chr19	33228937	33229086	[redacted]	-0.435646	[redacted]
chr11	44007656	44007716	[redacted]	-0.435571	[redacted]
chr13	44787204	44787244	[redacted]	-0.435546	[redacted]
chr15	59207441	59207516	[redacted]	-0.435487	[redacted]
chr7	138917940	138918010	[redacted]	-0.435389	[redacted]
chr9	65578523	65578552	[redacted]	-0.435182	[redacted]
chr1	160543539	160543645	[redacted]	-0.435166	[redacted]
chr16	96413797	96413879	[redacted]	-0.435143	[redacted]
chr9	103353311	103353405	[redacted]	-0.43512	[redacted]
chr15	93959061	93959114	[redacted]	-0.435096	[redacted]
chr13	94567775	94567796	[redacted]	-0.435075	[redacted]
chr14	8640363	8640444	[redacted]	-0.43507	[redacted]
chr1	10651914	10651951	[redacted]	-0.435068	[redacted]
chr12	64894567	64894635	[redacted]	-0.435052	[redacted]
chr10	92203413	92203448	[redacted]	-0.43505	[redacted]
chr9	92536818	92536874	[redacted]	-0.434968	[redacted]
chr13	28754361	28754378	[redacted]	-0.434736	[redacted]
chr12	98478343	98478359	[redacted]	-0.434706	[redacted]
chr2	117423133	117423186	[redacted]	-0.434653	[redacted]
chr18	87240151	87240213	[redacted]	-0.434549	[redacted]
chr9	43376199	43376280	[redacted]	-0.434304	[redacted]

chr10	69535767	69535871	[redacted]	-0.434276	[redacted]
chr19	53408423	53408511	[redacted]	-0.434275	[redacted]
chr4	137492380	137492436	[redacted]	-0.434262	[redacted]
chr11	113476965	113477035	[redacted]	-0.434222	[redacted]
chr7	81114412	81114484	[redacted]	-0.434221	[redacted]
chr17	82556929	82557010	[redacted]	-0.434148	[redacted]
chr3	86958335	86958359	[redacted]	-0.43406	[redacted]
chr17	31594519	31594592	[redacted]	-0.434052	[redacted]
chr8	107579819	107579880	[redacted]	-0.434041	[redacted]
chr5	34167538	34167577	[redacted]	-0.433954	[redacted]
chr14	111642020	111642105	[redacted]	-0.433908	[redacted]
chr9	120416349	120416381	[redacted]	-0.433899	[redacted]
chr15	11934558	11934641	[redacted]	-0.433819	[redacted]
chr15	36642825	36642882	[redacted]	-0.433779	[redacted]
chr6	148201520	148201578	[redacted]	-0.433772	[redacted]
chr12	10230737	10230773	[redacted]	-0.433731	[redacted]
chr1	170750763	170750811	[redacted]	-0.43373	[redacted]
chr7	16794082	16794148	[redacted]	-0.433711	[redacted]
chr9	32331250	32331332	[redacted]	-0.433694	[redacted]
chr10	108417974	108418008	[redacted]	-0.433683	[redacted]
chr5	65817641	65817669	[redacted]	-0.433634	[redacted]
chr14	47411371	47411450	[redacted]	-0.433629	[redacted]
chr16	9046707	9046785	[redacted]	-0.433612	[redacted]
chr2	18634132	18634200	[redacted]	-0.433606	[redacted]
chr14	78040814	78040852	[redacted]	-0.433552	[redacted]
chr11	20205285	20205303	[redacted]	-0.433496	[redacted]
chr2	169811474	169811557	[redacted]	-0.433463	[redacted]
chr6	140787945	140787987	[redacted]	-0.433329	[redacted]
chr16	9297923	9298016	[redacted]	-0.433328	[redacted]
chr13	102338946	102338990	[redacted]	-0.433292	[redacted]
chr2	34017262	34017320	[redacted]	-0.433273	[redacted]
chr15	58275266	58275311	[redacted]	-0.433223	[redacted]
chr4	5684068	5684106	[redacted]	-0.433189	[redacted]
chr19	43347683	43347746	[redacted]	-0.43318	[redacted]
chr8	126660371	126660438	[redacted]	-0.433145	[redacted]
chr14	27405059	27405149	[redacted]	-0.433131	[redacted]
chr2	62381830	62381906	[redacted]	-0.433088	[redacted]
chr16	21841139	21841205	[redacted]	-0.433055	[redacted]
chr17	8818031	8818133	[redacted]	-0.433019	[redacted]
chr10	116486904	116486914	[redacted]	-0.433003	[redacted]
chr3	127869472	127869557	[redacted]	-0.432982	[redacted]
chr15	63793293	63793354	[redacted]	-0.432968	[redacted]
chr10	3953734	3953789	[redacted]	-0.432963	[redacted]

chr2	157509747	157509791	[redacted]	-0.432952	[redacted]
chr12	91322106	91322173	[redacted]	-0.432905	[redacted]
chr2	153370262	153370347	[redacted]	-0.432895	[redacted]
chr17	50796834	50796856	[redacted]	-0.432863	[redacted]
chr3	31012132	31012158	[redacted]	-0.432854	[redacted]
chr9	115251582	115251655	[redacted]	-0.432824	[redacted]
chr4	106391003	106391084	[redacted]	-0.432789	[redacted]
chr15	51064733	51064787	[redacted]	-0.432778	[redacted]
chr10	111518352	111518388	[redacted]	-0.432764	[redacted]
chr10	87505518	87505586	[redacted]	-0.432738	[redacted]
chr12	83095956	83096064	[redacted]	-0.432736	[redacted]
chr10	12668247	12668330	[redacted]	-0.432714	[redacted]
chr9	120322713	120322737	[redacted]	-0.432643	[redacted]
chr10	8854097	8854133	[redacted]	-0.432582	[redacted]
chr1	79645202	79645285	[redacted]	-0.4325	[redacted]
chr5	99856033	99856123	[redacted]	-0.432465	[redacted]
chr9	61159518	61159703	[redacted]	-0.432351	[redacted]
chr15	74014961	74015047	[redacted]	-0.432334	[redacted]
chr9	116425547	116425616	[redacted]	-0.432293	[redacted]
chr1	43491087	43491114	[redacted]	-0.432284	[redacted]
chr12	64645641	64645693	[redacted]	-0.432229	[redacted]
chrX	142777211	142777258	[redacted]	-0.432198	[redacted]
chr2	76835929	76835979	[redacted]	-0.432192	[redacted]
chr7	120371564	120371639	[redacted]	-0.432185	[redacted]
chr17	50094035	50094046	[redacted]	-0.432112	[redacted]
chr7	16825884	16825902	[redacted]	-0.432085	[redacted]
chr5	124482322	124482375	[redacted]	-0.432025	[redacted]
chr16	15991725	15991822	[redacted]	-0.432001	[redacted]
chr6	27522335	27522413	[redacted]	-0.431892	[redacted]
chr5	147533573	147533617	[redacted]	-0.431874	[redacted]
chr15	77628613	77628649	[redacted]	-0.431797	[redacted]
chr13	13691314	13691391	[redacted]	-0.431687	[redacted]
chr3	104873784	104873887	[redacted]	-0.431677	[redacted]
chr14	30037994	30038060	[redacted]	-0.431583	[redacted]
chr4	82263389	82263453	[redacted]	-0.431477	[redacted]
chr1	134324125	134324150	[redacted]	-0.431447	[redacted]
chr12	73928077	73928105	[redacted]	-0.431433	[redacted]
chr6	80978651	80978697	[redacted]	-0.431414	[redacted]
chr14	86484622	86484661	[redacted]	-0.431349	[redacted]
chr9	96893139	96893192	[redacted]	-0.43132	[redacted]
chr4	136342478	136342545	[redacted]	-0.43128	[redacted]
chr7	91814780	91814819	[redacted]	-0.431188	[redacted]
chr16	44510682	44510730	[redacted]	-0.431184	[redacted]

chr14	47594810	47594867	[redacted]	-0.431142	[redacted]
chr8	75420926	75420991	[redacted]	-0.431109	[redacted]
chr11	68440496	68440530	[redacted]	-0.431108	[redacted]
chr9	122240046	122240094	[redacted]	-0.431047	[redacted]
chr6	37740993	37741021	[redacted]	-0.431021	[redacted]
chr12	92578424	92578487	[redacted]	-0.43098	[redacted]
chr5	124339904	124339962	[redacted]	-0.430967	[redacted]
chr11	96643808	96643854	[redacted]	-0.430962	[redacted]
chr7	90284814	90284881	[redacted]	-0.430947	[redacted]
chr6	122391383	122391453	[redacted]	-0.430929	[redacted]
chr15	11668900	11668953	[redacted]	-0.430905	[redacted]
chr16	3728346	3728416	[redacted]	-0.430882	[redacted]
chr16	46020971	46021042	[redacted]	-0.430855	[redacted]
chr7	126453838	126453917	[redacted]	-0.43084	[redacted]
chr17	66506445	66506498	[redacted]	-0.430627	[redacted]
chr2	160432184	160432229	[redacted]	-0.430616	[redacted]
chr9	66628174	66628184	[redacted]	-0.430577	[redacted]
chr7	59039563	59039653	[redacted]	-0.430562	[redacted]
chr1	177825925	177825947	[redacted]	-0.430554	[redacted]
chr6	83452659	83452714	[redacted]	-0.430541	[redacted]
chr2	48518278	48518318	[redacted]	-0.430525	[redacted]
chr9	30153032	30153095	[redacted]	-0.430446	[redacted]
chr3	119779892	119779919	[redacted]	-0.43038	[redacted]
chr10	59703634	59703661	[redacted]	-0.430346	[redacted]
chr14	58231277	58231320	[redacted]	-0.430247	[redacted]
chr17	87976558	87976597	[redacted]	-0.430225	[redacted]
chr6	99172771	99172868	[redacted]	-0.430215	[redacted]
chr14	101747498	101747538	[redacted]	-0.43008	[redacted]
chr19	43790669	43790720	[redacted]	-0.430069	[redacted]
chr6	32973485	32973548	[redacted]	-0.430033	[redacted]
chr6	142866375	142866426	[redacted]	-0.43	[redacted]
chr4	130842177	130842225	[redacted]	-0.429995	[redacted]
chr13	77424167	77424249	[redacted]	-0.429981	[redacted]
chr3	139018569	139018628	[redacted]	-0.429928	[redacted]
chr12	117535364	117535420	[redacted]	-0.429802	[redacted]
chr17	8007496	8007587	[redacted]	-0.429798	[redacted]
chr3	42732475	42732505	[redacted]	-0.429783	[redacted]
chr11	110311944	110312027	[redacted]	-0.429772	[redacted]
chr11	30470933	30470985	[redacted]	-0.429745	[redacted]
chr3	36331699	36331778	[redacted]	-0.429665	[redacted]
chr10	12550681	12550749	[redacted]	-0.429633	[redacted]
chr18	82155710	82155762	[redacted]	-0.42959	[redacted]
chr8	105642626	105642650	[redacted]	-0.429564	[redacted]

chr8	71103504	71103581	[redacted]	-0.429504	[redacted]
chr14	98332140	98332226	[redacted]	-0.429489	[redacted]
chrX	138849583	138849653	[redacted]	-0.429405	[redacted]
chr3	96975160	96975244	[redacted]	-0.429344	[redacted]
chr6	133393000	133393050	[redacted]	-0.429309	[redacted]
chr4	44505231	44505288	[redacted]	-0.429295	[redacted]
chr7	67453895	67453964	[redacted]	-0.42928	[redacted]
chr11	79827417	79827467	[redacted]	-0.429019	[redacted]
chr11	117294494	117294553	[redacted]	-0.428988	[redacted]
chr16	95752310	95752376	[redacted]	-0.428942	[redacted]
chr19	29046299	29046378	[redacted]	-0.428861	[redacted]
chr9	55174880	55174923	[redacted]	-0.428738	[redacted]
chr15	102479027	102479063	[redacted]	-0.428611	[redacted]
chr14	32606643	32606718	[redacted]	-0.428607	[redacted]
chr6	146860864	146860935	[redacted]	-0.428537	[redacted]
chr1	127978589	127978657	[redacted]	-0.428501	[redacted]
chr3	129537789	129537811	[redacted]	-0.428412	[redacted]
chr7	31133535	31133601	[redacted]	-0.42837	[redacted]
chr6	141433620	141433676	[redacted]	-0.428307	[redacted]
chr14	29814338	29814400	[redacted]	-0.428297	[redacted]
chr1	97977742	97977774	[redacted]	-0.428282	[redacted]
chr6	53264596	53264635	[redacted]	-0.428261	[redacted]
chr9	101288154	101288175	[redacted]	-0.428201	[redacted]
chr9	53843017	53843063	[redacted]	-0.42807	[redacted]
chr10	117958923	117958947	[redacted]	-0.427994	[redacted]
chr2	28729220	28729287	[redacted]	-0.427971	[redacted]
chr12	102447028	102447069	[redacted]	-0.427913	[redacted]
chr9	118901699	118901776	[redacted]	-0.427887	[redacted]
chr16	50473957	50474004	[redacted]	-0.427849	[redacted]
chr8	120916247	120916280	[redacted]	-0.427839	[redacted]
chr11	78890390	78890481	[redacted]	-0.427835	[redacted]
chr19	55750222	55750278	[redacted]	-0.427834	[redacted]
chr18	37896107	37896169	[redacted]	-0.427832	[redacted]
chr1	37940013	37940079	[redacted]	-0.427812	[redacted]
chr19	8894798	8894887	[redacted]	-0.427804	[redacted]
chr13	109954333	109954384	[redacted]	-0.427531	[redacted]
chr1	139480898	139480920	[redacted]	-0.427513	[redacted]
chr18	38760895	38760942	[redacted]	-0.427503	[redacted]
chr18	74844078	74844158	[redacted]	-0.427502	[redacted]
chr15	102343273	102343353	[redacted]	-0.427436	[redacted]
chr2	103489336	103489387	[redacted]	-0.42741	[redacted]
chr3	105652453	105652490	[redacted]	-0.427282	[redacted]
chr15	101007629	101007706	[redacted]	-0.427215	[redacted]

chr6	72453324	72453378	[redacted]	-0.427213	[redacted]
chr3	129936581	129936669	[redacted]	-0.42718	[redacted]
chr6	31113198	31113232	[redacted]	-0.427141	[redacted]
chr3	86832912	86832962	[redacted]	-0.427066	[redacted]
chr2	86005226	86005310	[redacted]	-0.427033	[redacted]
chr12	51879324	51879416	[redacted]	-0.426988	[redacted]
chr10	90091536	90091612	[redacted]	-0.426962	[redacted]
chr4	118302416	118302531	[redacted]	-0.426836	[redacted]
chr4	156013732	156013782	[redacted]	-0.426815	[redacted]
chr14	26766160	26766213	[redacted]	-0.426805	[redacted]
chr16	26512648	26512714	[redacted]	-0.426784	[redacted]
chr2	172366226	172366281	[redacted]	-0.426757	[redacted]
chr17	86434274	86434301	[redacted]	-0.426742	[redacted]
chr2	159988385	159988443	[redacted]	-0.426728	[redacted]
chr14	93172001	93172064	[redacted]	-0.426704	[redacted]
chr17	78295106	78295143	[redacted]	-0.426658	[redacted]
chr18	66404280	66404319	[redacted]	-0.426656	[redacted]
chr7	45778571	45778621	[redacted]	-0.426636	[redacted]
chr10	44401812	44401978	[redacted]	-0.426574	[redacted]
chr14	73379831	73379837	[redacted]	-0.426564	[redacted]
chr14	101751784	101751866	[redacted]	-0.426449	[redacted]
chr13	83512848	83512927	[redacted]	-0.426381	[redacted]
chr16	23346655	23346672	[redacted]	-0.426146	[redacted]
chr15	80956019	80956068	[redacted]	-0.426085	[redacted]
chr12	51867016	51867085	[redacted]	-0.426056	[redacted]
chr12	53835130	53835142	[redacted]	-0.426031	[redacted]
chr6	122639679	122639767	[redacted]	-0.426009	[redacted]
chr3	116465406	116465568	[redacted]	-0.425985	[redacted]
chr7	5068465	5068642	[redacted]	-0.425897	[redacted]
chr11	82371629	82371642	[redacted]	-0.425889	[redacted]
chr3	84389150	84389195	[redacted]	-0.425789	[redacted]
chr3	100641639	100641671	[redacted]	-0.425735	[redacted]
chr17	71206012	71206087	[redacted]	-0.425632	[redacted]
chr6	30949183	30949211	[redacted]	-0.42563	[redacted]
chr2	144730774	144730855	[redacted]	-0.425589	[redacted]
chr11	102011624	102011659	[redacted]	-0.42542	[redacted]
chr11	100052640	100052725	[redacted]	-0.425355	[redacted]
chr13	44092122	44092151	[redacted]	-0.425309	[redacted]
chr3	93598199	93598247	[redacted]	-0.42527	[redacted]
chr15	42799134	42799176	[redacted]	-0.425213	[redacted]
chr2	31100900	31101003	[redacted]	-0.425177	[redacted]
chr8	8127899	8127954	[redacted]	-0.425141	[redacted]
chr11	115477569	115477610	[redacted]	-0.425129	[redacted]

chr4	48013111	48013171	[redacted]	-0.425097	[redacted]
chr15	57884466	57884536	[redacted]	-0.425085	[redacted]
chr7	133624905	133625022	[redacted]	-0.425056	[redacted]
chr6	34034636	34034677	[redacted]	-0.425016	[redacted]
chr1	61245675	61245716	[redacted]	-0.42498	[redacted]
chr1	138536479	138536514	[redacted]	-0.42496	[redacted]
chr12	76841507	76841529	[redacted]	-0.424956	[redacted]
chr9	112540911	112540958	[redacted]	-0.424915	[redacted]
chr19	58120291	58120328	[redacted]	-0.424914	[redacted]
chr12	102970580	102970667	[redacted]	-0.424893	[redacted]
chr19	27196089	27196124	[redacted]	-0.424804	[redacted]
chr8	4289276	4289313	[redacted]	-0.424795	[redacted]
chr17	31260973	31261035	[redacted]	-0.424744	[redacted]
chr2	163075951	163075965	[redacted]	-0.42472	[redacted]
chr8	33407518	33407565	[redacted]	-0.424703	[redacted]
chr3	131176998	131177057	[redacted]	-0.424656	[redacted]
chr4	115749124	115749214	[redacted]	-0.424623	[redacted]
chr12	72777485	72777517	[redacted]	-0.424532	[redacted]
chr15	52743203	52743245	[redacted]	-0.424498	[redacted]
chr1	13587569	13587631	[redacted]	-0.424439	[redacted]
chr17	29116330	29116359	[redacted]	-0.424438	[redacted]
chr16	72821528	72821588	[redacted]	-0.424389	[redacted]
chr15	5806024	5806088	[redacted]	-0.424374	[redacted]
chr5	134093231	134093275	[redacted]	-0.424353	[redacted]
chr18	67662992	67663037	[redacted]	-0.424335	[redacted]
chr8	57578956	57578989	[redacted]	-0.424332	[redacted]
chr6	35936069	35936149	[redacted]	-0.424278	[redacted]
chr10	25876020	25876091	[redacted]	-0.424258	[redacted]
chr14	118728187	118728247	[redacted]	-0.424202	[redacted]
chr9	31967740	31967807	[redacted]	-0.424164	[redacted]
chr9	109736981	109737053	[redacted]	-0.424147	[redacted]
chr6	125152383	125152459	[redacted]	-0.424144	[redacted]
chr7	45092942	45093007	[redacted]	-0.424143	[redacted]
chr13	52909416	52909457	[redacted]	-0.424049	[redacted]
chr8	119861538	119861600	[redacted]	-0.42401	[redacted]
chr16	86541883	86541888	[redacted]	-0.423951	[redacted]
chr10	68393009	68393070	[redacted]	-0.423924	[redacted]
chr11	106379893	106379986	[redacted]	-0.42387	[redacted]
chr19	17411681	17411763	[redacted]	-0.423821	[redacted]
chr17	83566547	83566579	[redacted]	-0.423812	[redacted]
chr11	105525021	105525067	[redacted]	-0.423744	[redacted]
chr2	84379465	84379528	[redacted]	-0.423719	[redacted]
chr15	81849775	81849798	[redacted]	-0.423704	[redacted]

chr3	32206375	32206426	[redacted]	-0.423654	[redacted]
chr15	59532776	59532839	[redacted]	-0.423614	[redacted]
chr4	108324242	108324350	[redacted]	-0.423584	[redacted]
chr5	101013419	101013467	[redacted]	-0.423573	[redacted]
chr13	54501794	54501810	[redacted]	-0.423498	[redacted]
chr1	71528874	71528927	[redacted]	-0.423456	[redacted]
chr13	40203423	40203473	[redacted]	-0.423348	[redacted]
chr4	48988317	48988351	[redacted]	-0.423288	[redacted]
chr4	119603054	119603096	[redacted]	-0.42327	[redacted]
chr13	43607066	43607143	[redacted]	-0.423264	[redacted]
chr6	100294449	100294516	[redacted]	-0.423258	[redacted]
chrX	136383451	136383485	[redacted]	-0.423225	[redacted]
chr5	116130296	116130326	[redacted]	-0.423199	[redacted]
chr14	16145138	16145223	[redacted]	-0.423165	[redacted]
chr17	30686107	30686209	[redacted]	-0.423156	[redacted]
chr16	32979790	32979837	[redacted]	-0.423112	[redacted]
chr3	29633619	29633696	[redacted]	-0.423077	[redacted]
chr11	71057888	71057914	[redacted]	-0.423054	[redacted]
chr14	63835220	63835251	[redacted]	-0.423044	[redacted]
chr1	33779499	33779566	[redacted]	-0.423038	[redacted]
chr6	121053435	121053490	[redacted]	-0.422978	[redacted]
chr7	31281439	31281498	[redacted]	-0.422923	[redacted]
chr2	161967048	161967109	[redacted]	-0.42282	[redacted]
chr2	78060549	78060595	[redacted]	-0.422794	[redacted]
chr3	17584257	17584306	[redacted]	-0.422758	[redacted]
chr4	102949150	102949196	[redacted]	-0.422735	[redacted]
chr9	110737399	110737491	[redacted]	-0.4227	[redacted]
chr18	82502099	82502141	[redacted]	-0.422697	[redacted]
chr11	38544609	38544671	[redacted]	-0.422669	[redacted]
chr14	16195977	16196040	[redacted]	-0.422632	[redacted]
chr15	53632880	53632925	[redacted]	-0.422618	[redacted]
chr2	156210822	156210893	[redacted]	-0.422498	[redacted]
chr13	8857291	8857359	[redacted]	-0.422472	[redacted]
chr2	160552148	160552250	[redacted]	-0.422467	[redacted]
chr4	118065599	118065614	[redacted]	-0.42246	[redacted]
chr5	86076814	86076858	[redacted]	-0.422403	[redacted]
chr10	95258233	95258247	[redacted]	-0.422395	[redacted]
chr8	46583790	46583854	[redacted]	-0.422386	[redacted]
chr13	54944887	54944937	[redacted]	-0.422151	[redacted]
chr11	62862425	62862464	[redacted]	-0.422068	[redacted]
chr19	22119925	22119978	[redacted]	-0.422053	[redacted]
chr1	180728614	180728699	[redacted]	-0.422014	[redacted]
chr13	41135853	41135927	[redacted]	-0.422014	[redacted]

chr15	86168996	86169049	[redacted]	-0.421965	[redacted]
chr5	88766723	88766775	[redacted]	-0.42196	[redacted]
chr9	97089531	97089572	[redacted]	-0.421935	[redacted]
chr13	21213334	21213381	[redacted]	-0.421816	[redacted]
chr4	140780465	140780498	[redacted]	-0.421804	[redacted]
chr4	77038023	77038101	[redacted]	-0.421738	[redacted]
chr6	101094599	101094666	[redacted]	-0.421625	[redacted]
chr9	101335363	101335430	[redacted]	-0.421613	[redacted]
chr2	128731760	128731809	[redacted]	-0.421583	[redacted]
chr5	142790676	142790695	[redacted]	-0.421563	[redacted]
chr7	140287053	140287091	[redacted]	-0.421525	[redacted]
chr8	103253621	103253698	[redacted]	-0.421503	[redacted]
chr5	21597304	21597393	[redacted]	-0.421484	[redacted]
chr11	76825044	76825082	[redacted]	-0.421438	[redacted]
chr9	90107812	90107847	[redacted]	-0.421392	[redacted]
chr15	38609015	38609073	[redacted]	-0.421375	[redacted]
chr6	38577576	38577629	[redacted]	-0.421341	[redacted]
chr2	155363427	155363486	[redacted]	-0.421331	[redacted]
chr3	34525369	34525407	[redacted]	-0.421316	[redacted]
chr3	108055959	108055994	[redacted]	-0.421277	[redacted]
chr7	120140301	120140342	[redacted]	-0.421231	[redacted]
chr1	182733941	182733979	[redacted]	-0.421207	[redacted]
chr6	50850950	50851008	[redacted]	-0.4212	[redacted]
chr14	77842815	77842853	[redacted]	-0.421182	[redacted]
chr9	32868878	32868883	[redacted]	-0.420976	[redacted]
chr6	50301607	50301662	[redacted]	-0.420959	[redacted]
chr14	30017606	30017673	[redacted]	-0.420844	[redacted]
chr17	47486475	47486538	[redacted]	-0.420841	[redacted]
chr15	93483071	93483167	[redacted]	-0.420788	[redacted]
chr5	97934700	97934722	[redacted]	-0.420718	[redacted]
chr3	101523269	101523293	[redacted]	-0.420708	[redacted]
chr18	84713884	84713931	[redacted]	-0.420643	[redacted]
chr13	45158202	45158246	[redacted]	-0.420589	[redacted]
chr4	11975111	11975192	[redacted]	-0.420496	[redacted]
chr9	46144975	46145037	[redacted]	-0.420415	[redacted]
chr10	36806303	36806366	[redacted]	-0.420374	[redacted]
chr2	28765983	28765996	[redacted]	-0.420369	[redacted]
chr4	57833372	57833437	[redacted]	-0.420359	[redacted]
chr5	122279833	122279865	[redacted]	-0.420323	[redacted]
chr9	123538336	123538377	[redacted]	-0.420262	[redacted]
chrX	74764964	74765026	[redacted]	-0.420228	[redacted]
chr12	69221109	69221179	[redacted]	-0.420196	[redacted]
chr4	70816210	70816268	[redacted]	-0.420193	[redacted]

chr6	99138045	99138119	[redacted]	-0.420191	[redacted]
chr10	130328727	130328794	[redacted]	-0.420182	[redacted]
chr16	46458886	46458937	[redacted]	-0.420048	[redacted]
chr14	86331831	86331902	[redacted]	-0.420036	[redacted]
chr10	44523491	44523522	[redacted]	-0.419997	[redacted]
chr9	35564953	35565004	[redacted]	-0.419915	[redacted]
chr13	92315336	92315385	[redacted]	-0.419909	[redacted]
chr5	112203867	112203922	[redacted]	-0.419901	[redacted]
chr8	79708428	79708487	[redacted]	-0.419867	[redacted]
chr11	85281311	85281378	[redacted]	-0.419846	[redacted]
chr11	76775682	76775774	[redacted]	-0.419812	[redacted]
chr11	109639333	109639424	[redacted]	-0.4198	[redacted]
chr14	103901915	103901950	[redacted]	-0.419786	[redacted]
chr6	85896045	85896078	[redacted]	-0.419704	[redacted]
chr5	73191643	73191715	[redacted]	-0.419673	[redacted]
chr15	39011393	39011451	[redacted]	-0.419652	[redacted]
chr3	116472110	116472178	[redacted]	-0.419565	[redacted]
chr17	19745127	19745189	[redacted]	-0.419552	[redacted]
chr1	3637620	3637689	[redacted]	-0.419541	[redacted]
chr6	34773543	34773555	[redacted]	-0.419511	[redacted]
chr13	93553913	93554008	[redacted]	-0.419502	[redacted]
chr15	93165485	93165545	[redacted]	-0.419493	[redacted]
chr11	118267869	118267920	[redacted]	-0.41947	[redacted]
chr17	83656966	83657012	[redacted]	-0.419452	[redacted]
chr9	47054095	47054125	[redacted]	-0.419439	[redacted]
chr15	102805114	102805175	[redacted]	-0.419412	[redacted]
chr15	97325598	97325609	[redacted]	-0.419347	[redacted]
chr14	88105866	88105913	[redacted]	-0.419343	[redacted]
chr6	54759691	54759780	[redacted]	-0.419228	[redacted]
chr9	26950660	26950684	[redacted]	-0.419214	[redacted]
chr15	61983039	61983104	[redacted]	-0.419213	[redacted]
chr12	20219637	20219699	[redacted]	-0.4192	[redacted]
chr12	69217689	69217743	[redacted]	-0.419112	[redacted]
chr4	81693390	81693441	[redacted]	-0.419086	[redacted]
chr6	99260642	99260665	[redacted]	-0.418988	[redacted]
chr11	50321546	50321590	[redacted]	-0.418928	[redacted]
chrX	139878217	139878316	[redacted]	-0.418907	[redacted]
chr3	135427501	135427563	[redacted]	-0.418869	[redacted]
chr16	11035061	11035100	[redacted]	-0.418849	[redacted]
chr6	86429711	86429764	[redacted]	-0.418841	[redacted]
chr14	120198013	120198095	[redacted]	-0.418789	[redacted]
chr10	4127491	4127548	[redacted]	-0.418765	[redacted]
chr2	162974140	162974171	[redacted]	-0.41876	[redacted]

chr9	44925790	44925827	[redacted]	-0.418671	[redacted]
chr18	33457964	33457988	[redacted]	-0.418661	[redacted]
chr16	45737992	45738044	[redacted]	-0.418629	[redacted]
chr15	80592562	80592673	[redacted]	-0.418623	[redacted]
chr6	149333518	149333567	[redacted]	-0.418498	[redacted]
chr15	52042080	52042142	[redacted]	-0.41847	[redacted]
chr3	141547368	141547417	[redacted]	-0.418462	[redacted]
chr10	26340934	26341024	[redacted]	-0.418357	[redacted]
chr6	89006996	89007025	[redacted]	-0.418344	[redacted]
chr10	121041273	121041301	[redacted]	-0.418331	[redacted]
chr13	19350528	19350568	[redacted]	-0.418327	[redacted]
chr6	84161518	84161598	[redacted]	-0.418308	[redacted]
chr18	77856768	77856869	[redacted]	-0.418239	[redacted]
chr3	53567678	53567757	[redacted]	-0.418199	[redacted]
chr11	62261232	62261318	[redacted]	-0.41805	[redacted]
chr17	62848723	62848738	[redacted]	-0.418023	[redacted]
chr8	83717674	83717731	[redacted]	-0.418021	[redacted]
chr1	59975320	59975339	[redacted]	-0.41802	[redacted]
chr6	120612464	120612506	[redacted]	-0.417978	[redacted]
chr8	41010893	41010934	[redacted]	-0.417932	[redacted]
chr1	184310480	184310528	[redacted]	-0.417927	[redacted]
chr1	185264232	185264320	[redacted]	-0.417927	[redacted]
chr7	40100275	40100322	[redacted]	-0.417885	[redacted]
chr2	167783815	167783865	[redacted]	-0.417884	[redacted]
chr6	71345107	71345133	[redacted]	-0.417758	[redacted]
chr7	112828516	112828555	[redacted]	-0.417694	[redacted]
chr16	38380178	38380202	[redacted]	-0.41764	[redacted]
chr3	9643445	9643501	[redacted]	-0.417625	[redacted]
chr11	82857715	82857792	[redacted]	-0.417623	[redacted]
chr2	76043941	76044034	[redacted]	-0.417579	[redacted]
chr6	31344793	31344871	[redacted]	-0.417528	[redacted]
chr18	5599440	5599504	[redacted]	-0.417474	[redacted]
chr8	86483557	86483583	[redacted]	-0.417443	[redacted]
chr10	91154410	91154481	[redacted]	-0.41743	[redacted]
chr5	139740822	139740882	[redacted]	-0.417425	[redacted]
chr3	22066242	22066301	[redacted]	-0.417404	[redacted]
chr1	98082840	98082920	[redacted]	-0.417339	[redacted]
chr15	37998311	37998379	[redacted]	-0.417308	[redacted]
chr6	142362479	142362505	[redacted]	-0.417301	[redacted]
chr2	167466769	167466827	[redacted]	-0.417291	[redacted]
chr13	111305832	111305874	[redacted]	-0.417246	[redacted]
chr5	73136695	73136755	[redacted]	-0.417237	[redacted]
chr7	63853712	63853781	[redacted]	-0.417181	[redacted]

chr10	114326855	114326929	[redacted]	-0.417136	[redacted]
chr12	56256415	56256435	[redacted]	-0.417085	[redacted]
chr2	107179598	107179633	[redacted]	-0.417046	[redacted]
chr8	113756077	113756094	[redacted]	-0.416975	[redacted]
chr8	125222346	125222396	[redacted]	-0.416975	[redacted]
chr15	4815108	4815147	[redacted]	-0.416926	[redacted]
chr19	4789141	4789185	[redacted]	-0.416838	[redacted]
chr7	68301076	68301135	[redacted]	-0.416829	[redacted]
chr4	152342698	152342741	[redacted]	-0.416821	[redacted]
chr3	99171913	99171985	[redacted]	-0.416793	[redacted]
chr12	114964215	114964256	[redacted]	-0.416774	[redacted]
chr7	33807925	33807951	[redacted]	-0.416714	[redacted]
chr17	82522351	82522435	[redacted]	-0.416702	[redacted]
chr3	86139639	86139706	[redacted]	-0.416689	[redacted]
chr11	79297363	79297413	[redacted]	-0.416662	[redacted]
chr18	49776788	49776841	[redacted]	-0.416628	[redacted]
chr15	94005447	94005497	[redacted]	-0.416584	[redacted]
chr5	21299089	21299134	[redacted]	-0.416529	[redacted]
chr9	109005334	109005407	[redacted]	-0.416516	[redacted]
chr19	57505747	57505812	[redacted]	-0.416464	[redacted]
chrX	166393232	166393300	[redacted]	-0.416366	[redacted]
chr13	81839589	81839645	[redacted]	-0.416363	[redacted]
chr10	25370461	25370505	[redacted]	-0.416326	[redacted]
chr8	47657535	47657607	[redacted]	-0.4163	[redacted]
chr12	101941979	101942027	[redacted]	-0.416297	[redacted]
chr9	118087047	118087120	[redacted]	-0.416294	[redacted]
chr6	120756665	120756818	[redacted]	-0.416266	[redacted]
chr15	83451361	83451386	[redacted]	-0.416262	[redacted]
chr11	50368552	50368593	[redacted]	-0.416261	[redacted]
chr7	30744844	30744885	[redacted]	-0.416239	[redacted]
chr4	117982457	117982524	[redacted]	-0.416161	[redacted]
chr13	93140618	93140648	[redacted]	-0.41615	[redacted]
chr18	54792639	54792676	[redacted]	-0.416135	[redacted]
chrX	98943619	98943679	[redacted]	-0.416131	[redacted]
chr9	64161266	64161380	[redacted]	-0.416125	[redacted]
chr14	33340219	33340254	[redacted]	-0.416123	[redacted]
chr12	14182304	14182382	[redacted]	-0.415995	[redacted]
chr11	53279333	53279404	[redacted]	-0.415977	[redacted]
chr14	99332986	99333071	[redacted]	-0.415972	[redacted]
chr13	46938779	46938786	[redacted]	-0.415965	[redacted]
chr3	55340568	55340619	[redacted]	-0.415897	[redacted]
chr7	127639240	127639320	[redacted]	-0.415887	[redacted]
chr10	79861644	79861675	[redacted]	-0.415883	[redacted]

chr12	99601099	99601173	[redacted]	-0.415844	[redacted]
chr8	119931950	119932002	[redacted]	-0.415772	[redacted]
chr10	26245568	26245617	[redacted]	-0.415749	[redacted]
chr18	10389953	10390049	[redacted]	-0.415733	[redacted]
chr5	142746919	142746976	[redacted]	-0.415688	[redacted]
chr8	9562483	9562545	[redacted]	-0.415631	[redacted]
chr6	133931450	133931463	[redacted]	-0.415621	[redacted]
chr5	34621552	34621565	[redacted]	-0.415613	[redacted]
chr18	36167617	36167651	[redacted]	-0.415603	[redacted]
chr17	42683075	42683107	[redacted]	-0.415594	[redacted]
chr18	18383629	18383666	[redacted]	-0.415567	[redacted]
chr5	106526951	106527011	[redacted]	-0.415551	[redacted]
chr15	55419494	55419579	[redacted]	-0.415462	[redacted]
chr10	40634580	40634672	[redacted]	-0.415461	[redacted]
chr13	63211878	63211910	[redacted]	-0.415439	[redacted]
chr13	112942806	112942861	[redacted]	-0.415422	[redacted]
chr12	13270341	13270386	[redacted]	-0.415364	[redacted]
chr16	21831590	21831639	[redacted]	-0.415196	[redacted]
chr15	29644848	29644931	[redacted]	-0.415195	[redacted]
chr5	107465094	107465172	[redacted]	-0.415194	[redacted]
chr8	64792948	64792987	[redacted]	-0.415173	[redacted]
chr12	34526724	34526778	[redacted]	-0.41514	[redacted]
chr8	46180087	46180130	[redacted]	-0.415031	[redacted]
chr1	56512556	56512686	[redacted]	-0.414969	[redacted]
chr16	96359807	96359879	[redacted]	-0.414919	[redacted]
chr5	73179147	73179222	[redacted]	-0.414881	[redacted]
chr17	32502386	32502442	[redacted]	-0.414867	[redacted]
chr4	148617393	148617434	[redacted]	-0.414854	[redacted]
chr2	158969324	158969424	[redacted]	-0.41485	[redacted]
chr9	98940395	98940439	[redacted]	-0.414747	[redacted]
chr11	79366664	79366701	[redacted]	-0.414731	[redacted]
chr6	97697536	97697626	[redacted]	-0.414672	[redacted]
chr13	111551151	111551206	[redacted]	-0.414606	[redacted]
chr19	6170703	6170769	[redacted]	-0.41459	[redacted]
chr7	25216824	25216846	[redacted]	-0.4145	[redacted]
chr10	21921541	21921597	[redacted]	-0.414469	[redacted]
chr5	92140952	92141009	[redacted]	-0.414446	[redacted]
chr2	7870983	7871046	[redacted]	-0.414414	[redacted]
chr3	106786300	106786345	[redacted]	-0.414412	[redacted]
chr1	38130924	38130941	[redacted]	-0.414406	[redacted]
chr15	103082011	103082060	[redacted]	-0.414375	[redacted]
chr18	8147284	8147389	[redacted]	-0.414356	[redacted]
chr10	60115021	60115051	[redacted]	-0.41427	[redacted]

chr3	36416726	36416760	[redacted]	-0.414266	[redacted]
chr7	28172277	28172349	[redacted]	-0.414183	[redacted]
chr1	192069441	192069492	[redacted]	-0.414149	[redacted]
chr11	65950027	65950095	[redacted]	-0.414144	[redacted]
chr17	56006869	56006926	[redacted]	-0.414137	[redacted]
chr16	36040470	36040543	[redacted]	-0.414128	[redacted]
chr12	99399800	99399885	[redacted]	-0.414109	[redacted]
chr10	84586152	84586211	[redacted]	-0.414031	[redacted]
chr1	187852811	187852895	[redacted]	-0.414029	[redacted]
chr4	134751367	134751496	[redacted]	-0.414018	[redacted]
chr15	37604003	37604019	[redacted]	-0.414015	[redacted]
chr4	31464211	31464249	[redacted]	-0.414009	[redacted]
chr8	65731618	65731665	[redacted]	-0.413977	[redacted]
chr6	57531136	57531210	[redacted]	-0.413954	[redacted]
chr15	27604100	27604166	[redacted]	-0.413947	[redacted]
chr10	96705788	96705846	[redacted]	-0.413835	[redacted]
chr17	45624193	45624228	[redacted]	-0.413825	[redacted]
chr3	122012160	122012211	[redacted]	-0.413767	[redacted]
chr10	13867975	13868053	[redacted]	-0.413754	[redacted]
chr8	9971660	9971724	[redacted]	-0.413735	[redacted]
chr17	51426692	51426783	[redacted]	-0.413681	[redacted]
chr6	97354485	97354603	[redacted]	-0.41361	[redacted]
chr2	172873984	172874048	[redacted]	-0.413597	[redacted]
chr7	28099018	28099057	[redacted]	-0.413584	[redacted]
chr1	74608835	74608912	[redacted]	-0.413576	[redacted]
chr4	34147343	34147399	[redacted]	-0.413559	[redacted]
chr10	19114780	19114813	[redacted]	-0.413536	[redacted]
chr4	127886965	127887032	[redacted]	-0.413525	[redacted]
chr4	90339414	90339472	[redacted]	-0.413457	[redacted]
chr9	103595211	103595276	[redacted]	-0.413435	[redacted]
chr14	47102909	47102944	[redacted]	-0.413404	[redacted]
chr16	48218765	48218797	[redacted]	-0.413392	[redacted]
chr7	99825406	99825470	[redacted]	-0.413391	[redacted]
chr4	114921205	114921256	[redacted]	-0.413375	[redacted]
chr15	73450692	73450723	[redacted]	-0.413357	[redacted]
chr3	58809835	58809899	[redacted]	-0.413331	[redacted]
chr16	6347072	6347135	[redacted]	-0.413326	[redacted]
chr10	68782627	68782695	[redacted]	-0.413263	[redacted]
chr4	102928829	102928891	[redacted]	-0.413253	[redacted]
chr17	49988895	49988949	[redacted]	-0.413223	[redacted]
chr2	27410939	27410950	[redacted]	-0.413194	[redacted]
chr10	117833098	117833134	[redacted]	-0.41318	[redacted]
chr8	77108000	77108027	[redacted]	-0.413177	[redacted]

chr5	72572770	72572860	[redacted]	-0.413109	[redacted]
chr9	100135960	100136026	[redacted]	-0.413081	[redacted]
chr12	70246148	70246242	[redacted]	-0.413069	[redacted]
chr2	163636907	163636936	[redacted]	-0.413052	[redacted]
chr3	108319785	108319853	[redacted]	-0.413045	[redacted]
chr8	69207729	69207771	[redacted]	-0.413	[redacted]
chr14	29291223	29291279	[redacted]	-0.412995	[redacted]
chr16	55999689	55999760	[redacted]	-0.412986	[redacted]
chr5	130745208	130745300	[redacted]	-0.412956	[redacted]
chr13	83306878	83306943	[redacted]	-0.412941	[redacted]
chr4	33697192	33697214	[redacted]	-0.412894	[redacted]
chr2	43795336	43795383	[redacted]	-0.412885	[redacted]
chr17	23786707	23786733	[redacted]	-0.412855	[redacted]
chr2	50117990	50118039	[redacted]	-0.412843	[redacted]
chr2	69045766	69045817	[redacted]	-0.412813	[redacted]
chr9	110138731	110138780	[redacted]	-0.412788	[redacted]
chr14	8473313	8473397	[redacted]	-0.412768	[redacted]
chr2	72084111	72084171	[redacted]	-0.412764	[redacted]
chr3	69377395	69377425	[redacted]	-0.412758	[redacted]
chr10	122737317	122737347	[redacted]	-0.412723	[redacted]
chr8	84254553	84254585	[redacted]	-0.412713	[redacted]
chr18	50012740	50012793	[redacted]	-0.412666	[redacted]
chr13	56458291	56458351	[redacted]	-0.412656	[redacted]
chr19	48700215	48700268	[redacted]	-0.412655	[redacted]
chr11	77340937	77341012	[redacted]	-0.412653	[redacted]
chr10	118114159	118114223	[redacted]	-0.412573	[redacted]
chr11	59481701	59481764	[redacted]	-0.412482	[redacted]
chr12	60293020	60293064	[redacted]	-0.412446	[redacted]
chr6	62138945	62139009	[redacted]	-0.412389	[redacted]
chr10	53259596	53259656	[redacted]	-0.412318	[redacted]
chr11	93925497	93925600	[redacted]	-0.412294	[redacted]
chr5	16152068	16152168	[redacted]	-0.412289	[redacted]
chr16	25485025	25485058	[redacted]	-0.412269	[redacted]
chr2	163701409	163701456	[redacted]	-0.41225	[redacted]
chr18	62945186	62945235	[redacted]	-0.412242	[redacted]
chr8	10212400	10212422	[redacted]	-0.412211	[redacted]
chr1	183653999	183654066	[redacted]	-0.412185	[redacted]
chr2	71043660	71043726	[redacted]	-0.41217	[redacted]
chr8	104685124	104685193	[redacted]	-0.412125	[redacted]
chr11	99432118	99432191	[redacted]	-0.412119	[redacted]
chr6	119665249	119665306	[redacted]	-0.412086	[redacted]
chr4	61789476	61789558	[redacted]	-0.412076	[redacted]
chr19	54081539	54081582	[redacted]	-0.412068	[redacted]

chr2	140383687	140383732	[redacted]	-0.412027	[redacted]
chr2	126387808	126387901	[redacted]	-0.41201	[redacted]
chr16	63185180	63185254	[redacted]	-0.411982	[redacted]
chr4	8732837	8732853	[redacted]	-0.411874	[redacted]
chr3	152238259	152238291	[redacted]	-0.411871	[redacted]
chr7	108302744	108302828	[redacted]	-0.411871	[redacted]
chr14	29788532	29788574	[redacted]	-0.411853	[redacted]
chr10	24128933	24128996	[redacted]	-0.411795	[redacted]
chr8	46952116	46952148	[redacted]	-0.411794	[redacted]
chr15	96752924	96752985	[redacted]	-0.411766	[redacted]
chr4	99074874	99074920	[redacted]	-0.411741	[redacted]
chr16	30338978	30339038	[redacted]	-0.411667	[redacted]
chr10	59502619	59502650	[redacted]	-0.411651	[redacted]
chr4	50169198	50169263	[redacted]	-0.411625	[redacted]
chr6	51450218	51450274	[redacted]	-0.411516	[redacted]
chr10	28462692	28462760	[redacted]	-0.411513	[redacted]
chr2	179899305	179899336	[redacted]	-0.41147	[redacted]
chr3	69184915	69184970	[redacted]	-0.4114	[redacted]
chr14	66157722	66157743	[redacted]	-0.411395	[redacted]
chr11	101970083	101970139	[redacted]	-0.411392	[redacted]
chr18	18680054	18680114	[redacted]	-0.411392	[redacted]
chr1	106140214	106140325	[redacted]	-0.411341	[redacted]
chr8	109664109	109664126	[redacted]	-0.411329	[redacted]
chr11	102141359	102141425	[redacted]	-0.411292	[redacted]
chr10	18444517	18444579	[redacted]	-0.411242	[redacted]
chr4	73212614	73212665	[redacted]	-0.411235	[redacted]
chr12	117515115	117515148	[redacted]	-0.411221	[redacted]
chr3	14877658	14877686	[redacted]	-0.411182	[redacted]
chr14	57468585	57468632	[redacted]	-0.411164	[redacted]
chr1	39425637	39425800	[redacted]	-0.411033	[redacted]
chr5	54012807	54012862	[redacted]	-0.41101	[redacted]
chr13	60833771	60833795	[redacted]	-0.411008	[redacted]
chr5	100045129	100045150	[redacted]	-0.41095	[redacted]
chr19	31963526	31963597	[redacted]	-0.410948	[redacted]
chr19	37799646	37799729	[redacted]	-0.410911	[redacted]
chr6	143742024	143742077	[redacted]	-0.410884	[redacted]
chr12	85281604	85281674	[redacted]	-0.41086	[redacted]
chr6	17098016	17098051	[redacted]	-0.410807	[redacted]
chr1	175898810	175898853	[redacted]	-0.410795	[redacted]
chr2	139955771	139955795	[redacted]	-0.410789	[redacted]
chr4	56212703	56212745	[redacted]	-0.410785	[redacted]
chr12	54746541	54746585	[redacted]	-0.410727	[redacted]
chr17	26240198	26240234	[redacted]	-0.41066	[redacted]

chr16	72804955	72805074	[redacted]	-0.410654	[redacted]
chr1	179433153	179433261	[redacted]	-0.410547	[redacted]
chr12	76184093	76184144	[redacted]	-0.410509	[redacted]
chr14	12328627	12328659	[redacted]	-0.410475	[redacted]
chr3	143724253	143724340	[redacted]	-0.410449	[redacted]
chr9	67491080	67491101	[redacted]	-0.410399	[redacted]
chr16	60349521	60349605	[redacted]	-0.410359	[redacted]
chr17	43024314	43024355	[redacted]	-0.410231	[redacted]
chr15	52489895	52489904	[redacted]	-0.410176	[redacted]
chr17	7861335	7861381	[redacted]	-0.410169	[redacted]
chr6	102371604	102371628	[redacted]	-0.410106	[redacted]
chr5	28385830	28385877	[redacted]	-0.410029	[redacted]
chr2	60759446	60759498	[redacted]	-0.40997	[redacted]
chr1	59500209	59500304	[redacted]	-0.409929	[redacted]
chr4	8832130	8832174	[redacted]	-0.409889	[redacted]
chr9	78490156	78490218	[redacted]	-0.409809	[redacted]
chr3	19163991	19164000	[redacted]	-0.409799	[redacted]
chr4	101339306	101339373	[redacted]	-0.409784	[redacted]
chr3	76336961	76337010	[redacted]	-0.409769	[redacted]
chr5	123872413	123872481	[redacted]	-0.409736	[redacted]
chr1	11520969	11521056	[redacted]	-0.409711	[redacted]
chr13	92614711	92614787	[redacted]	-0.409704	[redacted]
chr10	47324616	47324684	[redacted]	-0.409688	[redacted]
chr12	107731515	107731580	[redacted]	-0.409675	[redacted]
chr6	125217330	125217356	[redacted]	-0.409651	[redacted]
chrX	105905255	105905294	[redacted]	-0.409605	[redacted]
chr5	89174707	89174753	[redacted]	-0.409564	[redacted]
chr6	138948667	138948746	[redacted]	-0.409558	[redacted]
chr7	79996530	79996611	[redacted]	-0.409512	[redacted]
chr14	79476567	79476635	[redacted]	-0.409401	[redacted]
chr17	7211088	7211116	[redacted]	-0.409363	[redacted]
chr6	71423128	71423148	[redacted]	-0.409357	[redacted]
chr13	119466269	119466303	[redacted]	-0.409354	[redacted]
chr1	19199703	19199792	[redacted]	-0.409324	[redacted]
chrX	142372380	142372425	[redacted]	-0.409254	[redacted]
chr16	81511176	81511241	[redacted]	-0.409244	[redacted]
chr17	78113405	78113456	[redacted]	-0.409229	[redacted]
chr3	133061854	133061880	[redacted]	-0.409227	[redacted]
chr7	28985665	28985738	[redacted]	-0.409114	[redacted]
chr13	114146317	114146339	[redacted]	-0.40909	[redacted]
chr1	30829132	30829181	[redacted]	-0.409074	[redacted]
chr5	117622790	117622800	[redacted]	-0.409072	[redacted]
chr18	84736101	84736156	[redacted]	-0.409047	[redacted]

chr8	3661673	3661722	[redacted]	-0.409011	[redacted]
chr3	107903781	107903841	[redacted]	-0.408977	[redacted]
chr10	84354095	84354202	[redacted]	-0.408962	[redacted]
chr10	90392141	90392222	[redacted]	-0.408954	[redacted]
chr17	70186624	70186659	[redacted]	-0.408952	[redacted]
chr2	132885007	132885090	[redacted]	-0.408936	[redacted]
chr10	111593699	111593770	[redacted]	-0.408885	[redacted]
chr7	36754231	36754302	[redacted]	-0.40888	[redacted]
chr19	41180991	41181041	[redacted]	-0.408877	[redacted]
chr9	96689392	96689421	[redacted]	-0.408868	[redacted]
chr6	35173548	35173598	[redacted]	-0.408861	[redacted]
chr6	67224092	67224221	[redacted]	-0.408843	[redacted]
chr6	122679158	122679221	[redacted]	-0.408839	[redacted]
chr10	61469195	61469262	[redacted]	-0.40883	[redacted]
chr15	63811909	63811965	[redacted]	-0.408828	[redacted]
chr13	23893108	23893179	[redacted]	-0.408822	[redacted]
chr1	171245872	171245902	[redacted]	-0.408815	[redacted]
chr18	68344830	68344905	[redacted]	-0.4088	[redacted]
chr15	53162857	53162903	[redacted]	-0.408796	[redacted]
chr2	45833395	45833465	[redacted]	-0.408775	[redacted]
chr8	81938180	81938228	[redacted]	-0.408764	[redacted]
chr4	3651489	3651516	[redacted]	-0.408656	[redacted]
chr15	76913996	76914013	[redacted]	-0.408535	[redacted]
chr19	31415878	31415932	[redacted]	-0.40852	[redacted]
chr2	165936209	165936227	[redacted]	-0.408514	[redacted]
chr2	162838508	162838544	[redacted]	-0.408504	[redacted]
chr11	97161903	97161943	[redacted]	-0.408503	[redacted]
chr11	47809962	47810032	[redacted]	-0.408472	[redacted]
chr13	99019962	99020023	[redacted]	-0.408422	[redacted]
chr9	67596040	67596072	[redacted]	-0.408418	[redacted]
chr11	109209635	109209652	[redacted]	-0.408414	[redacted]
chr7	109275620	109275650	[redacted]	-0.408368	[redacted]
chr13	44518968	44519054	[redacted]	-0.408337	[redacted]
chr1	140354076	140354142	[redacted]	-0.408267	[redacted]
chr17	88920820	88920911	[redacted]	-0.40823	[redacted]
chr14	28031407	28031430	[redacted]	-0.408214	[redacted]
chr17	80499624	80499682	[redacted]	-0.408196	[redacted]
chrX	84668430	84668494	[redacted]	-0.408187	[redacted]
chr2	76852733	76852801	[redacted]	-0.40817	[redacted]
chr2	17868288	17868295	[redacted]	-0.408169	[redacted]
chr14	116055003	116055044	[redacted]	-0.408078	[redacted]
chr10	90624662	90624728	[redacted]	-0.408061	[redacted]
chr2	76257055	76257122	[redacted]	-0.408048	[redacted]

chr6	33052488	33052714	[redacted]	-0.408031	[redacted]
chr14	33879248	33879276	[redacted]	-0.40803	[redacted]
chr14	9886407	9886483	[redacted]	-0.407998	[redacted]
chr6	113346284	113346368	[redacted]	-0.40794	[redacted]
chr2	65175179	65175249	[redacted]	-0.407913	[redacted]
chr2	161083407	161083475	[redacted]	-0.407913	[redacted]
chr5	130269744	130269793	[redacted]	-0.407904	[redacted]
chr4	58836178	58836226	[redacted]	-0.407872	[redacted]
chr15	72914580	72914750	[redacted]	-0.407849	[redacted]
chr16	94537134	94537202	[redacted]	-0.407836	[redacted]
chr1	163900662	163900733	[redacted]	-0.407827	[redacted]
chr5	143710891	143711007	[redacted]	-0.407801	[redacted]
chr18	62924540	62924626	[redacted]	-0.4078	[redacted]
chr1	62888393	62888471	[redacted]	-0.407765	[redacted]
chr9	84124164	84124235	[redacted]	-0.407729	[redacted]
chr9	67786029	67786113	[redacted]	-0.407702	[redacted]
chr6	100723738	100723781	[redacted]	-0.407696	[redacted]
chr17	70455907	70455946	[redacted]	-0.407645	[redacted]
chr10	127411053	127411090	[redacted]	-0.407642	[redacted]
chr11	69403044	69403103	[redacted]	-0.407632	[redacted]
chr1	76080335	76080372	[redacted]	-0.407628	[redacted]
chr3	145962551	145962604	[redacted]	-0.407627	[redacted]
chr19	35644004	35644088	[redacted]	-0.407576	[redacted]
chr15	98503371	98503394	[redacted]	-0.407575	[redacted]
chr19	27186044	27186072	[redacted]	-0.407555	[redacted]
chr6	34206141	34206265	[redacted]	-0.407551	[redacted]
chr6	18931205	18931271	[redacted]	-0.407549	[redacted]
chr5	65239561	65239589	[redacted]	-0.407535	[redacted]
chr12	25744914	25744972	[redacted]	-0.407531	[redacted]
chr3	122939031	122939087	[redacted]	-0.407464	[redacted]
chr7	68851901	68851943	[redacted]	-0.407442	[redacted]
chr3	152779959	152780005	[redacted]	-0.407399	[redacted]
chr11	70529984	70530015	[redacted]	-0.407394	[redacted]
chr17	26241023	26241053	[redacted]	-0.407307	[redacted]
chr12	68525724	68525817	[redacted]	-0.407275	[redacted]
chr18	14279948	14279985	[redacted]	-0.40727	[redacted]
chr16	38506073	38506123	[redacted]	-0.407268	[redacted]
chr12	118476237	118476299	[redacted]	-0.407263	[redacted]
chr12	72704939	72704988	[redacted]	-0.407216	[redacted]
chr7	80795747	80795813	[redacted]	-0.407114	[redacted]
chr14	55141906	55142035	[redacted]	-0.407043	[redacted]
chr13	116885393	116885447	[redacted]	-0.407025	[redacted]
chr19	45253767	45253813	[redacted]	-0.406993	[redacted]

chr12	31255359	31255430	[redacted]	-0.406941	[redacted]
chr4	6844072	6844123	[redacted]	-0.40694	[redacted]
chr10	5532075	5532107	[redacted]	-0.406921	[redacted]
chr10	41272030	41272056	[redacted]	-0.406878	[redacted]
chr1	117157923	117158005	[redacted]	-0.406831	[redacted]
chr2	164103719	164103764	[redacted]	-0.40678	[redacted]
chr19	47483904	47483915	[redacted]	-0.406688	[redacted]
chr4	35357096	35357152	[redacted]	-0.406658	[redacted]
chr2	58748718	58748810	[redacted]	-0.4066	[redacted]
chr16	51887511	51887568	[redacted]	-0.40658	[redacted]
chrX	11575453	11575520	[redacted]	-0.406552	[redacted]
chr1	70997634	70997694	[redacted]	-0.406537	[redacted]
chr14	47247033	47247081	[redacted]	-0.40651	[redacted]
chr16	33495764	33495803	[redacted]	-0.406396	[redacted]
chr13	83770203	83770264	[redacted]	-0.406371	[redacted]
chr3	69254197	69254264	[redacted]	-0.40637	[redacted]
chr6	135404914	135404947	[redacted]	-0.406259	[redacted]
chr9	115383553	115383571	[redacted]	-0.406254	[redacted]
chr6	88100912	88100959	[redacted]	-0.406177	[redacted]
chr3	51390582	51390626	[redacted]	-0.406071	[redacted]
chr6	87119292	87119336	[redacted]	-0.406062	[redacted]
chr17	87109912	87109948	[redacted]	-0.406048	[redacted]
chr3	113161757	113161808	[redacted]	-0.406008	[redacted]
chr16	44533011	44533046	[redacted]	-0.406	[redacted]
chr17	43765844	43765887	[redacted]	-0.405992	[redacted]
chr1	52645929	52646018	[redacted]	-0.405976	[redacted]
chr16	20140533	20140546	[redacted]	-0.405942	[redacted]
chr19	45312178	45312233	[redacted]	-0.405911	[redacted]
chr17	25331629	25331663	[redacted]	-0.405896	[redacted]
chr17	54289946	54290029	[redacted]	-0.405843	[redacted]
chr2	168708082	168708156	[redacted]	-0.405828	[redacted]
chr10	93232445	93232522	[redacted]	-0.40582	[redacted]
chr4	111163603	111163668	[redacted]	-0.405748	[redacted]
chr2	30071863	30071902	[redacted]	-0.405732	[redacted]
chr1	45007348	45007380	[redacted]	-0.405726	[redacted]
chr10	59170792	59170935	[redacted]	-0.405722	[redacted]
chr1	158596406	158596440	[redacted]	-0.405717	[redacted]
chr9	55949439	55949464	[redacted]	-0.405698	[redacted]
chr5	147697746	147697839	[redacted]	-0.405581	[redacted]
chr5	134368401	134368470	[redacted]	-0.405552	[redacted]
chr3	130939110	130939144	[redacted]	-0.405549	[redacted]
chr2	158364230	158364367	[redacted]	-0.405505	[redacted]
chr3	107043311	107043336	[redacted]	-0.405474	[redacted]

chr13	81668666	81668760	[redacted]	-0.405374	[redacted]
chr11	70909569	70909728	[redacted]	-0.405339	[redacted]
chr2	145369129	145369224	[redacted]	-0.405226	[redacted]
chr13	97419823	97419858	[redacted]	-0.405224	[redacted]
chr11	55487907	55487935	[redacted]	-0.405207	[redacted]
chr7	133051667	133051741	[redacted]	-0.405163	[redacted]
chr10	18405558	18405585	[redacted]	-0.40516	[redacted]
chr2	6357428	6357523	[redacted]	-0.40514	[redacted]
chr11	51634906	51634916	[redacted]	-0.405128	[redacted]
chr15	93003110	93003172	[redacted]	-0.405039	[redacted]
chr3	77322011	77322048	[redacted]	-0.405037	[redacted]
chr15	84726337	84726369	[redacted]	-0.404996	[redacted]
chr6	55892587	55892652	[redacted]	-0.404925	[redacted]
chr1	66499665	66499709	[redacted]	-0.404907	[redacted]
chr4	8803934	8804005	[redacted]	-0.404903	[redacted]
chr12	48512365	48512429	[redacted]	-0.404893	[redacted]
chr18	59251092	59251100	[redacted]	-0.404816	[redacted]
chr6	133835573	133835608	[redacted]	-0.404798	[redacted]
chr4	136216230	136216281	[redacted]	-0.404796	[redacted]
chrX	9673127	9673159	[redacted]	-0.404786	[redacted]
chr17	14956003	14956059	[redacted]	-0.404678	[redacted]
chr1	73844500	73844531	[redacted]	-0.404669	[redacted]
chr17	36998000	36998039	[redacted]	-0.404668	[redacted]
chr5	145187924	145187958	[redacted]	-0.404557	[redacted]
chr4	11372343	11372387	[redacted]	-0.404549	[redacted]
chr5	139457470	139457529	[redacted]	-0.404516	[redacted]
chr9	45432976	45433037	[redacted]	-0.40449	[redacted]
chr16	59156064	59156124	[redacted]	-0.404465	[redacted]
chr10	53684854	53684926	[redacted]	-0.404444	[redacted]
chr15	40200843	40200902	[redacted]	-0.40438	[redacted]
chr9	82877896	82877936	[redacted]	-0.404376	[redacted]
chr3	39154601	39154621	[redacted]	-0.404355	[redacted]
chr6	33108858	33108891	[redacted]	-0.404354	[redacted]
chr11	112340100	112340158	[redacted]	-0.404334	[redacted]
chr5	124782945	124782977	[redacted]	-0.404291	[redacted]
chr13	116855646	116855704	[redacted]	-0.404242	[redacted]
chr12	54695045	54695098	[redacted]	-0.40421	[redacted]
chr4	45703437	45703469	[redacted]	-0.4042	[redacted]
chr10	88462685	88462698	[redacted]	-0.40416	[redacted]
chr4	142876741	142876762	[redacted]	-0.404135	[redacted]
chr8	34783033	34783066	[redacted]	-0.404133	[redacted]
chr9	109577251	109577263	[redacted]	-0.404113	[redacted]
chr4	129558953	129559002	[redacted]	-0.404044	[redacted]

chr6	108756091	108756123	[redacted]	-0.403959	[redacted]
chr1	132234174	132234236	[redacted]	-0.403939	[redacted]
chr17	87826112	87826162	[redacted]	-0.403902	[redacted]
chr13	109975518	109975609	[redacted]	-0.403895	[redacted]
chr4	107333201	107333223	[redacted]	-0.403871	[redacted]
chr9	58436500	58436560	[redacted]	-0.403862	[redacted]
chr13	93514213	93514273	[redacted]	-0.403861	[redacted]
chr14	68223526	68223581	[redacted]	-0.403748	[redacted]
chr15	66446465	66446485	[redacted]	-0.403736	[redacted]
chr18	65213959	65214008	[redacted]	-0.403732	[redacted]
chr10	62185116	62185184	[redacted]	-0.403732	[redacted]
chr18	60990069	60990085	[redacted]	-0.403718	[redacted]
chr1	20501498	20501596	[redacted]	-0.403715	[redacted]
chr2	164106411	164106465	[redacted]	-0.403713	[redacted]
chrX	142692023	142692137	[redacted]	-0.40361	[redacted]
chr17	65284251	65284307	[redacted]	-0.403587	[redacted]
chr13	61791788	61791844	[redacted]	-0.403533	[redacted]
chr12	76772724	76772785	[redacted]	-0.40348	[redacted]
chr14	100287600	100287638	[redacted]	-0.403473	[redacted]
chr13	52137352	52137389	[redacted]	-0.403437	[redacted]
chr6	15531741	15531785	[redacted]	-0.403345	[redacted]
chr2	43855103	43855214	[redacted]	-0.403215	[redacted]
chr6	39746418	39746481	[redacted]	-0.403161	[redacted]
chr15	91754063	91754103	[redacted]	-0.403154	[redacted]
chr16	76454213	76454241	[redacted]	-0.403149	[redacted]
chr3	135089107	135089176	[redacted]	-0.403127	[redacted]
chr7	81826230	81826287	[redacted]	-0.403114	[redacted]
chr13	63560775	63560813	[redacted]	-0.403111	[redacted]
chr18	4195749	4195783	[redacted]	-0.40309	[redacted]
chr3	129562798	129562863	[redacted]	-0.40307	[redacted]
chr6	143066171	143066211	[redacted]	-0.40306	[redacted]
chr10	76425691	76425788	[redacted]	-0.403058	[redacted]
chr11	117483871	117483908	[redacted]	-0.403038	[redacted]
chr15	94313998	94314050	[redacted]	-0.403027	[redacted]
chr15	78019310	78019374	[redacted]	-0.403012	[redacted]
chr2	152614757	152614823	[redacted]	-0.403004	[redacted]
chr2	11393602	11393645	[redacted]	-0.402989	[redacted]
chr14	54381799	54381861	[redacted]	-0.402979	[redacted]
chr5	31008485	31008579	[redacted]	-0.402916	[redacted]
chr2	156156013	156156063	[redacted]	-0.402906	[redacted]
chr2	101893223	101893247	[redacted]	-0.402882	[redacted]
chr11	19930415	19930510	[redacted]	-0.402867	[redacted]
chr8	14482227	14482320	[redacted]	-0.402848	[redacted]

chr2	159370216	159370254	[redacted]	-0.402823	[redacted]
chr6	32931591	32931698	[redacted]	-0.402814	[redacted]
chr11	87235263	87235305	[redacted]	-0.40281	[redacted]
chr12	75931138	75931180	[redacted]	-0.402804	[redacted]
chr2	144312347	144312425	[redacted]	-0.402773	[redacted]
chr19	28782555	28782621	[redacted]	-0.402772	[redacted]
chr9	47779388	47779473	[redacted]	-0.402744	[redacted]
chr14	25100428	25100466	[redacted]	-0.402678	[redacted]
chr3	12578864	12578935	[redacted]	-0.402609	[redacted]
chr15	53259947	53259976	[redacted]	-0.402597	[redacted]
chr19	55414311	55414355	[redacted]	-0.40257	[redacted]
chr10	11227399	11227465	[redacted]	-0.402553	[redacted]
chr15	96996162	96996207	[redacted]	-0.402533	[redacted]
chr4	32427268	32427384	[redacted]	-0.402463	[redacted]
chr10	72882374	72882443	[redacted]	-0.402447	[redacted]
chr18	82518722	82518741	[redacted]	-0.402436	[redacted]
chr11	95901855	95901905	[redacted]	-0.402355	[redacted]
chr12	19840180	19840209	[redacted]	-0.402355	[redacted]
chr15	80585878	80585927	[redacted]	-0.402327	[redacted]
chr6	125564211	125564276	[redacted]	-0.40232	[redacted]
chr4	109927176	109927243	[redacted]	-0.402293	[redacted]
chr5	99850140	99850196	[redacted]	-0.40222	[redacted]
chr12	59152100	59152130	[redacted]	-0.402199	[redacted]
chr16	35204760	35204796	[redacted]	-0.402168	[redacted]
chr3	130883451	130883513	[redacted]	-0.402153	[redacted]
chr4	118251969	118252099	[redacted]	-0.40208	[redacted]
chr10	12540268	12540310	[redacted]	-0.402078	[redacted]
chr5	104539694	104539745	[redacted]	-0.402078	[redacted]
chr14	78779018	78779053	[redacted]	-0.401978	[redacted]
chr14	120535600	120535646	[redacted]	-0.401978	[redacted]
chr14	88753611	88753674	[redacted]	-0.401919	[redacted]
chr3	54988975	54989038	[redacted]	-0.401896	[redacted]
chr3	103708718	103708754	[redacted]	-0.401887	[redacted]
chr4	50436668	50436770	[redacted]	-0.401853	[redacted]
chr10	115372707	115372739	[redacted]	-0.401825	[redacted]
chr9	72665782	72665872	[redacted]	-0.4018	[redacted]
chr7	28747141	28747190	[redacted]	-0.401784	[redacted]
chr10	21266457	21266541	[redacted]	-0.401748	[redacted]
chr6	8930543	8930563	[redacted]	-0.40173	[redacted]
chr4	107363726	107363771	[redacted]	-0.401714	[redacted]
chr7	67935772	67935835	[redacted]	-0.401709	[redacted]
chr12	68766785	68766861	[redacted]	-0.401703	[redacted]
chr10	94110819	94110868	[redacted]	-0.401701	[redacted]

chrX	159712785	159712839	[redacted]	-0.401679	[redacted]
chr17	41547766	41547815	[redacted]	-0.401655	[redacted]
chr13	107784025	107784090	[redacted]	-0.401618	[redacted]
chr12	15644230	15644261	[redacted]	-0.401599	[redacted]
chr4	11931473	11931524	[redacted]	-0.401585	[redacted]
chr3	142175848	142175924	[redacted]	-0.401575	[redacted]
chr1	60243670	60243762	[redacted]	-0.401513	[redacted]
chr18	3471729	3471772	[redacted]	-0.401471	[redacted]
chr10	127031836	127031898	[redacted]	-0.401459	[redacted]
chr4	123713682	123713749	[redacted]	-0.401446	[redacted]
chr11	95453274	95453353	[redacted]	-0.401439	[redacted]
chr7	29288769	29288836	[redacted]	-0.401421	[redacted]
chr4	15233716	15233769	[redacted]	-0.401415	[redacted]
chr13	44203607	44203645	[redacted]	-0.401373	[redacted]
chr13	11588109	11588216	[redacted]	-0.401358	[redacted]
chr12	86535666	86535756	[redacted]	-0.401354	[redacted]
chr12	111883572	111883577	[redacted]	-0.40133	[redacted]
chr3	145505317	145505402	[redacted]	-0.401302	[redacted]
chrX	13357285	13357352	[redacted]	-0.401278	[redacted]
chr6	50633821	50633872	[redacted]	-0.401277	[redacted]
chr1	36680773	36680781	[redacted]	-0.401244	[redacted]
chr12	16158009	16158036	[redacted]	-0.401191	[redacted]
chr4	141191497	141191528	[redacted]	-0.401137	[redacted]
chr6	71399183	71399277	[redacted]	-0.401131	[redacted]
chr18	37364489	37364519	[redacted]	-0.401099	[redacted]
chr17	41901000	41901036	[redacted]	-0.401037	[redacted]
chr5	16618898	16618938	[redacted]	-0.401	[redacted]
chr6	4830409	4830467	[redacted]	-0.400998	[redacted]
chr9	96011002	96011053	[redacted]	-0.400923	[redacted]
chr1	34075282	34075369	[redacted]	-0.400895	[redacted]
chr5	118083966	118084024	[redacted]	-0.400876	[redacted]
chr15	96169667	96169708	[redacted]	-0.400839	[redacted]
chr10	101248337	101248415	[redacted]	-0.400816	[redacted]
chr19	14670275	14670314	[redacted]	-0.400791	[redacted]
chr1	55434609	55434635	[redacted]	-0.400777	[redacted]
chr2	64945178	64945255	[redacted]	-0.400756	[redacted]
chr2	104384737	104384806	[redacted]	-0.400753	[redacted]
chr16	87349740	87349809	[redacted]	-0.400741	[redacted]
chr7	6274057	6274098	[redacted]	-0.400723	[redacted]
chr17	87531838	87531856	[redacted]	-0.400684	[redacted]
chr15	67382551	67382628	[redacted]	-0.400677	[redacted]
chr6	24224191	24224273	[redacted]	-0.400661	[redacted]
chr7	86546590	86546654	[redacted]	-0.40063	[redacted]

chr11	98521408	98521488	[redacted]	-0.400618	[redacted]
chr4	119950109	119950178	[redacted]	-0.400579	[redacted]
chr19	8699980	8700031	[redacted]	-0.400538	[redacted]
chr19	31070873	31070949	[redacted]	-0.40048	[redacted]
chr2	72407401	72407421	[redacted]	-0.400447	[redacted]
chr10	20223430	20223568	[redacted]	-0.400437	[redacted]
chr7	68547133	68547163	[redacted]	-0.400391	[redacted]
chr10	70176538	70176620	[redacted]	-0.400316	[redacted]
chr1	79426219	79426266	[redacted]	-0.400246	[redacted]
chr3	97082085	97082264	[redacted]	-0.400227	[redacted]
chr15	81572330	81572367	[redacted]	-0.400224	[redacted]
chr4	14765615	14765659	[redacted]	-0.400218	[redacted]
chrX	142570313	142570405	[redacted]	-0.400208	[redacted]
chr3	24743113	24743133	[redacted]	-0.400196	[redacted]
chr1	62619799	62619841	[redacted]	-0.400189	[redacted]
chr8	104416342	104416396	[redacted]	-0.400079	[redacted]
chr5	46243723	46243767	[redacted]	-0.40005	[redacted]
chr6	114226768	114226800	[redacted]	-0.400037	[redacted]
chr1	95844044	95844132	[redacted]	-0.400033	[redacted]
chr16	6601001	6601059	[redacted]	-0.399994	[redacted]
chr12	76687388	76687482	[redacted]	-0.399939	[redacted]
chr17	43362396	43362404	[redacted]	-0.399936	[redacted]
chrX	83049077	83049157	[redacted]	-0.39992	[redacted]
chr1	39813010	39813094	[redacted]	-0.39991	[redacted]
chr7	59467046	59467106	[redacted]	-0.399904	[redacted]
chr18	45676945	45677040	[redacted]	-0.39988	[redacted]
chr4	131030097	131030171	[redacted]	-0.39984	[redacted]
chr9	46589289	46589330	[redacted]	-0.399837	[redacted]
chr12	70943451	70943495	[redacted]	-0.399836	[redacted]
chr19	29491380	29491438	[redacted]	-0.39982	[redacted]
chr6	125100879	125100990	[redacted]	-0.399809	[redacted]
chr2	65078378	65078433	[redacted]	-0.399809	[redacted]
chr6	71361646	71361658	[redacted]	-0.399738	[redacted]
chr19	49961026	49961111	[redacted]	-0.399693	[redacted]
chr4	44048466	44048491	[redacted]	-0.399682	[redacted]
chr3	131991759	131991817	[redacted]	-0.399681	[redacted]
chr16	49896411	49896468	[redacted]	-0.399674	[redacted]
chr19	41312676	41312729	[redacted]	-0.399672	[redacted]
chr14	56884497	56884597	[redacted]	-0.399652	[redacted]
chr17	85173481	85173525	[redacted]	-0.399632	[redacted]
chr4	43395621	43395681	[redacted]	-0.399607	[redacted]
chr1	149196526	149196569	[redacted]	-0.399577	[redacted]
chr11	62641669	62641721	[redacted]	-0.399476	[redacted]

chr15	61693918	61694007	[redacted]	-0.399409	[redacted]
chr4	144498988	144499029	[redacted]	-0.399392	[redacted]
chr18	46974012	46974034	[redacted]	-0.399362	[redacted]
chr5	15944902	15944961	[redacted]	-0.399354	[redacted]
chr11	51601458	51601539	[redacted]	-0.399345	[redacted]
chrX	140706287	140706345	[redacted]	-0.399316	[redacted]
chr15	87879926	87879971	[redacted]	-0.399302	[redacted]
chr3	50298342	50298393	[redacted]	-0.399299	[redacted]
chrX	41684077	41684157	[redacted]	-0.399262	[redacted]
chr2	10701467	10701484	[redacted]	-0.399258	[redacted]
chr10	129342505	129342587	[redacted]	-0.399223	[redacted]
chr11	98996755	98996793	[redacted]	-0.399182	[redacted]
chr4	140848549	140848590	[redacted]	-0.399165	[redacted]
chr4	69352141	69352186	[redacted]	-0.399164	[redacted]
chr2	80338468	80338481	[redacted]	-0.399161	[redacted]
chr10	82500497	82500518	[redacted]	-0.399161	[redacted]
chr19	27365367	27365407	[redacted]	-0.399126	[redacted]
chr16	76817966	76818049	[redacted]	-0.399104	[redacted]
chr16	10805878	10805932	[redacted]	-0.399087	[redacted]
chr4	52428633	52428676	[redacted]	-0.399051	[redacted]
chr2	140420920	140420968	[redacted]	-0.399036	[redacted]
chr17	3164600	3164657	[redacted]	-0.399033	[redacted]
chr12	83391867	83391948	[redacted]	-0.399031	[redacted]
chr9	122621971	122622044	[redacted]	-0.398989	[redacted]
chr10	68405779	68405835	[redacted]	-0.398942	[redacted]
chr2	38864083	38864135	[redacted]	-0.398937	[redacted]
chrX	21296813	21296840	[redacted]	-0.398855	[redacted]
chr6	72092157	72092234	[redacted]	-0.398854	[redacted]
chr6	135846041	135846090	[redacted]	-0.398786	[redacted]
chr18	15272512	15272562	[redacted]	-0.398777	[redacted]
chr8	67340215	67340309	[redacted]	-0.39876	[redacted]
chr12	71631689	71631721	[redacted]	-0.398741	[redacted]
chr12	112015005	112015049	[redacted]	-0.39873	[redacted]
chr17	44165731	44165762	[redacted]	-0.398723	[redacted]
chr5	57742043	57742099	[redacted]	-0.398691	[redacted]
chr4	86133423	86133489	[redacted]	-0.398644	[redacted]
chr2	169664028	169664047	[redacted]	-0.398641	[redacted]
chr16	76301429	76301484	[redacted]	-0.398607	[redacted]
chr6	127465554	127465595	[redacted]	-0.398573	[redacted]
chr14	31636709	31636784	[redacted]	-0.39854	[redacted]
chr1	71067074	71067147	[redacted]	-0.39853	[redacted]
chr13	90679936	90680012	[redacted]	-0.398525	[redacted]
chr15	43435899	43435934	[redacted]	-0.398524	[redacted]

chr5	130246362	130246450	[redacted]	-0.39851	[redacted]
chr6	95454347	95454389	[redacted]	-0.3985	[redacted]
chr8	83633918	83633977	[redacted]	-0.398491	[redacted]
chr11	46385846	46385946	[redacted]	-0.398417	[redacted]
chr16	19189863	19189928	[redacted]	-0.398375	[redacted]
chrX	52636281	52636326	[redacted]	-0.398375	[redacted]
chr2	19290177	19290191	[redacted]	-0.398372	[redacted]
chr10	103387238	103387310	[redacted]	-0.398355	[redacted]
chr18	69249703	69249737	[redacted]	-0.398332	[redacted]
chr2	4670132	4670189	[redacted]	-0.398325	[redacted]
chr15	63949198	63949205	[redacted]	-0.398316	[redacted]
chr19	7608668	7608756	[redacted]	-0.398286	[redacted]
chr10	117873480	117873547	[redacted]	-0.398276	[redacted]
chr8	36716636	36716688	[redacted]	-0.398211	[redacted]
chr13	112709470	112709498	[redacted]	-0.398179	[redacted]
chr6	139772463	139772523	[redacted]	-0.398153	[redacted]
chr2	62456050	62456088	[redacted]	-0.398114	[redacted]
chr10	122661313	122661359	[redacted]	-0.3981	[redacted]
chr19	35201988	35202072	[redacted]	-0.398094	[redacted]
chr1	191372777	191372863	[redacted]	-0.398063	[redacted]
chr10	62856694	62856781	[redacted]	-0.39806	[redacted]
chr12	107524270	107524342	[redacted]	-0.398042	[redacted]
chr8	77316996	77317026	[redacted]	-0.398034	[redacted]
chr9	58706496	58706506	[redacted]	-0.397983	[redacted]
chr17	40780912	40780951	[redacted]	-0.397923	[redacted]
chr3	87253483	87253516	[redacted]	-0.397889	[redacted]
chr13	45939209	45939263	[redacted]	-0.397886	[redacted]
chr9	98073024	98073080	[redacted]	-0.397819	[redacted]
chr4	133734172	133734190	[redacted]	-0.3978	[redacted]
chr6	33035578	33035597	[redacted]	-0.397787	[redacted]
chr13	58861253	58861273	[redacted]	-0.397778	[redacted]
chr6	37037485	37037519	[redacted]	-0.397764	[redacted]
chr13	31348761	31348828	[redacted]	-0.397763	[redacted]
chr1	44071266	44071350	[redacted]	-0.397709	[redacted]
chr2	73944756	73944794	[redacted]	-0.397706	[redacted]
chr16	21439442	21439472	[redacted]	-0.397705	[redacted]
chr19	31627445	31627503	[redacted]	-0.397701	[redacted]
chr5	132171113	132171220	[redacted]	-0.397657	[redacted]
chr16	60925783	60925801	[redacted]	-0.39763	[redacted]
chr2	151921310	151921349	[redacted]	-0.397627	[redacted]
chr11	45893713	45893744	[redacted]	-0.397615	[redacted]
chr7	80783144	80783170	[redacted]	-0.397512	[redacted]
chr5	97904533	97904568	[redacted]	-0.397497	[redacted]

chr13	49967285	49967356	[redacted]	-0.397451	[redacted]
chr12	40203396	40203421	[redacted]	-0.397433	[redacted]
chr11	83967887	83967941	[redacted]	-0.397419	[redacted]
chr13	36310118	36310190	[redacted]	-0.397419	[redacted]
chr2	141873186	141873252	[redacted]	-0.397335	[redacted]
chr16	87273064	87273094	[redacted]	-0.397333	[redacted]
chr10	40880107	40880159	[redacted]	-0.397327	[redacted]
chr1	105180624	105180701	[redacted]	-0.397283	[redacted]
chr3	25162324	25162355	[redacted]	-0.39728	[redacted]
chr13	113825801	113825849	[redacted]	-0.397279	[redacted]
chr9	99071432	99071505	[redacted]	-0.397267	[redacted]
chr9	41858547	41858608	[redacted]	-0.397244	[redacted]
chr3	63009035	63009048	[redacted]	-0.397207	[redacted]
chr16	92759444	92759458	[redacted]	-0.3972	[redacted]
chr7	105822512	105822593	[redacted]	-0.397188	[redacted]
chr3	37780296	37780388	[redacted]	-0.397153	[redacted]
chr6	112337496	112337536	[redacted]	-0.397118	[redacted]
chr11	45409957	45410037	[redacted]	-0.3971	[redacted]
chr10	99836181	99836244	[redacted]	-0.39709	[redacted]
chr9	13660644	13660686	[redacted]	-0.397045	[redacted]
chr13	106760272	106760285	[redacted]	-0.397019	[redacted]
chr2	40797323	40797370	[redacted]	-0.397001	[redacted]
chrX	13288789	13288924	[redacted]	-0.397	[redacted]
chr19	14931569	14931641	[redacted]	-0.396999	[redacted]
chr3	102899831	102899844	[redacted]	-0.396987	[redacted]
chr12	92652209	92652268	[redacted]	-0.396985	[redacted]
chr14	79290783	79290821	[redacted]	-0.396964	[redacted]
chr5	146956894	146956932	[redacted]	-0.396929	[redacted]
chr8	80968941	80969003	[redacted]	-0.396924	[redacted]
chr10	115309678	115309696	[redacted]	-0.39692	[redacted]
chr5	76662032	76662070	[redacted]	-0.396915	[redacted]
chr18	83708258	83708324	[redacted]	-0.396914	[redacted]
chr1	40869420	40869494	[redacted]	-0.396888	[redacted]
chr6	87125498	87125561	[redacted]	-0.396883	[redacted]
chr6	22553450	22553544	[redacted]	-0.396864	[redacted]
chr6	98579104	98579126	[redacted]	-0.396849	[redacted]
chr1	66944744	66944772	[redacted]	-0.396832	[redacted]
chr10	85104264	85104292	[redacted]	-0.396804	[redacted]
chr17	10767793	10767847	[redacted]	-0.396794	[redacted]
chr3	123012690	123012732	[redacted]	-0.396785	[redacted]
chr2	150824830	150824879	[redacted]	-0.396627	[redacted]
chr3	148811684	148811714	[redacted]	-0.396616	[redacted]
chr5	65877628	65877670	[redacted]	-0.396577	[redacted]

chr15	38584659	38584686	[redacted]	-0.396571	[redacted]
chr11	119140571	119140649	[redacted]	-0.396532	[redacted]
chr5	112194632	112194719	[redacted]	-0.396511	[redacted]
chr1	95262995	95263016	[redacted]	-0.396468	[redacted]
chr14	38757348	38757416	[redacted]	-0.396466	[redacted]
chr2	94692552	94692591	[redacted]	-0.396441	[redacted]
chr6	55147367	55147411	[redacted]	-0.396417	[redacted]
chr5	144964154	144964186	[redacted]	-0.396416	[redacted]
chr15	59741767	59741848	[redacted]	-0.396389	[redacted]
chr17	44460033	44460056	[redacted]	-0.396337	[redacted]
chr8	123027917	123027990	[redacted]	-0.396332	[redacted]
chr9	101006973	101007043	[redacted]	-0.396317	[redacted]
chr10	17296522	17296577	[redacted]	-0.396307	[redacted]
chr2	69204808	69204856	[redacted]	-0.396298	[redacted]
chr1	38562395	38562443	[redacted]	-0.396281	[redacted]
chr1	163682068	163682142	[redacted]	-0.396267	[redacted]
chr11	19404437	19404471	[redacted]	-0.396265	[redacted]
chr15	44380350	44380409	[redacted]	-0.396259	[redacted]
chr19	38947521	38947574	[redacted]	-0.39623	[redacted]
chr11	117590210	117590289	[redacted]	-0.396228	[redacted]
chr17	40901560	40901568	[redacted]	-0.396203	[redacted]
chr17	30548519	30548583	[redacted]	-0.396109	[redacted]
chr2	3227166	3227195	[redacted]	-0.396101	[redacted]
chr15	41716994	41717068	[redacted]	-0.396079	[redacted]
chr2	155163273	155163317	[redacted]	-0.396069	[redacted]
chr16	11169356	11169424	[redacted]	-0.396067	[redacted]
chr11	6584408	6584467	[redacted]	-0.396054	[redacted]
chr13	57786076	57786106	[redacted]	-0.396025	[redacted]
chr7	65742724	65742744	[redacted]	-0.396011	[redacted]
chr13	118383135	118383193	[redacted]	-0.395989	[redacted]
chr7	46790882	46790925	[redacted]	-0.39592	[redacted]
chr8	94013352	94013459	[redacted]	-0.395909	[redacted]
chr16	47065492	47065550	[redacted]	-0.395891	[redacted]
chr9	51272444	51272504	[redacted]	-0.395891	[redacted]
chr10	34213495	34213538	[redacted]	-0.39584	[redacted]
chr11	31277113	31277147	[redacted]	-0.395831	[redacted]
chr8	116086129	116086153	[redacted]	-0.395816	[redacted]
chr9	105930960	105930974	[redacted]	-0.395816	[redacted]
chr13	49437393	49437459	[redacted]	-0.395773	[redacted]
chr16	76948144	76948209	[redacted]	-0.395644	[redacted]
chr12	82961596	82961630	[redacted]	-0.395548	[redacted]
chr9	80172963	80172981	[redacted]	-0.39554	[redacted]
chr4	66838472	66838539	[redacted]	-0.395489	[redacted]

chr14	98141242	98141318	[redacted]	-0.395438	[redacted]
chr6	6187228	6187279	[redacted]	-0.395438	[redacted]
chr1	116813621	116813703	[redacted]	-0.395413	[redacted]
chr15	6593890	6593958	[redacted]	-0.395406	[redacted]
chr1	7531926	7531991	[redacted]	-0.395395	[redacted]
chr1	43473260	43473283	[redacted]	-0.395332	[redacted]
chr14	49682318	49682357	[redacted]	-0.395326	[redacted]
chr14	60465930	60466012	[redacted]	-0.3953	[redacted]
chr2	125104891	125104909	[redacted]	-0.395266	[redacted]
chr1	132870199	132870243	[redacted]	-0.395263	[redacted]
chr1	60243904	60243945	[redacted]	-0.395259	[redacted]
chr11	33576908	33576969	[redacted]	-0.395246	[redacted]
chr4	116691830	116691895	[redacted]	-0.39519	[redacted]
chr4	33300504	33300532	[redacted]	-0.395168	[redacted]
chr10	120611162	120611233	[redacted]	-0.395154	[redacted]
chrX	53020991	53021070	[redacted]	-0.395136	[redacted]
chr4	102967463	102967515	[redacted]	-0.395125	[redacted]
chr15	80592452	80592499	[redacted]	-0.395106	[redacted]
chr5	103397930	103397982	[redacted]	-0.395106	[redacted]
chr5	122786601	122786671	[redacted]	-0.395006	[redacted]
chr11	46266260	46266319	[redacted]	-0.394998	[redacted]
chr18	67458649	67458751	[redacted]	-0.394958	[redacted]
chr11	105484956	105485021	[redacted]	-0.394923	[redacted]
chr2	159273962	159274011	[redacted]	-0.394807	[redacted]
chr6	108217150	108217203	[redacted]	-0.394806	[redacted]
chr14	100613586	100613648	[redacted]	-0.394803	[redacted]
chr3	59227462	59227495	[redacted]	-0.394767	[redacted]
chr9	115259112	115259140	[redacted]	-0.39475	[redacted]
chr15	62653571	62653622	[redacted]	-0.394724	[redacted]
chr9	101401793	101401843	[redacted]	-0.394722	[redacted]
chr2	140168299	140168372	[redacted]	-0.394657	[redacted]
chr4	54990785	54990824	[redacted]	-0.39464	[redacted]
chr13	92800059	92800125	[redacted]	-0.394587	[redacted]
chrX	56857060	56857115	[redacted]	-0.394587	[redacted]
chr14	62966950	62967055	[redacted]	-0.394579	[redacted]
chr7	100382813	100382890	[redacted]	-0.394565	[redacted]
chr14	61932976	61933031	[redacted]	-0.394553	[redacted]
chr1	164830986	164831029	[redacted]	-0.394529	[redacted]
chr5	137839669	137839728	[redacted]	-0.394525	[redacted]
chr9	56421420	56421476	[redacted]	-0.39452	[redacted]
chr5	118570142	118570238	[redacted]	-0.394486	[redacted]
chr14	76599637	76599658	[redacted]	-0.394413	[redacted]
chr12	105987129	105987159	[redacted]	-0.394408	[redacted]

chr10	20646071	20646153	[redacted]	-0.3944	[redacted]
chr12	39121017	39121092	[redacted]	-0.394323	[redacted]
chr9	100543305	100543342	[redacted]	-0.394302	[redacted]
chr10	120005953	120006014	[redacted]	-0.394279	[redacted]
chr2	61406034	61406107	[redacted]	-0.394222	[redacted]
chr11	75848902	75848961	[redacted]	-0.394217	[redacted]
chr15	68096837	68096870	[redacted]	-0.394191	[redacted]
chr19	45892958	45893009	[redacted]	-0.394113	[redacted]
chr5	58942237	58942312	[redacted]	-0.394099	[redacted]
chr12	41044518	41044556	[redacted]	-0.394086	[redacted]
chr16	75313372	75313452	[redacted]	-0.394058	[redacted]
chr13	43607610	43607689	[redacted]	-0.394043	[redacted]
chr4	98495043	98495094	[redacted]	-0.394011	[redacted]
chr19	31198625	31198698	[redacted]	-0.39399	[redacted]
chr13	30147704	30147727	[redacted]	-0.39395	[redacted]
chr6	140141484	140141545	[redacted]	-0.393903	[redacted]
chr8	46949295	46949485	[redacted]	-0.393902	[redacted]
chr13	9552969	9553004	[redacted]	-0.393879	[redacted]
chr9	40974603	40974657	[redacted]	-0.393845	[redacted]
chr4	137599777	137599850	[redacted]	-0.393836	[redacted]
chr7	143664165	143664223	[redacted]	-0.39383	[redacted]
chr11	114785919	114786049	[redacted]	-0.393814	[redacted]
chr2	61269155	61269220	[redacted]	-0.393801	[redacted]
chr19	30120695	30120764	[redacted]	-0.393783	[redacted]
chrX	7142447	7142506	[redacted]	-0.393768	[redacted]
chr3	78174754	78174825	[redacted]	-0.393744	[redacted]
chr4	148586856	148586970	[redacted]	-0.39374	[redacted]
chr16	22454441	22454524	[redacted]	-0.393739	[redacted]
chr8	124347108	124347164	[redacted]	-0.393737	[redacted]
chr8	39086405	39086497	[redacted]	-0.393728	[redacted]
chr8	64842296	64842392	[redacted]	-0.393719	[redacted]
chr1	137328762	137328868	[redacted]	-0.393633	[redacted]
chr15	5276534	5276588	[redacted]	-0.393611	[redacted]
chr13	32424668	32424760	[redacted]	-0.393603	[redacted]
chrX	101754973	101754983	[redacted]	-0.393561	[redacted]
chr12	101974458	101974472	[redacted]	-0.393527	[redacted]
chr3	135297773	135297848	[redacted]	-0.393465	[redacted]
chr6	103600217	103600286	[redacted]	-0.393438	[redacted]
chr13	98903263	98903283	[redacted]	-0.393423	[redacted]
chr3	55896690	55896745	[redacted]	-0.393391	[redacted]
chr4	111190700	111190772	[redacted]	-0.39337	[redacted]
chr13	100528767	100528855	[redacted]	-0.39331	[redacted]
chr13	105316054	105316122	[redacted]	-0.393304	[redacted]

chr1	133991361	133991487	[redacted]	-0.393287	[redacted]
chr6	4973061	4973166	[redacted]	-0.393225	[redacted]
chr1	47301681	47301765	[redacted]	-0.393173	[redacted]
chr8	39021223	39021244	[redacted]	-0.393151	[redacted]
chr12	84692990	84693072	[redacted]	-0.393049	[redacted]
chr13	91187436	91187514	[redacted]	-0.392958	[redacted]
chr6	82749432	82749477	[redacted]	-0.392944	[redacted]
chr9	65038243	65038307	[redacted]	-0.392936	[redacted]
chr16	63335115	63335138	[redacted]	-0.392901	[redacted]
chr14	104271640	104271688	[redacted]	-0.39287	[redacted]
chr3	37668104	37668176	[redacted]	-0.392868	[redacted]
chr1	138319979	138320049	[redacted]	-0.392853	[redacted]
chr8	10808126	10808192	[redacted]	-0.392775	[redacted]
chr14	46248271	46248299	[redacted]	-0.392748	[redacted]
chr8	90481835	90481891	[redacted]	-0.392747	[redacted]
chr1	134150214	134150283	[redacted]	-0.392697	[redacted]
chr12	117566521	117566570	[redacted]	-0.392697	[redacted]
chrX	142197121	142197156	[redacted]	-0.392695	[redacted]
chr2	40909894	40909965	[redacted]	-0.392683	[redacted]
chr15	59317722	59317757	[redacted]	-0.392671	[redacted]
chr5	91743779	91743810	[redacted]	-0.392667	[redacted]
chr12	31133363	31133399	[redacted]	-0.392638	[redacted]
chr5	93120698	93120753	[redacted]	-0.392587	[redacted]
chr12	34023960	34024012	[redacted]	-0.392566	[redacted]
chr15	36119788	36119879	[redacted]	-0.39252	[redacted]
chr4	137919030	137919151	[redacted]	-0.392473	[redacted]
chr6	51548379	51548454	[redacted]	-0.392445	[redacted]
chr15	95722376	95722446	[redacted]	-0.39235	[redacted]
chr4	14555187	14555255	[redacted]	-0.392342	[redacted]
chr4	109492263	109492298	[redacted]	-0.392296	[redacted]
chr12	11268529	11268599	[redacted]	-0.392268	[redacted]
chr19	42317025	42317150	[redacted]	-0.392263	[redacted]
chr1_G	189952	189967	[redacted]	-0.392239	[redacted]
chr4	132030167	132030183	[redacted]	-0.392229	[redacted]
chr15	57717556	57717608	[redacted]	-0.392192	[redacted]
chr14	57100293	57100411	[redacted]	-0.392187	[redacted]
chr15	55382855	55382953	[redacted]	-0.392162	[redacted]
chr2	157501400	157501416	[redacted]	-0.39216	[redacted]
chr9	47534513	47534526	[redacted]	-0.392123	[redacted]
chr15	93452489	93452559	[redacted]	-0.392096	[redacted]
chr9	24222257	24222319	[redacted]	-0.391975	[redacted]
chr4	24634451	24634497	[redacted]	-0.391954	[redacted]
chr11	5397503	5397533	[redacted]	-0.391946	[redacted]

chrX	142330021	142330047	[redacted]	-0.391941	[redacted]
chr18	80523458	80523506	[redacted]	-0.391903	[redacted]
chr14	52357350	52357391	[redacted]	-0.391901	[redacted]
chr11	103937742	103937765	[redacted]	-0.39188	[redacted]
chr6	133606728	133606776	[redacted]	-0.391865	[redacted]
chr11	108914367	108914438	[redacted]	-0.391846	[redacted]
chr15	98068069	98068126	[redacted]	-0.391846	[redacted]
chr3	142758004	142758066	[redacted]	-0.391827	[redacted]
chr6	51553286	51553360	[redacted]	-0.391822	[redacted]
chr11	68162597	68162611	[redacted]	-0.391718	[redacted]
chr13	104154372	104154384	[redacted]	-0.39167	[redacted]
chr13	52453637	52453697	[redacted]	-0.391669	[redacted]
chr9	117750452	117750515	[redacted]	-0.391636	[redacted]
chr14	31747723	31747738	[redacted]	-0.391607	[redacted]
chr9	98901397	98901476	[redacted]	-0.391594	[redacted]
chr4	98944998	98945067	[redacted]	-0.391551	[redacted]
chr14	14023200	14023265	[redacted]	-0.391539	[redacted]
chr13	106292809	106292891	[redacted]	-0.391521	[redacted]
chr17	26050314	26050346	[redacted]	-0.39149	[redacted]
chr6	85682956	85683000	[redacted]	-0.391485	[redacted]
chr1	142336857	142336956	[redacted]	-0.39141	[redacted]
chr6	68316246	68316290	[redacted]	-0.391401	[redacted]
chr1	38081536	38081608	[redacted]	-0.39139	[redacted]
chr10	85507479	85507492	[redacted]	-0.391379	[redacted]
chr1	184110343	184110364	[redacted]	-0.391376	[redacted]
chr19	53214553	53214695	[redacted]	-0.391369	[redacted]
chr10	68972504	68972575	[redacted]	-0.391366	[redacted]
chr10	116169438	116169520	[redacted]	-0.391326	[redacted]
chr14	87438249	87438286	[redacted]	-0.391303	[redacted]
chr9	24983406	24983456	[redacted]	-0.391284	[redacted]
chr13	59424833	59424894	[redacted]	-0.391272	[redacted]
chr15	61962073	61962115	[redacted]	-0.391216	[redacted]
chr1	175739933	175739988	[redacted]	-0.391209	[redacted]
chr13	50956103	50956151	[redacted]	-0.391205	[redacted]
chr8	110855507	110855543	[redacted]	-0.391185	[redacted]
chr3	84977773	84977848	[redacted]	-0.391172	[redacted]
chr6	16974490	16974560	[redacted]	-0.391168	[redacted]
chr11	69043220	69043273	[redacted]	-0.39116	[redacted]
chr11	82858306	82858353	[redacted]	-0.391141	[redacted]
chr2	43739608	43739648	[redacted]	-0.391069	[redacted]
chr14	10533525	10533543	[redacted]	-0.390963	[redacted]
chr2	118750998	118751020	[redacted]	-0.390949	[redacted]
chr5	121914395	121914456	[redacted]	-0.390905	[redacted]

chr1	43540841	43540914	[redacted]	-0.390884	[redacted]
chr7	17051633	17051687	[redacted]	-0.390848	[redacted]
chr3	53515812	53515863	[redacted]	-0.390838	[redacted]
chr3	72303271	72303362	[redacted]	-0.390837	[redacted]
chr12	4113848	4113893	[redacted]	-0.390702	[redacted]
chr19	55386834	55386899	[redacted]	-0.390645	[redacted]
chr8	114725397	114725469	[redacted]	-0.390626	[redacted]
chr7	24281843	24281897	[redacted]	-0.39062	[redacted]
chr9	49637243	49637324	[redacted]	-0.390612	[redacted]
chr11	52271694	52271767	[redacted]	-0.390543	[redacted]
chr5	21789356	21789419	[redacted]	-0.390539	[redacted]
chr17	81073140	81073174	[redacted]	-0.390536	[redacted]
chr10	119270207	119270312	[redacted]	-0.390518	[redacted]
chr16	23196160	23196196	[redacted]	-0.390495	[redacted]
chr2	63670402	63670482	[redacted]	-0.390463	[redacted]
chr10	117339190	117339215	[redacted]	-0.39045	[redacted]
chr9	64272591	64272622	[redacted]	-0.390448	[redacted]
chr7	90497742	90497793	[redacted]	-0.390387	[redacted]
chr16	50783370	50783387	[redacted]	-0.390375	[redacted]
chr7	16494833	16494894	[redacted]	-0.390348	[redacted]
chr14	59072962	59073003	[redacted]	-0.390329	[redacted]
chr2	120922977	120923032	[redacted]	-0.390291	[redacted]
chr17	56478619	56478719	[redacted]	-0.390286	[redacted]
chr6	99644262	99644290	[redacted]	-0.390237	[redacted]
chr4	59044040	59044075	[redacted]	-0.390229	[redacted]
chr11	110766894	110766927	[redacted]	-0.390226	[redacted]
chr10	91080988	91081031	[redacted]	-0.390201	[redacted]
chr6	126416536	126416627	[redacted]	-0.390176	[redacted]
chr17	53591556	53591589	[redacted]	-0.390164	[redacted]
chr12	118619827	118619850	[redacted]	-0.390153	[redacted]
chr8	77428180	77428219	[redacted]	-0.390126	[redacted]
chr5	123638056	123638113	[redacted]	-0.39012	[redacted]
chr17	24291223	24291258	[redacted]	-0.390097	[redacted]
chr19	27469724	27469767	[redacted]	-0.390094	[redacted]
chr8	108911499	108911524	[redacted]	-0.390093	[redacted]
chr6	72645146	72645226	[redacted]	-0.390032	[redacted]
chr12	71573604	71573640	[redacted]	-0.390021	[redacted]
chr7	31532015	31532095	[redacted]	-0.389981	[redacted]
chr5	65824138	65824212	[redacted]	-0.389964	[redacted]
chr9	49727637	49727696	[redacted]	-0.38996	[redacted]
chr5	100654496	100654519	[redacted]	-0.38993	[redacted]
chr11	90425155	90425198	[redacted]	-0.389913	[redacted]
chr5	118581795	118581860	[redacted]	-0.389904	[redacted]

chr12	80310853	80310894	[redacted]	-0.389887	[redacted]
chr5	142747275	142747290	[redacted]	-0.389887	[redacted]
chr18	13026975	13027039	[redacted]	-0.389882	[redacted]
chr13	107811950	107811971	[redacted]	-0.389865	[redacted]
chr5	148224756	148224784	[redacted]	-0.389865	[redacted]
chr16	35759149	35759223	[redacted]	-0.389835	[redacted]
chr10	25368564	25368586	[redacted]	-0.389826	[redacted]
chr4	35137236	35137289	[redacted]	-0.389813	[redacted]
chr7	49367365	49367421	[redacted]	-0.389806	[redacted]
chr5	24780713	24780783	[redacted]	-0.3898	[redacted]
chr2	52313103	52313169	[redacted]	-0.389767	[redacted]
chr5	101985950	101986029	[redacted]	-0.389766	[redacted]
chr14	121253711	121253763	[redacted]	-0.389756	[redacted]
chr6	54106374	54106422	[redacted]	-0.389703	[redacted]
chr11	30688992	30689057	[redacted]	-0.389701	[redacted]
chr19	56815761	56815841	[redacted]	-0.38966	[redacted]
chr5	135394934	135394995	[redacted]	-0.389629	[redacted]
chr17	72539689	72539745	[redacted]	-0.389611	[redacted]
chr10	20861650	20861726	[redacted]	-0.389591	[redacted]
chr12	86559252	86559290	[redacted]	-0.389584	[redacted]
chr16	52640498	52640571	[redacted]	-0.389565	[redacted]
chr12	20953202	20953233	[redacted]	-0.389528	[redacted]
chr10	21127723	21127831	[redacted]	-0.389516	[redacted]
chr15	92001836	92001909	[redacted]	-0.389446	[redacted]
chr2	157509665	157509681	[redacted]	-0.389436	[redacted]
chr11	36093072	36093114	[redacted]	-0.389409	[redacted]
chr1	151585523	151585578	[redacted]	-0.389403	[redacted]
chr11	66151154	66151229	[redacted]	-0.389363	[redacted]
chr11	113906151	113906194	[redacted]	-0.389325	[redacted]
chr14	118395246	118395255	[redacted]	-0.389301	[redacted]
chr5	52257919	52257940	[redacted]	-0.389269	[redacted]
chr11	33495387	33495540	[redacted]	-0.389256	[redacted]
chr13	97217574	97217648	[redacted]	-0.389229	[redacted]
chr11	17927906	17927964	[redacted]	-0.389215	[redacted]
chr5	135032441	135032466	[redacted]	-0.389208	[redacted]
chr5	3973889	3973960	[redacted]	-0.389199	[redacted]
chr7	37711867	37711948	[redacted]	-0.389145	[redacted]
chr8	94688353	94688445	[redacted]	-0.389126	[redacted]
chr13	38632576	38632637	[redacted]	-0.389123	[redacted]
chr6	103335541	103335660	[redacted]	-0.389095	[redacted]
chr13	57929439	57929480	[redacted]	-0.389082	[redacted]
chr11	116884395	116884442	[redacted]	-0.389067	[redacted]
chr4	128595029	128595068	[redacted]	-0.38904	[redacted]

chr9	61130224	61130279	[redacted]	-0.389014	[redacted]
chr19	54113611	54113662	[redacted]	-0.388995	[redacted]
chr6	85036350	85036394	[redacted]	-0.388977	[redacted]
chr4	92148982	92149027	[redacted]	-0.388971	[redacted]
chr6	108149430	108149475	[redacted]	-0.388937	[redacted]
chr10	120746787	120746852	[redacted]	-0.38893	[redacted]
chr7	79923239	79923381	[redacted]	-0.388914	[redacted]
chr7	48906097	48906123	[redacted]	-0.3889	[redacted]
chr14	62404499	62404550	[redacted]	-0.388844	[redacted]
chr9	107023199	107023207	[redacted]	-0.388843	[redacted]
chr14	117342065	117342149	[redacted]	-0.38884	[redacted]
chr5	148319785	148319848	[redacted]	-0.388831	[redacted]
chr12	28301917	28301963	[redacted]	-0.388829	[redacted]
chr13	52862037	52862091	[redacted]	-0.388793	[redacted]
chr14	122055708	122055742	[redacted]	-0.388749	[redacted]
chr5	98004294	98004311	[redacted]	-0.388715	[redacted]
chr9	50547479	50547543	[redacted]	-0.388646	[redacted]
chr5	21619375	21619417	[redacted]	-0.388633	[redacted]
chr14	55689466	55689535	[redacted]	-0.388627	[redacted]
chr5	148677603	148677636	[redacted]	-0.388626	[redacted]
chr18	50501757	50501839	[redacted]	-0.388554	[redacted]
chr11	96133259	96133327	[redacted]	-0.388546	[redacted]
chr8	54323988	54324069	[redacted]	-0.388486	[redacted]
chr18	63666651	63666712	[redacted]	-0.388474	[redacted]
chr4	40547517	40547569	[redacted]	-0.388395	[redacted]
chr1	99228197	99228274	[redacted]	-0.388371	[redacted]
chr7	122507033	122507087	[redacted]	-0.388348	[redacted]
chr12	80912100	80912182	[redacted]	-0.388326	[redacted]
chr14	102136933	102136998	[redacted]	-0.388317	[redacted]
chr13	19385683	19385745	[redacted]	-0.3883	[redacted]
chr7	43315956	43316011	[redacted]	-0.388286	[redacted]
chr9	58183601	58183685	[redacted]	-0.38828	[redacted]
chr15	81044232	81044283	[redacted]	-0.388277	[redacted]
chr6	99823680	99823761	[redacted]	-0.388264	[redacted]
chr13	23813440	23813498	[redacted]	-0.388261	[redacted]
chr15	5148697	5148764	[redacted]	-0.388261	[redacted]
chr1	157432841	157432898	[redacted]	-0.388221	[redacted]
chr16	31913070	31913138	[redacted]	-0.388193	[redacted]
chr7	93007787	93007817	[redacted]	-0.388155	[redacted]
chr13	117263223	117263248	[redacted]	-0.388136	[redacted]
chr14	77696324	77696415	[redacted]	-0.388064	[redacted]
chrX	72763784	72763842	[redacted]	-0.38806	[redacted]
chr13	9322221	9322276	[redacted]	-0.38801	[redacted]

chr12	73806459	73806525	[redacted]	-0.388005	[redacted]
chr9	42585736	42585789	[redacted]	-0.387954	[redacted]
chr19	28523442	28523571	[redacted]	-0.387892	[redacted]
chr3	62221186	62221277	[redacted]	-0.387892	[redacted]
chr7	46178989	46179050	[redacted]	-0.38789	[redacted]
chr9	85717589	85717642	[redacted]	-0.387798	[redacted]
chr9	121851388	121851446	[redacted]	-0.387795	[redacted]
chr16	16467181	16467231	[redacted]	-0.387773	[redacted]
chr3	8882147	8882200	[redacted]	-0.387748	[redacted]
chr10	40295435	40295566	[redacted]	-0.387725	[redacted]
chr5	21316883	21316988	[redacted]	-0.387683	[redacted]
chr15	27925326	27925403	[redacted]	-0.387656	[redacted]
chr15	50791285	50791338	[redacted]	-0.387646	[redacted]
chr12	55292785	55292797	[redacted]	-0.387608	[redacted]
chr19	41770303	41770354	[redacted]	-0.38759	[redacted]
chr7	33818143	33818161	[redacted]	-0.387582	[redacted]
chr7	100326797	100326853	[redacted]	-0.387574	[redacted]
chr4	133153479	133153544	[redacted]	-0.387564	[redacted]
chr4	102343076	102343150	[redacted]	-0.387552	[redacted]
chr16	70466759	70466822	[redacted]	-0.387533	[redacted]
chr2	143884941	143884987	[redacted]	-0.387518	[redacted]
chr14	56165848	56165887	[redacted]	-0.387502	[redacted]
chr19	46553790	46553855	[redacted]	-0.387495	[redacted]
chr11	4245458	4245526	[redacted]	-0.387468	[redacted]
chr8	60832047	60832122	[redacted]	-0.387462	[redacted]
chr2	133380269	133380309	[redacted]	-0.387383	[redacted]
chr10	20522089	20522133	[redacted]	-0.387375	[redacted]
chr5	47212716	47212776	[redacted]	-0.387375	[redacted]
chr3	84977922	84978007	[redacted]	-0.387374	[redacted]
chr16	91918739	91918764	[redacted]	-0.387312	[redacted]
chr17	76382381	76382460	[redacted]	-0.387274	[redacted]
chr1	75751615	75751674	[redacted]	-0.387273	[redacted]
chr2	151123001	151123060	[redacted]	-0.38726	[redacted]
chr2	125903897	125903960	[redacted]	-0.387254	[redacted]
chr14	121957682	121957712	[redacted]	-0.38725	[redacted]
chr1	190044818	190044854	[redacted]	-0.387246	[redacted]
chr7	34424401	34424477	[redacted]	-0.387223	[redacted]
chr13	112289822	112289912	[redacted]	-0.387215	[redacted]
chr1	95587923	95588012	[redacted]	-0.387214	[redacted]
chr4	111654631	111654672	[redacted]	-0.387209	[redacted]
chr19	36507064	36507090	[redacted]	-0.38718	[redacted]
chr3	75862947	75862992	[redacted]	-0.387176	[redacted]
chr13	100824758	100824862	[redacted]	-0.387133	[redacted]

chr4	141544060	141544121	[redacted]	-0.387123	[redacted]
chr9	15718449	15718497	[redacted]	-0.3871	[redacted]
chr16	90929836	90929870	[redacted]	-0.387099	[redacted]
chr9	44451970	44452026	[redacted]	-0.387061	[redacted]
chr17	33836476	33836533	[redacted]	-0.387028	[redacted]
chr10	44446649	44446694	[redacted]	-0.386991	[redacted]
chr5	58033633	58033668	[redacted]	-0.386987	[redacted]
chr7	140280728	140280776	[redacted]	-0.386984	[redacted]
chr7	122358937	122358985	[redacted]	-0.386938	[redacted]
chr9	67904582	67904661	[redacted]	-0.3869	[redacted]
chr15	43974379	43974452	[redacted]	-0.386899	[redacted]
chr4	34891188	34891213	[redacted]	-0.386896	[redacted]
chr5	124995862	124995909	[redacted]	-0.386895	[redacted]
chr4	32266352	32266417	[redacted]	-0.386895	[redacted]
chr4	98245621	98245673	[redacted]	-0.386868	[redacted]
chr7	68287351	68287363	[redacted]	-0.386818	[redacted]
chr11	50287228	50287274	[redacted]	-0.38675	[redacted]
chr4	47996670	47996719	[redacted]	-0.386748	[redacted]
chr19	44874749	44874795	[redacted]	-0.386726	[redacted]
chr2	117308074	117308144	[redacted]	-0.386723	[redacted]
chr13	110534308	110534373	[redacted]	-0.386714	[redacted]
chr6	140441196	140441251	[redacted]	-0.386713	[redacted]
chr8	117261257	117261414	[redacted]	-0.386693	[redacted]
chr16	38462023	38462058	[redacted]	-0.386678	[redacted]
chr16	84230602	84230657	[redacted]	-0.386655	[redacted]
chrX	134730243	134730291	[redacted]	-0.386655	[redacted]
chr17	30750857	30750902	[redacted]	-0.386652	[redacted]
chr10	91358576	91358625	[redacted]	-0.386585	[redacted]
chr14	120083188	120083247	[redacted]	-0.386578	[redacted]
chr14	34023376	34023451	[redacted]	-0.386562	[redacted]
chr8	102685240	102685284	[redacted]	-0.386549	[redacted]
chr2	128899497	128899522	[redacted]	-0.386544	[redacted]
chr8	18827596	18827669	[redacted]	-0.386542	[redacted]
chr2	159193587	159193640	[redacted]	-0.386496	[redacted]
chr16	36572070	36572105	[redacted]	-0.386377	[redacted]
chr3	37134096	37134162	[redacted]	-0.386372	[redacted]
chr16	43622164	43622229	[redacted]	-0.386371	[redacted]
chr12	3931892	3931936	[redacted]	-0.386365	[redacted]
chr2	108049193	108049224	[redacted]	-0.386356	[redacted]
chr11	74685387	74685449	[redacted]	-0.386326	[redacted]
chr17	86594302	86594392	[redacted]	-0.386314	[redacted]
chr6	29515973	29515993	[redacted]	-0.38628	[redacted]
chr6	41000103	41000149	[redacted]	-0.386275	[redacted]

chr18	49777374	49777400	[redacted]	-0.38623	[redacted]
chr17	12499437	12499505	[redacted]	-0.386144	[redacted]
chr14	28226622	28226699	[redacted]	-0.386125	[redacted]
chr15	14212444	14212525	[redacted]	-0.386117	[redacted]
chr2	161076236	161076279	[redacted]	-0.386108	[redacted]
chr9	117964399	117964466	[redacted]	-0.386089	[redacted]
chr18	80169906	80169944	[redacted]	-0.386077	[redacted]
chr4	149928441	149928542	[redacted]	-0.38607	[redacted]
chr3	19627449	19627498	[redacted]	-0.386014	[redacted]
chr1	33297874	33297939	[redacted]	-0.38599	[redacted]
chr4	62256355	62256424	[redacted]	-0.385978	[redacted]
chr3	75968724	75968812	[redacted]	-0.385964	[redacted]
chr11	6085052	6085105	[redacted]	-0.385936	[redacted]
chr16	38048717	38048767	[redacted]	-0.385923	[redacted]
chr15	30485182	30485275	[redacted]	-0.385882	[redacted]
chr11	93997889	93997972	[redacted]	-0.385878	[redacted]
chr4	32389594	32389600	[redacted]	-0.385872	[redacted]
chr2	92917155	92917204	[redacted]	-0.385867	[redacted]
chr3	32034567	32034630	[redacted]	-0.385864	[redacted]
chr18	34027044	34027074	[redacted]	-0.385851	[redacted]
chr16	51877308	51877368	[redacted]	-0.385826	[redacted]
chr1	165869904	165869984	[redacted]	-0.385818	[redacted]
chr8	46181691	46181700	[redacted]	-0.385816	[redacted]
chr12	46808013	46808064	[redacted]	-0.385782	[redacted]
chr4	24880152	24880249	[redacted]	-0.385775	[redacted]
chr5	56587765	56587821	[redacted]	-0.385757	[redacted]
chr14	74357150	74357210	[redacted]	-0.385752	[redacted]
chr15	47567174	47567200	[redacted]	-0.385746	[redacted]
chr8	10165417	10165493	[redacted]	-0.385736	[redacted]
chr1	38607331	38607362	[redacted]	-0.385706	[redacted]
chr12	73372664	73372725	[redacted]	-0.385706	[redacted]
chr9	97737431	97737481	[redacted]	-0.385618	[redacted]
chr2	103364242	103364280	[redacted]	-0.385611	[redacted]
chr8	18570564	18570610	[redacted]	-0.385598	[redacted]
chr4	135509035	135509099	[redacted]	-0.385582	[redacted]
chr14	110130206	110130252	[redacted]	-0.385565	[redacted]
chr2	158853401	158853497	[redacted]	-0.385533	[redacted]
chr8	114668141	114668258	[redacted]	-0.385512	[redacted]
chr11	87953192	87953267	[redacted]	-0.385497	[redacted]
chr1	175629574	175629641	[redacted]	-0.38548	[redacted]
chr17	82237981	82238017	[redacted]	-0.385457	[redacted]
chr9	69895055	69895070	[redacted]	-0.38545	[redacted]
chr13	47771304	47771333	[redacted]	-0.385445	[redacted]

chr9	112177071	112177140	[redacted]	-0.38544	[redacted]
chr12	52961955	52961978	[redacted]	-0.385375	[redacted]
chr12	53038888	53038958	[redacted]	-0.385276	[redacted]
chr2	96554009	96554096	[redacted]	-0.385269	[redacted]
chr5	90335602	90335656	[redacted]	-0.385251	[redacted]
chr8	5033622	5033655	[redacted]	-0.385237	[redacted]
chrX	163440968	163441047	[redacted]	-0.385216	[redacted]
chr5	110753715	110753873	[redacted]	-0.385211	[redacted]
chr2	132349607	132349661	[redacted]	-0.385154	[redacted]
chr17	66723744	66723767	[redacted]	-0.385151	[redacted]
chr6	118584957	118585029	[redacted]	-0.385143	[redacted]
chr16	55982326	55982385	[redacted]	-0.385119	[redacted]
chr14	47663772	47663807	[redacted]	-0.385052	[redacted]
chr6	116284853	116284905	[redacted]	-0.38505	[redacted]
chr11	101970254	101970303	[redacted]	-0.385037	[redacted]
chr18	14082152	14082215	[redacted]	-0.385034	[redacted]
chr18	3513239	3513286	[redacted]	-0.384997	[redacted]
chr19	30515970	30516033	[redacted]	-0.384993	[redacted]
chr8	78704165	78704238	[redacted]	-0.384969	[redacted]
chr14	120136366	120136446	[redacted]	-0.384875	[redacted]
chrX	48193366	48193422	[redacted]	-0.384868	[redacted]
chr10	44170019	44170050	[redacted]	-0.384754	[redacted]
chr15	51850970	51851060	[redacted]	-0.384754	[redacted]
chr14	21498207	21498264	[redacted]	-0.384704	[redacted]
chr5	66136381	66136406	[redacted]	-0.38467	[redacted]
chr19	39609228	39609301	[redacted]	-0.384646	[redacted]
chr19	55256233	55256264	[redacted]	-0.384632	[redacted]
chr5	145581434	145581508	[redacted]	-0.384631	[redacted]
chr6	143288915	143288965	[redacted]	-0.384608	[redacted]
chr17	79037319	79037391	[redacted]	-0.384593	[redacted]
chr11	35978874	35978940	[redacted]	-0.384586	[redacted]
chr16	49797448	49797460	[redacted]	-0.384567	[redacted]
chr2	58625371	58625421	[redacted]	-0.384559	[redacted]
chr4	63138655	63138735	[redacted]	-0.384536	[redacted]
chr17	85318341	85318373	[redacted]	-0.384497	[redacted]
chr6	12078073	12078128	[redacted]	-0.384486	[redacted]
chr10	118399662	118399749	[redacted]	-0.384439	[redacted]
chr9	24653169	24653233	[redacted]	-0.384347	[redacted]
chr9	90395393	90395473	[redacted]	-0.384347	[redacted]
chr1	184895988	184896106	[redacted]	-0.384316	[redacted]
chr12	85000089	85000172	[redacted]	-0.384297	[redacted]
chr5	57791958	57792018	[redacted]	-0.38427	[redacted]
chr14	73312558	73312627	[redacted]	-0.384266	[redacted]

chr3	146072751	146072769	[redacted]	-0.384261	[redacted]
chr12	36235353	36235414	[redacted]	-0.384252	[redacted]
chr9	57564774	57564818	[redacted]	-0.38422	[redacted]
chr19	34959486	34959546	[redacted]	-0.384209	[redacted]
chr19	19551442	19551538	[redacted]	-0.384195	[redacted]
chr13	55696401	55696442	[redacted]	-0.384148	[redacted]
chr8	123223904	123223921	[redacted]	-0.384141	[redacted]
chr9	66525569	66525614	[redacted]	-0.384096	[redacted]
chr17	5454337	5454356	[redacted]	-0.384083	[redacted]
chr15	6388585	6388641	[redacted]	-0.384039	[redacted]
chr15	84868563	84868576	[redacted]	-0.384034	[redacted]
chr16	75525237	75525303	[redacted]	-0.383994	[redacted]
chr12	108533196	108533245	[redacted]	-0.383994	[redacted]
chr2	69654136	69654153	[redacted]	-0.383957	[redacted]
chr3	107885865	107885913	[redacted]	-0.383922	[redacted]
chr15	75965642	75965693	[redacted]	-0.383918	[redacted]
chr13	93098142	93098161	[redacted]	-0.383917	[redacted]
chr2	48647492	48647584	[redacted]	-0.383883	[redacted]
chr2	160301368	160301415	[redacted]	-0.383863	[redacted]
chr17	28083969	28083994	[redacted]	-0.383839	[redacted]
chr2	164929592	164929615	[redacted]	-0.383766	[redacted]
chr11	18477485	18477501	[redacted]	-0.383751	[redacted]
chr5	103358507	103358578	[redacted]	-0.383722	[redacted]
chr13	117263832	117263889	[redacted]	-0.383665	[redacted]
chr10	120799662	120799711	[redacted]	-0.383655	[redacted]
chr15	52347760	52347787	[redacted]	-0.383654	[redacted]
chrX	138396137	138396173	[redacted]	-0.383627	[redacted]
chr14	100279311	100279366	[redacted]	-0.383606	[redacted]
chr14	17489163	17489214	[redacted]	-0.383595	[redacted]
chr11	69719948	69719992	[redacted]	-0.383567	[redacted]
chr14	113429196	113429291	[redacted]	-0.383542	[redacted]
chr17	72966722	72966756	[redacted]	-0.383527	[redacted]
chr7	64390763	64390865	[redacted]	-0.383508	[redacted]
chr19	41896739	41896769	[redacted]	-0.383472	[redacted]
chr11	119865818	119865859	[redacted]	-0.38347	[redacted]
chr17	31204751	31204801	[redacted]	-0.383464	[redacted]
chr1	192313615	192313648	[redacted]	-0.383452	[redacted]
chr10	94500386	94500425	[redacted]	-0.383416	[redacted]
chr3	133877847	133877872	[redacted]	-0.383362	[redacted]
chr16	44402638	44402655	[redacted]	-0.383337	[redacted]
chr5	147814918	147815092	[redacted]	-0.383334	[redacted]
chr4	148991068	148991108	[redacted]	-0.383308	[redacted]
chr5	65666672	65666701	[redacted]	-0.383307	[redacted]

chr9	122735248	122735285	[redacted]	-0.383278	[redacted]
chr1	90098852	90098922	[redacted]	-0.383261	[redacted]
chr10	10370647	10370688	[redacted]	-0.383241	[redacted]
chr12	10724547	10724573	[redacted]	-0.383224	[redacted]
chr1	62375778	62375840	[redacted]	-0.383168	[redacted]
chr14	78804247	78804317	[redacted]	-0.383167	[redacted]
chr18	62049992	62050002	[redacted]	-0.38316	[redacted]
chr15	70386355	70386428	[redacted]	-0.383141	[redacted]
chr7	145202145	145202207	[redacted]	-0.383139	[redacted]
chr4	117013760	117013850	[redacted]	-0.383132	[redacted]
chr16	70317487	70317536	[redacted]	-0.383103	[redacted]
chr18	37314341	37314352	[redacted]	-0.383083	[redacted]
chr6	39161755	39161816	[redacted]	-0.383064	[redacted]
chr6	103329839	103329909	[redacted]	-0.383042	[redacted]
chr4	74258088	74258140	[redacted]	-0.383017	[redacted]
chr10	42877208	42877268	[redacted]	-0.382956	[redacted]
chr3	60562563	60562613	[redacted]	-0.382956	[redacted]
chr11	114145008	114145039	[redacted]	-0.382931	[redacted]
chr18	34794516	34794536	[redacted]	-0.382892	[redacted]
chr6	146837526	146837555	[redacted]	-0.382885	[redacted]
chr9	101425357	101425401	[redacted]	-0.382844	[redacted]
chr2	121915407	121915467	[redacted]	-0.382823	[redacted]
chr4	136083845	136083935	[redacted]	-0.382798	[redacted]
chr1	14650714	14650778	[redacted]	-0.382781	[redacted]
chr14	91072673	91072684	[redacted]	-0.382776	[redacted]
chr19	58548894	58548935	[redacted]	-0.382755	[redacted]
chr7	43155342	43155395	[redacted]	-0.38273	[redacted]
chr6	144117350	144117409	[redacted]	-0.382724	[redacted]
chr8	25651835	25651929	[redacted]	-0.382719	[redacted]
chr9	49815621	49815643	[redacted]	-0.382698	[redacted]
chr10	40347957	40347984	[redacted]	-0.382687	[redacted]
chr1	62353475	62353534	[redacted]	-0.382685	[redacted]
chr3	60639409	60639442	[redacted]	-0.382647	[redacted]
chr15	98874594	98874649	[redacted]	-0.38263	[redacted]
chr17	31262953	31263002	[redacted]	-0.382628	[redacted]
chr13	20279380	20279452	[redacted]	-0.382624	[redacted]
chr19	28640324	28640443	[redacted]	-0.382621	[redacted]
chr13	31104713	31104741	[redacted]	-0.382608	[redacted]
chr13	23522154	23522232	[redacted]	-0.382599	[redacted]
chr6	87771721	87771788	[redacted]	-0.382582	[redacted]
chr8	54059024	54059077	[redacted]	-0.382577	[redacted]
chr7	81056051	81056135	[redacted]	-0.382575	[redacted]
chr6	124918507	124918588	[redacted]	-0.382553	[redacted]

chr17	79996819	79996870	[redacted]	-0.382527	[redacted]
chr17	12434060	12434137	[redacted]	-0.382498	[redacted]
chr11	102551051	102551081	[redacted]	-0.382485	[redacted]
chr19	36088874	36088950	[redacted]	-0.382469	[redacted]
chr1	36687313	36687371	[redacted]	-0.382451	[redacted]
chr4	137714660	137714674	[redacted]	-0.382411	[redacted]
chr8	103169747	103169783	[redacted]	-0.382349	[redacted]
chr14	52174033	52174124	[redacted]	-0.382342	[redacted]
chr4	132631138	132631194	[redacted]	-0.382333	[redacted]
chr2	60779848	60780014	[redacted]	-0.382306	[redacted]
chr10	77817608	77817662	[redacted]	-0.382303	[redacted]
chr17	86900220	86900277	[redacted]	-0.382295	[redacted]
chr7	61437694	61437775	[redacted]	-0.382283	[redacted]
chr11	78174867	78174925	[redacted]	-0.382281	[redacted]
chr2	145604106	145604131	[redacted]	-0.382236	[redacted]
chr2	180278896	180278968	[redacted]	-0.382233	[redacted]
chr5	147749061	147749109	[redacted]	-0.382181	[redacted]
chr13	96431666	96431676	[redacted]	-0.382165	[redacted]
chr14	29641116	29641166	[redacted]	-0.382164	[redacted]
chr11	76827154	76827202	[redacted]	-0.38215	[redacted]
chr14	27563228	27563272	[redacted]	-0.382149	[redacted]
chr19	19097701	19097744	[redacted]	-0.382139	[redacted]
chr9	45057397	45057455	[redacted]	-0.382137	[redacted]
chr5	64004876	64004954	[redacted]	-0.382131	[redacted]
chr9	22400102	22400133	[redacted]	-0.38209	[redacted]
chr11	83266444	83266505	[redacted]	-0.382087	[redacted]
chr17	66066484	66066508	[redacted]	-0.382049	[redacted]
chr3	89122835	89122922	[redacted]	-0.382031	[redacted]
chr5	150197618	150197662	[redacted]	-0.382023	[redacted]
chr7	82075162	82075198	[redacted]	-0.382002	[redacted]
chr15	87156575	87156629	[redacted]	-0.381981	[redacted]
chr1	88264689	88264757	[redacted]	-0.3819	[redacted]
chr2	3887754	3887815	[redacted]	-0.381898	[redacted]
chr15	42972159	42972226	[redacted]	-0.381863	[redacted]
chr4	5338151	5338204	[redacted]	-0.381846	[redacted]
chr7	27543266	27543320	[redacted]	-0.381835	[redacted]
chr5	53602391	53602428	[redacted]	-0.381811	[redacted]
chr19	29524020	29524130	[redacted]	-0.38181	[redacted]
chr5	143624570	143624658	[redacted]	-0.381808	[redacted]
chr15	43864746	43864799	[redacted]	-0.381804	[redacted]
chr16	58699937	58699987	[redacted]	-0.381746	[redacted]
chr11	95665672	95665681	[redacted]	-0.38174	[redacted]
chr1	38500278	38500344	[redacted]	-0.38173	[redacted]

chr3	64351775	64351849	[redacted]	-0.381697	[redacted]
chr17	48055410	48055453	[redacted]	-0.381696	[redacted]
chr11	4453763	4453822	[redacted]	-0.381656	[redacted]
chr13	44356595	44356667	[redacted]	-0.381631	[redacted]
chr8	13611879	13612002	[redacted]	-0.381589	[redacted]
chr2	117221827	117221854	[redacted]	-0.381542	[redacted]
chr10	115387766	115387790	[redacted]	-0.38152	[redacted]
chr4	130318876	130318929	[redacted]	-0.381502	[redacted]
chr11	67917026	67917075	[redacted]	-0.381475	[redacted]
chr11	111563215	111563305	[redacted]	-0.381472	[redacted]
chr10	77254839	77254906	[redacted]	-0.381458	[redacted]
chr1	175887818	175887902	[redacted]	-0.381445	[redacted]
chr11	82038109	82038167	[redacted]	-0.381438	[redacted]
chr1	155122198	155122253	[redacted]	-0.381411	[redacted]
chr3	36502876	36502922	[redacted]	-0.381409	[redacted]
chr16	18769024	18769065	[redacted]	-0.381391	[redacted]
chr15	99302726	99302792	[redacted]	-0.381381	[redacted]
chr7	137770392	137770529	[redacted]	-0.38136	[redacted]
chr16	29891041	29891076	[redacted]	-0.381346	[redacted]
chr8	22166792	22166854	[redacted]	-0.381315	[redacted]
chr11	82469863	82469901	[redacted]	-0.381303	[redacted]
chr2	6375704	6375784	[redacted]	-0.381303	[redacted]
chr5	37490880	37490915	[redacted]	-0.381285	[redacted]
chr1	190080757	190080806	[redacted]	-0.381252	[redacted]
chr9	51307499	51307562	[redacted]	-0.381196	[redacted]
chr13	40374119	40374200	[redacted]	-0.381166	[redacted]
chr17	46932997	46933078	[redacted]	-0.381158	[redacted]
chr5	90404135	90404172	[redacted]	-0.381155	[redacted]
chr10	122832224	122832299	[redacted]	-0.381139	[redacted]
chr1	12964643	12964685	[redacted]	-0.381135	[redacted]
chr3	101817897	101817935	[redacted]	-0.381132	[redacted]
chr3	90245234	90245251	[redacted]	-0.381108	[redacted]
chr18	46332251	46332325	[redacted]	-0.381108	[redacted]
chr16	90818234	90818301	[redacted]	-0.381101	[redacted]
chr19	16626140	16626160	[redacted]	-0.381094	[redacted]
chr8	95452687	95452752	[redacted]	-0.381004	[redacted]
chr2	165812326	165812363	[redacted]	-0.380973	[redacted]
chr16	59537127	59537159	[redacted]	-0.380931	[redacted]
chr11	97289125	97289171	[redacted]	-0.380922	[redacted]
chr3	41453411	41453453	[redacted]	-0.380898	[redacted]
chr1	83995527	83995582	[redacted]	-0.380897	[redacted]
chr2	73001594	73001643	[redacted]	-0.380864	[redacted]
chr6	99221408	99221446	[redacted]	-0.380848	[redacted]

chr12	81566710	81566744	[redacted]	-0.380845	[redacted]
chr5	107103161	107103222	[redacted]	-0.380845	[redacted]
chr9	54527918	54527974	[redacted]	-0.380822	[redacted]
chr3	153816670	153816727	[redacted]	-0.380809	[redacted]
chr6	30046680	30046727	[redacted]	-0.380704	[redacted]
chr9	77801966	77802045	[redacted]	-0.380694	[redacted]
chr2	157656735	157656786	[redacted]	-0.380683	[redacted]
chr9	97926896	97926975	[redacted]	-0.380683	[redacted]
chr2	74409692	74409752	[redacted]	-0.380677	[redacted]
chr1	63842594	63842678	[redacted]	-0.380664	[redacted]
chr9	15398548	15398622	[redacted]	-0.380608	[redacted]
chr16	42617930	42618002	[redacted]	-0.380607	[redacted]
chr13	109515449	109515562	[redacted]	-0.380591	[redacted]
chr19	29557575	29557625	[redacted]	-0.380578	[redacted]
chr5	137480328	137480457	[redacted]	-0.380539	[redacted]
chr11	109127516	109127570	[redacted]	-0.380536	[redacted]
chr2	150192508	150192571	[redacted]	-0.38053	[redacted]
chrX	67752578	67752603	[redacted]	-0.380528	[redacted]
chr1	177150358	177150408	[redacted]	-0.380522	[redacted]
chr5	150082579	150082600	[redacted]	-0.380475	[redacted]
chr4	84767051	84767103	[redacted]	-0.38046	[redacted]
chr14	57753870	57753894	[redacted]	-0.380458	[redacted]
chr13	56723481	56723539	[redacted]	-0.380453	[redacted]
chrX	169979470	169979536	[redacted]	-0.380453	[redacted]
chr2	117344177	117344249	[redacted]	-0.380389	[redacted]
chr17	59211701	59211759	[redacted]	-0.380349	[redacted]
chr5	23416935	23417021	[redacted]	-0.380343	[redacted]
chr10	127255854	127255872	[redacted]	-0.380298	[redacted]
chrX	56553197	56553257	[redacted]	-0.380275	[redacted]
chrX	102548458	102548490	[redacted]	-0.380272	[redacted]
chr18	82647229	82647278	[redacted]	-0.380251	[redacted]
chr8	60638812	60638876	[redacted]	-0.380213	[redacted]
chr7	33255774	33255833	[redacted]	-0.380211	[redacted]
chr6	31278186	31278279	[redacted]	-0.380207	[redacted]
chr9	76069208	76069291	[redacted]	-0.380202	[redacted]
chr2	30614105	30614142	[redacted]	-0.380178	[redacted]
chr12	73724112	73724136	[redacted]	-0.380173	[redacted]
chr1	156844929	156844973	[redacted]	-0.380168	[redacted]
chr15	85937214	85937297	[redacted]	-0.380153	[redacted]
chr16	28558305	28558336	[redacted]	-0.38011	[redacted]
chr4	141688780	141688846	[redacted]	-0.380098	[redacted]
chr6	4015582	4015648	[redacted]	-0.380094	[redacted]
chr3	99713070	99713142	[redacted]	-0.38009	[redacted]

chr9	63331484	63331543	[redacted]	-0.380048	[redacted]
chr13	63276144	63276256	[redacted]	-0.380047	[redacted]
chr15	4272811	4272869	[redacted]	-0.37995	[redacted]
chr8	97684693	97684758	[redacted]	-0.379947	[redacted]
chr1	29571004	29571021	[redacted]	-0.379944	[redacted]
chr10	91982136	91982168	[redacted]	-0.379939	[redacted]
chr19	53389051	53389107	[redacted]	-0.379938	[redacted]
chr5	135596679	135596803	[redacted]	-0.379874	[redacted]
chr9	79329096	79329136	[redacted]	-0.379843	[redacted]
chr15	55285377	55285394	[redacted]	-0.379834	[redacted]
chr15	59537343	59537445	[redacted]	-0.379767	[redacted]
chr18	53236834	53236916	[redacted]	-0.379738	[redacted]
chr14	25828129	25828191	[redacted]	-0.379706	[redacted]
chr1	11743652	11743703	[redacted]	-0.379697	[redacted]
chr16	92854862	92854901	[redacted]	-0.379655	[redacted]
chr7	128364279	128364333	[redacted]	-0.379629	[redacted]
chr6	66875677	66875709	[redacted]	-0.379625	[redacted]
chr9	77353493	77353540	[redacted]	-0.379604	[redacted]
chr13	102976785	102976792	[redacted]	-0.379601	[redacted]
chr10	15746675	15746737	[redacted]	-0.379593	[redacted]
chr6	125737594	125737634	[redacted]	-0.379518	[redacted]
chr14	47676864	47676952	[redacted]	-0.379515	[redacted]
chr4	57000308	57000382	[redacted]	-0.379513	[redacted]
chrX	102504184	102504221	[redacted]	-0.379512	[redacted]
chr3	19573355	19573425	[redacted]	-0.379486	[redacted]
chr13	103589175	103589244	[redacted]	-0.379484	[redacted]
chr1	137048664	137048685	[redacted]	-0.379472	[redacted]
chr17	4536723	4536783	[redacted]	-0.379465	[redacted]
chr7	5013198	5013227	[redacted]	-0.379458	[redacted]
chr13	81785808	81785955	[redacted]	-0.379447	[redacted]
chr4	104450783	104450826	[redacted]	-0.379435	[redacted]
chr13	96293939	96293996	[redacted]	-0.379431	[redacted]
chr14	118890266	118890351	[redacted]	-0.379413	[redacted]
chr2	161070314	161070425	[redacted]	-0.379407	[redacted]
chr7	114154495	114154596	[redacted]	-0.379403	[redacted]
chr15	95383379	95383451	[redacted]	-0.37939	[redacted]
chr19	36927801	36927884	[redacted]	-0.37936	[redacted]
chr6	125777172	125777252	[redacted]	-0.379351	[redacted]
chr18	9227210	9227299	[redacted]	-0.379338	[redacted]
chr2	114615741	114615797	[redacted]	-0.379337	[redacted]
chr2	71207427	71207476	[redacted]	-0.379312	[redacted]
chr4	56918299	56918370	[redacted]	-0.379286	[redacted]
chr12	85070140	85070220	[redacted]	-0.379277	[redacted]

chr13	110484846	110484860	[redacted]	-0.379274	[redacted]
chr13	114884099	114884153	[redacted]	-0.379255	[redacted]
chr8	44304207	44304275	[redacted]	-0.379224	[redacted]
chr10	88840670	88840734	[redacted]	-0.379218	[redacted]
chr19	48008486	48008562	[redacted]	-0.379153	[redacted]
chr15	15465084	15465175	[redacted]	-0.379129	[redacted]
chr2	157984786	157984817	[redacted]	-0.379129	[redacted]
chr7	12640478	12640552	[redacted]	-0.379105	[redacted]
chr15	32403535	32403634	[redacted]	-0.3791	[redacted]
chr9	105796063	105796104	[redacted]	-0.379099	[redacted]
chr12	102973592	102973708	[redacted]	-0.379091	[redacted]
chr10	68283552	68283575	[redacted]	-0.379086	[redacted]
chr19	37990374	37990439	[redacted]	-0.379086	[redacted]
chr8	62967332	62967392	[redacted]	-0.379019	[redacted]
chr6	114900499	114900589	[redacted]	-0.379018	[redacted]
chr9	43412250	43412286	[redacted]	-0.379011	[redacted]
chr13	96435389	96435435	[redacted]	-0.379006	[redacted]
chr4	149367998	149368113	[redacted]	-0.378983	[redacted]
chr18	77559903	77559968	[redacted]	-0.378971	[redacted]
chr11	69517828	69517862	[redacted]	-0.378962	[redacted]
chr9	85519783	85519832	[redacted]	-0.378956	[redacted]
chr10	20046874	20046924	[redacted]	-0.378949	[redacted]
chr9	83546463	83546528	[redacted]	-0.378929	[redacted]
chr15	57173880	57173902	[redacted]	-0.378917	[redacted]
chr16	35756338	35756389	[redacted]	-0.37885	[redacted]
chr17	50759419	50759507	[redacted]	-0.378839	[redacted]
chr14	109269497	109269550	[redacted]	-0.378818	[redacted]
chr11	86600422	86600514	[redacted]	-0.378795	[redacted]
chr14	75244070	75244110	[redacted]	-0.378795	[redacted]
chr4	137134488	137134523	[redacted]	-0.378722	[redacted]
chr9	119547610	119547620	[redacted]	-0.37872	[redacted]
chr6	114936467	114936528	[redacted]	-0.378701	[redacted]
chr1	181996829	181996910	[redacted]	-0.378691	[redacted]
chr1	85584352	85584484	[redacted]	-0.378675	[redacted]
chr11	75659460	75659619	[redacted]	-0.378652	[redacted]
chr12	106680238	106680295	[redacted]	-0.378648	[redacted]
chr4	33013011	33013025	[redacted]	-0.378644	[redacted]
chr2	72189347	72189378	[redacted]	-0.378628	[redacted]
chr6	28609571	28609635	[redacted]	-0.378588	[redacted]
chr7	30611727	30611788	[redacted]	-0.378587	[redacted]
chr14	118039614	118039650	[redacted]	-0.378546	[redacted]
chr14	45525681	45525751	[redacted]	-0.378449	[redacted]
chr3	79366313	79366370	[redacted]	-0.378424	[redacted]

chr2	92521691	92521721	[redacted]	-0.378423	[redacted]
chr18	14494406	14494445	[redacted]	-0.378398	[redacted]
chr9	7915311	7915317	[redacted]	-0.378383	[redacted]
chr13	55724596	55724658	[redacted]	-0.378357	[redacted]
chr15	63092387	63092461	[redacted]	-0.378303	[redacted]
chr3	69869203	69869262	[redacted]	-0.378277	[redacted]
chr5	150845885	150845951	[redacted]	-0.378271	[redacted]
chr7	90276065	90276122	[redacted]	-0.378265	[redacted]
chr12	96623576	96623639	[redacted]	-0.378236	[redacted]
chr11	54775241	54775325	[redacted]	-0.378229	[redacted]
chr1	106615702	106615764	[redacted]	-0.378219	[redacted]
chr7	123453297	123453369	[redacted]	-0.378208	[redacted]
chr14	111243638	111243703	[redacted]	-0.378187	[redacted]
chr15	82289212	82289249	[redacted]	-0.378168	[redacted]
chr18	68095667	68095731	[redacted]	-0.378146	[redacted]
chr1	58417144	58417190	[redacted]	-0.378127	[redacted]
chr17	62273568	62273617	[redacted]	-0.378114	[redacted]
chr10	21473413	21473488	[redacted]	-0.378108	[redacted]
chr12	69393084	69393112	[redacted]	-0.3781	[redacted]
chr2	152235844	152235881	[redacted]	-0.378099	[redacted]
chr18	82224657	82224692	[redacted]	-0.378089	[redacted]
chr3	109189161	109189239	[redacted]	-0.378078	[redacted]
chr15	77361009	77361077	[redacted]	-0.378076	[redacted]
chr6	144550862	144550932	[redacted]	-0.378069	[redacted]
chr19	36059308	36059379	[redacted]	-0.378065	[redacted]
chr19	41953227	41953300	[redacted]	-0.37803	[redacted]
chr14	57902957	57903015	[redacted]	-0.378024	[redacted]
chr16	49846137	49846195	[redacted]	-0.377998	[redacted]
chr6	125256785	125256843	[redacted]	-0.37796	[redacted]
chr10	85015112	85015175	[redacted]	-0.377952	[redacted]
chr5	84472363	84472424	[redacted]	-0.377946	[redacted]
chr15	96753504	96753510	[redacted]	-0.377921	[redacted]
chr13	106816932	106816998	[redacted]	-0.377906	[redacted]
chr2	101985542	101985567	[redacted]	-0.377905	[redacted]
chr19	57440165	57440219	[redacted]	-0.377895	[redacted]
chr3	149686834	149686879	[redacted]	-0.377852	[redacted]
chr5	92077585	92077652	[redacted]	-0.37785	[redacted]
chr2	45024334	45024399	[redacted]	-0.377836	[redacted]
chr5	84341857	84341942	[redacted]	-0.377817	[redacted]
chr16	95504392	95504430	[redacted]	-0.377808	[redacted]
chr7	27444452	27444534	[redacted]	-0.377788	[redacted]
chr9	86567536	86567600	[redacted]	-0.377757	[redacted]
chr10	98921590	98921620	[redacted]	-0.377752	[redacted]

chr11	59449390	59449459	[redacted]	-0.377749	[redacted]
chr17	8633222	8633297	[redacted]	-0.377731	[redacted]
chr4	85140994	85141042	[redacted]	-0.377711	[redacted]
chr14	95013389	95013447	[redacted]	-0.377677	[redacted]
chr3	36478905	36478945	[redacted]	-0.377677	[redacted]
chr6	52700986	52701022	[redacted]	-0.377665	[redacted]
chr6	37535195	37535258	[redacted]	-0.377661	[redacted]
chr3	138453220	138453346	[redacted]	-0.377655	[redacted]
chr12	73606317	73606348	[redacted]	-0.377629	[redacted]
chr5	76022054	76022140	[redacted]	-0.3776	[redacted]
chr19	47554770	47554832	[redacted]	-0.377564	[redacted]
chr11	61878459	61878577	[redacted]	-0.377534	[redacted]
chr15	20601005	20601052	[redacted]	-0.377451	[redacted]
chr1	59298551	59298644	[redacted]	-0.37745	[redacted]
chr19	34362097	34362166	[redacted]	-0.377402	[redacted]
chr15	61533073	61533161	[redacted]	-0.377395	[redacted]
chr5	100737299	100737336	[redacted]	-0.377367	[redacted]
chr15	96310286	96310305	[redacted]	-0.377363	[redacted]
chr11	72461124	72461166	[redacted]	-0.377294	[redacted]
chr15	36411889	36411957	[redacted]	-0.37728	[redacted]
chr9	108145441	108145481	[redacted]	-0.377266	[redacted]
chr9	86323966	86324040	[redacted]	-0.377259	[redacted]
chr9	36758751	36758802	[redacted]	-0.377247	[redacted]
chr9	113466424	113466468	[redacted]	-0.377235	[redacted]
chr15	76556426	76556458	[redacted]	-0.377193	[redacted]
chr14	60451848	60451881	[redacted]	-0.377138	[redacted]
chr6	142595503	142595586	[redacted]	-0.377134	[redacted]
chr16	86209983	86210057	[redacted]	-0.377092	[redacted]
chr7	101388414	101388537	[redacted]	-0.377037	[redacted]
chr8	116460721	116460914	[redacted]	-0.377022	[redacted]
chr3	69311824	69311900	[redacted]	-0.377004	[redacted]
chr5	98508561	98508620	[redacted]	-0.376992	[redacted]
chr3	134489886	134489942	[redacted]	-0.376941	[redacted]
chrX	153713902	153713965	[redacted]	-0.376924	[redacted]
chr10	59691813	59691891	[redacted]	-0.376911	[redacted]
chr8	67723771	67723834	[redacted]	-0.376891	[redacted]
chr9	104348292	104348318	[redacted]	-0.376886	[redacted]
chr6	117624506	117624557	[redacted]	-0.376878	[redacted]
chr7	49412175	49412267	[redacted]	-0.376844	[redacted]
chr17	29694287	29694325	[redacted]	-0.376788	[redacted]
chr13	20331455	20331497	[redacted]	-0.376781	[redacted]
chr5	103786471	103786534	[redacted]	-0.376779	[redacted]
chr13	113954558	113954638	[redacted]	-0.376776	[redacted]

chr3	69019593	69019656	[redacted]	-0.37677	[redacted]
chr10	95247527	95247566	[redacted]	-0.376732	[redacted]
chr12	103611254	103611381	[redacted]	-0.376708	[redacted]
chr15	34890362	34890440	[redacted]	-0.376707	[redacted]
chr6	106745933	106745988	[redacted]	-0.376686	[redacted]
chr2	152752536	152752600	[redacted]	-0.376686	[redacted]
chr4	58583579	58583662	[redacted]	-0.376668	[redacted]
chr6	125220735	125220769	[redacted]	-0.376645	[redacted]
chr7	96267048	96267109	[redacted]	-0.376642	[redacted]
chr19	55209753	55209887	[redacted]	-0.376629	[redacted]
chr2	68990072	68990102	[redacted]	-0.376554	[redacted]
chr3	59326302	59326333	[redacted]	-0.376552	[redacted]
chr5	60118263	60118316	[redacted]	-0.376533	[redacted]
chr10	19901693	19901715	[redacted]	-0.376512	[redacted]
chr11	42551397	42551475	[redacted]	-0.376501	[redacted]
chr6	86386787	86386850	[redacted]	-0.376486	[redacted]
chr15	84030597	84030650	[redacted]	-0.376472	[redacted]
chr6	97329448	97329489	[redacted]	-0.376365	[redacted]
chr6	49107225	49107377	[redacted]	-0.376344	[redacted]
chr2	174328963	174329012	[redacted]	-0.376336	[redacted]
chr9	59693733	59693766	[redacted]	-0.376333	[redacted]
chr11	102726846	102726913	[redacted]	-0.376326	[redacted]
chr9	101889188	101889251	[redacted]	-0.3763	[redacted]
chr11	52285413	52285491	[redacted]	-0.376287	[redacted]
chr5	114078653	114078697	[redacted]	-0.376247	[redacted]
chr2	162526269	162526326	[redacted]	-0.376237	[redacted]
chr7	99241370	99241399	[redacted]	-0.376232	[redacted]
chr3	131071451	131071489	[redacted]	-0.376229	[redacted]
chr10	40234610	40234639	[redacted]	-0.376225	[redacted]
chr8	61356702	61356717	[redacted]	-0.37622	[redacted]
chr7	47063926	47063949	[redacted]	-0.376218	[redacted]
chr13	114953466	114953484	[redacted]	-0.376162	[redacted]
chr14	117679335	117679382	[redacted]	-0.37615	[redacted]
chr5	96818197	96818272	[redacted]	-0.376148	[redacted]
chr2	71839251	71839334	[redacted]	-0.37614	[redacted]
chr13	12519069	12519110	[redacted]	-0.376133	[redacted]
chr1	142959345	142959442	[redacted]	-0.3761	[redacted]
chr9	111970435	111970512	[redacted]	-0.376095	[redacted]
chr2	124558256	124558315	[redacted]	-0.376082	[redacted]
chr5	126603387	126603423	[redacted]	-0.376074	[redacted]
chr11	5204973	5205037	[redacted]	-0.376072	[redacted]
chr12	80028964	80029023	[redacted]	-0.376011	[redacted]
chr1	85164636	85164698	[redacted]	-0.375994	[redacted]

chr3	48049562	48049612	[redacted]	-0.375987	[redacted]
chr7	6324768	6324796	[redacted]	-0.375979	[redacted]
chr11	7219943	7220043	[redacted]	-0.37589	[redacted]
chr12	107932115	107932153	[redacted]	-0.375841	[redacted]
chr18	80367311	80367372	[redacted]	-0.375835	[redacted]
chr17	34538290	34538338	[redacted]	-0.3758	[redacted]
chr10	45848232	45848301	[redacted]	-0.375773	[redacted]
chr4	126033867	126033948	[redacted]	-0.375725	[redacted]
chr6	115849441	115849475	[redacted]	-0.375686	[redacted]
chr7	101005734	101005792	[redacted]	-0.37567	[redacted]
chr16	94300279	94300309	[redacted]	-0.375669	[redacted]
chr9	20612171	20612241	[redacted]	-0.375663	[redacted]
chr12	99696343	99696361	[redacted]	-0.375649	[redacted]
chr3	69889200	69889285	[redacted]	-0.375638	[redacted]
chr1	137931008	137931048	[redacted]	-0.375615	[redacted]
chr7	119596076	119596167	[redacted]	-0.375602	[redacted]
chr9	107067697	107067755	[redacted]	-0.375572	[redacted]
chr5	114766411	114766444	[redacted]	-0.375571	[redacted]
chr19	28104824	28104856	[redacted]	-0.375543	[redacted]
chr15	50694533	50694554	[redacted]	-0.375541	[redacted]
chr14	78738749	78738770	[redacted]	-0.375506	[redacted]
chr15	93958888	93958944	[redacted]	-0.375494	[redacted]
chr3	101913009	101913076	[redacted]	-0.375471	[redacted]
chrY	90740965	90740978	[redacted]	-0.375464	[redacted]
chr12	70636659	70636687	[redacted]	-0.375448	[redacted]
chr11	36409646	36409698	[redacted]	-0.375435	[redacted]
chr16	90446914	90446941	[redacted]	-0.37541	[redacted]
chr14	110444013	110444099	[redacted]	-0.375377	[redacted]
chr14	85807983	85808057	[redacted]	-0.375363	[redacted]
chr4	40836500	40836586	[redacted]	-0.375284	[redacted]
chr7	66386483	66386582	[redacted]	-0.375277	[redacted]
chr13	112758911	112758977	[redacted]	-0.375252	[redacted]
chr11	53752963	53753000	[redacted]	-0.375223	[redacted]
chr13	109506356	109506390	[redacted]	-0.375198	[redacted]
chr5	19410524	19410583	[redacted]	-0.375197	[redacted]
chr4	105252124	105252196	[redacted]	-0.375186	[redacted]
chr4	87998095	87998147	[redacted]	-0.37518	[redacted]
chr2	165588866	165588984	[redacted]	-0.375167	[redacted]
chr3	132714269	132714340	[redacted]	-0.375135	[redacted]
chr7	110637622	110637687	[redacted]	-0.375134	[redacted]
chr12	74581165	74581269	[redacted]	-0.375131	[redacted]
chr6	71430607	71430656	[redacted]	-0.375105	[redacted]
chr1	38455235	38455251	[redacted]	-0.375059	[redacted]

chr5	146335660	146335716	[redacted]	-0.375056	[redacted]
chr2	71271211	71271354	[redacted]	-0.375052	[redacted]
chr1	136974535	136974560	[redacted]	-0.375051	[redacted]
chr7	127516493	127516544	[redacted]	-0.375042	[redacted]
chr8	80737607	80737673	[redacted]	-0.37504	[redacted]
chr8	31968030	31968067	[redacted]	-0.374968	[redacted]
chr6	135207224	135207251	[redacted]	-0.374961	[redacted]
chr5	65806513	65806549	[redacted]	-0.374901	[redacted]
chr8	73583768	73583827	[redacted]	-0.374898	[redacted]
chr6	3206332	3206372	[redacted]	-0.374853	[redacted]
chr7	74115154	74115180	[redacted]	-0.374803	[redacted]
chr10	52961877	52961940	[redacted]	-0.374803	[redacted]
chr2	92317383	92317527	[redacted]	-0.374791	[redacted]
chr9	104760618	104760682	[redacted]	-0.374757	[redacted]
chr2	7480560	7480586	[redacted]	-0.374679	[redacted]
chr3	126702484	126702562	[redacted]	-0.374677	[redacted]
chr13	119463611	119463642	[redacted]	-0.374674	[redacted]
chr6	65608973	65609031	[redacted]	-0.374674	[redacted]
chr12	12338917	12338942	[redacted]	-0.374652	[redacted]
chr5	77408506	77408583	[redacted]	-0.374645	[redacted]
chr4	34886118	34886165	[redacted]	-0.374637	[redacted]
chrX	159462021	159462071	[redacted]	-0.374627	[redacted]
chr10	39533286	39533344	[redacted]	-0.374483	[redacted]
chr16	92959114	92959151	[redacted]	-0.37446	[redacted]
chr6	134266557	134266593	[redacted]	-0.374443	[redacted]
chr13	114479354	114479396	[redacted]	-0.374441	[redacted]
chr8	9973644	9973706	[redacted]	-0.37444	[redacted]
chr2	157952458	157952540	[redacted]	-0.374412	[redacted]
chr7	116824493	116824584	[redacted]	-0.374398	[redacted]
chr9	88653740	88653822	[redacted]	-0.374299	[redacted]
chr15	92609463	92609530	[redacted]	-0.37427	[redacted]
chr11	77661956	77662011	[redacted]	-0.374239	[redacted]
chr7	10604716	10604748	[redacted]	-0.374238	[redacted]
chr1	138081779	138081842	[redacted]	-0.374236	[redacted]
chr1	9681775	9681839	[redacted]	-0.374236	[redacted]
chr3	34658941	34658986	[redacted]	-0.374225	[redacted]
chr2	43694898	43694963	[redacted]	-0.374182	[redacted]
chr6	43257068	43257136	[redacted]	-0.374172	[redacted]
chr15	102104572	102104636	[redacted]	-0.37417	[redacted]
chr4	9485618	9485651	[redacted]	-0.374132	[redacted]
chr1	181357263	181357301	[redacted]	-0.374127	[redacted]
chr15	25477038	25477107	[redacted]	-0.374053	[redacted]
chr14	26915040	26915060	[redacted]	-0.374046	[redacted]

chr3	81753671	81753718	[redacted]	-0.374021	[redacted]
chr16	94522366	94522400	[redacted]	-0.374019	[redacted]
chr10	20481124	20481209	[redacted]	-0.374015	[redacted]
chr2	129149913	129149938	[redacted]	-0.374009	[redacted]
chr9	44207586	44207674	[redacted]	-0.373996	[redacted]
chrX	99534129	99534199	[redacted]	-0.373983	[redacted]
chr9	28864639	28864713	[redacted]	-0.373976	[redacted]
chr4	22207321	22207367	[redacted]	-0.373969	[redacted]
chr8	79756932	79756996	[redacted]	-0.373956	[redacted]
chr1	88499630	88499685	[redacted]	-0.373924	[redacted]
chr10	116935313	116935359	[redacted]	-0.37391	[redacted]
chr9	39040544	39040574	[redacted]	-0.373892	[redacted]
chr12	9700604	9700663	[redacted]	-0.37388	[redacted]
chr9	89805776	89805789	[redacted]	-0.373878	[redacted]
chr15	30952764	30952831	[redacted]	-0.373862	[redacted]
chr1	158916573	158916663	[redacted]	-0.373842	[redacted]
chr16	30572963	30572981	[redacted]	-0.373837	[redacted]
chr2	150663677	150663728	[redacted]	-0.373813	[redacted]
chr4	13030331	13030412	[redacted]	-0.373813	[redacted]
chr1	50159873	50159891	[redacted]	-0.37381	[redacted]
chr6	28789600	28789652	[redacted]	-0.37376	[redacted]
chr5	48146450	48146494	[redacted]	-0.37375	[redacted]
chr7	80210422	80210515	[redacted]	-0.373726	[redacted]
chr19	23579340	23579372	[redacted]	-0.373717	[redacted]
chr4	107339383	107339442	[redacted]	-0.373686	[redacted]
chr18	62824385	62824455	[redacted]	-0.37367	[redacted]
chr16	36661547	36661626	[redacted]	-0.373661	[redacted]
chr15	6972604	6972677	[redacted]	-0.37365	[redacted]
chr9	38306409	38306467	[redacted]	-0.373648	[redacted]
chr5	73253611	73253632	[redacted]	-0.373645	[redacted]
chr10	40946951	40947013	[redacted]	-0.373629	[redacted]
chr1	167911119	167911202	[redacted]	-0.373628	[redacted]
chr2	52467390	52467476	[redacted]	-0.3736	[redacted]
chr9	90110443	90110548	[redacted]	-0.373598	[redacted]
chr3	94216729	94216780	[redacted]	-0.373594	[redacted]
chr10	93854926	93854986	[redacted]	-0.373584	[redacted]
chr2	13195505	13195552	[redacted]	-0.373561	[redacted]
chr11	24949760	24949786	[redacted]	-0.373546	[redacted]
chr13	79333369	79333417	[redacted]	-0.373518	[redacted]
chr17	86485834	86485902	[redacted]	-0.373509	[redacted]
chr2	79052121	79052184	[redacted]	-0.373474	[redacted]
chr1	176053170	176053235	[redacted]	-0.373474	[redacted]
chr16	80563368	80563429	[redacted]	-0.373467	[redacted]

chr10	84494420	84494437	[redacted]	-0.373446	[redacted]
chr6	50765954	50766033	[redacted]	-0.373443	[redacted]
chr10	117949613	117949670	[redacted]	-0.373417	[redacted]
chr10	18390102	18390117	[redacted]	-0.373403	[redacted]
chr5	33139438	33139502	[redacted]	-0.373378	[redacted]
chr5	100423687	100423744	[redacted]	-0.373371	[redacted]
chr1	29687413	29687477	[redacted]	-0.373336	[redacted]
chr17	3704499	3704571	[redacted]	-0.373193	[redacted]
chr13	110353319	110353405	[redacted]	-0.373186	[redacted]
chr14	67879813	67879895	[redacted]	-0.373165	[redacted]
chr4	137635002	137635063	[redacted]	-0.373155	[redacted]
chr9	117526770	117526794	[redacted]	-0.373151	[redacted]
chr2	44166189	44166221	[redacted]	-0.373143	[redacted]
chr16	13060701	13060822	[redacted]	-0.373121	[redacted]
chr1	172014896	172014970	[redacted]	-0.373109	[redacted]
chr9	57885014	57885075	[redacted]	-0.373107	[redacted]
chr4	107316973	107317041	[redacted]	-0.373065	[redacted]
chr2	3418465	3418498	[redacted]	-0.372969	[redacted]
chr13	81753445	81753496	[redacted]	-0.372924	[redacted]
chr3	50301971	50302026	[redacted]	-0.372901	[redacted]
chr6	24729952	24730027	[redacted]	-0.372894	[redacted]
chr11	5069582	5069669	[redacted]	-0.372881	[redacted]
chr9	118683813	118683866	[redacted]	-0.372879	[redacted]
chr12	15864128	15864183	[redacted]	-0.372865	[redacted]
chr9	108197312	108197360	[redacted]	-0.372823	[redacted]
chr15	3374795	3374845	[redacted]	-0.372807	[redacted]
chr17	44552513	44552570	[redacted]	-0.372801	[redacted]
chr5	148866098	148866119	[redacted]	-0.37278	[redacted]
chr6	35245222	35245281	[redacted]	-0.372778	[redacted]
chr11	59209922	59209948	[redacted]	-0.37277	[redacted]
chr1	162352406	162352469	[redacted]	-0.372762	[redacted]
chr15	52861831	52861898	[redacted]	-0.372739	[redacted]
chr17	78110731	78110788	[redacted]	-0.372736	[redacted]
chr9	122218686	122218773	[redacted]	-0.37273	[redacted]
chr18	75523892	75523909	[redacted]	-0.372724	[redacted]
chr9	109120122	109120150	[redacted]	-0.372708	[redacted]
chr9	67005644	67005742	[redacted]	-0.372704	[redacted]
chr1	140775225	140775303	[redacted]	-0.372695	[redacted]
chr4	141511914	141512004	[redacted]	-0.372688	[redacted]
chr15	10462091	10462132	[redacted]	-0.372677	[redacted]
chr16	21360162	21360232	[redacted]	-0.372676	[redacted]
chr11	57977185	57977202	[redacted]	-0.372658	[redacted]
chr14	45611277	45611304	[redacted]	-0.372584	[redacted]

chr1	113621287	113621374	[redacted]	-0.372519	[redacted]
chr16	65015456	65015552	[redacted]	-0.372518	[redacted]
chr9	49677142	49677194	[redacted]	-0.372516	[redacted]
chr15	25136139	25136211	[redacted]	-0.372514	[redacted]
chr14	76854862	76854898	[redacted]	-0.372511	[redacted]
chr17	29534392	29534455	[redacted]	-0.372498	[redacted]
chr18	83116367	83116381	[redacted]	-0.372466	[redacted]
chr3	23572018	23572058	[redacted]	-0.372465	[redacted]
chr16	41314791	41314838	[redacted]	-0.372458	[redacted]
chr19	32067906	32067974	[redacted]	-0.372457	[redacted]
chr4	151874672	151874724	[redacted]	-0.372453	[redacted]
chr6	148261805	148261865	[redacted]	-0.372444	[redacted]
chr12	80224296	80224373	[redacted]	-0.372434	[redacted]
chr19	40284423	40284475	[redacted]	-0.372417	[redacted]
chrX	145338250	145338323	[redacted]	-0.372411	[redacted]
chr13	39625123	39625208	[redacted]	-0.372406	[redacted]
chr12	119218512	119218584	[redacted]	-0.372406	[redacted]
chr19	56717528	56717598	[redacted]	-0.372351	[redacted]
chr10	114100509	114100534	[redacted]	-0.372322	[redacted]
chr16	93144180	93144222	[redacted]	-0.372315	[redacted]
chr4	85063324	85063386	[redacted]	-0.372307	[redacted]
chr8	14052303	14052335	[redacted]	-0.372273	[redacted]
chr18	84877481	84877535	[redacted]	-0.372269	[redacted]
chr5	148569527	148569561	[redacted]	-0.37222	[redacted]
chr15	73283254	73283312	[redacted]	-0.372216	[redacted]
chr12	91772736	91772802	[redacted]	-0.372215	[redacted]
chr15	96696766	96696804	[redacted]	-0.372206	[redacted]
chr16	32947852	32947953	[redacted]	-0.372186	[redacted]
chr18	71428213	71428272	[redacted]	-0.372186	[redacted]
chr11	29878873	29878901	[redacted]	-0.372154	[redacted]
chr1	59033091	59033140	[redacted]	-0.372148	[redacted]
chr5	65877485	65877511	[redacted]	-0.372141	[redacted]
chrX	105794663	105794717	[redacted]	-0.372137	[redacted]
chr12	41826417	41826490	[redacted]	-0.37212	[redacted]
chr8	89341207	89341272	[redacted]	-0.372115	[redacted]
chr15	39637217	39637231	[redacted]	-0.372089	[redacted]
chr15	11431409	11431486	[redacted]	-0.372046	[redacted]
chr13	81736703	81736726	[redacted]	-0.372003	[redacted]
chr1	181944713	181944843	[redacted]	-0.371989	[redacted]
chr6	21889808	21889892	[redacted]	-0.371986	[redacted]
chr6	127128468	127128489	[redacted]	-0.371984	[redacted]
chr6	41016926	41016996	[redacted]	-0.3719	[redacted]
chr9	78458546	78458609	[redacted]	-0.371883	[redacted]

chr6	123688637	123688729	[redacted]	-0.371803	[redacted]
chr7	101515449	101515537	[redacted]	-0.371792	[redacted]
chr11	5821053	5821109	[redacted]	-0.371771	[redacted]
chrX	12247753	12247785	[redacted]	-0.371759	[redacted]
chr18	24542379	24542449	[redacted]	-0.371746	[redacted]
chrX	99357740	99357798	[redacted]	-0.371746	[redacted]
chr5	134991513	134991549	[redacted]	-0.37171	[redacted]
chr3	109259702	109259784	[redacted]	-0.371707	[redacted]
chr8	48447481	48447540	[redacted]	-0.371697	[redacted]
chr8	120840647	120840700	[redacted]	-0.37166	[redacted]
chr11	98530683	98530761	[redacted]	-0.371614	[redacted]
chr17	65281821	65281869	[redacted]	-0.37161	[redacted]
chr7	122464406	122464452	[redacted]	-0.37159	[redacted]
chr19	26308206	26308272	[redacted]	-0.371545	[redacted]
chr11	13229289	13229338	[redacted]	-0.371539	[redacted]
chr11	94917044	94917100	[redacted]	-0.371521	[redacted]
chr6	7615790	7615869	[redacted]	-0.37148	[redacted]
chr12	72094836	72094882	[redacted]	-0.371464	[redacted]
chr1	177776499	177776553	[redacted]	-0.371448	[redacted]
chr2	165802979	165802992	[redacted]	-0.371401	[redacted]
chr11	120994125	120994191	[redacted]	-0.371354	[redacted]
chr17	35262032	35262054	[redacted]	-0.371343	[redacted]
chr5	116489797	116489826	[redacted]	-0.371342	[redacted]
chr9	118160652	118160690	[redacted]	-0.371325	[redacted]
chr4	117838610	117838688	[redacted]	-0.371314	[redacted]
chr17	3756586	3756651	[redacted]	-0.371297	[redacted]
chr14	97396551	97396616	[redacted]	-0.371282	[redacted]
chrX	12125587	12125622	[redacted]	-0.371277	[redacted]
chr5	70290260	70290339	[redacted]	-0.371257	[redacted]
chr3	102189304	102189394	[redacted]	-0.37123	[redacted]
chr7	96581767	96581844	[redacted]	-0.371211	[redacted]
chr14	74900279	74900381	[redacted]	-0.37121	[redacted]
chr9	84114020	84114145	[redacted]	-0.371198	[redacted]
chr2	152632600	152632664	[redacted]	-0.371153	[redacted]
chr13	69196201	69196254	[redacted]	-0.371135	[redacted]
chr6	112685239	112685329	[redacted]	-0.371122	[redacted]
chr11	95443982	95444049	[redacted]	-0.371119	[redacted]
chr2	69076768	69076823	[redacted]	-0.371101	[redacted]
chr8	108136141	108136155	[redacted]	-0.371078	[redacted]
chr5	63154736	63154796	[redacted]	-0.371076	[redacted]
chr9	110204177	110204340	[redacted]	-0.371049	[redacted]
chr11	90270571	90270594	[redacted]	-0.371027	[redacted]
chr3	97218310	97218357	[redacted]	-0.371	[redacted]

chr9	82876084	82876164	[redacted]	-0.371	[redacted]
chr3	103173307	103173345	[redacted]	-0.37095	[redacted]
chr17	36906365	36906443	[redacted]	-0.370925	[redacted]
chr6	8855651	8855726	[redacted]	-0.370915	[redacted]
chr4	22958734	22958782	[redacted]	-0.370906	[redacted]
chr10	22729905	22729924	[redacted]	-0.370895	[redacted]
chr13	100071816	100071881	[redacted]	-0.370869	[redacted]
chr16	23247080	23247119	[redacted]	-0.370843	[redacted]
chr7	84631150	84631309	[redacted]	-0.370836	[redacted]
chr15	99066768	99066800	[redacted]	-0.370818	[redacted]
chr16	94962119	94962164	[redacted]	-0.370812	[redacted]
chr3	107901904	107901944	[redacted]	-0.370803	[redacted]
chr7	130518699	130518733	[redacted]	-0.370756	[redacted]
chr1	193555798	193555852	[redacted]	-0.370718	[redacted]
chr2	173138923	173138944	[redacted]	-0.3707	[redacted]
chr13	93773030	93773114	[redacted]	-0.370681	[redacted]
chr13	89354016	89354064	[redacted]	-0.370668	[redacted]
chr6	141327914	141327978	[redacted]	-0.370642	[redacted]
chr16	57303505	57303546	[redacted]	-0.370633	[redacted]
chr10	99298279	99298396	[redacted]	-0.370607	[redacted]
chr17	46109378	46109387	[redacted]	-0.370604	[redacted]
chr8	94829142	94829209	[redacted]	-0.370584	[redacted]
chr4	64001228	64001315	[redacted]	-0.37058	[redacted]
chr15	84463603	84463669	[redacted]	-0.370573	[redacted]
chr2	172121510	172121535	[redacted]	-0.370555	[redacted]
chr8	125938727	125938817	[redacted]	-0.37053	[redacted]
chr9	108687166	108687280	[redacted]	-0.370527	[redacted]
chr3	147007452	147007501	[redacted]	-0.370505	[redacted]
chr12	75790226	75790277	[redacted]	-0.37047	[redacted]
chr4	39803201	39803274	[redacted]	-0.370461	[redacted]
chr5	63800419	63800439	[redacted]	-0.370451	[redacted]
chr19	25526930	25526979	[redacted]	-0.370446	[redacted]
chr4	45663122	45663199	[redacted]	-0.370407	[redacted]
chr4	126236604	126236672	[redacted]	-0.370383	[redacted]
chr15	58803813	58803872	[redacted]	-0.37038	[redacted]
chr9	79805149	79805213	[redacted]	-0.37029	[redacted]
chr18	34328930	34328970	[redacted]	-0.37027	[redacted]
chr1	38955618	38955664	[redacted]	-0.370245	[redacted]
chr4	107100107	107100185	[redacted]	-0.370187	[redacted]
chr3	129425068	129425151	[redacted]	-0.370181	[redacted]
chr4	109575873	109575952	[redacted]	-0.370109	[redacted]
chr2	59384269	59384334	[redacted]	-0.3701	[redacted]
chr4	104198664	104198740	[redacted]	-0.370083	[redacted]

chr10	108379956	108380029	[redacted]	-0.370073	[redacted]
chr16	31651113	31651150	[redacted]	-0.370053	[redacted]
chr14	59796256	59796292	[redacted]	-0.370042	[redacted]
chr7	84136718	84136728	[redacted]	-0.370011	[redacted]
chr16	4846038	4846082	[redacted]	-0.369996	[redacted]
chr10	97475958	97476038	[redacted]	-0.369995	[redacted]
chr12	73559615	73559689	[redacted]	-0.369991	[redacted]
chr5	66858768	66858839	[redacted]	-0.369985	[redacted]
chr1	13053106	13053123	[redacted]	-0.369966	[redacted]
chr18	54236049	54236116	[redacted]	-0.369931	[redacted]
chr5	149843034	149843082	[redacted]	-0.369909	[redacted]
chr4	9622288	9622383	[redacted]	-0.369904	[redacted]
chr3	145167932	145168027	[redacted]	-0.369865	[redacted]
chr18	75807838	75807902	[redacted]	-0.369835	[redacted]
chr15	34087318	34087365	[redacted]	-0.369834	[redacted]
chr11	95113357	95113390	[redacted]	-0.369811	[redacted]
chr15	28110305	28110335	[redacted]	-0.369797	[redacted]
chr7	99824985	99825042	[redacted]	-0.369775	[redacted]
chr18	52472355	52472410	[redacted]	-0.369756	[redacted]
chr5	120232897	120232950	[redacted]	-0.369747	[redacted]
chr16	70399527	70399585	[redacted]	-0.369732	[redacted]
chr1	53718406	53718460	[redacted]	-0.369718	[redacted]
chr19	18678047	18678103	[redacted]	-0.36968	[redacted]
chr11	94925795	94925888	[redacted]	-0.369678	[redacted]
chr6	40110688	40110727	[redacted]	-0.369659	[redacted]
chr14	14390020	14390073	[redacted]	-0.369629	[redacted]
chr15	84875593	84875643	[redacted]	-0.369605	[redacted]
chr5	147291443	147291506	[redacted]	-0.3696	[redacted]
chr13	36637948	36638001	[redacted]	-0.369582	[redacted]
chr7	122316866	122316939	[redacted]	-0.369566	[redacted]
chr19	40068608	40068615	[redacted]	-0.369563	[redacted]
chr7	58695315	58695457	[redacted]	-0.369561	[redacted]
chr14	62665747	62665764	[redacted]	-0.369481	[redacted]
chr1	88482707	88482784	[redacted]	-0.369458	[redacted]
chr6	49633267	49633304	[redacted]	-0.369433	[redacted]
chr1	93769246	93769329	[redacted]	-0.36943	[redacted]
chr3	151913004	151913071	[redacted]	-0.369418	[redacted]
chr17	40285235	40285271	[redacted]	-0.369397	[redacted]
chr1	33620115	33620159	[redacted]	-0.369391	[redacted]
chr13	104678937	104678986	[redacted]	-0.369387	[redacted]
chr8	107100387	107100468	[redacted]	-0.369355	[redacted]
chr1	68183297	68183317	[redacted]	-0.369344	[redacted]
chr2	37852347	37852410	[redacted]	-0.369335	[redacted]

chr18	40439700	40439747	[redacted]	-0.369323	[redacted]
chr3	38908222	38908272	[redacted]	-0.369308	[redacted]
chr17	54400128	54400215	[redacted]	-0.369297	[redacted]
chr7	19189238	19189255	[redacted]	-0.369209	[redacted]
chr7	113960905	113960954	[redacted]	-0.369172	[redacted]
chr5	107793130	107793201	[redacted]	-0.369168	[redacted]
chr13	10913190	10913232	[redacted]	-0.369154	[redacted]
chr3	152956639	152956722	[redacted]	-0.369147	[redacted]
chr1	36807006	36807063	[redacted]	-0.369113	[redacted]
chr5	45039556	45039606	[redacted]	-0.369081	[redacted]
chr12	91177773	91177788	[redacted]	-0.369069	[redacted]
chr5	16237672	16237711	[redacted]	-0.369029	[redacted]
chr8	77102983	77103033	[redacted]	-0.368994	[redacted]
chr8	14658152	14658167	[redacted]	-0.368988	[redacted]
chr16	63404862	63404921	[redacted]	-0.368973	[redacted]
chr11	80692935	80692996	[redacted]	-0.368943	[redacted]
chr10	122430808	122430847	[redacted]	-0.368925	[redacted]
chr10	12547920	12548003	[redacted]	-0.368921	[redacted]
chr19	40658587	40658657	[redacted]	-0.368903	[redacted]
chr9	61934431	61934460	[redacted]	-0.368901	[redacted]
chr10	125647815	125647880	[redacted]	-0.368864	[redacted]
chr8	77018578	77018621	[redacted]	-0.368855	[redacted]
chr16	64898324	64898374	[redacted]	-0.368854	[redacted]
chr8	9971026	9971076	[redacted]	-0.368834	[redacted]
chr2	139911574	139911609	[redacted]	-0.368811	[redacted]
chr6	8435620	8435675	[redacted]	-0.368811	[redacted]
chr1	187122774	187122841	[redacted]	-0.368795	[redacted]
chr12	36268748	36268798	[redacted]	-0.368734	[redacted]
chr3	108947086	108947121	[redacted]	-0.368677	[redacted]
chr1	133733086	133733128	[redacted]	-0.368675	[redacted]
chr5	30494954	30495041	[redacted]	-0.368662	[redacted]
chr1	189932264	189932345	[redacted]	-0.368628	[redacted]
chr16	43807301	43807351	[redacted]	-0.36853	[redacted]
chr6	119726293	119726319	[redacted]	-0.368422	[redacted]
chr17	65727723	65727763	[redacted]	-0.368406	[redacted]
chr2	29849635	29849666	[redacted]	-0.368397	[redacted]
chr5	80196471	80196570	[redacted]	-0.368392	[redacted]
chr3	130758477	130758528	[redacted]	-0.368379	[redacted]
chr16	50407842	50407889	[redacted]	-0.368344	[redacted]
chr7	115445049	115445065	[redacted]	-0.368332	[redacted]
chr17	24959477	24959491	[redacted]	-0.368318	[redacted]
chr1	156514155	156514187	[redacted]	-0.368288	[redacted]
chr19	16633613	16633689	[redacted]	-0.368278	[redacted]

chr4	5652045	5652110	[redacted]	-0.368261	[redacted]
chr18	57941033	57941052	[redacted]	-0.36826	[redacted]
chr15	88645954	88645987	[redacted]	-0.368258	[redacted]
chr13	90935995	90936018	[redacted]	-0.368237	[redacted]
chr11	96450848	96450881	[redacted]	-0.368234	[redacted]
chr14	94893085	94893111	[redacted]	-0.368234	[redacted]
chr11	86739982	86740035	[redacted]	-0.368185	[redacted]
chr5	147348531	147348587	[redacted]	-0.368176	[redacted]
chr2	125280901	125280945	[redacted]	-0.368173	[redacted]
chr16	23793493	23793633	[redacted]	-0.36817	[redacted]
chr5	33824839	33824894	[redacted]	-0.36814	[redacted]
chr4	46147983	46148069	[redacted]	-0.368129	[redacted]
chr11	98104082	98104100	[redacted]	-0.368126	[redacted]
chr12	68665975	68666040	[redacted]	-0.368122	[redacted]
chr16	95563077	95563136	[redacted]	-0.368122	[redacted]
chr3	132885327	132885407	[redacted]	-0.368093	[redacted]
chr14	119203640	119203690	[redacted]	-0.368083	[redacted]
chr10	43091227	43091284	[redacted]	-0.368082	[redacted]
chrX	23453661	23453703	[redacted]	-0.368066	[redacted]
chr16	66848468	66848512	[redacted]	-0.368022	[redacted]
chr17	32659766	32659831	[redacted]	-0.368021	[redacted]
chr5	77449059	77449101	[redacted]	-0.368005	[redacted]
chr12	54113593	54113629	[redacted]	-0.367971	[redacted]
chr17	3313885	3313939	[redacted]	-0.367965	[redacted]
chr5	101567877	101568037	[redacted]	-0.367949	[redacted]
chr3	129746680	129746718	[redacted]	-0.367936	[redacted]
chr10	4267739	4267773	[redacted]	-0.367915	[redacted]
chr15	94704458	94704534	[redacted]	-0.367901	[redacted]
chr9	111682557	111682604	[redacted]	-0.367876	[redacted]
chr9	83865854	83865904	[redacted]	-0.367872	[redacted]
chr10	96956200	96956226	[redacted]	-0.367861	[redacted]
chr14	55947569	55947602	[redacted]	-0.367856	[redacted]
chr9	49127234	49127266	[redacted]	-0.367838	[redacted]
chr9	95970723	95970787	[redacted]	-0.367836	[redacted]
chr2	118216691	118216812	[redacted]	-0.36781	[redacted]
chr17	67181732	67181817	[redacted]	-0.367783	[redacted]
chr3	152449657	152449775	[redacted]	-0.367769	[redacted]
chr13	58353936	58353995	[redacted]	-0.367762	[redacted]
chr18	55603255	55603349	[redacted]	-0.36776	[redacted]
chr1	189526286	189526328	[redacted]	-0.367728	[redacted]
chr8	125141919	125141953	[redacted]	-0.367712	[redacted]
chr2	153773970	153774017	[redacted]	-0.367696	[redacted]
chr12	102676302	102676359	[redacted]	-0.367677	[redacted]

chr17	31262545	31262618	[redacted]	-0.367662	[redacted]
chr2	53198002	53198034	[redacted]	-0.367642	[redacted]
chr6	52600297	52600376	[redacted]	-0.367641	[redacted]
chr7	80775322	80775392	[redacted]	-0.367627	[redacted]
chr18	78169045	78169064	[redacted]	-0.367605	[redacted]
chr17	33595869	33595948	[redacted]	-0.367592	[redacted]
chr7	63718320	63718372	[redacted]	-0.367547	[redacted]
chr1	135934195	135934292	[redacted]	-0.367544	[redacted]
chr11	69998772	69998821	[redacted]	-0.367539	[redacted]
chr4	107433592	107433626	[redacted]	-0.367531	[redacted]
chr17	11721945	11722004	[redacted]	-0.367469	[redacted]
chr10	86984306	86984380	[redacted]	-0.367449	[redacted]
chr6	91849891	91849932	[redacted]	-0.36741	[redacted]
chr8	107189345	107189450	[redacted]	-0.367405	[redacted]
chr11	43437365	43437449	[redacted]	-0.367403	[redacted]
chr5	73131994	73132045	[redacted]	-0.367401	[redacted]
chr8	46868812	46868947	[redacted]	-0.367344	[redacted]
chr19	28302267	28302395	[redacted]	-0.36734	[redacted]
chr3	156629268	156629310	[redacted]	-0.367301	[redacted]
chrX	11692939	11692951	[redacted]	-0.3673	[redacted]
chr10	39352433	39352483	[redacted]	-0.367295	[redacted]
chr14	51375996	51376030	[redacted]	-0.367266	[redacted]
chr5	23627047	23627058	[redacted]	-0.367255	[redacted]
chr18	38661148	38661178	[redacted]	-0.367209	[redacted]
chrX	73916571	73916603	[redacted]	-0.367154	[redacted]
chr15	5122240	5122293	[redacted]	-0.367128	[redacted]
chr6	55005553	55005593	[redacted]	-0.36709	[redacted]
chr5	130528691	130528786	[redacted]	-0.367064	[redacted]
chr6	67176951	67177034	[redacted]	-0.36706	[redacted]
chr6	64119517	64119538	[redacted]	-0.367048	[redacted]
chr16	67149871	67149942	[redacted]	-0.366998	[redacted]
chr15	95487919	95487942	[redacted]	-0.366997	[redacted]
chr4	17657690	17657759	[redacted]	-0.366984	[redacted]
chr14	117476254	117476305	[redacted]	-0.366979	[redacted]
chr12	119896326	119896391	[redacted]	-0.366977	[redacted]
chr5	49053465	49053485	[redacted]	-0.366971	[redacted]
chr7	97126377	97126440	[redacted]	-0.366951	[redacted]
chr1	75508712	75508777	[redacted]	-0.366946	[redacted]
chr5	150548226	150548278	[redacted]	-0.366945	[redacted]
chr10	11092285	11092338	[redacted]	-0.366935	[redacted]
chr17	94478532	94478596	[redacted]	-0.366798	[redacted]
chr18	42054735	42054818	[redacted]	-0.366795	[redacted]
chr2	129743111	129743145	[redacted]	-0.366788	[redacted]

chr3	27251929	27252005	[redacted]	-0.366785	[redacted]
chr15	99788369	99788414	[redacted]	-0.366758	[redacted]
chrX	130351804	130351863	[redacted]	-0.366742	[redacted]
chr7	94437838	94437908	[redacted]	-0.366737	[redacted]
chr13	33067094	33067208	[redacted]	-0.366726	[redacted]
chr11	49209309	49209343	[redacted]	-0.366721	[redacted]
chr13	41571405	41571479	[redacted]	-0.366712	[redacted]
chr10	43619809	43619844	[redacted]	-0.366697	[redacted]
chr1	38911683	38911757	[redacted]	-0.36667	[redacted]
chr15	27368826	27368881	[redacted]	-0.366667	[redacted]
chr12	86226049	86226119	[redacted]	-0.366655	[redacted]
chr1	90951484	90951524	[redacted]	-0.366654	[redacted]
chr3	9000113	9000164	[redacted]	-0.36665	[redacted]
chr2	117329355	117329399	[redacted]	-0.366641	[redacted]
chr7	126149243	126149332	[redacted]	-0.36663	[redacted]
chr6	83282455	83282528	[redacted]	-0.366624	[redacted]
chr8	86920103	86920138	[redacted]	-0.366624	[redacted]
chrX	99383875	99383949	[redacted]	-0.36661	[redacted]
chr7	111003075	111003144	[redacted]	-0.366606	[redacted]
chr5	75683964	75684015	[redacted]	-0.366592	[redacted]
chr12	84968317	84968388	[redacted]	-0.366563	[redacted]
chr1	176586193	176586272	[redacted]	-0.366536	[redacted]
chr4	102929055	102929221	[redacted]	-0.366528	[redacted]
chr4	127358555	127358564	[redacted]	-0.366517	[redacted]
chr3	109479337	109479380	[redacted]	-0.366495	[redacted]
chr15	72498180	72498257	[redacted]	-0.366494	[redacted]
chr4	118139421	118139564	[redacted]	-0.366436	[redacted]
chr7	139717349	139717445	[redacted]	-0.366414	[redacted]
chr3	95947252	95947286	[redacted]	-0.366397	[redacted]
chr2	140523927	140523980	[redacted]	-0.36639	[redacted]
chr3	146176675	146176798	[redacted]	-0.366384	[redacted]
chr11	101354884	101354909	[redacted]	-0.366364	[redacted]
chr15	39412427	39412473	[redacted]	-0.366362	[redacted]
chr8	117974994	117975069	[redacted]	-0.366358	[redacted]
chr18	12644688	12644720	[redacted]	-0.366356	[redacted]
chr4	149975992	149976054	[redacted]	-0.366355	[redacted]
chr6	85283990	85284083	[redacted]	-0.366333	[redacted]
chr12	71339588	71339652	[redacted]	-0.366327	[redacted]
chr10	59453368	59453409	[redacted]	-0.366306	[redacted]
chr9	106615724	106615782	[redacted]	-0.36627	[redacted]
chr13	81668581	81668611	[redacted]	-0.36626	[redacted]
chr16	78686336	78686393	[redacted]	-0.36626	[redacted]
chr2	134343886	134343958	[redacted]	-0.366254	[redacted]

chr19	48304509	48304573	[redacted]	-0.366238	[redacted]
chr12	73805179	73805240	[redacted]	-0.366232	[redacted]
chr16	96156264	96156348	[redacted]	-0.366226	[redacted]
chr6	35221290	35221362	[redacted]	-0.366208	[redacted]
chr13	110036241	110036306	[redacted]	-0.366204	[redacted]
chr1	181947151	181947207	[redacted]	-0.366196	[redacted]
chr2	115907669	115907748	[redacted]	-0.366192	[redacted]
chr5	119606439	119606487	[redacted]	-0.366161	[redacted]
chr5	66240564	66240600	[redacted]	-0.36614	[redacted]
chr19	36305694	36305782	[redacted]	-0.366126	[redacted]
chr16	34360992	34361014	[redacted]	-0.366094	[redacted]
chr10	89731190	89731245	[redacted]	-0.36607	[redacted]
chr16	78373368	78373409	[redacted]	-0.36606	[redacted]
chr1	13842393	13842434	[redacted]	-0.36603	[redacted]
chr1	161907265	161907329	[redacted]	-0.365971	[redacted]
chr11	7452511	7452588	[redacted]	-0.365946	[redacted]
chrX	93186485	93186510	[redacted]	-0.365926	[redacted]
chr2	93558710	93558804	[redacted]	-0.365923	[redacted]
chr15	58941705	58941749	[redacted]	-0.365898	[redacted]
chr11	69080709	69080773	[redacted]	-0.36587	[redacted]
chr9	56011855	56011893	[redacted]	-0.365864	[redacted]
chr8	78085391	78085513	[redacted]	-0.365855	[redacted]
chr3	104544207	104544269	[redacted]	-0.36583	[redacted]
chr11	50881346	50881397	[redacted]	-0.365818	[redacted]
chr6	145892561	145892576	[redacted]	-0.365812	[redacted]
chr2	70576427	70576480	[redacted]	-0.365778	[redacted]
chr11	74443616	74443696	[redacted]	-0.365758	[redacted]
chr2	51604882	51604936	[redacted]	-0.365753	[redacted]
chr15	52506055	52506135	[redacted]	-0.365745	[redacted]
chr18	63249050	63249211	[redacted]	-0.36572	[redacted]
chr6	50617860	50617912	[redacted]	-0.36572	[redacted]
chr4	21797333	21797400	[redacted]	-0.365714	[redacted]
chr5	117936656	117936706	[redacted]	-0.365682	[redacted]
chr8	24742940	24742994	[redacted]	-0.365677	[redacted]
chr3	148806150	148806194	[redacted]	-0.365671	[redacted]
chr6	112623567	112623648	[redacted]	-0.365638	[redacted]
chr8	34164116	34164201	[redacted]	-0.365636	[redacted]
chr15	34114786	34114850	[redacted]	-0.36562	[redacted]
chr5	72741160	72741173	[redacted]	-0.365603	[redacted]
chr10	85402837	85402957	[redacted]	-0.365588	[redacted]
chrX	38881203	38881280	[redacted]	-0.365514	[redacted]
chr18	13028366	13028426	[redacted]	-0.365501	[redacted]
chr3	130541017	130541054	[redacted]	-0.365467	[redacted]

chr2	71647473	71647574	[redacted]	-0.365446	[redacted]
chr6	64920299	64920363	[redacted]	-0.365433	[redacted]
chr15	41843083	41843130	[redacted]	-0.36536	[redacted]
chr16	97985214	97985245	[redacted]	-0.365335	[redacted]
chr3	54760014	54760082	[redacted]	-0.365322	[redacted]
chr9	49502776	49502862	[redacted]	-0.365312	[redacted]
chr1	69014976	69015016	[redacted]	-0.365288	[redacted]
chr19	58090650	58090721	[redacted]	-0.365284	[redacted]
chr4	85665468	85665481	[redacted]	-0.36528	[redacted]
chr13	75419061	75419123	[redacted]	-0.365248	[redacted]
chr12	91727646	91727668	[redacted]	-0.365212	[redacted]
chr17	79280788	79280823	[redacted]	-0.365179	[redacted]
chr9	83218689	83218732	[redacted]	-0.365176	[redacted]
chr11	108870517	108870584	[redacted]	-0.365171	[redacted]
chr6	122632965	122633017	[redacted]	-0.365145	[redacted]
chr6	65840806	65840866	[redacted]	-0.365135	[redacted]
chr17	67581918	67581969	[redacted]	-0.365113	[redacted]
chr18	33088088	33088099	[redacted]	-0.365081	[redacted]
chr11	69113642	69113683	[redacted]	-0.365076	[redacted]
chr2	149150770	149150841	[redacted]	-0.365042	[redacted]
chrX	51724205	51724249	[redacted]	-0.365027	[redacted]
chr11	5952148	5952189	[redacted]	-0.365016	[redacted]
chr3	87255271	87255374	[redacted]	-0.365014	[redacted]
chr2	151215945	151215966	[redacted]	-0.365008	[redacted]
chr5	53577172	53577318	[redacted]	-0.364996	[redacted]
chr13	46835454	46835539	[redacted]	-0.364989	[redacted]
chr19	37381224	37381246	[redacted]	-0.364985	[redacted]
chr7	50338969	50339062	[redacted]	-0.364976	[redacted]
chr9	112024475	112024531	[redacted]	-0.364959	[redacted]
chr2	68406918	68406967	[redacted]	-0.364906	[redacted]
chr1	105422758	105422837	[redacted]	-0.364897	[redacted]
chr6	96850129	96850184	[redacted]	-0.364889	[redacted]
chr6	101871959	101871990	[redacted]	-0.364824	[redacted]
chr2	32179595	32179647	[redacted]	-0.364786	[redacted]
chr2	51692998	51693030	[redacted]	-0.364776	[redacted]
chr11	107107128	107107174	[redacted]	-0.364774	[redacted]
chr19	36767437	36767466	[redacted]	-0.36476	[redacted]
chr15	51828334	51828489	[redacted]	-0.364745	[redacted]
chr12	103578405	103578448	[redacted]	-0.364686	[redacted]
chr5	21651927	21651976	[redacted]	-0.364659	[redacted]
chr16	95815393	95815495	[redacted]	-0.364652	[redacted]
chr8	75301468	75301532	[redacted]	-0.364633	[redacted]
chr11	98513849	98513898	[redacted]	-0.364588	[redacted]

chr7	31250611	31250685	[redacted]	-0.364523	[redacted]
chr3	79576649	79576709	[redacted]	-0.364512	[redacted]
chr2	77017499	77017556	[redacted]	-0.3645	[redacted]
chr5	123745573	123745616	[redacted]	-0.364498	[redacted]
chr18	75258181	75258299	[redacted]	-0.364495	[redacted]
chr3	109520511	109520536	[redacted]	-0.364488	[redacted]
chr5	121179225	121179240	[redacted]	-0.364474	[redacted]
chr7	106706698	106706746	[redacted]	-0.364457	[redacted]
chr14	99569364	99569449	[redacted]	-0.364429	[redacted]
chr13	24740884	24740944	[redacted]	-0.364426	[redacted]
chr8	34100983	34101039	[redacted]	-0.364422	[redacted]
chr4	127198978	127199004	[redacted]	-0.364415	[redacted]
chr9	69482429	69482515	[redacted]	-0.364385	[redacted]
chr11	78126426	78126480	[redacted]	-0.364373	[redacted]
chr2	117326123	117326182	[redacted]	-0.364344	[redacted]
chr11	70382055	70382073	[redacted]	-0.364338	[redacted]
chr4	138479182	138479323	[redacted]	-0.364334	[redacted]
chr13	52437146	52437207	[redacted]	-0.364329	[redacted]
chr1	72243444	72243497	[redacted]	-0.364305	[redacted]
chr8	41091652	41091723	[redacted]	-0.364293	[redacted]
chr12	79299981	79300009	[redacted]	-0.364235	[redacted]
chr18	20870638	20870679	[redacted]	-0.364232	[redacted]
chr3	34813666	34813705	[redacted]	-0.364213	[redacted]
chr8	34506718	34506794	[redacted]	-0.364199	[redacted]
chr16	6030209	6030237	[redacted]	-0.36416	[redacted]
chr2	74919058	74919156	[redacted]	-0.364157	[redacted]
chr17	62696348	62696417	[redacted]	-0.364151	[redacted]
chr5	126899611	126899672	[redacted]	-0.364144	[redacted]
chr10	59171661	59171740	[redacted]	-0.364125	[redacted]
chr13	111671129	111671176	[redacted]	-0.364122	[redacted]
chr8	77107699	77107830	[redacted]	-0.364015	[redacted]
chr6	76979804	76979859	[redacted]	-0.364005	[redacted]
chr7	127093845	127093949	[redacted]	-0.364003	[redacted]
chr2	119422078	119422123	[redacted]	-0.363998	[redacted]
chr19	28125171	28125225	[redacted]	-0.363981	[redacted]
chr1	4451792	4451935	[redacted]	-0.363971	[redacted]
chr18	43359465	43359518	[redacted]	-0.363956	[redacted]
chr11	86077103	86077139	[redacted]	-0.363943	[redacted]
chr1	30284649	30284702	[redacted]	-0.363918	[redacted]
chr10	125292039	125292107	[redacted]	-0.363901	[redacted]
chr15	94869538	94869617	[redacted]	-0.363882	[redacted]
chr10	92899533	92899559	[redacted]	-0.363874	[redacted]
chr3	154210612	154210653	[redacted]	-0.363862	[redacted]

chr15	36074439	36074499	[redacted]	-0.363823	[redacted]
chr8	26852091	26852145	[redacted]	-0.363813	[redacted]
chr14	54782543	54782573	[redacted]	-0.363804	[redacted]
chr2	30070446	30070495	[redacted]	-0.363787	[redacted]
chr17	6840137	6840235	[redacted]	-0.363778	[redacted]
chr3	36681343	36681381	[redacted]	-0.363771	[redacted]
chr1	90737164	90737248	[redacted]	-0.363758	[redacted]
chr12	82180733	82180789	[redacted]	-0.363713	[redacted]
chr1	86659866	86659916	[redacted]	-0.363706	[redacted]
chr5	89553274	89553353	[redacted]	-0.363703	[redacted]
chr12	56297501	56297528	[redacted]	-0.363668	[redacted]
chr11	106780299	106780335	[redacted]	-0.363645	[redacted]
chr13	51250604	51250693	[redacted]	-0.363637	[redacted]
chr17	50517696	50517747	[redacted]	-0.363631	[redacted]
chr3	51826431	51826503	[redacted]	-0.363623	[redacted]
chr6	120122289	120122335	[redacted]	-0.363605	[redacted]
chr12	84066608	84066632	[redacted]	-0.36357	[redacted]
chr15	57670681	57670756	[redacted]	-0.363567	[redacted]
chr1	36543836	36543899	[redacted]	-0.363555	[redacted]
chr9	74328546	74328602	[redacted]	-0.363525	[redacted]
chr3	100304204	100304263	[redacted]	-0.363511	[redacted]
chr13	89176058	89176135	[redacted]	-0.363499	[redacted]
chr5	65832039	65832131	[redacted]	-0.363462	[redacted]
chr3	58961643	58961696	[redacted]	-0.363456	[redacted]
chr15	99792499	99792561	[redacted]	-0.363438	[redacted]
chr12	7630254	7630318	[redacted]	-0.363425	[redacted]
chr7	31317710	31317746	[redacted]	-0.363421	[redacted]
chr15	39051024	39051055	[redacted]	-0.363381	[redacted]
chr4	152238931	152238972	[redacted]	-0.363371	[redacted]
chr11	97179988	97180046	[redacted]	-0.363363	[redacted]
chr2	62489446	62489518	[redacted]	-0.36334	[redacted]
chr8	77651494	77651536	[redacted]	-0.363339	[redacted]
chr1	181672859	181672921	[redacted]	-0.363304	[redacted]
chr14	122359419	122359459	[redacted]	-0.363268	[redacted]
chr8	103604114	103604180	[redacted]	-0.363263	[redacted]
chr9	118163745	118163794	[redacted]	-0.363249	[redacted]
chr2	104376652	104376746	[redacted]	-0.363238	[redacted]
chr7	65984686	65984709	[redacted]	-0.363221	[redacted]
chr17	81071542	81071608	[redacted]	-0.363218	[redacted]
chr13	97245067	97245077	[redacted]	-0.363203	[redacted]
chr2	169475894	169475958	[redacted]	-0.363161	[redacted]
chr7	115305607	115305656	[redacted]	-0.363138	[redacted]
chr16	46051756	46051831	[redacted]	-0.363132	[redacted]

chr16	91616112	91616181	[redacted]	-0.363113	[redacted]
chr16	33255013	33255063	[redacted]	-0.363101	[redacted]
chr13	45868265	45868340	[redacted]	-0.363076	[redacted]
chr19	47950627	47950768	[redacted]	-0.363052	[redacted]
chr4	48109179	48109223	[redacted]	-0.36304	[redacted]
chr15	100747907	100747978	[redacted]	-0.363035	[redacted]
chr5	147634378	147634529	[redacted]	-0.36302	[redacted]
chr18	80709718	80709760	[redacted]	-0.363006	[redacted]
chr17	88186820	88186887	[redacted]	-0.362992	[redacted]
chr8	111088296	111088348	[redacted]	-0.362901	[redacted]
chr18	68697741	68697821	[redacted]	-0.362867	[redacted]
chr11	7105482	7105550	[redacted]	-0.362867	[redacted]
chr3	142090818	142090855	[redacted]	-0.362847	[redacted]
chr14	75448656	75448748	[redacted]	-0.362846	[redacted]
chr3	107429709	107429782	[redacted]	-0.362845	[redacted]
chr13	115231220	115231279	[redacted]	-0.362779	[redacted]
chr11	103462939	103462999	[redacted]	-0.362754	[redacted]
chr16	87560368	87560381	[redacted]	-0.36275	[redacted]
chr5	8651759	8651812	[redacted]	-0.362741	[redacted]
chr2	127132027	127132084	[redacted]	-0.362721	[redacted]
chr12	53267703	53267745	[redacted]	-0.362693	[redacted]
chr1	172988425	172988532	[redacted]	-0.362687	[redacted]
chr18	41860876	41860951	[redacted]	-0.362662	[redacted]
chr1	134493240	134493299	[redacted]	-0.362658	[redacted]
chr9	97367615	97367670	[redacted]	-0.362615	[redacted]
chr8	112209593	112209635	[redacted]	-0.362597	[redacted]
chr19	45067132	45067189	[redacted]	-0.362589	[redacted]
chr17	74753074	74753106	[redacted]	-0.362584	[redacted]
chr7	31245854	31245871	[redacted]	-0.362576	[redacted]
chr17	5079657	5079695	[redacted]	-0.362575	[redacted]
chr2	122041635	122041718	[redacted]	-0.362573	[redacted]
chr8	94265896	94265979	[redacted]	-0.362573	[redacted]
chrX	15251016	15251093	[redacted]	-0.362562	[redacted]
chr11	68445330	68445441	[redacted]	-0.362552	[redacted]
chr8	47981968	47982011	[redacted]	-0.362534	[redacted]
chr10	111489583	111489660	[redacted]	-0.36252	[redacted]
chr12	106857169	106857185	[redacted]	-0.362501	[redacted]
chr3	152065643	152065677	[redacted]	-0.362491	[redacted]
chr3	39174921	39175038	[redacted]	-0.36249	[redacted]
chr4	107225085	107225146	[redacted]	-0.362489	[redacted]
chr11	76851094	76851152	[redacted]	-0.362476	[redacted]
chrX	113487758	113487786	[redacted]	-0.362474	[redacted]
chr9	109883566	109883636	[redacted]	-0.362472	[redacted]

chr13	24494166	24494315	[redacted]	-0.362454	[redacted]
chr17	83084943	83084986	[redacted]	-0.362448	[redacted]
chr8	94946747	94946867	[redacted]	-0.362429	[redacted]
chr13	3549872	3549930	[redacted]	-0.362423	[redacted]
chr3	135216318	135216403	[redacted]	-0.362422	[redacted]
chr13	38609435	38609500	[redacted]	-0.362415	[redacted]
chr11	76191423	76191505	[redacted]	-0.362393	[redacted]
chr8	109515027	109515099	[redacted]	-0.362384	[redacted]
chr19	16463671	16463752	[redacted]	-0.362354	[redacted]
chr3	126759458	126759525	[redacted]	-0.362337	[redacted]
chr12	56877140	56877176	[redacted]	-0.362325	[redacted]
chr11	69718763	69718772	[redacted]	-0.362318	[redacted]
chr9	75944362	75944417	[redacted]	-0.362317	[redacted]
chr4	41515475	41515541	[redacted]	-0.362315	[redacted]
chr11	56484577	56484628	[redacted]	-0.362293	[redacted]
chr2	70967657	70967744	[redacted]	-0.362232	[redacted]
chr6	52949876	52949928	[redacted]	-0.362196	[redacted]
chr4	35754961	35755045	[redacted]	-0.36219	[redacted]
chr13	13479524	13479617	[redacted]	-0.362159	[redacted]
chr7	51388461	51388517	[redacted]	-0.362152	[redacted]
chr15	15320830	15320921	[redacted]	-0.362136	[redacted]
chr13	51146438	51146497	[redacted]	-0.362109	[redacted]
chr3	48741060	48741153	[redacted]	-0.362105	[redacted]
chr10	7728360	7728415	[redacted]	-0.362094	[redacted]
chr2	101286692	101286719	[redacted]	-0.362076	[redacted]
chr17	82827730	82827796	[redacted]	-0.361962	[redacted]
chr6	126746921	126747004	[redacted]	-0.361962	[redacted]
chr8	109108215	109108249	[redacted]	-0.361941	[redacted]
chr3	154148723	154148792	[redacted]	-0.361939	[redacted]
chr16	91293132	91293181	[redacted]	-0.361931	[redacted]
chrX	73535405	73535451	[redacted]	-0.361837	[redacted]
chr4	58039444	58039506	[redacted]	-0.361823	[redacted]
chr8	105632500	105632559	[redacted]	-0.361817	[redacted]
chr12	34133696	34133754	[redacted]	-0.361812	[redacted]
chr2	93562846	93562902	[redacted]	-0.361811	[redacted]
chr2	46768557	46768603	[redacted]	-0.361729	[redacted]
chr1	39906516	39906584	[redacted]	-0.361718	[redacted]
chr2	66499424	66499495	[redacted]	-0.361718	[redacted]
chr18	59104158	59104190	[redacted]	-0.361711	[redacted]
chr2	62355708	62355786	[redacted]	-0.361663	[redacted]
chr9	92550903	92550961	[redacted]	-0.361649	[redacted]
chr2	166579573	166579635	[redacted]	-0.361638	[redacted]
chr9	56324070	56324107	[redacted]	-0.361638	[redacted]

chr9	96379711	96379735	[redacted]	-0.361603	[redacted]
chr4	132394527	132394556	[redacted]	-0.361598	[redacted]
chr7	92638071	92638144	[redacted]	-0.361571	[redacted]
chr4	57941326	57941408	[redacted]	-0.361569	[redacted]
chr14	59743979	59744044	[redacted]	-0.361558	[redacted]
chr10	87226791	87226851	[redacted]	-0.361555	[redacted]
chr1	38726591	38726690	[redacted]	-0.361549	[redacted]
chr12	4686144	4686174	[redacted]	-0.361517	[redacted]
chr1	183553448	183553538	[redacted]	-0.361502	[redacted]
chr5	86790333	86790396	[redacted]	-0.361474	[redacted]
chr10	117842737	117842771	[redacted]	-0.361468	[redacted]
chr9	29565421	29565499	[redacted]	-0.361468	[redacted]
chr14	19064833	19064892	[redacted]	-0.361452	[redacted]
chr1	91864499	91864570	[redacted]	-0.361436	[redacted]
chr5	72258890	72258945	[redacted]	-0.36139	[redacted]
chr6	110629840	110629878	[redacted]	-0.361366	[redacted]
chr9	95104406	95104470	[redacted]	-0.361328	[redacted]
chr16	35638180	35638242	[redacted]	-0.361309	[redacted]
chr8	109348618	109348704	[redacted]	-0.361275	[redacted]
chr5	52174431	52174504	[redacted]	-0.361253	[redacted]
chr7	17062611	17062655	[redacted]	-0.361208	[redacted]
chr1	59300288	59300404	[redacted]	-0.361207	[redacted]
chr7	37868323	37868342	[redacted]	-0.361191	[redacted]
chr15	96295271	96295288	[redacted]	-0.361187	[redacted]
chr3	154812031	154812135	[redacted]	-0.361177	[redacted]
chr13	99433463	99433576	[redacted]	-0.361164	[redacted]
chr7	82765830	82765882	[redacted]	-0.36116	[redacted]
chr12	54268813	54268840	[redacted]	-0.361088	[redacted]
chr9	57483863	57483929	[redacted]	-0.361076	[redacted]
chr12	70714436	70714515	[redacted]	-0.361064	[redacted]
chr14	75838559	75838626	[redacted]	-0.361058	[redacted]
chr13	88836530	88836559	[redacted]	-0.361052	[redacted]
chr11	33654844	33654941	[redacted]	-0.361045	[redacted]
chr13	37849273	37849305	[redacted]	-0.361031	[redacted]
chr1	132623010	132623029	[redacted]	-0.361028	[redacted]
chr8	112420405	112420473	[redacted]	-0.360988	[redacted]
chr12	17814147	17814191	[redacted]	-0.360964	[redacted]
chr3	76416159	76416251	[redacted]	-0.36095	[redacted]
chr8	64732175	64732214	[redacted]	-0.360936	[redacted]
chr5	96529229	96529324	[redacted]	-0.360916	[redacted]
chr16	35586944	35587009	[redacted]	-0.36091	[redacted]
chr18	58108818	58108902	[redacted]	-0.360894	[redacted]
chr3	84738512	84738553	[redacted]	-0.360862	[redacted]

chr6	37594180	37594232	[redacted]	-0.360859	[redacted]
chrX	101902990	101903051	[redacted]	-0.360858	[redacted]
chr5	108677365	108677427	[redacted]	-0.360808	[redacted]
chr6	42445950	42445984	[redacted]	-0.360725	[redacted]
chr8	81098251	81098308	[redacted]	-0.360697	[redacted]
chr19	17085127	17085191	[redacted]	-0.360688	[redacted]
chr11	53477229	53477246	[redacted]	-0.36068	[redacted]
chr15	50686587	50686619	[redacted]	-0.36068	[redacted]
chr17	70239441	70239487	[redacted]	-0.360676	[redacted]
chr12	86103279	86103330	[redacted]	-0.360627	[redacted]
chr6	97745920	97746006	[redacted]	-0.360626	[redacted]
chr2	25923438	25923518	[redacted]	-0.360605	[redacted]
chr6	129322872	129322919	[redacted]	-0.360597	[redacted]
chr5	65915286	65915314	[redacted]	-0.360593	[redacted]
chr12	75375874	75375935	[redacted]	-0.360578	[redacted]
chr9	111521990	111522047	[redacted]	-0.36057	[redacted]
chrX	48027673	48027695	[redacted]	-0.360516	[redacted]
chr13	76117634	76117675	[redacted]	-0.360516	[redacted]
chr9	7430661	7430728	[redacted]	-0.360513	[redacted]
chr10	127920442	127920492	[redacted]	-0.360465	[redacted]
chr10	58284699	58284757	[redacted]	-0.360438	[redacted]
chr1	39313053	39313119	[redacted]	-0.360428	[redacted]
chr1	97748890	97748958	[redacted]	-0.360389	[redacted]
chr13	25702692	25702771	[redacted]	-0.360329	[redacted]
chr9	119434558	119434585	[redacted]	-0.360299	[redacted]
chr2	75795267	75795306	[redacted]	-0.360298	[redacted]
chr4	93036991	93037036	[redacted]	-0.36029	[redacted]
chr3	79944915	79944995	[redacted]	-0.360278	[redacted]
chr16	25168434	25168473	[redacted]	-0.360242	[redacted]
chr14	77678050	77678114	[redacted]	-0.360229	[redacted]
chr1	176971644	176971696	[redacted]	-0.360228	[redacted]
chr10	111248226	111248279	[redacted]	-0.360228	[redacted]
chr14	63084430	63084459	[redacted]	-0.36021	[redacted]
chr3	121894286	121894337	[redacted]	-0.360199	[redacted]
chr10	60898890	60898941	[redacted]	-0.360177	[redacted]
chr16	33069159	33069219	[redacted]	-0.360176	[redacted]
chr2	59041188	59041312	[redacted]	-0.360118	[redacted]
chr9	102710696	102710728	[redacted]	-0.360111	[redacted]
chr17	88333237	88333325	[redacted]	-0.360107	[redacted]
chr19	15544835	15544903	[redacted]	-0.36009	[redacted]
chr9	77005040	77005111	[redacted]	-0.36005	[redacted]
chr3	126600738	126600792	[redacted]	-0.360035	[redacted]
chr2	154459941	154459958	[redacted]	-0.360012	[redacted]

chr1	63250283	63250318	[redacted]	-0.359986	[redacted]
chr2	73540260	73540313	[redacted]	-0.359959	[redacted]
chr3	84568531	84568585	[redacted]	-0.359949	[redacted]
chr7	16437518	16437567	[redacted]	-0.359909	[redacted]
chr16	34105585	34105651	[redacted]	-0.359894	[redacted]
chr5	136262423	136262478	[redacted]	-0.359894	[redacted]
chr12	68567971	68568042	[redacted]	-0.359881	[redacted]
chr9	66552907	66552943	[redacted]	-0.359858	[redacted]
chr9	5166973	5167037	[redacted]	-0.359795	[redacted]
chr17	24086412	24086491	[redacted]	-0.359751	[redacted]
chr6	127078165	127078200	[redacted]	-0.359735	[redacted]
chr7	84357868	84357925	[redacted]	-0.359713	[redacted]
chr7	44724883	44724930	[redacted]	-0.359709	[redacted]
chr16	36784958	36785015	[redacted]	-0.359657	[redacted]
chr6	70649682	70649705	[redacted]	-0.359635	[redacted]
chr4	87840711	87840746	[redacted]	-0.359629	[redacted]
chr2	33975085	33975160	[redacted]	-0.359626	[redacted]
chr13	51145978	51146053	[redacted]	-0.359622	[redacted]
chr9	83402183	83402263	[redacted]	-0.359614	[redacted]
chr11	98305377	98305417	[redacted]	-0.359596	[redacted]
chr2	111812182	111812250	[redacted]	-0.359551	[redacted]
chr13	103325068	103325120	[redacted]	-0.35955	[redacted]
chr15	42174893	42174938	[redacted]	-0.359533	[redacted]
chr10	106477417	106477470	[redacted]	-0.359529	[redacted]
chr1	10930384	10930439	[redacted]	-0.359524	[redacted]
chr16	87646741	87646809	[redacted]	-0.35951	[redacted]
chr3	83493580	83493651	[redacted]	-0.359505	[redacted]
chr6	80250731	80250799	[redacted]	-0.359496	[redacted]
chr19	33242726	33242769	[redacted]	-0.359479	[redacted]
chr8	125163595	125163643	[redacted]	-0.359475	[redacted]
chr3	36822352	36822401	[redacted]	-0.359472	[redacted]
chr11	67993410	67993501	[redacted]	-0.359467	[redacted]
chr14	98984412	98984478	[redacted]	-0.359451	[redacted]
chr7	28765415	28765440	[redacted]	-0.359398	[redacted]
chr18	4755886	4755916	[redacted]	-0.359387	[redacted]
chr13	57686426	57686500	[redacted]	-0.359373	[redacted]
chr5	33341275	33341339	[redacted]	-0.359373	[redacted]
chr8	25846075	25846153	[redacted]	-0.359335	[redacted]
chr7	46598163	46598225	[redacted]	-0.359311	[redacted]
chr11	26435678	26435707	[redacted]	-0.359306	[redacted]
chr10	26344580	26344613	[redacted]	-0.359257	[redacted]
chr8	82127489	82127571	[redacted]	-0.359232	[redacted]
chr17	40061122	40061161	[redacted]	-0.3592	[redacted]

chr18	5618319	5618371	[redacted]	-0.359196	[redacted]
chr16	74396493	74396612	[redacted]	-0.359162	[redacted]
chr3	121236949	121236999	[redacted]	-0.359153	[redacted]
chr18	81752489	81752564	[redacted]	-0.35914	[redacted]
chrX	9673680	9673723	[redacted]	-0.359137	[redacted]
chr1	22393337	22393427	[redacted]	-0.359136	[redacted]
chr4	46488496	46488547	[redacted]	-0.359132	[redacted]
chr1	175086139	175086212	[redacted]	-0.359122	[redacted]
chr3	103880157	103880206	[redacted]	-0.359115	[redacted]
chr13	54900830	54900846	[redacted]	-0.35906	[redacted]
chr6	71351605	71351680	[redacted]	-0.35904	[redacted]
chr19	47666180	47666232	[redacted]	-0.359032	[redacted]
chr15	96283230	96283253	[redacted]	-0.35901	[redacted]
chr17	70460428	70460488	[redacted]	-0.358991	[redacted]
chr11	44341121	44341171	[redacted]	-0.35899	[redacted]
chr8	88692446	88692504	[redacted]	-0.358983	[redacted]
chr9	52342140	52342177	[redacted]	-0.358922	[redacted]
chr8	48165639	48165692	[redacted]	-0.358903	[redacted]
chr16	37977555	37977621	[redacted]	-0.358892	[redacted]
chr16	10651084	10651136	[redacted]	-0.358889	[redacted]
chr9	78430716	78430778	[redacted]	-0.358881	[redacted]
chr8	108398926	108399010	[redacted]	-0.358877	[redacted]
chr19	43517584	43517611	[redacted]	-0.358857	[redacted]
chr6	54426899	54426970	[redacted]	-0.358855	[redacted]
chr10	94762736	94762800	[redacted]	-0.358834	[redacted]
chr12	10000559	10000623	[redacted]	-0.358825	[redacted]
chr17	23603364	23603442	[redacted]	-0.358786	[redacted]
chr12	45743891	45744015	[redacted]	-0.358769	[redacted]
chr9	11347436	11347520	[redacted]	-0.358724	[redacted]
chr19	55554827	55554901	[redacted]	-0.35871	[redacted]
chr15	97223076	97223158	[redacted]	-0.358693	[redacted]
chr3	58986689	58986757	[redacted]	-0.358693	[redacted]
chr13	98246195	98246227	[redacted]	-0.358691	[redacted]
chr10	80288122	80288188	[redacted]	-0.358652	[redacted]
chr13	113302739	113302818	[redacted]	-0.358627	[redacted]
chr13	29588335	29588387	[redacted]	-0.358626	[redacted]
chr11	35310200	35310271	[redacted]	-0.358621	[redacted]
chr13	13686469	13686485	[redacted]	-0.35861	[redacted]
chr14	15410144	15410233	[redacted]	-0.35859	[redacted]
chr9	80595854	80595957	[redacted]	-0.358526	[redacted]
chr14	29169213	29169288	[redacted]	-0.358513	[redacted]
chr6	28640238	28640284	[redacted]	-0.358504	[redacted]
chr8	3808097	3808146	[redacted]	-0.358493	[redacted]

chr8	92894687	92894740	[redacted]	-0.358492	[redacted]
chr16	87722592	87722628	[redacted]	-0.35848	[redacted]
chr3	55021285	55021330	[redacted]	-0.358461	[redacted]
chr15	100015192	100015214	[redacted]	-0.358431	[redacted]
chr7	111462388	111462441	[redacted]	-0.358412	[redacted]
chr6	91543451	91543503	[redacted]	-0.358411	[redacted]
chr4	56809582	56809639	[redacted]	-0.358409	[redacted]
chr11	6791185	6791221	[redacted]	-0.358408	[redacted]
chr7	134892822	134892859	[redacted]	-0.358404	[redacted]
chr8	109276908	109276921	[redacted]	-0.358393	[redacted]
chr2	157349647	157349661	[redacted]	-0.35839	[redacted]
chr6	32209706	32209764	[redacted]	-0.358386	[redacted]
chr3	89835830	89835877	[redacted]	-0.358375	[redacted]
chr2	156410105	156410281	[redacted]	-0.358372	[redacted]
chr6	16055013	16055085	[redacted]	-0.358329	[redacted]
chr6	71001413	71001505	[redacted]	-0.358313	[redacted]
chr10	80171356	80171509	[redacted]	-0.358281	[redacted]
chr11	73216415	73216477	[redacted]	-0.35823	[redacted]
chr15	83061347	83061478	[redacted]	-0.358223	[redacted]
chr14	57426205	57426279	[redacted]	-0.358222	[redacted]
chr12	38442243	38442315	[redacted]	-0.358212	[redacted]
chrX	20384893	20384969	[redacted]	-0.358168	[redacted]
chr4	135868788	135868835	[redacted]	-0.358126	[redacted]
chr19	36078935	36078971	[redacted]	-0.358089	[redacted]
chrX	23620038	23620064	[redacted]	-0.358045	[redacted]
chr17	56637311	56637370	[redacted]	-0.358005	[redacted]
chr11	76891246	76891295	[redacted]	-0.358001	[redacted]
chr15	3951197	3951241	[redacted]	-0.357998	[redacted]
chr2	125788336	125788350	[redacted]	-0.357956	[redacted]
chr1	152922886	152922953	[redacted]	-0.35795	[redacted]
chr6	12545425	12545464	[redacted]	-0.357888	[redacted]
chr2	177217603	177217665	[redacted]	-0.357888	[redacted]
chr4	109337204	109337232	[redacted]	-0.357862	[redacted]
chr17	43781005	43781068	[redacted]	-0.35785	[redacted]
chr7	27796519	27796591	[redacted]	-0.357839	[redacted]
chr11	98535180	98535197	[redacted]	-0.357814	[redacted]
chrX	166172240	166172321	[redacted]	-0.357739	[redacted]
chr1	162243134	162243182	[redacted]	-0.357712	[redacted]
chr4	63387373	63387430	[redacted]	-0.35769	[redacted]
chr16	38407553	38407600	[redacted]	-0.357661	[redacted]
chr1	91643434	91643503	[redacted]	-0.357656	[redacted]
chr4	81638219	81638289	[redacted]	-0.357621	[redacted]
chr11	99008278	99008395	[redacted]	-0.357616	[redacted]

chr13	63083598	63083652	[redacted]	-0.357616	[redacted]
chr2	9642559	9642597	[redacted]	-0.357602	[redacted]
chr10	41527964	41527977	[redacted]	-0.357581	[redacted]
chr3	102049250	102049297	[redacted]	-0.357557	[redacted]
chr6	85838743	85838843	[redacted]	-0.35755	[redacted]
chr14	59514441	59514516	[redacted]	-0.35752	[redacted]
chr14	59107310	59107389	[redacted]	-0.357515	[redacted]
chr11	97690296	97690334	[redacted]	-0.357507	[redacted]
chr10	127137878	127137949	[redacted]	-0.357475	[redacted]
chrX	55516161	55516196	[redacted]	-0.357473	[redacted]
chr4	62162665	62162700	[redacted]	-0.357448	[redacted]
chr19	43577070	43577137	[redacted]	-0.357445	[redacted]
chr3	123469610	123469627	[redacted]	-0.357438	[redacted]
chr6	82456057	82456163	[redacted]	-0.35741	[redacted]
chr11	95547488	95547553	[redacted]	-0.357408	[redacted]
chrX	60316213	60316257	[redacted]	-0.35739	[redacted]
chr6	73719549	73719612	[redacted]	-0.357374	[redacted]
chr7	113853180	113853227	[redacted]	-0.357361	[redacted]
chr11	91169794	91169842	[redacted]	-0.357346	[redacted]
chr1	168243463	168243484	[redacted]	-0.357307	[redacted]
chr13	71486618	71486670	[redacted]	-0.357269	[redacted]
chr16	21850581	21850637	[redacted]	-0.357259	[redacted]
chr9	85970368	85970438	[redacted]	-0.357233	[redacted]
chr15	96866214	96866311	[redacted]	-0.357212	[redacted]
chr12	104634419	104634506	[redacted]	-0.357205	[redacted]
chr8	57754839	57754909	[redacted]	-0.357182	[redacted]
chr3	87886857	87886919	[redacted]	-0.357173	[redacted]
chr17	46866794	46866928	[redacted]	-0.357166	[redacted]
chr8	14603070	14603134	[redacted]	-0.357158	[redacted]
chr8	78473270	78473316	[redacted]	-0.357154	[redacted]
chr17	83834478	83834555	[redacted]	-0.357137	[redacted]
chr12	80762623	80762690	[redacted]	-0.357134	[redacted]
chr6	144429471	144429499	[redacted]	-0.357131	[redacted]
chr11	54567495	54567564	[redacted]	-0.357108	[redacted]
chr4	134541495	134541520	[redacted]	-0.357105	[redacted]
chr7	122356801	122356891	[redacted]	-0.357103	[redacted]
chr3	152116681	152116725	[redacted]	-0.357095	[redacted]
chr9	59983844	59983922	[redacted]	-0.357072	[redacted]
chr12	38347656	38347701	[redacted]	-0.35705	[redacted]
chr9	100447524	100447567	[redacted]	-0.357031	[redacted]
chr10	98626731	98626825	[redacted]	-0.357011	[redacted]
chr8	115064881	115064922	[redacted]	-0.356998	[redacted]
chr11	44258603	44258678	[redacted]	-0.356979	[redacted]

chr1	83679448	83679521	[redacted]	-0.356947	[redacted]
chr8	107145224	107145303	[redacted]	-0.356941	[redacted]
chr7	29846939	29846984	[redacted]	-0.35692	[redacted]
chr12	57092996	57093046	[redacted]	-0.356865	[redacted]
chr5	54098774	54098822	[redacted]	-0.356853	[redacted]
chrX	7150212	7150258	[redacted]	-0.356846	[redacted]
chr9	22788208	22788265	[redacted]	-0.356843	[redacted]
chr3	18901057	18901140	[redacted]	-0.35684	[redacted]
chr16	72062425	72062474	[redacted]	-0.35681	[redacted]
chr11	93092839	93092919	[redacted]	-0.356799	[redacted]
chr16	64867426	64867526	[redacted]	-0.356792	[redacted]
chr7	35579160	35579240	[redacted]	-0.356784	[redacted]
chr10	78501024	78501102	[redacted]	-0.356764	[redacted]
chr1	68065408	68065430	[redacted]	-0.356753	[redacted]
chr7	29164485	29164525	[redacted]	-0.356747	[redacted]
chr6	55258122	55258187	[redacted]	-0.356719	[redacted]
chr4	97654766	97654815	[redacted]	-0.356707	[redacted]
chr6	50280526	50280554	[redacted]	-0.356696	[redacted]
chr3	152217304	152217375	[redacted]	-0.356696	[redacted]
chr18	15379530	15379565	[redacted]	-0.356694	[redacted]
chrX	104554613	104554657	[redacted]	-0.356688	[redacted]
chr11	78972633	78972665	[redacted]	-0.356685	[redacted]
chr3	138326580	138326640	[redacted]	-0.356648	[redacted]
chr19	29377212	29377290	[redacted]	-0.356638	[redacted]
chr6	53915976	53916015	[redacted]	-0.356622	[redacted]
chr9	105062363	105062398	[redacted]	-0.356607	[redacted]
chr1	179815987	179816000	[redacted]	-0.356523	[redacted]
chr10	25060191	25060248	[redacted]	-0.356518	[redacted]
chr2	112592841	112592873	[redacted]	-0.356488	[redacted]
chr5	141222172	141222269	[redacted]	-0.356481	[redacted]
chr19	16243845	16243895	[redacted]	-0.356466	[redacted]
chr16	22359259	22359340	[redacted]	-0.356462	[redacted]
chr2	161841366	161841426	[redacted]	-0.356449	[redacted]
chr10	100008054	100008115	[redacted]	-0.356442	[redacted]
chr18	52707812	52707832	[redacted]	-0.356431	[redacted]
chr16	78321299	78321320	[redacted]	-0.35642	[redacted]
chr9	106396768	106396839	[redacted]	-0.356359	[redacted]
chr5	80391749	80391782	[redacted]	-0.356325	[redacted]
chr9	92554176	92554244	[redacted]	-0.356305	[redacted]
chr9	120927653	120927725	[redacted]	-0.35629	[redacted]
chr10	127878180	127878193	[redacted]	-0.356288	[redacted]
chr2	163313256	163313269	[redacted]	-0.356281	[redacted]
chr5	116076493	116076566	[redacted]	-0.356251	[redacted]

chr11	67368055	67368102	[redacted]	-0.356228	[redacted]
chr16	94972413	94972424	[redacted]	-0.356225	[redacted]
chr2	66167240	66167256	[redacted]	-0.356215	[redacted]
chr1	10150886	10150943	[redacted]	-0.356193	[redacted]
chr17	46558956	46558995	[redacted]	-0.356192	[redacted]
chr10	117036586	117036629	[redacted]	-0.35619	[redacted]
chr13	96828611	96828702	[redacted]	-0.356185	[redacted]
chr6	64077553	64077614	[redacted]	-0.356183	[redacted]
chr12	40341688	40341747	[redacted]	-0.356174	[redacted]
chr3	19017466	19017526	[redacted]	-0.356174	[redacted]
chr1	156501170	156501190	[redacted]	-0.356163	[redacted]
chr3	31081973	31082001	[redacted]	-0.356143	[redacted]
chr6	100811904	100811954	[redacted]	-0.356123	[redacted]
chr14	55401224	55401276	[redacted]	-0.356078	[redacted]
chr13	17691205	17691272	[redacted]	-0.356077	[redacted]
chr12	70109357	70109441	[redacted]	-0.356054	[redacted]
chr1	75702880	75702902	[redacted]	-0.356051	[redacted]
chr4	25006184	25006208	[redacted]	-0.356029	[redacted]
chr14	121905490	121905547	[redacted]	-0.356012	[redacted]
chr8	54423873	54423921	[redacted]	-0.356008	[redacted]
chr17	26859576	26859632	[redacted]	-0.35599	[redacted]
chr11	84752983	84753039	[redacted]	-0.355927	[redacted]
chr9	43837962	43838014	[redacted]	-0.355923	[redacted]
chr6	123099354	123099398	[redacted]	-0.355909	[redacted]
chr9	103016030	103016104	[redacted]	-0.355907	[redacted]
chr15	55286860	55286923	[redacted]	-0.355897	[redacted]
chr1	51418911	51418987	[redacted]	-0.355878	[redacted]
chr2	181805876	181805939	[redacted]	-0.355872	[redacted]
chr7	46744362	46744411	[redacted]	-0.355853	[redacted]
chr10	71400930	71400954	[redacted]	-0.355849	[redacted]
chr1	80121309	80121371	[redacted]	-0.355842	[redacted]
chr13	43140291	43140345	[redacted]	-0.355827	[redacted]
chr2	167199647	167199698	[redacted]	-0.355822	[redacted]
chr4	94989855	94989913	[redacted]	-0.355813	[redacted]
chr12	69217522	69217594	[redacted]	-0.355793	[redacted]
chrX	114997301	114997337	[redacted]	-0.355791	[redacted]
chr10	125447774	125447825	[redacted]	-0.355776	[redacted]
chr7	136926356	136926398	[redacted]	-0.355772	[redacted]
chr19	8972004	8972032	[redacted]	-0.355765	[redacted]
chr7	67131465	67131547	[redacted]	-0.355697	[redacted]
chr13	113064717	113064753	[redacted]	-0.355688	[redacted]
chr17	46353389	46353419	[redacted]	-0.355683	[redacted]
chr11	109107522	109107582	[redacted]	-0.355681	[redacted]

chr15	94949704	94949776	[redacted]	-0.355681	[redacted]
chr6	135461881	135461941	[redacted]	-0.355662	[redacted]
chr9	20246849	20246877	[redacted]	-0.35566	[redacted]
chr5	97594441	97594472	[redacted]	-0.355642	[redacted]
chr7	128410391	128410414	[redacted]	-0.355632	[redacted]
chr2	170208035	170208073	[redacted]	-0.355629	[redacted]
chr4	129917567	129917707	[redacted]	-0.355624	[redacted]
chr8	96115928	96115997	[redacted]	-0.355619	[redacted]
chr3	136171158	136171169	[redacted]	-0.355609	[redacted]
chr14	75203024	75203055	[redacted]	-0.355581	[redacted]
chr12	42059045	42059114	[redacted]	-0.355561	[redacted]
chr1	53610665	53610778	[redacted]	-0.355554	[redacted]
chr12	112088383	112088422	[redacted]	-0.355545	[redacted]
chr2	74912650	74912713	[redacted]	-0.355534	[redacted]
chr6	136200785	136200880	[redacted]	-0.355515	[redacted]
chr1	79734618	79734652	[redacted]	-0.355473	[redacted]
chr8	57515634	57515685	[redacted]	-0.355448	[redacted]
chr7	123002027	123002040	[redacted]	-0.355404	[redacted]
chr2	104504249	104504325	[redacted]	-0.355396	[redacted]
chrX	126600383	126600480	[redacted]	-0.355395	[redacted]
chr18	5606904	5606915	[redacted]	-0.35538	[redacted]
chr17	73218565	73218613	[redacted]	-0.35536	[redacted]
chr19	4754379	4754447	[redacted]	-0.355348	[redacted]
chr10	85267005	85267081	[redacted]	-0.355304	[redacted]
chr19	39491220	39491313	[redacted]	-0.35528	[redacted]
chr16	4013013	4013074	[redacted]	-0.355243	[redacted]
chr9	88394964	88395051	[redacted]	-0.355233	[redacted]
chr1	60287184	60287328	[redacted]	-0.355231	[redacted]
chr9	118667022	118667041	[redacted]	-0.355222	[redacted]
chr15	59755056	59755099	[redacted]	-0.355209	[redacted]
chr12	86784956	86785019	[redacted]	-0.355199	[redacted]
chr7	125862000	125862046	[redacted]	-0.355179	[redacted]
chr14	55023381	55023424	[redacted]	-0.355171	[redacted]
chr1_[illegible]	123912	123971	[redacted]	-0.355136	[redacted]
chr7	127055025	127055066	[redacted]	-0.35513	[redacted]
chr14	48943309	48943405	[redacted]	-0.355115	[redacted]
chr19	34485711	34485776	[redacted]	-0.355071	[redacted]
chr1	4666435	4666482	[redacted]	-0.355037	[redacted]
chr4	155622622	155622680	[redacted]	-0.355016	[redacted]
chr13	27320819	27320906	[redacted]	-0.354985	[redacted]
chr2	143468045	143468095	[redacted]	-0.354909	[redacted]
chr13	116323845	116323949	[redacted]	-0.354884	[redacted]
chr13	9965201	9965281	[redacted]	-0.354879	[redacted]

chr6	4438756	4438791	[redacted]	-0.354879	[redacted]
chr3	109074691	109074749	[redacted]	-0.354832	[redacted]
chr4	141990591	141990648	[redacted]	-0.354799	[redacted]
chr3	69232751	69232776	[redacted]	-0.35478	[redacted]
chr2	120984350	120984362	[redacted]	-0.354773	[redacted]
chr10	76990995	76991053	[redacted]	-0.354772	[redacted]
chr4	119696512	119696554	[redacted]	-0.354746	[redacted]
chr4	102970382	102970441	[redacted]	-0.354733	[redacted]
chr3	84156245	84156435	[redacted]	-0.354723	[redacted]
chr2	45023810	45023855	[redacted]	-0.354716	[redacted]
chr11	77849388	77849422	[redacted]	-0.354692	[redacted]
chr5	64601756	64601829	[redacted]	-0.354664	[redacted]
chr4	89505152	89505203	[redacted]	-0.354659	[redacted]
chr2	159059156	159059171	[redacted]	-0.354652	[redacted]
chr14	93380099	93380177	[redacted]	-0.35465	[redacted]
chr15	62645452	62645511	[redacted]	-0.354629	[redacted]
chr10	4940974	4941024	[redacted]	-0.354611	[redacted]
chr10	119099169	119099220	[redacted]	-0.354578	[redacted]
chr8	89727594	89727634	[redacted]	-0.354549	[redacted]
chr17	14006008	14006049	[redacted]	-0.354543	[redacted]
chr8	73239661	73239705	[redacted]	-0.354542	[redacted]
chr13	76559635	76559674	[redacted]	-0.354541	[redacted]
chr5	3233927	3233979	[redacted]	-0.354534	[redacted]
chr7	45360484	45360555	[redacted]	-0.354527	[redacted]
chr8	66874286	66874340	[redacted]	-0.354526	[redacted]
chr10	29066092	29066132	[redacted]	-0.354513	[redacted]
chr2	73096988	73097085	[redacted]	-0.354496	[redacted]
chr1	161397778	161397819	[redacted]	-0.354479	[redacted]
chr11	4953762	4953829	[redacted]	-0.354465	[redacted]
chr12	52012365	52012408	[redacted]	-0.354459	[redacted]
chr16	69563397	69563486	[redacted]	-0.354435	[redacted]
chr3	111827679	111827782	[redacted]	-0.354399	[redacted]
chr6	106603851	106603940	[redacted]	-0.354387	[redacted]
chr2	60447533	60447649	[redacted]	-0.35438	[redacted]
chr1	69219346	69219393	[redacted]	-0.354372	[redacted]
chr1	13466398	13466472	[redacted]	-0.354356	[redacted]
chr3	89907030	89907108	[redacted]	-0.354295	[redacted]
chr3	82332752	82332795	[redacted]	-0.354285	[redacted]
chr12	61823979	61824015	[redacted]	-0.35428	[redacted]
chr1	93328370	93328409	[redacted]	-0.354257	[redacted]
chr3	84748710	84748778	[redacted]	-0.354248	[redacted]
chr5	41628865	41628891	[redacted]	-0.354233	[redacted]
chr13	36792960	36793012	[redacted]	-0.354203	[redacted]

chr17	17425266	17425366	[redacted]	-0.354186	[redacted]
chr10	41097739	41097800	[redacted]	-0.354164	[redacted]
chr4	6268021	6268075	[redacted]	-0.35416	[redacted]
chr10	121646369	121646448	[redacted]	-0.354159	[redacted]
chr10	85104701	85104723	[redacted]	-0.354139	[redacted]
chr12	37284422	37284498	[redacted]	-0.35413	[redacted]
chr5	27223476	27223544	[redacted]	-0.354102	[redacted]
chr11	98482898	98482959	[redacted]	-0.354064	[redacted]
chr5	20510504	20510558	[redacted]	-0.354038	[redacted]
chr5	38431321	38431380	[redacted]	-0.354033	[redacted]
chr1	60204822	60204864	[redacted]	-0.354004	[redacted]
chr1	187245507	187245516	[redacted]	-0.354002	[redacted]
chr7	124369366	124369451	[redacted]	-0.353988	[redacted]
chr6	37317596	37317662	[redacted]	-0.353906	[redacted]
chr10	39481276	39481314	[redacted]	-0.353898	[redacted]
chr6	65425962	65426031	[redacted]	-0.353874	[redacted]
chr1	64912090	64912139	[redacted]	-0.353828	[redacted]
chr14	118935216	118935235	[redacted]	-0.353826	[redacted]
chr6	38965285	38965422	[redacted]	-0.353817	[redacted]
chr9	37047977	37047998	[redacted]	-0.353809	[redacted]
chr15	6776173	6776230	[redacted]	-0.353794	[redacted]
chr14	74591610	74591675	[redacted]	-0.353782	[redacted]
chr9	47041540	47041607	[redacted]	-0.35378	[redacted]
chr5	97417906	97418013	[redacted]	-0.35377	[redacted]
chr6	49789917	49789987	[redacted]	-0.353763	[redacted]
chr2	170438223	170438241	[redacted]	-0.35376	[redacted]
chr11	8092249	8092314	[redacted]	-0.353744	[redacted]
chr6	88516646	88516685	[redacted]	-0.353734	[redacted]
chr19	47733739	47733758	[redacted]	-0.353691	[redacted]
chr7	134638922	134639003	[redacted]	-0.353691	[redacted]
chr9	72044572	72044608	[redacted]	-0.353681	[redacted]
chr9	114074734	114074790	[redacted]	-0.353674	[redacted]
chr15	5354545	5354596	[redacted]	-0.353664	[redacted]
chr19	52071171	52071215	[redacted]	-0.353621	[redacted]
chr1	16373462	16373549	[redacted]	-0.353601	[redacted]
chr9	119467930	119467976	[redacted]	-0.353599	[redacted]
chr14	86742728	86742778	[redacted]	-0.353578	[redacted]
chr16	94395927	94396025	[redacted]	-0.353527	[redacted]
chr19	40510986	40511112	[redacted]	-0.353525	[redacted]
chr6	135098113	135098188	[redacted]	-0.353517	[redacted]
chr7	117224975	117224996	[redacted]	-0.353515	[redacted]
chr7	45251387	45251420	[redacted]	-0.353507	[redacted]
chr1	85607191	85607217	[redacted]	-0.353506	[redacted]

chr9	108417288	108417347	[redacted]	-0.353501	[redacted]
chr15	57380514	57380546	[redacted]	-0.353472	[redacted]
chr14	76009469	76009477	[redacted]	-0.353412	[redacted]
chr2	4131872	4131897	[redacted]	-0.353407	[redacted]
chr16	29135086	29135159	[redacted]	-0.353404	[redacted]
chr6	125100499	125100576	[redacted]	-0.353403	[redacted]
chr11	90016495	90016603	[redacted]	-0.353396	[redacted]
chr6	58620022	58620191	[redacted]	-0.35339	[redacted]
chr16	42450344	42450387	[redacted]	-0.353372	[redacted]
chr9	117165356	117165445	[redacted]	-0.353359	[redacted]
chr15	25789127	25789215	[redacted]	-0.353337	[redacted]
chr10	110899998	110900068	[redacted]	-0.353313	[redacted]
chr6	29508590	29508693	[redacted]	-0.353302	[redacted]
chr9	3018858	3018905	[redacted]	-0.353295	[redacted]
chr6	146929000	146929083	[redacted]	-0.353273	[redacted]
chr10	6814639	6814675	[redacted]	-0.353263	[redacted]
chr14	11183589	11183619	[redacted]	-0.353254	[redacted]
chr16	11062557	11062615	[redacted]	-0.353248	[redacted]
chr17	86466464	86466496	[redacted]	-0.353247	[redacted]
chr15	68385099	68385157	[redacted]	-0.353243	[redacted]
chr9	60867471	60867541	[redacted]	-0.353238	[redacted]
chr8	4491582	4491652	[redacted]	-0.353221	[redacted]
chr5	115065307	115065379	[redacted]	-0.353215	[redacted]
chr4	123715989	123716014	[redacted]	-0.353209	[redacted]
chr5	74239920	74239953	[redacted]	-0.3532	[redacted]
chr10	99298536	99298570	[redacted]	-0.35316	[redacted]
chr8	66622367	66622440	[redacted]	-0.353153	[redacted]
chr6	73205382	73205425	[redacted]	-0.353137	[redacted]
chr12	117403196	117403249	[redacted]	-0.353135	[redacted]
chr8	94570899	94570962	[redacted]	-0.353104	[redacted]
chr17	12522939	12522999	[redacted]	-0.353088	[redacted]
chr5	8210842	8210878	[redacted]	-0.353068	[redacted]
chr10	67522280	67522327	[redacted]	-0.353038	[redacted]
chr15	37249261	37249377	[redacted]	-0.353017	[redacted]
chr6	13749416	13749457	[redacted]	-0.353012	[redacted]
chr4	150266885	150266899	[redacted]	-0.352989	[redacted]
chr18	51940799	51940872	[redacted]	-0.352974	[redacted]
chr8	77068955	77069015	[redacted]	-0.352964	[redacted]
chr1	105126894	105127010	[redacted]	-0.35296	[redacted]
chr2	62455523	62455557	[redacted]	-0.352946	[redacted]
chr12	66754605	66754669	[redacted]	-0.352939	[redacted]
chr18	58392943	58393009	[redacted]	-0.352895	[redacted]
chr14	57305125	57305199	[redacted]	-0.352893	[redacted]

chr2	132160701	132160775	[redacted]	-0.352887	[redacted]
chr9	118481744	118481810	[redacted]	-0.35286	[redacted]
chr18	66757805	66757873	[redacted]	-0.352853	[redacted]
chr9	112783553	112783614	[redacted]	-0.352841	[redacted]
chr2	32582896	32582917	[redacted]	-0.352827	[redacted]
chr6	132414064	132414123	[redacted]	-0.352805	[redacted]
chr19	5074416	5074489	[redacted]	-0.352805	[redacted]
chr10	117909974	117910013	[redacted]	-0.352804	[redacted]
chr16	32928450	32928468	[redacted]	-0.352803	[redacted]
chr2	13980771	13980844	[redacted]	-0.352798	[redacted]
chr13	30890982	30891019	[redacted]	-0.352795	[redacted]
chr16	90710478	90710571	[redacted]	-0.352791	[redacted]
chr7	102632876	102632929	[redacted]	-0.352777	[redacted]
chr11	107841262	107841333	[redacted]	-0.352727	[redacted]
chr7	126148136	126148180	[redacted]	-0.35268	[redacted]
chr2	22876395	22876429	[redacted]	-0.352647	[redacted]
chr18	84821649	84821758	[redacted]	-0.352645	[redacted]
chr12	42297668	42297720	[redacted]	-0.352626	[redacted]
chr16	15702430	15702513	[redacted]	-0.352623	[redacted]
chr3	10242205	10242258	[redacted]	-0.352618	[redacted]
chr5	105961105	105961128	[redacted]	-0.352617	[redacted]
chr8	121716165	121716198	[redacted]	-0.352598	[redacted]
chr7	108080865	108080944	[redacted]	-0.352577	[redacted]
chr9	25054312	25054392	[redacted]	-0.352575	[redacted]
chr2	131445849	131445882	[redacted]	-0.352571	[redacted]
chr6	138223476	138223548	[redacted]	-0.35252	[redacted]
chr8	106604782	106604871	[redacted]	-0.35248	[redacted]
chr15	99600178	99600264	[redacted]	-0.352474	[redacted]
chr1	80130568	80130609	[redacted]	-0.352473	[redacted]
chr19	41297205	41297221	[redacted]	-0.352473	[redacted]
chr3	84675230	84675239	[redacted]	-0.352464	[redacted]
chr7	19911157	19911212	[redacted]	-0.352458	[redacted]
chr16	45734518	45734556	[redacted]	-0.352456	[redacted]
chr16	22470568	22470640	[redacted]	-0.352452	[redacted]
chr4	123527826	123527921	[redacted]	-0.352437	[redacted]
chr15	78924747	78924813	[redacted]	-0.352417	[redacted]
chr8	117459963	117460032	[redacted]	-0.352354	[redacted]
chr3	50522773	50522834	[redacted]	-0.352349	[redacted]
chr7	30497861	30497947	[redacted]	-0.352349	[redacted]
chr11	89428739	89428787	[redacted]	-0.352339	[redacted]
chr14	10984544	10984597	[redacted]	-0.352332	[redacted]
chr4	121000090	121000124	[redacted]	-0.35229	[redacted]
chr19	30011397	30011429	[redacted]	-0.352274	[redacted]

chr6	55089943	55090021	[redacted]	-0.352256	[redacted]
chr3	100734425	100734467	[redacted]	-0.35224	[redacted]
chr15	6770685	6770702	[redacted]	-0.352237	[redacted]
chr7	115476081	115476173	[redacted]	-0.352234	[redacted]
chr14	97572461	97572531	[redacted]	-0.352217	[redacted]
chr13	47334103	47334192	[redacted]	-0.352207	[redacted]
chr10	107490646	107490729	[redacted]	-0.352199	[redacted]
chrX	162967721	162967799	[redacted]	-0.352198	[redacted]
chr15	10074078	10074147	[redacted]	-0.352193	[redacted]
chr12	64011630	64011727	[redacted]	-0.352183	[redacted]
chr18	22763762	22763775	[redacted]	-0.352182	[redacted]
chr11	79908073	79908089	[redacted]	-0.352164	[redacted]
chr12	118866610	118866648	[redacted]	-0.35211	[redacted]
chr1	13577306	13577393	[redacted]	-0.352108	[redacted]
chr8	82873085	82873167	[redacted]	-0.352091	[redacted]
chr8	127470405	127470440	[redacted]	-0.35209	[redacted]
chr2	11406749	11406780	[redacted]	-0.352071	[redacted]
chr11	57957026	57957060	[redacted]	-0.352064	[redacted]
chr18	61897958	61897965	[redacted]	-0.352055	[redacted]
chr5	22515114	22515172	[redacted]	-0.352044	[redacted]
chr8	83287558	83287597	[redacted]	-0.352027	[redacted]
chrX	20645281	20645366	[redacted]	-0.352025	[redacted]
chr9	42605790	42605843	[redacted]	-0.352018	[redacted]
chr18	19656546	19656589	[redacted]	-0.351965	[redacted]
chr3	84043964	84043987	[redacted]	-0.351957	[redacted]
chr18	65953489	65953541	[redacted]	-0.351946	[redacted]
chr4	48543643	48543699	[redacted]	-0.351938	[redacted]
chr16	93513254	93513268	[redacted]	-0.351934	[redacted]
chr13	119206128	119206219	[redacted]	-0.3519	[redacted]
chr8	83773395	83773467	[redacted]	-0.351893	[redacted]
chr17	50097188	50097201	[redacted]	-0.351838	[redacted]
chr1	153451932	153451984	[redacted]	-0.351827	[redacted]
chr18	22534432	22534503	[redacted]	-0.351825	[redacted]
chr19	25280929	25280953	[redacted]	-0.351806	[redacted]
chr12	91275962	91275990	[redacted]	-0.351778	[redacted]
chr7	137901248	137901308	[redacted]	-0.351771	[redacted]
chr9	53744278	53744345	[redacted]	-0.351756	[redacted]
chr6	133709844	133709881	[redacted]	-0.351754	[redacted]
chr1	152718215	152718268	[redacted]	-0.351717	[redacted]
chr9	66664604	66664620	[redacted]	-0.351709	[redacted]
chr2	125254203	125254276	[redacted]	-0.351692	[redacted]
chr19	10957799	10957896	[redacted]	-0.351666	[redacted]
chr15	86145782	86145859	[redacted]	-0.351655	[redacted]

chr7	55790486	55790533	[redacted]	-0.351624	[redacted]
chr17	78088375	78088430	[redacted]	-0.351622	[redacted]
chr12	80360409	80360493	[redacted]	-0.35162	[redacted]
chr9	65037067	65037098	[redacted]	-0.351598	[redacted]
chr12	107348546	107348564	[redacted]	-0.351567	[redacted]
chr7	16292876	16292944	[redacted]	-0.351566	[redacted]
chr3	138735790	138735811	[redacted]	-0.351565	[redacted]
chr10	99106131	99106187	[redacted]	-0.351556	[redacted]
chr11	39442708	39442734	[redacted]	-0.351534	[redacted]
chr10	117433408	117433440	[redacted]	-0.351487	[redacted]
chr9	91235315	91235361	[redacted]	-0.35141	[redacted]
chr14	48198223	48198299	[redacted]	-0.351409	[redacted]
chr3	152923854	152923909	[redacted]	-0.351398	[redacted]
chr2	4981669	4981759	[redacted]	-0.351397	[redacted]
chr19	57021299	57021356	[redacted]	-0.351394	[redacted]
chr12	17677273	17677280	[redacted]	-0.351389	[redacted]
chr18	14680924	14680995	[redacted]	-0.351388	[redacted]
chr11	75425802	75425829	[redacted]	-0.351382	[redacted]
chr1	151379647	151379683	[redacted]	-0.351352	[redacted]
chr17	4729419	4729478	[redacted]	-0.351341	[redacted]
chr6	99274923	99274995	[redacted]	-0.351337	[redacted]
chr7	70026679	70026744	[redacted]	-0.351336	[redacted]
chr9	47702082	47702119	[redacted]	-0.351284	[redacted]
chr1	150426780	150426812	[redacted]	-0.351268	[redacted]
chr10	23209852	23209920	[redacted]	-0.351204	[redacted]
chr11	85851191	85851246	[redacted]	-0.351173	[redacted]
chr11	59699042	59699088	[redacted]	-0.351152	[redacted]
chr13	108542589	108542634	[redacted]	-0.351146	[redacted]
chrX	89076338	89076397	[redacted]	-0.351142	[redacted]
chr11	69583418	69583537	[redacted]	-0.351119	[redacted]
chr17	89232591	89232635	[redacted]	-0.351114	[redacted]
chr11	39438683	39438758	[redacted]	-0.351112	[redacted]
chr8	19713434	19713496	[redacted]	-0.351097	[redacted]
chr13	77530615	77530673	[redacted]	-0.351066	[redacted]
chr17	78889143	78889216	[redacted]	-0.351059	[redacted]
chr8	117860620	117860747	[redacted]	-0.351049	[redacted]
chr9	46255673	46255768	[redacted]	-0.351044	[redacted]
chrX	103185737	103185750	[redacted]	-0.35104	[redacted]
chr6	143534086	143534137	[redacted]	-0.351038	[redacted]
chr17	23545660	23545681	[redacted]	-0.351033	[redacted]
chr6	108611378	108611469	[redacted]	-0.351026	[redacted]
chr17	3199707	3199766	[redacted]	-0.351005	[redacted]
chr5	45620981	45621034	[redacted]	-0.351005	[redacted]

chr7	30870978	30871045	[redacted]	-0.350983	[redacted]
chr12	81320774	81320809	[redacted]	-0.350975	[redacted]
chr9	104503556	104503640	[redacted]	-0.35094	[redacted]
chr10	22605919	22605958	[redacted]	-0.350915	[redacted]
chr17	8691512	8691585	[redacted]	-0.350905	[redacted]
chr9	28872060	28872122	[redacted]	-0.350889	[redacted]
chr12	91570851	91570875	[redacted]	-0.350887	[redacted]
chr8	93452156	93452222	[redacted]	-0.350854	[redacted]
chr12	96728347	96728380	[redacted]	-0.350782	[redacted]
chr7	117444212	117444341	[redacted]	-0.350781	[redacted]
chr1	72584353	72584410	[redacted]	-0.350781	[redacted]
chr15	82187809	82187895	[redacted]	-0.350777	[redacted]
chr12	54694636	54694729	[redacted]	-0.350769	[redacted]
chr9	4714994	4715056	[redacted]	-0.350768	[redacted]
chr5	142392973	142393006	[redacted]	-0.350759	[redacted]
chr17	47849101	47849296	[redacted]	-0.350754	[redacted]
chr1	164079754	164079873	[redacted]	-0.350748	[redacted]
chr17	64571286	64571366	[redacted]	-0.35073	[redacted]
chr15	6324571	6324676	[redacted]	-0.350726	[redacted]
chr13	28772624	28772664	[redacted]	-0.35072	[redacted]
chr6	118535818	118535904	[redacted]	-0.350706	[redacted]
chr4	35398589	35398629	[redacted]	-0.350705	[redacted]
chr16	72453403	72453467	[redacted]	-0.350698	[redacted]
chr9	85750930	85750985	[redacted]	-0.350693	[redacted]
chr5	129752145	129752151	[redacted]	-0.350691	[redacted]
chr1	11019748	11019802	[redacted]	-0.350673	[redacted]
chrX	101137382	101137459	[redacted]	-0.350672	[redacted]
chr10	91238237	91238273	[redacted]	-0.350649	[redacted]
chr6	142855155	142855207	[redacted]	-0.350633	[redacted]
chr3	10338235	10338309	[redacted]	-0.350623	[redacted]
chr17	32577640	32577655	[redacted]	-0.350598	[redacted]
chr2	149939264	149939299	[redacted]	-0.350586	[redacted]
chr17	79774788	79774843	[redacted]	-0.350583	[redacted]
chr14	20584844	20584910	[redacted]	-0.35058	[redacted]
chr2	80310222	80310295	[redacted]	-0.350577	[redacted]
chr8	84736111	84736151	[redacted]	-0.350553	[redacted]
chr13	119759154	119759209	[redacted]	-0.350544	[redacted]
chr6	53370564	53370608	[redacted]	-0.350476	[redacted]
chrX	52444274	52444319	[redacted]	-0.350473	[redacted]
chr17	35175764	35175795	[redacted]	-0.350456	[redacted]
chr17	27540665	27540738	[redacted]	-0.35045	[redacted]
chr15	90082728	90082782	[redacted]	-0.35045	[redacted]
chr10	78964952	78965001	[redacted]	-0.350449	[redacted]

chr11	107521659	107521741	[redacted]	-0.350446	[redacted]
chr9	92541424	92541519	[redacted]	-0.350419	[redacted]
chr4	101071571	101071651	[redacted]	-0.350404	[redacted]
chr19	29938417	29938514	[redacted]	-0.350403	[redacted]
chr9	88390524	88390580	[redacted]	-0.350385	[redacted]
chr12	109775293	109775358	[redacted]	-0.350376	[redacted]
chr5	96242008	96242057	[redacted]	-0.350371	[redacted]
chr2	144079672	144079708	[redacted]	-0.350369	[redacted]
chr13	22082830	22082896	[redacted]	-0.350368	[redacted]
chr15	102851714	102851772	[redacted]	-0.350348	[redacted]
chr11	19105019	19105057	[redacted]	-0.350344	[redacted]
chr17	33522153	33522231	[redacted]	-0.350335	[redacted]
chr6	30614950	30614979	[redacted]	-0.350317	[redacted]
chr13	42212434	42212488	[redacted]	-0.350277	[redacted]
chr16	94185283	94185336	[redacted]	-0.350271	[redacted]
chr1	154876617	154876671	[redacted]	-0.350271	[redacted]
chr12	39217379	39217425	[redacted]	-0.35025	[redacted]
chr7	92029883	92029927	[redacted]	-0.350238	[redacted]
chr11	67355015	67355045	[redacted]	-0.350194	[redacted]
chr5	137108196	137108242	[redacted]	-0.350172	[redacted]
chr15	10732826	10732902	[redacted]	-0.350162	[redacted]
chr16	21353928	21353991	[redacted]	-0.350161	[redacted]
chr2	158026513	158026565	[redacted]	-0.350146	[redacted]
chr5	72159594	72159656	[redacted]	-0.350127	[redacted]
chr12	71901237	71901254	[redacted]	-0.350101	[redacted]
chr12	110684810	110684864	[redacted]	-0.350087	[redacted]
chr11	83353875	83353913	[redacted]	-0.350069	[redacted]
chr3	96959151	96959192	[redacted]	-0.350058	[redacted]
chr1	152521941	152521968	[redacted]	-0.350031	[redacted]
chr2	155335653	155335738	[redacted]	-0.350024	[redacted]
chr4	62589885	62589903	[redacted]	-0.35001	[redacted]
chr7	110699538	110699594	[redacted]	-0.349988	[redacted]
chr9	89148405	89148509	[redacted]	-0.349986	[redacted]
chr12	112306781	112306852	[redacted]	-0.349966	[redacted]
chr15	90855118	90855174	[redacted]	-0.349964	[redacted]
chr6	8735668	8735754	[redacted]	-0.349934	[redacted]
chr17	67955530	67955599	[redacted]	-0.349927	[redacted]
chr17	11442537	11442609	[redacted]	-0.349869	[redacted]
chr2	75962878	75962917	[redacted]	-0.349868	[redacted]
chr6	128828923	128828985	[redacted]	-0.349854	[redacted]
chr8	84326553	84326586	[redacted]	-0.349833	[redacted]
chr14	24978095	24978147	[redacted]	-0.349811	[redacted]
chr3	9133687	9133720	[redacted]	-0.349807	[redacted]

chr12	82523058	82523124	[redacted]	-0.349798	[redacted]
chr14	36548018	36548065	[redacted]	-0.349797	[redacted]
chr3	126629725	126629785	[redacted]	-0.349775	[redacted]
chr3	98366400	98366446	[redacted]	-0.34977	[redacted]
chr9	17677264	17677324	[redacted]	-0.349766	[redacted]
chr7	43673722	43673738	[redacted]	-0.349761	[redacted]
chr7	37808849	37808903	[redacted]	-0.349754	[redacted]
chr13	53594829	53594911	[redacted]	-0.349747	[redacted]
chr2	32823512	32823545	[redacted]	-0.349746	[redacted]
chr1	38485496	38485588	[redacted]	-0.349734	[redacted]
chr18	9187465	9187540	[redacted]	-0.349727	[redacted]
chr9	122506358	122506383	[redacted]	-0.349726	[redacted]
chr2	168587253	168587374	[redacted]	-0.349717	[redacted]
chr15	11248718	11248765	[redacted]	-0.349687	[redacted]
chr8	109132742	109132839	[redacted]	-0.349664	[redacted]
chr1	132155054	132155137	[redacted]	-0.349662	[redacted]
chr13	94293244	94293358	[redacted]	-0.34963	[redacted]
chr19	55382288	55382361	[redacted]	-0.349623	[redacted]
chr9	79286132	79286242	[redacted]	-0.349585	[redacted]
chr2	79437415	79437468	[redacted]	-0.349581	[redacted]
chr19	11367124	11367168	[redacted]	-0.349572	[redacted]
chr8	4100573	4100609	[redacted]	-0.349567	[redacted]
chr14	101703729	101703766	[redacted]	-0.349549	[redacted]
chr9	116744686	116744766	[redacted]	-0.349536	[redacted]
chr16	51759134	51759259	[redacted]	-0.349527	[redacted]
chr5	114905511	114905628	[redacted]	-0.349519	[redacted]
chr2	19839699	19839761	[redacted]	-0.349512	[redacted]
chr11	74594089	74594167	[redacted]	-0.349508	[redacted]
chr15	85198784	85198866	[redacted]	-0.3495	[redacted]
chr3	127644968	127645014	[redacted]	-0.349487	[redacted]
chr5	65876694	65876738	[redacted]	-0.349482	[redacted]
chr6	35195258	35195307	[redacted]	-0.349476	[redacted]
chr15	81383517	81383549	[redacted]	-0.349418	[redacted]
chr18	7170737	7170830	[redacted]	-0.349413	[redacted]
chr5	70051606	70051661	[redacted]	-0.349404	[redacted]
chr1	32859179	32859235	[redacted]	-0.349398	[redacted]
chr11	80053902	80053962	[redacted]	-0.349396	[redacted]
chr11	108232895	108232950	[redacted]	-0.349396	[redacted]
chr13	95170688	95170721	[redacted]	-0.349387	[redacted]
chr4	6438634	6438697	[redacted]	-0.349364	[redacted]
chr11	120196818	120196859	[redacted]	-0.349325	[redacted]
chr1	105538703	105538749	[redacted]	-0.349322	[redacted]
chr5	143910819	143910884	[redacted]	-0.349288	[redacted]

chr10	41599955	41600014	[redacted]	-0.349257	[redacted]
chr15	89688564	89688609	[redacted]	-0.349241	[redacted]
chr5	137690508	137690556	[redacted]	-0.349214	[redacted]
chr10	39924076	39924165	[redacted]	-0.349202	[redacted]
chr5	34363015	34363115	[redacted]	-0.349199	[redacted]
chr8	51531803	51531860	[redacted]	-0.349195	[redacted]
chr3	105077233	105077312	[redacted]	-0.349186	[redacted]
chr10	107542870	107542923	[redacted]	-0.349163	[redacted]
chr2	68759067	68759117	[redacted]	-0.349156	[redacted]
chr7	78229502	78229532	[redacted]	-0.349155	[redacted]
chr17	14020916	14021050	[redacted]	-0.349143	[redacted]
chr2	69664685	69664758	[redacted]	-0.349119	[redacted]
chr11	19172363	19172427	[redacted]	-0.349113	[redacted]
chr11	90239795	90239840	[redacted]	-0.349087	[redacted]
chr7	138258418	138258464	[redacted]	-0.349078	[redacted]
chr18	26663628	26663709	[redacted]	-0.349043	[redacted]
chr3	31887816	31887841	[redacted]	-0.349032	[redacted]
chr19	41064248	41064300	[redacted]	-0.349024	[redacted]
chr16	18529892	18529938	[redacted]	-0.349018	[redacted]
chr14	25024089	25024137	[redacted]	-0.349006	[redacted]
chr4	13919671	13919727	[redacted]	-0.349005	[redacted]
chr5	92343222	92343280	[redacted]	-0.348855	[redacted]
chr6	94881202	94881344	[redacted]	-0.348836	[redacted]
chr16	94748297	94748343	[redacted]	-0.348829	[redacted]
chr16	50608271	50608318	[redacted]	-0.348822	[redacted]
chr7	55798911	55798972	[redacted]	-0.348815	[redacted]
chr19	38303074	38303151	[redacted]	-0.348796	[redacted]
chr8	4594332	4594377	[redacted]	-0.348772	[redacted]
chr7	118309732	118309785	[redacted]	-0.348751	[redacted]
chr4	82298446	82298527	[redacted]	-0.348739	[redacted]
chr3	10686522	10686663	[redacted]	-0.348719	[redacted]
chr11	26439454	26439484	[redacted]	-0.348706	[redacted]
chr8	122173705	122173737	[redacted]	-0.348687	[redacted]
chr14	86643561	86643607	[redacted]	-0.348685	[redacted]
chr5	14149128	14149210	[redacted]	-0.348677	[redacted]
chr18	80078493	80078563	[redacted]	-0.348659	[redacted]
chr3	112768416	112768509	[redacted]	-0.348658	[redacted]
chrX	101844877	101844924	[redacted]	-0.348645	[redacted]
chr13	35053505	35053540	[redacted]	-0.348606	[redacted]
chr12	76201989	76202075	[redacted]	-0.348601	[redacted]
chr1	178153133	178153159	[redacted]	-0.348599	[redacted]
chr10	116330975	116330984	[redacted]	-0.348599	[redacted]
chr2	35557686	35557807	[redacted]	-0.348581	[redacted]

chr11	106252127	106252223	[redacted]	-0.348569	[redacted]
chr1	82972489	82972534	[redacted]	-0.348568	[redacted]
chr14	17621807	17621858	[redacted]	-0.348516	[redacted]
chr12	100165712	100165771	[redacted]	-0.348493	[redacted]
chr5	92866170	92866209	[redacted]	-0.348485	[redacted]
chr13	81418571	81418644	[redacted]	-0.348476	[redacted]
chr4	3895427	3895483	[redacted]	-0.348476	[redacted]
chr5	77532132	77532153	[redacted]	-0.34845	[redacted]
chr10	42399076	42399114	[redacted]	-0.348434	[redacted]
chr2	77434326	77434405	[redacted]	-0.348418	[redacted]
chr17	71738373	71738457	[redacted]	-0.348401	[redacted]
chr6	35328274	35328332	[redacted]	-0.348378	[redacted]
chr8	35991961	35991996	[redacted]	-0.348358	[redacted]
chr11	19764641	19764715	[redacted]	-0.348342	[redacted]
chr8	7030497	7030551	[redacted]	-0.348328	[redacted]
chr7	136656655	136656728	[redacted]	-0.348245	[redacted]
chr3	103607716	103607746	[redacted]	-0.348234	[redacted]
chr11	31594467	31594524	[redacted]	-0.348232	[redacted]
chr12	55566766	55566801	[redacted]	-0.348202	[redacted]
chr13	107832546	107832628	[redacted]	-0.348199	[redacted]
chr18	3667972	3668054	[redacted]	-0.348168	[redacted]
chrX	142196507	142196552	[redacted]	-0.348148	[redacted]
chr13	13932846	13932926	[redacted]	-0.348146	[redacted]
chr3	27510162	27510220	[redacted]	-0.34812	[redacted]
chr3	149981263	149981348	[redacted]	-0.348106	[redacted]
chr9	105872149	105872226	[redacted]	-0.348103	[redacted]
chr16	90632678	90632710	[redacted]	-0.348098	[redacted]
chr17	33032262	33032357	[redacted]	-0.348071	[redacted]
chr15	101728764	101728830	[redacted]	-0.348031	[redacted]
chr18	31788578	31788644	[redacted]	-0.348022	[redacted]
chr3	9065755	9065794	[redacted]	-0.347974	[redacted]
chr2	5047588	5047643	[redacted]	-0.347955	[redacted]
chr2	59814801	59814839	[redacted]	-0.347949	[redacted]
chr8	84238417	84238457	[redacted]	-0.347884	[redacted]
chr1	153346940	153346962	[redacted]	-0.347878	[redacted]
chr16	63879298	63879363	[redacted]	-0.347847	[redacted]
chr2	162579822	162579883	[redacted]	-0.347845	[redacted]
chr11	74299012	74299059	[redacted]	-0.347829	[redacted]
chr4	111871346	111871402	[redacted]	-0.347813	[redacted]
chr4	31914786	31914852	[redacted]	-0.347773	[redacted]
chr14	119131544	119131630	[redacted]	-0.347764	[redacted]
chr3	132988837	132988897	[redacted]	-0.347724	[redacted]
chr2	25219337	25219388	[redacted]	-0.347705	[redacted]

chr17	27009977	27010050	[redacted]	-0.347699	[redacted]
chr1	189932418	189932449	[redacted]	-0.347694	[redacted]
chr17	26145649	26145666	[redacted]	-0.347661	[redacted]
chr14	30764843	30764922	[redacted]	-0.347658	[redacted]
chr4	107258628	107258694	[redacted]	-0.347651	[redacted]
chr1	144096550	144096634	[redacted]	-0.347631	[redacted]
chr6	37121118	37121192	[redacted]	-0.347619	[redacted]
chr11	12400915	12400982	[redacted]	-0.347612	[redacted]
chr3	129131290	129131350	[redacted]	-0.347609	[redacted]
chr1	176898580	176898634	[redacted]	-0.347596	[redacted]
chr19	16835018	16835078	[redacted]	-0.347594	[redacted]
chr10	42868925	42869050	[redacted]	-0.347592	[redacted]
chr3	135361609	135361718	[redacted]	-0.347589	[redacted]
chr14	54682586	54682611	[redacted]	-0.347589	[redacted]
chr6	84355504	84355569	[redacted]	-0.34754	[redacted]
chr2	50706152	50706177	[redacted]	-0.347535	[redacted]
chr7	82488762	82488844	[redacted]	-0.34753	[redacted]
chr18	49710595	49710683	[redacted]	-0.347528	[redacted]
chr13	45910445	45910478	[redacted]	-0.347528	[redacted]
chr7	96971874	96971902	[redacted]	-0.347523	[redacted]
chr4	70329690	70329739	[redacted]	-0.347507	[redacted]
chr12	84587094	84587189	[redacted]	-0.347496	[redacted]
chr16	9415332	9415406	[redacted]	-0.347492	[redacted]
chr3	32344911	32344957	[redacted]	-0.347475	[redacted]
chr17	3053626	3053680	[redacted]	-0.347458	[redacted]
chr2	35021555	35021578	[redacted]	-0.347437	[redacted]
chrX	12681320	12681376	[redacted]	-0.347411	[redacted]
chr9	97002668	97002708	[redacted]	-0.347403	[redacted]
chr13	115416232	115416251	[redacted]	-0.347401	[redacted]
chr16	62903980	62904069	[redacted]	-0.347395	[redacted]
chr2	118240402	118240477	[redacted]	-0.347393	[redacted]
chr1	151538985	151539045	[redacted]	-0.347389	[redacted]
chr3	116100799	116100859	[redacted]	-0.347374	[redacted]
chr2	152666387	152666476	[redacted]	-0.347324	[redacted]
chr2	158973209	158973282	[redacted]	-0.347299	[redacted]
chr18	73793503	73793551	[redacted]	-0.347287	[redacted]
chr8	29156334	29156386	[redacted]	-0.347257	[redacted]
chr4	150396950	150396990	[redacted]	-0.347247	[redacted]
chr5	73211688	73211737	[redacted]	-0.347242	[redacted]
chr18	34681559	34681720	[redacted]	-0.347217	[redacted]
chr15	44627275	44627317	[redacted]	-0.347207	[redacted]
chr11	50701472	50701500	[redacted]	-0.347189	[redacted]
chr9	47765659	47765779	[redacted]	-0.347154	[redacted]

chr15	78920159	78920232	[redacted]	-0.34715	[redacted]
chr4	141567678	141567741	[redacted]	-0.347147	[redacted]
chr15	102270779	102270830	[redacted]	-0.347146	[redacted]
chr10	113974151	113974272	[redacted]	-0.34713	[redacted]
chr1	73028822	73028900	[redacted]	-0.347126	[redacted]
chr12	54223273	54223326	[redacted]	-0.347082	[redacted]
chr13	107909755	107909839	[redacted]	-0.347038	[redacted]
chr3	154746153	154746237	[redacted]	-0.347006	[redacted]
chr10	77683555	77683616	[redacted]	-0.346995	[redacted]
chr15	79012798	79012853	[redacted]	-0.346986	[redacted]
chr11	83567344	83567361	[redacted]	-0.346968	[redacted]
chr9	52373655	52373698	[redacted]	-0.346958	[redacted]
chr11	74621401	74621460	[redacted]	-0.346948	[redacted]
chr2	103996908	103996973	[redacted]	-0.34694	[redacted]
chr17	35348678	35348752	[redacted]	-0.346936	[redacted]
chr2	61150283	61150332	[redacted]	-0.346927	[redacted]
chr13	7869719	7869792	[redacted]	-0.346913	[redacted]
chr15	48867688	48867782	[redacted]	-0.346911	[redacted]
chr10	111880049	111880120	[redacted]	-0.346911	[redacted]
chr17	34113672	34113772	[redacted]	-0.346894	[redacted]
chr5	92921350	92921446	[redacted]	-0.346893	[redacted]
chr4	12761926	12761937	[redacted]	-0.346861	[redacted]
chr14	27379856	27379918	[redacted]	-0.34685	[redacted]
chr8	89026788	89026835	[redacted]	-0.346849	[redacted]
chr13	64506892	64506954	[redacted]	-0.346827	[redacted]
chr11	89427942	89428029	[redacted]	-0.346826	[redacted]
chr2	132283374	132283402	[redacted]	-0.346785	[redacted]
chr14	84974854	84974917	[redacted]	-0.346777	[redacted]
chr14	12211053	12211154	[redacted]	-0.34677	[redacted]
chr16	91653371	91653441	[redacted]	-0.346766	[redacted]
chr7	89314186	89314252	[redacted]	-0.346766	[redacted]
chr11	52906465	52906559	[redacted]	-0.346752	[redacted]
chr7	83023505	83023559	[redacted]	-0.346751	[redacted]
chr5	111012023	111012082	[redacted]	-0.346749	[redacted]
chr3	152247861	152248031	[redacted]	-0.346746	[redacted]
chr10	127880897	127881113	[redacted]	-0.346717	[redacted]
chr10	53727525	53727574	[redacted]	-0.346705	[redacted]
chr6	145202618	145202684	[redacted]	-0.346704	[redacted]
chr4	40025336	40025404	[redacted]	-0.346682	[redacted]
chr8	112551945	112552003	[redacted]	-0.346659	[redacted]
chr4	58708494	58708561	[redacted]	-0.346651	[redacted]
chr17	17403147	17403219	[redacted]	-0.346648	[redacted]
chr7	27134222	27134266	[redacted]	-0.346636	[redacted]

chr18	29223651	29223698	[redacted]	-0.346635	[redacted]
chr2	104065876	104065929	[redacted]	-0.346602	[redacted]
chr9	122293117	122293158	[redacted]	-0.346568	[redacted]
chr9	72666176	72666213	[redacted]	-0.346555	[redacted]
chr17	5898257	5898305	[redacted]	-0.346536	[redacted]
chr7	25564110	25564197	[redacted]	-0.346476	[redacted]
chr10	62648012	62648054	[redacted]	-0.346441	[redacted]
chr7	117717164	117717219	[redacted]	-0.34642	[redacted]
chr2	170051041	170051174	[redacted]	-0.346419	[redacted]
chr11	67874444	67874516	[redacted]	-0.346412	[redacted]
chr15	76386167	76386198	[redacted]	-0.346409	[redacted]
chr8	13929359	13929396	[redacted]	-0.346406	[redacted]
chr14	114909971	114910005	[redacted]	-0.346383	[redacted]
chr3	121880071	121880158	[redacted]	-0.346379	[redacted]
chrX	94408831	94408905	[redacted]	-0.346359	[redacted]
chr5	145439459	145439524	[redacted]	-0.346353	[redacted]
chr1	52697246	52697323	[redacted]	-0.346327	[redacted]
chr4	151073170	151073237	[redacted]	-0.346305	[redacted]
chr19	25826005	25826064	[redacted]	-0.346297	[redacted]
chr11	117424808	117424870	[redacted]	-0.346292	[redacted]
chr17	28736982	28737043	[redacted]	-0.346286	[redacted]
chr3	53815914	53815975	[redacted]	-0.346252	[redacted]
chr12	87174789	87174851	[redacted]	-0.346245	[redacted]
chr6	139454101	139454155	[redacted]	-0.346232	[redacted]
chr11	88690435	88690471	[redacted]	-0.346226	[redacted]
chr19	21490922	21490939	[redacted]	-0.346155	[redacted]
chr16	24527428	24527442	[redacted]	-0.346153	[redacted]
chr19	53884938	53884954	[redacted]	-0.346143	[redacted]
chr7	27778870	27778903	[redacted]	-0.346136	[redacted]
chr4	102344193	102344254	[redacted]	-0.346115	[redacted]
chrX	74764856	74764874	[redacted]	-0.346109	[redacted]
chr10	114949510	114949581	[redacted]	-0.346106	[redacted]
chr1	51196554	51196599	[redacted]	-0.34608	[redacted]
chr11	86271906	86271989	[redacted]	-0.346075	[redacted]
chr8	96124797	96124839	[redacted]	-0.346068	[redacted]
chr18	56472058	56472092	[redacted]	-0.346066	[redacted]
chr1	47150402	47150480	[redacted]	-0.346063	[redacted]
chr2	162073569	162073621	[redacted]	-0.346057	[redacted]
chr2	10024539	10024573	[redacted]	-0.346047	[redacted]
chr15	59268359	59268417	[redacted]	-0.346045	[redacted]
chr10	13418431	13418440	[redacted]	-0.346023	[redacted]
chr16	77503028	77503083	[redacted]	-0.346021	[redacted]
chr8	64625279	64625377	[redacted]	-0.346014	[redacted]

chr1	57003978	57004028	[redacted]	-0.345989	[redacted]
chr5	108195212	108195306	[redacted]	-0.345987	[redacted]
chr5	137281771	137281870	[redacted]	-0.345986	[redacted]
chr10	62006036	62006087	[redacted]	-0.345983	[redacted]
chr11	39112695	39112763	[redacted]	-0.345966	[redacted]
chr2	44663705	44663773	[redacted]	-0.345955	[redacted]
chr11	63412172	63412286	[redacted]	-0.345928	[redacted]
chr11	6685542	6685689	[redacted]	-0.345891	[redacted]
chr7	28201770	28201832	[redacted]	-0.345856	[redacted]
chr7	58786472	58786511	[redacted]	-0.34584	[redacted]
chr8	96485941	96485948	[redacted]	-0.345829	[redacted]
chr11	87505794	87505830	[redacted]	-0.345821	[redacted]
chr6	127475042	127475089	[redacted]	-0.345776	[redacted]
chr11	94249901	94249971	[redacted]	-0.345741	[redacted]
chr10	32486311	32486366	[redacted]	-0.345738	[redacted]
chrX	43958822	43958883	[redacted]	-0.345729	[redacted]
chr5	78287671	78287708	[redacted]	-0.345712	[redacted]
chr17	72650679	72650693	[redacted]	-0.34571	[redacted]
chr6	67205258	67205347	[redacted]	-0.345705	[redacted]
chr3	130087293	130087348	[redacted]	-0.345699	[redacted]
chr1	158378069	158378125	[redacted]	-0.345696	[redacted]
chrX	100477024	100477062	[redacted]	-0.345692	[redacted]
chr5	72202412	72202443	[redacted]	-0.345652	[redacted]
chr17	33239538	33239564	[redacted]	-0.345635	[redacted]
chr10	109874086	109874158	[redacted]	-0.345602	[redacted]
chr4	6030241	6030285	[redacted]	-0.345585	[redacted]
chr14	34393642	34393704	[redacted]	-0.345573	[redacted]
chr10	127200140	127200195	[redacted]	-0.345554	[redacted]
chr9	31638349	31638416	[redacted]	-0.345537	[redacted]
chr2	104212214	104212267	[redacted]	-0.345511	[redacted]
chr3	144557252	144557335	[redacted]	-0.345501	[redacted]
chr3	58821694	58821749	[redacted]	-0.34548	[redacted]
chr10	128340001	128340042	[redacted]	-0.345472	[redacted]
chr2	70725259	70725337	[redacted]	-0.345451	[redacted]
chr18	48864046	48864058	[redacted]	-0.345401	[redacted]
chr6	8328584	8328666	[redacted]	-0.345399	[redacted]
chr9	23682925	23682984	[redacted]	-0.345397	[redacted]
chrX	163940988	163941024	[redacted]	-0.345384	[redacted]
chr3	86724354	86724388	[redacted]	-0.345364	[redacted]
chr3	60614690	60614719	[redacted]	-0.34536	[redacted]
chr5	4484623	4484702	[redacted]	-0.345311	[redacted]
chr8	41778490	41778496	[redacted]	-0.345305	[redacted]
chr9	46144450	46144509	[redacted]	-0.345304	[redacted]

chr5	23618947	23618976	[redacted]	-0.345295	[redacted]
chr11	62713219	62713249	[redacted]	-0.345287	[redacted]
chr19	54289994	54290045	[redacted]	-0.345276	[redacted]
chr14	119929227	119929287	[redacted]	-0.345275	[redacted]
chr13	113912073	113912168	[redacted]	-0.345274	[redacted]
chr12	3102703	3102819	[redacted]	-0.345266	[redacted]
chr17	83544810	83544896	[redacted]	-0.345263	[redacted]
chr16	92679392	92679445	[redacted]	-0.345239	[redacted]
chr17	89460509	89460569	[redacted]	-0.345226	[redacted]
chr14	63114332	63114386	[redacted]	-0.345212	[redacted]
chr13	49678270	49678336	[redacted]	-0.345138	[redacted]
chr8	10090440	10090518	[redacted]	-0.345127	[redacted]
chr4	119209155	119209178	[redacted]	-0.345115	[redacted]
chr9	78777090	78777117	[redacted]	-0.345084	[redacted]
chr10	111763293	111763342	[redacted]	-0.345071	[redacted]
chr9	60876752	60876780	[redacted]	-0.345064	[redacted]
chrX	7558355	7558415	[redacted]	-0.345061	[redacted]
chr16	11582800	11582845	[redacted]	-0.34506	[redacted]
chr5	85959792	85959886	[redacted]	-0.345033	[redacted]
chr8	7749883	7749938	[redacted]	-0.345007	[redacted]
chr17	32527356	32527366	[redacted]	-0.344991	[redacted]
chr7	44362921	44362971	[redacted]	-0.34499	[redacted]
chr1	38424995	38425045	[redacted]	-0.344977	[redacted]
chr1	146819147	146819170	[redacted]	-0.344963	[redacted]
chr1	97880839	97880904	[redacted]	-0.344956	[redacted]
chr9	65452311	65452352	[redacted]	-0.344955	[redacted]
chr11	112853823	112853919	[redacted]	-0.344949	[redacted]
chr16	8262458	8262491	[redacted]	-0.344934	[redacted]
chr11	108070227	108070268	[redacted]	-0.34493	[redacted]
chr11	101543625	101543705	[redacted]	-0.344926	[redacted]
chr9	105729062	105729102	[redacted]	-0.34491	[redacted]
chr3	51249582	51249602	[redacted]	-0.344886	[redacted]
chr12	8953797	8953881	[redacted]	-0.34486	[redacted]
chr8	91397152	91397213	[redacted]	-0.344858	[redacted]
chr11	119014649	119014726	[redacted]	-0.344852	[redacted]
chr16	50703206	50703256	[redacted]	-0.344838	[redacted]
chr2	114695041	114695099	[redacted]	-0.344825	[redacted]
chrX	9105963	9106022	[redacted]	-0.34482	[redacted]
chr3	54828061	54828137	[redacted]	-0.344813	[redacted]
chr1	154789599	154789652	[redacted]	-0.344808	[redacted]
chr11	120975929	120975998	[redacted]	-0.344808	[redacted]
chr4	138330912	138330995	[redacted]	-0.344796	[redacted]
chr11	53783375	53783464	[redacted]	-0.344794	[redacted]

chrX	38148647	38148700	[redacted]	-0.344792	[redacted]
chr19	43517820	43517863	[redacted]	-0.344787	[redacted]
chr6	15906897	15906945	[redacted]	-0.34476	[redacted]
chrX	24401907	24401965	[redacted]	-0.344712	[redacted]
chr3	38728525	38728602	[redacted]	-0.344706	[redacted]
chr10	97245417	97245447	[redacted]	-0.344666	[redacted]
chr2	115225736	115225786	[redacted]	-0.344653	[redacted]
chr10	32113289	32113356	[redacted]	-0.34462	[redacted]
chr16	57190454	57190558	[redacted]	-0.344597	[redacted]
chr15	21070188	21070230	[redacted]	-0.344589	[redacted]
chr16	49665094	49665157	[redacted]	-0.344559	[redacted]
chr4	130073866	130073881	[redacted]	-0.344557	[redacted]
chr10	97163553	97163606	[redacted]	-0.344546	[redacted]
chr6	120659262	120659322	[redacted]	-0.344518	[redacted]
chr10	30482339	30482419	[redacted]	-0.344511	[redacted]
chr6	137402033	137402105	[redacted]	-0.344488	[redacted]
chr15	43872502	43872551	[redacted]	-0.344475	[redacted]
chr10	120349365	120349407	[redacted]	-0.344473	[redacted]
chr6	53029375	53029388	[redacted]	-0.344467	[redacted]
chr10	20430982	20431051	[redacted]	-0.344461	[redacted]
chrX	122868796	122868850	[redacted]	-0.344431	[redacted]
chr12	70268682	70268718	[redacted]	-0.344391	[redacted]
chr7	11650698	11650780	[redacted]	-0.344391	[redacted]
chr8	60900207	60900257	[redacted]	-0.344388	[redacted]
chr8	40983056	40983083	[redacted]	-0.344363	[redacted]
chr6	128317188	128317239	[redacted]	-0.344345	[redacted]
chr11	31583517	31583553	[redacted]	-0.344344	[redacted]
chr14	61044003	61044046	[redacted]	-0.344344	[redacted]
chr9	63047412	63047483	[redacted]	-0.34434	[redacted]
chr3	8999967	9000040	[redacted]	-0.344284	[redacted]
chr3	8886717	8886783	[redacted]	-0.344282	[redacted]
chr6	99486776	99486787	[redacted]	-0.344272	[redacted]
chr7	27134071	27134152	[redacted]	-0.344248	[redacted]
chr2	61721912	61721953	[redacted]	-0.344246	[redacted]
chr5	38664680	38664747	[redacted]	-0.344244	[redacted]
chr17	25498063	25498080	[redacted]	-0.344237	[redacted]
chr8	100766646	100766698	[redacted]	-0.344215	[redacted]
chr3	16698904	16698940	[redacted]	-0.344171	[redacted]
chr10	43340116	43340136	[redacted]	-0.344162	[redacted]
chr4	105315979	105316057	[redacted]	-0.344148	[redacted]
chr4	103085784	103085877	[redacted]	-0.344148	[redacted]
chr3	50608828	50608897	[redacted]	-0.344136	[redacted]
chr9	23554525	23554608	[redacted]	-0.344113	[redacted]

chr9	3025276	3025335	[redacted]	-0.344112	[redacted]
chr11	37242475	37242565	[redacted]	-0.344096	[redacted]
chr2	130671379	130671459	[redacted]	-0.344054	[redacted]
chrX	60614773	60614832	[redacted]	-0.344047	[redacted]
chr19	59076970	59077025	[redacted]	-0.34404	[redacted]
chr12	119196664	119196746	[redacted]	-0.344025	[redacted]
chr1	86253677	86253698	[redacted]	-0.344008	[redacted]
chr2	124351416	124351460	[redacted]	-0.343981	[redacted]
chr5	134977041	134977077	[redacted]	-0.343964	[redacted]
chr4	142825143	142825198	[redacted]	-0.343961	[redacted]
chr7	74445656	74445704	[redacted]	-0.34396	[redacted]
chr2	69705251	69705347	[redacted]	-0.34395	[redacted]
chr1	97804060	97804119	[redacted]	-0.343944	[redacted]
chr14	60528845	60528900	[redacted]	-0.343936	[redacted]
chr18	47115151	47115200	[redacted]	-0.343898	[redacted]
chr15	101011491	101011548	[redacted]	-0.343887	[redacted]
chr8	38233089	38233122	[redacted]	-0.343886	[redacted]
chr17	15728751	15728836	[redacted]	-0.343885	[redacted]
chr18	7403224	7403266	[redacted]	-0.34387	[redacted]
chr2	126506490	126506545	[redacted]	-0.343861	[redacted]
chr16	72589276	72589326	[redacted]	-0.343845	[redacted]
chr8	61178508	61178607	[redacted]	-0.343838	[redacted]
chr4	115178936	115178990	[redacted]	-0.343835	[redacted]
chr6	45425907	45425967	[redacted]	-0.343833	[redacted]
chr1	193357939	193358018	[redacted]	-0.343811	[redacted]
chr15	35211618	35211645	[redacted]	-0.343799	[redacted]
chr8	66612770	66612818	[redacted]	-0.343789	[redacted]
chr3	95099276	95099311	[redacted]	-0.343726	[redacted]
chr9	73505989	73506053	[redacted]	-0.343689	[redacted]
chr8	77697886	77697966	[redacted]	-0.343661	[redacted]
chr10	88597995	88598022	[redacted]	-0.343654	[redacted]
chr3	136168224	136168258	[redacted]	-0.343653	[redacted]
chr11	109729048	109729128	[redacted]	-0.343651	[redacted]
chr10	29405989	29406063	[redacted]	-0.343642	[redacted]
chr17	80221373	80221403	[redacted]	-0.343628	[redacted]
chr19	3617264	3617333	[redacted]	-0.343623	[redacted]
chr15	79457995	79458114	[redacted]	-0.34362	[redacted]
chr18	35259891	35259932	[redacted]	-0.343611	[redacted]
chr11	87402642	87402729	[redacted]	-0.343605	[redacted]
chr16	57281129	57281149	[redacted]	-0.343594	[redacted]
chr16	59426548	59426591	[redacted]	-0.343565	[redacted]
chr5	113817264	113817329	[redacted]	-0.343543	[redacted]
chr2	57547844	57547894	[redacted]	-0.343538	[redacted]

chr10	116656888	116656936	[redacted]	-0.343535	[redacted]
chr14	77215227	77215289	[redacted]	-0.343535	[redacted]
chr16	90039750	90039842	[redacted]	-0.343525	[redacted]
chr6	83237435	83237576	[redacted]	-0.34352	[redacted]
chr3	67558652	67558729	[redacted]	-0.343488	[redacted]
chr3	105045853	105045914	[redacted]	-0.343474	[redacted]
chr6	50810869	50810976	[redacted]	-0.343446	[redacted]
chr1	78465344	78465395	[redacted]	-0.343441	[redacted]
chr12	81214077	81214130	[redacted]	-0.343437	[redacted]
chr10	68173310	68173418	[redacted]	-0.343394	[redacted]
chr2	101591722	101591753	[redacted]	-0.34332	[redacted]
chr5	143004989	143005041	[redacted]	-0.343308	[redacted]
chr18	37227480	37227553	[redacted]	-0.343302	[redacted]
chr6	73337285	73337354	[redacted]	-0.343299	[redacted]
chr14	98753657	98753727	[redacted]	-0.343295	[redacted]
chr6	24959487	24959567	[redacted]	-0.34329	[redacted]
chr15	7488443	7488499	[redacted]	-0.343249	[redacted]
chr1	182048257	182048320	[redacted]	-0.343232	[redacted]
chr5	17499128	17499194	[redacted]	-0.343206	[redacted]
chr15	97571574	97571659	[redacted]	-0.343174	[redacted]
chr10	80518894	80518959	[redacted]	-0.343173	[redacted]
chr7	136691155	136691186	[redacted]	-0.343146	[redacted]
chr4	148783525	148783562	[redacted]	-0.343141	[redacted]
chr14	32552578	32552650	[redacted]	-0.343139	[redacted]
chr10	63026917	63026985	[redacted]	-0.343129	[redacted]
chr13	81107738	81107812	[redacted]	-0.343108	[redacted]
chr7	139002360	139002401	[redacted]	-0.343095	[redacted]
chr12	82949401	82949449	[redacted]	-0.343091	[redacted]
chr3	61668418	61668481	[redacted]	-0.34309	[redacted]
chrX	164288519	164288564	[redacted]	-0.34308	[redacted]
chr19	55323844	55323897	[redacted]	-0.343051	[redacted]
chr16	42945668	42945753	[redacted]	-0.343049	[redacted]
chr11	67106590	67106630	[redacted]	-0.343034	[redacted]
chr10	112715781	112715830	[redacted]	-0.343033	[redacted]
chr5	92802114	92802184	[redacted]	-0.343025	[redacted]
chr9	95119542	95119587	[redacted]	-0.343023	[redacted]
chr2	18663796	18663883	[redacted]	-0.343022	[redacted]
chr2	74938616	74938645	[redacted]	-0.342933	[redacted]
chr18	69483008	69483091	[redacted]	-0.342923	[redacted]
chr13	12159866	12159919	[redacted]	-0.342921	[redacted]
chr15	58892713	58892749	[redacted]	-0.34288	[redacted]
chr9	111729173	111729227	[redacted]	-0.342856	[redacted]
chr1	128024696	128024761	[redacted]	-0.342811	[redacted]

chr8	97386588	97386693	[redacted]	-0.342766	[redacted]
chr3	96062558	96062609	[redacted]	-0.342736	[redacted]
chr4	147744367	147744417	[redacted]	-0.342687	[redacted]
chr18	67208378	67208415	[redacted]	-0.342643	[redacted]
chrX	94071464	94071511	[redacted]	-0.342633	[redacted]
chr15	81656129	81656179	[redacted]	-0.342616	[redacted]
chr4	47961074	47961140	[redacted]	-0.342612	[redacted]
chr11	14500771	14500802	[redacted]	-0.342605	[redacted]
chr1	72201567	72201621	[redacted]	-0.3426	[redacted]
chr1	150906293	150906364	[redacted]	-0.3426	[redacted]
chr13	46766487	46766565	[redacted]	-0.342598	[redacted]
chr4	124085151	124085235	[redacted]	-0.342596	[redacted]
chr1	123689232	123689292	[redacted]	-0.342572	[redacted]
chr5	73242092	73242113	[redacted]	-0.342558	[redacted]
chr11	86213001	86213050	[redacted]	-0.342545	[redacted]
chr1	190007159	190007232	[redacted]	-0.342515	[redacted]
chr10	40896588	40896662	[redacted]	-0.342511	[redacted]
chr8	33611215	33611324	[redacted]	-0.342497	[redacted]
chr7	74240127	74240192	[redacted]	-0.342489	[redacted]
chr19	46000091	46000104	[redacted]	-0.342488	[redacted]
chr7	73789886	73789971	[redacted]	-0.342481	[redacted]
chr17	14697211	14697272	[redacted]	-0.342477	[redacted]
chr17	71998350	71998398	[redacted]	-0.342456	[redacted]
chr3	5118668	5118753	[redacted]	-0.342452	[redacted]
chr17	71482232	71482304	[redacted]	-0.342452	[redacted]
chr2	169845473	169845550	[redacted]	-0.342421	[redacted]
chr15	81300056	81300090	[redacted]	-0.342413	[redacted]
chr6	24000881	24000933	[redacted]	-0.342412	[redacted]
chr6	35847233	35847313	[redacted]	-0.3424	[redacted]
chr3	130090715	130090789	[redacted]	-0.342378	[redacted]
chr2	156490408	156490427	[redacted]	-0.342366	[redacted]
chr2	61013261	61013320	[redacted]	-0.342353	[redacted]
chr2	75288369	75288425	[redacted]	-0.34235	[redacted]
chr4	76297738	76297782	[redacted]	-0.34233	[redacted]
chr18	46765824	46765922	[redacted]	-0.342328	[redacted]
chr10	12019697	12019777	[redacted]	-0.342315	[redacted]
chr1	136557506	136557689	[redacted]	-0.342311	[redacted]
chr6	79843929	79843995	[redacted]	-0.342281	[redacted]
chr13	98046122	98046162	[redacted]	-0.342268	[redacted]
chrX	131893108	131893191	[redacted]	-0.342266	[redacted]
chr16	77846200	77846293	[redacted]	-0.342261	[redacted]
chr4	55759360	55759472	[redacted]	-0.342258	[redacted]
chr7	130402671	130402697	[redacted]	-0.34225	[redacted]

chr11	51782802	51782858	[redacted]	-0.342218	[redacted]
chr18	20218212	20218302	[redacted]	-0.342207	[redacted]
chr9	101864534	101864565	[redacted]	-0.342194	[redacted]
chr19	30111099	30111152	[redacted]	-0.342187	[redacted]
chr16	40624493	40624531	[redacted]	-0.342185	[redacted]
chr13	101003051	101003085	[redacted]	-0.342165	[redacted]
chr16	55823043	55823099	[redacted]	-0.342145	[redacted]
chr12	82212418	82212512	[redacted]	-0.342127	[redacted]
chr9	46660666	46660711	[redacted]	-0.342118	[redacted]
chr11	103202869	103202945	[redacted]	-0.342104	[redacted]
chr9	59361918	59361931	[redacted]	-0.342064	[redacted]
chr3	87444785	87444865	[redacted]	-0.342062	[redacted]
chr16	95412608	95412653	[redacted]	-0.342058	[redacted]
chr15	103932875	103932950	[redacted]	-0.342049	[redacted]
chr18	22558395	22558430	[redacted]	-0.342029	[redacted]
chr2	149311069	149311130	[redacted]	-0.341926	[redacted]
chr15	42316339	42316399	[redacted]	-0.341924	[redacted]
chr6	108383581	108383635	[redacted]	-0.341911	[redacted]
chr1	156044878	156044989	[redacted]	-0.34191	[redacted]
chr5	55022287	55022383	[redacted]	-0.34189	[redacted]
chr6	99165443	99165492	[redacted]	-0.341887	[redacted]
chr3	83792744	83792810	[redacted]	-0.341778	[redacted]
chr6	107389173	107389208	[redacted]	-0.341761	[redacted]
chr4	97998713	97998763	[redacted]	-0.34176	[redacted]
chr4	83892415	83892464	[redacted]	-0.341758	[redacted]
chr10	25160284	25160326	[redacted]	-0.341742	[redacted]
chr13	46841248	46841318	[redacted]	-0.341736	[redacted]
chr12	69566880	69566921	[redacted]	-0.341734	[redacted]
chr1	162154346	162154432	[redacted]	-0.341704	[redacted]
chr17	5337851	5337903	[redacted]	-0.341691	[redacted]
chr13	81170581	81170592	[redacted]	-0.341679	[redacted]
chr6	12321104	12321195	[redacted]	-0.341671	[redacted]
chr4	91605290	91605378	[redacted]	-0.34165	[redacted]
chr17	85899427	85899497	[redacted]	-0.34164	[redacted]
chr11	5641344	5641404	[redacted]	-0.341638	[redacted]
chr1	89188211	89188263	[redacted]	-0.341622	[redacted]
chr2	157287169	157287201	[redacted]	-0.341616	[redacted]
chr5	25957829	25957861	[redacted]	-0.341607	[redacted]
chr15	57576546	57576588	[redacted]	-0.341605	[redacted]
chr11	94579569	94579678	[redacted]	-0.341592	[redacted]
chr3	66776139	66776223	[redacted]	-0.341588	[redacted]
chr3	55353420	55353490	[redacted]	-0.341566	[redacted]
chr8	33903620	33903703	[redacted]	-0.341525	[redacted]

chr15	34906290	34906419	[redacted]	-0.341523	[redacted]
chr11	46126455	46126524	[redacted]	-0.341515	[redacted]
chr2	172976015	172976078	[redacted]	-0.341495	[redacted]
chr14	57804829	57804879	[redacted]	-0.341487	[redacted]
chr12	72282654	72282714	[redacted]	-0.34148	[redacted]
chr6	95045275	95045317	[redacted]	-0.341474	[redacted]
chr15	25628197	25628244	[redacted]	-0.341473	[redacted]
chr8	106555154	106555221	[redacted]	-0.341455	[redacted]
chr10	20286948	20286991	[redacted]	-0.34145	[redacted]
chr2	6398442	6398496	[redacted]	-0.341443	[redacted]
chr18	4830348	4830393	[redacted]	-0.341429	[redacted]
chr1	150696284	150696331	[redacted]	-0.341419	[redacted]
chr5	92168073	92168138	[redacted]	-0.34138	[redacted]
chr16	20614213	20614294	[redacted]	-0.341354	[redacted]
chr4	87632590	87632635	[redacted]	-0.341351	[redacted]
chr3	146818966	146818976	[redacted]	-0.341326	[redacted]
chr17	46894734	46894841	[redacted]	-0.341314	[redacted]
chr13	42714267	42714314	[redacted]	-0.341314	[redacted]
chr7	50187276	50187388	[redacted]	-0.341307	[redacted]
chr6	99525991	99526036	[redacted]	-0.341297	[redacted]
chr19	21740503	21740545	[redacted]	-0.341293	[redacted]
chr9	104032944	104033078	[redacted]	-0.341288	[redacted]
chr19	18285751	18285824	[redacted]	-0.341287	[redacted]
chr11	24051911	24051949	[redacted]	-0.341244	[redacted]
chr4	135161133	135161187	[redacted]	-0.341231	[redacted]
chr18	66588266	66588312	[redacted]	-0.341203	[redacted]
chr3	138555422	138555472	[redacted]	-0.341201	[redacted]
chr10	15936494	15936538	[redacted]	-0.341178	[redacted]
chr11	74941982	74942150	[redacted]	-0.341168	[redacted]
chr4	21934312	21934336	[redacted]	-0.341131	[redacted]
chr6	49948787	49948923	[redacted]	-0.341087	[redacted]
chr10	108435181	108435279	[redacted]	-0.341082	[redacted]
chr2	6140625	6140639	[redacted]	-0.34108	[redacted]
chr18	41916128	41916190	[redacted]	-0.341079	[redacted]
chr19	38177759	38177775	[redacted]	-0.341054	[redacted]
chr1	150952687	150952720	[redacted]	-0.34105	[redacted]
chr13	10033904	10033999	[redacted]	-0.341027	[redacted]
chr10	110716909	110716961	[redacted]	-0.341014	[redacted]
chr13	97581484	97581532	[redacted]	-0.340988	[redacted]
chr2	9234156	9234206	[redacted]	-0.340983	[redacted]
chr2	178695141	178695223	[redacted]	-0.340977	[redacted]
chr9	59595899	59595978	[redacted]	-0.340946	[redacted]
chr15	93302841	93302962	[redacted]	-0.340939	[redacted]

chr9	106515849	106515910	[redacted]	-0.340936	[redacted]
chr3	126663020	126663074	[redacted]	-0.340922	[redacted]
chr15	57124083	57124142	[redacted]	-0.340905	[redacted]
chr5	73182743	73182756	[redacted]	-0.340898	[redacted]
chr19	46690582	46690685	[redacted]	-0.340885	[redacted]
chr6	133566015	133566061	[redacted]	-0.340865	[redacted]
chr13	18792382	18792469	[redacted]	-0.34084	[redacted]
chr9	123862253	123862278	[redacted]	-0.340816	[redacted]
chr11	84676258	84676352	[redacted]	-0.340814	[redacted]
chr16	94108383	94108446	[redacted]	-0.3408	[redacted]
chr18	34800662	34800705	[redacted]	-0.340779	[redacted]
chr12	5151688	5151744	[redacted]	-0.340772	[redacted]
chr10	22701898	22702005	[redacted]	-0.340727	[redacted]
chr16	31857188	31857225	[redacted]	-0.340726	[redacted]
chr17	20981523	20981538	[redacted]	-0.340714	[redacted]
chr12	106853821	106853890	[redacted]	-0.340691	[redacted]
chrX	157977029	157977086	[redacted]	-0.340667	[redacted]
chr2	50359127	50359190	[redacted]	-0.340664	[redacted]
chr8	58897267	58897305	[redacted]	-0.340658	[redacted]
chr5	150439259	150439358	[redacted]	-0.340639	[redacted]
chr6	114916085	114916106	[redacted]	-0.340639	[redacted]
chr13	25201434	25201489	[redacted]	-0.340637	[redacted]
chr6	8352844	8352914	[redacted]	-0.340635	[redacted]
chr13	46427462	46427522	[redacted]	-0.340607	[redacted]
chr13	94223390	94223459	[redacted]	-0.340599	[redacted]
chr2	162844894	162844929	[redacted]	-0.340593	[redacted]
chr2	129073388	129073421	[redacted]	-0.340583	[redacted]
chrX	78501297	78501347	[redacted]	-0.340583	[redacted]
chr5	76647954	76648011	[redacted]	-0.340571	[redacted]
chr5	101171627	101171707	[redacted]	-0.340571	[redacted]
chr19	58878158	58878227	[redacted]	-0.340567	[redacted]
chr2	60330772	60330806	[redacted]	-0.340539	[redacted]
chr17	64238327	64238365	[redacted]	-0.340457	[redacted]
chr2	152323468	152323515	[redacted]	-0.340446	[redacted]
chr8	87066629	87066669	[redacted]	-0.340441	[redacted]
chr1	106033600	106033693	[redacted]	-0.340433	[redacted]
chr5	117472734	117472777	[redacted]	-0.340387	[redacted]
chr4	83280999	83281090	[redacted]	-0.34038	[redacted]
chr7	34414585	34414642	[redacted]	-0.340372	[redacted]
chrX	105874966	105875062	[redacted]	-0.340367	[redacted]
chr15	20250671	20250705	[redacted]	-0.34036	[redacted]
chr4	34261546	34261587	[redacted]	-0.340355	[redacted]
chr15	54651027	54651115	[redacted]	-0.340322	[redacted]

chr1	86467443	86467492	[redacted]	-0.340318	[redacted]
chr12	59021537	59021603	[redacted]	-0.340289	[redacted]
chr19	29846121	29846171	[redacted]	-0.340287	[redacted]
chr6	24525774	24525867	[redacted]	-0.340243	[redacted]
chr13	56698297	56698341	[redacted]	-0.340232	[redacted]
chr9	108336763	108336834	[redacted]	-0.34023	[redacted]
chr10	123171017	123171085	[redacted]	-0.340225	[redacted]
chr4	11076887	11076900	[redacted]	-0.340211	[redacted]
chr13	10920641	10920700	[redacted]	-0.340207	[redacted]
chr13	42442325	42442338	[redacted]	-0.340156	[redacted]
chr14	123694650	123694704	[redacted]	-0.340099	[redacted]
chr15	27171871	27171924	[redacted]	-0.340084	[redacted]
chr13	94546695	94546748	[redacted]	-0.340059	[redacted]
chr19	40042900	40042955	[redacted]	-0.340051	[redacted]
chr4	53891097	53891159	[redacted]	-0.340029	[redacted]
chr13	100916627	100916649	[redacted]	-0.340018	[redacted]
chr3	98332442	98332499	[redacted]	-0.340017	[redacted]
chr5	45487883	45487953	[redacted]	-0.340016	[redacted]
chr2	33212268	33212282	[redacted]	-0.340016	[redacted]
chr17	63288174	63288204	[redacted]	-0.340013	[redacted]
chr2	84241457	84241502	[redacted]	-0.340006	[redacted]
chr1	125544006	125544132	[redacted]	-0.339979	[redacted]
chr14	60574993	60575022	[redacted]	-0.339976	[redacted]
chr15	93385975	93386042	[redacted]	-0.339954	[redacted]
chr17	88521830	88521924	[redacted]	-0.339954	[redacted]
chr10	80171114	80171174	[redacted]	-0.339951	[redacted]
chr15	41819570	41819667	[redacted]	-0.339951	[redacted]
chr14	122516421	122516458	[redacted]	-0.339944	[redacted]
chr5	72263839	72263850	[redacted]	-0.339943	[redacted]
chr18	46611439	46611479	[redacted]	-0.339915	[redacted]
chr11	35678003	35678080	[redacted]	-0.339883	[redacted]
chr11	96360625	96360658	[redacted]	-0.33988	[redacted]
chr13	34154738	34154850	[redacted]	-0.339877	[redacted]
chr6	8523320	8523390	[redacted]	-0.339876	[redacted]
chr10	90350493	90350543	[redacted]	-0.339873	[redacted]
chr5	27353824	27353902	[redacted]	-0.339853	[redacted]
chr8	110190226	110190281	[redacted]	-0.339844	[redacted]
chr10	128612241	128612350	[redacted]	-0.339835	[redacted]
chr9	100493595	100493643	[redacted]	-0.339831	[redacted]
chr5	98027924	98027966	[redacted]	-0.339822	[redacted]
chr2	109953309	109953348	[redacted]	-0.339813	[redacted]
chr3	30557368	30557435	[redacted]	-0.339797	[redacted]
chr11	58947055	58947111	[redacted]	-0.339785	[redacted]

chr15	101590395	101590450	[redacted]	-0.339769	[redacted]
chr2	8649495	8649524	[redacted]	-0.339769	[redacted]
chr17	7434306	7434393	[redacted]	-0.339761	[redacted]
chr2	47076748	47076848	[redacted]	-0.339761	[redacted]
chr9	29125171	29125226	[redacted]	-0.339739	[redacted]
chr10	59960250	59960298	[redacted]	-0.339731	[redacted]
chr2	106641497	106641553	[redacted]	-0.339711	[redacted]
chr3	138157198	138157253	[redacted]	-0.339695	[redacted]
chr15	77918780	77918842	[redacted]	-0.339672	[redacted]
chr17	43658292	43658342	[redacted]	-0.339661	[redacted]
chr2	128137588	128137620	[redacted]	-0.33963	[redacted]
chr5	133801626	133801653	[redacted]	-0.339621	[redacted]
chr17	24417468	24417484	[redacted]	-0.339614	[redacted]
chr6	117686170	117686199	[redacted]	-0.339603	[redacted]
chr1	39672186	39672316	[redacted]	-0.339558	[redacted]
chr12	100545630	100545646	[redacted]	-0.339545	[redacted]
chr19	61229447	61229494	[redacted]	-0.339543	[redacted]
chr1	177038195	177038277	[redacted]	-0.339536	[redacted]
chr16	75398449	75398486	[redacted]	-0.339527	[redacted]
chr9	10683329	10683371	[redacted]	-0.339522	[redacted]
chr9	112008719	112008783	[redacted]	-0.339514	[redacted]
chr2	74482732	74482811	[redacted]	-0.339464	[redacted]
chr13	94251098	94251137	[redacted]	-0.339439	[redacted]
chrX	23327161	23327201	[redacted]	-0.339432	[redacted]
chr10	25293252	25293318	[redacted]	-0.33941	[redacted]
chr17	15142398	15142472	[redacted]	-0.339401	[redacted]
chr7	142039794	142039859	[redacted]	-0.339377	[redacted]
chr5	66360280	66360325	[redacted]	-0.33936	[redacted]
chr5	73245089	73245353	[redacted]	-0.339339	[redacted]
chr5	106635621	106635638	[redacted]	-0.339325	[redacted]
chr5	53567764	53567822	[redacted]	-0.33931	[redacted]
chr6	52367819	52367867	[redacted]	-0.339303	[redacted]
chr14	54621871	54621924	[redacted]	-0.339299	[redacted]
chr5	81798206	81798252	[redacted]	-0.339299	[redacted]
chr7	130357248	130357324	[redacted]	-0.339297	[redacted]
chr10	56646964	56647024	[redacted]	-0.339294	[redacted]
chr16	55608806	55608876	[redacted]	-0.339292	[redacted]
chr11	118180388	118180406	[redacted]	-0.339286	[redacted]
chr7	16359874	16359935	[redacted]	-0.339262	[redacted]
chr11	119220301	119220328	[redacted]	-0.339249	[redacted]
chr1	159126132	159126167	[redacted]	-0.339235	[redacted]
chr17	65967952	65967976	[redacted]	-0.339232	[redacted]
chr13	16655676	16655706	[redacted]	-0.339221	[redacted]

chr11	98516258	98516326	[redacted]	-0.339212	[redacted]
chr16	38780807	38780870	[redacted]	-0.339202	[redacted]
chr7	122505221	122505247	[redacted]	-0.339181	[redacted]
chr1	38658252	38658279	[redacted]	-0.33918	[redacted]
chr12	82642368	82642453	[redacted]	-0.339141	[redacted]
chr3	108299776	108299802	[redacted]	-0.339133	[redacted]
chr5	138894614	138894630	[redacted]	-0.339108	[redacted]
chr7	79925699	79925737	[redacted]	-0.339078	[redacted]
chr1	135682949	135682992	[redacted]	-0.339068	[redacted]
chr5	81897309	81897340	[redacted]	-0.339068	[redacted]
chr17	5613715	5613735	[redacted]	-0.339058	[redacted]
chr9	109888369	109888416	[redacted]	-0.339046	[redacted]
chr14	18846346	18846368	[redacted]	-0.339045	[redacted]
chr10	26371761	26371812	[redacted]	-0.339044	[redacted]
chr12	17883436	17883494	[redacted]	-0.339017	[redacted]
chr11	87218886	87218982	[redacted]	-0.339005	[redacted]
chr7	141046019	141046063	[redacted]	-0.338999	[redacted]
chr7	46975839	46975890	[redacted]	-0.338952	[redacted]
chr11	4699047	4699112	[redacted]	-0.338948	[redacted]
chr15	91870452	91870494	[redacted]	-0.338921	[redacted]
chr8	9029733	9029769	[redacted]	-0.338921	[redacted]
chr15	59511589	59511645	[redacted]	-0.338892	[redacted]
chr3	8429194	8429245	[redacted]	-0.338892	[redacted]
chr7	92214117	92214171	[redacted]	-0.338888	[redacted]
chr2	178262005	178262048	[redacted]	-0.338879	[redacted]
chr8	80547135	80547191	[redacted]	-0.338873	[redacted]
chr3	87309123	87309190	[redacted]	-0.338864	[redacted]
chr2	60200095	60200109	[redacted]	-0.33885	[redacted]
chr4	96071721	96071774	[redacted]	-0.338831	[redacted]
chr11	79197655	79197701	[redacted]	-0.338792	[redacted]
chr4	8803694	8803738	[redacted]	-0.338787	[redacted]
chr17	13821437	13821529	[redacted]	-0.338776	[redacted]
chr19	51451677	51451732	[redacted]	-0.338753	[redacted]
chr18	63557798	63557834	[redacted]	-0.338746	[redacted]
chr3	30787406	30787444	[redacted]	-0.338736	[redacted]
chr3	126640824	126640855	[redacted]	-0.338723	[redacted]
chr9	48276956	48276993	[redacted]	-0.338714	[redacted]
chr15	41674629	41674705	[redacted]	-0.338709	[redacted]
chr18	58484244	58484305	[redacted]	-0.33869	[redacted]
chr15	20716816	20716907	[redacted]	-0.338686	[redacted]
chr2	7101811	7101885	[redacted]	-0.338671	[redacted]
chr3	89212925	89213016	[redacted]	-0.338668	[redacted]
chr3	37959584	37959707	[redacted]	-0.338667	[redacted]

chr9	77935818	77935883	[redacted]	-0.33866	[redacted]
chr13	107799832	107799873	[redacted]	-0.338627	[redacted]
chr2	164634587	164634638	[redacted]	-0.338591	[redacted]
chr6	132707398	132707438	[redacted]	-0.338588	[redacted]
chr15	100883963	100884009	[redacted]	-0.338573	[redacted]
chr6	80613655	80613746	[redacted]	-0.338569	[redacted]
chr2	10170180	10170305	[redacted]	-0.338554	[redacted]
chr11	112744397	112744429	[redacted]	-0.338503	[redacted]
chr15	4920953	4920968	[redacted]	-0.338478	[redacted]
chr14	62561992	62562027	[redacted]	-0.338475	[redacted]
chr18	28566911	28566960	[redacted]	-0.338457	[redacted]
chr19	5987277	5987315	[redacted]	-0.338454	[redacted]
chr5	27820840	27820902	[redacted]	-0.338429	[redacted]
chr9	53368659	53368703	[redacted]	-0.338423	[redacted]
chr3	10654777	10654830	[redacted]	-0.338414	[redacted]
chr13	116300116	116300179	[redacted]	-0.338408	[redacted]
chr5	53145694	53145711	[redacted]	-0.338404	[redacted]
chr8	106172313	106172380	[redacted]	-0.338402	[redacted]
chr16	49766300	49766322	[redacted]	-0.338401	[redacted]
chr8	3698381	3698502	[redacted]	-0.338399	[redacted]
chr9	52255695	52255764	[redacted]	-0.338397	[redacted]
chr10	43035264	43035294	[redacted]	-0.338374	[redacted]
chr12	85544041	85544092	[redacted]	-0.338374	[redacted]
chr7	107046275	107046307	[redacted]	-0.338338	[redacted]
chr3	81026985	81027038	[redacted]	-0.338334	[redacted]
chr13	111371485	111371551	[redacted]	-0.338311	[redacted]
chr17	36102956	36102984	[redacted]	-0.33829	[redacted]
chr4	66447906	66448011	[redacted]	-0.338283	[redacted]
chr4	94651020	94651103	[redacted]	-0.338268	[redacted]
chr1	35695163	35695277	[redacted]	-0.338252	[redacted]
chr10	40725203	40725269	[redacted]	-0.338251	[redacted]
chr5	142572391	142572454	[redacted]	-0.338249	[redacted]
chrX	72795627	72795662	[redacted]	-0.338248	[redacted]
chr10	116709040	116709075	[redacted]	-0.338228	[redacted]
chr17	70578707	70578825	[redacted]	-0.338219	[redacted]
chr9	43522682	43522776	[redacted]	-0.338205	[redacted]
chr18	24356005	24356067	[redacted]	-0.338187	[redacted]
chr15	11846638	11846684	[redacted]	-0.338175	[redacted]
chr8	115073214	115073254	[redacted]	-0.338166	[redacted]
chr15	58941899	58942048	[redacted]	-0.338165	[redacted]
chr5	24382883	24382927	[redacted]	-0.338151	[redacted]
chr12	73926570	73926610	[redacted]	-0.338137	[redacted]
chr1	175987528	175987595	[redacted]	-0.338118	[redacted]

chr3	139896388	139896408	[redacted]	-0.338112	[redacted]
chr7	75528124	75528228	[redacted]	-0.338106	[redacted]
chr2	17295522	17295589	[redacted]	-0.338031	[redacted]
chr1	55160720	55160785	[redacted]	-0.338026	[redacted]
chr8	46000563	46000613	[redacted]	-0.33802	[redacted]
chr13	61587024	61587083	[redacted]	-0.338015	[redacted]
chr3	21097492	21097539	[redacted]	-0.338008	[redacted]
chr8	79350491	79350576	[redacted]	-0.337991	[redacted]
chrX	52631770	52631824	[redacted]	-0.337968	[redacted]
chr13	111522360	111522486	[redacted]	-0.33794	[redacted]
chr4	45014521	45014554	[redacted]	-0.337911	[redacted]
chr2	114873789	114873822	[redacted]	-0.337882	[redacted]
chr11	5178441	5178454	[redacted]	-0.33788	[redacted]
chr9	121852504	121852552	[redacted]	-0.337841	[redacted]
chr12	30564533	30564621	[redacted]	-0.337814	[redacted]
chr12	22773432	22773437	[redacted]	-0.337804	[redacted]
chr2	124536220	124536252	[redacted]	-0.337787	[redacted]
chr17	5495307	5495359	[redacted]	-0.337779	[redacted]
chr1	168911973	168912012	[redacted]	-0.337773	[redacted]
chr14	117698533	117698575	[redacted]	-0.337765	[redacted]
chr14	94086915	94086991	[redacted]	-0.337763	[redacted]
chr1	55756078	55756133	[redacted]	-0.337711	[redacted]
chr3	28603583	28603625	[redacted]	-0.337707	[redacted]
chr3	34313868	34313951	[redacted]	-0.337706	[redacted]
chr1	43294712	43294765	[redacted]	-0.337705	[redacted]
chr2	158770367	158770456	[redacted]	-0.337698	[redacted]
chr9	20999678	20999745	[redacted]	-0.337687	[redacted]
chr3	138342235	138342252	[redacted]	-0.337662	[redacted]
chr13	96470488	96470541	[redacted]	-0.337659	[redacted]
chr1	83849967	83850065	[redacted]	-0.337628	[redacted]
chr7	123025029	123025080	[redacted]	-0.337625	[redacted]
chr10	122953646	122953730	[redacted]	-0.337611	[redacted]
chr11	113284975	113285125	[redacted]	-0.337606	[redacted]
chr3	16420315	16420374	[redacted]	-0.337606	[redacted]
chr14	48493336	48493387	[redacted]	-0.33759	[redacted]
chr6	134858589	134858627	[redacted]	-0.337589	[redacted]
chr15	53987496	53987530	[redacted]	-0.337579	[redacted]
chr17	48214642	48214728	[redacted]	-0.337567	[redacted]
chr3	101138315	101138355	[redacted]	-0.33756	[redacted]
chr1	124736761	124736855	[redacted]	-0.337542	[redacted]
chr5	34346628	34346670	[redacted]	-0.33751	[redacted]
chr5	96095468	96095570	[redacted]	-0.337509	[redacted]
chrX	38680138	38680183	[redacted]	-0.337498	[redacted]

chr10	80687960	80688002	[redacted]	-0.337496	[redacted]
chr11	51451939	51452001	[redacted]	-0.337493	[redacted]
chr13	79650445	79650520	[redacted]	-0.337487	[redacted]
chr19	10911420	10911461	[redacted]	-0.337485	[redacted]
chr2	59296101	59296173	[redacted]	-0.33748	[redacted]
chr10	63698297	63698396	[redacted]	-0.337464	[redacted]
chr4	132511375	132511400	[redacted]	-0.337448	[redacted]
chr15	58111157	58111197	[redacted]	-0.337434	[redacted]
chr1	176221759	176221884	[redacted]	-0.337421	[redacted]
chr1	177867958	177867981	[redacted]	-0.33742	[redacted]
chr12	55006859	55006875	[redacted]	-0.337414	[redacted]
chr10	96628511	96628558	[redacted]	-0.337412	[redacted]
chr11	68167608	68167660	[redacted]	-0.337406	[redacted]
chr8	114779711	114779767	[redacted]	-0.33739	[redacted]
chr15	63821760	63821872	[redacted]	-0.337382	[redacted]
chr7	16005589	16005606	[redacted]	-0.337375	[redacted]
chr9	95375310	95375368	[redacted]	-0.337367	[redacted]
chr5	136743506	136743591	[redacted]	-0.337357	[redacted]
chr16	35893752	35893801	[redacted]	-0.33735	[redacted]
chr18	36017531	36017583	[redacted]	-0.337343	[redacted]
chr4	33246603	33246664	[redacted]	-0.337329	[redacted]
chr4	132052713	132052754	[redacted]	-0.337323	[redacted]
chr16	38234919	38235017	[redacted]	-0.337299	[redacted]
chr3	53538078	53538096	[redacted]	-0.337286	[redacted]
chr10	14254612	14254701	[redacted]	-0.33728	[redacted]
chr3	95866595	95866650	[redacted]	-0.337252	[redacted]
chr7	6274392	6274405	[redacted]	-0.337252	[redacted]
chr14	13687855	13687886	[redacted]	-0.337246	[redacted]
chr8	91126054	91126063	[redacted]	-0.337234	[redacted]
chr6	32998117	32998141	[redacted]	-0.337222	[redacted]
chr6	98333690	98333739	[redacted]	-0.337187	[redacted]
chr5	122495656	122495741	[redacted]	-0.337182	[redacted]
chr13	72521413	72521435	[redacted]	-0.337173	[redacted]
chr6	51974048	51974093	[redacted]	-0.337171	[redacted]
chr4	78062994	78063056	[redacted]	-0.337147	[redacted]
chr16	16990402	16990447	[redacted]	-0.337143	[redacted]
chr9	52521929	52521976	[redacted]	-0.337137	[redacted]
chr11	103285006	103285054	[redacted]	-0.337134	[redacted]
chr11	117055356	117055476	[redacted]	-0.337104	[redacted]
chr9	20800256	20800366	[redacted]	-0.3371	[redacted]
chr13	13139740	13139765	[redacted]	-0.337098	[redacted]
chr9	53211653	53211722	[redacted]	-0.337069	[redacted]
chr12	55537971	55538042	[redacted]	-0.337038	[redacted]

chr12	75663071	75663141	[redacted]	-0.337032	[redacted]
chr12	69610472	69610579	[redacted]	-0.337027	[redacted]
chr19	46563363	46563420	[redacted]	-0.337023	[redacted]
chr5	135100343	135100394	[redacted]	-0.336982	[redacted]
chr3	94784289	94784338	[redacted]	-0.336977	[redacted]
chr3	83625392	83625456	[redacted]	-0.336936	[redacted]
chr17	73921912	73921966	[redacted]	-0.33691	[redacted]
chr6	89096528	89096613	[redacted]	-0.33689	[redacted]
chr8	106245745	106245792	[redacted]	-0.336877	[redacted]
chr9	28920214	28920293	[redacted]	-0.336865	[redacted]
chr3	120314406	120314469	[redacted]	-0.336849	[redacted]
chr2	67266487	67266518	[redacted]	-0.336836	[redacted]
chr2	103254438	103254466	[redacted]	-0.33681	[redacted]
chr6	62937658	62937742	[redacted]	-0.336805	[redacted]
chr1	92899181	92899227	[redacted]	-0.336793	[redacted]
chr5	101856792	101856871	[redacted]	-0.336777	[redacted]
chr10	111979986	111980027	[redacted]	-0.336776	[redacted]
chr12	54304897	54304953	[redacted]	-0.336757	[redacted]
chr9	95790351	95790416	[redacted]	-0.336725	[redacted]
chr15	97707459	97707481	[redacted]	-0.336713	[redacted]
chrX	157468717	157468798	[redacted]	-0.336703	[redacted]
chr14	40073108	40073151	[redacted]	-0.336686	[redacted]
chrX	94364454	94364501	[redacted]	-0.336683	[redacted]
chrX	133681404	133681466	[redacted]	-0.336666	[redacted]
chr11	23708820	23708884	[redacted]	-0.336656	[redacted]
chrX	123098250	123098300	[redacted]	-0.336589	[redacted]
chr5	129079019	129079089	[redacted]	-0.33658	[redacted]
chr10	111682543	111682602	[redacted]	-0.336561	[redacted]
chr13	96746830	96746855	[redacted]	-0.336552	[redacted]
chr6	127478021	127478075	[redacted]	-0.336528	[redacted]
chr5	145108529	145108586	[redacted]	-0.336518	[redacted]
chr15	99405146	99405168	[redacted]	-0.336496	[redacted]
chr2	174676330	174676376	[redacted]	-0.336461	[redacted]
chr7	118958240	118958274	[redacted]	-0.33644	[redacted]
chr1	88330171	88330192	[redacted]	-0.336434	[redacted]
chr19	24613523	24613579	[redacted]	-0.336407	[redacted]
chr19	38720993	38721090	[redacted]	-0.336394	[redacted]
chr19	51700766	51700820	[redacted]	-0.336374	[redacted]
chr2	167490745	167490798	[redacted]	-0.336364	[redacted]
chr5	142060339	142060435	[redacted]	-0.336351	[redacted]
chr18	66463536	66463623	[redacted]	-0.336325	[redacted]
chr16	34504415	34504461	[redacted]	-0.336302	[redacted]
chr19	44372725	44372743	[redacted]	-0.336294	[redacted]

chr10	111143566	111143625	[redacted]	-0.336277	[redacted]
chr19	34749716	34749771	[redacted]	-0.336167	[redacted]
chr14	78629866	78629938	[redacted]	-0.336157	[redacted]
chr10	93934345	93934421	[redacted]	-0.336151	[redacted]
chr17	24712446	24712509	[redacted]	-0.336148	[redacted]
chr6	94869779	94869846	[redacted]	-0.336139	[redacted]
chr3	119358317	119358375	[redacted]	-0.336117	[redacted]
chr17	39767060	39767142	[redacted]	-0.336083	[redacted]
chr8	111928738	111928784	[redacted]	-0.336082	[redacted]
chr7	12603948	12604001	[redacted]	-0.336082	[redacted]
chr15	74754855	74754874	[redacted]	-0.33608	[redacted]
chr16	66550670	66550740	[redacted]	-0.336076	[redacted]
chr2	74915870	74915943	[redacted]	-0.336074	[redacted]
chr18	42281826	42281914	[redacted]	-0.336071	[redacted]
chr14	115028560	115028607	[redacted]	-0.336068	[redacted]
chr18	66329236	66329328	[redacted]	-0.336036	[redacted]
chr6	144484603	144484638	[redacted]	-0.336019	[redacted]
chr1	181920206	181920271	[redacted]	-0.33601	[redacted]
chr17	14457657	14457747	[redacted]	-0.336007	[redacted]
chr14	48333297	48333353	[redacted]	-0.335976	[redacted]
chr18	38897096	38897128	[redacted]	-0.335947	[redacted]
chr8	82979960	82980044	[redacted]	-0.335943	[redacted]
chr2	79878848	79878868	[redacted]	-0.335935	[redacted]
chr4	106581826	106582068	[redacted]	-0.335933	[redacted]
chr18	69578639	69578685	[redacted]	-0.335922	[redacted]
chr7	97268700	97268755	[redacted]	-0.335909	[redacted]
chr9	46103214	46103332	[redacted]	-0.335908	[redacted]
chr11	4434422	4434476	[redacted]	-0.335898	[redacted]
chr6	137051470	137051547	[redacted]	-0.335895	[redacted]
chr15	96693673	96693716	[redacted]	-0.335875	[redacted]
chr2	71043588	71043601	[redacted]	-0.335874	[redacted]
chr13	45910550	45910572	[redacted]	-0.335843	[redacted]
chr2	57508318	57508343	[redacted]	-0.335832	[redacted]
chr9	28877178	28877201	[redacted]	-0.335821	[redacted]
chr12	94789713	94789786	[redacted]	-0.335805	[redacted]
chr10	18542298	18542395	[redacted]	-0.335768	[redacted]
chr11	87237503	87237569	[redacted]	-0.335762	[redacted]
chr4	127873653	127873699	[redacted]	-0.335749	[redacted]
chr9	87148654	87148671	[redacted]	-0.335747	[redacted]
chr10	111140250	111140291	[redacted]	-0.335744	[redacted]
chr12	5415762	5415794	[redacted]	-0.335738	[redacted]
chr10	56595718	56595758	[redacted]	-0.335735	[redacted]
chr10	26293921	26294018	[redacted]	-0.335721	[redacted]

chr16	35510804	35510869	[redacted]	-0.33572	[redacted]
chr17	67107970	67108032	[redacted]	-0.335714	[redacted]
chr10	67033965	67033996	[redacted]	-0.335689	[redacted]
chr6	100622291	100622320	[redacted]	-0.335678	[redacted]
chr2	50226155	50226198	[redacted]	-0.335673	[redacted]
chr15	91009497	91009514	[redacted]	-0.33566	[redacted]
chr2	165682231	165682283	[redacted]	-0.335635	[redacted]
chr18	4860613	4860663	[redacted]	-0.33562	[redacted]
chr13	101680660	101680733	[redacted]	-0.335596	[redacted]
chr5	100930383	100930435	[redacted]	-0.335594	[redacted]
chr11	56040066	56040138	[redacted]	-0.335569	[redacted]
chr19	10744072	10744112	[redacted]	-0.335555	[redacted]
chr13	34083584	34083631	[redacted]	-0.335519	[redacted]
chr4	155084842	155084864	[redacted]	-0.335517	[redacted]
chr3	53226430	53226461	[redacted]	-0.335495	[redacted]
chr10	20248140	20248221	[redacted]	-0.335492	[redacted]
chr16	58699767	58699793	[redacted]	-0.335462	[redacted]
chr1	133722835	133722874	[redacted]	-0.335458	[redacted]
chr3	108126153	108126220	[redacted]	-0.335448	[redacted]
chr17	43365687	43365755	[redacted]	-0.335446	[redacted]
chr10	85448680	85448746	[redacted]	-0.335438	[redacted]
chr5	129372251	129372328	[redacted]	-0.335424	[redacted]
chr12	40073760	40073807	[redacted]	-0.335408	[redacted]
chr15	16170101	16170185	[redacted]	-0.335374	[redacted]
chr9	123201649	123201685	[redacted]	-0.335358	[redacted]
chrX	38129757	38129788	[redacted]	-0.335345	[redacted]
chr19	15040652	15040714	[redacted]	-0.33534	[redacted]
chr17	13348173	13348199	[redacted]	-0.335333	[redacted]
chr12	51805227	51805282	[redacted]	-0.335327	[redacted]
chr18	13931169	13931219	[redacted]	-0.33531	[redacted]
chrX	72015557	72015643	[redacted]	-0.335304	[redacted]
chr13	98452360	98452440	[redacted]	-0.335284	[redacted]
chr4	15426695	15426783	[redacted]	-0.335274	[redacted]
chr15	8510314	8510354	[redacted]	-0.335265	[redacted]
chr7	55281780	55281818	[redacted]	-0.335261	[redacted]
chr16	52393106	52393145	[redacted]	-0.335231	[redacted]
chr4	109558643	109558678	[redacted]	-0.33523	[redacted]
chr3	132790382	132790444	[redacted]	-0.33522	[redacted]
chr19	61303319	61303354	[redacted]	-0.335211	[redacted]
chr19	40752119	40752171	[redacted]	-0.335204	[redacted]
chr18	77859376	77859425	[redacted]	-0.335198	[redacted]
chr9	22414229	22414277	[redacted]	-0.33517	[redacted]
chr6	67276015	67276080	[redacted]	-0.335162	[redacted]

chr8	121256324	121256387	[redacted]	-0.335161	[redacted]
chr17	70498787	70498835	[redacted]	-0.335132	[redacted]
chr18	35527432	35527511	[redacted]	-0.335119	[redacted]
chr4	128011856	128011905	[redacted]	-0.335114	[redacted]
chr9	109577289	109577349	[redacted]	-0.335108	[redacted]
chr2	84894990	84895023	[redacted]	-0.335099	[redacted]
chr8	46357777	46357812	[redacted]	-0.335065	[redacted]
chr5	97493221	97493302	[redacted]	-0.335061	[redacted]
chr3	69135689	69135771	[redacted]	-0.335046	[redacted]
chr1	173934135	173934156	[redacted]	-0.335028	[redacted]
chr15	39855663	39855761	[redacted]	-0.335022	[redacted]
chr12	33795571	33795623	[redacted]	-0.335013	[redacted]
chr7	112906094	112906143	[redacted]	-0.335	[redacted]
chr7	130296884	130296926	[redacted]	-0.33498	[redacted]
chr5	64173941	64173994	[redacted]	-0.334971	[redacted]
chr2	172185327	172185399	[redacted]	-0.334963	[redacted]
chr11	109059451	109059530	[redacted]	-0.334941	[redacted]
chr8	11053521	11053591	[redacted]	-0.334933	[redacted]
chr3	88834974	88835017	[redacted]	-0.334932	[redacted]
chr2	72676832	72676889	[redacted]	-0.33493	[redacted]
chr15	97406452	97406501	[redacted]	-0.334925	[redacted]
chr19	47713701	47713820	[redacted]	-0.334916	[redacted]
chr11	72635704	72635746	[redacted]	-0.334901	[redacted]
chr9	119016885	119016997	[redacted]	-0.334892	[redacted]
chr6	24374065	24374127	[redacted]	-0.334888	[redacted]
chr10	119312407	119312474	[redacted]	-0.334882	[redacted]
chr3	159843290	159843396	[redacted]	-0.334878	[redacted]
chr16	44672290	44672328	[redacted]	-0.334847	[redacted]
chr8	40847042	40847118	[redacted]	-0.334838	[redacted]
chr10	110258384	110258446	[redacted]	-0.334812	[redacted]
chr19	59085266	59085358	[redacted]	-0.334806	[redacted]
chr11	6054394	6054486	[redacted]	-0.334779	[redacted]
chr18	29217136	29217185	[redacted]	-0.334771	[redacted]
chr7	133576426	133576464	[redacted]	-0.334753	[redacted]
chr2	66309669	66309730	[redacted]	-0.334753	[redacted]
chr15	33080024	33080114	[redacted]	-0.334746	[redacted]
chr19	56900556	56900600	[redacted]	-0.33474	[redacted]
chrX	108942661	108942746	[redacted]	-0.334736	[redacted]
chr7	64592950	64592999	[redacted]	-0.334715	[redacted]
chr10	29218444	29218501	[redacted]	-0.334702	[redacted]
chr6	34142422	34142473	[redacted]	-0.334694	[redacted]
chr2	21007817	21007908	[redacted]	-0.33467	[redacted]
chr10	115816401	115816464	[redacted]	-0.334649	[redacted]

chr17	7147893	7148048	[redacted]	-0.334642	[redacted]
chr12	60302191	60302222	[redacted]	-0.334637	[redacted]
chr13	21202993	21203025	[redacted]	-0.334634	[redacted]
chr14	30041302	30041333	[redacted]	-0.334628	[redacted]
chr14	28650686	28650777	[redacted]	-0.334628	[redacted]
chr13	108969016	108969092	[redacted]	-0.334623	[redacted]
chr15	88038498	88038532	[redacted]	-0.334599	[redacted]
chr5	141571365	141571385	[redacted]	-0.334585	[redacted]
chr11	77888328	77888360	[redacted]	-0.334582	[redacted]
chr1	24419798	24419838	[redacted]	-0.334574	[redacted]
chr9	110755497	110755562	[redacted]	-0.334566	[redacted]
chr11	103952651	103952684	[redacted]	-0.334545	[redacted]
chr15	77346575	77346601	[redacted]	-0.334537	[redacted]
chr15	79626193	79626250	[redacted]	-0.334532	[redacted]
chr5	103860236	103860288	[redacted]	-0.334518	[redacted]
chr1	177687120	177687139	[redacted]	-0.334495	[redacted]
chr2	89911617	89911688	[redacted]	-0.334493	[redacted]
chr12	14181785	14181839	[redacted]	-0.334492	[redacted]
chr2	144565482	144565554	[redacted]	-0.334491	[redacted]
chr5	75796704	75796752	[redacted]	-0.334487	[redacted]
chr12	71240154	71240219	[redacted]	-0.334482	[redacted]
chr2	124926707	124926757	[redacted]	-0.334481	[redacted]
chr15	49998139	49998224	[redacted]	-0.334466	[redacted]
chr4	40013197	40013264	[redacted]	-0.334446	[redacted]
chr18	68818426	68818519	[redacted]	-0.334443	[redacted]
chr15	95679788	95679854	[redacted]	-0.334418	[redacted]
chr4	38571305	38571391	[redacted]	-0.334403	[redacted]
chr2	126279223	126279296	[redacted]	-0.3344	[redacted]
chr8	62825240	62825274	[redacted]	-0.334387	[redacted]
chr8	109276644	109276716	[redacted]	-0.334377	[redacted]
chr1	53931528	53931619	[redacted]	-0.334376	[redacted]
chr10	75246254	75246291	[redacted]	-0.334374	[redacted]
chr14	103515821	103515941	[redacted]	-0.334366	[redacted]
chr6	15548546	15548638	[redacted]	-0.334347	[redacted]
chr15	46906037	46906081	[redacted]	-0.334337	[redacted]
chr14	47287484	47287552	[redacted]	-0.334331	[redacted]
chr3	84833987	84834036	[redacted]	-0.334307	[redacted]
chr7	86672174	86672220	[redacted]	-0.334299	[redacted]
chr19	33642781	33642829	[redacted]	-0.334289	[redacted]
chr11	25817957	25818048	[redacted]	-0.334276	[redacted]
chr5	19064481	19064555	[redacted]	-0.334275	[redacted]
chr6	137141805	137141848	[redacted]	-0.33427	[redacted]
chr1	65169815	65169891	[redacted]	-0.334269	[redacted]

chr14	60496588	60496652	[redacted]	-0.334259	[redacted]
chr1	60955620	60955711	[redacted]	-0.334258	[redacted]
chr4	136437494	136437570	[redacted]	-0.334253	[redacted]
chr16	37942464	37942528	[redacted]	-0.334245	[redacted]
chr6	51617926	51617967	[redacted]	-0.334206	[redacted]
chr5	32844638	32844667	[redacted]	-0.334192	[redacted]
chrX	101997915	101997994	[redacted]	-0.334186	[redacted]
chr15	56217571	56217633	[redacted]	-0.334177	[redacted]
chr9	16056667	16056804	[redacted]	-0.334169	[redacted]
chr1	170736440	170736493	[redacted]	-0.334168	[redacted]
chr11	76321739	76321817	[redacted]	-0.334165	[redacted]
chr15	12160491	12160516	[redacted]	-0.334161	[redacted]
chr14	65071858	65071915	[redacted]	-0.334159	[redacted]
chr2	164093244	164093310	[redacted]	-0.334119	[redacted]
chr10	58942902	58942977	[redacted]	-0.33408	[redacted]
chr6	84908130	84908165	[redacted]	-0.334051	[redacted]
chr16	38449950	38450031	[redacted]	-0.334048	[redacted]
chr14	107929286	107929363	[redacted]	-0.334032	[redacted]
chr15	62046270	62046331	[redacted]	-0.333992	[redacted]
chr18	62822768	62822831	[redacted]	-0.333972	[redacted]
chr9	54380993	54381021	[redacted]	-0.333968	[redacted]
chr9	53609556	53609579	[redacted]	-0.333965	[redacted]
chr2	146300486	146300510	[redacted]	-0.333935	[redacted]
chr11	83272973	83273056	[redacted]	-0.333904	[redacted]
chr2	60116330	60116373	[redacted]	-0.333864	[redacted]
chr13	24452793	24452871	[redacted]	-0.333855	[redacted]
chr5	129758337	129758391	[redacted]	-0.33384	[redacted]
chr10	117731931	117731975	[redacted]	-0.33384	[redacted]
chr1	38945994	38946064	[redacted]	-0.33383	[redacted]
chr9	41110089	41110119	[redacted]	-0.33383	[redacted]
chr6	101074654	101074687	[redacted]	-0.333821	[redacted]
chr15	5640176	5640205	[redacted]	-0.333814	[redacted]
chr13	20906134	20906207	[redacted]	-0.333808	[redacted]
chr6	32973879	32973896	[redacted]	-0.333806	[redacted]
chr12	32146812	32146856	[redacted]	-0.333792	[redacted]
chr16	97020290	97020399	[redacted]	-0.33378	[redacted]
chr15	7196304	7196389	[redacted]	-0.333776	[redacted]
chrX	161076641	161076668	[redacted]	-0.333768	[redacted]
chr1	152896383	152896439	[redacted]	-0.333752	[redacted]
chr7	75032051	75032114	[redacted]	-0.333728	[redacted]
chr6	144905917	144905971	[redacted]	-0.333719	[redacted]
chr16	8790532	8790636	[redacted]	-0.333712	[redacted]
chr17	66225084	66225116	[redacted]	-0.33367	[redacted]

chr6	12335662	12335710	[redacted]	-0.333666	[redacted]
chr5	65334587	65334633	[redacted]	-0.333652	[redacted]
chr3	13749908	13749926	[redacted]	-0.333636	[redacted]
chr6	4701707	4701779	[redacted]	-0.333623	[redacted]
chrX	12857767	12857772	[redacted]	-0.333622	[redacted]
chr10	23900604	23900662	[redacted]	-0.333614	[redacted]
chr11	34054430	34054474	[redacted]	-0.333607	[redacted]
chr1	57278088	57278141	[redacted]	-0.333605	[redacted]
chrX	52415114	52415139	[redacted]	-0.333574	[redacted]
chr2	16437328	16437387	[redacted]	-0.33357	[redacted]
chr10	117370071	117370103	[redacted]	-0.33357	[redacted]
chr9	28790752	28790812	[redacted]	-0.333544	[redacted]
chr9	15132562	15132619	[redacted]	-0.333544	[redacted]
chr1	189804887	189804944	[redacted]	-0.333543	[redacted]
chr5	105384278	105384348	[redacted]	-0.333542	[redacted]
chr17	42300402	42300529	[redacted]	-0.333538	[redacted]
chr11	87480613	87480654	[redacted]	-0.333526	[redacted]
chr7	118814109	118814188	[redacted]	-0.33351	[redacted]
chr17	78176395	78176461	[redacted]	-0.3335	[redacted]
chr14	98585635	98585668	[redacted]	-0.333491	[redacted]
chr10	64040779	64040810	[redacted]	-0.333475	[redacted]
chr9	49657124	49657187	[redacted]	-0.333474	[redacted]
chr6	49782592	49782655	[redacted]	-0.333474	[redacted]
chr11	67255240	67255316	[redacted]	-0.333456	[redacted]
chr10	121822912	121823004	[redacted]	-0.333451	[redacted]
chr7	6996549	6996619	[redacted]	-0.333446	[redacted]
chr5	4150648	4150683	[redacted]	-0.333444	[redacted]
chr5	73380935	73380976	[redacted]	-0.333444	[redacted]
chr1	185437021	185437048	[redacted]	-0.33343	[redacted]
chr13	111894659	111894705	[redacted]	-0.333408	[redacted]
chr14	47522515	47522564	[redacted]	-0.333394	[redacted]
chr17	80275133	80275164	[redacted]	-0.33338	[redacted]
chr4	21472595	21472638	[redacted]	-0.333372	[redacted]
chr17	80312359	80312437	[redacted]	-0.333354	[redacted]
chr7	87732460	87732530	[redacted]	-0.333349	[redacted]
chr13	8860054	8860078	[redacted]	-0.333338	[redacted]
chr6	30022586	30022639	[redacted]	-0.333318	[redacted]
chr10	119699015	119699081	[redacted]	-0.333316	[redacted]
chr8	111843727	111843784	[redacted]	-0.333316	[redacted]
chr3	54737354	54737409	[redacted]	-0.333302	[redacted]
chr10	6888821	6888878	[redacted]	-0.333269	[redacted]
chr9	88530696	88530745	[redacted]	-0.333266	[redacted]
chr7	79943937	79944033	[redacted]	-0.333259	[redacted]

chr1	194412401	194412461	[redacted]	-0.333242	[redacted]
chr13	19767783	19767849	[redacted]	-0.333241	[redacted]
chr9	37048670	37048757	[redacted]	-0.333229	[redacted]
chr18	12321706	12321775	[redacted]	-0.333229	[redacted]
chr12	14485210	14485291	[redacted]	-0.333226	[redacted]
chr10	12482152	12482246	[redacted]	-0.33322	[redacted]
chr9	37105628	37105677	[redacted]	-0.333217	[redacted]
chr14	51971911	51971935	[redacted]	-0.333213	[redacted]
chr3	86833205	86833229	[redacted]	-0.333207	[redacted]
chr1	136630893	136630916	[redacted]	-0.333204	[redacted]
chr9	81765436	81765503	[redacted]	-0.333199	[redacted]
chr11	24152223	24152278	[redacted]	-0.333155	[redacted]
chr11	69488089	69488205	[redacted]	-0.333145	[redacted]
chr6	37245397	37245473	[redacted]	-0.333144	[redacted]
chr14	36544041	36544057	[redacted]	-0.33312	[redacted]
chr9	99798300	99798360	[redacted]	-0.333116	[redacted]
chr12	46812204	46812246	[redacted]	-0.333113	[redacted]
chr16	22729636	22729675	[redacted]	-0.333088	[redacted]
chr6	118989424	118989448	[redacted]	-0.333075	[redacted]
chr2	144422474	144422510	[redacted]	-0.333071	[redacted]
chr6	22472444	22472491	[redacted]	-0.333067	[redacted]
chr10	42698120	42698165	[redacted]	-0.333017	[redacted]
chr6	48331751	48331822	[redacted]	-0.332984	[redacted]
chr11	22149229	22149302	[redacted]	-0.332971	[redacted]
chr18	78617979	78618036	[redacted]	-0.332947	[redacted]
chr9	118365659	118365717	[redacted]	-0.332938	[redacted]
chr2	49453795	49453812	[redacted]	-0.332927	[redacted]
chr14	79425633	79425664	[redacted]	-0.332925	[redacted]
chr3	88105078	88105164	[redacted]	-0.332921	[redacted]
chr14	54352175	54352315	[redacted]	-0.332914	[redacted]
chr4	10701216	10701261	[redacted]	-0.332913	[redacted]
chr6	134799511	134799553	[redacted]	-0.332904	[redacted]
chr17	31884135	31884184	[redacted]	-0.33289	[redacted]
chr15	51584998	51585152	[redacted]	-0.332874	[redacted]
chr17	15518230	15518284	[redacted]	-0.332872	[redacted]
chr15	51915985	51916045	[redacted]	-0.33287	[redacted]
chr18	36507912	36508007	[redacted]	-0.332864	[redacted]
chr11	68440040	68440117	[redacted]	-0.332856	[redacted]
chr5	138990163	138990215	[redacted]	-0.332852	[redacted]
chr6	107551682	107551743	[redacted]	-0.332851	[redacted]
chr7	37631629	37631688	[redacted]	-0.33285	[redacted]
chr8	24088567	24088632	[redacted]	-0.332848	[redacted]
chr13	19110024	19110081	[redacted]	-0.33282	[redacted]

chr2	160645322	160645386	[redacted]	-0.332818	[redacted]
chr19	14466605	14466686	[redacted]	-0.332797	[redacted]
chr5	99515120	99515188	[redacted]	-0.332794	[redacted]
chr6	144139880	144139906	[redacted]	-0.332794	[redacted]
chr6	53014249	53014303	[redacted]	-0.332784	[redacted]
chr9	32528882	32528923	[redacted]	-0.332769	[redacted]
chr3	81233267	81233316	[redacted]	-0.332756	[redacted]
chr18	69747387	69747430	[redacted]	-0.332744	[redacted]
chr13	46798577	46798725	[redacted]	-0.332736	[redacted]
chr17	6228792	6228852	[redacted]	-0.332719	[redacted]
chr15	81166057	81166137	[redacted]	-0.332704	[redacted]
chr1	130802189	130802290	[redacted]	-0.332696	[redacted]
chr13	20374610	20374668	[redacted]	-0.332696	[redacted]
chr9	95310861	95310927	[redacted]	-0.332686	[redacted]
chr3	153936100	153936174	[redacted]	-0.332684	[redacted]
chr8	124151141	124151190	[redacted]	-0.33268	[redacted]
chr19	49751118	49751175	[redacted]	-0.332678	[redacted]
chr6	128667161	128667301	[redacted]	-0.332674	[redacted]
chr1	100017873	100017974	[redacted]	-0.332668	[redacted]
chr1	34446667	34446766	[redacted]	-0.332666	[redacted]
chr14	100646064	100646139	[redacted]	-0.33266	[redacted]
chr15	54826861	54826886	[redacted]	-0.33265	[redacted]
chr19	38814280	38814293	[redacted]	-0.332647	[redacted]
chr19	17671614	17671664	[redacted]	-0.33264	[redacted]
chr11	112079377	112079429	[redacted]	-0.332638	[redacted]
chr14	15109036	15109118	[redacted]	-0.332631	[redacted]
chr1	182823977	182824051	[redacted]	-0.332583	[redacted]
chr4	119243376	119243423	[redacted]	-0.33258	[redacted]
chr17	30916902	30916940	[redacted]	-0.332576	[redacted]
chr13	24721450	24721508	[redacted]	-0.332537	[redacted]
chr18	10611790	10611826	[redacted]	-0.332515	[redacted]
chr10	12848669	12848721	[redacted]	-0.332498	[redacted]
chr8	94269375	94269412	[redacted]	-0.332498	[redacted]
chr2	59552507	59552569	[redacted]	-0.332497	[redacted]
chr15	66108179	66108240	[redacted]	-0.332491	[redacted]
chr14	18111779	18111844	[redacted]	-0.332477	[redacted]
chr2	125851137	125851203	[redacted]	-0.332477	[redacted]
chr4	63041715	63041786	[redacted]	-0.332472	[redacted]
chr6	87785632	87785745	[redacted]	-0.33247	[redacted]
chr4	41257193	41257320	[redacted]	-0.332469	[redacted]
chr1	73199476	73199509	[redacted]	-0.332466	[redacted]
chr4	54954338	54954423	[redacted]	-0.332465	[redacted]
chr16	70320175	70320237	[redacted]	-0.332458	[redacted]

chr3	15238313	15238423	[redacted]	-0.332435	[redacted]
chr8	6306021	6306071	[redacted]	-0.332423	[redacted]
chr15	3404054	3404140	[redacted]	-0.332411	[redacted]
chr3	39992246	39992310	[redacted]	-0.332382	[redacted]
chr14	72887051	72887110	[redacted]	-0.332378	[redacted]
chr1	55601083	55601186	[redacted]	-0.332368	[redacted]
chr13	102499178	102499225	[redacted]	-0.332361	[redacted]
chr2	160221446	160221515	[redacted]	-0.332353	[redacted]
chr15	37141058	37141124	[redacted]	-0.332352	[redacted]
chr19	46494993	46495029	[redacted]	-0.332341	[redacted]
chr9	58250528	58250572	[redacted]	-0.33234	[redacted]
chr3	142899511	142899554	[redacted]	-0.33234	[redacted]
chr4	123181101	123181171	[redacted]	-0.332335	[redacted]
chr17	12384741	12384819	[redacted]	-0.332328	[redacted]
chr7	29268873	29268892	[redacted]	-0.332325	[redacted]
chr9	60463094	60463158	[redacted]	-0.332322	[redacted]
chr8	91259692	91259762	[redacted]	-0.33232	[redacted]
chr11	46712406	46712494	[redacted]	-0.332308	[redacted]
chr5	122264971	122265048	[redacted]	-0.332305	[redacted]
chr10	96482879	96482945	[redacted]	-0.332299	[redacted]
chr8	79445765	79445852	[redacted]	-0.332289	[redacted]
chr13	97407474	97407536	[redacted]	-0.332285	[redacted]
chr15	7076674	7076755	[redacted]	-0.332278	[redacted]
chr2	114822837	114822924	[redacted]	-0.332262	[redacted]
chr8	46707385	46707458	[redacted]	-0.33225	[redacted]
chr10	93796633	93796659	[redacted]	-0.332238	[redacted]
chr1	132948215	132948269	[redacted]	-0.332229	[redacted]
chr4	40402369	40402449	[redacted]	-0.33221	[redacted]
chr6	90724128	90724199	[redacted]	-0.332203	[redacted]
chr4	28905485	28905523	[redacted]	-0.332193	[redacted]
chr19	36121142	36121220	[redacted]	-0.332183	[redacted]
chr15	44768587	44768648	[redacted]	-0.332175	[redacted]
chr14	54991715	54991784	[redacted]	-0.332164	[redacted]
chr15	85816171	85816257	[redacted]	-0.332163	[redacted]
chrX	17750687	17750708	[redacted]	-0.332147	[redacted]
chr19	18668341	18668520	[redacted]	-0.33214	[redacted]
chr9	120255805	120255892	[redacted]	-0.332125	[redacted]
chr15	12228488	12228574	[redacted]	-0.332115	[redacted]
chr10	40722127	40722207	[redacted]	-0.332107	[redacted]
chr2	81803121	81803172	[redacted]	-0.33207	[redacted]
chr16	35743511	35743563	[redacted]	-0.332057	[redacted]
chr13	42607289	42607335	[redacted]	-0.332047	[redacted]
chr9	31272224	31272295	[redacted]	-0.332032	[redacted]

chr11	51425886	51425917	-0.332011
chr3	103308228	103308300	-0.332
chr7	64961581	64961639	-0.331995
chr13	17861318	17861381	-0.331972
chr15	92490331	92490375	-0.331968
chr7	78537131	78537186	-0.331902
chr5	57785026	57785062	-0.331901
chr13	96309622	96309748	-0.331897
chr2	32038426	32038524	-0.331892
chr4	32464912	32464953	-0.331885
chr10	4821320	4821412	-0.331878
chr1	155454322	155454369	-0.331875
chr9	104493057	104493141	-0.331844
chr2	45991448	45991508	-0.331834
chr9	46588194	46588325	-0.331825
chr8	91249610	91249705	-0.331821
chr5	81966419	81966474	-0.33181
chr19	60286735	60286755	-0.331806
chr16	8902073	8902132	-0.331805
chr18	85174473	85174532	-0.331797
chr4	55591235	55591301	-0.331786
chr11	71987135	71987240	-0.331784
chr9	117765385	117765448	-0.331778
chr12	51151303	51151337	-0.331744
chr9	66852913	66852963	-0.331711
chr10	83805995	83806017	-0.331708
chr3	28857582	28857625	-0.331682
chr3	149045051	149045100	-0.331676
chr8	121706396	121706445	-0.331676
chr17	94711247	94711326	-0.331662
chr12	69435362	69435394	-0.331662
chr6	38950495	38950567	-0.331658
chr15	17926290	17926346	-0.331657
chr11	65989210	65989263	-0.331656
chr19	25292134	25292278	-0.331655
chr1	85774749	85774858	-0.331644
chr14	94306017	94306066	-0.331634
chr9	59545050	59545119	-0.33163
chr11	52420450	52420538	-0.331619
chr4	98062306	98062316	-0.331608
chr17	86960468	86960520	-0.331576
chr13	112303214	112303266	-0.331552
chr6	99633639	99633688	-0.331552

chr17	60973925	60974006	[redacted]	-0.331551	[redacted]
chr11	65948132	65948196	[redacted]	-0.331544	[redacted]
chr7	75053452	75053505	[redacted]	-0.331539	[redacted]
chr14	98640884	98640974	[redacted]	-0.331496	[redacted]
chr18	77000550	77000599	[redacted]	-0.331476	[redacted]
chr12	40429189	40429235	[redacted]	-0.331467	[redacted]
chr7	116183545	116183594	[redacted]	-0.331446	[redacted]
chr6	54579623	54579651	[redacted]	-0.331445	[redacted]
chr17	83208362	83208417	[redacted]	-0.331444	[redacted]
chr18	11658854	11658903	[redacted]	-0.331431	[redacted]
chr11	53441660	53441698	[redacted]	-0.331415	[redacted]
chr3	15137401	15137468	[redacted]	-0.331414	[redacted]
chr5	106850619	106850669	[redacted]	-0.331411	[redacted]
chr14	57339511	57339564	[redacted]	-0.331403	[redacted]
chr13	20132110	20132137	[redacted]	-0.331372	[redacted]
chr7	41018289	41018324	[redacted]	-0.331366	[redacted]
chr6	112638955	112639028	[redacted]	-0.331361	[redacted]
chr7	110100563	110100615	[redacted]	-0.331349	[redacted]
chr4	84021667	84021755	[redacted]	-0.331348	[redacted]
chr2	47066832	47066885	[redacted]	-0.331342	[redacted]
chr15	54495897	54495965	[redacted]	-0.331339	[redacted]
chr12	54791302	54791367	[redacted]	-0.331332	[redacted]
chr6	134233404	134233570	[redacted]	-0.331317	[redacted]
chr7	81844412	81844503	[redacted]	-0.331312	[redacted]
chr1	86702929	86702966	[redacted]	-0.331308	[redacted]
chr2	137940525	137940532	[redacted]	-0.331298	[redacted]
chr4	119079998	119080018	[redacted]	-0.331286	[redacted]
chr12	76683770	76683873	[redacted]	-0.331284	[redacted]
chr3	60010549	60010683	[redacted]	-0.331284	[redacted]
chr1	157341747	157341814	[redacted]	-0.331269	[redacted]
chr10	112632773	112632802	[redacted]	-0.331241	[redacted]
chrX	151954890	151954924	[redacted]	-0.331229	[redacted]
chr13	20205133	20205248	[redacted]	-0.331226	[redacted]
chr2	164369625	164369672	[redacted]	-0.331212	[redacted]
chrX	58541102	58541194	[redacted]	-0.331201	[redacted]
chr11	75553969	75554051	[redacted]	-0.331199	[redacted]
chr13	12177273	12177359	[redacted]	-0.331193	[redacted]
chr3	151357876	151357919	[redacted]	-0.331193	[redacted]
chr9	7790717	7790783	[redacted]	-0.331184	[redacted]
chr11	115067276	115067312	[redacted]	-0.331182	[redacted]
chr18	74193756	74193804	[redacted]	-0.331182	[redacted]
chr10	112879410	112879496	[redacted]	-0.331181	[redacted]
chr6	103025538	103025580	[redacted]	-0.331177	[redacted]

chr16	23613306	23613325	[redacted]	-0.331167	[redacted]
chr11	70667635	70667684	[redacted]	-0.331159	[redacted]
chr2	91981182	91981217	[redacted]	-0.33111	[redacted]
chr12	97568461	97568541	[redacted]	-0.331083	[redacted]
chr2	70809831	70809876	[redacted]	-0.331068	[redacted]
chr8	58575164	58575244	[redacted]	-0.331066	[redacted]
chr5	98238523	98238554	[redacted]	-0.331042	[redacted]
chr11	115442829	115442846	[redacted]	-0.331023	[redacted]
chr13	98872196	98872295	[redacted]	-0.331008	[redacted]
chr2	93517722	93517782	[redacted]	-0.331	[redacted]
chr15	101875672	101875760	[redacted]	-0.330989	[redacted]
chr10	97607660	97607703	[redacted]	-0.330932	[redacted]
chr10	124288886	124288969	[redacted]	-0.330923	[redacted]
chr15	98600483	98600513	[redacted]	-0.33092	[redacted]
chr7	130488160	130488206	[redacted]	-0.330912	[redacted]
chr17	61602095	61602122	[redacted]	-0.330906	[redacted]
chr1	184760674	184760754	[redacted]	-0.330892	[redacted]
chr2	75596500	75596586	[redacted]	-0.330882	[redacted]
chr6	139946392	139946453	[redacted]	-0.330878	[redacted]
chr18	70459852	70459974	[redacted]	-0.330871	[redacted]
chr9	44576371	44576424	[redacted]	-0.330867	[redacted]
chr2	103494517	103494528	[redacted]	-0.330858	[redacted]
chr13	110151303	110151312	[redacted]	-0.330841	[redacted]
chr8	33691037	33691142	[redacted]	-0.330837	[redacted]
chr15	81315173	81315210	[redacted]	-0.330833	[redacted]
chr1	133571668	133571733	[redacted]	-0.330831	[redacted]
chr9	118382137	118382183	[redacted]	-0.330825	[redacted]
chr11	101864874	101864892	[redacted]	-0.33081	[redacted]
chr18	74828588	74828674	[redacted]	-0.330809	[redacted]
chr15	36776273	36776335	[redacted]	-0.330779	[redacted]
chr14	118187062	118187077	[redacted]	-0.33077	[redacted]
chr13	117857967	117858022	[redacted]	-0.33077	[redacted]
chr10	118653839	118653870	[redacted]	-0.330735	[redacted]
chr2	102462218	102462267	[redacted]	-0.330732	[redacted]
chr11	101229562	101229679	[redacted]	-0.330728	[redacted]
chr11	45916880	45916926	[redacted]	-0.330718	[redacted]
chr15	7458731	7458785	[redacted]	-0.330716	[redacted]
chr14	27365742	27365822	[redacted]	-0.330713	[redacted]
chr2	164244714	164244742	[redacted]	-0.330711	[redacted]
chr15	50650097	50650176	[redacted]	-0.330705	[redacted]
chr12	90254761	90254794	[redacted]	-0.3307	[redacted]
chr14	76451341	76451418	[redacted]	-0.3307	[redacted]
chr9	72660513	72660587	[redacted]	-0.33069	[redacted]

chr2	173722306	173722369	[redacted]	-0.330678	[redacted]
chr8	109523849	109523908	[redacted]	-0.330674	[redacted]
chr4	95171989	95172049	[redacted]	-0.330669	[redacted]
chr2	144305648	144305679	[redacted]	-0.330662	[redacted]
chr2	59472599	59472659	[redacted]	-0.330641	[redacted]
chr18	66037782	66037827	[redacted]	-0.330636	[redacted]
chr2	151360778	151360826	[redacted]	-0.330626	[redacted]
chrX	102263080	102263140	[redacted]	-0.33062	[redacted]
chr3	54681322	54681402	[redacted]	-0.330613	[redacted]
chr5	53480679	53480727	[redacted]	-0.330555	[redacted]
chr15	86709015	86709132	[redacted]	-0.330543	[redacted]
chr2	146912779	146912836	[redacted]	-0.330514	[redacted]
chr9	63189612	63189687	[redacted]	-0.330495	[redacted]
chr12	40257900	40257944	[redacted]	-0.330469	[redacted]
chr1	101575457	101575551	[redacted]	-0.330454	[redacted]
chr18	3007284	3007374	[redacted]	-0.330444	[redacted]
chr11	86336019	86336119	[redacted]	-0.330436	[redacted]
chr2	11687993	11688036	[redacted]	-0.330433	[redacted]
chr1	91491512	91491573	[redacted]	-0.330423	[redacted]
chr3	135451974	135452016	[redacted]	-0.330415	[redacted]
chr6	32944006	32944060	[redacted]	-0.330376	[redacted]
chr6	34450317	34450366	[redacted]	-0.330369	[redacted]
chr10	125651791	125651847	[redacted]	-0.33036	[redacted]
chr6	83168718	83168737	[redacted]	-0.330339	[redacted]
chr14	32993777	32993849	[redacted]	-0.330317	[redacted]
chr11	107248038	107248098	[redacted]	-0.330316	[redacted]
chr14	102444400	102444455	[redacted]	-0.33031	[redacted]
chr10	20222731	20222816	[redacted]	-0.330293	[redacted]
chr11	67579446	67579503	[redacted]	-0.330272	[redacted]
chr1	74117547	74117653	[redacted]	-0.330257	[redacted]
chr7	44669772	44669803	[redacted]	-0.330251	[redacted]
chr2	76371285	76371356	[redacted]	-0.330244	[redacted]
chr8	11489932	11489990	[redacted]	-0.330241	[redacted]
chr8	46181899	46181912	[redacted]	-0.33024	[redacted]
chr6	12818623	12818654	[redacted]	-0.330232	[redacted]
chr14	21736879	21736945	[redacted]	-0.330209	[redacted]
chr9	71648501	71648561	[redacted]	-0.330204	[redacted]
chr11	94731367	94731439	[redacted]	-0.330199	[redacted]
chr11	106166033	106166092	[redacted]	-0.330191	[redacted]
chr12	110415820	110415888	[redacted]	-0.330173	[redacted]
chr14	93756797	93756828	[redacted]	-0.330166	[redacted]
chr9	45802516	45802615	[redacted]	-0.330152	[redacted]
chr5	29764428	29764464	[redacted]	-0.330121	[redacted]

chr15	92049081	92049133	[redacted]	-0.33009	[redacted]
chr2	12995090	12995155	[redacted]	-0.330078	[redacted]
chr11	21824633	21824672	[redacted]	-0.330069	[redacted]
chr10	25503385	25503417	[redacted]	-0.330067	[redacted]
chrX	102074373	102074427	[redacted]	-0.330062	[redacted]
chr4	137919766	137919855	[redacted]	-0.33005	[redacted]
chr8	110150706	110150785	[redacted]	-0.330043	[redacted]
chr5	79382275	79382349	[redacted]	-0.330039	[redacted]
chr14	57329709	57329750	[redacted]	-0.330026	[redacted]
chr8	123110092	123110178	[redacted]	-0.330018	[redacted]
chr9	54563282	54563341	[redacted]	-0.330003	[redacted]
chr15	91777896	91777939	[redacted]	-0.329991	[redacted]
chr8	60520308	60520371	[redacted]	-0.329966	[redacted]
chr3	151656016	151656045	[redacted]	-0.329964	[redacted]
chr3	14069172	14069211	[redacted]	-0.329961	[redacted]
chr3	24249305	24249361	[redacted]	-0.329942	[redacted]
chr15	96356644	96356736	[redacted]	-0.329937	[redacted]
chrX	147996829	147996893	[redacted]	-0.329931	[redacted]
chr3	142964851	142964902	[redacted]	-0.329927	[redacted]
chr10	119038687	119038711	[redacted]	-0.329921	[redacted]
chr9	117277867	117277920	[redacted]	-0.329888	[redacted]
chr16	30127982	30128049	[redacted]	-0.329887	[redacted]
chr9	29176494	29176582	[redacted]	-0.32988	[redacted]
chr2	59141687	59141774	[redacted]	-0.329878	[redacted]
chr1	125727670	125727750	[redacted]	-0.329877	[redacted]
chr10	116692101	116692170	[redacted]	-0.32987	[redacted]
chr11	91032441	91032523	[redacted]	-0.329826	[redacted]
chr2	142013474	142013548	[redacted]	-0.329804	[redacted]
chr15	67869650	67869773	[redacted]	-0.329802	[redacted]
chr14	24572411	24572477	[redacted]	-0.329802	[redacted]
chr10	75101888	75101944	[redacted]	-0.329799	[redacted]
chr5	125480106	125480170	[redacted]	-0.329799	[redacted]
chr7	113041858	113041929	[redacted]	-0.329796	[redacted]
chr7	102623873	102623930	[redacted]	-0.329789	[redacted]
chr10	62727867	62727934	[redacted]	-0.329785	[redacted]
chr18	66990537	66990596	[redacted]	-0.329764	[redacted]
chr14	25425787	25425859	[redacted]	-0.329757	[redacted]
chr7	34845121	34845141	[redacted]	-0.329742	[redacted]
chr12	57198799	57198850	[redacted]	-0.329737	[redacted]
chr10	41320527	41320559	[redacted]	-0.329721	[redacted]
chr1	150440031	150440114	[redacted]	-0.329699	[redacted]
chr3	96580609	96580704	[redacted]	-0.329695	[redacted]
chr11	16527967	16528028	[redacted]	-0.329686	[redacted]

chr7	141513123	141513173	[redacted]	-0.329678	[redacted]
chr5	64603492	64603558	[redacted]	-0.329668	[redacted]
chr16	22405440	22405475	[redacted]	-0.329658	[redacted]
chr2	7025654	7025715	[redacted]	-0.329653	[redacted]
chr14	98960512	98960590	[redacted]	-0.329642	[redacted]
chr13	114007690	114007745	[redacted]	-0.329642	[redacted]
chr9	69482870	69482921	[redacted]	-0.329624	[redacted]
chr15	31017759	31017811	[redacted]	-0.329622	[redacted]
chr3	69339981	69340026	[redacted]	-0.329618	[redacted]
chr5	118719948	118720047	[redacted]	-0.329605	[redacted]
chr5	67499279	67499350	[redacted]	-0.329601	[redacted]
chr9	77597354	77597402	[redacted]	-0.329593	[redacted]
chr13	41064667	41064736	[redacted]	-0.329558	[redacted]
chr1	183803130	183803193	[redacted]	-0.329553	[redacted]
chr5	147670949	147670978	[redacted]	-0.329548	[redacted]
chr13	75934783	75934848	[redacted]	-0.329546	[redacted]
chr7	46666991	46667033	[redacted]	-0.329543	[redacted]
chr17	87908555	87908586	[redacted]	-0.329538	[redacted]
chr6	20604549	20604598	[redacted]	-0.329536	[redacted]
chr9	58982378	58982402	[redacted]	-0.329533	[redacted]
chr6	18562101	18562177	[redacted]	-0.329532	[redacted]
chr5	137187559	137187618	[redacted]	-0.32951	[redacted]
chr7	88102850	88102978	[redacted]	-0.329496	[redacted]
chr19	28274989	28275030	[redacted]	-0.32949	[redacted]
chr11	81982420	81982469	[redacted]	-0.329483	[redacted]
chr8	64834420	64834454	[redacted]	-0.329478	[redacted]
chr12	50836810	50836848	[redacted]	-0.329469	[redacted]
chr12	110386666	110386729	[redacted]	-0.329457	[redacted]
chr2	11667251	11667321	[redacted]	-0.329435	[redacted]
chr12	49901239	49901306	[redacted]	-0.329421	[redacted]
chr19	19218594	19218637	[redacted]	-0.329418	[redacted]
chr2	76862299	76862386	[redacted]	-0.329386	[redacted]
chr4	98883612	98883714	[redacted]	-0.329374	[redacted]
chr7	58627168	58627236	[redacted]	-0.329312	[redacted]
chr12	78878228	78878307	[redacted]	-0.329308	[redacted]
chr3	145073626	145073677	[redacted]	-0.329307	[redacted]
chr3	83615202	83615263	[redacted]	-0.329294	[redacted]
chr16	37548815	37548832	[redacted]	-0.329285	[redacted]
chr2	152391250	152391311	[redacted]	-0.32926	[redacted]
chr4	70496263	70496299	[redacted]	-0.329248	[redacted]
chr10	85516554	85516610	[redacted]	-0.329224	[redacted]
chr9	27158972	27159097	[redacted]	-0.329215	[redacted]
chr13	64314479	64314525	[redacted]	-0.32921	[redacted]

chr1	98805668	98805745	[redacted]	-0.329207	[redacted]
chr11	35512079	35512093	[redacted]	-0.329205	[redacted]
chr16	34339359	34339410	[redacted]	-0.329201	[redacted]
chr8	82308580	82308634	[redacted]	-0.329198	[redacted]
chr9	61156670	61156723	[redacted]	-0.329191	[redacted]
chr6	91426258	91426314	[redacted]	-0.329187	[redacted]
chr12	38532348	38532407	[redacted]	-0.329171	[redacted]
chr1	175834652	175834736	[redacted]	-0.329167	[redacted]
chr14	20978248	20978321	[redacted]	-0.32916	[redacted]
chr6	5225514	5225562	[redacted]	-0.329152	[redacted]
chr16	33896809	33896841	[redacted]	-0.329143	[redacted]
chr5	76061807	76061863	[redacted]	-0.329128	[redacted]
chr14	21523681	21523741	[redacted]	-0.329118	[redacted]
chr4	82370337	82370390	[redacted]	-0.329116	[redacted]
chr11	108310487	108310500	[redacted]	-0.329111	[redacted]
chr18	34494219	34494290	[redacted]	-0.32911	[redacted]
chr1	190006848	190006908	[redacted]	-0.329097	[redacted]
chr3	30738444	30738516	[redacted]	-0.329096	[redacted]
chrX	17750459	17750534	[redacted]	-0.329078	[redacted]
chr15	66092276	66092313	[redacted]	-0.329074	[redacted]
chr3	99748408	99748469	[redacted]	-0.32907	[redacted]
chr4	53364396	53364473	[redacted]	-0.329063	[redacted]
chr10	118840482	118840530	[redacted]	-0.329062	[redacted]
chr12	72282071	72282138	[redacted]	-0.329052	[redacted]
chr6	68400373	68400432	[redacted]	-0.329039	[redacted]
chr5	14055714	14055807	[redacted]	-0.329022	[redacted]
chr2	116919421	116919496	[redacted]	-0.329019	[redacted]
chr14	31023106	31023156	[redacted]	-0.329018	[redacted]
chr6	139042029	139042077	[redacted]	-0.329015	[redacted]
chr9	21788624	21788685	[redacted]	-0.328981	[redacted]
chr6	130414232	130414318	[redacted]	-0.328954	[redacted]
chr19	14685063	14685106	[redacted]	-0.328946	[redacted]
chr10	76930497	76930566	[redacted]	-0.328941	[redacted]
chr4	43641571	43641664	[redacted]	-0.328927	[redacted]
chr4	118080721	118080774	[redacted]	-0.328888	[redacted]
chr18	61706538	61706625	[redacted]	-0.328866	[redacted]
chr5	126001807	126001907	[redacted]	-0.328859	[redacted]
chr1	22040689	22040777	[redacted]	-0.328858	[redacted]
chrX	60488100	60488156	[redacted]	-0.328828	[redacted]
chr5	4036943	4036972	[redacted]	-0.328785	[redacted]
chr1	105893891	105893956	[redacted]	-0.328785	[redacted]
chr3	87066275	87066303	[redacted]	-0.328765	[redacted]
chr7	44784539	44784605	[redacted]	-0.328758	[redacted]

chr14	17709967	17710030	[redacted]	-0.328746	[redacted]
chr16	37715153	37715188	[redacted]	-0.32874	[redacted]
chr5	113614193	113614357	[redacted]	-0.32873	[redacted]
chr13	56557649	56557672	[redacted]	-0.328722	[redacted]
chr6	73234441	73234504	[redacted]	-0.328709	[redacted]
chr2	117826481	117826502	[redacted]	-0.328685	[redacted]
chrX	166123805	166123864	[redacted]	-0.328678	[redacted]
chr15	18116756	18116798	[redacted]	-0.32861	[redacted]
chr11	47252166	47252240	[redacted]	-0.328606	[redacted]
chr7	42406475	42406538	[redacted]	-0.328603	[redacted]
chr12	107659870	107659901	[redacted]	-0.328602	[redacted]
chr6	66540429	66540525	[redacted]	-0.328593	[redacted]
chr4	60408593	60408634	[redacted]	-0.328585	[redacted]
chr5	79984418	79984502	[redacted]	-0.328584	[redacted]
chr4	66705850	66705929	[redacted]	-0.328558	[redacted]
chr5	100591772	100591845	[redacted]	-0.328546	[redacted]
chr13	69907479	69907529	[redacted]	-0.328545	[redacted]
chr17	66358938	66359010	[redacted]	-0.328519	[redacted]
chr1	76261468	76261533	[redacted]	-0.328512	[redacted]
chr9	47604021	47604056	[redacted]	-0.328499	[redacted]
chr15	63779909	63779952	[redacted]	-0.328498	[redacted]
chr8	84576249	84576294	[redacted]	-0.328472	[redacted]
chr14	119149349	119149395	[redacted]	-0.328472	[redacted]
chr4	116406622	116406719	[redacted]	-0.328428	[redacted]
chr1	47505220	47505279	[redacted]	-0.32841	[redacted]
chr2	149227583	149227628	[redacted]	-0.328404	[redacted]
chr5	21013335	21013390	[redacted]	-0.328382	[redacted]
chr1	33574738	33574753	[redacted]	-0.328376	[redacted]
chr8	109105426	109105470	[redacted]	-0.328347	[redacted]
chr9	35247348	35247402	[redacted]	-0.328345	[redacted]
chr3	136059394	136059476	[redacted]	-0.328343	[redacted]
chr8	79873703	79873766	[redacted]	-0.328342	[redacted]
chr17	48380477	48380523	[redacted]	-0.328341	[redacted]
chr15	59193429	59193459	[redacted]	-0.328339	[redacted]
chr3	146490080	146490147	[redacted]	-0.32833	[redacted]
chr11	60066802	60066841	[redacted]	-0.328323	[redacted]
chr5	118764355	118764500	[redacted]	-0.328317	[redacted]
chr14	57567345	57567384	[redacted]	-0.328299	[redacted]
chr18	83053367	83053427	[redacted]	-0.328278	[redacted]
chr17	47849407	47849428	[redacted]	-0.328257	[redacted]
chr10	122663504	122663555	[redacted]	-0.328255	[redacted]
chr12	15747379	15747459	[redacted]	-0.328254	[redacted]
chr12	69487127	69487207	[redacted]	-0.328252	[redacted]

chr5	90524835	90524874	[redacted]	-0.328249	[redacted]
chr18	21428656	21428704	[redacted]	-0.32824	[redacted]
chr1	173874360	173874442	[redacted]	-0.328237	[redacted]
chr13	8859845	8859860	[redacted]	-0.328211	[redacted]
chr5	73286041	73286062	[redacted]	-0.328198	[redacted]
chr18	73846665	73846722	[redacted]	-0.328175	[redacted]
chr2	162485722	162485739	[redacted]	-0.328171	[redacted]
chr9	94609990	94610102	[redacted]	-0.328147	[redacted]
chr10	20205437	20205569	[redacted]	-0.328132	[redacted]
chr9	90062500	90062551	[redacted]	-0.328128	[redacted]
chr7	133265618	133265656	[redacted]	-0.328097	[redacted]
chr9	63752221	63752236	[redacted]	-0.328093	[redacted]
chr4	116165946	116166030	[redacted]	-0.328084	[redacted]
chr15	78591202	78591263	[redacted]	-0.32806	[redacted]
chr5	82657363	82657428	[redacted]	-0.328054	[redacted]
chr9	44864850	44864890	[redacted]	-0.328028	[redacted]
chr14	67999049	67999125	[redacted]	-0.328028	[redacted]
chr7	67944520	67944575	[redacted]	-0.328014	[redacted]
chr9	95513277	95513341	[redacted]	-0.328006	[redacted]
chr19	28526137	28526257	[redacted]	-0.327996	[redacted]
chr10	73365040	73365125	[redacted]	-0.327993	[redacted]
chr9	65038889	65038983	[redacted]	-0.327957	[redacted]
chr16	62887511	62887617	[redacted]	-0.327951	[redacted]
chr3	154193465	154193528	[redacted]	-0.32794	[redacted]
chr3	88442588	88442602	[redacted]	-0.327939	[redacted]
chr2	122400314	122400418	[redacted]	-0.327918	[redacted]
chr3	100655187	100655268	[redacted]	-0.327877	[redacted]
chr5	85623164	85623238	[redacted]	-0.327858	[redacted]
chr13	38776801	38776846	[redacted]	-0.327856	[redacted]
chr10	93813881	93813962	[redacted]	-0.327848	[redacted]
chr3	123102651	123102746	[redacted]	-0.327815	[redacted]
chr2	55923097	55923115	[redacted]	-0.327805	[redacted]
chr8	78828699	78828790	[redacted]	-0.327803	[redacted]
chr15	49695849	49695915	[redacted]	-0.327794	[redacted]
chr7	130567380	130567431	[redacted]	-0.327793	[redacted]
chr7	137285665	137285714	[redacted]	-0.327782	[redacted]
chr16	63851876	63851892	[redacted]	-0.327778	[redacted]
chr6	94541888	94541937	[redacted]	-0.327768	[redacted]
chr19	53361901	53361943	[redacted]	-0.327752	[redacted]
chr1	14530244	14530302	[redacted]	-0.327746	[redacted]
chr18	78025828	78025902	[redacted]	-0.327718	[redacted]
chr5	76327208	76327264	[redacted]	-0.3277	[redacted]
chr1	170580934	170580954	[redacted]	-0.327692	[redacted]

chr12	91715420	91715499	[redacted]	-0.327657	[redacted]
chr12	22328715	22328764	[redacted]	-0.327657	[redacted]
chr15	95749316	95749340	[redacted]	-0.327655	[redacted]
chr4	148162922	148162954	[redacted]	-0.327651	[redacted]
chr17	19437798	19437843	[redacted]	-0.327618	[redacted]
chr13	101898134	101898185	[redacted]	-0.327607	[redacted]
chr7	127763964	127764040	[redacted]	-0.327594	[redacted]
chr18	57784481	57784600	[redacted]	-0.32758	[redacted]
chr10	58814945	58815008	[redacted]	-0.327572	[redacted]
chr1	137142070	137142134	[redacted]	-0.327572	[redacted]
chr10	118435106	118435152	[redacted]	-0.327533	[redacted]
chr1	175521185	175521259	[redacted]	-0.327526	[redacted]
chr9	103498526	103498561	[redacted]	-0.327513	[redacted]
chr1	33689916	33689969	[redacted]	-0.327468	[redacted]
chr7	33098614	33098667	[redacted]	-0.327465	[redacted]
chr9	99394258	99394333	[redacted]	-0.327463	[redacted]
chr11	25401863	25401905	[redacted]	-0.327449	[redacted]
chr7	83377522	83377587	[redacted]	-0.327446	[redacted]
chr18	33192830	33192875	[redacted]	-0.327441	[redacted]
chr18	65162540	65162572	[redacted]	-0.327435	[redacted]
chr1	69538089	69538137	[redacted]	-0.327407	[redacted]
chr5	67474023	67474069	[redacted]	-0.327403	[redacted]
chr8	25610237	25610263	[redacted]	-0.327393	[redacted]
chr3	69271310	69271391	[redacted]	-0.32737	[redacted]
chr2	24522900	24522974	[redacted]	-0.32736	[redacted]
chr7	19407536	19407596	[redacted]	-0.32736	[redacted]
chr10	56612131	56612152	[redacted]	-0.327355	[redacted]
chr9	120664314	120664378	[redacted]	-0.327347	[redacted]
chr12	86766214	86766293	[redacted]	-0.327342	[redacted]
chr16	34065210	34065280	[redacted]	-0.327306	[redacted]
chr3	89837658	89837671	[redacted]	-0.327277	[redacted]
chr5	72367880	72367938	[redacted]	-0.327272	[redacted]
chr5	71563612	71563680	[redacted]	-0.327233	[redacted]
chr7	122420614	122420667	[redacted]	-0.327232	[redacted]
chr6	30025863	30025887	[redacted]	-0.327173	[redacted]
chr7	13782083	13782097	[redacted]	-0.327143	[redacted]
chr15	25444400	25444430	[redacted]	-0.327135	[redacted]
chr4	83215465	83215501	[redacted]	-0.327128	[redacted]
chr8	107696808	107696853	[redacted]	-0.327128	[redacted]
chr17	31650157	31650173	[redacted]	-0.327123	[redacted]
chr6	67120948	67121020	[redacted]	-0.327101	[redacted]
chr13	107499013	107499089	[redacted]	-0.327092	[redacted]
chr13	4933514	4933577	[redacted]	-0.327091	[redacted]

chr4	33225117	33225170	[redacted]	-0.327083	[redacted]
chr1	105141305	105141381	[redacted]	-0.327079	[redacted]
chr11	5987297	5987312	[redacted]	-0.327071	[redacted]
chr12	57325408	57325475	[redacted]	-0.327068	[redacted]
chr14	79742364	79742473	[redacted]	-0.327059	[redacted]
chr4	118711409	118711448	[redacted]	-0.327051	[redacted]
chr16	94041061	94041116	[redacted]	-0.327043	[redacted]
chr11	116118334	116118389	[redacted]	-0.32704	[redacted]
chr4	136394740	136394777	[redacted]	-0.327033	[redacted]
chr17	46893961	46894021	[redacted]	-0.327029	[redacted]
chrX	98139521	98139594	[redacted]	-0.327027	[redacted]
chr1	55538604	55538730	[redacted]	-0.32702	[redacted]
chr1	95819616	95819650	[redacted]	-0.327016	[redacted]
chr11	97468230	97468292	[redacted]	-0.327015	[redacted]
chr10	121845399	121845480	[redacted]	-0.327	[redacted]
chr9	32158082	32158153	[redacted]	-0.326996	[redacted]
chr18	7923383	7923517	[redacted]	-0.326986	[redacted]
chr5	145228652	145228739	[redacted]	-0.326975	[redacted]
chr5	45209592	45209630	[redacted]	-0.326974	[redacted]
chr13	46229822	46229871	[redacted]	-0.32696	[redacted]
chr11	106476347	106476404	[redacted]	-0.326958	[redacted]
chr4	120257716	120257745	[redacted]	-0.326912	[redacted]
chr1	150700509	150700553	[redacted]	-0.326874	[redacted]
chr6	81840986	81841080	[redacted]	-0.326866	[redacted]
chr3	19382013	19382080	[redacted]	-0.326865	[redacted]
chr17	15761364	15761427	[redacted]	-0.32686	[redacted]
chr5	51805283	51805352	[redacted]	-0.326803	[redacted]
chr10	14239785	14239806	[redacted]	-0.326797	[redacted]
chr11	76664538	76664565	[redacted]	-0.326778	[redacted]
chr12	3486147	3486198	[redacted]	-0.326734	[redacted]
chr17	65810024	65810047	[redacted]	-0.326709	[redacted]
chr12	106268285	106268347	[redacted]	-0.3267	[redacted]
chr6	88848336	88848445	[redacted]	-0.326686	[redacted]
chr15	42697878	42697953	[redacted]	-0.326663	[redacted]
chr6	108126296	108126384	[redacted]	-0.326657	[redacted]
chr6	71745918	71745933	[redacted]	-0.326644	[redacted]
chr4	8841976	8842044	[redacted]	-0.32664	[redacted]
chr2	54302677	54302735	[redacted]	-0.326633	[redacted]
chr5	30412004	30412065	[redacted]	-0.326629	[redacted]
chr3	121784486	121784513	[redacted]	-0.326621	[redacted]
chr16	45630182	45630220	[redacted]	-0.326615	[redacted]
chr16	32821389	32821439	[redacted]	-0.326612	[redacted]
chrX	38520634	38520710	[redacted]	-0.326605	[redacted]

chr18	47526658	47526678	[redacted]	-0.326604	[redacted]
chr14	86762489	86762552	[redacted]	-0.3266	[redacted]
chr2	23410514	23410566	[redacted]	-0.326546	[redacted]
chr7	27725374	27725404	[redacted]	-0.326544	[redacted]
chr5	67357032	67357056	[redacted]	-0.326542	[redacted]
chr5	104993006	104993083	[redacted]	-0.326523	[redacted]
chr19	54896304	54896375	[redacted]	-0.326493	[redacted]
chr10	26477163	26477280	[redacted]	-0.326487	[redacted]
chr17	30858534	30858560	[redacted]	-0.32648	[redacted]
chr8	108367839	108367902	[redacted]	-0.326471	[redacted]
chr10	86141093	86141106	[redacted]	-0.326466	[redacted]
chr10	40880517	40880622	[redacted]	-0.326457	[redacted]
chr17	3330316	3330381	[redacted]	-0.326452	[redacted]
chr1	186472746	186472776	[redacted]	-0.326404	[redacted]
chr7	97965189	97965306	[redacted]	-0.32638	[redacted]
chr16	34209669	34209743	[redacted]	-0.32638	[redacted]
chr3	131973866	131973886	[redacted]	-0.326366	[redacted]
chr6	45120119	45120172	[redacted]	-0.326363	[redacted]
chr18	84077999	84078055	[redacted]	-0.326357	[redacted]
chr1	83509637	83509670	[redacted]	-0.326342	[redacted]
chr11	6227411	6227483	[redacted]	-0.326338	[redacted]
chr17	31529658	31529699	[redacted]	-0.326332	[redacted]
chr1	121601827	121601891	[redacted]	-0.326305	[redacted]
chr2	60901084	60901152	[redacted]	-0.326289	[redacted]
chr11	98080046	98080083	[redacted]	-0.326267	[redacted]
chr4	121323717	121323796	[redacted]	-0.326252	[redacted]
chr5	114148817	114148857	[redacted]	-0.32624	[redacted]
chr11	19331334	19331417	[redacted]	-0.32623	[redacted]
chr5	63920244	63920304	[redacted]	-0.326222	[redacted]
chr9	7964412	7964488	[redacted]	-0.326216	[redacted]
chr16	50370826	50370884	[redacted]	-0.326215	[redacted]
chr15	25803386	25803439	[redacted]	-0.326204	[redacted]
chr9	36592011	36592117	[redacted]	-0.326184	[redacted]
chr11	105080109	105080187	[redacted]	-0.326181	[redacted]
chr18	11496457	11496540	[redacted]	-0.326179	[redacted]
chr15	96272948	96272982	[redacted]	-0.326172	[redacted]
chr15	98702670	98702774	[redacted]	-0.32617	[redacted]
chr1	53976421	53976455	[redacted]	-0.326155	[redacted]
chr18	33799267	33799328	[redacted]	-0.326149	[redacted]
chr13	75149364	75149405	[redacted]	-0.326144	[redacted]
chr2	71351496	71351532	[redacted]	-0.326129	[redacted]
chr15	45003864	45003920	[redacted]	-0.326128	[redacted]
chr11	78894388	78894454	[redacted]	-0.326113	[redacted]

chr16	55995067	55995126	[redacted]	-0.3261	[redacted]
chr6	124914887	124914951	[redacted]	-0.326096	[redacted]
chr1	80025344	80025428	[redacted]	-0.326092	[redacted]
chr11	78247388	78247416	[redacted]	-0.326078	[redacted]
chr15	94253405	94253452	[redacted]	-0.326073	[redacted]
chr10	77196888	77196951	[redacted]	-0.32607	[redacted]
chr18	29124868	29124886	[redacted]	-0.326063	[redacted]
chr10	79498253	79498295	[redacted]	-0.326062	[redacted]
chr7	76912567	76912619	[redacted]	-0.326062	[redacted]
chr15	61405013	61405066	[redacted]	-0.326048	[redacted]
chr10	19887089	19887154	[redacted]	-0.326046	[redacted]
chr10	13237883	13237907	[redacted]	-0.326045	[redacted]
chr12	17091180	17091197	[redacted]	-0.326041	[redacted]
chr9	75817459	75817527	[redacted]	-0.326034	[redacted]
chr13	100847192	100847235	[redacted]	-0.326004	[redacted]
chr10	76209993	76210013	[redacted]	-0.325997	[redacted]
chr10	39351833	39351896	[redacted]	-0.325995	[redacted]
chr1	88901490	88901526	[redacted]	-0.32595	[redacted]
chr13	55701751	55701808	[redacted]	-0.325947	[redacted]
chr5	147524482	147524518	[redacted]	-0.325927	[redacted]
chr17	10749254	10749338	[redacted]	-0.325902	[redacted]
chr5	67188533	67188569	[redacted]	-0.3259	[redacted]
chr4	150557143	150557152	[redacted]	-0.325889	[redacted]
chr11	108444831	108444903	[redacted]	-0.325887	[redacted]
chr7	120601483	120601541	[redacted]	-0.325884	[redacted]
chr8	105190684	105190756	[redacted]	-0.325865	[redacted]
chr4	69476278	69476359	[redacted]	-0.325836	[redacted]
chr13	101746997	101747046	[redacted]	-0.325821	[redacted]
chr1	192765069	192765126	[redacted]	-0.325817	[redacted]
chr15	73552235	73552309	[redacted]	-0.325816	[redacted]
chr1	163570965	163570994	[redacted]	-0.32581	[redacted]
chr7	43255211	43255277	[redacted]	-0.32581	[redacted]
chr9	57081102	57081141	[redacted]	-0.325802	[redacted]
chr4	4463130	4463196	[redacted]	-0.3258	[redacted]
chr8	31973050	31973091	[redacted]	-0.325796	[redacted]
chrX	104993807	104993901	[redacted]	-0.325795	[redacted]
chr3	79387712	79387744	[redacted]	-0.325793	[redacted]
chr16	49351331	49351376	[redacted]	-0.325781	[redacted]
chr9	31350329	31350412	[redacted]	-0.32576	[redacted]
chr17	85049405	85049461	[redacted]	-0.325757	[redacted]
chr1	13807290	13807321	[redacted]	-0.325756	[redacted]
chr17	65601034	65601106	[redacted]	-0.325744	[redacted]
chr18	23277538	23277585	[redacted]	-0.325742	[redacted]

chr4	125887251	125887338	[redacted]	-0.325742	[redacted]
chr11	106618021	106618090	[redacted]	-0.325732	[redacted]
chr16	26325525	26325564	[redacted]	-0.325718	[redacted]
chr8	89348282	89348335	[redacted]	-0.325715	[redacted]
chr10	79784090	79784112	[redacted]	-0.325704	[redacted]
chr4	118283070	118283152	[redacted]	-0.325698	[redacted]
chr12	111472399	111472454	[redacted]	-0.325692	[redacted]
chr6	100694045	100694117	[redacted]	-0.325691	[redacted]
chr14	20243042	20243105	[redacted]	-0.325688	[redacted]
chr6	115803456	115803488	[redacted]	-0.325683	[redacted]
chr13	97984305	97984334	[redacted]	-0.325683	[redacted]
chr13	119686185	119686251	[redacted]	-0.325661	[redacted]
chr14	76592036	76592094	[redacted]	-0.325655	[redacted]
chr10	108147342	108147404	[redacted]	-0.325652	[redacted]
chr6	129396237	129396349	[redacted]	-0.325644	[redacted]
chr3	52862516	52862617	[redacted]	-0.32564	[redacted]
chr13	44272567	44272622	[redacted]	-0.325632	[redacted]
chr3	46525119	46525198	[redacted]	-0.325624	[redacted]
chr10	118191105	118191135	[redacted]	-0.325619	[redacted]
chr11	96370823	96370882	[redacted]	-0.325606	[redacted]
chr10	10138364	10138409	[redacted]	-0.325601	[redacted]
chr7	30415637	30415684	[redacted]	-0.325594	[redacted]
chr10	54410239	54410295	[redacted]	-0.325572	[redacted]
chr12	73636831	73636928	[redacted]	-0.325571	[redacted]
chr2	169797525	169797578	[redacted]	-0.325571	[redacted]
chrX	9380114	9380187	[redacted]	-0.325559	[redacted]
chr17	79872691	79872778	[redacted]	-0.325557	[redacted]
chr9	32788720	32788860	[redacted]	-0.325557	[redacted]
chr2	28638503	28638561	[redacted]	-0.325555	[redacted]
chr15	8458059	8458125	[redacted]	-0.325511	[redacted]
chr9	72712026	72712082	[redacted]	-0.325474	[redacted]
chr18	53676761	53676811	[redacted]	-0.325474	[redacted]
chr13	118169417	118169470	[redacted]	-0.325472	[redacted]
chr9	109753297	109753354	[redacted]	-0.325467	[redacted]
chr2	73094772	73094844	[redacted]	-0.325451	[redacted]
chr15	75142684	75142735	[redacted]	-0.325437	[redacted]
chr15	79996652	79996706	[redacted]	-0.325437	[redacted]
chr12	119751190	119751266	[redacted]	-0.32542	[redacted]
chr9	114451056	114451092	[redacted]	-0.325413	[redacted]
chr10	11077470	11077519	[redacted]	-0.325407	[redacted]
chr7	129020508	129020525	[redacted]	-0.325403	[redacted]
chr8	123358927	123359019	[redacted]	-0.325402	[redacted]
chr1	187044731	187044782	[redacted]	-0.325392	[redacted]

chr8	81946910	81946962	[redacted]	-0.325366	[redacted]
chr11	41113894	41113945	[redacted]	-0.325354	[redacted]
chr15	56458940	56459001	[redacted]	-0.325341	[redacted]
chr8	119905237	119905303	[redacted]	-0.325327	[redacted]
chr9	116043215	116043303	[redacted]	-0.325299	[redacted]
chr4	129824284	129824381	[redacted]	-0.325297	[redacted]
chr8	11079760	11079801	[redacted]	-0.325286	[redacted]
chr6	121086870	121086980	[redacted]	-0.325274	[redacted]
chr3	126222012	126222091	[redacted]	-0.325269	[redacted]
chr17	34193187	34193229	[redacted]	-0.32526	[redacted]
chr3	53590936	53591005	[redacted]	-0.325259	[redacted]
chr3	146300380	146300438	[redacted]	-0.325249	[redacted]
chr13	43400932	43400961	[redacted]	-0.32523	[redacted]
chr10	126968496	126968548	[redacted]	-0.325227	[redacted]
chr5	49269562	49269657	[redacted]	-0.32522	[redacted]
chr15	90031562	90031638	[redacted]	-0.325203	[redacted]
chrX	94365173	94365209	[redacted]	-0.325181	[redacted]
chr3	56901934	56902021	[redacted]	-0.325178	[redacted]
chr11	91286117	91286186	[redacted]	-0.325177	[redacted]
chr4	32327334	32327379	[redacted]	-0.325167	[redacted]
chr13	55649042	55649058	[redacted]	-0.325141	[redacted]
chr5	81727589	81727656	[redacted]	-0.325141	[redacted]
chr14	102713048	102713149	[redacted]	-0.325134	[redacted]
chr6	123954184	123954256	[redacted]	-0.32513	[redacted]
chr1	94753345	94753412	[redacted]	-0.325114	[redacted]
chr12	118378193	118378254	[redacted]	-0.325114	[redacted]
chr1	76330763	76330840	[redacted]	-0.325103	[redacted]
chr16	58029693	58029752	[redacted]	-0.3251	[redacted]
chr6	17273192	17273249	[redacted]	-0.325098	[redacted]
chr16	95225127	95225219	[redacted]	-0.325096	[redacted]
chr6	14792629	14792688	[redacted]	-0.325084	[redacted]
chr8	60236530	60236599	[redacted]	-0.325082	[redacted]
chr1	156351910	156352031	[redacted]	-0.325069	[redacted]
chr7	129056526	129056559	[redacted]	-0.325059	[redacted]
chr8	81846669	81846716	[redacted]	-0.325047	[redacted]
chr17	14920554	14920590	[redacted]	-0.325043	[redacted]
chr8	64847079	64847150	[redacted]	-0.325038	[redacted]
chr6	49071921	49071944	[redacted]	-0.325026	[redacted]
chr10	4353602	4353689	[redacted]	-0.325024	[redacted]
chr19	58495530	58495598	[redacted]	-0.325022	[redacted]
chr10	94021312	94021325	[redacted]	-0.325013	[redacted]
chr11	83551300	83551341	[redacted]	-0.324991	[redacted]
chrX	50844924	50844961	[redacted]	-0.324974	[redacted]

chr13	15932360	15932420	[redacted]	-0.324961	[redacted]
chr1	106073438	106073473	[redacted]	-0.324955	[redacted]
chr10	71361552	71361607	[redacted]	-0.324929	[redacted]
chr17	84761772	84761892	[redacted]	-0.324921	[redacted]
chr9	109520761	109520831	[redacted]	-0.324908	[redacted]
chr15	35129151	35129196	[redacted]	-0.324907	[redacted]
chr16	31682589	31682610	[redacted]	-0.324896	[redacted]
chr5	54100854	54100885	[redacted]	-0.324882	[redacted]
chr1	179561743	179561798	[redacted]	-0.324858	[redacted]
chr3	8611740	8611789	[redacted]	-0.324847	[redacted]
chr11	57019643	57019803	[redacted]	-0.324815	[redacted]
chr15	50430408	50430467	[redacted]	-0.324815	[redacted]
chr4	136084057	136084106	[redacted]	-0.324808	[redacted]
chr12	15020695	15020773	[redacted]	-0.324802	[redacted]
chr3	99695993	99696044	[redacted]	-0.324794	[redacted]
chr2	174535539	174535612	[redacted]	-0.324791	[redacted]
chr5	54088268	54088341	[redacted]	-0.324744	[redacted]
chr1	83250777	83250825	[redacted]	-0.324735	[redacted]
chrX	94071569	94071601	[redacted]	-0.324732	[redacted]
chr8	73001049	73001148	[redacted]	-0.324714	[redacted]
chr6	136528971	136529095	[redacted]	-0.324702	[redacted]
chr5	54126866	54126914	[redacted]	-0.324693	[redacted]
chr19	25243931	25244002	[redacted]	-0.32469	[redacted]
chr7	28838315	28838395	[redacted]	-0.324669	[redacted]
chr15	92759078	92759152	[redacted]	-0.324662	[redacted]
chr9	103746721	103746781	[redacted]	-0.324657	[redacted]
chr3	60991884	60991935	[redacted]	-0.324644	[redacted]
chr10	122142098	122142142	[redacted]	-0.324631	[redacted]
chr3	103048389	103048484	[redacted]	-0.324626	[redacted]
chr17	10216359	10216396	[redacted]	-0.324623	[redacted]
chr3	137948848	137948903	[redacted]	-0.324619	[redacted]
chr3	138594208	138594246	[redacted]	-0.324596	[redacted]
chr8	11487750	11487860	[redacted]	-0.324573	[redacted]
chr17	10249941	10249954	[redacted]	-0.324568	[redacted]
chr10	84220028	84220098	[redacted]	-0.32453	[redacted]
chr13	70596400	70596428	[redacted]	-0.324519	[redacted]
chr2	9204721	9204843	[redacted]	-0.324516	[redacted]
chr10	83918635	83918711	[redacted]	-0.324515	[redacted]
chr8	68319253	68319307	[redacted]	-0.324482	[redacted]
chr16	49349905	49349991	[redacted]	-0.324479	[redacted]
chr14	104427418	104427496	[redacted]	-0.324453	[redacted]
chr3	143121340	143121408	[redacted]	-0.324431	[redacted]
chr5	139511502	139511556	[redacted]	-0.324416	[redacted]

chr10	122808517	122808607	[redacted]	-0.324412	[redacted]
chr3	129546475	129546500	[redacted]	-0.324412	[redacted]
chr6	29899567	29899627	[redacted]	-0.324411	[redacted]
chr1	187545221	187545286	[redacted]	-0.324409	[redacted]
chr3	99667327	99667365	[redacted]	-0.324385	[redacted]
chrX	57152467	57152487	[redacted]	-0.324381	[redacted]
chr17	89302962	89302984	[redacted]	-0.324319	[redacted]
chr16	9356262	9356360	[redacted]	-0.324316	[redacted]
chr4	4095368	4095460	[redacted]	-0.324304	[redacted]
chr9	42739454	42739494	[redacted]	-0.324303	[redacted]
chr2	118145482	118145554	[redacted]	-0.324301	[redacted]
chr14	56325605	56325665	[redacted]	-0.3243	[redacted]
chr3	142477344	142477395	[redacted]	-0.32429	[redacted]
chr13	8985113	8985145	[redacted]	-0.324283	[redacted]
chr14	57282111	57282174	[redacted]	-0.324262	[redacted]
chr6	143323530	143323575	[redacted]	-0.32426	[redacted]
chr1	150608421	150608486	[redacted]	-0.324254	[redacted]
chr12	85050949	85050978	[redacted]	-0.324253	[redacted]
chr15	11990178	11990231	[redacted]	-0.324245	[redacted]
chr6	53402415	53402426	[redacted]	-0.324244	[redacted]
chr1	190255372	190255456	[redacted]	-0.324232	[redacted]
chr1	30691052	30691112	[redacted]	-0.324216	[redacted]
chr9	122364972	122365033	[redacted]	-0.3242	[redacted]
chr18	77658242	77658285	[redacted]	-0.324152	[redacted]
chr8	34969937	34970028	[redacted]	-0.32415	[redacted]
chr3	30516011	30516080	[redacted]	-0.324139	[redacted]
chr18	77652566	77652647	[redacted]	-0.324116	[redacted]
chr3	69243898	69243936	[redacted]	-0.324113	[redacted]
chr5	81119665	81119724	[redacted]	-0.324091	[redacted]
chr15	59742203	59742263	[redacted]	-0.32409	[redacted]
chr1	183357925	183357940	[redacted]	-0.324081	[redacted]
chr5	17687358	17687428	[redacted]	-0.324081	[redacted]
chr16	78322404	78322433	[redacted]	-0.324071	[redacted]
chr14	75803687	75803747	[redacted]	-0.324063	[redacted]
chr7	127262348	127262431	[redacted]	-0.324054	[redacted]
chr15	84729003	84729081	[redacted]	-0.324044	[redacted]
chr16	22874077	22874131	[redacted]	-0.324043	[redacted]
chr17	64721853	64721877	[redacted]	-0.324041	[redacted]
chr16	72956980	72957057	[redacted]	-0.324036	[redacted]
chr15	28081919	28081954	[redacted]	-0.324027	[redacted]
chr16	38358881	38358907	[redacted]	-0.324023	[redacted]
chr7	56840096	56840160	[redacted]	-0.32402	[redacted]
chr14	37113793	37113883	[redacted]	-0.324002	[redacted]

chr18	8087131	8087173	[redacted]	-0.32399	[redacted]
chr19	8709480	8709503	[redacted]	-0.323983	[redacted]
chr6	10690452	10690497	[redacted]	-0.323975	[redacted]
chr10	19971780	19971860	[redacted]	-0.323962	[redacted]
chr11	19193080	19193153	[redacted]	-0.323946	[redacted]
chr13	101450255	101450325	[redacted]	-0.323938	[redacted]
chr2	152453261	152453325	[redacted]	-0.323931	[redacted]
chr10	9827021	9827046	[redacted]	-0.323909	[redacted]
chr14	62206938	62207003	[redacted]	-0.323906	[redacted]
chr4	63548351	63548417	[redacted]	-0.323846	[redacted]
chr13	3828514	3828546	[redacted]	-0.323832	[redacted]
chr8	87100409	87100451	[redacted]	-0.323831	[redacted]
chr9	117412053	117412080	[redacted]	-0.323806	[redacted]
chr1	57764636	57764697	[redacted]	-0.323794	[redacted]
chr8	51128833	51128874	[redacted]	-0.323791	[redacted]
chr4	86519702	86519765	[redacted]	-0.323789	[redacted]
chr6	87640874	87640920	[redacted]	-0.323775	[redacted]
chr4	116184621	116184675	[redacted]	-0.323774	[redacted]
chr6	97386812	97386891	[redacted]	-0.323753	[redacted]
chr17	30940919	30940959	[redacted]	-0.323751	[redacted]
chr17	16006484	16006547	[redacted]	-0.32374	[redacted]
chr13	58397152	58397214	[redacted]	-0.32372	[redacted]
chr17	72801709	72801759	[redacted]	-0.323711	[redacted]
chr11	68298210	68298269	[redacted]	-0.32371	[redacted]
chr19	37022716	37022771	[redacted]	-0.323695	[redacted]
chr4	8337082	8337116	[redacted]	-0.323691	[redacted]
chr9	56350388	56350417	[redacted]	-0.323687	[redacted]
chr5	124219545	124219711	[redacted]	-0.323684	[redacted]
chr6	124858328	124858391	[redacted]	-0.323682	[redacted]
chr15	58281399	58281413	[redacted]	-0.323639	[redacted]
chr6	135865526	135865573	[redacted]	-0.323635	[redacted]
chr18	14968895	14968906	[redacted]	-0.323592	[redacted]
chr12	74891745	74891807	[redacted]	-0.323563	[redacted]
chr14	79525952	79525981	[redacted]	-0.323562	[redacted]
chr1	94791638	94791757	[redacted]	-0.323549	[redacted]
chr1	7803247	7803303	[redacted]	-0.323545	[redacted]
chr7	81063059	81063178	[redacted]	-0.323533	[redacted]
chr14	47664003	47664062	[redacted]	-0.323512	[redacted]
chr10	74980087	74980141	[redacted]	-0.323497	[redacted]
chr15	98322935	98322987	[redacted]	-0.323496	[redacted]
chr13	77553980	77554062	[redacted]	-0.323495	[redacted]
chr15	56349297	56349340	[redacted]	-0.323488	[redacted]
chr5	38431583	38431659	[redacted]	-0.323479	[redacted]

chr9	75521172	75521233	[redacted]	-0.323474	[redacted]
chr9	91124014	91124089	[redacted]	-0.323465	[redacted]
chr14	8139216	8139261	[redacted]	-0.323461	[redacted]
chr9	72761664	72761715	[redacted]	-0.323456	[redacted]
chr2	158853556	158853611	[redacted]	-0.323453	[redacted]
chr8	126660329	126660358	[redacted]	-0.323436	[redacted]
chr5	107046061	107046139	[redacted]	-0.32343	[redacted]
chr13	119679080	119679157	[redacted]	-0.323425	[redacted]
chr11	48281784	48281819	[redacted]	-0.323415	[redacted]
chr3	144357056	144357176	[redacted]	-0.323391	[redacted]
chr13	110493015	110493098	[redacted]	-0.323391	[redacted]
chr10	115142946	115142989	[redacted]	-0.323386	[redacted]
chr6	96327389	96327433	[redacted]	-0.32338	[redacted]
chr2	72484046	72484095	[redacted]	-0.323354	[redacted]
chr13	105533570	105533593	[redacted]	-0.323323	[redacted]
chr11	97303316	97303335	[redacted]	-0.323314	[redacted]
chr9	64178232	64178324	[redacted]	-0.323297	[redacted]
chr3	50004794	50004883	[redacted]	-0.323288	[redacted]
chr2	28386188	28386233	[redacted]	-0.323286	[redacted]
chr2	89111735	89111817	[redacted]	-0.323275	[redacted]
chr10	5205737	5205771	[redacted]	-0.323273	[redacted]
chr15	8213117	8213192	[redacted]	-0.323272	[redacted]
chr6	55246522	55246584	[redacted]	-0.323247	[redacted]
chr5	74636494	74636551	[redacted]	-0.32324	[redacted]
chr15	36730908	36730969	[redacted]	-0.323233	[redacted]
chr1	73709235	73709294	[redacted]	-0.323219	[redacted]
chr8	40233284	40233334	[redacted]	-0.323218	[redacted]
chr11	5459074	5459189	[redacted]	-0.323217	[redacted]
chr2	91617635	91617680	[redacted]	-0.323214	[redacted]
chr7	113908345	113908388	[redacted]	-0.32321	[redacted]
chr19	41698442	41698497	[redacted]	-0.323209	[redacted]
chr18	76773193	76773258	[redacted]	-0.323199	[redacted]
chr8	24516389	24516540	[redacted]	-0.32319	[redacted]
chr16	30817546	30817594	[redacted]	-0.323188	[redacted]
chr4	6115901	6115945	[redacted]	-0.323184	[redacted]
chr10	85740357	85740430	[redacted]	-0.323177	[redacted]
chr4	94165838	94165898	[redacted]	-0.323173	[redacted]
chr15	100182461	100182725	[redacted]	-0.323168	[redacted]
chr10	9945793	9945831	[redacted]	-0.323165	[redacted]
chr15	12767981	12768046	[redacted]	-0.323163	[redacted]
chr1	119208763	119208831	[redacted]	-0.323159	[redacted]
chr11	63644345	63644418	[redacted]	-0.323159	[redacted]
chr12	37110698	37110753	[redacted]	-0.323154	[redacted]

chr8	10021677	10021755	[redacted]	-0.323149	[redacted]
chr12	49614302	49614351	[redacted]	-0.323136	[redacted]
chr2	157698731	157698797	[redacted]	-0.323135	[redacted]
chr9	34392766	34392899	[redacted]	-0.323128	[redacted]
chr9	72927833	72927908	[redacted]	-0.323098	[redacted]
chr11	59442335	59442481	[redacted]	-0.323093	[redacted]
chr17	74554266	74554342	[redacted]	-0.323088	[redacted]
chr13	83895712	83895759	[redacted]	-0.323086	[redacted]
chr19	29084334	29084393	[redacted]	-0.323081	[redacted]
chr4	13202609	13202694	[redacted]	-0.323071	[redacted]
chr6	61632958	61633022	[redacted]	-0.323071	[redacted]
chr6	36539981	36540045	[redacted]	-0.32307	[redacted]
chr11	69549611	69549660	[redacted]	-0.323048	[redacted]
chr15	53272752	53272810	[redacted]	-0.323044	[redacted]
chr5	62162558	62162610	[redacted]	-0.323031	[redacted]
chr2	126542735	126542813	[redacted]	-0.32303	[redacted]
chr14	31005570	31005688	[redacted]	-0.322985	[redacted]
chr5	91663794	91663857	[redacted]	-0.322967	[redacted]
chr5	122044810	122044879	[redacted]	-0.322962	[redacted]
chr1	22866252	22866310	[redacted]	-0.322954	[redacted]
chr9	31293666	31293732	[redacted]	-0.322954	[redacted]
chr10	117368268	117368273	[redacted]	-0.32295	[redacted]
chr19	44309505	44309533	[redacted]	-0.322938	[redacted]
chr10	111530421	111530471	[redacted]	-0.322914	[redacted]
chr1	184749037	184749070	[redacted]	-0.322907	[redacted]
chr18	49222718	49222784	[redacted]	-0.3229	[redacted]
chr19	38089443	38089525	[redacted]	-0.322885	[redacted]
chr11	69119686	69119705	[redacted]	-0.322863	[redacted]
chr4	114816327	114816380	[redacted]	-0.322837	[redacted]
chr2	137259367	137259428	[redacted]	-0.32281	[redacted]
chr3	31358539	31358575	[redacted]	-0.322806	[redacted]
chr15	83281738	83281901	[redacted]	-0.322798	[redacted]
chr15	63829172	63829179	[redacted]	-0.322797	[redacted]
chrX	83343414	83343501	[redacted]	-0.322783	[redacted]
chr15	72496962	72497051	[redacted]	-0.322782	[redacted]
chr7	47225108	47225169	[redacted]	-0.322767	[redacted]
chr1	55836350	55836425	[redacted]	-0.322764	[redacted]
chr2	24310857	24310921	[redacted]	-0.322764	[redacted]
chr13	68432055	68432103	[redacted]	-0.322756	[redacted]
chr4	135196189	135196258	[redacted]	-0.322744	[redacted]
chr16	4023912	4023923	[redacted]	-0.32273	[redacted]
chr2	154600214	154600303	[redacted]	-0.322728	[redacted]
chr15	34275533	34275642	[redacted]	-0.322724	[redacted]

chr12	107210638	107210693	[redacted]	-0.322719	[redacted]
chr5	73152969	73152984	[redacted]	-0.322711	[redacted]
chr12	109521591	109521641	[redacted]	-0.322692	[redacted]
chr5	136558616	136558665	[redacted]	-0.32269	[redacted]
chr1	96617298	96617354	[redacted]	-0.322657	[redacted]
chr6	44027230	44027296	[redacted]	-0.322634	[redacted]
chr8	120652359	120652428	[redacted]	-0.322614	[redacted]
chr19	21022233	21022282	[redacted]	-0.322613	[redacted]
chr5	53232516	53232570	[redacted]	-0.322605	[redacted]
chr14	26566319	26566375	[redacted]	-0.3226	[redacted]
chr10	19190986	19191055	[redacted]	-0.32256	[redacted]
chr11	69714837	69714857	[redacted]	-0.322547	[redacted]
chr4	105100228	105100326	[redacted]	-0.322546	[redacted]
chr2	127788398	127788431	[redacted]	-0.322529	[redacted]
chr2	85224286	85224325	[redacted]	-0.322524	[redacted]
chr4	29348376	29348497	[redacted]	-0.322523	[redacted]
chr18	68010778	68010912	[redacted]	-0.322515	[redacted]
chr11	33659856	33659941	[redacted]	-0.322514	[redacted]
chr17	44631253	44631308	[redacted]	-0.322513	[redacted]
chr12	38839108	38839182	[redacted]	-0.322496	[redacted]
chr14	94953283	94953337	[redacted]	-0.322472	[redacted]
chr3	47194484	47194534	[redacted]	-0.322461	[redacted]
chr7	102658139	102658204	[redacted]	-0.322456	[redacted]
chr6	48952570	48952666	[redacted]	-0.322454	[redacted]
chr4	6941568	6941653	[redacted]	-0.322444	[redacted]
chr3	126931414	126931509	[redacted]	-0.322435	[redacted]
chr6	34676451	34676514	[redacted]	-0.322431	[redacted]
chr3	148961952	148962033	[redacted]	-0.322423	[redacted]
chr1	195244571	195244625	[redacted]	-0.322414	[redacted]
chrX	26801862	26801916	[redacted]	-0.322393	[redacted]
chr3	30227467	30227532	[redacted]	-0.322389	[redacted]
chr3	123883827	123883886	[redacted]	-0.322388	[redacted]
chr18	46595152	46595187	[redacted]	-0.322348	[redacted]
chr8	47276712	47276857	[redacted]	-0.322328	[redacted]
chr1	181848703	181848767	[redacted]	-0.32231	[redacted]
chr11	22185652	22185710	[redacted]	-0.322306	[redacted]
chr1	125408826	125408867	[redacted]	-0.322286	[redacted]
chr1	39438480	39438501	[redacted]	-0.322284	[redacted]
chr7	104050064	104050140	[redacted]	-0.32228	[redacted]
chr9	55353825	55353875	[redacted]	-0.322266	[redacted]
chr13	15932267	15932308	[redacted]	-0.32226	[redacted]
chr12	12489904	12489965	[redacted]	-0.322255	[redacted]
chr17	14955067	14955102	[redacted]	-0.322255	[redacted]

chr15	86269735	86269828	[redacted]	-0.322228	[redacted]
chr13	56723793	56723865	[redacted]	-0.322228	[redacted]
chr11	76343094	76343152	[redacted]	-0.322228	[redacted]
chr9	73174827	73174862	[redacted]	-0.322227	[redacted]
chr10	60135373	60135440	[redacted]	-0.322225	[redacted]
chrX	7805903	7805962	[redacted]	-0.322204	[redacted]
chr9	87377007	87377115	[redacted]	-0.322184	[redacted]
chr4	97559307	97559342	[redacted]	-0.322183	[redacted]
chr3	125851921	125851989	[redacted]	-0.322178	[redacted]
chr6	36987225	36987377	[redacted]	-0.322138	[redacted]
chr1	125205893	125205981	[redacted]	-0.322118	[redacted]
chr14	10577224	10577279	[redacted]	-0.322103	[redacted]
chr9	99196027	99196072	[redacted]	-0.322102	[redacted]
chr2	75596376	75596443	[redacted]	-0.322101	[redacted]
chr9	28782120	28782158	[redacted]	-0.322089	[redacted]
chr10	100068325	100068399	[redacted]	-0.322076	[redacted]
chr5	20704255	20704276	[redacted]	-0.322074	[redacted]
chr14	56486975	56487023	[redacted]	-0.322067	[redacted]
chr10	108146430	108146499	[redacted]	-0.322052	[redacted]
chr10	72966113	72966172	[redacted]	-0.322042	[redacted]
chr14	122719652	122719696	[redacted]	-0.322035	[redacted]
chr7	82222454	82222510	[redacted]	-0.322024	[redacted]
chr18	68011140	68011228	[redacted]	-0.322002	[redacted]
chr3	88066727	88066793	[redacted]	-0.321998	[redacted]
chr14	59089292	59089324	[redacted]	-0.321991	[redacted]
chr1	182945640	182945795	[redacted]	-0.321968	[redacted]
chr10	116925517	116925634	[redacted]	-0.321967	[redacted]
chr19	38485186	38485266	[redacted]	-0.321948	[redacted]
chr5	38427131	38427174	[redacted]	-0.321924	[redacted]
chr3	108240800	108240894	[redacted]	-0.321922	[redacted]
chr6	63344634	63344703	[redacted]	-0.321913	[redacted]
chr7	16088278	16088331	[redacted]	-0.321908	[redacted]
chr13	116849672	116849720	[redacted]	-0.321905	[redacted]
chr10	120250596	120250715	[redacted]	-0.321894	[redacted]
chr2	173443654	173443703	[redacted]	-0.321888	[redacted]
chr6	144384658	144384710	[redacted]	-0.321845	[redacted]
chr1	132524762	132524897	[redacted]	-0.321832	[redacted]
chr10	16899858	16899930	[redacted]	-0.321831	[redacted]
chr18	77360404	77360462	[redacted]	-0.321828	[redacted]
chr3	144781053	144781100	[redacted]	-0.321827	[redacted]
chr1	191143198	191143223	[redacted]	-0.321801	[redacted]
chr14	16838733	16838830	[redacted]	-0.321798	[redacted]
chr17	49342999	49343073	[redacted]	-0.321798	[redacted]

chr8	122818276	122818336	[redacted]	-0.321787	[redacted]
chr7	75429249	75429362	[redacted]	-0.321764	[redacted]
chr1	56309318	56309381	[redacted]	-0.32172	[redacted]
chr16	95985922	95985978	[redacted]	-0.321715	[redacted]
chr4	54591530	54591591	[redacted]	-0.321702	[redacted]
chr1	88782007	88782068	[redacted]	-0.321675	[redacted]
chr19	29081957	29082022	[redacted]	-0.321665	[redacted]
chr5	35109822	35109844	[redacted]	-0.321662	[redacted]
chr15	6177240	6177287	[redacted]	-0.321656	[redacted]
chr17	73152479	73152511	[redacted]	-0.321653	[redacted]
chr17	59357831	59357892	[redacted]	-0.321653	[redacted]
chr12	65320586	65320607	[redacted]	-0.32165	[redacted]
chr6	146872584	146872624	[redacted]	-0.321648	[redacted]
chr11	116255840	116255885	[redacted]	-0.321638	[redacted]
chr10	26696967	26697004	[redacted]	-0.321629	[redacted]
chr17	77892367	77892424	[redacted]	-0.321613	[redacted]
chrX	166311865	166311926	[redacted]	-0.321605	[redacted]
chr8	23074862	23074927	[redacted]	-0.321576	[redacted]
chrX	52583346	52583482	[redacted]	-0.321574	[redacted]
chr15	58799783	58799809	[redacted]	-0.321555	[redacted]
chr17	73289821	73289892	[redacted]	-0.321538	[redacted]
chr18	77529746	77529816	[redacted]	-0.321508	[redacted]
chr19	21709318	21709406	[redacted]	-0.321505	[redacted]
chr1	190585406	190585461	[redacted]	-0.321501	[redacted]
chr16	77293288	77293316	[redacted]	-0.321499	[redacted]
chr6	149009799	149009840	[redacted]	-0.321498	[redacted]
chr4	4813497	4813544	[redacted]	-0.321483	[redacted]
chr1	150485587	150485629	[redacted]	-0.321464	[redacted]
chr13	23705233	23705289	[redacted]	-0.321461	[redacted]
chr5	53242481	53242545	[redacted]	-0.321457	[redacted]
chr18	65020537	65020621	[redacted]	-0.321453	[redacted]
chr9	53436183	53436255	[redacted]	-0.321451	[redacted]
chr1	76693411	76693477	[redacted]	-0.321451	[redacted]
chr6	49479183	49479250	[redacted]	-0.321446	[redacted]
chr11	90662270	90662338	[redacted]	-0.321438	[redacted]
chr3	126957266	126957343	[redacted]	-0.321431	[redacted]
chr18	33809954	33810025	[redacted]	-0.321419	[redacted]
chr9	103769738	103769797	[redacted]	-0.321399	[redacted]
chr19	53021550	53021609	[redacted]	-0.321398	[redacted]
chr7	83645703	83645777	[redacted]	-0.321389	[redacted]
chr11	12008206	12008228	[redacted]	-0.321385	[redacted]
chr4	83216364	83216425	[redacted]	-0.321383	[redacted]
chr5	122302812	122302846	[redacted]	-0.321379	[redacted]

chr4	135068291	135068356	[redacted]	-0.321368	[redacted]
chr11	41075935	41075971	[redacted]	-0.321355	[redacted]
chr2	147023398	147023429	[redacted]	-0.321341	[redacted]
chr10	97411226	97411295	[redacted]	-0.321331	[redacted]
chr6	127305182	127305218	[redacted]	-0.32131	[redacted]
chr15	57240012	57240050	[redacted]	-0.321301	[redacted]
chr9	97809447	97809484	[redacted]	-0.321298	[redacted]
chr4	141586695	141586725	[redacted]	-0.321297	[redacted]
chr3	135698056	135698119	[redacted]	-0.32127	[redacted]
chr2	154156304	154156399	[redacted]	-0.321262	[redacted]
chr16	33147144	33147169	[redacted]	-0.32125	[redacted]
chr14	117977924	117977993	[redacted]	-0.321247	[redacted]
chr11	110324981	110325028	[redacted]	-0.32124	[redacted]
chr2	7887581	7887639	[redacted]	-0.321228	[redacted]
chr13	30639802	30639841	[redacted]	-0.321223	[redacted]
chr13	45956538	45956562	[redacted]	-0.321218	[redacted]
chr17	74635525	74635572	[redacted]	-0.321208	[redacted]
chr3	9826581	9826608	[redacted]	-0.321195	[redacted]
chrX	138813462	138813540	[redacted]	-0.321176	[redacted]
chr17	15728428	15728486	[redacted]	-0.321171	[redacted]
chr12	15739015	15739093	[redacted]	-0.321151	[redacted]
chr19	53758414	53758452	[redacted]	-0.321149	[redacted]
chr7	81172410	81172446	[redacted]	-0.321147	[redacted]
chr8	83421773	83421852	[redacted]	-0.321129	[redacted]
chr9	14135816	14135851	[redacted]	-0.321128	[redacted]
chr11	22285921	22285967	[redacted]	-0.321115	[redacted]
chr14	10686027	10686088	[redacted]	-0.321106	[redacted]
chr2	93157881	93157936	[redacted]	-0.321089	[redacted]
chr1	162214002	162214096	[redacted]	-0.321057	[redacted]
chr3	141275714	141275778	[redacted]	-0.321048	[redacted]
chr2	17926646	17926712	[redacted]	-0.321032	[redacted]
chr6	17527980	17528007	[redacted]	-0.321024	[redacted]
chr13	45709432	45709480	[redacted]	-0.32101	[redacted]
chrX	85646754	85646777	[redacted]	-0.320999	[redacted]
chr2	115839269	115839314	[redacted]	-0.320958	[redacted]
chr2	170037502	170037607	[redacted]	-0.320945	[redacted]
chr13	56622997	56623061	[redacted]	-0.320943	[redacted]
chr11	11925263	11925329	[redacted]	-0.320931	[redacted]
chr14	32605003	32605036	[redacted]	-0.320913	[redacted]
chr11	6168867	6168912	[redacted]	-0.320911	[redacted]
chr6	57819214	57819308	[redacted]	-0.320907	[redacted]
chr5	117126577	117126633	[redacted]	-0.320904	[redacted]
chr3	142579949	142580023	[redacted]	-0.32089	[redacted]

chr10	119607088	119607132	[redacted]	-0.32089	[redacted]
chr5	86539743	86539786	[redacted]	-0.320886	[redacted]
chr14	119454991	119455043	[redacted]	-0.320884	[redacted]
chr5	140112004	140112015	[redacted]	-0.320839	[redacted]
chr14	87312580	87312643	[redacted]	-0.320839	[redacted]
chr9	95117791	95117800	[redacted]	-0.320803	[redacted]
chr14	62834328	62834413	[redacted]	-0.320786	[redacted]
chr2	52905063	52905124	[redacted]	-0.320779	[redacted]
chr19	47453374	47453424	[redacted]	-0.320773	[redacted]
chr14	41678323	41678353	[redacted]	-0.320756	[redacted]
chr15	72730741	72730838	[redacted]	-0.320747	[redacted]
chr5	151515081	151515136	[redacted]	-0.320733	[redacted]
chr3	79007346	79007402	[redacted]	-0.320717	[redacted]
chr3	69022274	69022357	[redacted]	-0.320696	[redacted]
chr1	8007631	8007660	[redacted]	-0.320695	[redacted]
chr10	20609540	20609566	[redacted]	-0.320686	[redacted]
chr9	108985929	108985985	[redacted]	-0.320673	[redacted]
chr12	56035583	56035708	[redacted]	-0.320671	[redacted]
chr4	126320798	126320823	[redacted]	-0.320661	[redacted]
chr1	87253624	87253672	[redacted]	-0.320659	[redacted]
chr6	120449038	120449105	[redacted]	-0.320641	[redacted]
chr7	134673148	134673228	[redacted]	-0.320638	[redacted]
chr12	77710634	77710722	[redacted]	-0.320634	[redacted]
chr6	93091586	93091639	[redacted]	-0.320631	[redacted]
chr11	74756399	74756461	[redacted]	-0.320627	[redacted]
chr4	109681526	109681598	[redacted]	-0.320624	[redacted]
chr4	119567063	119567155	[redacted]	-0.320619	[redacted]
chr13	102794965	102795022	[redacted]	-0.320614	[redacted]
chr18	30256512	30256582	[redacted]	-0.320608	[redacted]
chr9	41426684	41426752	[redacted]	-0.320599	[redacted]
chr18	14761403	14761457	[redacted]	-0.320591	[redacted]
chr6	142347012	142347052	[redacted]	-0.32059	[redacted]
chr5	124626026	124626059	[redacted]	-0.320583	[redacted]
chr2	116098317	116098400	[redacted]	-0.320583	[redacted]
chr11	65947975	65948022	[redacted]	-0.320573	[redacted]
chr7	113154365	113154415	[redacted]	-0.320571	[redacted]
chr7	42737726	42737772	[redacted]	-0.320552	[redacted]
chr13	63686807	63686825	[redacted]	-0.32055	[redacted]
chr8	59385934	59385974	[redacted]	-0.320549	[redacted]
chr8	120993201	120993268	[redacted]	-0.320539	[redacted]
chr2	72156594	72156612	[redacted]	-0.320532	[redacted]
chr8	71992601	71992646	[redacted]	-0.320488	[redacted]
chr13	40152041	40152072	[redacted]	-0.320485	[redacted]

chr3	45132427	45132481	[redacted]	-0.320484	[redacted]
chr3	143411770	143411849	[redacted]	-0.320474	[redacted]
chr3	134025764	134025827	[redacted]	-0.320455	[redacted]
chr3	99661088	99661138	[redacted]	-0.320442	[redacted]
chr17	46490684	46490720	[redacted]	-0.320433	[redacted]
chr17	4176403	4176448	[redacted]	-0.320427	[redacted]
chr7	112131617	112131701	[redacted]	-0.320417	[redacted]
chr1	70796983	70797055	[redacted]	-0.320412	[redacted]
chr11	10266379	10266444	[redacted]	-0.320407	[redacted]
chr12	111980166	111980286	[redacted]	-0.320394	[redacted]
chr3	81215279	81215376	[redacted]	-0.320385	[redacted]
chr15	98689524	98689581	[redacted]	-0.320378	[redacted]
chr12	71826788	71826839	[redacted]	-0.320374	[redacted]
chr14	33325349	33325440	[redacted]	-0.320349	[redacted]
chrX	134696242	134696318	[redacted]	-0.320347	[redacted]
chr5	122371298	122371335	[redacted]	-0.320317	[redacted]
chr17	35958630	35958700	[redacted]	-0.320316	[redacted]
chr11	54828618	54828674	[redacted]	-0.320289	[redacted]
chr13	56021171	56021213	[redacted]	-0.320278	[redacted]
chr19	26912194	26912237	[redacted]	-0.320278	[redacted]
chr1	69532470	69532509	[redacted]	-0.320271	[redacted]
chr9	112243121	112243155	[redacted]	-0.32027	[redacted]
chr5	73878519	73878588	[redacted]	-0.320248	[redacted]
chr17	29855739	29855782	[redacted]	-0.32024	[redacted]
chr19	52746393	52746452	[redacted]	-0.320236	[redacted]
chr4	98536564	98536620	[redacted]	-0.320234	[redacted]
chr9	73360744	73360762	[redacted]	-0.320227	[redacted]
chr13	81346737	81346872	[redacted]	-0.320217	[redacted]
chr15	28690752	28690828	[redacted]	-0.320183	[redacted]
chr11	53698246	53698293	[redacted]	-0.320177	[redacted]
chr7	18875443	18875495	[redacted]	-0.320172	[redacted]
chr1	190508248	190508300	[redacted]	-0.320162	[redacted]
chr2	9034465	9034536	[redacted]	-0.320155	[redacted]
chr17	31712745	31712818	[redacted]	-0.320141	[redacted]
chrX	85532627	85532654	[redacted]	-0.320139	[redacted]
chr1	135493153	135493220	[redacted]	-0.320137	[redacted]
chr12	107991757	107991786	[redacted]	-0.320119	[redacted]
chr5	46836802	46836877	[redacted]	-0.320107	[redacted]
chr10	126159337	126159389	[redacted]	-0.320107	[redacted]
chr7	140981700	140981720	[redacted]	-0.320086	[redacted]
chr9	22038693	22038731	[redacted]	-0.320082	[redacted]
chr12	7862642	7862703	[redacted]	-0.320081	[redacted]
chr6	81959117	81959210	[redacted]	-0.320064	[redacted]

chr10	72468587	72468674	[redacted]	-0.320061	[redacted]
chr17	87212400	87212487	[redacted]	-0.320043	[redacted]
chr6	144522563	144522588	[redacted]	-0.320032	[redacted]
chr16	92610929	92611005	[redacted]	-0.320025	[redacted]
chr5	64652753	64652839	[redacted]	-0.320022	[redacted]
chr1	72413421	72413458	[redacted]	-0.320022	[redacted]
chr16	18422764	18422797	[redacted]	-0.32002	[redacted]
chr18	57960162	57960248	[redacted]	-0.320013	[redacted]
chr1	51742366	51742414	[redacted]	-0.320011	[redacted]
chr19	11843355	11843447	[redacted]	-0.320004	[redacted]
chr11	36507463	36507568	[redacted]	-0.31999	[redacted]
chr15	75227169	75227244	[redacted]	-0.319982	[redacted]
chr3	53340775	53340906	[redacted]	-0.319971	[redacted]
chr3	49865937	49865956	[redacted]	-0.319969	[redacted]
chr6	39978140	39978234	[redacted]	-0.319963	[redacted]
chr8	57379250	57379313	[redacted]	-0.31996	[redacted]
chr13	43865345	43865368	[redacted]	-0.319958	[redacted]
chrX	78869455	78869517	[redacted]	-0.319954	[redacted]
chr2	125184795	125184836	[redacted]	-0.319943	[redacted]
chr13	109794559	109794590	[redacted]	-0.319932	[redacted]
chr8	129152602	129152689	[redacted]	-0.319926	[redacted]
chr4	3812022	3812109	[redacted]	-0.319925	[redacted]
chr18	74816138	74816204	[redacted]	-0.319907	[redacted]
chr2	110303098	110303163	[redacted]	-0.319905	[redacted]
chr2	140256967	140257053	[redacted]	-0.319898	[redacted]
chr12	118708267	118708333	[redacted]	-0.319896	[redacted]
chr13	29918864	29918925	[redacted]	-0.319891	[redacted]
chr13	43645906	43645955	[redacted]	-0.319882	[redacted]
chr2	33326032	33326062	[redacted]	-0.31988	[redacted]
chr16	32800634	32800654	[redacted]	-0.319866	[redacted]
chr16	9709207	9709249	[redacted]	-0.319863	[redacted]
chr8	35625738	35625762	[redacted]	-0.319861	[redacted]
chr14	21110693	21110773	[redacted]	-0.319855	[redacted]
chr6	92375817	92375890	[redacted]	-0.319855	[redacted]
chr11	32143994	32144033	[redacted]	-0.31984	[redacted]
chr6	70944423	70944473	[redacted]	-0.319837	[redacted]
chr19	32493738	32493792	[redacted]	-0.319799	[redacted]
chrX	121914832	121914849	[redacted]	-0.319799	[redacted]
chr10	118163382	118163436	[redacted]	-0.319796	[redacted]
chr5	45786984	45787056	[redacted]	-0.319782	[redacted]
chr19	48277751	48277809	[redacted]	-0.319755	[redacted]
chr19	27641240	27641263	[redacted]	-0.319752	[redacted]
chr15	4085872	4085936	[redacted]	-0.319716	[redacted]

chr14	106676498	106676553	[redacted]	-0.319713	[redacted]
chr18	70633200	70633280	[redacted]	-0.31971	[redacted]
chr4	11477190	11477218	[redacted]	-0.319699	[redacted]
chr10	67492736	67492798	[redacted]	-0.319682	[redacted]
chr2	156659917	156660046	[redacted]	-0.319671	[redacted]
chr14	31000641	31000747	[redacted]	-0.319658	[redacted]
chr3	19823920	19823967	[redacted]	-0.319657	[redacted]
chr7	133478485	133478566	[redacted]	-0.319653	[redacted]
chr8	106510022	106510081	[redacted]	-0.319628	[redacted]
chr10	87341910	87341997	[redacted]	-0.319623	[redacted]
chr4	26000708	26000795	[redacted]	-0.319619	[redacted]
chr12	69855215	69855309	[redacted]	-0.319612	[redacted]
chr6	68187722	68187804	[redacted]	-0.319611	[redacted]
chr6	144951264	144951330	[redacted]	-0.319578	[redacted]
chr2	163836116	163836167	[redacted]	-0.319551	[redacted]
chr12	86505623	86505705	[redacted]	-0.319535	[redacted]
chr12	80564086	80564106	[redacted]	-0.319535	[redacted]
chr2	69025372	69025501	[redacted]	-0.319515	[redacted]
chrX	157457374	157457498	[redacted]	-0.319496	[redacted]
chr1	192739168	192739307	[redacted]	-0.31949	[redacted]
chr11	91027406	91027515	[redacted]	-0.319485	[redacted]
chr19	29845707	29845784	[redacted]	-0.319459	[redacted]
chr7	15961036	15961050	[redacted]	-0.319453	[redacted]
chr13	73424939	73425021	[redacted]	-0.31944	[redacted]
chr13	58871205	58871272	[redacted]	-0.319411	[redacted]
chr7	90979946	90980006	[redacted]	-0.319393	[redacted]
chr2	165550585	165550628	[redacted]	-0.31937	[redacted]
chr2	77583128	77583181	[redacted]	-0.319368	[redacted]
chr6	29883092	29883148	[redacted]	-0.319358	[redacted]
chrX	53633469	53633515	[redacted]	-0.319345	[redacted]
chr17	82215210	82215263	[redacted]	-0.319342	[redacted]
chr2	93932632	93932688	[redacted]	-0.319325	[redacted]
chr15	7108134	7108233	[redacted]	-0.31931	[redacted]
chr9	94982520	94982569	[redacted]	-0.319279	[redacted]
chr1	85284014	85284058	[redacted]	-0.319253	[redacted]
chr6	100232849	100232905	[redacted]	-0.319245	[redacted]
chr8	104274496	104274536	[redacted]	-0.319229	[redacted]
chr1	162706512	162706541	[redacted]	-0.319227	[redacted]
chr8	13876837	13876892	[redacted]	-0.319226	[redacted]
chr18	81221953	81221984	[redacted]	-0.319198	[redacted]
chr9	31603285	31603322	[redacted]	-0.31919	[redacted]
chr13	56465909	56466040	[redacted]	-0.319179	[redacted]
chr19	44254397	44254487	[redacted]	-0.319162	[redacted]

chr11	97693917	97693963	[redacted]	-0.319153	[redacted]
chr5	28348005	28348039	[redacted]	-0.319147	[redacted]
chr7	135213841	135213863	[redacted]	-0.319146	[redacted]
chr7	121090206	121090248	[redacted]	-0.319138	[redacted]
chr13	117097185	117097234	[redacted]	-0.319136	[redacted]
chr5	105317511	105317576	[redacted]	-0.31912	[redacted]
chr6	40721844	40721898	[redacted]	-0.319117	[redacted]
chr11	90204179	90204255	[redacted]	-0.319115	[redacted]
chr7	141825408	141825438	[redacted]	-0.319112	[redacted]
chr1	167426910	167426949	[redacted]	-0.319108	[redacted]
chr10	120562547	120562604	[redacted]	-0.319106	[redacted]
chr17	58892893	58892943	[redacted]	-0.319081	[redacted]
chr9	48163507	48163563	[redacted]	-0.319073	[redacted]
chr9	99050778	99050805	[redacted]	-0.319069	[redacted]
chr4	145378263	145378313	[redacted]	-0.319068	[redacted]
chr2	28273935	28274015	[redacted]	-0.319065	[redacted]
chr8	38094668	38094730	[redacted]	-0.319052	[redacted]
chr11	120201506	120201559	[redacted]	-0.319051	[redacted]
chr8	126863022	126863087	[redacted]	-0.319034	[redacted]
chr2	72292513	72292584	[redacted]	-0.319013	[redacted]
chr19	31857031	31857075	[redacted]	-0.319008	[redacted]
chr5	21434369	21434440	[redacted]	-0.319	[redacted]
chr8	32777346	32777403	[redacted]	-0.318994	[redacted]
chr6	90533248	90533297	[redacted]	-0.318992	[redacted]
chr1	53811686	53811747	[redacted]	-0.318981	[redacted]
chr14	14913652	14913727	[redacted]	-0.31896	[redacted]
chr6	139562953	139563016	[redacted]	-0.318934	[redacted]
chr2	125714993	125715033	[redacted]	-0.318932	[redacted]
chrX	7817566	7817624	[redacted]	-0.318931	[redacted]
chr19	41262621	41262669	[redacted]	-0.31893	[redacted]
chr2	29095879	29095930	[redacted]	-0.318929	[redacted]
chr17	45264590	45264683	[redacted]	-0.318922	[redacted]
chr8	80138630	80138689	[redacted]	-0.318921	[redacted]
chr7	59271951	59272060	[redacted]	-0.318918	[redacted]
chrX	153941620	153941649	[redacted]	-0.318907	[redacted]
chr15	41696222	41696256	[redacted]	-0.318899	[redacted]
chr14	73451527	73451572	[redacted]	-0.318892	[redacted]
chr2	37853019	37853058	[redacted]	-0.318886	[redacted]
chr9	115462437	115462511	[redacted]	-0.318858	[redacted]
chr11	110302941	110303138	[redacted]	-0.318858	[redacted]
chr18	63092766	63092822	[redacted]	-0.318853	[redacted]
chr2	74913828	74913926	[redacted]	-0.318833	[redacted]
chr12	105792223	105792240	[redacted]	-0.318814	[redacted]

chr9	3017134	3017166	[redacted]	-0.318806	[redacted]
chr15	94419063	94419081	[redacted]	-0.318791	[redacted]
chr6	7954516	7954571	[redacted]	-0.31877	[redacted]
chr9	33593664	33593702	[redacted]	-0.318723	[redacted]
chr11	21339803	21339857	[redacted]	-0.318722	[redacted]
chr10	31085011	31085059	[redacted]	-0.318717	[redacted]
chr4	148169026	148169074	[redacted]	-0.318715	[redacted]
chr14	123990436	123990519	[redacted]	-0.318708	[redacted]
chr13	8608119	8608169	[redacted]	-0.318698	[redacted]
chr13	42628947	42628988	[redacted]	-0.318695	[redacted]
chr1	36047615	36047701	[redacted]	-0.318689	[redacted]
chr16	93038000	93038026	[redacted]	-0.318682	[redacted]
chr14	55116494	55116556	[redacted]	-0.318681	[redacted]
chr17	46346825	46346886	[redacted]	-0.318672	[redacted]
chr2	136792707	136792754	[redacted]	-0.318648	[redacted]
chr12	53817028	53817158	[redacted]	-0.318646	[redacted]
chr12	108955380	108955433	[redacted]	-0.318645	[redacted]
chr10	92991541	92991566	[redacted]	-0.318634	[redacted]
chr1	152989222	152989318	[redacted]	-0.318628	[redacted]
chr7	46125903	46125980	[redacted]	-0.318622	[redacted]
chr3	142978956	142979001	[redacted]	-0.318615	[redacted]
chr5	122128277	122128341	[redacted]	-0.318608	[redacted]
chr10	17464891	17464954	[redacted]	-0.318597	[redacted]
chr9	56920041	56920179	[redacted]	-0.318583	[redacted]
chr17	34948134	34948198	[redacted]	-0.318579	[redacted]
chr11	49916286	49916331	[redacted]	-0.318574	[redacted]
chr6	76821168	76821208	[redacted]	-0.318574	[redacted]
chr14	56217034	56217130	[redacted]	-0.318573	[redacted]
chr2	104403066	104403110	[redacted]	-0.318561	[redacted]
chr15	68272156	68272208	[redacted]	-0.318558	[redacted]
chr9	53287210	53287219	[redacted]	-0.318552	[redacted]
chr1	118194771	118194813	[redacted]	-0.318515	[redacted]
chr3	79666411	79666478	[redacted]	-0.318515	[redacted]
chr1	155041513	155041597	[redacted]	-0.318508	[redacted]
chr11	43987466	43987523	[redacted]	-0.318505	[redacted]
chr13	101892329	101892400	[redacted]	-0.318496	[redacted]
chr2	174784148	174784197	[redacted]	-0.318496	[redacted]
chr10	117882995	117883078	[redacted]	-0.318495	[redacted]
chr12	107618379	107618441	[redacted]	-0.318487	[redacted]
chr18	38549791	38549889	[redacted]	-0.318471	[redacted]
chr8	121925631	121925654	[redacted]	-0.318464	[redacted]
chr4	37755915	37755967	[redacted]	-0.318459	[redacted]
chr1	180902701	180902750	[redacted]	-0.318454	[redacted]

chr19	18970532	18970590	[redacted]	-0.318451	[redacted]
chr8	58774346	58774411	[redacted]	-0.318429	[redacted]
chr11	82849950	82849997	[redacted]	-0.318422	[redacted]
chr4	34666909	34666940	[redacted]	-0.31842	[redacted]
chr15	85234337	85234476	[redacted]	-0.318397	[redacted]
chr4	34904500	34904583	[redacted]	-0.318391	[redacted]
chr19	6535884	6535907	[redacted]	-0.318386	[redacted]
chr11	87713558	87713633	[redacted]	-0.318385	[redacted]
chr19	29670124	29670178	[redacted]	-0.318379	[redacted]
chr18	52727649	52727693	[redacted]	-0.318372	[redacted]
chr8	11013724	11013802	[redacted]	-0.318368	[redacted]
chr10	42839689	42839766	[redacted]	-0.318355	[redacted]
chr14	65758125	65758258	[redacted]	-0.318323	[redacted]
chr1	119264943	119265033	[redacted]	-0.318299	[redacted]
chr4	101035892	101035951	[redacted]	-0.31829	[redacted]
chr13	49397970	49398094	[redacted]	-0.318282	[redacted]
chr13	53393138	53393219	[redacted]	-0.318261	[redacted]
chrX	143710539	143710590	[redacted]	-0.318261	[redacted]
chr18	4532486	4532551	[redacted]	-0.318257	[redacted]
chr8	40795551	40795584	[redacted]	-0.318255	[redacted]
chr10	119320022	119320062	[redacted]	-0.318221	[redacted]
chr9	96716277	96716286	[redacted]	-0.318218	[redacted]
chr11	94222886	94222939	[redacted]	-0.31821	[redacted]
chr15	90043235	90043278	[redacted]	-0.318176	[redacted]
chr15	50687017	50687067	[redacted]	-0.31816	[redacted]
chr4	25258623	25258731	[redacted]	-0.318151	[redacted]
chr14	62255036	62255081	[redacted]	-0.318138	[redacted]
chr9	6747347	6747438	[redacted]	-0.318126	[redacted]
chr11	66904035	66904097	[redacted]	-0.318117	[redacted]
chr14	34505214	34505247	[redacted]	-0.318112	[redacted]
chr9	87242095	87242170	[redacted]	-0.318106	[redacted]
chr14	36085262	36085346	[redacted]	-0.318104	[redacted]
chr14	8665289	8665343	[redacted]	-0.318103	[redacted]
chr1	126172303	126172362	[redacted]	-0.318099	[redacted]
chr5	100714365	100714444	[redacted]	-0.318095	[redacted]
chr5	147338685	147338699	[redacted]	-0.318069	[redacted]
chr9	85826298	85826380	[redacted]	-0.318066	[redacted]
chr7	79469740	79469821	[redacted]	-0.318064	[redacted]
chr18	53922580	53922652	[redacted]	-0.318064	[redacted]
chr9	123652844	123652924	[redacted]	-0.318062	[redacted]
chr14	48542367	48542438	[redacted]	-0.318061	[redacted]
chr12	83246343	83246435	[redacted]	-0.318008	[redacted]
chr13	117696150	117696201	[redacted]	-0.318003	[redacted]

chr3	67970721	67970826	[redacted]	-0.317993	[redacted]
chr17	23577837	23577898	[redacted]	-0.317987	[redacted]
chr10	105439509	105439532	[redacted]	-0.31798	[redacted]
chr10	102624930	102624978	[redacted]	-0.317975	[redacted]
chr5	107224744	107224794	[redacted]	-0.317973	[redacted]
chr13	14727831	14727868	[redacted]	-0.317971	[redacted]
chr7	72034336	72034382	[redacted]	-0.317967	[redacted]
chr5	88583816	88583874	[redacted]	-0.317923	[redacted]
chr2	99165569	99165697	[redacted]	-0.31792	[redacted]
chr14	55108124	55108199	[redacted]	-0.317916	[redacted]
chr5	88782538	88782564	[redacted]	-0.317909	[redacted]
chr8	84929646	84929661	[redacted]	-0.317908	[redacted]
chr9	82091395	82091450	[redacted]	-0.317907	[redacted]
chr7	80847007	80847030	[redacted]	-0.317894	[redacted]
chr8	12057605	12057773	[redacted]	-0.317883	[redacted]
chr5	72450206	72450259	[redacted]	-0.317879	[redacted]
chr17	76689959	76690002	[redacted]	-0.317878	[redacted]
chr2	129690768	129690813	[redacted]	-0.317866	[redacted]
chr6	148499052	148499181	[redacted]	-0.317864	[redacted]
chr5	57947178	57947214	[redacted]	-0.317863	[redacted]
chr10	54807215	54807308	[redacted]	-0.317858	[redacted]
chr18	12790793	12790846	[redacted]	-0.31785	[redacted]
chr14	66520412	66520472	[redacted]	-0.317831	[redacted]
chr7	75808541	75808639	[redacted]	-0.317823	[redacted]
chr2	101874256	101874311	[redacted]	-0.317818	[redacted]
chr2	102148203	102148276	[redacted]	-0.317783	[redacted]
chr10	116316061	116316070	[redacted]	-0.317774	[redacted]
chr17	70061551	70061598	[redacted]	-0.317773	[redacted]
chr8	79295743	79295807	[redacted]	-0.317771	[redacted]
chr3	34951578	34951635	[redacted]	-0.317771	[redacted]
chr8	18617387	18617416	[redacted]	-0.31777	[redacted]
chr13	18054464	18054547	[redacted]	-0.317766	[redacted]
chr13	93773435	93773486	[redacted]	-0.317742	[redacted]
chr3	124595985	124596031	[redacted]	-0.317742	[redacted]
chr19	46708862	46708950	[redacted]	-0.317703	[redacted]
chr17	47228859	47228896	[redacted]	-0.317691	[redacted]
chr5	104005257	104005330	[redacted]	-0.317682	[redacted]
chr2	72211744	72211805	[redacted]	-0.31768	[redacted]
chr11	15896047	15896148	[redacted]	-0.317671	[redacted]
chr19	51650312	51650357	[redacted]	-0.317667	[redacted]
chr9	78636493	78636534	[redacted]	-0.31766	[redacted]
chr11	33833749	33833822	[redacted]	-0.31765	[redacted]
chr18	64303844	64303889	[redacted]	-0.317644	[redacted]

chr7	100307212	100307277	[redacted]	-0.317644	[redacted]
chrX	103369525	103369571	[redacted]	-0.31763	[redacted]
chr7	73423226	73423333	[redacted]	-0.317627	[redacted]
chr2	140070138	140070212	[redacted]	-0.317624	[redacted]
chr10	80982315	80982367	[redacted]	-0.317622	[redacted]
chr11	120840732	120840776	[redacted]	-0.317615	[redacted]
chr2	21474589	21474674	[redacted]	-0.317608	[redacted]
chr6	49320990	49321019	[redacted]	-0.317606	[redacted]
chr1	82221379	82221434	[redacted]	-0.317603	[redacted]
chr3	157699791	157699852	[redacted]	-0.317602	[redacted]
chr3	115666798	115666864	[redacted]	-0.317594	[redacted]
chr1	54332973	54333054	[redacted]	-0.317551	[redacted]
chr1	187442832	187442908	[redacted]	-0.317541	[redacted]
chr9	20991064	20991120	[redacted]	-0.317533	[redacted]
chr13	107953349	107953402	[redacted]	-0.317515	[redacted]
chr14	24762107	24762133	[redacted]	-0.317514	[redacted]
chr1	61630366	61630420	[redacted]	-0.3175	[redacted]
chr3	121787903	121787965	[redacted]	-0.317499	[redacted]
chr8	61489796	61489889	[redacted]	-0.317485	[redacted]
chr16	11914001	11914084	[redacted]	-0.317484	[redacted]
chr9	51944677	51944701	[redacted]	-0.317483	[redacted]
chr16	55989703	55989759	[redacted]	-0.31747	[redacted]
chr6	66562746	66562804	[redacted]	-0.317437	[redacted]
chr6	25428729	25428805	[redacted]	-0.31743	[redacted]
chr18	47526518	47526565	[redacted]	-0.317427	[redacted]
chr3	34918737	34918764	[redacted]	-0.317424	[redacted]
chr10	104850470	104850486	[redacted]	-0.31741	[redacted]
chr14	120506474	120506511	[redacted]	-0.317408	[redacted]
chr8	9598658	9598705	[redacted]	-0.317395	[redacted]
chr10	93714455	93714507	[redacted]	-0.317387	[redacted]
chr11	113089805	113089844	[redacted]	-0.317386	[redacted]
chr13	29937521	29937585	[redacted]	-0.317376	[redacted]
chr17	62282022	62282093	[redacted]	-0.317376	[redacted]
chr5	64443492	64443544	[redacted]	-0.317375	[redacted]
chr7	137854896	137854941	[redacted]	-0.317353	[redacted]
chr8	91263216	91263295	[redacted]	-0.317347	[redacted]
chr12	15591556	15591600	[redacted]	-0.317345	[redacted]
chr6	21725054	21725131	[redacted]	-0.317343	[redacted]
chr12	34082397	34082435	[redacted]	-0.317331	[redacted]
chr8	12258345	12258396	[redacted]	-0.31733	[redacted]
chr11	99073044	99073076	[redacted]	-0.317315	[redacted]
chr1	61663415	61663486	[redacted]	-0.317295	[redacted]
chr1	13294191	13294251	[redacted]	-0.317295	[redacted]

chr14	41592021	41592087	[redacted]	-0.317294	[redacted]
chr17	45569025	45569085	[redacted]	-0.317281	[redacted]
chr3	127406618	127406651	[redacted]	-0.317281	[redacted]
chr8	82936178	82936361	[redacted]	-0.317277	[redacted]
chr5	123779749	123779800	[redacted]	-0.31727	[redacted]
chr18	5845710	5845797	[redacted]	-0.317266	[redacted]
chrX	56990485	56990510	[redacted]	-0.317251	[redacted]
chr6	73233048	73233137	[redacted]	-0.317246	[redacted]
chr2	64193087	64193125	[redacted]	-0.317242	[redacted]
chr1	177092818	177092862	[redacted]	-0.317236	[redacted]
chrX	72183037	72183071	[redacted]	-0.317213	[redacted]
chr6	72034669	72034730	[redacted]	-0.317209	[redacted]
chr2	158883418	158883454	[redacted]	-0.317207	[redacted]
chr11	53231755	53231799	[redacted]	-0.3172	[redacted]
chr13	97500384	97500490	[redacted]	-0.317184	[redacted]
chr7	112570829	112570886	[redacted]	-0.317178	[redacted]
chr12	75537483	75537515	[redacted]	-0.31717	[redacted]
chr3	68437828	68437852	[redacted]	-0.317164	[redacted]
chr10	116075925	116075972	[redacted]	-0.31714	[redacted]
chr2	60455117	60455220	[redacted]	-0.317122	[redacted]
chr12	106444583	106444675	[redacted]	-0.317119	[redacted]
chr12	98219128	98219247	[redacted]	-0.317112	[redacted]
chr11	76725335	76725403	[redacted]	-0.317087	[redacted]
chr2	34668067	34668168	[redacted]	-0.317086	[redacted]
chr6	96956803	96956889	[redacted]	-0.317079	[redacted]
chr9	62346634	62346673	[redacted]	-0.317079	[redacted]
chr2	55445497	55445539	[redacted]	-0.317077	[redacted]
chr1	55814804	55814858	[redacted]	-0.31707	[redacted]
chr4	152063614	152063655	[redacted]	-0.317053	[redacted]
chr12	31327639	31327720	[redacted]	-0.317047	[redacted]
chr14	65522800	65522847	[redacted]	-0.317041	[redacted]
chr6	55607020	55607084	[redacted]	-0.317027	[redacted]
chr11	80796547	80796607	[redacted]	-0.317017	[redacted]
chr18	80384216	80384258	[redacted]	-0.317009	[redacted]
chr8	13991559	13991576	[redacted]	-0.317009	[redacted]
chr7	54880745	54880804	[redacted]	-0.317008	[redacted]
chr18	79155323	79155383	[redacted]	-0.317003	[redacted]
chr2	69664451	69664499	[redacted]	-0.317003	[redacted]
chr7	98578721	98578797	[redacted]	-0.316993	[redacted]
chr7	71750245	71750300	[redacted]	-0.316985	[redacted]
chr2	65203934	65204049	[redacted]	-0.31697	[redacted]
chr2	141996568	141996616	[redacted]	-0.316949	[redacted]
chr1	153162530	153162597	[redacted]	-0.316941	[redacted]

chr12	44590249	44590289	[redacted]	-0.31693	[redacted]
chr4	94066653	94066712	[redacted]	-0.316921	[redacted]
chr12	33856136	33856217	[redacted]	-0.316889	[redacted]
chr11	96444039	96444132	[redacted]	-0.316876	[redacted]
chr13	62527040	62527107	[redacted]	-0.316874	[redacted]
chr15	30073317	30073363	[redacted]	-0.316871	[redacted]
chr17	65595566	65595656	[redacted]	-0.316868	[redacted]
chr2	64079394	64079452	[redacted]	-0.316827	[redacted]
chr18	81187245	81187282	[redacted]	-0.316817	[redacted]
chr2	83829718	83829795	[redacted]	-0.316803	[redacted]
chr1	80823900	80823972	[redacted]	-0.316801	[redacted]
chr9	50471854	50471920	[redacted]	-0.316781	[redacted]
chr1	138278776	138278838	[redacted]	-0.316779	[redacted]
chrX	112462125	112462211	[redacted]	-0.316754	[redacted]
chr4	57180587	57180653	[redacted]	-0.31675	[redacted]
chr14	48294766	48294814	[redacted]	-0.31674	[redacted]
chr14	121108684	121108744	[redacted]	-0.31674	[redacted]
chr3	41813943	41813995	[redacted]	-0.316735	[redacted]
chr11	67528511	67528550	[redacted]	-0.316722	[redacted]
chr6	30575823	30575866	[redacted]	-0.316714	[redacted]
chr8	98990297	98990378	[redacted]	-0.316709	[redacted]
chr16	42788920	42788970	[redacted]	-0.316702	[redacted]
chr2	144305535	144305598	[redacted]	-0.316696	[redacted]
chr4	19956294	19956358	[redacted]	-0.316693	[redacted]
chr2	157703079	157703128	[redacted]	-0.316692	[redacted]
chr9	95120906	95120965	[redacted]	-0.316689	[redacted]
chr8	112238966	112239035	[redacted]	-0.316689	[redacted]
chr8	38234570	38234642	[redacted]	-0.316658	[redacted]
chr15	43291540	43291599	[redacted]	-0.316657	[redacted]
chr7	68743784	68743869	[redacted]	-0.316645	[redacted]
chr15	25514148	25514233	[redacted]	-0.316645	[redacted]
chr11	31756891	31756995	[redacted]	-0.316639	[redacted]
chr10	93929341	93929436	[redacted]	-0.316614	[redacted]
chr15	83254377	83254474	[redacted]	-0.316609	[redacted]
chr9	122523666	122523736	[redacted]	-0.316584	[redacted]
chr7	8510748	8510810	[redacted]	-0.316573	[redacted]
chr8	105173324	105173381	[redacted]	-0.316562	[redacted]
chr9	4627748	4627783	[redacted]	-0.316549	[redacted]
chr9	101025294	101025355	[redacted]	-0.316536	[redacted]
chr7	66947282	66947335	[redacted]	-0.31653	[redacted]
chr9	117664408	117664468	[redacted]	-0.31653	[redacted]
chr8	100811256	100811314	[redacted]	-0.31652	[redacted]
chr2	131029467	131029504	[redacted]	-0.316491	[redacted]

chr11	105773421	105773507	[redacted]	-0.316488	[redacted]
chr10	18785429	18785500	[redacted]	-0.316484	[redacted]
chr13	23952713	23952749	[redacted]	-0.316478	[redacted]
chr5	116341113	116341159	[redacted]	-0.316466	[redacted]
chr13	40957410	40957472	[redacted]	-0.31646	[redacted]
chr5	90630621	90630713	[redacted]	-0.316456	[redacted]
chr14	101761390	101761436	[redacted]	-0.316445	[redacted]
chr17	86040950	86041006	[redacted]	-0.316431	[redacted]
chr18	81455226	81455285	[redacted]	-0.316419	[redacted]
chr8	35187538	35187556	[redacted]	-0.3164	[redacted]
chr11	21039277	21039346	[redacted]	-0.31638	[redacted]
chr5	121336174	121336229	[redacted]	-0.316354	[redacted]
chr2	39114305	39114323	[redacted]	-0.316353	[redacted]
chr19	30407920	30407972	[redacted]	-0.316349	[redacted]
chr9	120149698	120149738	[redacted]	-0.316345	[redacted]
chr7	139865960	139866041	[redacted]	-0.316344	[redacted]
chr16	56569473	56569529	[redacted]	-0.316341	[redacted]
chr10	32773049	32773127	[redacted]	-0.316341	[redacted]
chr7	100583394	100583465	[redacted]	-0.316313	[redacted]
chrX	151109325	151109406	[redacted]	-0.316304	[redacted]
chr12	76249523	76249621	[redacted]	-0.316301	[redacted]
chr16	85358322	85358382	[redacted]	-0.316301	[redacted]
chr5	111695433	111695492	[redacted]	-0.316293	[redacted]
chr8	110369325	110369355	[redacted]	-0.316281	[redacted]
chr11	100702462	100702495	[redacted]	-0.316262	[redacted]
chrX	38945270	38945362	[redacted]	-0.316237	[redacted]
chr6	32689123	32689167	[redacted]	-0.316223	[redacted]
chr12	106795631	106795691	[redacted]	-0.316222	[redacted]
chr6	70028115	70028185	[redacted]	-0.316203	[redacted]
chr9	24307552	24307610	[redacted]	-0.316188	[redacted]
chr19	28682142	28682321	[redacted]	-0.316182	[redacted]
chr10	105417490	105417547	[redacted]	-0.316181	[redacted]
chr9	99504578	99504649	[redacted]	-0.316181	[redacted]
chr14	61259240	61259294	[redacted]	-0.316179	[redacted]
chr13	17852644	17852662	[redacted]	-0.316155	[redacted]
chr5	21880238	21880298	[redacted]	-0.316138	[redacted]
chr17	31529451	31529584	[redacted]	-0.316109	[redacted]
chr4	44296432	44296499	[redacted]	-0.316101	[redacted]
chr1	175810253	175810319	[redacted]	-0.316096	[redacted]
chr7	61493656	61493713	[redacted]	-0.316057	[redacted]
chr11	40746493	40746569	[redacted]	-0.316044	[redacted]
chr13	29570694	29570735	[redacted]	-0.316009	[redacted]
chr19	23119691	23119728	[redacted]	-0.315993	[redacted]

chr2	122400509	122400525	[redacted]	-0.315988	[redacted]
chr4	71421401	71421475	[redacted]	-0.315978	[redacted]
chr1	181623801	181623831	[redacted]	-0.315977	[redacted]
chr13	61836594	61836633	[redacted]	-0.315969	[redacted]
chrX	139438228	139438292	[redacted]	-0.315957	[redacted]
chr16	91743146	91743199	[redacted]	-0.315948	[redacted]
chr10	26509080	26509153	[redacted]	-0.315935	[redacted]
chr17	72905279	72905343	[redacted]	-0.315917	[redacted]
chr16	79519245	79519286	[redacted]	-0.315916	[redacted]
chr9	110517557	110517650	[redacted]	-0.315914	[redacted]
chr4	83325213	83325270	[redacted]	-0.31591	[redacted]
chr9	52933064	52933117	[redacted]	-0.31591	[redacted]
chr11	6193317	6193349	[redacted]	-0.315907	[redacted]
chr3	83052601	83052640	[redacted]	-0.315905	[redacted]
chr13	90803397	90803421	[redacted]	-0.315864	[redacted]
chr9	119959268	119959296	[redacted]	-0.315831	[redacted]
chr2	61257275	61257297	[redacted]	-0.315826	[redacted]
chr2	75045850	75045920	[redacted]	-0.315814	[redacted]
chr7	102080476	102080560	[redacted]	-0.315795	[redacted]
chr3	145630647	145630743	[redacted]	-0.315792	[redacted]
chr19	23870893	23870946	[redacted]	-0.315775	[redacted]
chr5	76483395	76483472	[redacted]	-0.31576	[redacted]
chr7	81083820	81083859	[redacted]	-0.315747	[redacted]
chr5	73845600	73845623	[redacted]	-0.315734	[redacted]
chr16	85206810	85206872	[redacted]	-0.315727	[redacted]
chr13	95165171	95165253	[redacted]	-0.315725	[redacted]
chr9	106564019	106564091	[redacted]	-0.315719	[redacted]
chr15	12431910	12431954	[redacted]	-0.315702	[redacted]
chr1	133779826	133779884	[redacted]	-0.3157	[redacted]
chr11	71033976	71034031	[redacted]	-0.315675	[redacted]
chr14	63977982	63978042	[redacted]	-0.315658	[redacted]
chr10	75289140	75289182	[redacted]	-0.315657	[redacted]
chr11	49104367	49104401	[redacted]	-0.31564	[redacted]
chr10	12609913	12609958	[redacted]	-0.315631	[redacted]
chr14	18679882	18679936	[redacted]	-0.315628	[redacted]
chr16	3129912	3129984	[redacted]	-0.315626	[redacted]
chr12	107210434	107210483	[redacted]	-0.315612	[redacted]
chr12	31326708	31326750	[redacted]	-0.315611	[redacted]
chrX	137595807	137595866	[redacted]	-0.315605	[redacted]
chr16	24426716	24426779	[redacted]	-0.315597	[redacted]
chr13	17280256	17280314	[redacted]	-0.315587	[redacted]
chr13	94482949	94483000	[redacted]	-0.315583	[redacted]
chr12	106220253	106220302	[redacted]	-0.315569	[redacted]

chr2	58380879	58380938	[redacted]	-0.315567	[redacted]
chr4	4165557	4165615	[redacted]	-0.315564	[redacted]
chr15	12048757	12048799	[redacted]	-0.315555	[redacted]
chr13	59782083	59782203	[redacted]	-0.315553	[redacted]
chr5	97467410	97467446	[redacted]	-0.31555	[redacted]
chr2	21986152	21986195	[redacted]	-0.315527	[redacted]
chr1	189362392	189362454	[redacted]	-0.315518	[redacted]
chr18	66465267	66465332	[redacted]	-0.315513	[redacted]
chr17	77763295	77763348	[redacted]	-0.315505	[redacted]
chr11	101163206	101163225	[redacted]	-0.315499	[redacted]
chr8	103997668	103997691	[redacted]	-0.315495	[redacted]
chr13	103421401	103421535	[redacted]	-0.315486	[redacted]
chr2	166929774	166929824	[redacted]	-0.315485	[redacted]
chr13	24827669	24827706	[redacted]	-0.315481	[redacted]
chr17	28453644	28453744	[redacted]	-0.315477	[redacted]
chr1	190041123	190041178	[redacted]	-0.315473	[redacted]
chr7	106775863	106775937	[redacted]	-0.315463	[redacted]
chr3	100779027	100779070	[redacted]	-0.315461	[redacted]
chr5	98088124	98088164	[redacted]	-0.315435	[redacted]
chr13	106913951	106914007	[redacted]	-0.315416	[redacted]
chr14	98398227	98398293	[redacted]	-0.315413	[redacted]
chr12	54304000	54304028	[redacted]	-0.315413	[redacted]
chr4	46035376	46035427	[redacted]	-0.315401	[redacted]
chr7	142866783	142866835	[redacted]	-0.315393	[redacted]
chr1	175959232	175959299	[redacted]	-0.31539	[redacted]
chr3	58577932	58577976	[redacted]	-0.315368	[redacted]
chr16	32523941	32524031	[redacted]	-0.315361	[redacted]
chr15	91310457	91310515	[redacted]	-0.315348	[redacted]
chr1	87947184	87947238	[redacted]	-0.315338	[redacted]
chr5	6159774	6159846	[redacted]	-0.315334	[redacted]
chr8	22189636	22189814	[redacted]	-0.315318	[redacted]
chr2	70955941	70956017	[redacted]	-0.315315	[redacted]
chr2	157650304	157650343	[redacted]	-0.315308	[redacted]
chr9	49655802	49655871	[redacted]	-0.315289	[redacted]
chr1	74051952	74052023	[redacted]	-0.315282	[redacted]
chr5	129121745	129121821	[redacted]	-0.31528	[redacted]
chr18	74196565	74196691	[redacted]	-0.315262	[redacted]
chr11	104074520	104074580	[redacted]	-0.315257	[redacted]
chr8	18603567	18603615	[redacted]	-0.315233	[redacted]
chr12	48392499	48392550	[redacted]	-0.315221	[redacted]
chr10	23821429	23821482	[redacted]	-0.315203	[redacted]
chr15	95608817	95608890	[redacted]	-0.3152	[redacted]
chr11	99231678	99231721	[redacted]	-0.315169	[redacted]

chr4	45563025	45563150	[redacted]	-0.315152	[redacted]
chr6	67229123	67229169	[redacted]	-0.315149	[redacted]
chr6	18026110	18026159	[redacted]	-0.315139	[redacted]
chr8	33993540	33993599	[redacted]	-0.315139	[redacted]
chr13	98900076	98900114	[redacted]	-0.315136	[redacted]
chr7	80724458	80724559	[redacted]	-0.315129	[redacted]
chr14	67545173	67545229	[redacted]	-0.315129	[redacted]
chr10	121905984	121906044	[redacted]	-0.315116	[redacted]
chr6	50303350	50303356	[redacted]	-0.315083	[redacted]
chr11	68756992	68757022	[redacted]	-0.315081	[redacted]
chr7	104596491	104596533	[redacted]	-0.315081	[redacted]
chr2	25488844	25488912	[redacted]	-0.31508	[redacted]
chr11	44550009	44550073	[redacted]	-0.315073	[redacted]
chr11	80970796	80970854	[redacted]	-0.315059	[redacted]
chr4	147412644	147412697	[redacted]	-0.315053	[redacted]
chr6	73463479	73463547	[redacted]	-0.315035	[redacted]
chr5	113867643	113867652	[redacted]	-0.315033	[redacted]
chr1	74386458	74386494	[redacted]	-0.315031	[redacted]
chr16	35273019	35273092	[redacted]	-0.315002	[redacted]
chr10	103230341	103230389	[redacted]	-0.314981	[redacted]
chr7	46662219	46662231	[redacted]	-0.314954	[redacted]
chr17	65582413	65582480	[redacted]	-0.314928	[redacted]
chr6	19694972	19695045	[redacted]	-0.314922	[redacted]
chr7	132804591	132804629	[redacted]	-0.314914	[redacted]
chr2	114526203	114526252	[redacted]	-0.314911	[redacted]
chr3	9717829	9717865	[redacted]	-0.314898	[redacted]
chr10	20206777	20206817	[redacted]	-0.314897	[redacted]
chr7	86599541	86599623	[redacted]	-0.314895	[redacted]
chr16	49757159	49757240	[redacted]	-0.314894	[redacted]
chr6	72267261	72267286	[redacted]	-0.314884	[redacted]
chr13	49009267	49009318	[redacted]	-0.314866	[redacted]
chr15	16579609	16579650	[redacted]	-0.314859	[redacted]
chr18	35558373	35558434	[redacted]	-0.314843	[redacted]
chr7	104222968	104223041	[redacted]	-0.314841	[redacted]
chr6	73715795	73715853	[redacted]	-0.314827	[redacted]
chr3	142166107	142166157	[redacted]	-0.314812	[redacted]
chr14	104411729	104411809	[redacted]	-0.31481	[redacted]
chr11	73319156	73319185	[redacted]	-0.314806	[redacted]
chr3	122059382	122059446	[redacted]	-0.3148	[redacted]
chr6	12149383	12149417	[redacted]	-0.314774	[redacted]
chr17	85703403	85703421	[redacted]	-0.314773	[redacted]
chr18	73697384	73697508	[redacted]	-0.31477	[redacted]
chr6	54653048	54653129	[redacted]	-0.314769	[redacted]

chr2	171186153	171186224	[redacted]	-0.314736	[redacted]
chr5	36998666	36998738	[redacted]	-0.314725	[redacted]
chr15	89019486	89019547	[redacted]	-0.314724	[redacted]
chr5	41371773	41371817	[redacted]	-0.314723	[redacted]
chr14	10700248	10700296	[redacted]	-0.31472	[redacted]
chr7	133635028	133635085	[redacted]	-0.314713	[redacted]
chr11	80266979	80267055	[redacted]	-0.314707	[redacted]
chr14	36368625	36368688	[redacted]	-0.3147	[redacted]
chr2	114107657	114107737	[redacted]	-0.314676	[redacted]
chr2	56010499	56010530	[redacted]	-0.314673	[redacted]
chr4	11248658	11248691	[redacted]	-0.314672	[redacted]
chr16	69733345	69733427	[redacted]	-0.314672	[redacted]
chr7	53325697	53325752	[redacted]	-0.314661	[redacted]
chr13	6420420	6420480	[redacted]	-0.314647	[redacted]
chr4	96803865	96803929	[redacted]	-0.314639	[redacted]
chr16	77306475	77306573	[redacted]	-0.314636	[redacted]
chr11	117548332	117548377	[redacted]	-0.314611	[redacted]
chr16	43475759	43475821	[redacted]	-0.314604	[redacted]
chr18	68355114	68355156	[redacted]	-0.314587	[redacted]
chr3	8512769	8512855	[redacted]	-0.314583	[redacted]
chr7	6605941	6605949	[redacted]	-0.314575	[redacted]
chr5	146093158	146093222	[redacted]	-0.314566	[redacted]
chr14	99820660	99820718	[redacted]	-0.314565	[redacted]
chr9	72799741	72799777	[redacted]	-0.31455	[redacted]
chr9	61024007	61024147	[redacted]	-0.314544	[redacted]
chr4	147502812	147502897	[redacted]	-0.314536	[redacted]
chr2	3735250	3735289	[redacted]	-0.31453	[redacted]
chr15	32535072	32535141	[redacted]	-0.314525	[redacted]
chr17	12296917	12296970	[redacted]	-0.314479	[redacted]
chr6	42323421	42323462	[redacted]	-0.314461	[redacted]
chr15	40842821	40842880	[redacted]	-0.314453	[redacted]
chr7	90973542	90973609	[redacted]	-0.314452	[redacted]
chr11	110030442	110030548	[redacted]	-0.314439	[redacted]
chr8	89542630	89542700	[redacted]	-0.314437	[redacted]
chr11	114345289	114345389	[redacted]	-0.31442	[redacted]
chr6	112527118	112527183	[redacted]	-0.314413	[redacted]
chr13	60306416	60306484	[redacted]	-0.314402	[redacted]
chr11	4347428	4347481	[redacted]	-0.314399	[redacted]
chr6	135794329	135794396	[redacted]	-0.314389	[redacted]
chr11	59943140	59943229	[redacted]	-0.314388	[redacted]
chr15	100412817	100412908	[redacted]	-0.31438	[redacted]
chr4	47412565	47412605	[redacted]	-0.314373	[redacted]
chr6	148925367	148925399	[redacted]	-0.314359	[redacted]

chr5	105875440	105875538	[redacted]	-0.314356	[redacted]
chr10	22747846	22747881	[redacted]	-0.314349	[redacted]
chrX	7485813	7485859	[redacted]	-0.314346	[redacted]
chr9	89992735	89992882	[redacted]	-0.314344	[redacted]
chr18	90416772	90416842	[redacted]	-0.314339	[redacted]
chr18	80352500	80352534	[redacted]	-0.314329	[redacted]
chr15	19787688	19787779	[redacted]	-0.314324	[redacted]
chr15	81280415	81280481	[redacted]	-0.314314	[redacted]
chr7	114802641	114802663	[redacted]	-0.314291	[redacted]
chr6	22076257	22076321	[redacted]	-0.314286	[redacted]
chr17	56867123	56867294	[redacted]	-0.314281	[redacted]
chr11	51601357	51601394	[redacted]	-0.314242	[redacted]
chr2	164725747	164725795	[redacted]	-0.314239	[redacted]
chr14	101752359	101752411	[redacted]	-0.314234	[redacted]
chr10	20695762	20695800	[redacted]	-0.314198	[redacted]
chr11	101804841	101804884	[redacted]	-0.314198	[redacted]
chr16	59803762	59803845	[redacted]	-0.314198	[redacted]
chr2	132343637	132343723	[redacted]	-0.314195	[redacted]
chr8	110062582	110062644	[redacted]	-0.314193	[redacted]
chr4	118091030	118091103	[redacted]	-0.314186	[redacted]
chr19	37232570	37232637	[redacted]	-0.314173	[redacted]
chr19	5364790	5364855	[redacted]	-0.314125	[redacted]
chr8	30734334	30734381	[redacted]	-0.314121	[redacted]
chr12	118233914	118233935	[redacted]	-0.314119	[redacted]
chr9	22448618	22448625	[redacted]	-0.314077	[redacted]
chr4	118023532	118023601	[redacted]	-0.314053	[redacted]
chr4	99601339	99601381	[redacted]	-0.314045	[redacted]
chr15	76065636	76065690	[redacted]	-0.31398	[redacted]
chr7	109354191	109354226	[redacted]	-0.313974	[redacted]
chr15	22892520	22892562	[redacted]	-0.313954	[redacted]
chr6	37876646	37876665	[redacted]	-0.313936	[redacted]
chr8	11482360	11482436	[redacted]	-0.313933	[redacted]
chr14	95595746	95595799	[redacted]	-0.31393	[redacted]
chr9	114810765	114810836	[redacted]	-0.313901	[redacted]
chr5	44298378	44298426	[redacted]	-0.313901	[redacted]
chr1	101933146	101933179	[redacted]	-0.313899	[redacted]
chr3	9783756	9783820	[redacted]	-0.313899	[redacted]
chr15	75750631	75750698	[redacted]	-0.313896	[redacted]
chr9	94838991	94839032	[redacted]	-0.313881	[redacted]
chr4	39911933	39911966	[redacted]	-0.313871	[redacted]
chrX	57217647	57217732	[redacted]	-0.313868	[redacted]
chr2	165095456	165095555	[redacted]	-0.313865	[redacted]
chr9	22198450	22198513	[redacted]	-0.313848	[redacted]

chr15	77764542	77764607	[redacted]	-0.313845	[redacted]
chr8	35204406	35204444	[redacted]	-0.313842	[redacted]
chr6	83159804	83159859	[redacted]	-0.31382	[redacted]
chr19	52199334	52199397	[redacted]	-0.313813	[redacted]
chr17	28389666	28389751	[redacted]	-0.313802	[redacted]
chr2	145623914	145623965	[redacted]	-0.313797	[redacted]
chr7	109106528	109106578	[redacted]	-0.313786	[redacted]
chr2	170594110	170594200	[redacted]	-0.313781	[redacted]
chr5	118288227	118288253	[redacted]	-0.313781	[redacted]
chr16	84925908	84925954	[redacted]	-0.313765	[redacted]
chr3	74660280	74660329	[redacted]	-0.313765	[redacted]
chr3	54753538	54753633	[redacted]	-0.313763	[redacted]
chr18	56917244	56917274	[redacted]	-0.313761	[redacted]
chr8	7993114	7993140	[redacted]	-0.31376	[redacted]
chr11	121883683	121883711	[redacted]	-0.313756	[redacted]
chr10	60266418	60266510	[redacted]	-0.313749	[redacted]
chr6	5257229	5257300	[redacted]	-0.31374	[redacted]
chr3	145512293	145512331	[redacted]	-0.313706	[redacted]
chr7	4249314	4249382	[redacted]	-0.313695	[redacted]
chr14	12909629	12909702	[redacted]	-0.313695	[redacted]
chr2	67618627	67618693	[redacted]	-0.313687	[redacted]
chr18	49533978	49534029	[redacted]	-0.313686	[redacted]
chr5	52691361	52691396	[redacted]	-0.313682	[redacted]
chr16	29457989	29458056	[redacted]	-0.313662	[redacted]
chr4	62502798	62502810	[redacted]	-0.313661	[redacted]
chr5	131937982	131938017	[redacted]	-0.313655	[redacted]
chr11	103663448	103663493	[redacted]	-0.313651	[redacted]
chr16	42214896	42214941	[redacted]	-0.31365	[redacted]
chr13	58838511	58838578	[redacted]	-0.313649	[redacted]
chr3	8586485	8586545	[redacted]	-0.313647	[redacted]
chr17	71870381	71870418	[redacted]	-0.313614	[redacted]
chr9	41995171	41995267	[redacted]	-0.313583	[redacted]
chr2	172391956	172391993	[redacted]	-0.313583	[redacted]
chr10	90058637	90058719	[redacted]	-0.313581	[redacted]
chr14	33086176	33086316	[redacted]	-0.313577	[redacted]
chr9	31586614	31586673	[redacted]	-0.313574	[redacted]
chr12	77211762	77211815	[redacted]	-0.313568	[redacted]
chr3	10071004	10071016	[redacted]	-0.313565	[redacted]
chr6	71188905	71188991	[redacted]	-0.31356	[redacted]
chr12	42173158	42173190	[redacted]	-0.313554	[redacted]
chr7	114689162	114689224	[redacted]	-0.313554	[redacted]
chr11	66981920	66981973	[redacted]	-0.313546	[redacted]
chr9	65043743	65043818	[redacted]	-0.313535	[redacted]

chr2	71867244	71867295	[redacted]	-0.313528	[redacted]
chr2	180827580	180827633	[redacted]	-0.313521	[redacted]
chr4	145316984	145317042	[redacted]	-0.313494	[redacted]
chr2	6920540	6920614	[redacted]	-0.313483	[redacted]
chr6	6491763	6491822	[redacted]	-0.313462	[redacted]
chr12	54861569	54861596	[redacted]	-0.313452	[redacted]
chr1	160007881	160007955	[redacted]	-0.313448	[redacted]
chr13	104603604	104603620	[redacted]	-0.313428	[redacted]
chr17	25418137	25418159	[redacted]	-0.313428	[redacted]
chr7	25594331	25594373	[redacted]	-0.313417	[redacted]
chr18	10041255	10041281	[redacted]	-0.313408	[redacted]
chr18	81399013	81399044	[redacted]	-0.313395	[redacted]
chr17	12102368	12102391	[redacted]	-0.313386	[redacted]
chr11	7870792	7870863	[redacted]	-0.313384	[redacted]
chr12	117010095	117010128	[redacted]	-0.31337	[redacted]
chr1	63896460	63896475	[redacted]	-0.313369	[redacted]
chr8	84773514	84773558	[redacted]	-0.313366	[redacted]
chr7	30702004	30702055	[redacted]	-0.313351	[redacted]
chr1	145029542	145029618	[redacted]	-0.313346	[redacted]
chr5	35805603	35805647	[redacted]	-0.313332	[redacted]
chr1	96045467	96045553	[redacted]	-0.313331	[redacted]
chr1	33766644	33766735	[redacted]	-0.313327	[redacted]
chr6	64509793	64509844	[redacted]	-0.31331	[redacted]
chr13	38425525	38425558	[redacted]	-0.313299	[redacted]
chr10	36882195	36882230	[redacted]	-0.313292	[redacted]
chr6	124884528	124884564	[redacted]	-0.313288	[redacted]
chr13	54892488	54892522	[redacted]	-0.313287	[redacted]
chr14	105828828	105828892	[redacted]	-0.313286	[redacted]
chr14	56471522	56471593	[redacted]	-0.313284	[redacted]
chrX	51534814	51534877	[redacted]	-0.313282	[redacted]
chr6	35777405	35777461	[redacted]	-0.313272	[redacted]
chr19	34719677	34719726	[redacted]	-0.313225	[redacted]
chr12	39756568	39756623	[redacted]	-0.313209	[redacted]
chrX	159710977	159710996	[redacted]	-0.313198	[redacted]
chr5	74400202	74400245	[redacted]	-0.313196	[redacted]
chr16	14651521	14651548	[redacted]	-0.313194	[redacted]
chr6	43261793	43261872	[redacted]	-0.313187	[redacted]
chr9	85495746	85495803	[redacted]	-0.313172	[redacted]
chr5	149205131	149205202	[redacted]	-0.313171	[redacted]
chr19	55891935	55891984	[redacted]	-0.313151	[redacted]
chr19	34462654	34462701	[redacted]	-0.313149	[redacted]
chr5	66671646	66671724	[redacted]	-0.313139	[redacted]
chr3	136375992	136376042	[redacted]	-0.313134	[redacted]

chr18	37746225	37746276	[redacted]	-0.313133	[redacted]
chr12	103031543	103031608	[redacted]	-0.313125	[redacted]
chr10	84318619	84318681	[redacted]	-0.313115	[redacted]
chr1	93123215	93123272	[redacted]	-0.313103	[redacted]
chr3	65011396	65011433	[redacted]	-0.313102	[redacted]
chr9	22823012	22823083	[redacted]	-0.313101	[redacted]
chr10	23437837	23437877	[redacted]	-0.313095	[redacted]
chr18	36245923	36245967	[redacted]	-0.313088	[redacted]
chr3	58695478	58695552	[redacted]	-0.313087	[redacted]
chr11	70773765	70773827	[redacted]	-0.313045	[redacted]
chr2	141434223	141434304	[redacted]	-0.313045	[redacted]
chr1	162283179	162283252	[redacted]	-0.313036	[redacted]
chr13	112221346	112221428	[redacted]	-0.313034	[redacted]
chr17	67760299	67760352	[redacted]	-0.31303	[redacted]
chr18	50719876	50719923	[redacted]	-0.313027	[redacted]
chr11	34817497	34817580	[redacted]	-0.313022	[redacted]
chr12	60705063	60705105	[redacted]	-0.313017	[redacted]
chr8	15418960	15419055	[redacted]	-0.313016	[redacted]
chr1	52678934	52678975	[redacted]	-0.313008	[redacted]
chr11	96353988	96354087	[redacted]	-0.313007	[redacted]
chr4	148993153	148993245	[redacted]	-0.312999	[redacted]
chr10	124151843	124151882	[redacted]	-0.312997	[redacted]
chr12	103159723	103159798	[redacted]	-0.312995	[redacted]
chr5	80561405	80561464	[redacted]	-0.312983	[redacted]
chr15	51249680	51249752	[redacted]	-0.31297	[redacted]
chr15	92264257	92264308	[redacted]	-0.312959	[redacted]
chr6	76214563	76214642	[redacted]	-0.312957	[redacted]
chr18	43616732	43616768	[redacted]	-0.312956	[redacted]
chr8	63723169	63723222	[redacted]	-0.312952	[redacted]
chr15	27986919	27986969	[redacted]	-0.312943	[redacted]
chr15	76143983	76144057	[redacted]	-0.312938	[redacted]
chr2	148017730	148017764	[redacted]	-0.312933	[redacted]
chr17	84022750	84022792	[redacted]	-0.312921	[redacted]
chr7	105827501	105827609	[redacted]	-0.312918	[redacted]
chr1	40027875	40027890	[redacted]	-0.312914	[redacted]
chr2	66621232	66621325	[redacted]	-0.312904	[redacted]
chr14	9861891	9861951	[redacted]	-0.312889	[redacted]
chr15	5449357	5449421	[redacted]	-0.312883	[redacted]
chr2	67839843	67839889	[redacted]	-0.312881	[redacted]
chrX	23869375	23869457	[redacted]	-0.312879	[redacted]
chr3	32466206	32466312	[redacted]	-0.312871	[redacted]
chr9	83478073	83478152	[redacted]	-0.312848	[redacted]
chr11	119212946	119213006	[redacted]	-0.312846	[redacted]

chr10	117862010	117862072	[redacted]	-0.312845	[redacted]
chr18	80939686	80939748	[redacted]	-0.312843	[redacted]
chrX	95717536	95717615	[redacted]	-0.312831	[redacted]
chr2	31772901	31772964	[redacted]	-0.312825	[redacted]
chr5	149144668	149144707	[redacted]	-0.312816	[redacted]
chr4	82887578	82887661	[redacted]	-0.312815	[redacted]
chr2	141423072	141423148	[redacted]	-0.312802	[redacted]
chr9	110914714	110914782	[redacted]	-0.312796	[redacted]
chr12	41686421	41686479	[redacted]	-0.31278	[redacted]
chr3	157163036	157163130	[redacted]	-0.312779	[redacted]
chr6	54686348	54686430	[redacted]	-0.312741	[redacted]
chrY	90798825	90798906	[redacted]	-0.312737	[redacted]
chr14	31199883	31199936	[redacted]	-0.312728	[redacted]
chr12	98326251	98326319	[redacted]	-0.312721	[redacted]
chr11	83804227	83804280	[redacted]	-0.312715	[redacted]
chr11	45671617	45671644	[redacted]	-0.312707	[redacted]
chr7	134154986	134155037	[redacted]	-0.312705	[redacted]
chr6	30420804	30420848	[redacted]	-0.312701	[redacted]
chr7	18850960	18851027	[redacted]	-0.312654	[redacted]
chr4	122746476	122746522	[redacted]	-0.312653	[redacted]
chr4	8821860	8821908	[redacted]	-0.312643	[redacted]
chr13	66631754	66631785	[redacted]	-0.312641	[redacted]
chr1	162656469	162656571	[redacted]	-0.31264	[redacted]
chr13	95201016	95201101	[redacted]	-0.312628	[redacted]
chr19	28263495	28263568	[redacted]	-0.312624	[redacted]
chr7	140292245	140292301	[redacted]	-0.312563	[redacted]
chr18	34822721	34822746	[redacted]	-0.312562	[redacted]
chr4	98736453	98736490	[redacted]	-0.312558	[redacted]
chr2	61206237	61206286	[redacted]	-0.312537	[redacted]
chr9	49178913	49178937	[redacted]	-0.312519	[redacted]
chr18	72947434	72947459	[redacted]	-0.312514	[redacted]
chr10	68055592	68055667	[redacted]	-0.312508	[redacted]
chr6	26115419	26115477	[redacted]	-0.312493	[redacted]
chr13	46828527	46828641	[redacted]	-0.312471	[redacted]
chr13	112319634	112319701	[redacted]	-0.31247	[redacted]
chr4	105630570	105630648	[redacted]	-0.312465	[redacted]
chr13	89810234	89810298	[redacted]	-0.312443	[redacted]
chr9	8173637	8173682	[redacted]	-0.312441	[redacted]
chr6	124779101	124779144	[redacted]	-0.312438	[redacted]
chr4	52772776	52772853	[redacted]	-0.312435	[redacted]
chr15	73674113	73674174	[redacted]	-0.312418	[redacted]
chr2	101651287	101651349	[redacted]	-0.312408	[redacted]
chr1	139478363	139478418	[redacted]	-0.312396	[redacted]

chr17	11050461	11050506	[redacted]	-0.312392	[redacted]
chr2	33952248	33952394	[redacted]	-0.31239	[redacted]
chr16	72964838	72964899	[redacted]	-0.312373	[redacted]
chr9	108099165	108099193	[redacted]	-0.312364	[redacted]
chr1	105813133	105813153	[redacted]	-0.312361	[redacted]
chr15	78352781	78352811	[redacted]	-0.312348	[redacted]
chr6	149182644	149182675	[redacted]	-0.312331	[redacted]
chr16	43583685	43583711	[redacted]	-0.312317	[redacted]
chr2	46636957	46636986	[redacted]	-0.312286	[redacted]
chr1	56756807	56756877	[redacted]	-0.312285	[redacted]
chr12	56256563	56256629	[redacted]	-0.312271	[redacted]
chr2	106120695	106120738	[redacted]	-0.312261	[redacted]
chr14	120144173	120144234	[redacted]	-0.312256	[redacted]
chr7	95834076	95834149	[redacted]	-0.312254	[redacted]
chr9	40365536	40365618	[redacted]	-0.312237	[redacted]
chr9	78051118	78051158	[redacted]	-0.312228	[redacted]
chr11	106574560	106574610	[redacted]	-0.312211	[redacted]
chr4	11702999	11703053	[redacted]	-0.31219	[redacted]
chr3	21092239	21092290	[redacted]	-0.31219	[redacted]
chr14	85790671	85790732	[redacted]	-0.312189	[redacted]
chr9	95856917	95856969	[redacted]	-0.312186	[redacted]
chr10	110736544	110736609	[redacted]	-0.312174	[redacted]
chr1	92887468	92887516	[redacted]	-0.312173	[redacted]
chr16	23246281	23246335	[redacted]	-0.312157	[redacted]
chr4	115916170	115916245	[redacted]	-0.312133	[redacted]
chr13	62042480	62042517	[redacted]	-0.31213	[redacted]
chr10	54066279	54066341	[redacted]	-0.31211	[redacted]
chr10	48206331	48206389	[redacted]	-0.31211	[redacted]
chr7	115682740	115682772	[redacted]	-0.312109	[redacted]
chr9	102185869	102185899	[redacted]	-0.312094	[redacted]
chr6	139015198	139015253	[redacted]	-0.31209	[redacted]
chr12	76765088	76765135	[redacted]	-0.31209	[redacted]
chr5	115928608	115928690	[redacted]	-0.312086	[redacted]
chr10	57190574	57190643	[redacted]	-0.312084	[redacted]
chr10	20280034	20280114	[redacted]	-0.312079	[redacted]
chr9	111779751	111779900	[redacted]	-0.312077	[redacted]
chr18	30700912	30700925	[redacted]	-0.312074	[redacted]
chr6	10848682	10848740	[redacted]	-0.312065	[redacted]
chr2	91507879	91507947	[redacted]	-0.312052	[redacted]
chr11	98564543	98564624	[redacted]	-0.312047	[redacted]
chr1	180806105	180806246	[redacted]	-0.312045	[redacted]
chr10	25728224	25728294	[redacted]	-0.312039	[redacted]
chr9	107440063	107440171	[redacted]	-0.312035	[redacted]

chr5	100587401	100587504	[redacted]	-0.312033	[redacted]
chr14	118875569	118875606	[redacted]	-0.312024	[redacted]
chr6	52579009	52579036	[redacted]	-0.312018	[redacted]
chr1	173752649	173752686	[redacted]	-0.312015	[redacted]
chr15	50153197	50153270	[redacted]	-0.312013	[redacted]
chr2	68815192	68815246	[redacted]	-0.312007	[redacted]
chr2	115192269	115192285	[redacted]	-0.312007	[redacted]
chr16	34786368	34786436	[redacted]	-0.312	[redacted]
chr2	151048476	151048535	[redacted]	-0.311998	[redacted]
chr19	29980366	29980434	[redacted]	-0.311989	[redacted]
chr18	74927972	74928070	[redacted]	-0.311976	[redacted]
chr13	101303533	101303615	[redacted]	-0.311974	[redacted]
chr11	9356754	9356794	[redacted]	-0.311973	[redacted]
chr4	135369095	135369187	[redacted]	-0.311964	[redacted]
chr8	57835975	57835992	[redacted]	-0.311948	[redacted]
chr10	4996424	4996483	[redacted]	-0.311932	[redacted]
chr1	58150332	58150394	[redacted]	-0.311927	[redacted]
chr12	39770627	39770710	[redacted]	-0.31192	[redacted]
chrX	32099221	32099258	[redacted]	-0.311919	[redacted]
chr10	76759300	76759373	[redacted]	-0.311916	[redacted]
chr8	119608059	119608100	[redacted]	-0.311916	[redacted]
chr15	5887586	5887674	[redacted]	-0.311897	[redacted]
chr11	120082770	120082908	[redacted]	-0.311895	[redacted]
chr7	24734434	24734469	[redacted]	-0.311885	[redacted]
chr15	55193063	55193117	[redacted]	-0.311864	[redacted]
chr19	5832493	5832533	[redacted]	-0.311855	[redacted]
chr8	24023302	24023362	[redacted]	-0.311842	[redacted]
chr6	81518580	81518725	[redacted]	-0.31184	[redacted]
chr14	25339976	25340058	[redacted]	-0.311838	[redacted]
chr11	64034493	64034569	[redacted]	-0.311836	[redacted]
chrX	36742952	36743013	[redacted]	-0.311828	[redacted]
chr8	128375409	128375478	[redacted]	-0.311822	[redacted]
chr7	118097766	118097803	[redacted]	-0.311819	[redacted]
chr1	55310442	55310513	[redacted]	-0.311809	[redacted]
chr8	100788553	100788605	[redacted]	-0.311789	[redacted]
chr1	172184916	172184931	[redacted]	-0.311787	[redacted]
chr10	126812904	126812964	[redacted]	-0.311751	[redacted]
chr4	32840066	32840141	[redacted]	-0.311746	[redacted]
chr19	11813778	11813816	[redacted]	-0.311725	[redacted]
chr11	82838306	82838315	[redacted]	-0.311722	[redacted]
chr2	70099854	70099905	[redacted]	-0.311719	[redacted]
chr10	28694649	28694688	[redacted]	-0.311716	[redacted]
chr11	65358904	65358998	[redacted]	-0.3117	[redacted]

chr3	92900892	92900952	[redacted]	-0.311696	[redacted]
chr17	85166324	85166473	[redacted]	-0.311691	[redacted]
chr6	113419987	113420089	[redacted]	-0.311676	[redacted]
chr1	135851553	135851606	[redacted]	-0.311671	[redacted]
chr4	151855718	151855727	[redacted]	-0.31167	[redacted]
chr8	104606188	104606272	[redacted]	-0.311653	[redacted]
chr11	34052675	34052737	[redacted]	-0.311646	[redacted]
chr5	113077237	113077302	[redacted]	-0.311639	[redacted]
chr10	13782366	13782402	[redacted]	-0.311638	[redacted]
chr12	80814254	80814271	[redacted]	-0.311632	[redacted]
chr7	6617682	6617730	[redacted]	-0.311613	[redacted]
chr5	23450774	23450878	[redacted]	-0.311609	[redacted]
chr8	124493827	124493896	[redacted]	-0.311604	[redacted]
chr4	105747526	105747546	[redacted]	-0.311583	[redacted]
chr11	33380957	33381044	[redacted]	-0.31156	[redacted]
chr1	138643141	138643184	[redacted]	-0.311534	[redacted]
chr8	20524481	20524518	[redacted]	-0.311529	[redacted]
chrX	11523428	11523505	[redacted]	-0.311526	[redacted]
chr15	5165615	5165691	[redacted]	-0.311524	[redacted]
chrX	7550963	7551042	[redacted]	-0.311517	[redacted]
chr11	118238841	118238873	[redacted]	-0.311507	[redacted]
chr18	55841342	55841407	[redacted]	-0.311503	[redacted]
chr15	92672593	92672666	[redacted]	-0.311484	[redacted]
chr12	54915433	54915465	[redacted]	-0.311482	[redacted]
chr2	174508475	174508515	[redacted]	-0.31148	[redacted]
chr9	36944056	36944110	[redacted]	-0.311477	[redacted]
chr7	24806864	24806899	[redacted]	-0.311451	[redacted]
chr19	48914077	48914206	[redacted]	-0.311441	[redacted]
chr1	85950443	85950499	[redacted]	-0.311438	[redacted]
chr11	90990006	90990066	[redacted]	-0.311438	[redacted]
chr17	46489800	46489863	[redacted]	-0.311409	[redacted]
chr6	32877379	32877412	[redacted]	-0.311404	[redacted]
chr10	6663936	6664042	[redacted]	-0.311391	[redacted]
chr19	25355436	25355504	[redacted]	-0.311368	[redacted]
chr18	85301667	85301705	[redacted]	-0.311365	[redacted]
chr10	112749200	112749246	[redacted]	-0.311362	[redacted]
chr2	59041664	59041712	[redacted]	-0.311362	[redacted]
chr11	54055743	54055797	[redacted]	-0.311357	[redacted]
chr2	24642943	24642983	[redacted]	-0.311345	[redacted]
chr11	114938526	114938567	[redacted]	-0.311327	[redacted]
chr15	35184708	35184761	[redacted]	-0.311324	[redacted]
chr8	82018349	82018405	[redacted]	-0.311315	[redacted]
chr5	142548436	142548468	[redacted]	-0.311303	[redacted]

chr11	35830137	35830192	[redacted]	-0.311288	[redacted]
chr6	120007451	120007494	[redacted]	-0.311275	[redacted]
chr19	19224532	19224660	[redacted]	-0.311267	[redacted]
chr7	87829849	87829894	[redacted]	-0.311256	[redacted]
chr4	120527735	120527753	[redacted]	-0.311252	[redacted]
chr13	23280972	23281022	[redacted]	-0.311242	[redacted]
chr13	100878989	100879041	[redacted]	-0.311235	[redacted]
chr11	85960109	85960147	[redacted]	-0.31122	[redacted]
chr10	95052159	95052220	[redacted]	-0.311218	[redacted]
chr3	19119562	19119593	[redacted]	-0.311202	[redacted]
chr19	8530020	8530102	[redacted]	-0.311185	[redacted]
chr2	148894574	148894639	[redacted]	-0.311184	[redacted]
chr16	32769659	32769729	[redacted]	-0.311182	[redacted]
chr10	42880581	42880634	[redacted]	-0.31118	[redacted]
chr1	75210060	75210126	[redacted]	-0.311175	[redacted]
chr12	36812599	36812681	[redacted]	-0.311167	[redacted]
chr5	67497518	67497577	[redacted]	-0.311146	[redacted]
chr11	114632622	114632671	[redacted]	-0.311138	[redacted]
chr16	78852429	78852500	[redacted]	-0.311138	[redacted]
chr10	111879671	111879702	[redacted]	-0.311117	[redacted]
chr6	35808881	35808939	[redacted]	-0.311113	[redacted]
chr2	74117526	74117580	[redacted]	-0.311112	[redacted]
chr7	98584524	98584626	[redacted]	-0.311098	[redacted]
chr15	81184137	81184177	[redacted]	-0.311098	[redacted]
chr2	45059711	45059764	[redacted]	-0.311095	[redacted]
chr1	169644250	169644344	[redacted]	-0.311092	[redacted]
chr15	39822015	39822032	[redacted]	-0.311071	[redacted]
chr19	5897054	5897080	[redacted]	-0.31107	[redacted]
chr17	87972416	87972452	[redacted]	-0.311051	[redacted]
chr5	9643188	9643278	[redacted]	-0.311032	[redacted]
chr10	104116046	104116106	[redacted]	-0.311019	[redacted]
chr9	72478691	72478713	[redacted]	-0.311013	[redacted]
chr14	24887403	24887466	[redacted]	-0.311008	[redacted]
chr17	86368205	86368235	[redacted]	-0.310998	[redacted]
chrX	52120327	52120390	[redacted]	-0.310983	[redacted]
chr8	19684379	19684392	[redacted]	-0.310981	[redacted]
chr2	102190317	102190374	[redacted]	-0.31097	[redacted]
chr2	27429562	27429629	[redacted]	-0.310956	[redacted]
chr12	83504060	83504127	[redacted]	-0.310952	[redacted]
chr5	98100031	98100077	[redacted]	-0.310952	[redacted]
chr16	30671086	30671155	[redacted]	-0.310947	[redacted]
chr19	43987496	43987515	[redacted]	-0.310942	[redacted]
chr9	61400384	61400434	[redacted]	-0.310939	[redacted]

chr3	89875149	89875234	[redacted]	-0.310932	[redacted]
chrUn_	71132	71191	[redacted]	-0.310928	[redacted]
chr6	113058257	113058333	[redacted]	-0.310925	[redacted]
chr7	17012702	17012747	[redacted]	-0.310924	[redacted]
chr4	8831808	8831866	[redacted]	-0.310921	[redacted]
chr17	30845230	30845283	[redacted]	-0.310914	[redacted]
chr6	83844465	83844546	[redacted]	-0.310909	[redacted]
chr6	6404219	6404290	[redacted]	-0.310907	[redacted]
chr8	109963068	109963094	[redacted]	-0.310898	[redacted]
chr6	48099971	48100029	[redacted]	-0.310877	[redacted]
chr9	107560376	107560430	[redacted]	-0.310847	[redacted]
chr12	35202181	35202249	[redacted]	-0.310839	[redacted]
chr1	26607776	26607824	[redacted]	-0.310829	[redacted]
chr14	68663212	68663273	[redacted]	-0.310811	[redacted]
chr11	81839082	81839174	[redacted]	-0.310801	[redacted]
chr7	93028405	93028484	[redacted]	-0.310795	[redacted]
chr2	76423750	76423796	[redacted]	-0.310765	[redacted]
chr16	84601283	84601338	[redacted]	-0.310763	[redacted]
chr5	149144344	149144371	[redacted]	-0.310756	[redacted]
chr2	33975829	33975879	[redacted]	-0.310747	[redacted]
chr1	23856057	23856142	[redacted]	-0.310745	[redacted]
chr3	125122843	125122905	[redacted]	-0.310729	[redacted]
chr18	78106862	78106920	[redacted]	-0.310728	[redacted]
chr6	111917472	111917531	[redacted]	-0.310723	[redacted]
chr3	146290474	146290532	[redacted]	-0.310715	[redacted]
chr3	26550124	26550218	[redacted]	-0.310659	[redacted]
chr11	109210766	109210802	[redacted]	-0.310653	[redacted]
chr18	62658038	62658086	[redacted]	-0.310625	[redacted]
chr10	13963857	13963904	[redacted]	-0.310622	[redacted]
chr2	62249355	62249426	[redacted]	-0.310618	[redacted]
chr15	88419729	88419802	[redacted]	-0.310613	[redacted]
chr17	4500028	4500074	[redacted]	-0.310596	[redacted]
chr2	26871007	26871078	[redacted]	-0.310594	[redacted]
chr17	87984536	87984580	[redacted]	-0.310579	[redacted]
chr2	21934322	21934383	[redacted]	-0.310534	[redacted]
chr6	86247608	86247681	[redacted]	-0.310531	[redacted]
chr1	191202688	191202740	[redacted]	-0.310526	[redacted]
chr12	28849580	28849713	[redacted]	-0.310508	[redacted]
chr19	58542239	58542270	[redacted]	-0.310504	[redacted]
chr6	125218200	125218233	[redacted]	-0.310503	[redacted]
chr12	73849674	73849745	[redacted]	-0.310496	[redacted]
chr5	118671110	118671139	[redacted]	-0.310495	[redacted]
chr10	79908087	79908128	[redacted]	-0.310456	[redacted]

chr6	96363864	96363934		-0.310455	
chr10	42420529	42420571		-0.310453	
chr17	42431913	42431974		-0.310453	
chr6	51907716	51907769		-0.31044	
chr19	44341980	44342017		-0.310439	
chr11	54774207	54774275		-0.310425	
chr8	83259430	83259489		-0.31042	
chr4	42899700	42899754		-0.310399	
chr1	138736496	138736574		-0.310398	
chr1	86312226	86312289		-0.310345	
chr2	132206809	132206892		-0.310336	
chr8	13612418	13612461		-0.310291	
chr10	89667242	89667315		-0.310291	
chr4	98946706	98946754		-0.310268	
chr2	90771648	90771713		-0.310262	
chr13	56583663	56583705		-0.310254	
chr2	84484022	84484073		-0.31024	
chr6	134291281	134291377		-0.310228	
chr15	81327833	81327901		-0.310221	
chr8	101627209	101627301		-0.310217	
chr7	4470721	4470821		-0.310211	
chr3	52839610	52839632		-0.310188	
chr15	10511147	10511183		-0.310173	
chr4	16564300	16564336		-0.310152	
chr14	84059324	84059378		-0.31015	
chr6	22236371	22236381		-0.310149	
chr10	95892253	95892326		-0.310134	
chr18	11021942	11022000		-0.310128	
chr14	8877083	8877160		-0.31011	
chr8	97743661	97743751		-0.31009	
chr16	82565372	82565417		-0.310089	
chr1	33346033	33346061		-0.310089	
chr15	78102098	78102143		-0.310076	
chr1	159666681	159666705		-0.310073	
chr18	82860227	82860278		-0.310069	
chr3	80122751	80122836		-0.310064	
chr12	98967988	98968040		-0.310064	
chrX	59742879	59742970		-0.31006	
chr8	31923419	31923510		-0.310054	
chr18	58201154	58201209		-0.310021	
chr18	70583413	70583468		-0.310009	
chr9	53299820	53299862		-0.309971	
chr11	77088191	77088211		-0.309965	

chrX	89837837	89837868	[redacted]	-0.309963	[redacted]
chr2	145449107	145449162	[redacted]	-0.309949	[redacted]
chr7	31492595	31492624	[redacted]	-0.309943	[redacted]
chr15	47028305	47028359	[redacted]	-0.309941	[redacted]
chr17	89271385	89271475	[redacted]	-0.30994	[redacted]
chr13	112675991	112676104	[redacted]	-0.309937	[redacted]
chr9	60596717	60596776	[redacted]	-0.309936	[redacted]
chr16	92057360	92057415	[redacted]	-0.309908	[redacted]
chr12	104956405	104956471	[redacted]	-0.309903	[redacted]
chr9	6002778	6002840	[redacted]	-0.309896	[redacted]
chr6	29599946	29599991	[redacted]	-0.30989	[redacted]
chr2	85220373	85220458	[redacted]	-0.309888	[redacted]
chr13	35028734	35028789	[redacted]	-0.309877	[redacted]
chr12	89793164	89793231	[redacted]	-0.309874	[redacted]
chr7	96306868	96306954	[redacted]	-0.309874	[redacted]
chr1	172457786	172457990	[redacted]	-0.309872	[redacted]
chr15	86454655	86454684	[redacted]	-0.309859	[redacted]
chr8	36721227	36721312	[redacted]	-0.309851	[redacted]
chr8	25061878	25061934	[redacted]	-0.309845	[redacted]
chr10	76788831	76788888	[redacted]	-0.309844	[redacted]
chr17	88262481	88262528	[redacted]	-0.309841	[redacted]
chr18	89450923	89450988	[redacted]	-0.309822	[redacted]
chr17	64483713	64483823	[redacted]	-0.309813	[redacted]
chr3	65477036	65477074	[redacted]	-0.309812	[redacted]
chr9	58692387	58692482	[redacted]	-0.309763	[redacted]
chr5	69722648	69722711	[redacted]	-0.309761	[redacted]
chr19	37413152	37413232	[redacted]	-0.309749	[redacted]
chr12	69848437	69848486	[redacted]	-0.309745	[redacted]
chr9	7509761	7509781	[redacted]	-0.309742	[redacted]
chr17	38165643	38165714	[redacted]	-0.30974	[redacted]
chr3	123580327	123580387	[redacted]	-0.309724	[redacted]
chr4	64005716	64005774	[redacted]	-0.30972	[redacted]
chr11	49828187	49828254	[redacted]	-0.309719	[redacted]
chr7	90401333	90401371	[redacted]	-0.309703	[redacted]
chr16	55824423	55824527	[redacted]	-0.3097	[redacted]
chr15	85456941	85457028	[redacted]	-0.309682	[redacted]
chr2	11057583	11057641	[redacted]	-0.309668	[redacted]
chr16	38319002	38319073	[redacted]	-0.309662	[redacted]
chr11	87525871	87525932	[redacted]	-0.309645	[redacted]
chr7	33452790	33452851	[redacted]	-0.309626	[redacted]
chr1	171040497	171040553	[redacted]	-0.309625	[redacted]
chr1	59167041	59167078	[redacted]	-0.309623	[redacted]
chr19	55476567	55476617	[redacted]	-0.309615	[redacted]

chr10	76971619	76971717	[redacted]	-0.309598	[redacted]
chr2	152592087	152592139	[redacted]	-0.309596	[redacted]
chr8	27118169	27118237	[redacted]	-0.309594	[redacted]
chr9	29700083	29700126	[redacted]	-0.309589	[redacted]
chr3	90095595	90095619	[redacted]	-0.309585	[redacted]
chr4	58935377	58935451	[redacted]	-0.309585	[redacted]
chr4	62778315	62778408	[redacted]	-0.309581	[redacted]
chr2	125481805	125481865	[redacted]	-0.30957	[redacted]
chr19	5075917	5075940	[redacted]	-0.309563	[redacted]
chr15	82957612	82957745	[redacted]	-0.309548	[redacted]
chr5	92367583	92367632	[redacted]	-0.309533	[redacted]
chr9	120877697	120877750	[redacted]	-0.309531	[redacted]
chr2	60828984	60829037	[redacted]	-0.309525	[redacted]
chr6	116613295	116613333	[redacted]	-0.309507	[redacted]
chr6	21505190	21505253	[redacted]	-0.309506	[redacted]
chr15	18944866	18944912	[redacted]	-0.309499	[redacted]
chr5	88789262	88789320	[redacted]	-0.309447	[redacted]
chr18	47171767	47171869	[redacted]	-0.309438	[redacted]
chr15	53803165	53803206	[redacted]	-0.309438	[redacted]
chr9	96916544	96916591	[redacted]	-0.309435	[redacted]
chr6	83155550	83155577	[redacted]	-0.309434	[redacted]
chr3	130251300	130251362	[redacted]	-0.309425	[redacted]
chr2	179934193	179934263	[redacted]	-0.30942	[redacted]
chr9	102680422	102680482	[redacted]	-0.309413	[redacted]
chr3	151811713	151811789	[redacted]	-0.309403	[redacted]
chr12	4906154	4906177	[redacted]	-0.3094	[redacted]
chr15	55116263	55116315	[redacted]	-0.309396	[redacted]
chr9	18095442	18095483	[redacted]	-0.309394	[redacted]
chr5	10930552	10930632	[redacted]	-0.309386	[redacted]
chr19	11364861	11364926	[redacted]	-0.309381	[redacted]
chr15	8847141	8847213	[redacted]	-0.309371	[redacted]
chr11	59253151	59253215	[redacted]	-0.309357	[redacted]
chr2	65397294	65397351	[redacted]	-0.309351	[redacted]
chr9	31437865	31437889	[redacted]	-0.309349	[redacted]
chr9	37387019	37387079	[redacted]	-0.309336	[redacted]
chr15	6806708	6806752	[redacted]	-0.309328	[redacted]
chr9	43871536	43871565	[redacted]	-0.30932	[redacted]
chr10	121636511	121636567	[redacted]	-0.309319	[redacted]
chr12	87008427	87008479	[redacted]	-0.309318	[redacted]
chr14	17630745	17630810	[redacted]	-0.309305	[redacted]
chr1	130764433	130764465	[redacted]	-0.309304	[redacted]
chr14	36895191	36895265	[redacted]	-0.309288	[redacted]
chr17	75768699	75768772	[redacted]	-0.309265	[redacted]

chr10	14066028	14066136	[redacted]	-0.309256	[redacted]
chr12	8221739	8221795	[redacted]	-0.309252	[redacted]
chr19	18094487	18094553	[redacted]	-0.309225	[redacted]
chr2	41204761	41204829	[redacted]	-0.309223	[redacted]
chr2	152426047	152426099	[redacted]	-0.309207	[redacted]
chr1	79862382	79862458	[redacted]	-0.309201	[redacted]
chr12	79455428	79455494	[redacted]	-0.309197	[redacted]
chr13	57742679	57742722	[redacted]	-0.309183	[redacted]
chr18	34917991	34918018	[redacted]	-0.309165	[redacted]
chr8	25906043	25906067	[redacted]	-0.309157	[redacted]
chr1	72210632	72210679	[redacted]	-0.309147	[redacted]
chr3	116734566	116734575	[redacted]	-0.309147	[redacted]
chr1	41670261	41670317	[redacted]	-0.309141	[redacted]
chr4	94268604	94268658	[redacted]	-0.309119	[redacted]
chr16	10112397	10112494	[redacted]	-0.309097	[redacted]
chr1	78092907	78092967	[redacted]	-0.309077	[redacted]
chr4	62770160	62770235	[redacted]	-0.309073	[redacted]
chr7	60670131	60670203	[redacted]	-0.309061	[redacted]
chr9	11584670	11584719	[redacted]	-0.309056	[redacted]
chr1	30333866	30333942	[redacted]	-0.309055	[redacted]
chr15	9992660	9992753	[redacted]	-0.309031	[redacted]
chr1	184543248	184543335	[redacted]	-0.309006	[redacted]
chr6	72645285	72645325	[redacted]	-0.308974	[redacted]
chr15	25800285	25800379	[redacted]	-0.308968	[redacted]
chr17	71504699	71504753	[redacted]	-0.308916	[redacted]
chr11	16542642	16542687	[redacted]	-0.308914	[redacted]
chr17	65509425	65509492	[redacted]	-0.3089	[redacted]
chr15	82063445	82063494	[redacted]	-0.308869	[redacted]
chr5	129955958	129955998	[redacted]	-0.308865	[redacted]
chr15	53450393	53450463	[redacted]	-0.308862	[redacted]
chr6	23478915	23479006	[redacted]	-0.30886	[redacted]
chr3	148335338	148335418	[redacted]	-0.308853	[redacted]
chr12	111457281	111457361	[redacted]	-0.308851	[redacted]
chr9	55207053	55207091	[redacted]	-0.308848	[redacted]
chr16	77684332	77684408	[redacted]	-0.308847	[redacted]
chr14	21585604	21585628	[redacted]	-0.308832	[redacted]
chr5	37053325	37053362	[redacted]	-0.308829	[redacted]
chrUn_	32220	32337	[redacted]	-0.308827	[redacted]
chr3	41175198	41175275	[redacted]	-0.308826	[redacted]
chr17	26476375	26476407	[redacted]	-0.308821	[redacted]
chr3	137901928	137902001	[redacted]	-0.308814	[redacted]
chr16	57669091	57669163	[redacted]	-0.308809	[redacted]
chr12	99531467	99531534	[redacted]	-0.308793	[redacted]

chr2	137342255	137342295	[redacted]	-0.308781	[redacted]
chr7	54467357	54467388	[redacted]	-0.308771	[redacted]
chr9	61156178	61156225	[redacted]	-0.308751	[redacted]
chr1	86416705	86416750	[redacted]	-0.308746	[redacted]
chr14	17761877	17761960	[redacted]	-0.308737	[redacted]
chr1	57521036	57521063	[redacted]	-0.308721	[redacted]
chr3	134136253	134136300	[redacted]	-0.308719	[redacted]
chr9	76536386	76536466	[redacted]	-0.308712	[redacted]
chr7	112294395	112294417	[redacted]	-0.308703	[redacted]
chr13	99433221	99433404	[redacted]	-0.3087	[redacted]
chr12	52738142	52738192	[redacted]	-0.3087	[redacted]
chr19	59517882	59517966	[redacted]	-0.308677	[redacted]
chr19	9929667	9929724	[redacted]	-0.308659	[redacted]
chr13	16191583	16191619	[redacted]	-0.308654	[redacted]
chr17	56313887	56313958	[redacted]	-0.308652	[redacted]
chr18	90273712	90273789	[redacted]	-0.308643	[redacted]
chr13	105037732	105037787	[redacted]	-0.308631	[redacted]
chr8	111408610	111408676	[redacted]	-0.30863	[redacted]
chr2	28770667	28770694	[redacted]	-0.308623	[redacted]
chr13	91657797	91657860	[redacted]	-0.308622	[redacted]
chr4	22395075	22395108	[redacted]	-0.308614	[redacted]
chr7	18953226	18953273	[redacted]	-0.308614	[redacted]
chr11	18847969	18848053	[redacted]	-0.308609	[redacted]
chr6	66574646	66574730	[redacted]	-0.308607	[redacted]
chr8	18879971	18879992	[redacted]	-0.30859	[redacted]
chr8	39519194	39519222	[redacted]	-0.308554	[redacted]
chr18	78927676	78927741	[redacted]	-0.308549	[redacted]
chr6	113504583	113504628	[redacted]	-0.308549	[redacted]
chr14	57753465	57753511	[redacted]	-0.308548	[redacted]
chr16	76302167	76302242	[redacted]	-0.308537	[redacted]
chr3	103355868	103355908	[redacted]	-0.308515	[redacted]
chr16	72481038	72481091	[redacted]	-0.308511	[redacted]
chr11	111667993	111668029	[redacted]	-0.308506	[redacted]
chr4	111190617	111190668	[redacted]	-0.308499	[redacted]
chr18	52803327	52803409	[redacted]	-0.308432	[redacted]
chr10	58619990	58620009	[redacted]	-0.308416	[redacted]
chr5	64420288	64420344	[redacted]	-0.308411	[redacted]
chr4	67002774	67002871	[redacted]	-0.308404	[redacted]
chr2	53019217	53019281	[redacted]	-0.308369	[redacted]
chr13	61832222	61832313	[redacted]	-0.308359	[redacted]
chr16	74500826	74500895	[redacted]	-0.308347	[redacted]
chr14	118859181	118859223	[redacted]	-0.308337	[redacted]
chr9	120532231	120532273	[redacted]	-0.308326	[redacted]

chr3	37979233	37979330	[redacted]	-0.308321	[redacted]
chr12	84757050	84757116	[redacted]	-0.308315	[redacted]
chr12	8247732	8247761	[redacted]	-0.308276	[redacted]
chrX	86040688	86040759	[redacted]	-0.308276	[redacted]
chr6	27176310	27176396	[redacted]	-0.308275	[redacted]
chr15	101785516	101785582	[redacted]	-0.308274	[redacted]
chr19	30468926	30468959	[redacted]	-0.308273	[redacted]
chr10	44580460	44580507	[redacted]	-0.308268	[redacted]
chr13	71036437	71036481	[redacted]	-0.308259	[redacted]
chr3	107350446	107350513	[redacted]	-0.308255	[redacted]
chr10	85689880	85689929	[redacted]	-0.308254	[redacted]
chr11	64308917	64308966	[redacted]	-0.308251	[redacted]
chr5	26244456	26244522	[redacted]	-0.308249	[redacted]
chr18	7401416	7401443	[redacted]	-0.308238	[redacted]
chr8	111509468	111509503	[redacted]	-0.308227	[redacted]
chr10	100500159	100500227	[redacted]	-0.308222	[redacted]
chr3	154192427	154192476	[redacted]	-0.30822	[redacted]
chr8	76519510	76519553	[redacted]	-0.308169	[redacted]
chr15	65094145	65094203	[redacted]	-0.308166	[redacted]
chr9	116228362	116228427	[redacted]	-0.308138	[redacted]
chr9	42714675	42714702	[redacted]	-0.30813	[redacted]
chr1	161316824	161316850	[redacted]	-0.308128	[redacted]
chr14	65490001	65490051	[redacted]	-0.308118	[redacted]
chr7	67531627	67531651	[redacted]	-0.308118	[redacted]
chr11	57101067	57101155	[redacted]	-0.308113	[redacted]
chr9	22506446	22506561	[redacted]	-0.308107	[redacted]
chr8	33916737	33916770	[redacted]	-0.308082	[redacted]
chr6	51625447	51625514	[redacted]	-0.308069	[redacted]
chr1	171932962	171932980	[redacted]	-0.308062	[redacted]
chr5	92185903	92185978	[redacted]	-0.308025	[redacted]
chr1	193729966	193730034	[redacted]	-0.308018	[redacted]
chrX	82273260	82273296	[redacted]	-0.308012	[redacted]
chr15	30528807	30528880	[redacted]	-0.30801	[redacted]
chr15	52981335	52981412	[redacted]	-0.307999	[redacted]
chr9	119567479	119567490	[redacted]	-0.307995	[redacted]
chr10	62378019	62378039	[redacted]	-0.307979	[redacted]
chr19	16587925	16588096	[redacted]	-0.307941	[redacted]
chr10	37358370	37358435	[redacted]	-0.307934	[redacted]
chr10	75331660	75331711	[redacted]	-0.307927	[redacted]
chr18	61860568	61860600	[redacted]	-0.307925	[redacted]
chr1	136396126	136396216	[redacted]	-0.30792	[redacted]
chr5	53717200	53717256	[redacted]	-0.307917	[redacted]
chr13	116912491	116912540	[redacted]	-0.307908	[redacted]

chr17	30008031	30008092	[redacted]	-0.307903	[redacted]
chr8	45086397	45086434	[redacted]	-0.307903	[redacted]
chr5	147511446	147511465	[redacted]	-0.307896	[redacted]
chr15	32048367	32048386	[redacted]	-0.307893	[redacted]
chr10	63026542	63026610	[redacted]	-0.307878	[redacted]
chr13	4563556	4563663	[redacted]	-0.307869	[redacted]
chr9	112414739	112414814	[redacted]	-0.307861	[redacted]
chr5	100148307	100148404	[redacted]	-0.307855	[redacted]
chr11	59929197	59929256	[redacted]	-0.307854	[redacted]
chr2	99832499	99832550	[redacted]	-0.30785	[redacted]
chr3	116273579	116273657	[redacted]	-0.307847	[redacted]
chr18	79459154	79459240	[redacted]	-0.30783	[redacted]
chr8	27579877	27579942	[redacted]	-0.307826	[redacted]
chr8	80437824	80437880	[redacted]	-0.307826	[redacted]
chr2	113297336	113297412	[redacted]	-0.30782	[redacted]
chr15	89545474	89545529	[redacted]	-0.307813	[redacted]
chr18	47148629	47148681	[redacted]	-0.307794	[redacted]
chr18	22637042	22637115	[redacted]	-0.307787	[redacted]
chr3	107453553	107453623	[redacted]	-0.307787	[redacted]
chr6	3504676	3504718	[redacted]	-0.307774	[redacted]
chr3	100703836	100703930	[redacted]	-0.307759	[redacted]
chr2	31681619	31681668	[redacted]	-0.307756	[redacted]
chr14	20798022	20798102	[redacted]	-0.307741	[redacted]
chr18	17656332	17656393	[redacted]	-0.307738	[redacted]
chr10	8283204	8283272	[redacted]	-0.307726	[redacted]
chr16	33261097	33261175	[redacted]	-0.307692	[redacted]
chr6	42394549	42394597	[redacted]	-0.307685	[redacted]
chr9	101718862	101718887	[redacted]	-0.307676	[redacted]
chrX	3858775	3858838	[redacted]	-0.307673	[redacted]
chr5	146401725	146401766	[redacted]	-0.307671	[redacted]
chr4	56352256	56352295	[redacted]	-0.307644	[redacted]
chr2	90437316	90437367	[redacted]	-0.307642	[redacted]
chr4	80319230	80319280	[redacted]	-0.307638	[redacted]
chr2	168815953	168816059	[redacted]	-0.307623	[redacted]
chr1	34085118	34085195	[redacted]	-0.307622	[redacted]
chrX	139824000	139824070	[redacted]	-0.307602	[redacted]
chr2	169989859	169989964	[redacted]	-0.307577	[redacted]
chr3	110021073	110021125	[redacted]	-0.307567	[redacted]
chr9	20548378	20548451	[redacted]	-0.307563	[redacted]
chr4	87375955	87376045	[redacted]	-0.307559	[redacted]
chr10	61455213	61455296	[redacted]	-0.307549	[redacted]
chr1	132162832	132162920	[redacted]	-0.307549	[redacted]
chr2	145715445	145715511	[redacted]	-0.307538	[redacted]

chr14	75552325	75552426	[redacted]	-0.307528	[redacted]
chr1	121679847	121679870	[redacted]	-0.307526	[redacted]
chrX	101132633	101132751	[redacted]	-0.307512	[redacted]
chr9	41803753	41803807	[redacted]	-0.307502	[redacted]
chr5	75737898	75738001	[redacted]	-0.30749	[redacted]
chr11	94651164	94651214	[redacted]	-0.307486	[redacted]
chr4	83869364	83869414	[redacted]	-0.307485	[redacted]
chr15	27904266	27904301	[redacted]	-0.307455	[redacted]
chr15	52742087	52742233	[redacted]	-0.30745	[redacted]
chr12	14619647	14619705	[redacted]	-0.307441	[redacted]
chr11	96122491	96122507	[redacted]	-0.307438	[redacted]
chr4	126742128	126742155	[redacted]	-0.30742	[redacted]
chr4	148620623	148620704	[redacted]	-0.30741	[redacted]
chr10	117717104	117717188	[redacted]	-0.307397	[redacted]
chr6	115921129	115921196	[redacted]	-0.307389	[redacted]
chr10	4125330	4125390	[redacted]	-0.307385	[redacted]
chr3	11988681	11988747	[redacted]	-0.307367	[redacted]
chr11	117215862	117215911	[redacted]	-0.307356	[redacted]
chr17	5915993	5916033	[redacted]	-0.307342	[redacted]
chr15	28238436	28238498	[redacted]	-0.307337	[redacted]
chr5	27006257	27006316	[redacted]	-0.307337	[redacted]
chr4	32499322	32499377	[redacted]	-0.307336	[redacted]
chrX	166518283	166518372	[redacted]	-0.307304	[redacted]
chr1	97659280	97659328	[redacted]	-0.307303	[redacted]
chr12	53100027	53100102	[redacted]	-0.307298	[redacted]
chr19	20550531	20550595	[redacted]	-0.307294	[redacted]
chr6	71540334	71540371	[redacted]	-0.307291	[redacted]
chrX	98214533	98214602	[redacted]	-0.307275	[redacted]
chr3	90210343	90210392	[redacted]	-0.307272	[redacted]
chr14	14023640	14024056	[redacted]	-0.307271	[redacted]
chr4	108395301	108395330	[redacted]	-0.307269	[redacted]
chr1	39610977	39611038	[redacted]	-0.307265	[redacted]
chr4	95933804	95933857	[redacted]	-0.307258	[redacted]
chr3	60302368	60302482	[redacted]	-0.307257	[redacted]
chr9	35541951	35542007	[redacted]	-0.307243	[redacted]
chr19	32503799	32503824	[redacted]	-0.307237	[redacted]
chr4	88541427	88541496	[redacted]	-0.307235	[redacted]
chr6	114910402	114910435	[redacted]	-0.307233	[redacted]
chr6	7316570	7316608	[redacted]	-0.307232	[redacted]
chr5	143654093	143654160	[redacted]	-0.307226	[redacted]
chr17	49745641	49745713	[redacted]	-0.307222	[redacted]
chr9	59592617	59592661	[redacted]	-0.307221	[redacted]
chr1	83701276	83701351	[redacted]	-0.307219	[redacted]

chr10	5400618	5400671	[redacted]	-0.307216	[redacted]
chr5	150461562	150461594	[redacted]	-0.307213	[redacted]
chr9	100511725	100511817	[redacted]	-0.307211	[redacted]
chr18	90508528	90508661	[redacted]	-0.307204	[redacted]
chr8	80805713	80805792	[redacted]	-0.307198	[redacted]
chr16	96981916	96981975	[redacted]	-0.30719	[redacted]
chr14	77247659	77247721	[redacted]	-0.307183	[redacted]
chr4	4092665	4092723	[redacted]	-0.30718	[redacted]
chr15	61858371	61858428	[redacted]	-0.307157	[redacted]
chr5	132107842	132107917	[redacted]	-0.307156	[redacted]
chr17	46893696	46893763	[redacted]	-0.307155	[redacted]
chr16	31626322	31626336	[redacted]	-0.307154	[redacted]
chr13	77476522	77476612	[redacted]	-0.307143	[redacted]
chr11	94592319	94592473	[redacted]	-0.307137	[redacted]
chr17	72816278	72816356	[redacted]	-0.307099	[redacted]
chr3	63930859	63930983	[redacted]	-0.30707	[redacted]
chr15	72973368	72973420	[redacted]	-0.307058	[redacted]
chr4	107513469	107513554	[redacted]	-0.307055	[redacted]
chr18	32985234	32985271	[redacted]	-0.307048	[redacted]
chr1	46909482	46909513	[redacted]	-0.307043	[redacted]
chr2	6153408	6153473	[redacted]	-0.307042	[redacted]
chr3	57600345	57600361	[redacted]	-0.307032	[redacted]
chr6	21856551	21856599	[redacted]	-0.307031	[redacted]
chr9	118901408	118901493	[redacted]	-0.307025	[redacted]
chr15	7268743	7268829	[redacted]	-0.306992	[redacted]
chr11	114326190	114326253	[redacted]	-0.306984	[redacted]
chr1	157980880	157980915	[redacted]	-0.306981	[redacted]
chr4	11288378	11288383	[redacted]	-0.306976	[redacted]
chr8	47339393	47339436	[redacted]	-0.306962	[redacted]
chr13	89772684	89772701	[redacted]	-0.306954	[redacted]
chr15	35938734	35938841	[redacted]	-0.306951	[redacted]
chr11	98812887	98812936	[redacted]	-0.306951	[redacted]
chr11	100863863	100863905	[redacted]	-0.306942	[redacted]
chr15	5377019	5377122	[redacted]	-0.30694	[redacted]
chr1	134314942	134314992	[redacted]	-0.306937	[redacted]
chr14	8694621	8694670	[redacted]	-0.306918	[redacted]
chr16	21736916	21736956	[redacted]	-0.306906	[redacted]
chr3	100596433	100596550	[redacted]	-0.306903	[redacted]
chr5	123252327	123252366	[redacted]	-0.306898	[redacted]
chr17	86977135	86977157	[redacted]	-0.306895	[redacted]
chr17	47893140	47893200	[redacted]	-0.306881	[redacted]
chr4	150788694	150788756	[redacted]	-0.306876	[redacted]
chr16	90744788	90744846	[redacted]	-0.306859	[redacted]

chr9	56518188	56518253	[redacted]	-0.306851	[redacted]
chr2	20013124	20013197	[redacted]	-0.306847	[redacted]
chr9	108959018	108959077	[redacted]	-0.306841	[redacted]
chr17	46670516	46670589	[redacted]	-0.306833	[redacted]
chr17	5139305	5139386	[redacted]	-0.30682	[redacted]
chr5	52907822	52907865	[redacted]	-0.306814	[redacted]
chr10	60675186	60675268	[redacted]	-0.306778	[redacted]
chr17	11556675	11556733	[redacted]	-0.306772	[redacted]
chr13	113480447	113480493	[redacted]	-0.30677	[redacted]
chr13	34806006	34806090	[redacted]	-0.306766	[redacted]
chr3	39892916	39892980	[redacted]	-0.306743	[redacted]
chr2	166258362	166258426	[redacted]	-0.306742	[redacted]
chr7	10119597	10119680	[redacted]	-0.306732	[redacted]
chr5	150124974	150125049	[redacted]	-0.306719	[redacted]
chr9	114066157	114066248	[redacted]	-0.306711	[redacted]
chr1	23756924	23756980	[redacted]	-0.306707	[redacted]
chr7	41867650	41867697	[redacted]	-0.306706	[redacted]
chr17	87385445	87385534	[redacted]	-0.306704	[redacted]
chr13	25186236	25186299	[redacted]	-0.30666	[redacted]
chr6	95084079	95084130	[redacted]	-0.30665	[redacted]
chr1	137523152	137523175	[redacted]	-0.306648	[redacted]
chr11	90179041	90179126	[redacted]	-0.306646	[redacted]
chr1	77265953	77266015	[redacted]	-0.306643	[redacted]
chr9	69115289	69115371	[redacted]	-0.306631	[redacted]
chr11	4409938	4410006	[redacted]	-0.306612	[redacted]
chr11	78422348	78422416	[redacted]	-0.3066	[redacted]
chr14	60754201	60754249	[redacted]	-0.306599	[redacted]
chr1	46072450	46072507	[redacted]	-0.306598	[redacted]
chr4	116407392	116407465	[redacted]	-0.306591	[redacted]
chr6	61099899	61099960	[redacted]	-0.306586	[redacted]
chr14	124177138	124177208	[redacted]	-0.306584	[redacted]
chr3	111702132	111702198	[redacted]	-0.30658	[redacted]
chrX	12919278	12919329	[redacted]	-0.306579	[redacted]
chr7	3946871	3946939	[redacted]	-0.306567	[redacted]
chr9	31586317	31586359	[redacted]	-0.306554	[redacted]
chr4	51055081	51055154	[redacted]	-0.306541	[redacted]
chr15	51600327	51600398	[redacted]	-0.306535	[redacted]
chr17	17614593	17614666	[redacted]	-0.306526	[redacted]
chr15	33397749	33397888	[redacted]	-0.306523	[redacted]
chr9	118155050	118155109	[redacted]	-0.30652	[redacted]
chr18	89424319	89424394	[redacted]	-0.306509	[redacted]
chr1	92679339	92679371	[redacted]	-0.306507	[redacted]
chr5	25758390	25758435	[redacted]	-0.306494	[redacted]

chr1	83712843	83712885	[redacted]	-0.30648	[redacted]
chr9	13702290	13702354	[redacted]	-0.306464	[redacted]
chr16	90694016	90694080	[redacted]	-0.306462	[redacted]
chr2	104749939	104750002	[redacted]	-0.306455	[redacted]
chr1	40314037	40314146	[redacted]	-0.306454	[redacted]
chr17	43032661	43032713	[redacted]	-0.306444	[redacted]
chr11	79821140	79821221	[redacted]	-0.306442	[redacted]
chr1	23330173	23330220	[redacted]	-0.30644	[redacted]
chr9	45193105	45193201	[redacted]	-0.306412	[redacted]
chr17	35261777	35261817	[redacted]	-0.306409	[redacted]
chr11	10268247	10268306	[redacted]	-0.306397	[redacted]
chr7	126726578	126726634	[redacted]	-0.306381	[redacted]
chr2	65070528	65070584	[redacted]	-0.306357	[redacted]
chr5	110640513	110640567	[redacted]	-0.306356	[redacted]
chr3	81921131	81921193	[redacted]	-0.306351	[redacted]
chr6	16805271	16805330	[redacted]	-0.306333	[redacted]
chr6	134587678	134587745	[redacted]	-0.30633	[redacted]
chr1	191419495	191419534	[redacted]	-0.30631	[redacted]
chr5	142898347	142898376	[redacted]	-0.306305	[redacted]
chr10	40649711	40649735	[redacted]	-0.306299	[redacted]
chr8	115408639	115408698	[redacted]	-0.306298	[redacted]
chr12	52329195	52329252	[redacted]	-0.306296	[redacted]
chr15	100285413	100285485	[redacted]	-0.306295	[redacted]
chr7	75689533	75689585	[redacted]	-0.306293	[redacted]
chr3	59252197	59252317	[redacted]	-0.306292	[redacted]
chr6	95732564	95732655	[redacted]	-0.306275	[redacted]
chr1	62822005	62822050	[redacted]	-0.306246	[redacted]
chr6	50028966	50029000	[redacted]	-0.306237	[redacted]
chr1	143235465	143235538	[redacted]	-0.306237	[redacted]
chr6	31035656	31035697	[redacted]	-0.306236	[redacted]
chr14	14254605	14254680	[redacted]	-0.306221	[redacted]
chr9	113543218	113543278	[redacted]	-0.306215	[redacted]
chr5	73472783	73472839	[redacted]	-0.306214	[redacted]
chr2	77045295	77045362	[redacted]	-0.306213	[redacted]
chr19	31951067	31951116	[redacted]	-0.30621	[redacted]
chr6	111630383	111630463	[redacted]	-0.306193	[redacted]
chr10	13106831	13106925	[redacted]	-0.306185	[redacted]
chr9	7540393	7540437	[redacted]	-0.306184	[redacted]
chr3	98215770	98215843	[redacted]	-0.306183	[redacted]
chr8	57754546	57754597	[redacted]	-0.306181	[redacted]
chr11	24487213	24487269	[redacted]	-0.30618	[redacted]
chr4	89707865	89707919	[redacted]	-0.306178	[redacted]
chr6	34629455	34629524	[redacted]	-0.306174	[redacted]

chr11	120837730	120837788	[redacted]	-0.306157	[redacted]
chr5	135667273	135667337	[redacted]	-0.306145	[redacted]
chr4	106891170	106891237	[redacted]	-0.306096	[redacted]
chr1	60857481	60857518	[redacted]	-0.306096	[redacted]
chrX	48066883	48066934	[redacted]	-0.306095	[redacted]
chr6	119442784	119442847	[redacted]	-0.306091	[redacted]
chr8	26022894	26022919	[redacted]	-0.30609	[redacted]
chr12	72967954	72968014	[redacted]	-0.306071	[redacted]
chr17	87120799	87120851	[redacted]	-0.306058	[redacted]
chr10	59224067	59224115	[redacted]	-0.306058	[redacted]
chr16	76329806	76329843	[redacted]	-0.306053	[redacted]
chr5	55670365	55670424	[redacted]	-0.306053	[redacted]
chr14	120077364	120077433	[redacted]	-0.306051	[redacted]
chr17	5605031	5605087	[redacted]	-0.306049	[redacted]
chr5	54197215	54197284	[redacted]	-0.30604	[redacted]
chr8	113693006	113693047	[redacted]	-0.306039	[redacted]
chr18	18479358	18479440	[redacted]	-0.306021	[redacted]
chrX	106767452	106767520	[redacted]	-0.306012	[redacted]
chr7	4261123	4261174	[redacted]	-0.306001	[redacted]
chr8	9560087	9560139	[redacted]	-0.305997	[redacted]
chr2	166355110	166355178	[redacted]	-0.30599	[redacted]
chr19	47715101	47715172	[redacted]	-0.305984	[redacted]
chr6	32363851	32363914	[redacted]	-0.305955	[redacted]
chr16	56162965	56163004	[redacted]	-0.305951	[redacted]
chr10	91098370	91098427	[redacted]	-0.305946	[redacted]
chr9	78501975	78502030	[redacted]	-0.305928	[redacted]
chr4	58426153	58426207	[redacted]	-0.305921	[redacted]
chr5	34229105	34229157	[redacted]	-0.305919	[redacted]
chr3	30079593	30079689	[redacted]	-0.305919	[redacted]
chr13	68903619	68903663	[redacted]	-0.305916	[redacted]
chr15	33443435	33443512	[redacted]	-0.305914	[redacted]
chr8	74796895	74796972	[redacted]	-0.30591	[redacted]
chr6	118540175	118540235	[redacted]	-0.305863	[redacted]
chr13	81756626	81756639	[redacted]	-0.305851	[redacted]
chr10	128982722	128982797	[redacted]	-0.305846	[redacted]
chr8	36065014	36065121	[redacted]	-0.305844	[redacted]
chr13	55647785	55647792	[redacted]	-0.30584	[redacted]
chr19	57875761	57875815	[redacted]	-0.30583	[redacted]
chr3	80657708	80657793	[redacted]	-0.305813	[redacted]
chr12	81757882	81757935	[redacted]	-0.305781	[redacted]
chr10	30310707	30310786	[redacted]	-0.305759	[redacted]
chr11	23634400	23634488	[redacted]	-0.305755	[redacted]
chr4	6943873	6943954	[redacted]	-0.30574	[redacted]

chr7	16143716	16143776		-0.30573	
chr16	24981399	24981459		-0.305728	
chr6	137817188	137817237		-0.305727	
chr5	53618491	53618580		-0.305726	
chr9	49482621	49482660		-0.305726	
chr7	80624602	80624679		-0.30572	
chr11	10511722	10511789		-0.305713	
chr3	78826848	78826889		-0.305695	
chr5	100421031	100421073		-0.305689	
chr11	120054021	120054085		-0.305684	
chr13	83017604	83017671		-0.305672	
chr6	88982217	88982287		-0.305654	
chr10	111497654	111497736		-0.305652	
chr11	69584094	69584136		-0.305642	
chr8	110733293	110733342		-0.305641	
chr19	25395595	25395665		-0.305621	
chr10	77834191	77834220		-0.305612	
chr1	184959563	184959652		-0.305607	
chr13	86216843	86216871		-0.305605	
chr11	99473952	99474034		-0.305605	
chr14	99449539	99449557		-0.305573	
chr2	93146348	93146433		-0.305567	
chr10	78333291	78333366		-0.305563	
chr19	20940760	20940817		-0.305562	
chr17	79916133	79916163		-0.305561	
chr7	24463677	24463702		-0.30556	
chr14	73431828	73431862		-0.305544	
chr14	84668896	84668993		-0.305536	
chr10	83830643	83830677		-0.305528	
chr4	90603387	90603446		-0.305512	
chr1	78603711	78603814		-0.305511	
chr1	152131460	152131515		-0.305509	
chr11	78941004	78941094		-0.305503	
chrX	112434497	112434546		-0.305499	
chr18	66573616	66573665		-0.305495	
chr6	145450674	145450780		-0.305489	
chr19	48471815	48471866		-0.305487	
chr9	55843426	55843497		-0.305487	
chr9	65575092	65575202		-0.305466	
chr5	33312531	33312565		-0.305461	
chr13	32210526	32210653		-0.305458	
chr6	135260653	135260724		-0.305458	
chr8	33940601	33940652		-0.305453	

chr3	66418792	66418850	[redacted]	-0.305449	[redacted]
chr9	30129892	30129955	[redacted]	-0.305439	[redacted]
chr4	129906788	129906810	[redacted]	-0.305431	[redacted]
chr2	126708175	126708224	[redacted]	-0.305427	[redacted]
chr5	122754512	122754557	[redacted]	-0.305424	[redacted]
chr12	81275031	81275083	[redacted]	-0.305418	[redacted]
chr12	81512317	81512330	[redacted]	-0.305408	[redacted]
chr1	51895127	51895186	[redacted]	-0.305384	[redacted]
chr9	41767645	41767731	[redacted]	-0.305383	[redacted]
chr1	68803408	68803493	[redacted]	-0.30537	[redacted]
chr11	84594197	84594243	[redacted]	-0.30537	[redacted]
chr14	15121849	15121903	[redacted]	-0.305361	[redacted]
chr4	58932699	58932828	[redacted]	-0.305361	[redacted]
chr10	118615380	118615435	[redacted]	-0.30536	[redacted]
chr17	15709243	15709278	[redacted]	-0.305353	[redacted]
chr3	126075272	126075323	[redacted]	-0.305353	[redacted]
chr13	117199147	117199214	[redacted]	-0.30535	[redacted]
chr9	61629396	61629474	[redacted]	-0.305336	[redacted]
chr1	7300858	7300906	[redacted]	-0.305336	[redacted]
chr12	82522780	82522819	[redacted]	-0.305323	[redacted]
chr2	111353482	111353573	[redacted]	-0.305307	[redacted]
chr5	132607988	132608027	[redacted]	-0.305295	[redacted]
chr2	84713543	84713558	[redacted]	-0.305294	[redacted]
chr9	64341932	64341967	[redacted]	-0.305293	[redacted]
chr2	156159066	156159158	[redacted]	-0.305289	[redacted]
chr17	62304799	62304857	[redacted]	-0.305286	[redacted]
chr10	43059594	43059607	[redacted]	-0.30528	[redacted]
chr8	81397364	81397423	[redacted]	-0.305272	[redacted]
chr3	139392620	139392711	[redacted]	-0.305258	[redacted]
chrX	20868245	20868314	[redacted]	-0.305248	[redacted]
chr3	8998345	8998382	[redacted]	-0.305241	[redacted]
chr5	77180697	77180766	[redacted]	-0.305237	[redacted]
chr12	56102191	56102261	[redacted]	-0.305235	[redacted]
chr6	41571052	41571106	[redacted]	-0.305235	[redacted]
chr4	149471526	149471596	[redacted]	-0.305231	[redacted]
chr5	105750302	105750392	[redacted]	-0.305219	[redacted]
chr18	9744695	9744768	[redacted]	-0.305216	[redacted]
chr13	38371337	38371448	[redacted]	-0.305208	[redacted]
chr6	145457349	145457378	[redacted]	-0.305208	[redacted]
chr16	90540129	90540168	[redacted]	-0.305204	[redacted]
chr11	98749342	98749400	[redacted]	-0.305196	[redacted]
chr10	41616455	41616555	[redacted]	-0.305186	[redacted]
chr13	113800765	113800831	[redacted]	-0.305154	[redacted]

chr3	152203542	152203592	[redacted]	-0.305145	[redacted]
chr9	82967445	82967496	[redacted]	-0.30513	[redacted]
chr8	33408362	33408386	[redacted]	-0.305123	[redacted]
chr10	65403385	65403500	[redacted]	-0.305102	[redacted]
chr4	45778599	45778691	[redacted]	-0.305098	[redacted]
chr3	119733184	119733308	[redacted]	-0.305095	[redacted]
chr12	82992977	82993057	[redacted]	-0.305079	[redacted]
chr6	106722116	106722202	[redacted]	-0.305071	[redacted]
chr1	195058841	195058918	[redacted]	-0.30507	[redacted]
chr10	78360809	78360854	[redacted]	-0.30507	[redacted]
chr5	113847031	113847050	[redacted]	-0.305053	[redacted]
chr3	128910346	128910396	[redacted]	-0.305046	[redacted]
chr4	9183847	9183937	[redacted]	-0.305038	[redacted]
chr15	93481430	93481492	[redacted]	-0.305037	[redacted]
chr15	68241639	68241695	[redacted]	-0.305033	[redacted]
chr1	55446780	55446848	[redacted]	-0.305033	[redacted]
chr11	105489280	105489371	[redacted]	-0.305021	[redacted]
chr14	54642073	54642103	[redacted]	-0.30502	[redacted]
chr5	92069005	92069065	[redacted]	-0.305001	[redacted]
chr3	90068352	90068404	[redacted]	-0.304997	[redacted]
chr6	113609017	113609079	[redacted]	-0.304994	[redacted]
chr1	131235122	131235208	[redacted]	-0.304992	[redacted]
chr5	90162703	90162780	[redacted]	-0.30499	[redacted]
chr19	27468369	27468434	[redacted]	-0.304969	[redacted]
chr1	36986962	36987004	[redacted]	-0.304965	[redacted]
chr9	49709755	49709806	[redacted]	-0.304957	[redacted]
chr4	61821995	61822026	[redacted]	-0.304949	[redacted]
chr4	149867932	149867995	[redacted]	-0.304946	[redacted]
chr7	111075196	111075236	[redacted]	-0.304941	[redacted]
chr14	22210304	22210319	[redacted]	-0.304939	[redacted]
chr9	114518537	114518590	[redacted]	-0.304938	[redacted]
chr6	77842470	77842503	[redacted]	-0.304933	[redacted]
chr4	43246037	43246058	[redacted]	-0.304928	[redacted]
chr15	96708628	96708688	[redacted]	-0.304927	[redacted]
chr8	117725281	117725342	[redacted]	-0.304927	[redacted]
chr9	42881396	42881506	[redacted]	-0.304923	[redacted]
chr10	111496001	111496058	[redacted]	-0.30492	[redacted]
chr15	9835909	9835925	[redacted]	-0.30491	[redacted]
chr3	130983155	130983175	[redacted]	-0.304907	[redacted]
chr11	59609925	59609997	[redacted]	-0.304893	[redacted]
chr6	70803452	70803495	[redacted]	-0.304885	[redacted]
chr2	169855710	169855766	[redacted]	-0.304876	[redacted]
chr6	28296266	28296341	[redacted]	-0.304864	[redacted]

chr6	49833239	49833289	[redacted]	-0.304862	[redacted]
chr8	68325626	68325673	[redacted]	-0.304851	[redacted]
chr1	56494973	56494988	[redacted]	-0.304846	[redacted]
chr2	13510902	13510946	[redacted]	-0.304846	[redacted]
chrX	102823497	102823559	[redacted]	-0.304845	[redacted]
chr18	46529274	46529333	[redacted]	-0.304844	[redacted]
chr14	109432094	109432161	[redacted]	-0.304841	[redacted]
chr19	5932814	5932855	[redacted]	-0.304828	[redacted]
chr12	8756018	8756059	[redacted]	-0.304826	[redacted]
chr8	16706223	16706273	[redacted]	-0.304824	[redacted]
chr1	130962364	130962411	[redacted]	-0.304821	[redacted]
chr1	86091167	86091199	[redacted]	-0.30482	[redacted]
chr19	14365724	14365773	[redacted]	-0.304806	[redacted]
chr12	50403987	50404079	[redacted]	-0.304799	[redacted]
chr4	100540834	100540945	[redacted]	-0.304765	[redacted]
chr7	58484888	58484954	[redacted]	-0.304762	[redacted]
chr2	17852484	17852531	[redacted]	-0.304761	[redacted]
chr9	116929280	116929322	[redacted]	-0.304753	[redacted]
chr5	24836273	24836311	[redacted]	-0.304749	[redacted]
chr3	143376079	143376139	[redacted]	-0.304734	[redacted]
chr16	55668927	55669027	[redacted]	-0.304719	[redacted]
chr3	102311544	102311573	[redacted]	-0.304709	[redacted]
chr2	17969282	17969334	[redacted]	-0.304695	[redacted]
chr2	59292677	59292728	[redacted]	-0.304693	[redacted]
chr11	75861223	75861288	[redacted]	-0.304676	[redacted]
chr1	145936089	145936156	[redacted]	-0.304675	[redacted]
chr13	30149258	30149310	[redacted]	-0.304672	[redacted]
chr15	5583556	5583642	[redacted]	-0.304672	[redacted]
chr10	116310699	116310749	[redacted]	-0.304663	[redacted]
chr3	119518800	119518837	[redacted]	-0.304663	[redacted]
chr10	126906384	126906477	[redacted]	-0.304659	[redacted]
chr9	59085762	59085804	[redacted]	-0.304652	[redacted]
chr15	69308232	69308288	[redacted]	-0.304641	[redacted]
chr1	181884219	181884325	[redacted]	-0.30464	[redacted]
chrX	79631413	79631475	[redacted]	-0.304633	[redacted]
chr3	148984797	148984845	[redacted]	-0.304626	[redacted]
chr11	96444354	96444377	[redacted]	-0.304626	[redacted]
chr1	96115276	96115328	[redacted]	-0.304617	[redacted]
chr6	145692357	145692403	[redacted]	-0.304611	[redacted]
chr15	96964175	96964225	[redacted]	-0.304594	[redacted]
chr5	151127226	151127274	[redacted]	-0.304592	[redacted]
chr19	31674958	31675040	[redacted]	-0.304569	[redacted]
chr8	66483689	66483747	[redacted]	-0.304565	[redacted]

chr10	8556452	8556471	[redacted]	-0.304563	[redacted]
chr13	17008724	17008780	[redacted]	-0.304563	[redacted]
chr1	16939802	16939839	[redacted]	-0.304559	[redacted]
chr5	141890850	141890898	[redacted]	-0.304548	[redacted]
chr5	34903554	34903602	[redacted]	-0.304545	[redacted]
chr14	29945886	29945928	[redacted]	-0.304531	[redacted]
chr11	22070507	22070591	[redacted]	-0.304519	[redacted]
chr18	55343821	55343910	[redacted]	-0.304519	[redacted]
chr3	96415589	96415633	[redacted]	-0.304508	[redacted]
chr6	31297738	31297893	[redacted]	-0.304488	[redacted]
chr13	107808888	107808937	[redacted]	-0.304486	[redacted]
chr11	54407935	54407952	[redacted]	-0.304472	[redacted]
chr8	20511928	20511965	[redacted]	-0.304467	[redacted]
chr10	117819581	117819624	[redacted]	-0.304466	[redacted]
chr1	132473628	132473667	[redacted]	-0.304457	[redacted]
chr19	55507419	55507514	[redacted]	-0.304452	[redacted]
chr3	16421459	16421544	[redacted]	-0.304443	[redacted]
chr1	96867890	96867975	[redacted]	-0.304421	[redacted]
chr15	72744237	72744319	[redacted]	-0.304417	[redacted]
chr14	16488604	16488650	[redacted]	-0.304386	[redacted]
chr11	29833160	29833176	[redacted]	-0.304372	[redacted]
chr13	78381965	78382029	[redacted]	-0.304362	[redacted]
chr14	102739463	102739560	[redacted]	-0.304351	[redacted]
chr3	41152206	41152258	[redacted]	-0.304338	[redacted]
chr16	90697818	90697853	[redacted]	-0.304304	[redacted]
chr7	131700082	131700140	[redacted]	-0.304299	[redacted]
chr6	4995202	4995286	[redacted]	-0.304296	[redacted]
chr11	19429704	19429772	[redacted]	-0.304289	[redacted]
chr8	64106083	64106152	[redacted]	-0.304289	[redacted]
chr9	69147575	69147630	[redacted]	-0.304274	[redacted]
chr9	59300897	59300987	[redacted]	-0.304269	[redacted]
chr2	4982377	4982441	[redacted]	-0.304268	[redacted]
chrY	90804503	90804533	[redacted]	-0.304266	[redacted]
chr5	139700697	139700778	[redacted]	-0.304253	[redacted]
chr15	4891158	4891240	[redacted]	-0.304244	[redacted]
chr19	53811480	53811543	[redacted]	-0.304243	[redacted]
chr11	43160618	43160654	[redacted]	-0.304225	[redacted]
chr1	36931024	36931032	[redacted]	-0.304223	[redacted]
chr2	78996903	78996959	[redacted]	-0.304215	[redacted]
chr9	7995409	7995458	[redacted]	-0.304214	[redacted]
chr19	37379879	37379924	[redacted]	-0.304194	[redacted]
chr13	54754042	54754088	[redacted]	-0.304191	[redacted]
chr11	11665498	11665553	[redacted]	-0.304174	[redacted]

chr7	47030932	47030981	[redacted]	-0.304164	[redacted]
chr11	71546707	71546760	[redacted]	-0.304149	[redacted]
chr9	65626124	65626194	[redacted]	-0.304137	[redacted]
chr1	64781622	64781641	[redacted]	-0.304134	[redacted]
chr17	31321814	31321845	[redacted]	-0.304133	[redacted]
chr9	83182213	83182235	[redacted]	-0.30413	[redacted]
chr10	81507612	81507676	[redacted]	-0.304127	[redacted]
chr11	57199229	57199283	[redacted]	-0.304124	[redacted]
chr2	61210315	61210389	[redacted]	-0.304117	[redacted]
chr3	130982793	130982836	[redacted]	-0.304112	[redacted]
chr9	119401075	119401112	[redacted]	-0.30411	[redacted]
chr1	102050045	102050134	[redacted]	-0.304102	[redacted]
chr15	51028231	51028305	[redacted]	-0.3041	[redacted]
chr15	84566523	84566602	[redacted]	-0.30409	[redacted]
chr3	157455757	157455825	[redacted]	-0.304079	[redacted]
chr12	55380217	55380311	[redacted]	-0.304075	[redacted]
chr8	53403624	53403661	[redacted]	-0.304061	[redacted]
chr1	30299963	30300000	[redacted]	-0.304042	[redacted]
chr11	50562834	50562895	[redacted]	-0.30404	[redacted]
chr18	48943335	48943384	[redacted]	-0.304034	[redacted]
chr4	32870735	32870789	[redacted]	-0.304014	[redacted]
chr5	8354367	8354418	[redacted]	-0.304014	[redacted]
chr11	107566814	107566833	[redacted]	-0.304012	[redacted]
chr11	102711203	102711216	[redacted]	-0.304002	[redacted]
chr14	32607126	32607208	[redacted]	-0.303997	[redacted]
chr10	121410508	121410609	[redacted]	-0.30397	[redacted]
chrX	75812200	75812220	[redacted]	-0.303968	[redacted]
chr11	26132843	26132906	[redacted]	-0.303961	[redacted]
chr6	108333992	108334066	[redacted]	-0.303944	[redacted]
chr2	104953845	104953910	[redacted]	-0.303936	[redacted]
chr8	89048705	89048767	[redacted]	-0.30392	[redacted]
chr15	97803121	97803144	[redacted]	-0.303909	[redacted]
chrX	134417646	134417735	[redacted]	-0.303891	[redacted]
chr17	46404585	46404611	[redacted]	-0.303887	[redacted]
chr19	36972929	36973016	[redacted]	-0.303887	[redacted]
chr13	109303414	109303488	[redacted]	-0.30388	[redacted]
chr11	66981815	66981859	[redacted]	-0.303879	[redacted]
chr7	142615451	142615524	[redacted]	-0.303867	[redacted]
chr15	93866857	93866926	[redacted]	-0.303863	[redacted]
chr7	122706133	122706172	[redacted]	-0.303857	[redacted]
chr6	50362112	50362170	[redacted]	-0.303853	[redacted]
chr15	97729479	97729570	[redacted]	-0.303853	[redacted]
chr9	63052797	63052864	[redacted]	-0.303852	[redacted]

chr1	130854827	130854907	[redacted]	-0.303848	[redacted]
chr6	85305158	85305231	[redacted]	-0.303836	[redacted]
chr4	98466460	98466508	[redacted]	-0.303826	[redacted]
chr10	116136259	116136320	[redacted]	-0.303801	[redacted]
chr14	40162598	40162624	[redacted]	-0.303793	[redacted]
chr10	25674475	25674541	[redacted]	-0.303792	[redacted]
chr19	40803829	40803940	[redacted]	-0.303787	[redacted]
chr9	59079878	59079959	[redacted]	-0.30378	[redacted]
chr5	17569720	17569770	[redacted]	-0.30378	[redacted]
chr11	114975703	114975753	[redacted]	-0.303773	[redacted]
chr10	43835121	43835177	[redacted]	-0.303771	[redacted]
chr2	160169593	160169643	[redacted]	-0.303769	[redacted]
chr5	97189934	97190021	[redacted]	-0.303768	[redacted]
chr4	128864063	128864107	[redacted]	-0.303768	[redacted]
chr10	116832604	116832649	[redacted]	-0.303765	[redacted]
chr3	94961718	94961751	[redacted]	-0.303764	[redacted]
chr6	108074599	108074663	[redacted]	-0.303761	[redacted]
chr4	132227081	132227132	[redacted]	-0.303736	[redacted]
chr14	71091798	71091851	[redacted]	-0.303731	[redacted]
chr8	95180593	95180644	[redacted]	-0.303698	[redacted]
chr15	94922804	94922840	[redacted]	-0.303686	[redacted]
chr2	125259694	125259823	[redacted]	-0.303682	[redacted]
chr5	135705430	135705489	[redacted]	-0.303677	[redacted]
chr18	60577502	60577566	[redacted]	-0.303672	[redacted]
chr15	91007097	91007183	[redacted]	-0.303666	[redacted]
chr13	20446004	20446056	[redacted]	-0.30366	[redacted]
chr6	55387759	55387803	[redacted]	-0.303656	[redacted]
chr11	46296591	46296676	[redacted]	-0.30365	[redacted]
chr11	52015028	52015078	[redacted]	-0.303611	[redacted]
chr3	54001455	54001520	[redacted]	-0.303606	[redacted]
chr5	65001104	65001139	[redacted]	-0.303605	[redacted]
chr1	57844850	57844891	[redacted]	-0.303588	[redacted]
chr5	91564488	91564579	[redacted]	-0.303582	[redacted]
chr3	96422362	96422425	[redacted]	-0.303577	[redacted]
chr11	75506744	75506798	[redacted]	-0.303536	[redacted]
chrX	153550289	153550347	[redacted]	-0.303528	[redacted]
chr8	28510673	28510733	[redacted]	-0.303526	[redacted]
chrX	35849918	35849962	[redacted]	-0.303526	[redacted]
chr6	113699582	113699649	[redacted]	-0.303525	[redacted]
chr16	46582435	46582491	[redacted]	-0.303521	[redacted]
chr14	104439745	104439789	[redacted]	-0.303517	[redacted]
chr2	10405272	10405307	[redacted]	-0.303502	[redacted]
chr4	138297473	138297541	[redacted]	-0.303493	[redacted]

chr5	65905823	65905927	[redacted]	-0.303476	[redacted]
chr8	39046563	39046587	[redacted]	-0.303474	[redacted]
chr15	99263147	99263196	[redacted]	-0.303458	[redacted]
chr7	67996094	67996187	[redacted]	-0.303446	[redacted]
chr9	92554690	92554729	[redacted]	-0.303424	[redacted]
chr7	42530822	42530894	[redacted]	-0.303414	[redacted]
chr7	92104353	92104419	[redacted]	-0.303404	[redacted]
chr16	33367698	33367738	[redacted]	-0.303402	[redacted]
chr14	114958232	114958328	[redacted]	-0.303401	[redacted]
chr15	12389087	12389133	[redacted]	-0.3034	[redacted]
chr6	121968195	121968286	[redacted]	-0.303391	[redacted]
chr5	122675004	122675101	[redacted]	-0.303363	[redacted]
chrX	113514014	113514075	[redacted]	-0.303361	[redacted]
chr3	104847229	104847314	[redacted]	-0.303358	[redacted]
chr18	53097117	53097162	[redacted]	-0.303356	[redacted]
chr11	104268065	104268146	[redacted]	-0.303342	[redacted]
chr1	185358157	185358197	[redacted]	-0.303334	[redacted]
chr15	45692521	45692556	[redacted]	-0.303324	[redacted]
chr8	27740598	27740716	[redacted]	-0.303322	[redacted]
chrX	159327781	159327838	[redacted]	-0.303313	[redacted]
chr6	38579729	38579746	[redacted]	-0.303312	[redacted]
chr14	102107561	102107652	[redacted]	-0.303311	[redacted]
chr2	20505805	20505889	[redacted]	-0.303307	[redacted]
chr1	162252093	162252179	[redacted]	-0.30328	[redacted]
chr2	141681277	141681319	[redacted]	-0.303278	[redacted]
chr6	88775088	88775183	[redacted]	-0.303263	[redacted]
chr7	69490704	69490755	[redacted]	-0.303232	[redacted]
chr14	29454694	29454750	[redacted]	-0.303225	[redacted]
chr7	67781577	67781630	[redacted]	-0.303224	[redacted]
chr4	9440937	9441008	[redacted]	-0.30321	[redacted]
chr3	90798837	90798923	[redacted]	-0.303199	[redacted]
chr16	26951118	26951207	[redacted]	-0.303198	[redacted]
chr10	40879712	40879802	[redacted]	-0.303194	[redacted]
chr6	82440920	82440985	[redacted]	-0.303192	[redacted]
chr3	126610500	126610545	[redacted]	-0.303192	[redacted]
chr10	124289156	124289246	[redacted]	-0.303186	[redacted]
chr11	82319018	82319045	[redacted]	-0.303183	[redacted]
chr8	45606475	45606539	[redacted]	-0.303162	[redacted]
chr4	40067928	40068010	[redacted]	-0.303152	[redacted]
chr2	91280955	91281007	[redacted]	-0.30315	[redacted]
chr18	10981126	10981168	[redacted]	-0.303121	[redacted]
chr2	125663907	125663944	[redacted]	-0.303101	[redacted]
chr3	9486451	9486487	[redacted]	-0.303099	[redacted]

chr6	81966835	81966938	[redacted]	-0.303078	[redacted]
chr14	120297540	120297739	[redacted]	-0.303075	[redacted]
chr6	46170174	46170301	[redacted]	-0.303062	[redacted]
chr3	129043259	129043318	[redacted]	-0.303048	[redacted]
chr3	45077616	45077701	[redacted]	-0.303027	[redacted]
chr5	114991132	114991172	[redacted]	-0.303021	[redacted]
chr5	19906007	19906062	[redacted]	-0.303	[redacted]
chr5	63679294	63679374	[redacted]	-0.303	[redacted]
chr2	72600030	72600087	[redacted]	-0.302974	[redacted]
chr1	191376322	191376399	[redacted]	-0.302961	[redacted]
chr8	98291738	98291777	[redacted]	-0.302943	[redacted]
chr5	148328422	148328458	[redacted]	-0.302894	[redacted]
chr7	109072509	109072600	[redacted]	-0.302888	[redacted]
chr8	122420971	122421014	[redacted]	-0.302878	[redacted]
chr4	130854820	130854897	[redacted]	-0.302869	[redacted]
chr18	5475381	5475422	[redacted]	-0.302864	[redacted]
chr15	94948237	94948293	[redacted]	-0.302863	[redacted]
chr1	71929176	71929228	[redacted]	-0.30285	[redacted]
chr14	55116928	55116936	[redacted]	-0.302847	[redacted]
chr16	22483690	22483815	[redacted]	-0.302836	[redacted]
chr12	33577784	33577806	[redacted]	-0.302836	[redacted]
chr2	60664384	60664435	[redacted]	-0.302829	[redacted]
chr6	67707591	67707660	[redacted]	-0.302817	[redacted]
chr5	119356003	119356051	[redacted]	-0.302799	[redacted]
chr14	51025905	51025985	[redacted]	-0.302797	[redacted]
chr9	11422916	11423031	[redacted]	-0.302788	[redacted]
chr12	67230356	67230441	[redacted]	-0.302787	[redacted]
chr7	105361117	105361196	[redacted]	-0.302779	[redacted]
chr1	29435653	29435688	[redacted]	-0.302772	[redacted]
chr3	123137508	123137546	[redacted]	-0.302749	[redacted]
chr12	52359609	52359647	[redacted]	-0.302744	[redacted]
chr11	82646927	82646990	[redacted]	-0.302741	[redacted]
chr17	43647659	43647708	[redacted]	-0.30273	[redacted]
chr1	13822886	13822914	[redacted]	-0.302717	[redacted]
chr8	19503276	19503339	[redacted]	-0.302715	[redacted]
chr13	70498823	70498885	[redacted]	-0.302712	[redacted]
chr12	83385079	83385153	[redacted]	-0.302707	[redacted]
chr9	3664726	3664788	[redacted]	-0.302705	[redacted]
chr3	156502163	156502220	[redacted]	-0.302687	[redacted]
chr7	134358097	134358125	[redacted]	-0.30268	[redacted]
chr14	30473004	30473040	[redacted]	-0.302678	[redacted]
chr10	128633808	128633948	[redacted]	-0.302674	[redacted]
chr4	63488603	63488679	[redacted]	-0.30267	[redacted]

chr11	61396781	61396844	[redacted]	-0.302669	[redacted]
chr18	69122111	69122184	[redacted]	-0.302668	[redacted]
chr1	152457397	152457427	[redacted]	-0.302667	[redacted]
chr2	132114029	132114081	[redacted]	-0.302666	[redacted]
chr14	21904520	21904550	[redacted]	-0.302662	[redacted]
chr1	73075048	73075108	[redacted]	-0.302658	[redacted]
chr3	128122824	128122854	[redacted]	-0.302637	[redacted]
chr8	90545505	90545589	[redacted]	-0.302632	[redacted]
chr13	108917893	108917955	[redacted]	-0.302632	[redacted]
chr1	186651264	186651310	[redacted]	-0.302628	[redacted]
chr14	68200384	68200479	[redacted]	-0.302601	[redacted]
chr17	60893581	60893622	[redacted]	-0.3026	[redacted]
chr8	112460835	112460881	[redacted]	-0.302597	[redacted]
chr15	95466669	95466703	[redacted]	-0.302575	[redacted]
chr4	89012400	89012442	[redacted]	-0.302551	[redacted]
chr16	25135189	25135266	[redacted]	-0.302551	[redacted]
chr7	97752144	97752208	[redacted]	-0.302546	[redacted]
chr7	47036551	47036606	[redacted]	-0.302531	[redacted]
chr3	127468571	127468638	[redacted]	-0.302523	[redacted]
chr16	37498889	37498957	[redacted]	-0.302518	[redacted]
chr16	38398812	38398865	[redacted]	-0.302512	[redacted]
chr14	67718788	67718840	[redacted]	-0.302512	[redacted]
chr11	45996564	45996594	[redacted]	-0.30251	[redacted]
chr11	33716272	33716351	[redacted]	-0.302509	[redacted]
chr8	110596270	110596337	[redacted]	-0.302504	[redacted]
chr19	37410143	37410188	[redacted]	-0.302503	[redacted]
chr4	48580188	48580244	[redacted]	-0.302486	[redacted]
chr2	73491285	73491350	[redacted]	-0.302482	[redacted]
chr15	39406638	39406692	[redacted]	-0.302468	[redacted]
chr9	95851670	95851749	[redacted]	-0.302429	[redacted]
chr3	93493237	93493316	[redacted]	-0.302422	[redacted]
chr1	173435603	173435654	[redacted]	-0.302402	[redacted]
chr7	36520362	36520430	[redacted]	-0.302398	[redacted]
chr15	57469160	57469229	[redacted]	-0.302381	[redacted]
chr1	188461770	188461838	[redacted]	-0.30238	[redacted]
chr9	75456438	75456589	[redacted]	-0.302351	[redacted]
chr8	107793003	107793066	[redacted]	-0.302349	[redacted]
chr7	33862619	33862645	[redacted]	-0.302346	[redacted]
chrX	6403332	6403395	[redacted]	-0.302344	[redacted]
chr13	81737494	81737533	[redacted]	-0.30234	[redacted]
chr17	4756471	4756527	[redacted]	-0.302332	[redacted]
chr13	104262745	104262810	[redacted]	-0.302314	[redacted]
chr17	5847372	5847476	[redacted]	-0.302313	[redacted]

chr1	180126707	180126761	[redacted]	-0.302309	[redacted]
chr5	57800766	57800835	[redacted]	-0.302304	[redacted]
chr2	163979395	163979466	[redacted]	-0.3023	[redacted]
chr10	73994789	73994885	[redacted]	-0.302296	[redacted]
chr16	16804934	16804999	[redacted]	-0.302279	[redacted]
chr17	35816296	35816328	[redacted]	-0.30227	[redacted]
chr13	107672630	107672669	[redacted]	-0.302267	[redacted]
chr13	39095201	39095244	[redacted]	-0.302264	[redacted]
chrX	114465221	114465248	[redacted]	-0.302263	[redacted]
chr18	73548558	73548589	[redacted]	-0.30226	[redacted]
chr7	127652888	127652977	[redacted]	-0.302257	[redacted]
chr12	3743687	3743772	[redacted]	-0.302256	[redacted]
chr18	54500988	54501036	[redacted]	-0.302249	[redacted]
chr2	116036008	116036101	[redacted]	-0.302242	[redacted]
chr15	5882533	5882581	[redacted]	-0.302241	[redacted]
chr19	37673405	37673441	[redacted]	-0.302236	[redacted]
chr2	59801818	59801853	[redacted]	-0.302225	[redacted]
chr3	40845884	40845966	[redacted]	-0.302222	[redacted]
chr4	82808861	82808903	[redacted]	-0.302221	[redacted]
chr2	76105779	76105869	[redacted]	-0.302187	[redacted]
chrX	150704288	150704343	[redacted]	-0.302187	[redacted]
chr18	36142239	36142296	[redacted]	-0.302174	[redacted]
chr4	53147913	53148004	[redacted]	-0.302174	[redacted]
chr2	106447000	106447048	[redacted]	-0.302158	[redacted]
chr11	108952404	108952465	[redacted]	-0.302156	[redacted]
chr3	104253710	104253773	[redacted]	-0.302153	[redacted]
chr11	32814313	32814347	[redacted]	-0.302152	[redacted]
chr13	103114303	103114370	[redacted]	-0.302147	[redacted]
chr9	85206714	85206796	[redacted]	-0.302104	[redacted]
chr2	91310300	91310351	[redacted]	-0.302076	[redacted]
chrX	157429761	157429818	[redacted]	-0.302062	[redacted]
chr13	20267627	20267705	[redacted]	-0.302055	[redacted]
chr5	67668442	67668478	[redacted]	-0.302045	[redacted]
chr19	12542685	12542731	[redacted]	-0.302034	[redacted]
chr15	15479293	15479320	[redacted]	-0.302011	[redacted]
chr2	72579947	72580027	[redacted]	-0.302	[redacted]
chr8	64990806	64990853	[redacted]	-0.301961	[redacted]
chr11	6382058	6382113	[redacted]	-0.301956	[redacted]
chr7	127686976	127686997	[redacted]	-0.301941	[redacted]
chr3	99540915	99540976	[redacted]	-0.30194	[redacted]
chr6	14897846	14897867	[redacted]	-0.301937	[redacted]
chr6	95874557	95874602	[redacted]	-0.301909	[redacted]
chr9	8447454	8447507	[redacted]	-0.301905	[redacted]

chrX	126108177	126108247	[redacted]	-0.301905	[redacted]
chr17	47811630	47811710	[redacted]	-0.301886	[redacted]
chr19	38365217	38365295	[redacted]	-0.301876	[redacted]
chr6	122349748	122349800	[redacted]	-0.301874	[redacted]
chr7	99671497	99671585	[redacted]	-0.30187	[redacted]
chr5	147651116	147651136	[redacted]	-0.301867	[redacted]
chr11	43343594	43343642	[redacted]	-0.301859	[redacted]
chr19	54215173	54215258	[redacted]	-0.301853	[redacted]
chr7	141346410	141346477	[redacted]	-0.301838	[redacted]
chr9	96952512	96952558	[redacted]	-0.301831	[redacted]
chr10	60237476	60237532	[redacted]	-0.301828	[redacted]
chr19	40716081	40716145	[redacted]	-0.301827	[redacted]
chr10	70557810	70557872	[redacted]	-0.301823	[redacted]
chr9	30061963	30062028	[redacted]	-0.301819	[redacted]
chr4	25259904	25259974	[redacted]	-0.301818	[redacted]
chr1	71111843	71111891	[redacted]	-0.301813	[redacted]
chr16	63423957	63424031	[redacted]	-0.301803	[redacted]
chr18	69994521	69994583	[redacted]	-0.301791	[redacted]
chr6	149494565	149494633	[redacted]	-0.301781	[redacted]
chr18	50084798	50084862	[redacted]	-0.301772	[redacted]
chr13	51352594	51352626	[redacted]	-0.301756	[redacted]
chr10	64674623	64674673	[redacted]	-0.301752	[redacted]
chr4	115093094	115093153	[redacted]	-0.301745	[redacted]
chr6	21853608	21853656	[redacted]	-0.301743	[redacted]
chr18	75773709	75773781	[redacted]	-0.301735	[redacted]
chr3	69770249	69770287	[redacted]	-0.30173	[redacted]
chr7	142940803	142940870	[redacted]	-0.301724	[redacted]
chr2	171133218	171133304	[redacted]	-0.301708	[redacted]
chr4	101750704	101750762	[redacted]	-0.301678	[redacted]
chr12	72510253	72510339	[redacted]	-0.301678	[redacted]
chr10	117353288	117353348	[redacted]	-0.301672	[redacted]
chr12	74495613	74495680	[redacted]	-0.301672	[redacted]
chr9	82882739	82882778	[redacted]	-0.30166	[redacted]
chr10	67498016	67498040	[redacted]	-0.301656	[redacted]
chr1	94650059	94650092	[redacted]	-0.301649	[redacted]
chr3	102773181	102773256	[redacted]	-0.301617	[redacted]
chr18	46327879	46327923	[redacted]	-0.301609	[redacted]
chr11	21848046	21848083	[redacted]	-0.301594	[redacted]
chr8	9702640	9702695	[redacted]	-0.30159	[redacted]
chr10	16838500	16838554	[redacted]	-0.301589	[redacted]
chr16	90643817	90643866	[redacted]	-0.301581	[redacted]
chr13	53983274	53983344	[redacted]	-0.30158	[redacted]
chr1	70896059	70896117	[redacted]	-0.301569	[redacted]

chr1	88027679	88027725	[redacted]	-0.301561	[redacted]
chr12	12367619	12367684	[redacted]	-0.30156	[redacted]
chr4	103095535	103095584	[redacted]	-0.301546	[redacted]
chr13	3636590	3636657	[redacted]	-0.301545	[redacted]
chr11	70524243	70524327	[redacted]	-0.30154	[redacted]
chr7	68264426	68264446	[redacted]	-0.301534	[redacted]
chr5	38290981	38291025	[redacted]	-0.30153	[redacted]
chr11	5516135	5516199	[redacted]	-0.301526	[redacted]
chr19	14811383	14811439	[redacted]	-0.301526	[redacted]
chr3	59610849	59610939	[redacted]	-0.301514	[redacted]
chr13	97966398	97966435	[redacted]	-0.30151	[redacted]
chr14	102662456	102662528	[redacted]	-0.301507	[redacted]
chr6	112679808	112679887	[redacted]	-0.301506	[redacted]
chr6	124005876	124005915	[redacted]	-0.301505	[redacted]
chr19	35222702	35222767	[redacted]	-0.301496	[redacted]
chr2	10927765	10927830	[redacted]	-0.301493	[redacted]
chr4	123649288	123649328	[redacted]	-0.301462	[redacted]
chr18	61782439	61782481	[redacted]	-0.301452	[redacted]
chr17	69648535	69648594	[redacted]	-0.301449	[redacted]
chr16	33090476	33090554	[redacted]	-0.301445	[redacted]
chr5	7946170	7946260	[redacted]	-0.301435	[redacted]
chr1	43100765	43100792	[redacted]	-0.301427	[redacted]
chr15	102754565	102754647	[redacted]	-0.301411	[redacted]
chr14	84452322	84452395	[redacted]	-0.3014	[redacted]
chr10	19781624	19781677	[redacted]	-0.301397	[redacted]
chr13	102327263	102327337	[redacted]	-0.301394	[redacted]
chr6	14147800	14147835	[redacted]	-0.301392	[redacted]
chr11	31059708	31059786	[redacted]	-0.301391	[redacted]
chr3	42651731	42651801	[redacted]	-0.301378	[redacted]
chr17	83246029	83246080	[redacted]	-0.301364	[redacted]
chr9	60363154	60363228	[redacted]	-0.301364	[redacted]
chr8	22523660	22523674	[redacted]	-0.301359	[redacted]
chr16	10294624	10294699	[redacted]	-0.301357	[redacted]
chr4	155704306	155704374	[redacted]	-0.301356	[redacted]
chr1	59713590	59713695	[redacted]	-0.301354	[redacted]
chr1	192899648	192899716	[redacted]	-0.30133	[redacted]
chr3	132725698	132725761	[redacted]	-0.301327	[redacted]
chr10	116181748	116181824	[redacted]	-0.301321	[redacted]
chr2	180224232	180224301	[redacted]	-0.301317	[redacted]
chr11	5935933	5935975	[redacted]	-0.301315	[redacted]
chr9	63693503	63693572	[redacted]	-0.301297	[redacted]
chr5	21265336	21265363	[redacted]	-0.301291	[redacted]
chr4	141940396	141940459	[redacted]	-0.30129	[redacted]

chr6	38700864	38700903	[redacted]	-0.301289	[redacted]
chr9	53474712	53474772	[redacted]	-0.301285	[redacted]
chr9	20087151	20087235	[redacted]	-0.301283	[redacted]
chr3	53165010	53165088	[redacted]	-0.301281	[redacted]
chr9	72477665	72477739	[redacted]	-0.30126	[redacted]
chr17	80288034	80288065	[redacted]	-0.301259	[redacted]
chr19	5124068	5124100	[redacted]	-0.30125	[redacted]
chr11	98771170	98771273	[redacted]	-0.301248	[redacted]
chr6	124684278	124684353	[redacted]	-0.301228	[redacted]
chr6	128828322	128828434	[redacted]	-0.30122	[redacted]
chr13	51021227	51021264	[redacted]	-0.301218	[redacted]
chr8	26304725	26304747	[redacted]	-0.301216	[redacted]
chr2	179021723	179021797	[redacted]	-0.3012	[redacted]
chrX	142580636	142580691	[redacted]	-0.301189	[redacted]
chr6	15827964	15828077	[redacted]	-0.301173	[redacted]
chr11	23029499	23029563	[redacted]	-0.301173	[redacted]
chr17	55725464	55725528	[redacted]	-0.301153	[redacted]
chr14	34042075	34042131	[redacted]	-0.301148	[redacted]
chr9	3721465	3721547	[redacted]	-0.301141	[redacted]
chr5	17718330	17718369	[redacted]	-0.301134	[redacted]
chr1	34040703	34040764	[redacted]	-0.301129	[redacted]
chr7	37807868	37808054	[redacted]	-0.301121	[redacted]
chr10	66335817	66335884	[redacted]	-0.30112	[redacted]
chr13	96685740	96685823	[redacted]	-0.301119	[redacted]
chr2	84471143	84471212	[redacted]	-0.301105	[redacted]
chr11	35152710	35152782	[redacted]	-0.301104	[redacted]
chr17	8169530	8169551	[redacted]	-0.3011	[redacted]
chr12	34664715	34664757	[redacted]	-0.301098	[redacted]
chr10	116561137	116561191	[redacted]	-0.301089	[redacted]
chrX	8179833	8179888	[redacted]	-0.301079	[redacted]
chr15	98512300	98512331	[redacted]	-0.301079	[redacted]
chr4	35427747	35427795	[redacted]	-0.301075	[redacted]
chr2	34266418	34266477	[redacted]	-0.301055	[redacted]
chr14	83019352	83019438	[redacted]	-0.301054	[redacted]
chr13	61190284	61190357	[redacted]	-0.301049	[redacted]
chr10	120788256	120788352	[redacted]	-0.30104	[redacted]
chr3	150394106	150394144	[redacted]	-0.301034	[redacted]
chr2	28703672	28703706	[redacted]	-0.301033	[redacted]
chr2	156622706	156622758	[redacted]	-0.300994	[redacted]
chr3	81782187	81782233	[redacted]	-0.300992	[redacted]
chr7	128257527	128257556	[redacted]	-0.30099	[redacted]
chr13	109599665	109599747	[redacted]	-0.300986	[redacted]
chr13	94441135	94441158	[redacted]	-0.300976	[redacted]

chr2	126757556	126757647	[redacted]	-0.300968	[redacted]
chr11	121207795	121207842	[redacted]	-0.300921	[redacted]
chr7	30299809	30299868	[redacted]	-0.300908	[redacted]
chr16	26455664	26455743	[redacted]	-0.300908	[redacted]
chr11	109619076	109619128	[redacted]	-0.300879	[redacted]
chr18	47412120	47412197	[redacted]	-0.300876	[redacted]
chr5	93265313	93265374	[redacted]	-0.300863	[redacted]
chr12	96196298	96196358	[redacted]	-0.300857	[redacted]
chr15	82002003	82002032	[redacted]	-0.300854	[redacted]
chr10	79578094	79578147	[redacted]	-0.300849	[redacted]
chr7	65383999	65384026	[redacted]	-0.300829	[redacted]
chr18	53773346	53773426	[redacted]	-0.300827	[redacted]
chr2	10814294	10814312	[redacted]	-0.300822	[redacted]
chr8	115045269	115045330	[redacted]	-0.300821	[redacted]
chr6	72621246	72621283	[redacted]	-0.300818	[redacted]
chr9	7590173	7590238	[redacted]	-0.300813	[redacted]
chr8	38688037	38688114	[redacted]	-0.300808	[redacted]
chr10	9981622	9981718	[redacted]	-0.300797	[redacted]
chr8	4974495	4974623	[redacted]	-0.300793	[redacted]
chr17	73207843	73207897	[redacted]	-0.300792	[redacted]
chr18	48171600	48171667	[redacted]	-0.300781	[redacted]
chr1	63352357	63352411	[redacted]	-0.300775	[redacted]
chr2	156941174	156941223	[redacted]	-0.300775	[redacted]
chr2	165207678	165207762	[redacted]	-0.30077	[redacted]
chr2	158227649	158227721	[redacted]	-0.300767	[redacted]
chr17	60288658	60288749	[redacted]	-0.300762	[redacted]
chr15	30322263	30322306	[redacted]	-0.300747	[redacted]
chr18	10482059	10482239	[redacted]	-0.300746	[redacted]
chr1	13708760	13708821	[redacted]	-0.300742	[redacted]
chr12	9640242	9640289	[redacted]	-0.300731	[redacted]
chr15	81383040	81383151	[redacted]	-0.300717	[redacted]
chr11	55296074	55296140	[redacted]	-0.300705	[redacted]
chr15	81493939	81494028	[redacted]	-0.300702	[redacted]
chr16	87136313	87136345	[redacted]	-0.300701	[redacted]
chr9	22420742	22420853	[redacted]	-0.300699	[redacted]
chr12	24912454	24912495	[redacted]	-0.300697	[redacted]
chr4	48760652	48760696	[redacted]	-0.300678	[redacted]
chr4	44069710	44069731	[redacted]	-0.300655	[redacted]
chr13	53179594	53179655	[redacted]	-0.300652	[redacted]
chr13	4544409	4544513	[redacted]	-0.300647	[redacted]
chr10	67034288	67034343	[redacted]	-0.300643	[redacted]
chr15	73542788	73542870	[redacted]	-0.300643	[redacted]
chr13	30503268	30503296	[redacted]	-0.300642	[redacted]

chr7	7368907	7368990	[redacted]	-0.300629	[redacted]
chr4	12321841	12321856	[redacted]	-0.300611	[redacted]
chr3	29864101	29864168	[redacted]	-0.30058	[redacted]
chr8	10526139	10526161	[redacted]	-0.300569	[redacted]
chr9	103921590	103921627	[redacted]	-0.300566	[redacted]
chr17	8925218	8925302	[redacted]	-0.300551	[redacted]
chr15	76234496	76234541	[redacted]	-0.300539	[redacted]
chr2	93823406	93823539	[redacted]	-0.300535	[redacted]
chr1	89393976	89394028	[redacted]	-0.300535	[redacted]
chr11	67330831	67330894	[redacted]	-0.300514	[redacted]
chr1	70387515	70387592	[redacted]	-0.300512	[redacted]
chr13	62349296	62349387	[redacted]	-0.300505	[redacted]
chr8	110447309	110447436	[redacted]	-0.300492	[redacted]
chr4	46396991	46397066	[redacted]	-0.300479	[redacted]
chr15	10515490	10515526	[redacted]	-0.300478	[redacted]
chr11	68483154	68483189	[redacted]	-0.300477	[redacted]
chr11	32149291	32149368	[redacted]	-0.30047	[redacted]
chr17	24690278	24690323	[redacted]	-0.300467	[redacted]
chr15	80487281	80487321	[redacted]	-0.300465	[redacted]
chrX	128560225	128560282	[redacted]	-0.300429	[redacted]
chr4	86783139	86783216	[redacted]	-0.300424	[redacted]
chr18	54744601	54744734	[redacted]	-0.30042	[redacted]
chr7	49520964	49521065	[redacted]	-0.300407	[redacted]
chr14	76591857	76591923	[redacted]	-0.300407	[redacted]
chr2	76555776	76555837	[redacted]	-0.300404	[redacted]
chr17	53687724	53687792	[redacted]	-0.300402	[redacted]
chr10	73446681	73446736	[redacted]	-0.3004	[redacted]
chr1	71920531	71920593	[redacted]	-0.300398	[redacted]
chr15	89382424	89382475	[redacted]	-0.300376	[redacted]
chr4	44225316	44225366	[redacted]	-0.300355	[redacted]
chrX	77707728	77707791	[redacted]	-0.300351	[redacted]
chr3	135893095	135893151	[redacted]	-0.300346	[redacted]
chr12	78409167	78409208	[redacted]	-0.300337	[redacted]
chr2	115664822	115664864	[redacted]	-0.300334	[redacted]
chr16	41016832	41016897	[redacted]	-0.300331	[redacted]
chrX	94509118	94509172	[redacted]	-0.300308	[redacted]
chr3	136988488	136988540	[redacted]	-0.300302	[redacted]
chr11	53015867	53015923	[redacted]	-0.300286	[redacted]
chr4	107718379	107718416	[redacted]	-0.300269	[redacted]
chr5	118185739	118185839	[redacted]	-0.300255	[redacted]
chr3	118797457	118797516	[redacted]	-0.300242	[redacted]
chr15	30777266	30777304	[redacted]	-0.300216	[redacted]
chr14	75193264	75193332	[redacted]	-0.300206	[redacted]

chr1	174589275	174589359		-0.300196	
chr16	64902924	64902994		-0.300184	
chr15	98449122	98449197		-0.300181	
chr11	103727927	103727982		-0.300178	
chr8	122842695	122842808		-0.300167	
chr1	12158833	12158891		-0.300153	
chr11	105166229	105166446		-0.300108	
chr11	84915506	84915525		-0.300106	
chr2	121193994	121194026		-0.300105	
chr16	7823822	7823861		-0.300104	
chr17	39146162	39146210		-0.300102	
chr9	81822293	81822345		-0.3001	
chr14	117056633	117056708		-0.300099	
chr5	105608981	105609014		-0.300092	
chr18	82646448	82646577		-0.300091	
chr3	147938330	147938398		-0.300091	
chr13	56062194	56062255		-0.300072	
chr2	24805716	24805785		-0.30006	
chr4	59410546	59410594		-0.300058	
chrX	103483993	103484090		-0.300058	
chr3	82819826	82819901		-0.300057	
chr18	78758656	78758770		-0.300052	
chr2	120377043	120377096		-0.30005	
chr6	87044555	87044607		-0.300048	
chr6	127737124	127737184		-0.300037	
chr7	126188151	126188207		-0.300021	
chr2	84262010	84262093		-0.30002	
chr8	71531438	71531496		-0.300014	
chr14	59827786	59827857		-0.300012	
chr2	166735522	166735575		-0.300009	
chr9	69673667	69673726		-0.300008	
chr13	85976369	85976440		-0.300007	
chr14	8587948	8588029		-0.300007	
chr11	75594252	75594282	0.300504		0.334796
chr10	98942385	98942488	0.300982		0.31789
chr10	98831629	98831735	0.30102		0.346706
chr4	15888867	15888983	0.301262		0.495872
chr15	50361483	50361520	0.30128		0.387118
chr9	63246987	63247011	0.301354		0.41457
chr16	26691627	26691714	0.301369		0.347006
chr15	101155451	101155566	0.302079		0.312267
chr17	84124326	84124365	0.30216		0.311272
chr9	105554262	105554278	0.302362		0.427424

chr10	127590299	127590361	0.302486	[redacted]	0.448144
chr9	117656985	117657019	0.302496	[redacted]	0.334797
chr7	30438331	30438357	0.303037	[redacted]	0.300756
chr19	28208084	28208130	0.303475	[redacted]	0.502869
chr14	69957389	69957449	0.304317	[redacted]	0.366544
chr10	20233683	20233776	0.304873	[redacted]	0.304318
chr14	117625612	117625633	0.305169	[redacted]	0.397308
chr19	29498098	29498160	0.305185	[redacted]	0.326407
chr19	10843002	10843131	0.305612	[redacted]	0.351801
chr4	97752174	97752225	0.306094	[redacted]	0.340351
chr4	151742486	151742522	0.306145	[redacted]	0.378248
chr10	95319101	95319152	0.306367	[redacted]	0.354474
chr14	70081755	70081808	0.306572	[redacted]	0.311853
chr10	8037838	8037884	0.306975	[redacted]	0.304601
chr2	57208569	57208597	0.307917	[redacted]	0.399917
chr4	45883488	45883582	0.307978	[redacted]	0.385119
chr2	72369069	72369094	0.307989	[redacted]	0.369853
chr16	76053910	76053952	0.308175	[redacted]	0.35614
chr15	100494584	100494622	0.308403	[redacted]	0.352041
chr13	54838729	54838788	0.308487	[redacted]	0.422543
chr9	55237152	55237190	0.308654	[redacted]	0.312194
chr9	110452575	110452667	0.30878	[redacted]	0.32346
chr11	70445679	70445737	0.309089	[redacted]	0.39031
chr14	8518329	8518367	0.309257	[redacted]	0.415257
chr17	35867240	35867297	0.309567	[redacted]	0.415625
chr3	9521003	9521058	0.309574	[redacted]	0.380784
chr5	86318282	86318295	0.309837	[redacted]	0.389926
chr14	96990741	96990834	0.310059	[redacted]	0.550603
chr2	106650667	106650678	0.311208	[redacted]	0.320384
chr4	117488628	117488687	0.311418	[redacted]	0.35314
chr1	35862351	35862422	0.311487	[redacted]	0.314327
chr16	64879954	64879989	0.311759	[redacted]	0.347099
chr12	117688667	117688712	0.314054	[redacted]	0.305533
chr7	112678267	112678333	0.314532	[redacted]	0.308826
chr5	115363191	115363295	0.315632	[redacted]	0.312471
chr4	87487817	87487887	0.317777	[redacted]	0.377365
chr4	127389594	127389666	0.318044	[redacted]	0.357034
chr4	141547685	141547733	0.318441	[redacted]	0.507039
chr10	117215576	117215767	0.318488	[redacted]	0.480875
chr19	29655271	29655366	0.318698	[redacted]	0.337387
chr9	70237997	70238041	0.318848	[redacted]	0.320399
chr4	47893607	47893635	0.318992	[redacted]	0.550731
chr1	177444120	177444181	0.31915	[redacted]	0.357329

chr18	37848885	37848926	0.319353	[redacted]	0.365385
chr7	79467351	79467375	0.31992	[redacted]	0.43676
chr17	27075047	27075094	0.320084	[redacted]	0.300893
chr8	25353864	25353893	0.320103	[redacted]	0.444803
chr11	70203845	70203938	0.320615	[redacted]	0.359524
chr18	38928575	38928603	0.321391	[redacted]	0.416835
chr15	80564282	80564345	0.322998	[redacted]	0.379928
chr13	28883894	28883975	0.323071	[redacted]	0.350885
chr2	122630270	122630375	0.323502	[redacted]	0.440631
chr2	62498681	62498749	0.323583	[redacted]	0.488117
chr14	70008368	70008414	0.324002	[redacted]	0.400006
chr9	56146403	56146410	0.324545	[redacted]	0.440433
chr16	28798432	28798507	0.324641	[redacted]	0.319309
chr6	108208806	108208858	0.324854	[redacted]	0.307026
chr11	53487907	53487961	0.324975	[redacted]	0.317744
chr12	85469208	85469266	0.32516	[redacted]	0.331414
chr8	105565029	105565046	0.326444	[redacted]	0.37325
chr10	75473502	75473550	0.326533	[redacted]	0.392219
chr12	85497436	85497500	0.326728	[redacted]	0.381058
chr5	53540506	53540550	0.326756	[redacted]	0.381113
chr17	35787973	35788062	0.326786	[redacted]	0.348128
chr9	78366268	78366277	0.326826	[redacted]	0.352467
chrX	36839495	36839568	0.326918	[redacted]	0.36453
chr7	37649169	37649194	0.327334	[redacted]	0.493325
chr15	68228570	68228652	0.327537	[redacted]	0.429375
chr18	66460709	66460797	0.327963	[redacted]	0.484247
chr14	64834598	64834648	0.328241	[redacted]	0.330538
chr11	96681303	96681341	0.328738	[redacted]	0.413512
chr10	116443684	116443759	0.32889	[redacted]	0.312518
chr4	132563836	132563919	0.329058	[redacted]	0.46008
chr16	21607029	21607094	0.329069	[redacted]	0.486412
chr7	127082383	127082438	0.329322	[redacted]	0.343356
chr8	57441044	57441135	0.329804	[redacted]	0.312976
chr3	151749947	151750028	0.330576	[redacted]	0.463643
chr1	155283986	155284065	0.330732	[redacted]	0.358057
chr19	26966554	26966613	0.330739	[redacted]	0.439704
chr7	98562383	98562449	0.331168	[redacted]	0.381921
chr13	46360871	46360882	0.33129	[redacted]	0.377696
chr5	122456521	122456548	0.33132	[redacted]	0.560989
chr6	124639262	124639299	0.331657	[redacted]	0.460021
chr1	136331392	136331406	0.331931	[redacted]	0.381695
chr12	98996609	98996670	0.33223	[redacted]	0.340331
chr9	42095073	42095080	0.332519	[redacted]	0.323496

chr3	10317139	10317212	0.335134	[redacted]	0.370872
chr2	24545523	24545623	0.335671	[redacted]	0.367865
chr13	57605294	57605350	0.336567	[redacted]	0.31547
chr1	171629950	171629971	0.337874	[redacted]	0.538068
chr6	28319088	28319196	0.338082	[redacted]	0.595169
chr18	82695206	82695230	0.338441	[redacted]	0.35189
chr2	154353825	154353856	0.338448	[redacted]	0.329321
chr17	48458075	48458143	0.338824	[redacted]	0.303668
chr10	119487326	119487356	0.338863	[redacted]	0.400412
chr2	91459489	91459544	0.33895	[redacted]	0.306907
chr11	51898436	51898471	0.339018	[redacted]	0.424305
chr13	101664262	101664321	0.339361	[redacted]	0.364957
chr12	77240418	77240540	0.339844	[redacted]	0.485113
chr16	95445873	95445923	0.340714	[redacted]	0.349765
chr15	64198095	64198163	0.34083	[redacted]	0.355745
chr2	35061104	35061138	0.341389	[redacted]	0.351536
chr6	121206944	121206982	0.341554	[redacted]	0.406902
chr17	35686524	35686597	0.341933	[redacted]	0.372743
chr6	22402421	22402480	0.342122	[redacted]	0.396892
chr10	80565738	80565812	0.342917	[redacted]	0.44485
chr7	29784652	29784758	0.343209	[redacted]	0.428585
chr13	49253038	49253075	0.343522	[redacted]	0.328548
chr2	169610143	169610163	0.343707	[redacted]	0.342625
chr1	35332327	35332384	0.343715	[redacted]	0.621289
chr17	30658016	30658083	0.344158	[redacted]	0.42041
chr5	123509199	123509216	0.344166	[redacted]	0.306785
chr5	67846030	67846097	0.344653	[redacted]	0.312251
chr12	117916789	117916810	0.34471	[redacted]	0.343502
chr5	136603327	136603381	0.345209	[redacted]	0.434835
chr17	30866747	30866860	0.345396	[redacted]	0.360177
chr9	40343266	40343317	0.345714	[redacted]	0.346777
chr16	31209593	31209629	0.345896	[redacted]	0.353095
chr10	70152580	70152666	0.346101	[redacted]	0.339101
chr2	65333865	65333892	0.346447	[redacted]	0.493787
chr7	25651339	25651385	0.347201	[redacted]	0.418733
chr16	17759190	17759346	0.347414	[redacted]	0.477569
chr7	19459251	19459304	0.348181	[redacted]	0.41638
chr13	21004853	21004929	0.348288	[redacted]	0.384147
chr11	96729628	96729671	0.348519	[redacted]	0.335174
chr4	107675684	107675699	0.348703	[redacted]	0.300959
chr2	92205648	92205697	0.34894	[redacted]	0.448255
chr9	64797913	64797990	0.349072	[redacted]	0.363037
chr13	83341558	83341579	0.349076	[redacted]	0.478114

chr6	34867869	34867888	0.3491	[redacted]	0.431401
chr9	52167885	52167924	0.349613	[redacted]	0.434083
chr18	73650146	73650190	0.350008	[redacted]	0.362311
chr18	81399204	81399254	0.350291	[redacted]	0.382372
chr7	47157572	47157627	0.350291	[redacted]	0.461138
chr19	53792304	53792328	0.350523	[redacted]	0.589819
chr16	51428837	51428873	0.351117	[redacted]	0.306333
chr10	69918969	69918978	0.351526	[redacted]	0.459707
chr17	22817338	22817402	0.351574	[redacted]	0.334686
chr17	44677887	44677948	0.352197	[redacted]	0.57394
chr5	91283345	91283414	0.352317	[redacted]	0.564853
chr12	102605770	102605827	0.352652	[redacted]	0.42091
chr10	107991513	107991547	0.35267	[redacted]	0.343502
chr15	50802442	50802514	0.352993	[redacted]	0.386661
chr17	44858812	44858859	0.353055	[redacted]	0.360693
chr7	122868287	122868328	0.354793	[redacted]	0.393447
chr3	136376933	136376976	0.354989	[redacted]	0.520268
chr7	30939202	30939238	0.355089	[redacted]	0.392445
chr7	98803312	98803364	0.355718	[redacted]	0.399641
chr8	89534608	89534637	0.355721	[redacted]	0.378543
chr19	24636622	24636659	0.355953	[redacted]	0.42565
chr10	13173017	13173026	0.356105	[redacted]	0.385052
chr15	52739971	52740040	0.356257	[redacted]	0.511509
chr9	80286951	80287093	0.356511	[redacted]	0.446374
chr8	118936856	118936898	0.356648	[redacted]	0.346657
chr4	132795804	132795843	0.356795	[redacted]	0.353414
chr3	132947580	132947628	0.357555	[redacted]	0.374426
chr7	110242168	110242174	0.358374	[redacted]	0.370607
chr1	168598499	168598514	0.358425	[redacted]	0.398532
chr15	80477285	80477320	0.35846	[redacted]	0.303823
chr17	39843001	39843769	0.35949	[redacted]	0.400515
chr6	54954845	54954858	0.360074	[redacted]	0.424408
chr4	45532107	45532162	0.36012	[redacted]	0.404537
chr15	40020979	40021039	0.360147	[redacted]	0.391927
chr1	136516374	136516415	0.360456	[redacted]	0.3307
chr2	69856627	69856680	0.361079	[redacted]	0.485329
chr7	68278084	68278141	0.361783	[redacted]	0.332357
chr9	96544126	96544188	0.362221	[redacted]	0.358454
chr8	14088856	14088911	0.362512	[redacted]	0.639092
chr1	93071805	93071949	0.362533	[redacted]	0.330271
chr9	102860108	102860225	0.363062	[redacted]	0.510219
chr10	84549822	84549840	0.363088	[redacted]	0.427429
chr3	123314402	123314460	0.363648	[redacted]	0.393519

chr9	51767244	51767279	0.364395	[redacted]	0.316011
chr1	87960258	87960301	0.365152	[redacted]	0.419015
chr11	121605687	121605747	0.365612	[redacted]	0.407025
chr12	107812802	107812829	0.366154	[redacted]	0.4819
chr3	51378332	51378388	0.36643	[redacted]	0.347158
chr11	112090298	112090355	0.366456	[redacted]	0.430188
chr5	111058466	111058743	0.36654	[redacted]	0.354672
chr17	7468727	7468742	0.366671	[redacted]	0.410071
chr1	55446455	55446465	0.367166	[redacted]	0.576711
chr2	26608192	26608222	0.368704	[redacted]	0.50965
chr4	129556354	129556422	0.370215	[redacted]	0.359484
chr15	81210456	81210492	0.370276	[redacted]	0.349405
chr16	33921917	33922070	0.370341	[redacted]	0.358886
chr4	45794889	45794999	0.370965	[redacted]	0.431446
chr10	16682174	16682212	0.371032	[redacted]	0.328937
chr3	154647165	154647199	0.371422	[redacted]	0.302648
chr17	29118314	29118365	0.371665	[redacted]	0.362836
chr7	98562505	98562542	0.37212	[redacted]	0.442706
chr3	51311330	51311371	0.372162	[redacted]	0.388415
chr16	31909176	31909200	0.372605	[redacted]	0.461683
chr17	72208873	72208900	0.372631	[redacted]	0.58543
chr12	51969667	51969728	0.372715	[redacted]	0.355491
chr6	52937174	52937268	0.372939	[redacted]	0.410443
chr3	94867696	94867724	0.373066	[redacted]	0.363324
chr19	46645961	46646032	0.374868	[redacted]	0.416241
chr6	144483570	144483642	0.375094	[redacted]	0.400893
chr5	77213362	77213418	0.375536	[redacted]	0.50471
chr17	45137904	45137959	0.376181	[redacted]	0.342884
chrX	12840522	12840596	0.376417	[redacted]	0.397622
chr8	10794655	10794706	0.376452	[redacted]	0.386494
chr10	62891069	62891137	0.376476	[redacted]	0.490785
chr13	109904529	109904553	0.377599	[redacted]	0.337577
chr2	51509772	51509823	0.377778	[redacted]	0.418548
chr11	64588886	64588925	0.379689	[redacted]	0.463638
chr7	46586612	46586666	0.379869	[redacted]	0.400268
chr10	20471963	20472059	0.380049	[redacted]	0.497131
chr10	99247356	99247407	0.380064	[redacted]	0.414619
chr8	26162688	26162731	0.38041	[redacted]	0.361467
chr8	32160074	32160162	0.380574	[redacted]	0.304058
chr11	74531297	74531384	0.38106	[redacted]	0.351336
chr3	103927729	103927737	0.382547	[redacted]	0.488744
chr5	100750772	100750830	0.383876	[redacted]	0.403876
chr9	70334263	70334347	0.383884	[redacted]	0.500683

chr8	4162721	4162735	0.384147		0.526242
chr9	60808247	60808273	0.384195		0.319053
chr18	81462168	81462325	0.384239		0.440095
chr14	122491561	122491603	0.384274		0.479509
chr8	95021463	95021534	0.384342		0.398813
chr4	117492255	117492278	0.384754		0.444272
chr1	95573089	95573169	0.384897		0.466894
chr10	24327468	24327491	0.38495		0.337839
chr4	44963305	44963326	0.386039		0.560473
chr17	52311918	52311965	0.386426		0.373499
chr1	160408707	160408741	0.387314		0.357078
chr5	73904610	73904642	0.387657		0.425237
chr8	46742241	46742281	0.388053		0.335986
chr18	84355435	84355494	0.388157		0.473608
chr5	124014974	124015022	0.389273		0.441954
chr6	136344147	136344183	0.389953		0.387515
chr14	77037143	77037190	0.390692		0.315778
chr17	3521618	3521684	0.390976		0.407122
chr2	59327978	59328036	0.391147		0.35434
chr16	10713546	10713603	0.392419		0.665599
chr1	106321716	106321738	0.393325		0.48423
chr2	26625375	26625436	0.39421		0.513714
chr15	38015006	38015071	0.394306		0.341351
chr12	100712005	100712062	0.395052		0.394692
chr13	68597414	68597518	0.395079		0.303606
chr1	14667261	14667330	0.395091		0.322802
chr12	32007193	32007322	0.396392		0.475898
chr4	151006374	151006427	0.396641		0.452283
chr6	73315647	73315677	0.396954		0.353492
chr2	62461206	62461235	0.398238		0.343643
chr2	49333713	49333737	0.39857		0.762744
chr1	14667819	14667831	0.398733		0.465936
chr18	58009246	58009270	0.400052		0.5691
chr8	84872982	84873056	0.40006		0.415026
chr10	67540273	67540319	0.400722		0.410454
chr8	122962367	122962410	0.400883		0.301684
chr6	136921880	136921929	0.401309		0.413114
chr5	148970220	148970254	0.401315		0.389508
chr9	77470396	77470415	0.401435		0.43482
chr18	37985661	37985750	0.402111		0.513822
chr5	125478209	125478240	0.402323		0.432979
chr8	46753095	46753174	0.402515		0.372924
chr18	77835706	77835757	0.403315		0.330522

chr8	61286826	61286923	0.403434	[redacted]	0.40531
chr9	115654896	115654967	0.40377	[redacted]	0.430723
chr11	107198812	107198840	0.404258	[redacted]	0.345056
chr18	39801398	39801461	0.404724	[redacted]	0.357865
chr11	25523054	25523093	0.405162	[redacted]	0.487
chr13	78483376	78483427	0.405169	[redacted]	0.348015
chr13	30901524	30901551	0.405459	[redacted]	0.490403
chrX	101784316	101784345	0.406125	[redacted]	0.410744
chr11	116361606	116361644	0.406556	[redacted]	0.416704
chr15	42207639	42207694	0.408466	[redacted]	0.392424
chr11	54029291	54029353	0.408498	[redacted]	0.453771
chr1	59253162	59253175	0.408631	[redacted]	0.38307
chr17	75366316	75366350	0.408964	[redacted]	0.434697
chr16	52464437	52464483	0.409079	[redacted]	0.332382
chr13	117194816	117194881	0.409184	[redacted]	0.341477
chr2	35882517	35882532	0.409488	[redacted]	0.304789
chr1	179958950	179958993	0.410261	[redacted]	0.516461
chr8	83989509	83989531	0.410359	[redacted]	0.492887
chr10	69533846	69533896	0.410408	[redacted]	0.357778
chr8	64832058	64832166	0.410806	[redacted]	0.439606
chr11	97642006	97642024	0.411239	[redacted]	0.437333
chr3	65873147	65873227	0.411396	[redacted]	0.496907
chr7	25782867	25782906	0.412011	[redacted]	0.366204
chr16	93819030	93819102	0.41303	[redacted]	0.442112
chr17	59298181	59298212	0.413656	[redacted]	0.41623
chr2	75613361	75613440	0.41455	[redacted]	0.396738
chr10	93939194	93939251	0.415509	[redacted]	0.524284
chr9	70206434	70206482	0.416858	[redacted]	0.444777
chr5	114922563	114922663	0.418459	[redacted]	0.575295
chr15	66729043	66729081	0.418845	[redacted]	0.462846
chr11	44546670	44546712	0.418997	[redacted]	0.453315
chr12	117688764	117688777	0.41917	[redacted]	0.404279
chr2	35327780	35327854	0.420134	[redacted]	0.404599
chr15	93790900	93790952	0.420843	[redacted]	0.363925
chr13	43666988	43667049	0.421172	[redacted]	0.322115
chr2	116972647	116972743	0.421637	[redacted]	0.496284
chr13	83530015	83530137	0.421845	[redacted]	0.385685
chr4	135122471	135122551	0.422482	[redacted]	0.390357
chr19	17828730	17828748	0.423053	[redacted]	0.4903
chr14	54351563	54351622	0.423513	[redacted]	0.354264
chr1	161798731	161798752	0.424168	[redacted]	0.457102
chr7	25652519	25652547	0.424561	[redacted]	0.452854
chr11	116572223	116572366	0.42578	[redacted]	0.570373

chr2	18687921	18687983	0.425983	[redacted]	0.493074
chr10	44427498	44427558	0.426656	[redacted]	0.396767
chr16	35981733	35981755	0.426876	[redacted]	0.305821
chr13	32670756	32670839	0.42898	[redacted]	0.437598
chr13	104818570	104818619	0.429004	[redacted]	0.396978
chr1	64756945	64757014	0.429299	[redacted]	0.58464
chr3	51603950	51604064	0.430064	[redacted]	0.638027
chr8	24311125	24311176	0.430449	[redacted]	0.634148
chr3	38672268	38672299	0.43049	[redacted]	0.385575
chr16	51650850	51650870	0.430655	[redacted]	0.440607
chr10	59917050	59917108	0.43148	[redacted]	0.503053
chr17	5249068	5249148	0.431754	[redacted]	0.48885
chr4	125168474	125168492	0.431898	[redacted]	0.434836
chr2	158305860	158305919	0.432476	[redacted]	0.41809
chr11	32580899	32580963	0.432479	[redacted]	0.512945
chr9	40346199	40346226	0.433586	[redacted]	0.499251
chr13	35783904	35783929	0.434899	[redacted]	0.55755
chr15	81416880	81416907	0.435469	[redacted]	0.660997
chr15	39853488	39853513	0.435637	[redacted]	0.567011
chr6	128754394	128754459	0.435939	[redacted]	0.486785
chr7	24300291	24300338	0.437876	[redacted]	0.561598
chr15	38202001	38202057	0.438742	[redacted]	0.425245
chr6	97182255	97182272	0.438755	[redacted]	0.514518
chr7	38183346	38183356	0.439146	[redacted]	0.393345
chr19	47508694	47508717	0.439644	[redacted]	0.353742
chr12	110908465	110908513	0.439807	[redacted]	0.531613
chr6	34864072	34864134	0.441256	[redacted]	0.426947
chr3	98046391	98046469	0.441415	[redacted]	0.425663
chr11	101263534	101263552	0.441858	[redacted]	0.325351
chr3	146421913	146421946	0.442605	[redacted]	0.330678
chr5	100717016	100717044	0.442615	[redacted]	0.457686
chr2	126490793	126490838	0.443969	[redacted]	0.392492
chr2	155663276	155663344	0.444414	[redacted]	0.502158
chr8	120505863	120505913	0.444652	[redacted]	0.559033
chr15	76802263	76802300	0.444688	[redacted]	0.444815
chr9	48661328	48661399	0.4451	[redacted]	0.527131
chr15	95880982	95881023	0.446076	[redacted]	0.354727
chr15	82028367	82028445	0.446266	[redacted]	0.601909
chr13	44980568	44980660	0.446657	[redacted]	0.480709
chr7	30143906	30143929	0.447731	[redacted]	0.667341
chr17	66108195	66108242	0.44898	[redacted]	0.475757
chr4	103039673	103039848	0.450803	[redacted]	0.41949
chr2	62460592	62460617	0.452006	[redacted]	0.386423

chr3	132932261	132932285	0.452037	[redacted]	0.380484
chr6	34626658	34626703	0.454311	[redacted]	0.308956
chr10	44427617	44427674	0.454613	[redacted]	0.424161
chr10	37333803	37333839	0.455235	[redacted]	0.420146
chr2	19158322	19158360	0.456029	[redacted]	0.321397
chr5	118563686	118563704	0.458011	[redacted]	0.540886
chr16	87844307	87844383	0.458264	[redacted]	0.533547
chr9	78071099	78071168	0.458368	[redacted]	0.465411
chr10	13428814	13428929	0.458948	[redacted]	0.569224
chr3	90446291	90446346	0.460874	[redacted]	0.508752
chr11	79519566	79519587	0.460936	[redacted]	0.446293
chr7	112841852	112841877	0.461196	[redacted]	0.539777
chr12	73901382	73901403	0.462296	[redacted]	0.360855
chr6	34086850	34086956	0.462478	[redacted]	0.433448
chrX	71215813	71215848	0.462871	[redacted]	0.558496
chr14	26879725	26879790	0.463307	[redacted]	0.621866
chr14	78731649	78731703	0.464982	[redacted]	0.470451
chr15	73017730	73017856	0.465188	[redacted]	0.497567
chr12	51976530	51976599	0.4653	[redacted]	0.496087
chr2	10167238	10167287	0.46756	[redacted]	0.548843
chr1	13573977	13574042	0.467656	[redacted]	0.542815
chr2	128008855	128009048	0.467676	[redacted]	0.371797
chr17	80004557	80004572	0.467929	[redacted]	0.554096
chr10	117906098	117906169	0.468218	[redacted]	0.43049
chr10	17684002	17684078	0.470407	[redacted]	0.457838
chr17	88119042	88119090	0.471057	[redacted]	0.538336
chr16	20496933	20497161	0.471436	[redacted]	0.422119
chr8	120720101	120720179	0.474025	[redacted]	0.397905
chr4	138172576	138172643	0.475966	[redacted]	0.435887
chr1	59253329	59253407	0.47615	[redacted]	0.379476
chr6	99435455	99435485	0.476977	[redacted]	0.441005
chr8	120752333	120752378	0.478117	[redacted]	0.409679
chr11	32012446	32012489	0.481386	[redacted]	0.504626
chr19	31602931	31602942	0.481449	[redacted]	0.333644
chr17	51203513	51203527	0.482424	[redacted]	0.487633
chr4	62494507	62494563	0.482666	[redacted]	0.437606
chr13	30901613	30901645	0.483499	[redacted]	0.564356
chr9	50452162	50452226	0.483853	[redacted]	0.360646
chr11	78825915	78825977	0.484434	[redacted]	0.41676
chr6	42354127	42354164	0.486494	[redacted]	0.372033
chr18	33408636	33408652	0.489385	[redacted]	0.673136
chr4	135271583	135271635	0.489949	[redacted]	0.470983
chr2	122150458	122150566	0.490545	[redacted]	0.464184

chr13	93354965	93354990	0.491481	[redacted]	0.30607
chr9	60687389	60687436	0.492231	[redacted]	0.381588
chr13	28781840	28781888	0.49336	[redacted]	0.467591
chr7	80652834	80652851	0.494712	[redacted]	0.522947
chr9	78071532	78071571	0.495372	[redacted]	0.511531
chr10	128646254	128646265	0.496271	[redacted]	0.600382
chr13	97462468	97462520	0.497063	[redacted]	0.323235
chr6	51012559	51012585	0.497452	[redacted]	0.606398
chr9	61924991	61925053	0.497734	[redacted]	0.427854
chr2	73069287	73069317	0.497953	[redacted]	0.447795
chr6	118779282	118779337	0.498338	[redacted]	0.320259
chr2	160780270	160780315	0.499052	[redacted]	0.448874
chr8	46472244	46472307	0.500552	[redacted]	0.46794
chr17	90698294	90698377	0.500614	[redacted]	0.600811
chr15	84208601	84208615	0.501389	[redacted]	0.481111
chrX	134140200	134140290	0.501845	[redacted]	0.355513
chr12	66095298	66095342	0.504493	[redacted]	0.444054
chr13	112751947	112752022	0.504967	[redacted]	0.345159
chr6	134932758	134932846	0.506405	[redacted]	0.538258
chr3	65873479	65873516	0.506744	[redacted]	0.552677
chr1	180132383	180132419	0.508118	[redacted]	0.343499
chr1	51036455	51036472	0.510766	[redacted]	0.550064
chr4	103170799	103170836	0.511644	[redacted]	0.450935
chr16	94556224	94556270	0.514457	[redacted]	0.470449
chr1	52471104	52471133	0.515261	[redacted]	0.559078
chr16	44758298	44758328	0.515436	[redacted]	0.427658
chr5	148968460	148968476	0.515527	[redacted]	0.563216
chr7	114081917	114081960	0.516019	[redacted]	0.493113
chr10	40131513	40131628	0.516218	[redacted]	0.646766
chr10	79321950	79321963	0.516492	[redacted]	0.609098
chr1	33918627	33918684	0.517613	[redacted]	0.55223
chr3	147060933	147060965	0.518514	[redacted]	0.42648
chr15	86363880	86363947	0.520439	[redacted]	0.558573
chr11	100571488	100571500	0.520484	[redacted]	0.517291
chr16	76538024	76538061	0.522657	[redacted]	0.470168
chr1	92127907	92127982	0.522714	[redacted]	0.653551
chr2	6363365	6363445	0.528199	[redacted]	0.49236
chr5	44217831	44217925	0.528695	[redacted]	0.620488
chr2	4561648	4561678	0.529491	[redacted]	0.516462
chr19	46343724	46343766	0.529993	[redacted]	0.323225
chr5	92394239	92394288	0.535208	[redacted]	0.351554
chr4	133162238	133162318	0.53819	[redacted]	0.739689
chr9	51952965	51953043	0.545143	[redacted]	0.528558

chr4	133906840	133906856	0.547533		0.68786
chr17	87181371	87181401	0.548708		0.592038
chr14	54351458	54351501	0.549371		0.531083
chr10	127104097	127104117	0.551734		0.656783
chr14	61757840	61757886	0.552161		0.60976
chr10	115505442	115505458	0.554213		0.480383
chr9	68753493	68753518	0.556959		0.423988
chr5	107701212	107701290	0.558462		0.51548
chr4	138913204	138913238	0.558573		0.494576
chr13	93355358	93355407	0.558843		0.654398
chr16	33892063	33892081	0.564916		0.482841
chr5	124537022	124537029	0.571296		0.51491
chr16	76532677	76532709	0.571759		0.551701
chr5	64342311	64342384	0.575027		0.358564
chr13	103984945	103984990	0.576617		0.604369
chr16	19884763	19884877	0.580698		0.666642
chr11	6008158	6008175	0.583186		0.642903
chr4	134165190	134165260	0.587922		0.567505
chr9	37567984	37568026	0.597864		0.503336
chr10	25312082	25312126	0.598591		0.650443
chr11	87245339	87245462	0.598866		0.702345
chr8	25017993	25018012	0.599529		0.677258
chr11	116265463	116265477	0.605348		0.585834
chr10	4080173	4080244	0.61226		0.505062
chr10	93257481	93257531	0.615487		0.567703
chr10	4080058	4080101	0.621196		0.409708
chr2	146051205	146051224	0.622664		0.583511
chr4	130148566	130148577	0.623581		0.514817
chr4	103122908	103123011	0.626848		0.638353
chrX	167359716	167359737	0.626972		0.557019
chr9	61925468	61925626	0.63132		0.62815
chr7	46059210	46059250	0.634086		0.672876
chr2	76219900	76219916	0.651651		0.55937
chr10	25404239	25404336	0.659158		0.626819
chr5	92205216	92205247	0.680263		0.678279
chr12	102674931	102674954	0.699518		0.594415
chr10	25404082	25404120	0.724156		0.771997
chr19	6895739	6895753	0.775933		0.772012
chr19	5406420	5406545	0.848867		0.833331
chr5	112082044	112082101		-0.758475	-0.818661
chr7	118395066	118395085		-0.712232	-0.438026
chr7	82814110	82814146		-0.697107	-0.685699
chr1	170210725	170210781		-0.692175	-0.661595

chr2	69569240	69569307	[redacted]	-0.692079	-0.635054
chr13	102543701	102543740	[redacted]	-0.688458	-0.704772
chr14	88160087	88160147	[redacted]	-0.687883	-0.653084
chr3	36353379	36353420	[redacted]	-0.679663	-0.612203
chr13	118909998	118910029	[redacted]	-0.676523	-0.654194
chr1	133533188	133533277	[redacted]	-0.669321	-0.646317
chr13	95952814	95952850	[redacted]	-0.668951	-0.677549
chr9	47045476	47045541	[redacted]	-0.667131	-0.616959
chr6	143363121	143363168	[redacted]	-0.666412	-0.645116
chr19	22331611	22331690	[redacted]	-0.663626	-0.685722
chr18	39856701	39856724	[redacted]	-0.659896	-0.688652
chr9	67337512	67337562	[redacted]	-0.656425	-0.646505
chr8	90475630	90475696	[redacted]	-0.655924	-0.729998
chr1	66172559	66172607	[redacted]	-0.655492	-0.601861
chr9	64321596	64321627	[redacted]	-0.655097	-0.660632
chr17	84930918	84930933	[redacted]	-0.652632	-0.690914
chr8	66892647	66892734	[redacted]	-0.650795	-0.717975
chr17	24280477	24280505	[redacted]	-0.650045	-0.719733
chr2	80868267	80868278	[redacted]	-0.645924	-0.655414
chr2	87784160	87784203	[redacted]	-0.645044	-0.653137
chr15	84563307	84563396	[redacted]	-0.643094	-0.599439
chr11	6900430	6900490	[redacted]	-0.639362	-0.685051
chr10	86735034	86735089	[redacted]	-0.638089	-0.439952
chr7	37385446	37385514	[redacted]	-0.637446	-0.673809
chr12	113110070	113110108	[redacted]	-0.637161	-0.356816
chr8	91610745	91610797	[redacted]	-0.631984	-0.346376
chr14	112478059	112478121	[redacted]	-0.630508	-0.643062
chr2	160489969	160490007	[redacted]	-0.630312	-0.300271
chr5	151110637	151110712	[redacted]	-0.629813	-0.605981
chr11	24152382	24152422	[redacted]	-0.629263	-0.459541
chr17	25303754	25303804	[redacted]	-0.626993	-0.642164
chr17	46498142	46498211	[redacted]	-0.626017	-0.300382
chr6	73194746	73194810	[redacted]	-0.625808	-0.629352
chr14	52538794	52538877	[redacted]	-0.623242	-0.532249
chr5	136530032	136530086	[redacted]	-0.623215	-0.377565
chr12	18247516	18247532	[redacted]	-0.622893	-0.654038
chr5	141820499	141820546	[redacted]	-0.618895	-0.601335
chr3	49096260	49096336	[redacted]	-0.618847	-0.68933
chr14	88926233	88926272	[redacted]	-0.617322	-0.650655
chr1	42419790	42419825	[redacted]	-0.614728	-0.457288
chr3	144412116	144412183	[redacted]	-0.613384	-0.622933
chr1	189069090	189069137	[redacted]	-0.610736	-0.319428
chr11	58222347	58222381	[redacted]	-0.60846	-0.417686

chr17	7717243	7717282	[redacted]	-0.606826	-0.392972
chr11	84895988	84896060	[redacted]	-0.606093	-0.308595
chr8	43161560	43161590	[redacted]	-0.603754	-0.568011
chr5	49481626	49481682	[redacted]	-0.601861	-0.599573
chr8	29954450	29954472	[redacted]	-0.601768	-0.636613
chr2	159591241	159591298	[redacted]	-0.601715	-0.401919
chr7	27133503	27133586	[redacted]	-0.601153	-0.379257
chr15	45510391	45510444	[redacted]	-0.600814	-0.613956
chr2	128472471	128472510	[redacted]	-0.600337	-0.537685
chr18	68741951	68742043	[redacted]	-0.600275	-0.522633
chr16	50107365	50107431	[redacted]	-0.599209	-0.602688
chr9	24328577	24328612	[redacted]	-0.594284	-0.539408
chr2	27635478	27635513	[redacted]	-0.593132	-0.336024
chr5	104887626	104887679	[redacted]	-0.592154	-0.515765
chr6	58624818	58624857	[redacted]	-0.591315	-0.414747
chr9	30319241	30319275	[redacted]	-0.587785	-0.330761
chr9	64165296	64165375	[redacted]	-0.587295	-0.443979
chr9	34575314	34575348	[redacted]	-0.58727	-0.492156
chr6	148417786	148417911	[redacted]	-0.585692	-0.44085
chr11	59538102	59538141	[redacted]	-0.5834	-0.491473
chr16	30302940	30302996	[redacted]	-0.582242	-0.452862
chr3	115731072	115731152	[redacted]	-0.581818	-0.336869
chr4	59505505	59505567	[redacted]	-0.58177	-0.308029
chr14	105474695	105474735	[redacted]	-0.581273	-0.425014
chr2	65818845	65818883	[redacted]	-0.58079	-0.355047
chr12	29254135	29254179	[redacted]	-0.579912	-0.554157
chr18	67761553	67761607	[redacted]	-0.579751	-0.378707
chr3	19560779	19560826	[redacted]	-0.577223	-0.364821
chr8	37887793	37887881	[redacted]	-0.57608	-0.610784
chr2	3876941	3876978	[redacted]	-0.574018	-0.387949
chrX	163045694	163045764	[redacted]	-0.572594	-0.389975
chr3	70333125	70333189	[redacted]	-0.572309	-0.518553
chr3	50081942	50081992	[redacted]	-0.571543	-0.661897
chr8	23752445	23752507	[redacted]	-0.568941	-0.425669
chr13	115428736	115428782	[redacted]	-0.565066	-0.529516
chr1	6455171	6455262	[redacted]	-0.564212	-0.35181
chr14	28161803	28161847	[redacted]	-0.563527	-0.459895
chr5	108732809	108732861	[redacted]	-0.562669	-0.51894
chr1	184048149	184048225	[redacted]	-0.562326	-0.372734
chr3	121619316	121619349	[redacted]	-0.560913	-0.426057
chr18	20585136	20585179	[redacted]	-0.56076	-0.395964
chr19	48534960	48534981	[redacted]	-0.559751	-0.365724
chr10	41068796	41068876	[redacted]	-0.559524	-0.34393

chr9	89759489	89759524	[redacted]	-0.55881	-0.555092
chr19	59505185	59505240	[redacted]	-0.557962	-0.459142
chr5	56801833	56801896	[redacted]	-0.557874	-0.421686
chr15	86949939	86950018	[redacted]	-0.556687	-0.323219
chr2	72036158	72036252	[redacted]	-0.555479	-0.361213
chr11	90015914	90015945	[redacted]	-0.554405	-0.539699
chr4	124074634	124074675	[redacted]	-0.553656	-0.424532
chr3	147483895	147483933	[redacted]	-0.553601	-0.424288
chr9	99506103	99506156	[redacted]	-0.552571	-0.364499
chr12	72253904	72253990	[redacted]	-0.552049	-0.491375
chr9	66296338	66296367	[redacted]	-0.552027	-0.629344
chr1	81250161	81250191	[redacted]	-0.551743	-0.311837
chr18	79692035	79692088	[redacted]	-0.549445	-0.566962
chr6	23635669	23635721	[redacted]	-0.548752	-0.406501
chr12	27934672	27934751	[redacted]	-0.548746	-0.430396
chr18	89505292	89505359	[redacted]	-0.548605	-0.565724
chr1	119887880	119887923	[redacted]	-0.548063	-0.51473
chr9	43150546	43150589	[redacted]	-0.544691	-0.356169
chr2	33280088	33280149	[redacted]	-0.544538	-0.334681
chr12	72103762	72103785	[redacted]	-0.544025	-0.404118
chr1	75008822	75008857	[redacted]	-0.542355	-0.317312
chr10	126863353	126863409	[redacted]	-0.542271	-0.557496
chr9	8717060	8717129	[redacted]	-0.54154	-0.579828
chr5	55439578	55439662	[redacted]	-0.541182	-0.549796
chr7	78128910	78128989	[redacted]	-0.541022	-0.566994
chr11	108845624	108845674	[redacted]	-0.538466	-0.370658
chr13	114005504	114005553	[redacted]	-0.537176	-0.411104
chr6	127438196	127438234	[redacted]	-0.536695	-0.398616
chr8	38981933	38981996	[redacted]	-0.536623	-0.472093
chr13	13454305	13454342	[redacted]	-0.536172	-0.485526
chr5	81749727	81749786	[redacted]	-0.535785	-0.453019
chr16	58687730	58687826	[redacted]	-0.534468	-0.400244
chr4	151251490	151251499	[redacted]	-0.534136	-0.387692
chr7	41580100	41580171	[redacted]	-0.533595	-0.381506
chr18	73624214	73624260	[redacted]	-0.532312	-0.47921
chr9	26133121	26133159	[redacted]	-0.531738	-0.48721
chr5	127237397	127237457	[redacted]	-0.531612	-0.548973
chr3	65188855	65188907	[redacted]	-0.53107	-0.395126
chr19	57328493	57328517	[redacted]	-0.529744	-0.405058
chr1	193777979	193778019	[redacted]	-0.529728	-0.418407
chr7	34677611	34677671	[redacted]	-0.529622	-0.314711
chrX	74154891	74154944	[redacted]	-0.529285	-0.473086
chr9	119204313	119204364	[redacted]	-0.529129	-0.425837

chr9	111431494	111431532	[redacted]	-0.528577	-0.4107
chr1	137324353	137324387	[redacted]	-0.528538	-0.452439
chr2	71827743	71827819	[redacted]	-0.527991	-0.511885
chr3	81815805	81815887	[redacted]	-0.527447	-0.371544
chr7	51859615	51859666	[redacted]	-0.526686	-0.371858
chrX	42785641	42785716	[redacted]	-0.526398	-0.375917
chr6	40610562	40610619	[redacted]	-0.526275	-0.445396
chr12	40796624	40796702	[redacted]	-0.525995	-0.378196
chrX	50925245	50925305	[redacted]	-0.525896	-0.46748
chr2	69529567	69529632	[redacted]	-0.52515	-0.484252
chr14	115611065	115611152	[redacted]	-0.524797	-0.538237
chr10	70138298	70138361	[redacted]	-0.52457	-0.509965
chr2	70400360	70400462	[redacted]	-0.523678	-0.529053
chr17	73788148	73788191	[redacted]	-0.522568	-0.455296
chr17	71160395	71160451	[redacted]	-0.521614	-0.510965
chr5	109849516	109849572	[redacted]	-0.521413	-0.374892
chr11	50684913	50684987	[redacted]	-0.521338	-0.401773
chr17	34105021	34105055	[redacted]	-0.521003	-0.407863
chr12	26331132	26331178	[redacted]	-0.520924	-0.453468
chr16	50392775	50392838	[redacted]	-0.520834	-0.346247
chr12	18303762	18303802	[redacted]	-0.520704	-0.50176
chr2	52509728	52509742	[redacted]	-0.520165	-0.497848
chr9	115600725	115600758	[redacted]	-0.519735	-0.472419
chr5	146624213	146624291	[redacted]	-0.519316	-0.381675
chr16	15838488	15838548	[redacted]	-0.519143	-0.460046
chr12	52052627	52052695	[redacted]	-0.517045	-0.305634
chr10	88726980	88727028	[redacted]	-0.516444	-0.324497
chr6	137614359	137614406	[redacted]	-0.516371	-0.488629
chr16	95259201	95259249	[redacted]	-0.516322	-0.303388
chr2	79237445	79237497	[redacted]	-0.51628	-0.345746
chr12	53597020	53597099	[redacted]	-0.516124	-0.506916
chr18	16474448	16474485	[redacted]	-0.516002	-0.586439
chr12	116075536	116075607	[redacted]	-0.515088	-0.383826
chr8	33195347	33195435	[redacted]	-0.514832	-0.524228
chr7	37361531	37361587	[redacted]	-0.514781	-0.398125
chr1	106900999	106901110	[redacted]	-0.514537	-0.439178
chr3	121788134	121788170	[redacted]	-0.51428	-0.399051
chr13	85854282	85854340	[redacted]	-0.514007	-0.481602
chr6	52649501	52649538	[redacted]	-0.513172	-0.408766
chr1	150008606	150008655	[redacted]	-0.513037	-0.515306
chr18	79053470	79053529	[redacted]	-0.512544	-0.459077
chr19	26237131	26237252	[redacted]	-0.512387	-0.390266
chr3	156265527	156265576	[redacted]	-0.511789	-0.401436

chr3	49414947	49414993	[redacted]	-0.511678	-0.589858
chr5	49410607	49410646	[redacted]	-0.511609	-0.663093
chr19	19673085	19673140	[redacted]	-0.510478	-0.429922
chrX	36590407	36590483	[redacted]	-0.510313	-0.307824
chr3	50338841	50338884	[redacted]	-0.510103	-0.387641
chr10	39446116	39446177	[redacted]	-0.510066	-0.304511
chr2	89265100	89265140	[redacted]	-0.50956	-0.434113
chr18	66274229	66274295	[redacted]	-0.509488	-0.373575
chr13	16171234	16171313	[redacted]	-0.50922	-0.53739
chr16	39032151	39032238	[redacted]	-0.509069	-0.370228
chr1	185494765	185494839	[redacted]	-0.508597	-0.337776
chr6	53435953	53436016	[redacted]	-0.507756	-0.341988
chr6	31969879	31969924	[redacted]	-0.507627	-0.311071
chr1	132982723	132982751	[redacted]	-0.507383	-0.400921
chr12	115495107	115495144	[redacted]	-0.507033	-0.639249
chr1	185474860	185474907	[redacted]	-0.506792	-0.35677
chr9	98874879	98874933	[redacted]	-0.505833	-0.370598
chr1	40922538	40922624	[redacted]	-0.505628	-0.453185
chr15	13264888	13264959	[redacted]	-0.505594	-0.302321
chr2	14592660	14592733	[redacted]	-0.505024	-0.536455
chr16	91542132	91542170	[redacted]	-0.504734	-0.370093
chr2	65557522	65557608	[redacted]	-0.504433	-0.389909
chr15	74043747	74043774	[redacted]	-0.504013	-0.460046
chr2	104670436	104670479	[redacted]	-0.503619	-0.512691
chr16	89196604	89196668	[redacted]	-0.503312	-0.335668
chr19	31517608	31517666	[redacted]	-0.502405	-0.389799
chr2	71104471	71104487	[redacted]	-0.501944	-0.398057
chr9	51205632	51205692	[redacted]	-0.501376	-0.40878
chr1	35870245	35870311	[redacted]	-0.500718	-0.581682
chr2	4699385	4699432	[redacted]	-0.500407	-0.347387
chr8	124763047	124763062	[redacted]	-0.500223	-0.482494
chr3	59149517	59149564	[redacted]	-0.500066	-0.711992
chr13	81991292	81991356	[redacted]	-0.499868	-0.371007
chr4	151064990	151065017	[redacted]	-0.49959	-0.404574
chr9	9960007	9960049	[redacted]	-0.499142	-0.51538
chr12	74803875	74803934	[redacted]	-0.499108	-0.491009
chr4	54998358	54998430	[redacted]	-0.49891	-0.616966
chr8	108928566	108928600	[redacted]	-0.498349	-0.414292
chr3	142948835	142948849	[redacted]	-0.498251	-0.555753
chr16	44463500	44463568	[redacted]	-0.49815	-0.365531
chr2	131533825	131533836	[redacted]	-0.497617	-0.34586
chr11	88110352	88110395	[redacted]	-0.496247	-0.307258
chr6	137277090	137277157	[redacted]	-0.496128	-0.467813

chr4	132655165	132655246	[redacted]	-0.495358	-0.307364
chr10	87836584	87836648	[redacted]	-0.495347	-0.597662
chr1	85575782	85575833	[redacted]	-0.495274	-0.368144
chr15	20867886	20867916	[redacted]	-0.495175	-0.411178
chr5	139928607	139928643	[redacted]	-0.494353	-0.407211
chr5	74545502	74545572	[redacted]	-0.493905	-0.306883
chr10	40775398	40775466	[redacted]	-0.49276	-0.326223
chr8	91397361	91397421	[redacted]	-0.492577	-0.426755
chr11	29181508	29181565	[redacted]	-0.4925	-0.372824
chr14	61778512	61778575	[redacted]	-0.492155	-0.361148
chr5	148212474	148212515	[redacted]	-0.491935	-0.439637
chr6	35476480	35476544	[redacted]	-0.491451	-0.402928
chr13	69832647	69832654	[redacted]	-0.490454	-0.412084
chr2	14178425	14178508	[redacted]	-0.490389	-0.32376
chr15	11460377	11460409	[redacted]	-0.49003	-0.431099
chr1	148664371	148664430	[redacted]	-0.489901	-0.49813
chr12	66540129	66540209	[redacted]	-0.489747	-0.537119
chr15	81730687	81730724	[redacted]	-0.489544	-0.517619
chr15	81308280	81308351	[redacted]	-0.489364	-0.419457
chr1	179108432	179108466	[redacted]	-0.489251	-0.487023
chr13	45790645	45790741	[redacted]	-0.488906	-0.333642
chrX	106377769	106377840	[redacted]	-0.488763	-0.320916
chr18	86416585	86416634	[redacted]	-0.48853	-0.412531
chr9	66971144	66971209	[redacted]	-0.488379	-0.370242
chr7	113438022	113438111	[redacted]	-0.488238	-0.370167
chrX	33856352	33856406	[redacted]	-0.488189	-0.387209
chr16	87530169	87530219	[redacted]	-0.488155	-0.560622
chr4	150835748	150835762	[redacted]	-0.487831	-0.303596
chr11	56172036	56172079	[redacted]	-0.48781	-0.433893
chr3	79873408	79873456	[redacted]	-0.487547	-0.411537
chr1	164094546	164094609	[redacted]	-0.487527	-0.465205
chr9	115572224	115572243	[redacted]	-0.487003	-0.407777
chr11	57512476	57512546	[redacted]	-0.486386	-0.328886
chr18	62840904	62840986	[redacted]	-0.485907	-0.316218
chr5	57738793	57738862	[redacted]	-0.485748	-0.534634
chr3	143378721	143378781	[redacted]	-0.485254	-0.465493
chr9	19110200	19110249	[redacted]	-0.485182	-0.510002
chr2	18074631	18074676	[redacted]	-0.484646	-0.311026
chr1	62636446	62636469	[redacted]	-0.484388	-0.373109
chr2	115514392	115514424	[redacted]	-0.48432	-0.547657
chr6	12489414	12489486	[redacted]	-0.484259	-0.438675
chr19	31200758	31200819	[redacted]	-0.484011	-0.449202
chr2	96213356	96213408	[redacted]	-0.483885	-0.56807

chr8	27014370	27014418		-0.483672	-0.363036
chr9	55004828	55004887		-0.483449	-0.372577
chr14	95625285	95625329		-0.483052	-0.47672
chr1	14808719	14808778		-0.482791	-0.384979
chr9	26945138	26945160		-0.482356	-0.408448
chr19	45350840	45350901		-0.482242	-0.438479
chr3	19514608	19514652		-0.48209	-0.509481
chr11	36068930	36069044		-0.481445	-0.393867
chr8	101917129	101917199		-0.481075	-0.381391
chr2	152491310	152491350		-0.480782	-0.427447
chr17	14018316	14018366		-0.480745	-0.487586
chr14	47975054	47975122		-0.480546	-0.474315
chr3	127913870	127913945		-0.480537	-0.373408
chr2	115817986	115818057		-0.479976	-0.470429
chr5	53678169	53678220		-0.479922	-0.3854
chr8	40875772	40875826		-0.479676	-0.433287
chr12	35764969	35765047		-0.479392	-0.346799
chr17	10732526	10732550		-0.479029	-0.314527
chr15	80268773	80268849		-0.479012	-0.433536
chr8	7775792	7775849		-0.47899	-0.330439
chr8	112226008	112226127		-0.478634	-0.350795
chr1	38260407	38260474		-0.47821	-0.383182
chr2	70158872	70158913		-0.478176	-0.428501
chr2	80724176	80724204		-0.478045	-0.569566
chr3	94848725	94848787		-0.477913	-0.36023
chr7	118257067	118257219		-0.476973	-0.377834
chr9	86034187	86034199		-0.476956	-0.441245
chr3	156656849	156656971		-0.476933	-0.444399
chr12	26955357	26955391		-0.476818	-0.464609
chr6	70735187	70735255		-0.476608	-0.345825
chr4	155277621	155277666		-0.476512	-0.358727
chr10	101336265	101336354		-0.475751	-0.390571
chr15	39785686	39785758		-0.475346	-0.556226
chr13	8291445	8291525		-0.475324	-0.351487
chr2	58584113	58584163		-0.475324	-0.325628
chr13	111436447	111436489		-0.475298	-0.311554
chr18	31937914	31937968		-0.474944	-0.454462
chr10	52981843	52981884		-0.474611	-0.305397
chr18	43815235	43815271		-0.474546	-0.399777
chr2	168956750	168956803		-0.474333	-0.471079
chr12	112084778	112084836		-0.474268	-0.404157
chr1	173971909	173971931		-0.474256	-0.453762
chr6	6813351	6813429		-0.474242	-0.504919

chr3	115727915	115727949	[redacted]	-0.473924	-0.394722
chr16	70199238	70199312	[redacted]	-0.473708	-0.38919
chr9	77787527	77787566	[redacted]	-0.4735	-0.34029
chr10	127138668	127138719	[redacted]	-0.47343	-0.449027
chr19	21547852	21547914	[redacted]	-0.473378	-0.378411
chr3	63977565	63977633	[redacted]	-0.473271	-0.396927
chr1	157674424	157674466	[redacted]	-0.472772	-0.376368
chrX	37970698	37970765	[redacted]	-0.472106	-0.37156
chr14	93235396	93235460	[redacted]	-0.471802	-0.425125
chr11	44329888	44329922	[redacted]	-0.47141	-0.374202
chr17	3905338	3905413	[redacted]	-0.471351	-0.371707
chr13	18938860	18938911	[redacted]	-0.471104	-0.300519
chr3	30488183	30488231	[redacted]	-0.470565	-0.380864
chr14	77827405	77827430	[redacted]	-0.470192	-0.375032
chr3	139576661	139576717	[redacted]	-0.470082	-0.381029
chr17	41323655	41323718	[redacted]	-0.469934	-0.422981
chr6	37754776	37754813	[redacted]	-0.46954	-0.316477
chr8	47372488	47372532	[redacted]	-0.469354	-0.644707
chr5	92328535	92328586	[redacted]	-0.469228	-0.303794
chr6	113031546	113031611	[redacted]	-0.469053	-0.344212
chr7	135268586	135268627	[redacted]	-0.468977	-0.427987
chr7	83548834	83548916	[redacted]	-0.468307	-0.415862
chr9	107552391	107552455	[redacted]	-0.468149	-0.386324
chr3	87421998	87422064	[redacted]	-0.468133	-0.408195
chr9	81238896	81238944	[redacted]	-0.467997	-0.379587
chr5	147055277	147055336	[redacted]	-0.467891	-0.347521
chr8	14771851	14771914	[redacted]	-0.467853	-0.341188
chr5	62982516	62982605	[redacted]	-0.467759	-0.468602
chrX	72826520	72826576	[redacted]	-0.467702	-0.435681
chr1	158629476	158629505	[redacted]	-0.467683	-0.483782
chr10	56156483	56156555	[redacted]	-0.467139	-0.337775
chr11	70070161	70070200	[redacted]	-0.467066	-0.401088
chr6	131717306	131717406	[redacted]	-0.466415	-0.311721
chr9	76245294	76245348	[redacted]	-0.466085	-0.407636
chr15	51305395	51305447	[redacted]	-0.46605	-0.321991
chr5	77252109	77252183	[redacted]	-0.465882	-0.311643
chr17	20353797	20353873	[redacted]	-0.465842	-0.424631
chr14	108684575	108684606	[redacted]	-0.465787	-0.494604
chr16	51447671	51447714	[redacted]	-0.465501	-0.414937
chr3	117792683	117792750	[redacted]	-0.465075	-0.428319
chr12	45924765	45924850	[redacted]	-0.464914	-0.395319
chr11	55185049	55185107	[redacted]	-0.464889	-0.372128
chr17	55402870	55402897	[redacted]	-0.464158	-0.462993

chr12	72392844	72392888	[redacted]	-0.464012	-0.415223
chr14	66272253	66272259	[redacted]	-0.464002	-0.370729
chr12	75047368	75047421	[redacted]	-0.464001	-0.434906
chr1	77528948	77528983	[redacted]	-0.463807	-0.434563
chr10	115933940	115934012	[redacted]	-0.463745	-0.389439
chr13	85231755	85231792	[redacted]	-0.463738	-0.452053
chr9	21700922	21700944	[redacted]	-0.463472	-0.365234
chr12	34587638	34587709	[redacted]	-0.463459	-0.424855
chr11	70271914	70271999	[redacted]	-0.463447	-0.30243
chr1	88910024	88910070	[redacted]	-0.46338	-0.380514
chr10	53802026	53802137	[redacted]	-0.463324	-0.310601
chr14	29630761	29630809	[redacted]	-0.463068	-0.31917
chr1	8509804	8509854	[redacted]	-0.462739	-0.372119
chr15	77720318	77720326	[redacted]	-0.462687	-0.362159
chr7	101608748	101608772	[redacted]	-0.462406	-0.435095
chr9	30888663	30888696	[redacted]	-0.462046	-0.367326
chr4	102862573	102862610	[redacted]	-0.461985	-0.319983
chr7	82816323	82816394	[redacted]	-0.461786	-0.365757
chr14	93613910	93613956	[redacted]	-0.461669	-0.379843
chr6	67819575	67819632	[redacted]	-0.461604	-0.30364
chrX	121918438	121918468	[redacted]	-0.461499	-0.470051
chr18	88545155	88545222	[redacted]	-0.461499	-0.389453
chr2	68363799	68363851	[redacted]	-0.46102	-0.574142
chr10	93831546	93831695	[redacted]	-0.460967	-0.796063
chr1	121244285	121244325	[redacted]	-0.46059	-0.381301
chr16	40735469	40735552	[redacted]	-0.460584	-0.448619
chr10	67890522	67890628	[redacted]	-0.460298	-0.344093
chr5	144975603	144975645	[redacted]	-0.460176	-0.481499
chr11	102449863	102449935	[redacted]	-0.460002	-0.347366
chr9	38919988	38920040	[redacted]	-0.459989	-0.407655
chr1	135935970	135935997	[redacted]	-0.459939	-0.323692
chrX	106442379	106442420	[redacted]	-0.459746	-0.30181
chr6	106750421	106750469	[redacted]	-0.45972	-0.432304
chr6	93730450	93730473	[redacted]	-0.459546	-0.427562
chr3	96540150	96540232	[redacted]	-0.4595	-0.484481
chr7	137778412	137778469	[redacted]	-0.458991	-0.301115
chr9	91672356	91672419	[redacted]	-0.458927	-0.469565
chr8	39353575	39353618	[redacted]	-0.458843	-0.491662
chr14	100159055	100159115	[redacted]	-0.458549	-0.434276
chr12	26791942	26792010	[redacted]	-0.458477	-0.429424
chr9	56234160	56234196	[redacted]	-0.458436	-0.407176
chr5	144325942	144326002	[redacted]	-0.45839	-0.574496
chr6	4486298	4486339	[redacted]	-0.458308	-0.495437

chr10	88701148	88701166	[redacted]	-0.458264	-0.359642
chr5	60528258	60528300	[redacted]	-0.458221	-0.473566
chr1	66321954	66322218	[redacted]	-0.458165	-0.474992
chr10	111684819	111684894	[redacted]	-0.458074	-0.300689
chr12	26637089	26637147	[redacted]	-0.458053	-0.36804
chr12	110435044	110435093	[redacted]	-0.457821	-0.318449
chr10	58697883	58697967	[redacted]	-0.457661	-0.367582
chr6	82973205	82973281	[redacted]	-0.457165	-0.353214
chr4	43303878	43303947	[redacted]	-0.456757	-0.538382
chr5	144991460	144991506	[redacted]	-0.45656	-0.310536
chr6	114385449	114385496	[redacted]	-0.456551	-0.38906
chr8	57245410	57245484	[redacted]	-0.45633	-0.576634
chr17	14727959	14728013	[redacted]	-0.456271	-0.421881
chr1	95024133	95024188	[redacted]	-0.456219	-0.500289
chr18	67112441	67112507	[redacted]	-0.456056	-0.305072
chr9	31560299	31560323	[redacted]	-0.455769	-0.316989
chr7	75096070	75096154	[redacted]	-0.455731	-0.392876
chr17	4922447	4922560	[redacted]	-0.455617	-0.374442
chr7	7618567	7618666	[redacted]	-0.455479	-0.498566
chr16	80630311	80630348	[redacted]	-0.455208	-0.487994
chr8	75732018	75732081	[redacted]	-0.454769	-0.467985
chr2	30471066	30471081	[redacted]	-0.454717	-0.306869
chr11	40616383	40616452	[redacted]	-0.454341	-0.301393
chr7	19734920	19734982	[redacted]	-0.454337	-0.348632
chr2	51891753	51891762	[redacted]	-0.454274	-0.361125
chr19	50857404	50857437	[redacted]	-0.454208	-0.409133
chr1	157436536	157436611	[redacted]	-0.454147	-0.455995
chrX	38164978	38165049	[redacted]	-0.454107	-0.463522
chr4	138221692	138221708	[redacted]	-0.454102	-0.367383
chr9	43336079	43336120	[redacted]	-0.453047	-0.368953
chr2	36885173	36885255	[redacted]	-0.452645	-0.329758
chr7	111428081	111428147	[redacted]	-0.452424	-0.313343
chr18	81765742	81765802	[redacted]	-0.452422	-0.430251
chr15	11457462	11457510	[redacted]	-0.452212	-0.312488
chr4	85857272	85857321	[redacted]	-0.451862	-0.343812
chr12	75387630	75387668	[redacted]	-0.451837	-0.355734
chr11	43435041	43435094	[redacted]	-0.451706	-0.419678
chr4	118568967	118569043	[redacted]	-0.451428	-0.495523
chr2	49861893	49861918	[redacted]	-0.451345	-0.531142
chr16	30701139	30701177	[redacted]	-0.451089	-0.501633
chr15	63039418	63039482	[redacted]	-0.451078	-0.42489
chr12	9319903	9319940	[redacted]	-0.450801	-0.365195
chr7	74019479	74019572	[redacted]	-0.450609	-0.410968

chr3	36575556	36575688	[redacted]	-0.450489	-0.376461
chr6	134804216	134804277	[redacted]	-0.450123	-0.347434
chr8	26631290	26631345	[redacted]	-0.44953	-0.38485
chr13	19438194	19438247	[redacted]	-0.449203	-0.56423
chr11	70207593	70207638	[redacted]	-0.449183	-0.51254
chr10	93594939	93594974	[redacted]	-0.449115	-0.459099
chr14	35125802	35125898	[redacted]	-0.449093	-0.35861
chr9	64759409	64759433	[redacted]	-0.448933	-0.415988
chr17	46350865	46350929	[redacted]	-0.448897	-0.319954
chr8	31952791	31952852	[redacted]	-0.448701	-0.392406
chr7	11636237	11636287	[redacted]	-0.448677	-0.391523
chr3	131750526	131750602	[redacted]	-0.448565	-0.422362
chr15	43542457	43542530	[redacted]	-0.448269	-0.393105
chr4	152198393	152198410	[redacted]	-0.448264	-0.31097
chr9	95743205	95743287	[redacted]	-0.44786	-0.308227
chr9	82600506	82600560	[redacted]	-0.447594	-0.323171
chr8	66316068	66316121	[redacted]	-0.44756	-0.426118
chr7	76457593	76457659	[redacted]	-0.447411	-0.326318
chr13	8727128	8727174	[redacted]	-0.447245	-0.406479
chr17	56445561	56445633	[redacted]	-0.447234	-0.37396
chr14	86622132	86622180	[redacted]	-0.447087	-0.417726
chr1	34012344	34012397	[redacted]	-0.446977	-0.499296
chr9	20950271	20950313	[redacted]	-0.446779	-0.523265
chr2	164006907	164006944	[redacted]	-0.446769	-0.550716
chr14	93221372	93221395	[redacted]	-0.44672	-0.404471
chr8	77236556	77236601	[redacted]	-0.446282	-0.431737
chr1	88884308	88884388	[redacted]	-0.446226	-0.409983
chr1	68922749	68922798	[redacted]	-0.446047	-0.47317
chr6	109879644	109879703	[redacted]	-0.445977	-0.353649
chr9	117870191	117870251	[redacted]	-0.445831	-0.369146
chr11	66325912	66325953	[redacted]	-0.445292	-0.420386
chr5	97751713	97751778	[redacted]	-0.445121	-0.3524
chr11	86545568	86545615	[redacted]	-0.444791	-0.412043
chr1	180695629	180695678	[redacted]	-0.44476	-0.305765
chr12	71783177	71783211	[redacted]	-0.444731	-0.34109
chr6	143921171	143921241	[redacted]	-0.444709	-0.44957
chr14	54898459	54898541	[redacted]	-0.444674	-0.388753
chr6	51248486	51248537	[redacted]	-0.444634	-0.356124
chr9	33800577	33800633	[redacted]	-0.444095	-0.3859
chr6	72293490	72293533	[redacted]	-0.444045	-0.33902
chr12	53521690	53521746	[redacted]	-0.444013	-0.406676
chr6	139856816	139856846	[redacted]	-0.444013	-0.384154
chr14	106057445	106057486	[redacted]	-0.443931	-0.479285

chr6	4546354	4546434	[redacted]	-0.443781	-0.34195
chr7	78981224	78981306	[redacted]	-0.443546	-0.506563
chr7	134216041	134216122	[redacted]	-0.443521	-0.464935
chr10	40386378	40386434	[redacted]	-0.443341	-0.339184
chrX	74135953	74136041	[redacted]	-0.443225	-0.358392
chr4	7961961	7961987	[redacted]	-0.443212	-0.303523
chr14	99593360	99593396	[redacted]	-0.443203	-0.466092
chr9	73766662	73766749	[redacted]	-0.442944	-0.370347
chr6	12449718	12449756	[redacted]	-0.442885	-0.529195
chr11	69871519	69871556	[redacted]	-0.442832	-0.386809
chr9	71052071	71052119	[redacted]	-0.442546	-0.396535
chr13	88454059	88454141	[redacted]	-0.442252	-0.350646
chr1	20617303	20617370	[redacted]	-0.442154	-0.447709
chr11	94415482	94415545	[redacted]	-0.442087	-0.457781
chr11	8258527	8258591	[redacted]	-0.441985	-0.38451
chr7	82487425	82487471	[redacted]	-0.441876	-0.393862
chr2	133688565	133688676	[redacted]	-0.441735	-0.42035
chr4	128037498	128037608	[redacted]	-0.44173	-0.334644
chr9	65540750	65540763	[redacted]	-0.44166	-0.443578
chr16	84366119	84366200	[redacted]	-0.441629	-0.367665
chr6	34057856	34057912	[redacted]	-0.441566	-0.321657
chr17	74450626	74450691	[redacted]	-0.441472	-0.306536
chr11	32935126	32935144	[redacted]	-0.441451	-0.409109
chr16	41435754	41435800	[redacted]	-0.441443	-0.463597
chr17	37614057	37614111	[redacted]	-0.441284	-0.598692
chr5	103049723	103049764	[redacted]	-0.441022	-0.358104
chr1	38658421	38658527	[redacted]	-0.440895	-0.372482
chrX	163135526	163135580	[redacted]	-0.440779	-0.387309
chr9	42159521	42159567	[redacted]	-0.440594	-0.478476
chr8	36945186	36945245	[redacted]	-0.440282	-0.419632
chr18	58350397	58350472	[redacted]	-0.440215	-0.301868
chr4	24375237	24375297	[redacted]	-0.440036	-0.421107
chr11	35264928	35264970	[redacted]	-0.439849	-0.39699
chr12	99185167	99185188	[redacted]	-0.439799	-0.381175
chr10	81504729	81504772	[redacted]	-0.439775	-0.379216
chr1	42185981	42186028	[redacted]	-0.439417	-0.570549
chr7	87852309	87852328	[redacted]	-0.43925	-0.418406
chr11	17954184	17954259	[redacted]	-0.439124	-0.380411
chr14	78239304	78239318	[redacted]	-0.438951	-0.391668
chr6	141019666	141019700	[redacted]	-0.438693	-0.412682
chrX	93278318	93278384	[redacted]	-0.438679	-0.406755
chr19	39255382	39255439	[redacted]	-0.438565	-0.424666
chr10	11115338	11115413	[redacted]	-0.438458	-0.319034

chr14	88573102	88573173	[redacted]	-0.43825	-0.431934
chr1	127351565	127351626	[redacted]	-0.438079	-0.464606
chr5	120848077	120848172	[redacted]	-0.437925	-0.307051
chr13	43117597	43117668	[redacted]	-0.437889	-0.449268
chr2	128163536	128163616	[redacted]	-0.437801	-0.374733
chr17	8504157	8504192	[redacted]	-0.437506	-0.49854
chr8	20011013	20011175	[redacted]	-0.437255	-0.384371
chr2	56853227	56853263	[redacted]	-0.436971	-0.745725
chr2	121016408	121016486	[redacted]	-0.436716	-0.442645
chr4	134871688	134871701	[redacted]	-0.436628	-0.383732
chr9	53975933	53976060	[redacted]	-0.436615	-0.529819
chr10	32931301	32931380	[redacted]	-0.43659	-0.357552
chr16	46453379	46453419	[redacted]	-0.436508	-0.375576
chr18	68178378	68178446	[redacted]	-0.436435	-0.378431
chr17	68726762	68726839	[redacted]	-0.436405	-0.435262
chr11	14037369	14037436	[redacted]	-0.43616	-0.383955
chr1	180475686	180475746	[redacted]	-0.436071	-0.351143
chr14	100397875	100397911	[redacted]	-0.435843	-0.441335
chr2	180747508	180747527	[redacted]	-0.435655	-0.487388
chr10	82370265	82370320	[redacted]	-0.435612	-0.422758
chr14	106651785	106651807	[redacted]	-0.435438	-0.394745
chr6	42247343	42247424	[redacted]	-0.435385	-0.482591
chr3	67482934	67483017	[redacted]	-0.435315	-0.384126
chr11	18836777	18836833	[redacted]	-0.435055	-0.409962
chr7	3935339	3935406	[redacted]	-0.434852	-0.352608
chr11	30298042	30298101	[redacted]	-0.434692	-0.319712
chr10	122462697	122462726	[redacted]	-0.43447	-0.316037
chr11	92020511	92020577	[redacted]	-0.434223	-0.506285
chr9	58475337	58475372	[redacted]	-0.434112	-0.419231
chr17	71636838	71636907	[redacted]	-0.433889	-0.315854
chr2	118880319	118880338	[redacted]	-0.433871	-0.398
chr18	8886848	8886903	[redacted]	-0.433744	-0.348655
chr2	136348566	136348630	[redacted]	-0.433699	-0.318159
chr13	108407325	108407463	[redacted]	-0.433493	-0.449892
chr5	60102823	60102905	[redacted]	-0.433418	-0.362755
chr5	34326848	34326881	[redacted]	-0.433395	-0.334393
chr10	20387641	20387711	[redacted]	-0.433254	-0.329533
chr1	189136246	189136317	[redacted]	-0.433223	-0.366071
chr9	32378558	32378672	[redacted]	-0.433143	-0.417068
chr15	84744164	84744212	[redacted]	-0.433135	-0.324141
chr5	104852937	104852968	[redacted]	-0.43301	-0.393894
chr1	94268555	94268623	[redacted]	-0.432933	-0.427502
chr14	51916051	51916076	[redacted]	-0.432889	-0.406278

chr13	115312078	115312124	[redacted]	-0.432883	-0.413935
chr17	87398130	87398160	[redacted]	-0.432585	-0.393729
chr9	73740925	73740964	[redacted]	-0.432447	-0.434531
chr15	95741699	95741733	[redacted]	-0.432333	-0.347449
chr14	17286618	17286705	[redacted]	-0.432195	-0.343193
chr5	91146648	91146708	[redacted]	-0.432171	-0.473943
chr19	59518062	59518108	[redacted]	-0.431955	-0.360619
chr12	16603407	16603463	[redacted]	-0.431735	-0.389692
chr6	106679723	106679789	[redacted]	-0.431688	-0.328401
chr11	80622368	80622402	[redacted]	-0.431447	-0.422281
chr18	75704268	75704344	[redacted]	-0.431437	-0.539567
chr10	124777136	124777177	[redacted]	-0.431408	-0.484729
chr17	43166761	43166811	[redacted]	-0.431343	-0.369368
chr4	121248348	121248405	[redacted]	-0.431069	-0.317492
chr14	87966515	87966566	[redacted]	-0.431016	-0.431621
chrX	10872962	10873004	[redacted]	-0.430895	-0.529837
chr1	127154085	127154105	[redacted]	-0.430589	-0.392098
chr6	92635042	92635083	[redacted]	-0.430454	-0.461413
chr18	44366339	44366393	[redacted]	-0.430304	-0.38288
chr4	148860854	148860885	[redacted]	-0.429657	-0.345243
chr15	82686030	82686074	[redacted]	-0.429227	-0.460476
chrX	150888885	150888935	[redacted]	-0.429116	-0.583071
chr15	10170906	10171005	[redacted]	-0.428666	-0.359536
chr3	86045216	86045263	[redacted]	-0.428516	-0.317784
chr10	3959343	3959405	[redacted]	-0.428443	-0.349138
chr5	90907962	90908019	[redacted]	-0.428368	-0.454992
chr1	82326271	82326373	[redacted]	-0.428208	-0.447362
chr12	44895873	44895970	[redacted]	-0.427972	-0.406539
chr5	74859664	74859706	[redacted]	-0.427702	-0.381899
chr10	53794519	53794577	[redacted]	-0.427557	-0.333091
chr12	82689634	82689684	[redacted]	-0.427451	-0.316576
chr5	85967463	85967503	[redacted]	-0.427241	-0.395648
chrX	102218454	102218552	[redacted]	-0.427179	-0.635869
chr13	95611253	95611392	[redacted]	-0.427065	-0.458928
chr16	43548085	43548172	[redacted]	-0.426932	-0.506338
chr5	41216657	41216674	[redacted]	-0.426853	-0.485278
chr1	55448971	55449050	[redacted]	-0.426756	-0.33651
chr12	54059690	54059784	[redacted]	-0.426649	-0.39995
chr18	42230142	42230175	[redacted]	-0.426612	-0.357425
chr19	51420607	51420653	[redacted]	-0.426519	-0.353911
chr16	51671852	51671897	[redacted]	-0.42648	-0.440655
chr6	106178576	106178658	[redacted]	-0.426439	-0.395741
chr9	48215215	48215307	[redacted]	-0.425941	-0.378761

chr1	151265718	151265772	[redacted]	-0.42586	-0.381774
chr8	40627568	40627617	[redacted]	-0.425804	-0.404276
chr16	6936889	6936942	[redacted]	-0.425738	-0.493779
chr17	18367289	18367360	[redacted]	-0.425736	-0.412949
chr4	151251582	151251631	[redacted]	-0.425654	-0.322368
chr17	19325306	19325332	[redacted]	-0.425611	-0.389811
chr3	16726264	16726277	[redacted]	-0.425566	-0.459625
chr8	75472068	75472130	[redacted]	-0.425503	-0.367765
chr9	118485146	118485167	[redacted]	-0.425332	-0.349654
chr5	134289315	134289399	[redacted]	-0.425306	-0.311384
chr13	43603414	43603457	[redacted]	-0.425255	-0.406372
chr14	8086358	8086404	[redacted]	-0.425112	-0.37666
chr19	21496727	21496778	[redacted]	-0.42482	-0.423642
chr6	147003852	147003894	[redacted]	-0.424588	-0.384495
chr3	123645416	123645472	[redacted]	-0.424558	-0.36269
chr7	39992385	39992410	[redacted]	-0.424556	-0.434117
chr3	30325675	30325730	[redacted]	-0.42447	-0.449946
chr11	22872911	22872979	[redacted]	-0.424282	-0.44728
chr5	70320392	70320456	[redacted]	-0.424212	-0.350204
chr8	78219027	78219072	[redacted]	-0.423949	-0.388918
chr10	3809760	3809808	[redacted]	-0.423935	-0.429901
chrX	94827963	94828030	[redacted]	-0.423829	-0.310635
chr9	33389727	33389791	[redacted]	-0.42334	-0.329045
chr14	48245489	48245555	[redacted]	-0.423277	-0.435046
chr8	49995542	49995604	[redacted]	-0.423183	-0.373039
chr3	80697518	80697538	[redacted]	-0.42308	-0.474237
chr9	65608093	65608123	[redacted]	-0.422862	-0.382824
chr1	29014383	29014436	[redacted]	-0.422837	-0.446327
chr8	91335477	91335572	[redacted]	-0.422722	-0.405376
chr4	3260098	3260170	[redacted]	-0.422715	-0.315615
chr1	118170068	118170134	[redacted]	-0.422547	-0.487314
chr9	12185194	12185216	[redacted]	-0.422542	-0.485451
chr2	80787414	80787463	[redacted]	-0.422535	-0.346562
chr17	23641789	23641829	[redacted]	-0.422371	-0.313438
chr2	119985866	119985920	[redacted]	-0.422157	-0.398271
chr7	19991784	19991853	[redacted]	-0.422009	-0.378649
chr15	38197540	38197582	[redacted]	-0.422005	-0.403681
chr10	86992588	86992613	[redacted]	-0.421844	-0.529198
chr10	126764528	126764548	[redacted]	-0.421782	-0.552386
chr9	84108674	84108728	[redacted]	-0.421711	-0.370562
chr14	105722609	105722665	[redacted]	-0.421708	-0.305253
chr5	16055006	16055061	[redacted]	-0.421687	-0.330803
chr9	54810862	54810938	[redacted]	-0.421651	-0.407228

chr12	112554901	112554940	[redacted]	-0.421604	-0.440429
chr3	7507796	7507832	[redacted]	-0.421112	-0.359044
chr2	151434384	151434460	[redacted]	-0.420997	-0.387947
chr12	40588268	40588302	[redacted]	-0.420915	-0.448576
chr9	21849614	21849683	[redacted]	-0.420902	-0.375312
chr7	103660256	103660333	[redacted]	-0.420791	-0.444172
chr17	90481670	90481765	[redacted]	-0.420791	-0.425867
chr17	66518171	66518260	[redacted]	-0.420706	-0.362022
chr10	24588392	24588465	[redacted]	-0.420681	-0.312657
chr13	108807917	108808002	[redacted]	-0.420678	-0.343971
chr13	65390385	65390445	[redacted]	-0.42063	-0.411678
chr18	67267125	67267152	[redacted]	-0.420476	-0.31078
chr15	3761522	3761620	[redacted]	-0.420414	-0.398979
chr18	33208214	33208301	[redacted]	-0.420364	-0.358329
chr2	127947562	127947640	[redacted]	-0.42013	-0.380532
chr1	68591420	68591436	[redacted]	-0.420104	-0.402967
chr12	117712681	117712740	[redacted]	-0.42006	-0.303884
chr15	55357828	55357880	[redacted]	-0.419985	-0.359515
chr1	141057380	141057436	[redacted]	-0.419947	-0.479285
chr2	10285980	10286012	[redacted]	-0.419923	-0.472097
chr9	24920369	24920396	[redacted]	-0.419772	-0.576969
chr1	164347163	164347220	[redacted]	-0.419315	-0.382723
chr1	75679012	75679063	[redacted]	-0.419271	-0.340579
chr16	37771159	37771204	[redacted]	-0.419158	-0.371725
chr3	143006989	143007060	[redacted]	-0.419144	-0.341966
chr1	111711181	111711224	[redacted]	-0.419024	-0.542141
chr6	43873915	43873996	[redacted]	-0.418946	-0.336479
chr18	13093705	13093765	[redacted]	-0.418864	-0.345441
chr6	100671870	100671902	[redacted]	-0.418503	-0.356734
chr1	150755639	150755675	[redacted]	-0.418423	-0.354829
chr11	85298211	85298296	[redacted]	-0.418355	-0.465678
chr3	147129957	147130065	[redacted]	-0.418261	-0.358353
chr15	86009820	86009893	[redacted]	-0.418167	-0.351494
chr1	6040441	6040483	[redacted]	-0.418028	-0.360356
chr4	82532898	82532977	[redacted]	-0.418001	-0.427724
chr19	20551845	20551895	[redacted]	-0.417924	-0.467215
chr3	50980056	50980104	[redacted]	-0.41792	-0.364679
chr3	50045651	50045660	[redacted]	-0.417748	-0.410052
chr1	139734237	139734299	[redacted]	-0.417203	-0.44356
chr3	41256417	41256489	[redacted]	-0.417128	-0.415885
chr5	48521893	48521954	[redacted]	-0.41683	-0.434921
chr4	80448180	80448231	[redacted]	-0.416812	-0.379524
chr17	73417722	73417804	[redacted]	-0.416717	-0.412007

chr1	78223562	78223634	[redacted]	-0.416703	-0.468347
chr5	90164273	90164326	[redacted]	-0.416584	-0.408471
chr2	4850415	4850451	[redacted]	-0.41652	-0.338258
chr8	68619344	68619408	[redacted]	-0.41648	-0.564925
chr6	29165254	29165315	[redacted]	-0.41644	-0.319468
chr17	44609466	44609527	[redacted]	-0.416383	-0.319017
chr8	36552144	36552199	[redacted]	-0.416369	-0.406194
chr9	92336618	92336649	[redacted]	-0.416319	-0.489711
chr18	77618725	77618878	[redacted]	-0.416218	-0.362663
chr4	29324173	29324216	[redacted]	-0.416186	-0.43845
chr10	115992489	115992553	[redacted]	-0.416173	-0.446302
chr2	120471953	120471977	[redacted]	-0.416004	-0.386476
chr8	36363206	36363253	[redacted]	-0.415779	-0.385436
chr2	76214296	76214349	[redacted]	-0.415673	-0.323012
chr13	108356307	108356338	[redacted]	-0.415663	-0.394095
chr17	57312123	57312165	[redacted]	-0.415578	-0.320158
chr2	52502336	52502387	[redacted]	-0.415535	-0.425058
chr14	101975516	101975555	[redacted]	-0.415274	-0.329628
chr11	30377108	30377148	[redacted]	-0.415258	-0.415835
chr10	118774840	118774881	[redacted]	-0.414991	-0.39571
chr10	97403707	97403762	[redacted]	-0.414944	-0.335502
chr2	14190735	14190798	[redacted]	-0.414924	-0.401564
chr9	111000051	111000101	[redacted]	-0.414773	-0.314136
chr2	110351873	110351919	[redacted]	-0.414729	-0.434999
chr9	61137011	61137056	[redacted]	-0.414616	-0.411176
chr11	13554167	13554247	[redacted]	-0.414593	-0.420495
chr5	44722213	44722246	[redacted]	-0.414559	-0.379622
chr15	72309906	72309945	[redacted]	-0.414358	-0.399774
chr14	86331017	86331049	[redacted]	-0.413763	-0.41757
chr7	79618075	79618093	[redacted]	-0.413636	-0.38292
chr10	111873585	111873659	[redacted]	-0.413595	-0.322385
chr16	46528324	46528339	[redacted]	-0.413578	-0.394044
chr18	67162656	67162722	[redacted]	-0.413541	-0.335675
chr2	51212727	51212784	[redacted]	-0.413466	-0.375807
chr16	73074581	73074632	[redacted]	-0.413406	-0.496283
chr3	21487189	21487275	[redacted]	-0.413338	-0.363868
chr16	89836293	89836342	[redacted]	-0.413258	-0.477157
chr1	64043223	64043269	[redacted]	-0.413109	-0.381126
chr9	98109169	98109201	[redacted]	-0.41303	-0.345732
chr2	27115638	27115677	[redacted]	-0.413017	-0.440365
chr3	136566071	136566123	[redacted]	-0.412859	-0.374075
chr14	64235973	64235998	[redacted]	-0.412752	-0.368627
chr1	156818661	156818710	[redacted]	-0.412749	-0.449113

chr7	65961838	65961869	[redacted]	-0.412446	-0.364624
chr4	31006629	31006678	[redacted]	-0.412354	-0.419903
chr14	113599502	113599636	[redacted]	-0.41224	-0.384747
chr5	131403375	131403419	[redacted]	-0.412235	-0.417973
chr1	21995259	21995270	[redacted]	-0.412182	-0.394669
chr11	18852284	18852348	[redacted]	-0.411907	-0.48065
chr15	86874475	86874557	[redacted]	-0.411869	-0.321897
chr12	79946914	79946955	[redacted]	-0.411847	-0.374934
chr6	93826506	93826565	[redacted]	-0.411818	-0.422451
chr5	118373187	118373246	[redacted]	-0.411786	-0.379481
chr16	68382833	68382898	[redacted]	-0.411695	-0.316846
chr2	55593081	55593138	[redacted]	-0.411485	-0.343285
chr11	55003961	55004016	[redacted]	-0.411356	-0.428662
chr13	82422921	82422976	[redacted]	-0.411031	-0.325052
chr8	30375533	30375584	[redacted]	-0.410999	-0.368982
chr1	6250492	6250564	[redacted]	-0.410985	-0.385035
chr10	71104433	71104461	[redacted]	-0.410833	-0.360976
chr6	19470878	19470928	[redacted]	-0.410619	-0.317939
chr12	17139922	17140012	[redacted]	-0.410617	-0.341019
chr10	29349671	29349698	[redacted]	-0.410444	-0.384071
chr1	150372959	150373014	[redacted]	-0.410385	-0.471472
chr16	91789219	91789256	[redacted]	-0.410325	-0.310765
chr8	85267534	85267586	[redacted]	-0.410298	-0.364771
chr14	58301600	58301653	[redacted]	-0.410284	-0.304918
chr14	53563147	53563191	[redacted]	-0.41026	-0.449624
chr17	67890648	67890714	[redacted]	-0.410242	-0.443253
chr17	85512459	85512503	[redacted]	-0.41018	-0.303137
chr1	85995244	85995313	[redacted]	-0.410148	-0.36619
chr14	95031128	95031157	[redacted]	-0.410083	-0.400715
chr1	131599495	131599558	[redacted]	-0.409865	-0.436741
chr9	94493731	94493768	[redacted]	-0.409836	-0.366252
chr12	72153866	72153899	[redacted]	-0.409834	-0.470711
chr16	33601480	33601570	[redacted]	-0.409776	-0.451898
chr10	63720635	63720718	[redacted]	-0.409771	-0.304001
chr3	73804481	73804538	[redacted]	-0.409639	-0.422694
chr9	72109668	72109717	[redacted]	-0.409494	-0.427306
chr2	26670069	26670120	[redacted]	-0.409236	-0.38738
chr11	104692572	104692652	[redacted]	-0.409215	-0.412456
chr5	30401299	30401390	[redacted]	-0.409175	-0.39868
chr3	102006430	102006502	[redacted]	-0.409154	-0.34108
chr3	130693325	130693372	[redacted]	-0.409144	-0.479109
chr17	10587796	10587813	[redacted]	-0.409141	-0.329858
chr11	90204053	90204126	[redacted]	-0.40902	-0.31581

chr11	11463412	11463477		-0.408979	-0.30057
chr11	32815420	32815478		-0.408953	-0.317621
chr1	96976333	96976461		-0.408745	-0.370017
chr8	8371965	8371993		-0.40872	-0.388548
chr3	138562055	138562104		-0.408711	-0.328243
chr11	19360626	19360683		-0.408653	-0.415488
chr7	104738118	104738146		-0.408552	-0.494987
chr9	63821677	63821722		-0.408497	-0.409951
chr10	88385535	88385584		-0.408419	-0.422814
chr12	46952585	46952628		-0.408349	-0.372664
chr13	62703349	62703424		-0.408324	-0.38667
chr1	67681826	67681893		-0.408319	-0.394546
chr4	115653411	115653518		-0.408266	-0.365166
chr17	51450901	51450925		-0.408133	-0.378702
chr6	89156230	89156238		-0.408096	-0.372995
chr3	60067795	60067861		-0.408008	-0.392091
chr8	39162557	39162611		-0.407905	-0.353147
chr3	136948718	136948888		-0.407881	-0.483346
chr6	88206821	88206866		-0.40766	-0.386233
chr18	57378312	57378369		-0.407638	-0.346981
chr3	6383408	6383427		-0.407585	-0.410044
chr14	66967700	66967780		-0.407528	-0.388114
chr18	77787867	77787910		-0.407478	-0.303743
chr14	48240743	48240864		-0.407423	-0.366398
chr9	22404286	22404349		-0.407319	-0.328026
chr15	60576694	60576755		-0.407262	-0.35362
chr16	14726932	14727037		-0.407215	-0.337749
chrX	49854487	49854576		-0.407144	-0.524152
chr6	139565387	139565429		-0.407122	-0.406329
chr11	14162365	14162415		-0.406866	-0.343395
chr15	43444613	43444665		-0.40679	-0.530719
chr12	60281840	60281880		-0.406782	-0.462413
chr17	33404937	33405018		-0.406636	-0.569653
chr6	6710302	6710382		-0.406626	-0.405826
chr9	46550732	46550807		-0.406505	-0.347804
chr18	46894725	46894764		-0.406403	-0.524359
chr8	6392238	6392297		-0.405777	-0.310335
chr6	142434162	142434216		-0.405655	-0.347934
chr13	85861631	85861673		-0.405469	-0.379839
chr11	8284277	8284334		-0.405454	-0.300208
chr10	62382268	62382318		-0.405379	-0.421805
chr10	115486160	115486230		-0.405224	-0.330602
chr8	55992418	55992468		-0.405191	-0.332848

chr15	26791684	26791747	[redacted]	-0.405158	-0.32035
chr5	76828226	76828285	[redacted]	-0.405114	-0.500092
chr1	185808372	185808438	[redacted]	-0.404917	-0.420788
chr2	29764097	29764160	[redacted]	-0.404687	-0.317665
chr2	91651587	91651626	[redacted]	-0.404613	-0.477927
chr7	45792194	45792262	[redacted]	-0.404581	-0.406564
chr7	142887612	142887664	[redacted]	-0.404448	-0.373894
chr14	88764169	88764218	[redacted]	-0.404306	-0.459862
chr12	81707003	81707056	[redacted]	-0.404165	-0.442569
chr4	94604206	94604308	[redacted]	-0.404122	-0.378011
chr6	137584564	137584595	[redacted]	-0.404072	-0.42982
chr1	144166791	144166856	[redacted]	-0.404054	-0.335191
chr13	46058176	46058241	[redacted]	-0.404035	-0.382553
chr14	10878189	10878219	[redacted]	-0.403975	-0.396256
chr2	111761314	111761335	[redacted]	-0.403878	-0.557252
chr8	118625517	118625567	[redacted]	-0.403739	-0.521281
chr13	29346330	29346363	[redacted]	-0.403531	-0.317705
chr11	51130069	51130116	[redacted]	-0.403504	-0.595432
chr1	30756745	30756783	[redacted]	-0.403308	-0.335603
chr14	48214412	48214451	[redacted]	-0.403283	-0.383036
chr2	121963718	121963771	[redacted]	-0.403269	-0.326858
chr6	144012272	144012316	[redacted]	-0.403185	-0.367242
chr1	172342275	172342334	[redacted]	-0.403182	-0.512104
chr16	45914469	45914505	[redacted]	-0.40314	-0.420187
chr13	103516648	103516671	[redacted]	-0.40312	-0.362392
chr1	67951758	67951791	[redacted]	-0.402945	-0.405277
chr9	107472730	107472777	[redacted]	-0.402902	-0.415016
chr18	80020423	80020520	[redacted]	-0.402887	-0.312218
chr9	66629813	66629858	[redacted]	-0.402772	-0.421985
chr16	58281229	58281301	[redacted]	-0.402694	-0.377473
chr17	64857534	64857598	[redacted]	-0.402679	-0.302111
chr13	74296681	74296719	[redacted]	-0.402366	-0.519717
chr2	12808701	12808781	[redacted]	-0.402258	-0.352985
chr17	35304174	35304200	[redacted]	-0.402062	-0.358644
chr11	93206354	93206408	[redacted]	-0.401842	-0.498059
chr17	21762111	21762177	[redacted]	-0.401839	-0.31308
chr14	88641046	88641072	[redacted]	-0.401791	-0.39722
chr10	43183517	43183564	[redacted]	-0.401771	-0.37073
chr8	112903956	112904021	[redacted]	-0.401747	-0.312922
chr3	142578374	142578422	[redacted]	-0.401742	-0.359944
chr18	64949487	64949570	[redacted]	-0.40174	-0.438991
chr2	74196407	74196477	[redacted]	-0.401679	-0.332802
chr12	74080988	74081004	[redacted]	-0.40146	-0.370471

chr14	51367273	51367301	[redacted]	-0.401352	-0.409957
chr7	37828009	37828095	[redacted]	-0.401329	-0.336325
chr17	74069920	74069987	[redacted]	-0.401257	-0.321322
chr11	82152703	82152727	[redacted]	-0.40115	-0.454753
chr3	138856199	138856274	[redacted]	-0.401148	-0.392265
chr18	82042006	82042074	[redacted]	-0.401052	-0.310029
chr13	63671397	63671429	[redacted]	-0.401	-0.338623
chr11	110358345	110358385	[redacted]	-0.40095	-0.487919
chr4	35934466	35934498	[redacted]	-0.400666	-0.469688
chr5	60431478	60431545	[redacted]	-0.400621	-0.445857
chr5	46669405	46669457	[redacted]	-0.400597	-0.421133
chr2	33579451	33579513	[redacted]	-0.400436	-0.385694
chr2	64858431	64858477	[redacted]	-0.400405	-0.421735
chr17	43641662	43641672	[redacted]	-0.400397	-0.403174
chr8	25323477	25323532	[redacted]	-0.400396	-0.385099
chr15	24743295	24743348	[redacted]	-0.400384	-0.412196
chr9	42850706	42850783	[redacted]	-0.400157	-0.32453
chr3	139693693	139693746	[redacted]	-0.400106	-0.438775
chr1	169405497	169405543	[redacted]	-0.400078	-0.407728
chr13	120042534	120042565	[redacted]	-0.400014	-0.573203
chr12	118734325	118734388	[redacted]	-0.39999	-0.423317
chr11	89948606	89948689	[redacted]	-0.399846	-0.472488
chrX_G	289449	289522	[redacted]	-0.399804	-0.359266
chr9	25255527	25255580	[redacted]	-0.399799	-0.381103
chr12	43334853	43334892	[redacted]	-0.399736	-0.481009
chr7	17710692	17710775	[redacted]	-0.399709	-0.326679
chr11	10235457	10235476	[redacted]	-0.399654	-0.605531
chr12	31522592	31522652	[redacted]	-0.399628	-0.301367
chr13	38087503	38087534	[redacted]	-0.399303	-0.343436
chr2	79288436	79288521	[redacted]	-0.399259	-0.319716
chr5	134989524	134989602	[redacted]	-0.399257	-0.35866
chr5	138113833	138113889	[redacted]	-0.39918	-0.324288
chr5	113893812	113893846	[redacted]	-0.399175	-0.416978
chr12	71653451	71653500	[redacted]	-0.399091	-0.453092
chr10	42002302	42002361	[redacted]	-0.399022	-0.378788
chrX	9476223	9476289	[redacted]	-0.398925	-0.390693
chr13	18679990	18680038	[redacted]	-0.398735	-0.422076
chr1	30816442	30816485	[redacted]	-0.398727	-0.392556
chr2	77172504	77172571	[redacted]	-0.39865	-0.301249
chr17	17009488	17009576	[redacted]	-0.398618	-0.428879
chr8	95318825	95318862	[redacted]	-0.398463	-0.356426
chr18	62340214	62340242	[redacted]	-0.398434	-0.301784
chr17	42051508	42051532	[redacted]	-0.398381	-0.40343

chr18	33621470	33621508		-0.398294	-0.440269
chr6	142643234	142643280		-0.398249	-0.422637
chr17	46352085	46352122		-0.398098	-0.444376
chr6	8178509	8178522		-0.397995	-0.338562
chr15	42096256	42096358		-0.397886	-0.37047
chr17	57381164	57381238		-0.397813	-0.311176
chr1	90976489	90976517		-0.397632	-0.429168
chr18	52993884	52993912		-0.397379	-0.318946
chr8	128030618	128030670		-0.39721	-0.340721
chr2	87001941	87001994		-0.397204	-0.447073
chr12	34761137	34761165		-0.397169	-0.385434
chr1	44223918	44224040		-0.397156	-0.475227
chr14	11653426	11653485		-0.397055	-0.388116
chr17	89079454	89079527		-0.397032	-0.32183
chr6	147592238	147592330		-0.397027	-0.308343
chr4	55567484	55567560		-0.396886	-0.393104
chr5	109707743	109707804		-0.396746	-0.321639
chr4	89094715	89094745		-0.396452	-0.311305
chr13	15096247	15096326		-0.396408	-0.422701
chr1	68387840	68387862		-0.396391	-0.359419
chr16	86376409	86376449		-0.396217	-0.40481
chr15	102712684	102712715		-0.396186	-0.364772
chr8	29997524	29997570		-0.396181	-0.417489
chr18	34330432	34330470		-0.396153	-0.352589
chr6	8839708	8839788		-0.39615	-0.323317
chr2	51672878	51672940		-0.396023	-0.383198
chr14	89641667	89641728		-0.395999	-0.386629
chr9	85238720	85238818		-0.395911	-0.38572
chr8	30459611	30459651		-0.395885	-0.413931
chr4	99363247	99363287		-0.395846	-0.420804
chr16	26961621	26961671		-0.395817	-0.32709
chr17	65517096	65517197		-0.395696	-0.300845
chr9	41864839	41864884		-0.395585	-0.419247
chr2	140324655	140324724		-0.395558	-0.361871
chr2	169359970	169360039		-0.395556	-0.403231
chr5	90026845	90026940		-0.395517	-0.407332
chr3	117485265	117485293		-0.395467	-0.394723
chr13	119724603	119724708		-0.395452	-0.330063
chr1	65877378	65877441		-0.395408	-0.415759
chr18	66713357	66713380		-0.395407	-0.361781
chr4	136414297	136414329		-0.395399	-0.321463
chrX	50050290	50050323		-0.395342	-0.455588
chr4	149672357	149672392		-0.395264	-0.388091

chr14	59844230	59844305		-0.395261	-0.367654
chr2	45719232	45719289		-0.395082	-0.385285
chr3	88233453	88233489		-0.394941	-0.41327
chr8	11033733	11033795		-0.394882	-0.386967
chr18	84195215	84195307		-0.394657	-0.370306
chr6	12942384	12942401		-0.39465	-0.422334
chr9	21359837	21359887		-0.394633	-0.352315
chr14	92753339	92753381		-0.394601	-0.416368
chr18	33360604	33360657		-0.394518	-0.353575
chr10	122133285	122133372		-0.394497	-0.376414
chr17	48523475	48523496		-0.394483	-0.384045
chr9	94048252	94048302		-0.394283	-0.468412
chr17	64186104	64186155		-0.394216	-0.312701
chr6	53839496	53839560		-0.393999	-0.33484
chr11	56151480	56151528		-0.393988	-0.392531
chr3	16398705	16398776		-0.393825	-0.361953
chr2	73971057	73971100		-0.393771	-0.406035
chr8	83488027	83488043		-0.39375	-0.481944
chr12	97640347	97640371		-0.393667	-0.380979
chr7	66245053	66245084		-0.393658	-0.374948
chr12	90629530	90629557		-0.393585	-0.382127
chr16	74479777	74479859		-0.393561	-0.543713
chr17	80083425	80083493		-0.393465	-0.360998
chr12	68880514	68880581		-0.393411	-0.314145
chr11	70820954	70821026		-0.393393	-0.36067
chr10	95904938	95904981		-0.393347	-0.33961
chr8	32372908	32372929		-0.393088	-0.37316
chr12	34947207	34947266		-0.392929	-0.375463
chr17	76105027	76105114		-0.39272	-0.300338
chr4	17190322	17190377		-0.392604	-0.408916
chr14	92864940	92864981		-0.392575	-0.382573
chr12	81040823	81040860		-0.392511	-0.35001
chr11	80831108	80831172		-0.392421	-0.371673
chr7	86737433	86737458		-0.392375	-0.30611
chr11	11418830	11418883		-0.392325	-0.569253
chr1	179371635	179371689		-0.392308	-0.382734
chr2	71190731	71190763		-0.392248	-0.364657
chr4	143076123	143076170		-0.392247	-0.433636
chr4	75957064	75957117		-0.39211	-0.356572
chr7	35451575	35451633		-0.392064	-0.301376
chr6	140396730	140396755		-0.391954	-0.397774
chr16	86585427	86585469		-0.391881	-0.373969
chr18	50138690	50138697		-0.391751	-0.351604

chr6	85081558	85081637		-0.391604	-0.309574
chr13	99407731	99407760		-0.391485	-0.350463
chr19	36536924	36536963		-0.391414	-0.382697
chr2	162027819	162027944		-0.391375	-0.558684
chr12	104491944	104491980		-0.391359	-0.406825
chr9	80335916	80336003		-0.391318	-0.319773
chr9	119548920	119548983		-0.391246	-0.442561
chr7	70415536	70415591		-0.391187	-0.313629
chr10	26561336	26561368		-0.391169	-0.359708
chr18	79515961	79516009		-0.39116	-0.319077
chr7	121119915	121119963		-0.391131	-0.300546
chr11	58234936	58235005		-0.391027	-0.467731
chr15	103436083	103436115		-0.390921	-0.32184
chr7	72202908	72202954		-0.390888	-0.364083
chr6	22002659	22002701		-0.390883	-0.492768
chr11	29316326	29316343		-0.390764	-0.450961
chr15	65967193	65967244		-0.390726	-0.365027
chr2	160938058	160938132		-0.390705	-0.412972
chr13	53357291	53357329		-0.390556	-0.384323
chr6	108614747	108614775		-0.390466	-0.381827
chr13	11543823	11543895		-0.390301	-0.331428
chr13	97376246	97376304		-0.390222	-0.354148
chr7	105784952	105785024		-0.390207	-0.371764
chr5	130856852	130856873		-0.390125	-0.346508
chr12	86674531	86674575		-0.390073	-0.37424
chr6	124544804	124544872		-0.389996	-0.315322
chr13	70041558	70041662		-0.389993	-0.396589
chr8	80511255	80511300		-0.389985	-0.316593
chr14	48740645	48740719		-0.389891	-0.473864
chr13	95823452	95823505		-0.389886	-0.43479
chr16	91004912	91005007		-0.389739	-0.37823
chrX	52899588	52899662		-0.389704	-0.4206
chr3	89662544	89662616		-0.389692	-0.445796
chr17	78649811	78649890		-0.389515	-0.338266
chr2	107702317	107702366		-0.389459	-0.399364
chr6	24296089	24296161		-0.389257	-0.346156
chr1	151013028	151013075		-0.389197	-0.345567
chr5	39455733	39455761		-0.389117	-0.373227
chr19	60113091	60113117		-0.389048	-0.36493
chr16	7086814	7086874		-0.388958	-0.39164
chr16	22576036	22576134		-0.388934	-0.346981
chr10	41860707	41860744		-0.388882	-0.318782
chr5	52608572	52608668		-0.38888	-0.310464

chr13	23492041	23492079	[redacted]	-0.388841	-0.333597
chr11	96222550	96222584	[redacted]	-0.388682	-0.458728
chr19	38199068	38199099	[redacted]	-0.388615	-0.479596
chr14	65166594	65166649	[redacted]	-0.388576	-0.380193
chr12	26661982	26662029	[redacted]	-0.388529	-0.478325
chr11	44890143	44890190	[redacted]	-0.388387	-0.367259
chr8	31629899	31629934	[redacted]	-0.388348	-0.423846
chr7	98693412	98693468	[redacted]	-0.388217	-0.413231
chr6	67970402	67970443	[redacted]	-0.388184	-0.568647
chr5	127121481	127121530	[redacted]	-0.387862	-0.410693
chr14	6899949	6899990	[redacted]	-0.387841	-0.42528
chr4	84288834	84288850	[redacted]	-0.387799	-0.36049
chr1	7047216	7047292	[redacted]	-0.387792	-0.423176
chr7	110656242	110656281	[redacted]	-0.387582	-0.315598
chr17	92047679	92047745	[redacted]	-0.387491	-0.447786
chr2	58352862	58352943	[redacted]	-0.38745	-0.423212
chr1	172577463	172577560	[redacted]	-0.38744	-0.30919
chr12	47062941	47062971	[redacted]	-0.387423	-0.399863
chr1	12977528	12977562	[redacted]	-0.387394	-0.379585
chr2	99706013	99706073	[redacted]	-0.387336	-0.453222
chr5	106605298	106605368	[redacted]	-0.387299	-0.389412
chr4	40318790	40318829	[redacted]	-0.387253	-0.302723
chr6	35412882	35412986	[redacted]	-0.387231	-0.368157
chr6	125320962	125321026	[redacted]	-0.387191	-0.397354
chr19	8839781	8839891	[redacted]	-0.387181	-0.372377
chr17	33404337	33404367	[redacted]	-0.387161	-0.42896
chr15	83023081	83023146	[redacted]	-0.387108	-0.326429
chr2	177808008	177808062	[redacted]	-0.387057	-0.365019
chr18	75710491	75710548	[redacted]	-0.387013	-0.416167
chr8	23637487	23637580	[redacted]	-0.386834	-0.374839
chr6	38858360	38858430	[redacted]	-0.386807	-0.303542
chr6	135625860	135625874	[redacted]	-0.386771	-0.388755
chr9	105818728	105818805	[redacted]	-0.386708	-0.545484
chr8	81343882	81343972	[redacted]	-0.386569	-0.311246
chr12	10365032	10365072	[redacted]	-0.386542	-0.401607
chrX	58820300	58820362	[redacted]	-0.386542	-0.309293
chr3	67174603	67174611	[redacted]	-0.386514	-0.3743
chr6	65476575	65476653	[redacted]	-0.386402	-0.301613
chr8	36816458	36816508	[redacted]	-0.386335	-0.4329
chr16	45263290	45263307	[redacted]	-0.386334	-0.36012
chr2	36198448	36198511	[redacted]	-0.386197	-0.380288
chr11	18933354	18933411	[redacted]	-0.386189	-0.369655
chr13	98512498	98512560	[redacted]	-0.386041	-0.337826

chr19	37589702	37589734	[redacted]	-0.386015	-0.360691
chr15	51778485	51778512	[redacted]	-0.385998	-0.386311
chrX	90143370	90143426	[redacted]	-0.385614	-0.30994
chr14	88901308	88901363	[redacted]	-0.385566	-0.391829
chr15	27204227	27204307	[redacted]	-0.385501	-0.392259
chr14	97023505	97023602	[redacted]	-0.385376	-0.388261
chr7	20083160	20083211	[redacted]	-0.385359	-0.37485
chr17	78507241	78507325	[redacted]	-0.385335	-0.362857
chr18	55144288	55144339	[redacted]	-0.385302	-0.388593
chr9	109600690	109600756	[redacted]	-0.385231	-0.392655
chr9	98695093	98695130	[redacted]	-0.385214	-0.383336
chr11	118566111	118566163	[redacted]	-0.384829	-0.34749
chr3	69244413	69244442	[redacted]	-0.384824	-0.470692
chr9	74135055	74135114	[redacted]	-0.384743	-0.441621
chr14	106665915	106665991	[redacted]	-0.384684	-0.408621
chr19	61150677	61150736	[redacted]	-0.384504	-0.387093
chr15	82474402	82474467	[redacted]	-0.384317	-0.351615
chr12	67623575	67623606	[redacted]	-0.384179	-0.381375
chr1	77004898	77004933	[redacted]	-0.384164	-0.388434
chr19	6654624	6654730	[redacted]	-0.384151	-0.305888
chr5	36216493	36216554	[redacted]	-0.384125	-0.311793
chr1	168890326	168890343	[redacted]	-0.384072	-0.386625
chr11	67230685	67230741	[redacted]	-0.383907	-0.343944
chr10	126014113	126014163	[redacted]	-0.383848	-0.71325
chr7	89930782	89930824	[redacted]	-0.383848	-0.368807
chrX	141730228	141730243	[redacted]	-0.383788	-0.402912
chr14	6274114	6274190	[redacted]	-0.383668	-0.380326
chr1	177918954	177919024	[redacted]	-0.383629	-0.320537
chr16	70575214	70575259	[redacted]	-0.383444	-0.45112
chr18	63433044	63433102	[redacted]	-0.383405	-0.363847
chrX	82420299	82420323	[redacted]	-0.383333	-0.351091
chrX	53475277	53475388	[redacted]	-0.383258	-0.67101
chr8	41215764	41215812	[redacted]	-0.383117	-0.384563
chr12	74703926	74703986	[redacted]	-0.38306	-0.365278
chr2	122828570	122828633	[redacted]	-0.38303	-0.355126
chr18	67324670	67324716	[redacted]	-0.383021	-0.310493
chr1	77884907	77884921	[redacted]	-0.382981	-0.419453
chr4	124678675	124678707	[redacted]	-0.382958	-0.378652
chr1	64054571	64054626	[redacted]	-0.38275	-0.368304
chr2	48916609	48916664	[redacted]	-0.382659	-0.369326
chr1	74809922	74809998	[redacted]	-0.382533	-0.369496
chr2	101137381	101137431	[redacted]	-0.382522	-0.464898
chr16	41184871	41184954	[redacted]	-0.382397	-0.38003

chr15	31833006	31833080	[redacted]	-0.382393	-0.364885
chr16	5154367	5154466	[redacted]	-0.382325	-0.368713
chr18	3015256	3015311	[redacted]	-0.382228	-0.467089
chr3	84092553	84092621	[redacted]	-0.38208	-0.329439
chr1	179287696	179287734	[redacted]	-0.382052	-0.361525
chr17	51614702	51614761	[redacted]	-0.381899	-0.375851
chr17	37422850	37422904	[redacted]	-0.381863	-0.354258
chr1	114712440	114712510	[redacted]	-0.381718	-0.420966
chr1	160383037	160383078	[redacted]	-0.38164	-0.438669
chr11	95649795	95649814	[redacted]	-0.381622	-0.308392
chr3	5226972	5227039	[redacted]	-0.381617	-0.331156
chr1	13727139	13727279	[redacted]	-0.38151	-0.311344
chr11	55645231	55645292	[redacted]	-0.381506	-0.36432
chr3	34338294	34338375	[redacted]	-0.381481	-0.461254
chr8	24758091	24758131	[redacted]	-0.381395	-0.376954
chr14	48813513	48813559	[redacted]	-0.381362	-0.387413
chr17	69000558	69000592	[redacted]	-0.381194	-0.368833
chr17	72424013	72424069	[redacted]	-0.381064	-0.373793
chr3	41497462	41497494	[redacted]	-0.381014	-0.346652
chr5	38456564	38456638	[redacted]	-0.380932	-0.38268
chr18	71934347	71934412	[redacted]	-0.380833	-0.339838
chr18	52534940	52534989	[redacted]	-0.380802	-0.348982
chr12	89491468	89491543	[redacted]	-0.380782	-0.311282
chr7	18090391	18090422	[redacted]	-0.380741	-0.410992
chr6	85408182	85408248	[redacted]	-0.380675	-0.453616
chr6	58491101	58491176	[redacted]	-0.380627	-0.377014
chr13	39811877	39811935	[redacted]	-0.380617	-0.324038
chr2	58799344	58799405	[redacted]	-0.380602	-0.385461
chr17	33970134	33970158	[redacted]	-0.380483	-0.311387
chr8	24611118	24611204	[redacted]	-0.38047	-0.404553
chr9	68774491	68774532	[redacted]	-0.380153	-0.460301
chr16	84250740	84250768	[redacted]	-0.380141	-0.536701
chr11	23981687	23981743	[redacted]	-0.379835	-0.416443
chr12	84653359	84653421	[redacted]	-0.379813	-0.447331
chr14	93770513	93770581	[redacted]	-0.379727	-0.373854
chr13	69246203	69246251	[redacted]	-0.379648	-0.349968
chr17	87952466	87952517	[redacted]	-0.379627	-0.345145
chr12	93472916	93472996	[redacted]	-0.379583	-0.41546
chr3	103464565	103464653	[redacted]	-0.37947	-0.328709
chr14	58634693	58634758	[redacted]	-0.379465	-0.311731
chr14	18912149	18912224	[redacted]	-0.37944	-0.389005
chr19	21550169	21550220	[redacted]	-0.3794	-0.420039
chr7	50255303	50255343	[redacted]	-0.379319	-0.423423

chr14	54609980	54610043	[redacted]	-0.379202	-0.387924
chr14	112387284	112387335	[redacted]	-0.37912	-0.490336
chr3	139653003	139653054	[redacted]	-0.379015	-0.45321
chrX	164512217	164512308	[redacted]	-0.378991	-0.401696
chr13	118216946	118217044	[redacted]	-0.378982	-0.454606
chr15	72372671	72372727	[redacted]	-0.378916	-0.378177
chr6	8519910	8519918	[redacted]	-0.378756	-0.398658
chr12	92073223	92073259	[redacted]	-0.378726	-0.405507
chr5	96050962	96051021	[redacted]	-0.378635	-0.363808
chr2	98970042	98970105	[redacted]	-0.378612	-0.311298
chr19	21985658	21985716	[redacted]	-0.378608	-0.341429
chr3	133141610	133141650	[redacted]	-0.378491	-0.327529
chr11	100996652	100996710	[redacted]	-0.378405	-0.353208
chr12	79041129	79041165	[redacted]	-0.378404	-0.485082
chr9	71050814	71050869	[redacted]	-0.378404	-0.403415
chr12	80738973	80739022	[redacted]	-0.378345	-0.547084
chr6	137392581	137392637	[redacted]	-0.378342	-0.365942
chr17	83290841	83290860	[redacted]	-0.378285	-0.37017
chr10	56937086	56937137	[redacted]	-0.378264	-0.316872
chr15	57621651	57621687	[redacted]	-0.378252	-0.408499
chr15	41796637	41796680	[redacted]	-0.378186	-0.407655
chr2	108373727	108373806	[redacted]	-0.378068	-0.419823
chr19	32478028	32478049	[redacted]	-0.377884	-0.433005
chr8	75564081	75564175	[redacted]	-0.377882	-0.40142
chr16	44685747	44685796	[redacted]	-0.377882	-0.382821
chr7	97064712	97064794	[redacted]	-0.377838	-0.322544
chr2	19156499	19156542	[redacted]	-0.377815	-0.357783
chr12	92695406	92695464	[redacted]	-0.377788	-0.383636
chr7	19172065	19172105	[redacted]	-0.37763	-0.401758
chr14	55460298	55460318	[redacted]	-0.377591	-0.465316
chr14	90313174	90313207	[redacted]	-0.377577	-0.360449
chr13	96423747	96423787	[redacted]	-0.377527	-0.374602
chr14	46236141	46236215	[redacted]	-0.377523	-0.360406
chr3	69184683	69184729	[redacted]	-0.377472	-0.30558
chr14	73370650	73370707	[redacted]	-0.377468	-0.319429
chr8	52141937	52141997	[redacted]	-0.377468	-0.411676
chr4	6883095	6883140	[redacted]	-0.377466	-0.380175
chr8	31278183	31278288	[redacted]	-0.377397	-0.382095
chr9	56260755	56260852	[redacted]	-0.377363	-0.33344
chr4	106427298	106427322	[redacted]	-0.377311	-0.378209
chr7	72024392	72024425	[redacted]	-0.37709	-0.411132
chr6	131177845	131177898	[redacted]	-0.377035	-0.439527
chr5	105914862	105914925	[redacted]	-0.377035	-0.359387

chr2	174673270	174673316	[redacted]	-0.376993	-0.461745
chr14	41201904	41202024	[redacted]	-0.376882	-0.390784
chr3	123580200	123580268	[redacted]	-0.376683	-0.363578
chr2	48162759	48162827	[redacted]	-0.376369	-0.493728
chr11	42680320	42680364	[redacted]	-0.376329	-0.415994
chr5	104520893	104520950	[redacted]	-0.376223	-0.457403
chr7	54320217	54320281	[redacted]	-0.376222	-0.352176
chr17	73671181	73671226	[redacted]	-0.376213	-0.343447
chr17	47610163	47610236	[redacted]	-0.376162	-0.337861
chr6	76368251	76368300	[redacted]	-0.375936	-0.361086
chr6	143465762	143465868	[redacted]	-0.375842	-0.401205
chr3	140027897	140027931	[redacted]	-0.375739	-0.383073
chr13	115153031	115153108	[redacted]	-0.375666	-0.36748
chr17	88806362	88806393	[redacted]	-0.375632	-0.358139
chr2	57265546	57265606	[redacted]	-0.375461	-0.358266
chr2	14292510	14292565	[redacted]	-0.375367	-0.416515
chr12	27028748	27028816	[redacted]	-0.375246	-0.41703
chr1	91162634	91162681	[redacted]	-0.37521	-0.359117
chr16	27573821	27573879	[redacted]	-0.37516	-0.318166
chr11	59310022	59310043	[redacted]	-0.375102	-0.303583
chr1	128343321	128343380	[redacted]	-0.375088	-0.377998
chr15	49337811	49337918	[redacted]	-0.374942	-0.399147
chr6	51253538	51253615	[redacted]	-0.37478	-0.364846
chr4	46270965	46271024	[redacted]	-0.374772	-0.427891
chr2	117923689	117923809	[redacted]	-0.374673	-0.387355
chr11	9749341	9749395	[redacted]	-0.374663	-0.318799
chr9	45641146	45641193	[redacted]	-0.374633	-0.367324
chr6	24249986	24250101	[redacted]	-0.374612	-0.340797
chr1	117904704	117904740	[redacted]	-0.374485	-0.390395
chr10	88874986	88875048	[redacted]	-0.374361	-0.578802
chr16	59457052	59457100	[redacted]	-0.374356	-0.472812
chr2	121616564	121616608	[redacted]	-0.374194	-0.359824
chr15	52123196	52123265	[redacted]	-0.374188	-0.393658
chr6	137930169	137930217	[redacted]	-0.373887	-0.383742
chr9	67398402	67398456	[redacted]	-0.373848	-0.380347
chr4	124420555	124420650	[redacted]	-0.373788	-0.301206
chr17	8595591	8595633	[redacted]	-0.373731	-0.392052
chr4	24813690	24813719	[redacted]	-0.373684	-0.392071
chrX	72278312	72278334	[redacted]	-0.373532	-0.361197
chr11	48347468	48347525	[redacted]	-0.373486	-0.301805
chr12	41710045	41710094	[redacted]	-0.373461	-0.401936
chr17	49106115	49106168	[redacted]	-0.373399	-0.530034
chr1	117852847	117852912	[redacted]	-0.373373	-0.404648

chr11	89577669	89577722	[redacted]	-0.37335	-0.361121
chr2	154954627	154954761	[redacted]	-0.373268	-0.34675
chr7	45656037	45656066	[redacted]	-0.373262	-0.331578
chr12	40733811	40733903	[redacted]	-0.373201	-0.302732
chr6	86705063	86705081	[redacted]	-0.373186	-0.366059
chr15	96227728	96227773	[redacted]	-0.373177	-0.423643
chr3	127462384	127462417	[redacted]	-0.373167	-0.396717
chr14	87365605	87365656	[redacted]	-0.373085	-0.334183
chr5	29985655	29985690	[redacted]	-0.373052	-0.340171
chr11	108848141	108848172	[redacted]	-0.373025	-0.383307
chr11	95533271	95533325	[redacted]	-0.372999	-0.312035
chr17	63571267	63571328	[redacted]	-0.372932	-0.358263
chr1	124716453	124716543	[redacted]	-0.372904	-0.546391
chr18	82329731	82329782	[redacted]	-0.37285	-0.520873
chr2	72193072	72193112	[redacted]	-0.372815	-0.367871
chr10	44730726	44730762	[redacted]	-0.372772	-0.375574
chr16	36160002	36160065	[redacted]	-0.372674	-0.354802
chr8	11430559	11430614	[redacted]	-0.372606	-0.48517
chr7	91531226	91531278	[redacted]	-0.372533	-0.314279
chr6	141437601	141437683	[redacted]	-0.37237	-0.439082
chr9	44125211	44125217	[redacted]	-0.372367	-0.368101
chr12	72145001	72145026	[redacted]	-0.372326	-0.401492
chrX	13797099	13797178	[redacted]	-0.372323	-0.331251
chr1	145195345	145195424	[redacted]	-0.372296	-0.338549
chr19	42450896	42450944	[redacted]	-0.372219	-0.393054
chr12	112708436	112708490	[redacted]	-0.372184	-0.33335
chr19	22255463	22255490	[redacted]	-0.37209	-0.369476
chr19	26380930	26380972	[redacted]	-0.372089	-0.349052
chr8	11189968	11190001	[redacted]	-0.371872	-0.496221
chr1	173465757	173465797	[redacted]	-0.37184	-0.378655
chr17	88668040	88668247	[redacted]	-0.371823	-0.552503
chr1	74762592	74762627	[redacted]	-0.371804	-0.55502
chr14	74194642	74194721	[redacted]	-0.371734	-0.347376
chr14	46933646	46933698	[redacted]	-0.371717	-0.359553
chr11	58655314	58655383	[redacted]	-0.371653	-0.390705
chr1	15578556	15578601	[redacted]	-0.37164	-0.379827
chr10	68035667	68035710	[redacted]	-0.371574	-0.376869
chr11	89832264	89832284	[redacted]	-0.371569	-0.413782
chr18	51851928	51851961	[redacted]	-0.371455	-0.394725
chr12	11138311	11138362	[redacted]	-0.371419	-0.405119
chr6	132691648	132691711	[redacted]	-0.371347	-0.546973
chr19	30642271	30642350	[redacted]	-0.371185	-0.317935
chr4	115633515	115633568	[redacted]	-0.371174	-0.34544

chr9	105461855	105461898	[redacted]	-0.371078	-0.400757
chr5	115681340	115681407	[redacted]	-0.37105	-0.327469
chr2	71169660	71169703	[redacted]	-0.370994	-0.364653
chr16	34621324	34621406	[redacted]	-0.370905	-0.320804
chr13	103409124	103409192	[redacted]	-0.370809	-0.381559
chr6	131968919	131969004	[redacted]	-0.370778	-0.45707
chr10	101450862	101450986	[redacted]	-0.370745	-0.3226
chr17	78566306	78566329	[redacted]	-0.370727	-0.364203
chr8	18565326	18565358	[redacted]	-0.370722	-0.508003
chr1	177926959	177927077	[redacted]	-0.370575	-0.39521
chr12	116263360	116263468	[redacted]	-0.370545	-0.340572
chr16	94968050	94968093	[redacted]	-0.370509	-0.475881
chr7	90063092	90063136	[redacted]	-0.370467	-0.302425
chr12	91476254	91476314	[redacted]	-0.370404	-0.304868
chr8	41624877	41624898	[redacted]	-0.370304	-0.39236
chr11	21896441	21896485	[redacted]	-0.370286	-0.301385
chr13	54424148	54424236	[redacted]	-0.370248	-0.318998
chr5	138712127	138712190	[redacted]	-0.370153	-0.338795
chr1	170035986	170036034	[redacted]	-0.369454	-0.435533
chr18	50605033	50605110	[redacted]	-0.369365	-0.355768
chrX	48074543	48074584	[redacted]	-0.369355	-0.418946
chr3	89420175	89420215	[redacted]	-0.369337	-0.487561
chr17	90321853	90321858	[redacted]	-0.369244	-0.370236
chr18	63836510	63836588	[redacted]	-0.36922	-0.325492
chr6	12518453	12518468	[redacted]	-0.369204	-0.313015
chr17	5585561	5585602	[redacted]	-0.369141	-0.350888
chr12	79118394	79118461	[redacted]	-0.369078	-0.31649
chr12	111330211	111330296	[redacted]	-0.368921	-0.3669
chr15	93643356	93643476	[redacted]	-0.368858	-0.378104
chr13	97501662	97501743	[redacted]	-0.368774	-0.319097
chr15	53744454	53744514	[redacted]	-0.368732	-0.379096
chr7	113658580	113658623	[redacted]	-0.368611	-0.316072
chr18	17558126	17558141	[redacted]	-0.368583	-0.407895
chr11	5700450	5700467	[redacted]	-0.36858	-0.370261
chr11	68934173	68934242	[redacted]	-0.368566	-0.363936
chr7	70700905	70700968	[redacted]	-0.36856	-0.342661
chr2	151407583	151407651	[redacted]	-0.368532	-0.348444
chr5	89819393	89819485	[redacted]	-0.368522	-0.327522
chr18	32164272	32164358	[redacted]	-0.368465	-0.303288
chr10	47718386	47718428	[redacted]	-0.36845	-0.336185
chr2	12882474	12882538	[redacted]	-0.368364	-0.359375
chr5	147579154	147579184	[redacted]	-0.368229	-0.35886
chr13	33098195	33098265	[redacted]	-0.368085	-0.322227

chr3	71909209	71909283	[redacted]	-0.367967	-0.316739
chr7	44248663	44248721	[redacted]	-0.367967	-0.425701
chrX	128086443	128086491	[redacted]	-0.367892	-0.4335
chr1	191414543	191414576	[redacted]	-0.367868	-0.599862
chr18	69271460	69271580	[redacted]	-0.36784	-0.348705
chr1	164602233	164602289	[redacted]	-0.36781	-0.341391
chr14	114489773	114489823	[redacted]	-0.367695	-0.363618
chr8	41257880	41257901	[redacted]	-0.36769	-0.366413
chr14	36794943	36795003	[redacted]	-0.367682	-0.383675
chr8	18543997	18544044	[redacted]	-0.367659	-0.361563
chr9	89702066	89702098	[redacted]	-0.367609	-0.365213
chr1	75385349	75385392	[redacted]	-0.367596	-0.433807
chr4	12351319	12351402	[redacted]	-0.367572	-0.302302
chr5	9464034	9464117	[redacted]	-0.367326	-0.323631
chr9	69316009	69316082	[redacted]	-0.367305	-0.307933
chr19	3693252	3693266	[redacted]	-0.367249	-0.348855
chr12	20674698	20674755	[redacted]	-0.367153	-0.366705
chr16	96814332	96814416	[redacted]	-0.367084	-0.357171
chr7	89422257	89422345	[redacted]	-0.367017	-0.313953
chr15	40235983	40236091	[redacted]	-0.366938	-0.328053
chr2	10284141	10284192	[redacted]	-0.366887	-0.306616
chr5	135123452	135123530	[redacted]	-0.366784	-0.300488
chr8	89000327	89000338	[redacted]	-0.366758	-0.346157
chr11	38233300	38233351	[redacted]	-0.366639	-0.302093
chr18	6182602	6182702	[redacted]	-0.366558	-0.31721
chr18	9945990	9946043	[redacted]	-0.366552	-0.303451
chr1	156198242	156198312	[redacted]	-0.366511	-0.324886
chr4	53824929	53824990	[redacted]	-0.366495	-0.40643
chr14	93806357	93806390	[redacted]	-0.366444	-0.429119
chr12	118432856	118432891	[redacted]	-0.366386	-0.321288
chr14	23557775	23557854	[redacted]	-0.366381	-0.361944
chr15	58863908	58863971	[redacted]	-0.366314	-0.333406
chr12	84971645	84971696	[redacted]	-0.366299	-0.404548
chr15	41187121	41187200	[redacted]	-0.366203	-0.362597
chr4	130647559	130647591	[redacted]	-0.366192	-0.395477
chr18	64203545	64203598	[redacted]	-0.366191	-0.494141
chr19	31497499	31497518	[redacted]	-0.366191	-0.368906
chr7	72911484	72911547	[redacted]	-0.366107	-0.359735
chr17	63823149	63823197	[redacted]	-0.366047	-0.327655
chr11	66523673	66523775	[redacted]	-0.365768	-0.422443
chr17	64230381	64230451	[redacted]	-0.36571	-0.365673
chr6	135264694	135264736	[redacted]	-0.365699	-0.4094
chr8	14971714	14971752	[redacted]	-0.365566	-0.361703

chr15	9965688	9965771		-0.365525	-0.356225
chr5	124900452	124900503		-0.365382	-0.318662
chr4	62817847	62817868		-0.36518	-0.302254
chr1	164367338	164367406		-0.365033	-0.363876
chr17	28550376	28550436		-0.364954	-0.312309
chr6	42795420	42795504		-0.364907	-0.41361
chr10	26482766	26482780		-0.364904	-0.362023
chr19	37034616	37034685		-0.364877	-0.307122
chr11	6834723	6834765		-0.364871	-0.321216
chr8	7204735	7204831		-0.364861	-0.411105
chr17	84723096	84723152		-0.364844	-0.383704
chr9	33211920	33211969		-0.3648	-0.386743
chr15	9795931	9796006		-0.364783	-0.33947
chr9	115442979	115443177		-0.364703	-0.321874
chr15	98495474	98495533		-0.364626	-0.375657
chr3	41414983	41415062		-0.364611	-0.431811
chr13	9244624	9244695		-0.36456	-0.326652
chr7	122586824	122586859		-0.364474	-0.393905
chr9	39647379	39647432		-0.364473	-0.32012
chr8	48676478	48676500		-0.364442	-0.380445
chr8	39723195	39723228		-0.36426	-0.323998
chr18	57309739	57309808		-0.364246	-0.306567
chrX	78818038	78818098		-0.364064	-0.328812
chr7	135395834	135395931		-0.364046	-0.30472
chr10	67489620	67489688		-0.364034	-0.343857
chr16	45337284	45337360		-0.363817	-0.321713
chrX	111216536	111216616		-0.363754	-0.369707
chr1	52487696	52487738		-0.363744	-0.309838
chr14	106135039	106135080		-0.363717	-0.376795
chr10	96610022	96610107		-0.363688	-0.381817
chr14	53534831	53534905		-0.363573	-0.392892
chr12	80165295	80165431		-0.363409	-0.401487
chr11	29676734	29676801		-0.363398	-0.331449
chr4	114915350	114915426		-0.363398	-0.691708
chr7	78552578	78552630		-0.363387	-0.372735
chr16	76477616	76477664		-0.363303	-0.338811
chr10	6328878	6328929		-0.36329	-0.43881
chr16	55969926	55969964		-0.363243	-0.422466
chr18	23489081	23489133		-0.363202	-0.319641
chr3	41332515	41332606		-0.363091	-0.328505
chr11	36165101	36165157		-0.363085	-0.384906
chr17	86833203	86833264		-0.362936	-0.320206
chr17	59223638	59223705		-0.3627	-0.349459

chr7	112591448	112591516		-0.362698	-0.372101
chr7	45663940	45664005		-0.362691	-0.350629
chr17	65246922	65246966		-0.362667	-0.545379
chr12	99758302	99758312		-0.362655	-0.366583
chr9	69079298	69079361		-0.362645	-0.34252
chr17	28240405	28240434		-0.362622	-0.361172
chr9	75353278	75353306		-0.36257	-0.313185
chr3	130923574	130923602		-0.362568	-0.30094
chr11	67558934	67558976		-0.362544	-0.497893
chr3	73589150	73589199		-0.362427	-0.385493
chr8	38421473	38421591		-0.362409	-0.407031
chr2	69251538	69251585		-0.362343	-0.460062
chr10	121366607	121366639		-0.36227	-0.35554
chr3	147076026	147076087		-0.362136	-0.403322
chr7	55911483	55911533		-0.362074	-0.339389
chr15	69556167	69556230		-0.362068	-0.329747
chr2	112142687	112142769		-0.362036	-0.387808
chr3	134983587	134983617		-0.361982	-0.368063
chr12	10163074	10163123		-0.361981	-0.385908
chr3	54332632	54332696		-0.361952	-0.369226
chr2	137919936	137919991		-0.361911	-0.407832
chr8	107906503	107906566		-0.361881	-0.389014
chr5	96495528	96495579		-0.361856	-0.369867
chr6	26438385	26438438		-0.361823	-0.434062
chr17	55626392	55626527		-0.361794	-0.306305
chr2	25747115	25747199		-0.361758	-0.458005
chr2	70412207	70412260		-0.361731	-0.500863
chr17	72930783	72930813		-0.361656	-0.36478
chr1	96105002	96105070		-0.361628	-0.374407
chr18	70866325	70866368		-0.361587	-0.375298
chr1	161687069	161687104		-0.361341	-0.38554
chr18	88948084	88948145		-0.361316	-0.50135
chr4	146739504	146739568		-0.361309	-0.325943
chr12	99001469	99001534		-0.361295	-0.37805
chr3	131486442	131486515		-0.36125	-0.357513
chr13	75937712	75937796		-0.361214	-0.304499
chr10	56421623	56421701		-0.361176	-0.316632
chrX	38844065	38844133		-0.361046	-0.561976
chr6	7579533	7579600		-0.361006	-0.445308
chr9	117868634	117868670		-0.360899	-0.460955
chr12	49239418	49239468		-0.360889	-0.306192
chr11	5494972	5495003		-0.360887	-0.374451
chr7	81732618	81732631		-0.360886	-0.416704

chr4	30292919	30292950		-0.360831	-0.398904
chr12	5134422	5134469		-0.360689	-0.375795
chr1	109584876	109584931		-0.360664	-0.367739
chr11	33033899	33033979		-0.360595	-0.473408
chr1	96439640	96439694		-0.360559	-0.372792
chr1	174667393	174667443		-0.360505	-0.38015
chr17	90449888	90449931		-0.3605	-0.447049
chr10	120237436	120237484		-0.360476	-0.35024
chr9	74442788	74442833		-0.360375	-0.319049
chr1	105823685	105823727		-0.360371	-0.360919
chr17	39834572	39834623		-0.360356	-0.613432
chr15	87458509	87458518		-0.360331	-0.497126
chr5	144291294	144291368		-0.360274	-0.313071
chr11	77040398	77040430		-0.36	-0.338115
chr14	22784473	22784525		-0.359975	-0.324584
chr6	140192777	140192889		-0.359954	-0.347175
chr9	71260430	71260463		-0.35994	-0.372171
chr4	54208459	54208547		-0.3599	-0.408598
chr14	88060319	88060368		-0.359885	-0.316337
chr8	60373362	60373414		-0.359878	-0.41855
chr14	123396156	123396196		-0.35964	-0.305462
chr3	11943863	11943909		-0.359625	-0.387533
chr10	87592616	87592669		-0.35953	-0.343828
chr13	45165648	45165685		-0.359528	-0.434528
chr4	133112400	133112444		-0.359504	-0.586041
chr13	113106821	113106847		-0.359427	-0.362975
chr11	80088922	80088957		-0.35942	-0.336371
chr13	43090305	43090369		-0.359382	-0.353007
chr11	74295466	74295538		-0.359367	-0.486817
chr8	21677917	21677938		-0.35936	-0.529688
chr2	168709626	168709651		-0.359279	-0.338323
chr11	18911240	18911284		-0.3592	-0.362507
chr10	125776500	125776561		-0.359178	-0.347864
chr13	41131149	41131203		-0.359161	-0.348464
chr5	90905931	90905998		-0.35916	-0.353395
chr8	92298711	92298774		-0.359149	-0.404911
chr19	53071656	53071691		-0.359066	-0.45852
chr1	174648993	174649033		-0.359019	-0.403242
chr17	72897337	72897391		-0.358903	-0.35088
chr13	116829053	116829138		-0.358863	-0.417763
chr3	142945004	142945068		-0.358825	-0.314036
chr6	129930245	129930293		-0.358788	-0.331759
chr8	43237273	43237326		-0.358718	-0.439347

chr12	75967295	75967374	[redacted]	-0.358554	-0.390446
chr8	59864459	59864522	[redacted]	-0.358518	-0.333089
chr6	112110625	112110674	[redacted]	-0.358452	-0.408327
chr11	14905940	14905990	[redacted]	-0.358422	-0.36618
chr17	67249865	67249905	[redacted]	-0.35836	-0.359507
chr1	144401036	144401104	[redacted]	-0.358347	-0.323536
chr6	30549968	30550010	[redacted]	-0.358257	-0.528952
chr3	56712820	56712849	[redacted]	-0.358234	-0.372287
chr9	84924589	84924657	[redacted]	-0.35813	-0.387024
chr16	76500198	76500258	[redacted]	-0.358116	-0.374002
chr15	87068199	87068213	[redacted]	-0.358046	-0.34743
chr3	105152986	105153044	[redacted]	-0.358037	-0.323257
chr7	36457789	36457825	[redacted]	-0.357976	-0.412592
chr13	48318535	48318583	[redacted]	-0.357907	-0.309472
chr2	126216799	126216878	[redacted]	-0.357876	-0.325339
chr10	20280735	20280801	[redacted]	-0.357766	-0.305366
chr13	53923039	53923106	[redacted]	-0.357651	-0.40829
chr14	86770044	86770079	[redacted]	-0.357645	-0.380541
chr2	75539925	75539940	[redacted]	-0.357628	-0.383239
chr1	162905248	162905304	[redacted]	-0.357613	-0.303234
chr14	121786214	121786235	[redacted]	-0.357609	-0.34378
chr17	62566312	62566388	[redacted]	-0.357495	-0.354047
chr14	6919962	6919989	[redacted]	-0.35748	-0.408867
chr3	97200890	97200972	[redacted]	-0.357464	-0.374514
chr8	104963443	104963495	[redacted]	-0.357273	-0.388539
chr9	10208452	10208534	[redacted]	-0.357257	-0.458181
chr9	111906990	111907098	[redacted]	-0.357251	-0.317123
chr14	8736825	8736905	[redacted]	-0.357241	-0.355422
chr11	18332794	18332828	[redacted]	-0.357234	-0.348318
chr9	25103711	25103774	[redacted]	-0.357066	-0.355444
chr17	77419806	77419857	[redacted]	-0.357065	-0.379049
chr8	69136341	69136358	[redacted]	-0.357038	-0.417716
chr15	100766517	100766537	[redacted]	-0.356836	-0.345101
chr17	19322653	19322659	[redacted]	-0.356735	-0.307054
chr12	72539452	72539496	[redacted]	-0.356722	-0.313302
chr5	114933262	114933305	[redacted]	-0.356676	-0.320837
chr2	134793328	134793424	[redacted]	-0.356669	-0.386003
chr4	6859964	6859984	[redacted]	-0.356665	-0.397717
chr2	164031387	164031466	[redacted]	-0.356604	-0.420403
chr4	104272559	104272605	[redacted]	-0.356368	-0.378305
chr15	51730155	51730260	[redacted]	-0.356279	-0.39319
chr10	78421889	78421908	[redacted]	-0.356258	-0.472711
chr4	30429608	30429661	[redacted]	-0.356184	-0.498661

chr15	84041615	84041701		-0.35613	-0.306401
chr8	58203268	58203329		-0.356118	-0.397434
chr17	42500882	42500953		-0.355995	-0.325863
chr14	94409913	94409960		-0.355942	-0.42162
chr1	36984046	36984077		-0.355919	-0.317921
chr6	136782321	136782391		-0.355896	-0.325908
chr12	25045996	25046052		-0.355887	-0.345239
chr14	65704834	65704843		-0.355765	-0.395958
chr13	44615703	44615761		-0.355753	-0.376565
chr1	118400278	118400331		-0.355623	-0.382157
chr12	60256266	60256306		-0.355512	-0.357255
chr10	98433573	98433643		-0.355441	-0.445597
chr17	26317052	26317098		-0.355415	-0.404074
chr14	16672024	16672112		-0.355358	-0.305463
chr11	21870813	21870847		-0.355205	-0.354938
chr3	41415592	41415628		-0.355093	-0.30121
chr10	39315212	39315245		-0.355083	-0.403263
chr3	119648023	119648044		-0.355039	-0.504903
chr16	64970790	64970894		-0.35497	-0.368704
chr13	71263847	71263895		-0.354942	-0.352673
chr9	41651553	41651577		-0.354845	-0.406451
chr7	69288934	69288976		-0.354783	-0.324419
chr7	80474815	80474840		-0.354725	-0.316821
chr5	92317409	92317480		-0.354647	-0.304357
chr17	31212849	31212905		-0.35457	-0.401098
chr1	147273094	147273142		-0.354529	-0.358175
chr10	96854956	96854995		-0.354459	-0.434709
chr8	87149810	87149831		-0.35431	-0.396446
chr10	129183859	129183938		-0.354257	-0.42371
chr13	28293890	28293974		-0.354212	-0.330336
chr8	96093544	96093578		-0.354188	-0.478547
chr7	125324295	125324380		-0.354156	-0.36316
chr9	21932974	21933015		-0.354123	-0.414411
chr9	22443374	22443420		-0.354118	-0.354663
chr9	106974033	106974069		-0.354113	-0.36214
chr16	45942693	45942768		-0.354081	-0.445961
chr3	53818461	53818527		-0.353967	-0.314927
chr6	100873675	100873686		-0.353957	-0.430422
chr2	104874076	104874146		-0.353862	-0.415391
chr3	142895380	142895396		-0.353861	-0.343816
chr12	69435567	69435630		-0.353846	-0.317205
chr14	68224914	68224989		-0.353801	-0.395207
chr16	45103052	45103071		-0.353774	-0.407378

chr3	100770624	100770657		-0.353746	-0.302124
chr11	44934153	44934263		-0.353734	-0.316747
chr14	99665870	99665938		-0.353615	-0.345127
chr11	52003324	52003388		-0.353508	-0.398141
chr5	45242262	45242371		-0.353456	-0.337964
chr5	79868033	79868067		-0.353451	-0.313474
chr13	62340229	62340256		-0.353332	-0.389944
chr19	41843549	41843583		-0.353253	-0.301137
chr13	18708617	18708696		-0.353181	-0.397288
chr6	12912120	12912203		-0.353148	-0.34445
chr14	60346789	60346843		-0.353096	-0.441341
chr16	57573520	57573541		-0.353075	-0.329396
chr15	28004387	28004420		-0.353013	-0.371991
chr8	30721587	30721617		-0.352945	-0.361573
chr1	74196299	74196361		-0.352923	-0.304016
chr6	134960286	134960348		-0.352667	-0.346996
chr3	63765547	63765561		-0.352661	-0.395928
chr6	56924576	56924634		-0.352624	-0.407193
chr8	100356008	100356034		-0.352571	-0.374685
chr15	60226837	60226893		-0.352509	-0.37667
chr12	92367075	92367130		-0.352427	-0.368557
chr1	84972737	84972796		-0.352304	-0.334008
chr8	86355584	86355626		-0.35223	-0.437175
chr7	142752436	142752455		-0.352209	-0.460691
chr5	132383991	132384075		-0.352037	-0.336804
chr5	65505643	65505685		-0.351998	-0.360396
chr12	41875854	41875879		-0.351942	-0.380346
chr12	70914674	70914728		-0.351839	-0.301476
chr18	22824004	22824088		-0.351802	-0.415307
chr15	37803694	37803764		-0.351789	-0.356557
chr2	50688096	50688167		-0.351696	-0.335067
chr1	65640136	65640181		-0.351626	-0.426812
chr1	8043028	8043052		-0.351509	-0.386361
chr12	56358980	56359048		-0.351431	-0.380823
chr18	6343887	6343943		-0.351394	-0.325937
chr17	11792899	11792947		-0.351264	-0.35125
chr16	58519032	58519069		-0.351181	-0.405612
chr15	44352892	44352975		-0.351163	-0.4401
chr9	28869611	28869676		-0.351122	-0.366279
chr10	127204176	127204195		-0.351083	-0.379837
chr16	87560511	87560601		-0.351082	-0.399519
chr1	168445622	168445696		-0.351073	-0.356778
chr9	46482687	46482728		-0.351064	-0.304771

chr18	49641805	49641866		-0.351044	-0.312121
chr6	78168013	78168070		-0.351028	-0.373894
chr2	170636016	170636099		-0.351018	-0.390792
chr9	19013997	19014035		-0.351004	-0.329969
chr11	98275514	98275567		-0.350881	-0.375033
chr4	27571884	27571917		-0.350687	-0.458263
chr5	104337933	104337986		-0.350682	-0.427601
chr13	11291740	11291766		-0.350676	-0.451353
chr1	63602919	63602995		-0.350675	-0.423664
chr3	138366852	138366925		-0.350633	-0.382663
chr10	63412979	63412996		-0.350482	-0.465784
chr7	49622784	49622850		-0.350427	-0.363415
chr1	71990733	71990812		-0.350323	-0.360577
chr12	104907928	104907950		-0.350202	-0.324528
chr2	54012277	54012327		-0.350201	-0.422886
chr1	50438872	50438942		-0.350134	-0.338531
chr4	58865337	58865372		-0.350059	-0.380842
chr9	121414414	121414501		-0.349961	-0.456858
chr6	143656930	143657007		-0.349906	-0.332411
chr9	101264759	101264882		-0.349895	-0.31254
chr18	12492165	12492233		-0.349778	-0.371033
chr3	130097328	130097357		-0.349672	-0.367916
chr13	10318007	10318064		-0.349583	-0.399373
chr6	137530379	137530427		-0.349447	-0.313574
chr2	3333364	3333413		-0.349384	-0.399186
chr3	128557228	128557281		-0.349336	-0.306952
chr5	106166154	106166246		-0.349276	-0.361204
chr11	43879285	43879339		-0.349256	-0.34317
chr11	29244498	29244535		-0.349246	-0.355025
chr6	97702944	97703018		-0.348984	-0.36155
chr8	66631840	66631889		-0.3489	-0.349736
chr16	36769855	36770014		-0.348771	-0.379134
chr2	156607896	156607954		-0.34867	-0.335613
chr17	71525737	71525795		-0.348652	-0.407004
chr18	76887551	76887631		-0.348642	-0.618073
chr4	99236802	99236847		-0.348623	-0.357484
chr14	121511942	121511967		-0.348556	-0.339962
chr4	6335175	6335212		-0.348504	-0.342582
chrX	94616009	94616096		-0.348374	-0.361455
chr6	127377816	127377835		-0.348304	-0.397241
chr11	44926312	44926331		-0.348285	-0.401063
chr7	111896091	111896128		-0.347999	-0.345883
chr5	148887752	148887847		-0.347986	-0.306253

chr2	71370961	71371015		-0.34782	-0.3196
chr14	83508356	83508426		-0.347777	-0.54468
chr13	40756269	40756295		-0.34773	-0.344386
chr7	34514448	34514486		-0.347608	-0.496614
chr17	28469691	28469727		-0.347489	-0.355482
chr12	30640575	30640618		-0.347359	-0.348153
chrX	43563990	43564049		-0.347281	-0.560268
chr14	93654262	93654317		-0.347272	-0.394284
chr6	39763668	39763705		-0.347266	-0.318201
chr8	82932950	82933062		-0.347242	-0.322917
chr9	78181287	78181337		-0.347186	-0.347345
chrX	66986651	66986732		-0.347126	-0.333802
chr10	122245745	122245807		-0.347041	-0.523727
chr18	14497778	14497818		-0.346957	-0.403626
chr12	42825469	42825504		-0.346955	-0.356736
chr13	118618849	118618911		-0.346955	-0.501233
chr8	64365987	64366089		-0.346894	-0.321161
chr14	48643586	48643605		-0.34689	-0.340728
chr5	77384811	77384948		-0.346886	-0.409952
chr3	139000563	139000656		-0.346878	-0.313675
chr3	65647742	65647809		-0.346837	-0.373244
chr6	139217855	139217903		-0.346773	-0.55496
chr2	83662258	83662281		-0.34663	-0.370777
chr2	5851312	5851367		-0.346414	-0.512969
chr1	164945543	164945564		-0.346143	-0.430848
chr13	99423727	99423811		-0.346078	-0.380276
chr8	41290198	41290243		-0.34599	-0.411201
chr6	22202456	22202553		-0.345641	-0.310574
chr1	48495903	48495974		-0.345627	-0.405136
chr8	33515593	33515638		-0.345506	-0.365856
chr18	40257487	40257557		-0.345486	-0.483531
chr7	23379897	23379936		-0.345479	-0.493089
chr19	43539617	43539704		-0.345434	-0.319462
chr1	183813038	183813106		-0.34541	-0.395993
chr4	49676912	49676939		-0.345396	-0.481163
chr18	46121056	46121120		-0.345232	-0.339805
chr15	82678039	82678135		-0.345176	-0.312034
chr12	37698466	37698523		-0.345064	-0.329746
chr13	77520102	77520133		-0.345062	-0.363562
chr2	135354397	135354457		-0.344978	-0.344409
chr9	82360899	82360997		-0.344968	-0.32775
chr19	29680602	29680620		-0.34492	-0.395234
chr12	29862081	29862206		-0.344864	-0.318943

chrX	109497288	109497358		-0.344683	-0.338334
chr5	52680843	52680918		-0.344647	-0.377036
chr2	173770191	173770336		-0.344526	-0.377054
chr12	103810784	103810810		-0.344521	-0.494784
chr17	62590847	62590945		-0.34451	-0.326766
chr1	51890444	51890475		-0.344459	-0.413994
chr4	26590196	26590253		-0.344367	-0.365334
chr11	108963904	108963949		-0.344319	-0.31644
chr13	98597483	98597532		-0.344303	-0.395533
chr1	172608150	172608206		-0.344192	-0.308218
chr2	164396276	164396338		-0.344101	-0.475404
chr1	64728180	64728311		-0.344016	-0.341324
chr12	109520478	109520532		-0.344	-0.38655
chr7	136881906	136881991		-0.34399	-0.352721
chrX	69851175	69851236		-0.343927	-0.364887
chr13	62631193	62631261		-0.343902	-0.335695
chr10	14284020	14284076		-0.343768	-0.30146
chr11	96348563	96348632		-0.343751	-0.338266
chr8	91028999	91029095		-0.343743	-0.329151
chr18	44045414	44045481		-0.343683	-0.323455
chr4	127134609	127134626		-0.343663	-0.362182
chrX	107951842	107951939		-0.343659	-0.480575
chr16	57094042	57094121		-0.343462	-0.406443
chrX	131061516	131061554		-0.343376	-0.345222
chr5	107255777	107255802		-0.343365	-0.349965
chr10	110270033	110270068		-0.34335	-0.355357
chr11	99380990	99381020		-0.343312	-0.352662
chr1	47557320	47557344		-0.343312	-0.430323
chr6	143540862	143540921		-0.343288	-0.359301
chr2	68564208	68564270		-0.343278	-0.351281
chr6	108755151	108755230		-0.343197	-0.310396
chr3	157122995	157123017		-0.343167	-0.422151
chr13	4643257	4643322		-0.343029	-0.374016
chr16	87544148	87544224		-0.342917	-0.344118
chr12	28434755	28434795		-0.342818	-0.368559
chr8	19060195	19060219		-0.342816	-0.309248
chr19	4136226	4136240		-0.342794	-0.339335
chr6	49878414	49878468		-0.342741	-0.363648
chr13	110254895	110254958		-0.342644	-0.351435
chr8	92727331	92727371		-0.342594	-0.326948
chr13	76141940	76141955		-0.342532	-0.345193
chr14	27963763	27963857		-0.342515	-0.328168
chr16	77743334	77743421		-0.342472	-0.302445

chr2	88068748	88068830		-0.342418	-0.32594
chr2	32321058	32321094		-0.342402	-0.305938
chr14	80086759	80086842		-0.342389	-0.388615
chr6	90897477	90897550		-0.342179	-0.314666
chr18	68424526	68424586		-0.342151	-0.339989
chr17	78442627	78442707		-0.341996	-0.308208
chr1	172784464	172784565		-0.341891	-0.331314
chr7	85385017	85385054		-0.341819	-0.360927
chr16	35599279	35599374		-0.341812	-0.318714
chr18	65740507	65740675		-0.341762	-0.305446
chr14	24360330	24360394		-0.341716	-0.41275
chr19	59434443	59434531		-0.341701	-0.335807
chr7	5533660	5533735		-0.341567	-0.327137
chr6	106271574	106271609		-0.341439	-0.372065
chr12	15538531	15538611		-0.341426	-0.443038
chr18	51500499	51500577		-0.341397	-0.395327
chr17	78905520	78905578		-0.341382	-0.33014
chr1	155485090	155485145		-0.341361	-0.318917
chr11	36751436	36751481		-0.341334	-0.408532
chr6	110134639	110134675		-0.341263	-0.317458
chr15	42282500	42282560		-0.341195	-0.411763
chr1	120186769	120186786		-0.341194	-0.502527
chr12	20567852	20567915		-0.341074	-0.310269
chrX	154929491	154929586		-0.341023	-0.364238
chr3	49303857	49303909		-0.340951	-0.437849
chr3	74563844	74563901		-0.340763	-0.322532
chr7	135794665	135794728		-0.340756	-0.36612
chr14	69863515	69863569		-0.340735	-0.321428
chr6	130933838	130933902		-0.340704	-0.367435
chr5	115342105	115342147		-0.340702	-0.437046
chr15	57640269	57640349		-0.340569	-0.583632
chr2	112556869	112556941		-0.340529	-0.361776
chr17	59482258	59482323		-0.34047	-0.408494
chr14	104372971	104373050		-0.340445	-0.415803
chr15	102230202	102230300		-0.340405	-0.375487
chr5	73960087	73960134		-0.340315	-0.368048
chr16	73637952	73638029		-0.340311	-0.343635
chr13	104208571	104208615		-0.340269	-0.352625
chr14	69854901	69854937		-0.340256	-0.305839
chr16	14326150	14326195		-0.340248	-0.449303
chr2	33539177	33539235		-0.340103	-0.319762
chr5	33937045	33937092		-0.339997	-0.362883
chr13	61596388	61596453		-0.339838	-0.33795

chr10	76097603	76097656	[redacted]	-0.339742	-0.30787
chr4	124188822	124188873	[redacted]	-0.339642	-0.502147
chr3	93830879	93830897	[redacted]	-0.339611	-0.35019
chr5	118158824	118158933	[redacted]	-0.339416	-0.337079
chr12	31437378	31437449	[redacted]	-0.339373	-0.363934
chr18	36205038	36205115	[redacted]	-0.339285	-0.453135
chr2	174674777	174674852	[redacted]	-0.339101	-0.413888
chr18	3644718	3644751	[redacted]	-0.339025	-0.434634
chrX	26470758	26470845	[redacted]	-0.338924	-0.418301
chr16	59475834	59475886	[redacted]	-0.338661	-0.340581
chr11	102987671	102987763	[redacted]	-0.338591	-0.316694
chr18	37694935	37694971	[redacted]	-0.338528	-0.326123
chr15	56147282	56147340	[redacted]	-0.338262	-0.311771
chr15	49524373	49524437	[redacted]	-0.33819	-0.356842
chr17	47269540	47269652	[redacted]	-0.338161	-0.305618
chr12	110160100	110160159	[redacted]	-0.338106	-0.336198
chr6	85660446	85660481	[redacted]	-0.338067	-0.386583
chr2	78976995	78977049	[redacted]	-0.338052	-0.428551
chr14	118313294	118313421	[redacted]	-0.338016	-0.327026
chr18	36929157	36929257	[redacted]	-0.337843	-0.447959
chr8	10609985	10610068	[redacted]	-0.337835	-0.347602
chr5	64624738	64624800	[redacted]	-0.337762	-0.520763
chr16	92863052	92863138	[redacted]	-0.337694	-0.331047
chr9	72236948	72237017	[redacted]	-0.337684	-0.357666
chr5	38430991	38431091	[redacted]	-0.337574	-0.33905
chr1	194913911	194913959	[redacted]	-0.337566	-0.370311
chr2	15110281	15110337	[redacted]	-0.337525	-0.313423
chr9	92003223	92003235	[redacted]	-0.337418	-0.305749
chr4	123752839	123752862	[redacted]	-0.337328	-0.36577
chr9	95547394	95547461	[redacted]	-0.337225	-0.305134
chr9	70037760	70037800	[redacted]	-0.337202	-0.35299
chr4	151874294	151874387	[redacted]	-0.337133	-0.305903
chr4	115947493	115947598	[redacted]	-0.337117	-0.318837
chr15	33399659	33399702	[redacted]	-0.337069	-0.392408
chr8	58509428	58509489	[redacted]	-0.337062	-0.35825
chr12	66691005	66691073	[redacted]	-0.33705	-0.311077
chr12	76648037	76648179	[redacted]	-0.336962	-0.324827
chr5	144312533	144312590	[redacted]	-0.336909	-0.383967
chr14	72340749	72340803	[redacted]	-0.336904	-0.365664
chr1	156768342	156768433	[redacted]	-0.336881	-0.350554
chr18	60432566	60432630	[redacted]	-0.33685	-0.322917
chr3	97685585	97685675	[redacted]	-0.336806	-0.350095
chr5	19828336	19828405	[redacted]	-0.336762	-0.329776

chr17	90561356	90561478		-0.33668	-0.460002
chr10	28986952	28987036		-0.33663	-0.37408
chr13	110627784	110627835		-0.33658	-0.363268
chr3	150904493	150904541		-0.336568	-0.345788
chr4	83325822	83325856		-0.336354	-0.350403
chr15	5632653	5632703		-0.336342	-0.42536
chr18	65854069	65854101		-0.336198	-0.322826
chr15	56146271	56146367		-0.336197	-0.310323
chr1	28910540	28910613		-0.336183	-0.318182
chr17	49412018	49412069		-0.336014	-0.387841
chr7	17062737	17062807		-0.336004	-0.331559
chr8	90300809	90300874		-0.336002	-0.362119
chr3	158206948	158207004		-0.335978	-0.318488
chr4	32520393	32520449		-0.335939	-0.333804
chr15	54998366	54998436		-0.335916	-0.346593
chr8	78337774	78337797		-0.335887	-0.357601
chr6	140349086	140349133		-0.335856	-0.460099
chr7	69988491	69988575		-0.335824	-0.313357
chr12	96433407	96433477		-0.335697	-0.357551
chr15	79012646	79012684		-0.335689	-0.334925
chr12	83039158	83039245		-0.335674	-0.317069
chr1	142696330	142696402		-0.335652	-0.421027
chr7	66576709	66576751		-0.335392	-0.436653
chr8	31322057	31322121		-0.335313	-0.406308
chr8	48507188	48507268		-0.335302	-0.390113
chr7	88518118	88518185		-0.335288	-0.379215
chr13	58490151	58490193		-0.335161	-0.361079
chr14	76473933	76473954		-0.335124	-0.423705
chr3	105619264	105619337		-0.335113	-0.351306
chr3	150396079	150396208		-0.335068	-0.344172
chr14	48473970	48474019		-0.335027	-0.388896
chr2	45220541	45220615		-0.335017	-0.325979
chr13	76421706	76421749		-0.334983	-0.343956
chr9	41874620	41874686		-0.334963	-0.488354
chr8	46384930	46385003		-0.334907	-0.311873
chr17	16786104	16786159		-0.334897	-0.327131
chr4	142657071	142657167		-0.334887	-0.353582
chr3	49750284	49750293		-0.334866	-0.540224
chr2	74958347	74958388		-0.334761	-0.312568
chr17	49142775	49142822		-0.334749	-0.456333
chr11	96350020	96350061		-0.334628	-0.419914
chr5	71232427	71232455		-0.334581	-0.325705
chr7	124851291	124851358		-0.334525	-0.438078

chr14	63393786	63393879		-0.334511	-0.33479
chr8	112127168	112127215		-0.334486	-0.312432
chr16	66928246	66928299		-0.334424	-0.35218
chr1	109067836	109067928		-0.334341	-0.371318
chrX	41478241	41478281		-0.33432	-0.306542
chr7	54798873	54798958		-0.334271	-0.305195
chr11	72277109	72277197		-0.334242	-0.53078
chr6	104773394	104773485		-0.334045	-0.405334
chr12	23754421	23754461		-0.333976	-0.354151
chr19	20492870	20492917		-0.33392	-0.374381
chr6	17463077	17463133		-0.333915	-0.455329
chr7	18621820	18621868		-0.333911	-0.328337
chr11	104604321	104604344		-0.333909	-0.359098
chr8	31627100	31627156		-0.333829	-0.325341
chr18	37492260	37492325		-0.33374	-0.543165
chr9	90871786	90871837		-0.333718	-0.300621
chr12	118560289	118560418		-0.333709	-0.481216
chrX	105522379	105522467		-0.333591	-0.463579
chr12	21013894	21013989		-0.333476	-0.300148
chr3	92921527	92921593		-0.333476	-0.356897
chr17	4866714	4866781		-0.333422	-0.407633
chr8	114203280	114203367		-0.333372	-0.376128
chr11	58682914	58682958		-0.333351	-0.330205
chr10	126976616	126976708		-0.333327	-0.312317
chr16	65726604	65726699		-0.333167	-0.358492
chr6	115769697	115769789		-0.333163	-0.347829
chr1	44350612	44350677		-0.333099	-0.345548
chr10	40796464	40796528		-0.333071	-0.320045
chr11	18720306	18720390		-0.332988	-0.351618
chr8	61933968	61934036		-0.332918	-0.380123
chr14	34351304	34351324		-0.332878	-0.39929
chr6	124888416	124888451		-0.332821	-0.385549
chr2	143709694	143709756		-0.332816	-0.380525
chr18	68062436	68062481		-0.332701	-0.355658
chr19	31678664	31678783		-0.332552	-0.40559
chr13	4759863	4759981		-0.332492	-0.326573
chr14	46166226	46166264		-0.332476	-0.352559
chr6	100883262	100883329		-0.332337	-0.462762
chr13	61211843	61211928		-0.332303	-0.307009
chr12	6243836	6243908		-0.332272	-0.387258
chr13	21927881	21927986		-0.332241	-0.609591
chr4	63935937	63935982		-0.332115	-0.324359
chr4	58011871	58011925		-0.332066	-0.316376

chr10	22469263	22469315		-0.332032	-0.440659
chr15	41079463	41079566		-0.3318	-0.453375
chr10	82269179	82269234		-0.331798	-0.422652
chr13	85388311	85388389		-0.331794	-0.300042
chr2	155527172	155527248		-0.331559	-0.320154
chr10	123515965	123516041		-0.331502	-0.366654
chr8	37495645	37495703		-0.331487	-0.377386
chr18	22952273	22952305		-0.331434	-0.45182
chr19	34382259	34382288		-0.331417	-0.345923
chr4	59226899	59226906		-0.331362	-0.359904
chr16	27164663	27164749		-0.331252	-0.386667
chr19	18649906	18649971		-0.331102	-0.340116
chrX	78235365	78235380		-0.33108	-0.327742
chr12	10857600	10857705		-0.330939	-0.336047
chr14	11930212	11930254		-0.33092	-0.303634
chr4	98519394	98519496		-0.330905	-0.312423
chr13	81306086	81306170		-0.330823	-0.30851
chr15	97224739	97224826		-0.330789	-0.359994
chr3	159720316	159720401		-0.33078	-0.397161
chr5	129067467	129067540		-0.330775	-0.302341
chr18	64204348	64204400		-0.330687	-0.368418
chr6	90283052	90283096		-0.330635	-0.535865
chr13	95639443	95639494		-0.330619	-0.367288
chr14	26533406	26533451		-0.330575	-0.39214
chr14	99605028	99605069		-0.330548	-0.320763
chr11	105436238	105436285		-0.33054	-0.341279
chr14	35163753	35163822		-0.330501	-0.416959
chr1	34107932	34107996		-0.330489	-0.54371
chr8	34080599	34080680		-0.330475	-0.318827
chr4	85380302	85380373		-0.330427	-0.328987
chr7	29168009	29168061		-0.330413	-0.394058
chr17	83629525	83629578		-0.330129	-0.318535
chr16	15140351	15140400		-0.330124	-0.304719
chr5	97396410	97396485		-0.329989	-0.383475
chr2	152554641	152554700		-0.329954	-0.316479
chr18	85504002	85504083		-0.329948	-0.497786
chr18	67904833	67904924		-0.329912	-0.329834
chr5	108955480	108955540		-0.329902	-0.37557
chr2	81288337	81288414		-0.32986	-0.305469
chr4	133816665	133816762		-0.329858	-0.384488
chr5	65856887	65856963		-0.329682	-0.326049
chr7	44276503	44276540		-0.329681	-0.356575
chr7	12359173	12359204		-0.329653	-0.543144

chr17	33269073	33269111		-0.329639	-0.376005
chr11	23094725	23094760		-0.329573	-0.394117
chr14	56345432	56345495		-0.32954	-0.377328
chr8	38743736	38743803		-0.329517	-0.344807
chr18	53752957	53753294		-0.329483	-0.382249
chr7	105155932	105155986		-0.32945	-0.325352
chr2	28975784	28975863		-0.329137	-0.32441
chr2	145634469	145634596		-0.329034	-0.442005
chr4	36119910	36119975		-0.328916	-0.324699
chr2	118356812	118356855		-0.328646	-0.322403
chr6	85417380	85417448		-0.328575	-0.320345
chr11	10508781	10508849		-0.328568	-0.340412
chr14	88942397	88942519		-0.328506	-0.317765
chr10	115188150	115188234		-0.328483	-0.373293
chr2	10367442	10367504		-0.328422	-0.441472
chr19	34187570	34187607		-0.328421	-0.353811
chr9	43119132	43119166		-0.328374	-0.351503
chr3	108390273	108390322		-0.32831	-0.314878
chr8	27494864	27494939		-0.328303	-0.419232
chr1	11590539	11590608		-0.328286	-0.354089
chr9	63793941	63794005		-0.328166	-0.404103
chr14	51971430	51971510		-0.327941	-0.303416
chr9	42971629	42971674		-0.327922	-0.326989
chr5	96947365	96947412		-0.327876	-0.304961
chr4	132091902	132091953		-0.327839	-0.372546
chr8	23271036	23271138		-0.327819	-0.469822
chr12	108313775	108313854		-0.327791	-0.48583
chr5	81848378	81848465		-0.327659	-0.306956
chr10	102670485	102670564		-0.327636	-0.426209
chr2	159963877	159963962		-0.327628	-0.363213
chr5	104297066	104297158		-0.327617	-0.300052
chr5	46823349	46823389		-0.32755	-0.312391
chr11	58849367	58849397		-0.327523	-0.357347
chr11	46539859	46539867		-0.327514	-0.373657
chr10	107320726	107320830		-0.327459	-0.37287
chr18	52848355	52848385		-0.32743	-0.327733
chr8	112282026	112282084		-0.327429	-0.351637
chr6	107138745	107138806		-0.327373	-0.407127
chr7	89068695	89068774		-0.327329	-0.301585
chr11	51057928	51057976		-0.327263	-0.303281
chr12	98613247	98613324		-0.327225	-0.303987
chr5	29106353	29106454		-0.327181	-0.374792
chr11	92800979	92801017		-0.327181	-0.45682

chrX	135211313	135211361	[redacted]	-0.327043	-0.300566
chr1	61087659	61087742	[redacted]	-0.327026	-0.386823
chr18	89512154	89512221	[redacted]	-0.326951	-0.336222
chr14	24943965	24944003	[redacted]	-0.326831	-0.35863
chr5	68225676	68225725	[redacted]	-0.326779	-0.376231
chr11	87852575	87852643	[redacted]	-0.326774	-0.312858
chr6	60972350	60972398	[redacted]	-0.326724	-0.359731
chr2	43037943	43038041	[redacted]	-0.326608	-0.316474
chr2	165180309	165180375	[redacted]	-0.326503	-0.34537
chr18	33529874	33529946	[redacted]	-0.326502	-0.312274
chr9	113422068	113422159	[redacted]	-0.326292	-0.402762
chr1	116339203	116339225	[redacted]	-0.326261	-0.429249
chr6	141403408	141403451	[redacted]	-0.326236	-0.354238
chr9	68904562	68904656	[redacted]	-0.326105	-0.314933
chr3	153756134	153756147	[redacted]	-0.325984	-0.326244
chr3	13208600	13208635	[redacted]	-0.325942	-0.359484
chr2	102249152	102249236	[redacted]	-0.325932	-0.321815
chr16	67060918	67060972	[redacted]	-0.325869	-0.317297
chr16	14683856	14683904	[redacted]	-0.325798	-0.351772
chr8	51163276	51163338	[redacted]	-0.325786	-0.320755
chr8	75216204	75216272	[redacted]	-0.325761	-0.313615
chr11	92384554	92384619	[redacted]	-0.325624	-0.318514
chr2	74987276	74987354	[redacted]	-0.325587	-0.339132
chr10	110477479	110477552	[redacted]	-0.325562	-0.309759
chr11	94719825	94719888	[redacted]	-0.325514	-0.365414
chr8	102841345	102841416	[redacted]	-0.3255	-0.41878
chr1	180903420	180903551	[redacted]	-0.325444	-0.308884
chr5	104769889	104769961	[redacted]	-0.325382	-0.339682
chr11	10141332	10141404	[redacted]	-0.325281	-0.434395
chr7	89040176	89040232	[redacted]	-0.325179	-0.437218
chr10	92684913	92684973	[redacted]	-0.325145	-0.449838
chr12	64495132	64495224	[redacted]	-0.325083	-0.31252
chr18	70943287	70943307	[redacted]	-0.325001	-0.330939
chr2	28290297	28290313	[redacted]	-0.324901	-0.454946
chr12	33823686	33823734	[redacted]	-0.324874	-0.353011
chrX	75661135	75661228	[redacted]	-0.324836	-0.427173
chr19	45516947	45516981	[redacted]	-0.324826	-0.363513
chr13	112881825	112881880	[redacted]	-0.324811	-0.415476
chr9	54228081	54228147	[redacted]	-0.324759	-0.307239
chr7	123063580	123063614	[redacted]	-0.324735	-0.536486
chr6	70190987	70191040	[redacted]	-0.324704	-0.498956
chr5	46636202	46636286	[redacted]	-0.324684	-0.396364
chr10	71300405	71300487	[redacted]	-0.324651	-0.557774

chr7	115634513	115634597	[redacted]	-0.324574	-0.338417
chr4	131558375	131558463	[redacted]	-0.324568	-0.420303
chr2	110837684	110837841	[redacted]	-0.324566	-0.387556
chr15	49418544	49418628	[redacted]	-0.324552	-0.355959
chr7	113380090	113380129	[redacted]	-0.324504	-0.374975
chr18	64883288	64883468	[redacted]	-0.324498	-0.391054
chr11	95017186	95017236	[redacted]	-0.324469	-0.394078
chr10	66163908	66163943	[redacted]	-0.324464	-0.3917
chr10	87956416	87956475	[redacted]	-0.324336	-0.3337
chr1	183862176	183862296	[redacted]	-0.324299	-0.528299
chr11	48079647	48079699	[redacted]	-0.324174	-0.348237
chr4	89717654	89717790	[redacted]	-0.324132	-0.307463
chr14	51943452	51943507	[redacted]	-0.324006	-0.613735
chr6	87947423	87947546	[redacted]	-0.323898	-0.489352
chr2	70106222	70106280	[redacted]	-0.323811	-0.339469
chr4	147808800	147808900	[redacted]	-0.32369	-0.399102
chrX	66657858	66657932	[redacted]	-0.323515	-0.365437
chr12	11206474	11206539	[redacted]	-0.323441	-0.435435
chr15	86607199	86607231	[redacted]	-0.323421	-0.31956
chr3	149333021	149333087	[redacted]	-0.323354	-0.311902
chr11	79503911	79503964	[redacted]	-0.323351	-0.314855
chr2	14189623	14189685	[redacted]	-0.323349	-0.480972
chr19	17739468	17739529	[redacted]	-0.323303	-0.420189
chr5	20099316	20099379	[redacted]	-0.323285	-0.338871
chr12	52710884	52710951	[redacted]	-0.323198	-0.326863
chr8	109424177	109424281	[redacted]	-0.323142	-0.332856
chr8	112404540	112404662	[redacted]	-0.323064	-0.3927
chr3	66217619	66217707	[redacted]	-0.322988	-0.355053
chr1	69093481	69093535	[redacted]	-0.32288	-0.417593
chr17	25179674	25179741	[redacted]	-0.322859	-0.307772
chrX	48909482	48909560	[redacted]	-0.322794	-0.306199
chr4	9527660	9527735	[redacted]	-0.32279	-0.309852
chr2	12832797	12832838	[redacted]	-0.322739	-0.305262
chr18	68076082	68076117	[redacted]	-0.322702	-0.339649
chr7	57418827	57418877	[redacted]	-0.322558	-0.468155
chr2	89345084	89345138	[redacted]	-0.322516	-0.342981
chr8	43435625	43435680	[redacted]	-0.322454	-0.462809
chr5	109234763	109234835	[redacted]	-0.322421	-0.429528
chr11	110358416	110358448	[redacted]	-0.322416	-0.381455
chrX	113317313	113317391	[redacted]	-0.322316	-0.406686
chr11	85966094	85966119	[redacted]	-0.322281	-0.300143
chr3	8021802	8021929	[redacted]	-0.322213	-0.309977
chr9	6649500	6649554	[redacted]	-0.322198	-0.342719

chr17	36351795	36351858		-0.322174	-0.301518
chr14	119296014	119296086		-0.322124	-0.388857
chr4	115965997	115966035		-0.322037	-0.415935
chr5	12324416	12324482		-0.321982	-0.358088
chr3	51042161	51042256		-0.321955	-0.315425
chr5	144286389	144286531		-0.321905	-0.33403
chr8	52826721	52826815		-0.321896	-0.60053
chr2	75424883	75424933		-0.321895	-0.412093
chr1	37515363	37515423		-0.321794	-0.328267
chr3	121028166	121028221		-0.321599	-0.334634
chr17	69514580	69514694		-0.321593	-0.332097
chr8	26542043	26542098		-0.321562	-0.309525
chr19	44686880	44686959		-0.321554	-0.415555
chr3	79641312	79641398		-0.321544	-0.314873
chr6	115296743	115296805		-0.321543	-0.33695
chr10	23231538	23231624		-0.32153	-0.43249
chr18	10882469	10882536		-0.321274	-0.537621
chr6	6440534	6440606		-0.321258	-0.336714
chr11	51085693	51085752		-0.321234	-0.345585
chr5	72410786	72410819		-0.321205	-0.353908
chr3	119678509	119678582		-0.321167	-0.310579
chr10	44715568	44715619		-0.321085	-0.358368
chr16	19223008	19223049		-0.321047	-0.349994
chrX	155379097	155379148		-0.320883	-0.402332
chr5	3279947	3279987		-0.320844	-0.426141
chr15	14177262	14177362		-0.320757	-0.380099
chr14	32200097	32200146		-0.320737	-0.312336
chr13	12895018	12895077		-0.320642	-0.465303
chr14	34809049	34809080		-0.320639	-0.307235
chr19	17962632	17962729		-0.320606	-0.412412
chr7	43066392	43066465		-0.320535	-0.333194
chr1	61183559	61183637		-0.320413	-0.37697
chr2	164538712	164538767		-0.320219	-0.326781
chr2	28410458	28410521		-0.320097	-0.305028
chrUn_	98015	98074		-0.320064	-0.361777
chr11	8811180	8811263		-0.320019	-0.320796
chr11	6765099	6765143		-0.319948	-0.307561
chr10	108435349	108435418		-0.319945	-0.332744
chr4	45967004	45967040		-0.319815	-0.427944
chr17	93250633	93250709		-0.319762	-0.358915
chr17	45264945	45264987		-0.319744	-0.357725
chr7	31467504	31467576		-0.319741	-0.505033
chr5	84319145	84319196		-0.319741	-0.378625

chr3	139247830	139247898		-0.319671	-0.315469
chrX	39889688	39889767		-0.319554	-0.346776
chr14	26844606	26844678		-0.319503	-0.315043
chr17	11522360	11522445		-0.319419	-0.384305
chr1	126727408	126727471		-0.319389	-0.31744
chr19	39826320	39826364		-0.319379	-0.397616
chr17	56497540	56497595		-0.319345	-0.347423
chr13	48724054	48724091		-0.3193	-0.354333
chr19	35368607	35368635		-0.319218	-0.434189
chr3	97056248	97056286		-0.319178	-0.349008
chr6	114581023	114581063		-0.319155	-0.368598
chr9	99362188	99362217		-0.319126	-0.345072
chr18	44158395	44158455		-0.3191	-0.339567
chr9	124156371	124156406		-0.319095	-0.305739
chr1	172408307	172408403		-0.319091	-0.409695
chr14	64513609	64513683		-0.319031	-0.311387
chr13	78238324	78238390		-0.319018	-0.341107
chr15	56150442	56150501		-0.318918	-0.334296
chr3	34212670	34212703		-0.318813	-0.308211
chr1	56081635	56081712		-0.318812	-0.316602
chr8	121813024	121813140		-0.318768	-0.367123
chr13	16538031	16538079		-0.318735	-0.479546
chr11	100994919	100994984		-0.318699	-0.372348
chr10	9969720	9969777		-0.318637	-0.38392
chr8	56552346	56552363		-0.318448	-0.319698
chr6	87962372	87962416		-0.318435	-0.334462
chr3	79743477	79743492		-0.318409	-0.321639
chr15	52574539	52574687		-0.318296	-0.395815
chr4	33979922	33979947		-0.318209	-0.304316
chr6	32239883	32239928		-0.318205	-0.324238
chr3	121235299	121235369		-0.318129	-0.387898
chr3	133211612	133211685		-0.318118	-0.358158
chr11	18556921	18556984		-0.31805	-0.350499
chr5	64915179	64915194		-0.318049	-0.336453
chr3	97796722	97796803		-0.317902	-0.317589
chr3	148093073	148093120		-0.317889	-0.337125
chr19	39119674	39119706		-0.317857	-0.310399
chr1	169663421	169663486		-0.317816	-0.325656
chr16	28625762	28625799		-0.317743	-0.407616
chr4	117230351	117230376		-0.317684	-0.366956
chr18	58038778	58038826		-0.317674	-0.465106
chr15	6817013	6817075		-0.317484	-0.350396
chr17	18559343	18559490		-0.317466	-0.412572

chr8	127823021	127823158	[redacted]	-0.31745	-0.328281
chr10	98001058	98001110	[redacted]	-0.317437	-0.332172
chr12	10479396	10479470	[redacted]	-0.317433	-0.40478
chr16	60322176	60322237	[redacted]	-0.317385	-0.35985
chr11	114934874	114934951	[redacted]	-0.317354	-0.398302
chr5	137186890	137187006	[redacted]	-0.317229	-0.350876
chr3	157837588	157837670	[redacted]	-0.316889	-0.330654
chr8	36454988	36455076	[redacted]	-0.316861	-0.464163
chr14	66634142	66634223	[redacted]	-0.31684	-0.322162
chr10	90088283	90088324	[redacted]	-0.316829	-0.392396
chrX	142309245	142309321	[redacted]	-0.316807	-0.506813
chr2	121719213	121719266	[redacted]	-0.316739	-0.32946
chr5	22742668	22742736	[redacted]	-0.316557	-0.353699
chr2	87150200	87150296	[redacted]	-0.316522	-0.322566
chr14	44964198	44964257	[redacted]	-0.31644	-0.439409
chr7	44287319	44287355	[redacted]	-0.31642	-0.316496
chr13	39392589	39392634	[redacted]	-0.316214	-0.314703
chr5	31494128	31494191	[redacted]	-0.316179	-0.406308
chr15	64626533	64626615	[redacted]	-0.316121	-0.355363
chr11	44223042	44223075	[redacted]	-0.316032	-0.364198
chr8	46983186	46983230	[redacted]	-0.316026	-0.343682
chr15	11970749	11970768	[redacted]	-0.315992	-0.339768
chr1	37658532	37658557	[redacted]	-0.31599	-0.317436
chr16	61848381	61848439	[redacted]	-0.315889	-0.314697
chr18	66646031	66646131	[redacted]	-0.315821	-0.327469
chr1	33402373	33402443	[redacted]	-0.315798	-0.304009
chr15	6960324	6960414	[redacted]	-0.315751	-0.337572
chr15	51631906	51631982	[redacted]	-0.315712	-0.326225
chr2	140383095	140383185	[redacted]	-0.315632	-0.361456
chr1	133351871	133351971	[redacted]	-0.315565	-0.389146
chr14	99734052	99734137	[redacted]	-0.315543	-0.44056
chr15	44515373	44515439	[redacted]	-0.31549	-0.411663
chr15	24367929	24368029	[redacted]	-0.315489	-0.316396
chr17	23890637	23890758	[redacted]	-0.315412	-0.31843
chr8	40590324	40590396	[redacted]	-0.315352	-0.398995
chr1	151931808	151931856	[redacted]	-0.315299	-0.492977
chr8	15039475	15039552	[redacted]	-0.315192	-0.320464
chr17	65606247	65606286	[redacted]	-0.315148	-0.331938
chr1	136794172	136794204	[redacted]	-0.315059	-0.341834
chr18	39252684	39252748	[redacted]	-0.315024	-0.355348
chr3	73617960	73618015	[redacted]	-0.315005	-0.610513
chr15	26103625	26103695	[redacted]	-0.31493	-0.320778
chr15	91624098	91624163	[redacted]	-0.314871	-0.383592

chr1	65378100	65378194	[redacted]	-0.31481	-0.337383
chrX	41195774	41195823	[redacted]	-0.314779	-0.419421
chr2	165343319	165343350	[redacted]	-0.314712	-0.381617
chr14	58110555	58110606	[redacted]	-0.314683	-0.308127
chr2	10871314	10871388	[redacted]	-0.314663	-0.309617
chr16	32475194	32475218	[redacted]	-0.314627	-0.302221
chr9	99147519	99147636	[redacted]	-0.314573	-0.416012
chr3	82429317	82429380	[redacted]	-0.314539	-0.329891
chr1	41713257	41713322	[redacted]	-0.314511	-0.336774
chr11	68906333	68906383	[redacted]	-0.314371	-0.358153
chr19	20317841	20317961	[redacted]	-0.314356	-0.364515
chr2	162669405	162669435	[redacted]	-0.314336	-0.344341
chr8	62141286	62141345	[redacted]	-0.314267	-0.336828
chr10	11097539	11097632	[redacted]	-0.314245	-0.355814
chr10	95074754	95074827	[redacted]	-0.314203	-0.397528
chr11	75246584	75246682	[redacted]	-0.314142	-0.310516
chr11	5590590	5590625	[redacted]	-0.314132	-0.300988
chr3	96324015	96324082	[redacted]	-0.314125	-0.335181
chr11	73557429	73557545	[redacted]	-0.3141	-0.33044
chr15	4704724	4704798	[redacted]	-0.314024	-0.382747
chr6	19029646	19029736	[redacted]	-0.314004	-0.354304
chr1	156874192	156874273	[redacted]	-0.313947	-0.31393
chrX	143472531	143472559	[redacted]	-0.313942	-0.319208
chr4	42925023	42925057	[redacted]	-0.31386	-0.361355
chr13	54577847	54577893	[redacted]	-0.313837	-0.443549
chr14	63568077	63568146	[redacted]	-0.313829	-0.333859
chr7	13003288	13003368	[redacted]	-0.31368	-0.321358
chr1	94923062	94923141	[redacted]	-0.313593	-0.323829
chr13	48607831	48607862	[redacted]	-0.313591	-0.305165
chr14	101518225	101518323	[redacted]	-0.313587	-0.318077
chr15	26564966	26565030	[redacted]	-0.313584	-0.429166
chr6	37460992	37461022	[redacted]	-0.313539	-0.405708
chr10	79330699	79330861	[redacted]	-0.313486	-0.322927
chr2	114123110	114123163	[redacted]	-0.313245	-0.431385
chr10	126215669	126215726	[redacted]	-0.313079	-0.377949
chr13	42293144	42293162	[redacted]	-0.313006	-0.3111
chr9	10445213	10445288	[redacted]	-0.312943	-0.437686
chr5	36755494	36755562	[redacted]	-0.312899	-0.404442
chr4	127108007	127108096	[redacted]	-0.312887	-0.414282
chr13	31563199	31563268	[redacted]	-0.312819	-0.301121
chr13	56374452	56374509	[redacted]	-0.312686	-0.312705
chr16	96963612	96963692	[redacted]	-0.312669	-0.459069
chr12	15932175	15932197	[redacted]	-0.312667	-0.401473

chr19	36329350	36329401	[redacted]	-0.312563	-0.310515
chr13	98224248	98224363	[redacted]	-0.312203	-0.408846
chr12	77994331	77994403	[redacted]	-0.3122	-0.446635
chr2	17473636	17473712	[redacted]	-0.312156	-0.429739
chr16	22701958	22702026	[redacted]	-0.312131	-0.412771
chr9	89037259	89037306	[redacted]	-0.311839	-0.47182
chr16	78833585	78833641	[redacted]	-0.311742	-0.38636
chr1	15670953	15671053	[redacted]	-0.311689	-0.300098
chr19	43120437	43120458	[redacted]	-0.311679	-0.403362
chr18	73864987	73865019	[redacted]	-0.311675	-0.506653
chr17	81707951	81708014	[redacted]	-0.311654	-0.31733
chr1	170606757	170606800	[redacted]	-0.311624	-0.33252
chr17	79516393	79516422	[redacted]	-0.311429	-0.304562
chr1	158527404	158527505	[redacted]	-0.311397	-0.372059
chr11	110278771	110278820	[redacted]	-0.311386	-0.32225
chr5	141403073	141403139	[redacted]	-0.311376	-0.338498
chr3	16269315	16269361	[redacted]	-0.311152	-0.308839
chrX	94672002	94672090	[redacted]	-0.31101	-0.303885
chr6	98851702	98851745	[redacted]	-0.310894	-0.332441
chr9	36672545	36672636	[redacted]	-0.310821	-0.314847
chr3	127378618	127378668	[redacted]	-0.310778	-0.307594
chr19	13708904	13708977	[redacted]	-0.310713	-0.432985
chr2	75424763	75424830	[redacted]	-0.310696	-0.316423
chr3	20760991	20761026	[redacted]	-0.310622	-0.357665
chr10	116558765	116558782	[redacted]	-0.310567	-0.345388
chr5	89916208	89916257	[redacted]	-0.31056	-0.369485
chr3	101387518	101387538	[redacted]	-0.310535	-0.377197
chr8	91224357	91224444	[redacted]	-0.310517	-0.307538
chr3	86141322	86141400	[redacted]	-0.310434	-0.321545
chr4	106678196	106678268	[redacted]	-0.310375	-0.454417
chr10	69087057	69087151	[redacted]	-0.310345	-0.493508
chr4	47495250	47495304	[redacted]	-0.310327	-0.430362
chr2	150502633	150502673	[redacted]	-0.310095	-0.426186
chr7	46928034	46928076	[redacted]	-0.310081	-0.348
chr9	115624766	115624845	[redacted]	-0.310026	-0.323082
chr6	60417774	60417822	[redacted]	-0.310025	-0.366644
chr3	49128618	49128659	[redacted]	-0.309983	-0.33872
chr7	135711078	135711126	[redacted]	-0.309891	-0.314152
chr4	33193227	33193321	[redacted]	-0.309889	-0.379627
chr5	91250016	91250064	[redacted]	-0.309876	-0.340236
chr3	122834580	122834598	[redacted]	-0.309852	-0.301877
chr2	40012224	40012295	[redacted]	-0.309803	-0.307408
chr11	83835889	83835921	[redacted]	-0.309795	-0.417421

chr1	192746616	192746671	[redacted]	-0.309791	-0.449234
chr2	83803148	83803196	[redacted]	-0.309769	-0.300381
chr5	103500987	103501084	[redacted]	-0.309666	-0.366508
chr5	26144069	26144165	[redacted]	-0.309636	-0.301547
chr3	145989857	145989902	[redacted]	-0.309619	-0.307421
chr8	49165182	49165233	[redacted]	-0.309544	-0.347978
chr16	20440428	20440458	[redacted]	-0.30947	-0.341421
chr1	104161453	104161471	[redacted]	-0.309464	-0.301253
chr18	75701398	75701425	[redacted]	-0.309425	-0.331293
chr10	109820720	109820795	[redacted]	-0.309411	-0.329625
chr9	11726748	11726841	[redacted]	-0.309334	-0.335846
chrX	55982478	55982557	[redacted]	-0.309223	-0.381215
chr9	24978312	24978369	[redacted]	-0.309177	-0.365006
chr4	51973482	51973517	[redacted]	-0.309149	-0.387916
chr16	53972740	53972824	[redacted]	-0.309143	-0.35919
chr11	65950197	65950285	[redacted]	-0.309124	-0.511997
chr17	40051817	40051880	[redacted]	-0.309101	-0.387674
chr18	71231362	71231421	[redacted]	-0.309002	-0.331125
chr13	81569799	81569826	[redacted]	-0.308969	-0.316298
chr12	9536190	9536235	[redacted]	-0.308967	-0.313646
chr6	19995832	19995859	[redacted]	-0.308961	-0.315779
chr5	149562385	149562542	[redacted]	-0.308961	-0.332659
chr15	80268548	80268620	[redacted]	-0.308936	-0.319431
chr13	52409765	52409812	[redacted]	-0.308918	-0.301974
chr16	95898090	95898171	[redacted]	-0.308873	-0.410241
chr2	97543388	97543469	[redacted]	-0.308817	-0.388999
chr12	44050506	44050562	[redacted]	-0.308703	-0.452378
chrX	10435730	10435823	[redacted]	-0.308686	-0.380362
chr9	95777466	95777535	[redacted]	-0.308666	-0.308451
chr12	19163907	19163985	[redacted]	-0.308605	-0.408485
chr5	61358204	61358227	[redacted]	-0.308563	-0.439485
chr15	92499407	92499505	[redacted]	-0.308559	-0.320453
chr10	93686365	93686399	[redacted]	-0.308527	-0.327494
chr13	86449697	86449733	[redacted]	-0.308411	-0.443348
chr6	111017751	111017833	[redacted]	-0.308399	-0.334199
chr11	111249111	111249189	[redacted]	-0.308298	-0.320507
chr17	61095845	61095863	[redacted]	-0.308031	-0.319014
chr15	72740127	72740203	[redacted]	-0.308	-0.35404
chr2	67880299	67880351	[redacted]	-0.307791	-0.313399
chr4	90872501	90872560	[redacted]	-0.307743	-0.375263
chr5	62355551	62355621	[redacted]	-0.307637	-0.386917
chr15	97974058	97974106	[redacted]	-0.307608	-0.706403
chr6	130842410	130842485	[redacted]	-0.307606	-0.418796

chr1	159188688	159188731		-0.307558	-0.354657
chr5	102688005	102688092		-0.307536	-0.345286
chr4	37322620	37322691		-0.307529	-0.39928
chr2	164223473	164223562		-0.307526	-0.336186
chr5	36464217	36464271		-0.307414	-0.346388
chr10	27285754	27285777		-0.307313	-0.399541
chr7	32584403	32584433		-0.307301	-0.311582
chr3	153167120	153167162		-0.307237	-0.304002
chr10	10624902	10624950		-0.30719	-0.366817
chr9	39196066	39196123		-0.30707	-0.323126
chr14	76359862	76359894		-0.306995	-0.308996
chr3	143309477	143309540		-0.306903	-0.421237
chrX	18315164	18315181		-0.30672	-0.301442
chr18	46740581	46740598		-0.306708	-0.435561
chr18	82163392	82163454		-0.306639	-0.314222
chr15	72215586	72215648		-0.306631	-0.32291
chr3	84313627	84313644		-0.306527	-0.308182
chr10	87394138	87394268		-0.306526	-0.441231
chr13	62323209	62323326		-0.306288	-0.311377
chr14	48039221	48039343		-0.306156	-0.362561
chr13	41239806	41239849		-0.306116	-0.49048
chr1	80993200	80993266		-0.306105	-0.40201
chr11	34125396	34125440		-0.305889	-0.364226
chr12	115781185	115781208		-0.305859	-0.393559
chr9	89197983	89198066		-0.305838	-0.444442
chr8	31365061	31365122		-0.305808	-0.346315
chr3	106201672	106201732		-0.305797	-0.435616
chr17	69160024	69160075		-0.305667	-0.351052
chr5	151687243	151687322		-0.305657	-0.475448
chr13	4378252	4378311		-0.305624	-0.330704
chr1	56212082	56212175		-0.305597	-0.306524
chr13	5082852	5082921		-0.30548	-0.322284
chr9	61778645	61778726		-0.305427	-0.305687
chr9	23203806	23203880		-0.305286	-0.427922
chr15	51239342	51239390		-0.305257	-0.350091
chr5	145716845	145716967		-0.305076	-0.31272
chr1	57377092	57377135		-0.305036	-0.35003
chr18	6920064	6920108		-0.304554	-0.566138
chr4	41465698	41465732		-0.304541	-0.318089
chr2	105594664	105594726		-0.304493	-0.344378
chr13	4124052	4124118		-0.304478	-0.304487
chr2	80704983	80705086		-0.304463	-0.334157
chr11	46698346	46698418		-0.304405	-0.304172

chr8	96702618	96702668		-0.304329	-0.583893
chr15	54789010	54789084		-0.304283	-0.324337
chr19	48005204	48005226		-0.304267	-0.358867
chr18	19367969	19368017		-0.304266	-0.321355
chr5	100916959	100917025		-0.30422	-0.304552
chr4	86943408	86943482		-0.304172	-0.342324
chr11	64420434	64420503		-0.304129	-0.31774
chr4	4556283	4556372		-0.30411	-0.425135
chr17	49241326	49241349		-0.30408	-0.394057
chr6	113846581	113846637		-0.303993	-0.313871
chr17	9338549	9338591		-0.303798	-0.357766
chr10	28649154	28649174		-0.303662	-0.389779
chr9	103166072	103166104		-0.303526	-0.350386
chr3	58750445	58750484		-0.303385	-0.596903
chr2	164203080	164203122		-0.303337	-0.353926
chr14	8094763	8094818		-0.303324	-0.338062
chr6	95102463	95102516		-0.303214	-0.372893
chr4	46665196	46665227		-0.303173	-0.362432
chr2	164704413	164704445		-0.303157	-0.499168
chr17	39456203	39456225		-0.303138	-0.473844
chr1	50189775	50189840		-0.303069	-0.303248
chr10	5961931	5961987		-0.302987	-0.341782
chr6	127267918	127267931		-0.302959	-0.329933
chr8	19535980	19536115		-0.302848	-0.399889
chr7	111411094	111411170		-0.30279	-0.329062
chr4	52365317	52365406		-0.30275	-0.487067
chr3	93865012	93865023		-0.302745	-0.315284
chr17	34776134	34776188		-0.302531	-0.383984
chr9	100574225	100574266		-0.302408	-0.426855
chr12	115237415	115237494		-0.30239	-0.40989
chr13	70505319	70505379		-0.30237	-0.370805
chr8	90373781	90373872		-0.302288	-0.302017
chr2	142577435	142577581		-0.302124	-0.314704
chr19	34669179	34669240		-0.302088	-0.590414
chr16	78784692	78784762		-0.302016	-0.360814
chr4	131362104	131362150		-0.301975	-0.395588
chr4	125939477	125939500		-0.30189	-0.328352
chr2	117637187	117637244		-0.301879	-0.342178
chr6	126544844	126544906		-0.301826	-0.399344
chr4	105033473	105033534		-0.30182	-0.347694
chr14	65297786	65297861		-0.301708	-0.308673
chrX	76989736	76989784		-0.30167	-0.379032
chr2	168276138	168276195		-0.301645	-0.56843

chr19	9070218	9070302		-0.301451	-0.33665
chr12	47729730	47729785		-0.301256	-0.410608
chr14	43041761	43041795		-0.301242	-0.348863
chr2	80887092	80887168		-0.301226	-0.328068
chr3	16338167	16338226		-0.301149	-0.329531
chr3	159592802	159592893		-0.301054	-0.303405
chr3	93837350	93837448		-0.301006	-0.331882
chr18	46346232	46346242		-0.300997	-0.356786
chr10	28105597	28105636		-0.300903	-0.396164
chr10	40016960	40017088		-0.300858	-0.475576
chr10	107603196	107603233		-0.300857	-0.403676
chr1	156727924	156727978		-0.300829	-0.358285
chr19	42344537	42344656		-0.300773	-0.3137
chrX	108555070	108555085		-0.300768	-0.38712
chr4	96990141	96990209		-0.300764	-0.314004
chr10	120723406	120723465		-0.300668	-0.338556
chr8	107586756	107586891		-0.300661	-0.303511
chr11	78439477	78439561		-0.300653	-0.347009
chr13	75453518	75453567		-0.300568	-0.376084
chr18	73119534	73119586		-0.300494	-0.32277
chr11	72277262	72277422		-0.300422	-0.470308
chr6	66306278	66306353		-0.300418	-0.302959
chr12	35199583	35199635		-0.300409	-0.344036
chr1	18971718	18971839		-0.300312	-0.345223
chr9	84532412	84532510		-0.300268	-0.373367
chr1	169444338	169444432		-0.300166	-0.353247
chr2	105261628	105261711		-0.30012	-0.300665
chr19	19620248	19620310		-0.300109	-0.328439
chr12	105933023	105933075		-0.300103	-0.386682
chr5	59764147	59764209		-0.300089	-0.363725
chr6	29389206	29389342		-0.300061	-0.347545
chr16	44671935	44672029		-0.300002	-0.332231
chr14	34570212	34570248	0.373496		
chr6	57534096	57534144	0.343348		
chr17	44618802	44618945	0.300531		
chr5	137683540	137683599	0.33698		
chr9	98210425	98210439	0.302091		
chrX	159415812	159415872	0.495975		
chr17	80775443	80775486	0.332886		
chr2	69128806	69128819	0.34772		
chr13	109929075	109929123	0.323115		
chr17	86710846	86710882	0.30339		
chr7	80063899	80063919	0.304544		

chr6	135363497	135363523	0.481153
chr15	28980237	28980244	0.319444
chr2	91049806	91049903	0.352196
chr6	39780821	39780840	0.38335
chr11	5954765	5954790	0.331774
chr2	57331442	57331510	0.316184
chr7	24300858	24300905	0.408625
chr7	38183448	38183481	0.736565
chr19	58194525	58194550	0.350607
chr8	9780649	9780710	0.415008
chr8	35956174	35956227	0.306118
chr7	29725864	29725993	0.369893
chr14	119307578	119307683	0.385118
chr5	119741007	119741045	0.428386
chr8	95952378	95952396	0.364418
chr15	84183292	84183312	0.331696
chr16	90044301	90044378	0.36779
chr10	116738770	116738799	0.308354
chr13	45445007	45445078	0.360363
chr4	32347242	32347260	0.345063
chr4	28912388	28912443	0.31615
chr12	85955671	85955684	0.341483
chrX	72656154	72656159	0.334921
chr1	161707281	161707325	0.324216
chr15	58075241	58075278	0.544792
chr1	66820282	66820417	0.315763
chr1	84236169	84236224	0.407867
chr2	43862702	43862748	0.337757
chr2	19447179	19447210	0.339602
chr1	86013172	86013217	0.373815
chr8	70630982	70631000	0.423067
chr2	106003702	106003733	0.398731
chr9	64227903	64227914	0.323292
chr2	170287444	170287469	0.365881
chr3	37976958	37976967	0.315351
chr9	59096910	59096957	0.342042
chr6	149566633	149566651	0.394137
chr4	46348843	46348891	0.345618
chr5	67865764	67865778	0.339587
chr3	134145284	134145316	0.39222
chr18	9210794	9210820	0.356141
chr1	189174352	189174445	0.30231
chr4	58040050	58040090	0.352834

chr9	69575688	69575792	0.348713
chr4	50369509	50369587	0.347578
chr10	87255708	87255752	0.39243
chr11	96729528	96729573	0.357768
chr5	148970112	148970157	0.311457
chr13	55826721	55826749	0.390772
chr15	48792528	48792577	0.328607
chr14	80176554	80176599	0.340726
chr17	80127354	80127359	0.310974
chr17	59792491	59792570	0.397678
chr12	69130294	69130338	0.312533
chr9	54750741	54750795	0.343435
chr4	83221314	83221355	0.312363
chr1	107506396	107506462	0.307201
chr7	128049034	128049074	0.335123
chr10	42760923	42760937	0.322557
chr3	101203570	101203595	0.30212
chr13	43515244	43515254	0.555338
chr12	12401546	12401560	0.609893
chr2	18055177	18055197	0.338202
chr15	98779972	98779985	0.440899
chr5	15506992	15507035	0.324442
chr10	108191315	108191383	0.382046
chr7	118233133	118233173	0.365459
chr11	83854199	83854216	0.342158
chr1	151010834	151010860	0.322016
chr8	114742614	114742677	0.33517
chr8	22861640	22861661	0.302249
chr18	63863651	63863663	0.316079
chr13	9796680	9796738	0.308465
chr1	84943424	84943467	0.310699
chr2	59427313	59427332	0.343531
chr9	118469161	118469226	0.314435
chr11	95143627	95143648	0.380248
chr18	53469020	53469052	0.314019
chr11	25848976	25849063	0.320213
chr3	107232323	107232415	0.337319
chr10	100017777	100017891	0.323253
chr9	64918992	64919027	0.352915
chr9	72335343	72335396	0.320945
chr7	44647269	44647283	0.480156
chr15	11969504	11969551	0.308274
chr2	77684020	77684056	0.311982

chr5	134080936	134081071	0.330742
chr9	61112254	61112270	0.395848
chr8	94913186	94913218	0.320447
chr1	191163730	191163782	0.354575
chr14	104454968	104455022	0.332905
chr17	43758845	43758860	0.654989
chr1	35698356	35698393	0.44913
chr6	21214452	21214474	0.333036
chr17	88431996	88432011	0.350814
chr5	74792963	74792994	0.316035
chr10	50903771	50903852	0.336507
chr7	71214844	71214889	0.407251
chr9	46829625	46829637	0.314613
chr8	117684773	117684796	0.439598
chr2	102146568	102146611	0.31894
chr7	28702783	28702822	0.696348
chr5	141365469	141365488	0.309458
chr18	65763974	65764051	0.342515
chr1	76973622	76973643	0.342646
chr13	108776202	108776223	0.393174
chr2	95406891	95406900	0.393634
chr8	54446658	54446677	0.318413
chr12	9576752	9576765	0.416887
chr2	8818113	8818225	0.377457
chr7	25848493	25848572	0.424671
chr1	154006073	154006102	0.447358
chr2	20445436	20445491	0.389948
chr18	67465451	67465461	0.321726
chr10	61167419	61167473	0.399437
chr9	74857114	74857131	0.461393
chr2	80314856	80314895	0.66121
chr2	126490687	126490701	0.413505
chr17	47999466	47999493	0.314381
chr11	57951931	57952010	0.429105
chr8	115133264	115133303	0.353364
chr13	69738394	69738419	0.369582
chr4	54365171	54365216	0.3217
chr7	25848632	25848716	0.368041
chr12	57550192	57550232	0.414298
chr1	160339612	160339674	0.371088
chr14	61598851	61598934	0.403458
chr14	106327902	106327936	0.370902
chr10	95685443	95685479	0.465507

chr4	102618156	102618209	0.342253	[redacted]
chr7	3318915	3318954	0.392849	[redacted]
chr15	94914863	94914885	0.304994	[redacted]
chr13	65379655	65379699	0.301358	[redacted]
chr7	39928467	39928502	0.303254	[redacted]
chr13	18949283	18949334	0.396328	[redacted]
chr10	120210045	120210079	0.346667	[redacted]
chr13	38156415	38156437	0.301549	[redacted]
chr5	115745489	115745524	0.37041	[redacted]
chr10	50897525	50897569	0.457233	[redacted]
chr2	170324419	170324568	0.381749	[redacted]
chr11	16752087	16752153	0.304006	[redacted]
chr3	83112733	83112768	0.33963	[redacted]
chrX	124125244	124125265	0.431932	[redacted]
chr2	155793493	155793554	0.449991	[redacted]
chr10	44691896	44691960	0.30593	[redacted]
chr2	75600227	75600302	0.35892	[redacted]
chr17	39843045	39843370	0.573011	[redacted]
chr13	75992800	75992934	0.335409	[redacted]
chr16	50437796	50437853	0.533231	[redacted]
chr6	124998078	124998130	0.306035	[redacted]
chr8	109464594	109464639	0.3301	[redacted]
chr9	47829103	47829112	0.381744	[redacted]
chr15	37381904	37381946	0.315297	[redacted]
chr15	25354723	25354766	0.323413	[redacted]
chr2	74912157	74912207	0.305728	[redacted]
chr9	29963032	29963108	0.359923	[redacted]
chr5	67100359	67100397	0.390197	[redacted]
chr14	34569893	34570034	0.370365	[redacted]
chr10	115185260	115185308	0.323492	[redacted]
chr11	68853769	68853784	0.304295	[redacted]
chr14	32062361	32062370	0.301674	[redacted]
chr10	93750064	93750115	0.301615	[redacted]
chr3	149305315	149305352	0.353122	[redacted]
chr8	10794408	10794467	0.342334	[redacted]
chr19	30331060	30331083	0.315281	[redacted]
chr15	81524280	81524355	0.3056	[redacted]
chr8	46281865	46281884	0.422538	[redacted]
chr16	12826457	12826470	0.443898	[redacted]
chr7	119486775	119486824	0.322937	[redacted]
chr2	131563420	131563468	0.319192	[redacted]
chr16	31347867	31347898	0.37111	[redacted]
chr15	37243366	37243421	0.310061	[redacted]

chrX	106483897	106484023	0.320707
chr13	110281244	110281249	0.317809
chr11	100571563	100571630	0.372693
chr5	128552023	128552031	0.358766
chr9	40685792	40685825	0.411306
chr18	35214865	35214895	0.330879
chr13	66715945	66715965	0.31644
chr7	127362744	127362761	0.321299
chr10	88496865	88496890	0.323161
chr6	71312660	71312700	0.420487
chr19	36918398	36918410	0.322464
chr6	137143242	137143263	0.446037
chr16	22359039	22359073	0.345654
chr5	66259612	66259643	0.446325
chr12	72441504	72441654	0.300217
chr13	112064996	112065016	0.380548
chr4	128273286	128273361	0.406497
chr19	59463985	59464017	0.525069
chrX	109715217	109715224	0.383155
chr12	87329995	87330027	0.311447
chr1	161142537	161142578	0.505928
chr16	4561207	4561227	0.388454
chr7	92837260	92837323	0.319891
chr16	49839404	49839479	0.363913
chr6	28215245	28215279	0.374102
chr14	74976533	74976551	0.423128
chr1	186552780	186552835	0.4317
chr8	23562498	23562523	0.360506
chr18	4102583	4102634	0.398918
chr17	28483834	28483858	0.317437
chr1	158133131	158133315	0.321018
chr4	44704815	44704850	0.307963
chr10	79717661	79717678	0.370615
chr12	83005721	83005770	0.360579
chr7	65517084	65517097	0.323805
chr9	13296143	13296203	0.381552
chr4	35259120	35259133	0.36294
chr12	73036376	73036457	0.367577
chr2	63969147	63969176	0.323055
chr17	27693143	27693173	0.375767
chr13	83528182	83528242	0.533936
chr2	169869693	169869724	0.380435
chr6	124108651	124108690	0.34812

chr2	69822211	69822251	0.348338
chr2	180526956	180526980	0.384396
chr15	98799403	98799444	0.320549
chrX	106595870	106595914	0.344162
chr16	65030330	65030359	0.369419
chr10	70064162	70064218	0.324967
chr13	71967991	71968039	0.40713
chr18	73122593	73122627	0.326288
chr1	171160844	171160885	0.340799
chr12	72942470	72942490	0.375591
chr10	111118517	111118618	0.340397
chr3	55517413	55517427	0.34656
chr13	47652594	47652645	0.317858
chr16	13160846	13160881	0.375731
chr11	85835826	85835844	0.333752
chr10	50891476	50891530	0.452981
chr1	92216260	92216323	0.301421
chr4	53278587	53278650	0.305173
chr2	102146672	102146719	0.316488
chr13	80921168	80921181	0.425822
chr2	32602376	32602415	0.340903
chr11	70805486	70805512	0.307335
chr9	107382250	107382320	0.425581
chr16	91347633	91347673	0.373863
chr16	87841191	87841297	0.34655
chr1	161887832	161887916	0.323891
chr3	107778230	107778250	0.493872
chr1	171466948	171467000	0.41455
chr9	107379616	107379667	0.393943
chr9	78578945	78578994	0.513683
chr14	19759926	19759946	0.48226
chr5	14669212	14669223	0.339834
chr11	102377222	102377273	0.397644
chr2	133498037	133498065	0.384462
chr10	107869162	107869189	0.356909
chr9	30285118	30285161	0.310833
chr7	62209224	62209262	0.400923
chr1	19209756	19209773	0.436364
chr12	40797075	40797187	0.328068
chr4	116222911	116222964	0.436578
chr3	148387860	148387926	0.317525
chr10	106932579	106932615	0.410636
chrX	9261556	9261632	0.511807

chr10	110795069	110795127	0.340806
chr14	76506289	76506306	0.370165
chr11	69117508	69117577	0.308315
chr2	152950948	152950986	0.312823
chr9	31290213	31290242	0.330373
chr8	112570092	112570153	0.308666
chr1	138849631	138849688	0.355879
chr19	9836732	9836770	0.408781
chr17	9633135	9633157	0.338875
chr11	18753840	18753885	0.528359
chr1	136423412	136423426	0.417728
chr2	14078839	14078870	0.332191
chr10	20197266	20197331	0.495507
chr2	9906399	9906460	0.32805
chr2	60347857	60347908	0.303552
chr3	139012523	139012548	0.331485
chr13	65379570	65379589	0.306345
chr2	154902293	154902332	0.368369
chr4	125916692	125916741	0.349296
chr11	97393886	97393901	0.301768
chr15	101858931	101858939	0.311161
chr1	25830878	25830965	0.541313
chr13	94878109	94878125	0.345458
chr15	103021615	103021671	0.453501
chr1	25178960	25179001	0.350271
chr17	39843001	39843044	0.340897
chr15	50869909	50870005	0.41066
chr15	27560920	27560965	0.333636
chr2	170287493	170287516	0.361383
chr18	15196050	15196112	0.373485
chr10	19494729	19494869	0.307857
chr6	137517129	137517138	0.388533
chr5	99869161	99869186	0.397501
chr8	106138625	106138667	0.372743
chr5	77370165	77370249	0.361853
chr2	69414578	69414641	0.348806
chr10	23960770	23960797	0.331105
chr10	120954535	120954585	0.380938
chr13	115346867	115347014	0.313893
chr19	27180574	27180618	0.344164
chr9	111417394	111417425	0.318956
chr19	30452805	30452874	0.461275
chr7	36917311	36917357	0.425796

chr6	42245667	42245706	0.368258
chr10	114831472	114831518	0.325518
chr5	65934967	65935023	0.433409
chr10	50910878	50910950	0.400529
chr1	90222534	90222563	0.332701
chr10	117628261	117628321	0.316765
chrX	135863144	135863163	0.35217
chr5	117313719	117313790	0.43587
chr12	117560795	117560823	0.547102
chr3	123350836	123350877	0.388433
chr2	79454914	79454945	0.314919
chr8	48531913	48531964	0.392473
chr7	106711545	106711601	0.347156
chr2	116984043	116984061	0.323286
chrX	7789294	7789310	0.323358
chr1	41919031	41919061	0.439273
chr12	68997048	68997091	0.304005
chr14	120352412	120352457	0.356793
chr2	137357480	137357491	0.311106
chr4	109983435	109983452	0.309838
chr15	96143957	96144007	0.330612
chr6	141255707	141255750	0.455379
chr10	115479139	115479212	0.395225
chr3	109491155	109491221	0.356308
chr10	68757337	68757379	0.495538
chr10	78480962	78480979	0.300272
chr9	41612401	41612465	0.306051
chr18	50009041	50009088	0.384166
chr3	135568545	135568578	0.485284
chr17	84073977	84073997	0.321466
chr6	83729459	83729478	0.326148
chr8	87469479	87469525	0.45129
chr11	17872529	17872602	0.383876
chr11	120047888	120047905	0.300012
chr8	121751418	121751438	0.317101
chr15	34530214	34530235	0.321594
chr17	84037984	84038069	0.352039
chr13	69482386	69482399	0.495251
chr10	87487949	87488004	0.416679
chr3	27899153	27899222	0.313319
chr9	69687813	69687839	0.33045
chr6	144209400	144209443	0.411075
chr13	107599790	107599829	0.345559

chr7	100216017	100216101	0.32676
chr18	25753698	25753715	0.319259
chr3	66546770	66546810	0.39773
chr15	76521762	76521769	0.311978
chr1	90084610	90084660	0.342298
chr11	64589084	64589114	0.302582
chr4	140367454	140367473	0.344407
chr2	79370778	79370877	0.350159
chr18	62819348	62819375	0.439091
chr3	53756614	53756653	0.339945
chrX	37095706	37095749	0.504186
chr7	16055661	16055697	0.379293
chr9	90238078	90238105	0.462271
chr6	40571512	40571545	0.457486
chrX	57110967	57110994	0.431637
chr5	147401079	147401132	0.323106
chr15	81860178	81860309	0.4698
chr12	59040219	59040243	0.305396
chr5	71700335	71700366	0.487002
chr9	59977751	59977774	0.334281
chr11	108892270	108892290	0.441414
chr3	69505213	69505250	0.493274
chr10	70651988	70652020	0.379589
chr10	69706776	69706909	0.376027
chr2	162867146	162867200	0.460266
chr8	45627490	45627515	0.384906
chr15	25135575	25135627	0.384498
chr13	101734209	101734289	0.326904
chr12	36040768	36040861	0.366914
chr7	65086169	65086188	0.50885
chr12	85288077	85288128	0.357591
chr5	122898877	122898900	0.330157
chr4	21712635	21712671	0.366358
chr2	132972242	132972288	0.332691
chr8	95952449	95952487	0.306845
chr4	33556174	33556236	0.328404
chr8	10519509	10519539	0.427691
chr4	136967682	136967724	0.352296
chrX	20379848	20379895	0.431278
chr18	37295075	37295105	0.304588
chr5	64275186	64275228	0.315301
chr4	120406245	120406270	0.321149
chr15	80934984	80935000	0.457733

chr4	75744713	75744772	0.410642	[redacted]
chr11	45799216	45799284	0.326634	[redacted]
chr16	22807808	22807836	0.354104	[redacted]
chr14	11624522	11624603	0.361774	[redacted]
chr19	31652566	31652606	0.313795	[redacted]
chr16	78303119	78303170	0.342192	[redacted]
chr14	105499833	105499848	0.536884	[redacted]
chr10	117335878	117335937	0.334238	[redacted]
chr12	33125889	33125945	0.30588	[redacted]
chr18	25505830	25505847	0.420449	[redacted]
chr13	83713565	83713624	0.300162	[redacted]
chr3	123313957	123313979	0.310586	[redacted]
chr4	101550658	101550691	0.369533	[redacted]
chr3	27709838	27709853	0.328744	[redacted]
chr8	45068806	45068859	0.323781	[redacted]
chr11	109097865	109097913	0.321609	[redacted]
chr17	48647419	48647433	0.32766	[redacted]
chr11	45847483	45847536	0.301458	[redacted]
chr16	33892417	33892462	0.490872	[redacted]
chr19	19042014	19042044	0.324338	[redacted]
chr2	74651362	74651401	0.366268	[redacted]
chr16	50237563	50237595	0.315785	[redacted]
chr5	51894294	51894311	0.306968	[redacted]
chr5	91401944	91401997	0.320496	[redacted]
chr2	84593180	84593219	0.339085	[redacted]
chr7	36692177	36692202	0.325144	[redacted]
chr3	26810904	26810913	0.342081	[redacted]
chr15	34453325	34453361	0.315278	[redacted]
chr3	17793058	17793101	0.502428	[redacted]
chr15	59501649	59501675	0.306885	[redacted]
chr13	94889352	94889394	0.328591	[redacted]
chr5	98100595	98100632	0.341758	[redacted]
chr7	4777661	4777677	0.450182	[redacted]
chr11	63413377	63413401	0.375121	[redacted]
chr15	4821948	4822018	0.332246	[redacted]
chr14	75722337	75722357	0.35147	[redacted]
chr7	18748754	18748830	0.474728	[redacted]
chr10	93225793	93225828	0.371138	[redacted]
chr7	131325652	131325713	0.366541	[redacted]
chr5	15507191	15507230	0.302102	[redacted]
chr15	10769829	10769948	0.308498	[redacted]
chr3	146275232	146275282	0.328942	[redacted]
chr4	43454014	43454059	0.435256	[redacted]

chr13	116143451	116143469	0.358509
chr13	29769881	29769953	0.351243
chr5	48286149	48286213	0.381805
chr10	96204346	96204371	0.360583
chr11	53501562	53501658	0.409262
chr14	80263388	80263469	0.333845
chr1	84933492	84933585	0.361531
chr3	5860292	5860368	0.331191
chrUn_	5159	5219	0.311346
chr3	35930121	35930246	0.3044
chr19	23272969	23272995	0.315126
chr9	101690652	101690725	0.305253
chr2	150307535	150307550	0.40178
chr6	103527089	103527177	0.312963
chr3	126770321	126770350	0.392365
chr10	120826787	120826831	0.381048
chr15	99127240	99127378	0.423505
chr11	83854423	83854448	0.355055
chr16	88290795	88290883	0.303324
chr13	22010037	22010075	0.458889
chr3	66972894	66972990	0.387489
chr9	100590663	100590749	0.344974
chr3	150610681	150610708	0.437094
chr15	55113680	55113728	0.302359
chr1	43659621	43659653	0.333741
chr10	26799945	26799968	0.365847
chr7	98977173	98977198	0.552238
chr5	119290121	119290161	0.31884
chr11	113496739	113496749	0.397086
chr11	118981353	118981375	0.407758
chr3	20198045	20198060	0.395756
chr14	51989613	51989666	0.382172
chr5	119830930	119830974	0.331793
chr11	75572739	75572830	0.435099
chr11	78826087	78826169	0.326466
chr2	137357290	137357351	0.366506
chr3	38672098	38672170	0.399263
chr5	148969979	148970009	0.310647
chr8	45884691	45884808	0.399715
chr11	82378684	82378699	0.329908
chr4	106229741	106229765	0.307446
chr11	81902989	81903052	0.304662
chr5	100217326	100217338	0.342837

chr1	36982376	36982429	0.429008
chr7	143295746	143295799	0.329049
chr10	70640101	70640188	0.316478
chr5	139841973	139842147	0.314049
chr3	53483968	53483984	0.315094
chr6	39819342	39819351	0.308502
chr4	99194838	99194895	0.309765
chr11	83572501	83572523	0.355008
chr2	79087396	79087469	0.455316
chr12	102947865	102947903	0.382572
chr14	69812800	69812815	0.400519
chr1	127836851	127836929	0.350439
chr4	65123331	65123366	0.360238
chr1	180265246	180265306	0.340121
chr8	12407835	12407894	0.370614
chr1	180848312	180848402	0.358436
chr16	89788257	89788290	0.308263
chr1	71939223	71939286	0.31035
chr11	114578880	114578933	0.397143
chr8	46641220	46641256	0.321282
chr4	12825915	12825935	0.435528
chr18	79327455	79327590	0.393594
chr12	79381237	79381303	0.309026
chr10	19939813	19939831	0.325991
chr12	104997226	104997254	0.31062
chr7	130326011	130326020	0.301626
chr18	68692098	68692242	0.345373
chr1	24302275	24302300	0.373603
chr5	147956369	147956396	0.34987
chr15	51136545	51136584	0.347364
chr9	99836969	99837015	0.333864
chr11	114234359	114234400	0.346918
chr15	11566063	11566079	0.301724
chr11	52764131	52764187	0.316566
chr5	148728164	148728233	0.407124
chr7	36684689	36684746	0.349906
chr2	169298001	169298032	0.503705
chr10	14018680	14018734	0.326426
chr16	95584988	95585009	0.461
chrX	37095948	37096004	0.333968

Supplementary Information 5 - primer sequences

Mouse RT q-PCR

Igf1-F	CGCTCTGCTTGCTCACCTTCAC
Igf1-R	CACTCATCCACAATGCCTGTCTG
β-actin-F	GACGGCCAGGTCATCACTATT
β-actin-R	AGGAAGGCTGGAAAAGAGCC

Mouse Cobra primers

Il2rb-F	GTTTGATGGTTAATGAGTGGTTTTT
Il2rb-R	CTTATCATATTTCCCCAAACCTAA
Ppil1-F	TTAAAAGTAAAGAGTATGTGGAAAGAGG
Ppil1-R	CCTACATTTCCAACTCCAAAAATAC
Coro2a-F	GAGTTAAGGTTAGTTTAGGTTATAGAGTGA
Coro2a-R	AAACTTCTAACTTCCATAAAACTCC
